# The genus *Lycianthes* (Solanaceae, Capsiceae) in Mexico and Guatemala

**DOI:** 10.3897/phytokeys.168.51904

**Published:** 2020-11-27

**Authors:** Ellen Dean, Jennifer Poore, Marco Antonio Anguiano-Constante, Michael H. Nee, Hannah Kang, Thomas Starbuck, Annamarie Rodrígues, Matthew Conner

**Affiliations:** 1 UC Davis Center for Plant Diversity, Plant Sciences M.S. 7, One Shields Ave., Davis, CA 95616, USA University of California Davis United States of America; 2 Laboratorio Nacional de Identificación y Caracterización Vegetal (LaniVeg), Consejo Nacional de Ciencia y Tecnología (CONACyT), Centro Universitario de Ciencias Biológicas y Agropecuarias, Universidad de Guadalajara, Camino Ramón Padilla Sánchez 2100, 45110 Nextipac, Zapopan, Jalisco, México Universidad de Guadalajara Guadalajara Mexico; 3 26776 US Hwy 14, Richland Center, WI 53581, USA Unaffiliated Richland Center United States of America

**Keywords:** Guatemala, *
Lycianthes
*, Mexico, Neotropics, Solanaceae, Taxonomy

## Abstract

*Lycianthes*, the third most species-rich genus in the Solanaceae, is distributed in both the New and Old Worlds and is especially diverse in Mexico. Here we provide an identification key, taxonomic descriptions, distribution maps, and illustrations of specimens, trichomes, flowers, and fruits for the 53 known *Lycianthes* taxa of Mexico and Guatemala. The new combination *Lycianthes scandens* (Mill.) M.Nee is made and replaces the name *Lycianthes lenta* (Cav.) Bitter, which is placed in synonymy. Within *L. scandens*, two varieties are recognized (Lycianthes scandens var. scandens and Lycianthes scandens var. flavicans (Bitter) J.Poore & E.Dean, **comb. nov.**). In addition, one new species (*Lycianthes rafatorresii* E.Dean, **sp. nov.**) is described from eastern Mexico, and 10 names (either recognized taxa or synonyms of recognized taxa) are lectotypified, including the names *Solanum heteroclitum* Sendtn., *S. rantonnetii* Carrière, and *S. synantherum* Sendtn. The species *L. multiflora* Bitter and *L. synanthera* (Sendtn.) Bitter are excluded from the treatment, as research indicates that they do not occur in Mexico and Guatemala, however full synonymy for both names is given.

## Introduction

**Figure 1.**
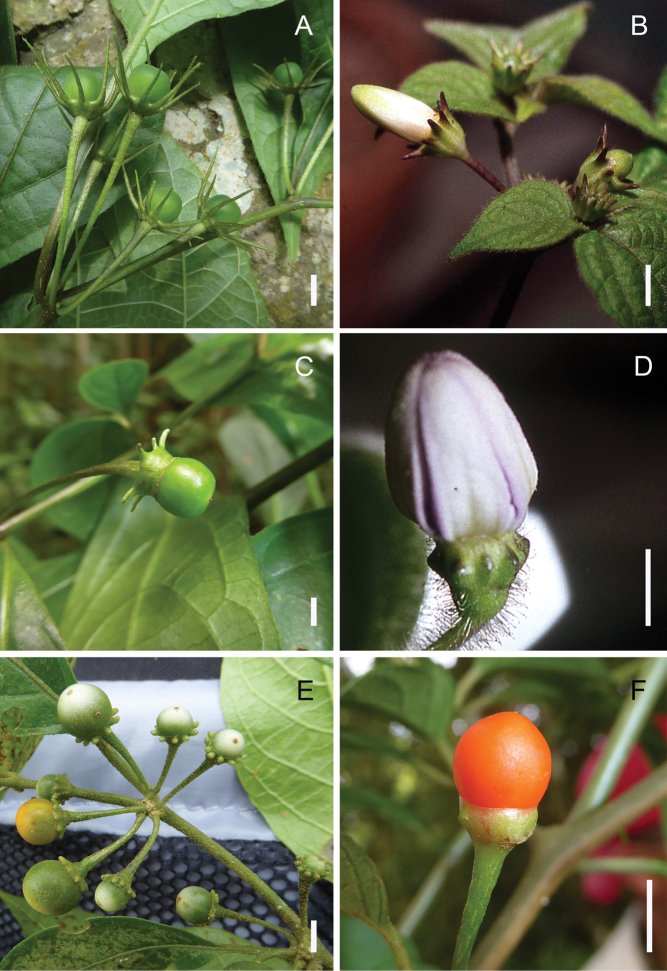
*Lycianthes* calyces **A** green, immature fruits and relatively long calyx appendages in *L. mariovelizii* E. Dean, field photo of *Dean 9509* (DAV) **B** flower buds and relatively short calyx appendages in *L. jalicensis* E. Dean, field photo of *Dean 248* (DAV) **C** green, immature fruit and calyx with long, sleeve-like calyx margin and connate appendage bases in *L. connata* J.L. Gentry, field photo of *Dean 9530* (DAV) **D** flower bud and glandular pubescent calyx with very short, nearly absent appendages (appearing as oval bulges) and scarious calyx margin with undulating rim in *L. pringlei* (B.L.Rob. & Greenm.) Bitter, field photo of *Dean 327* (DAV) **E** immature pale green to yellow fruits and calyces with obovate appendages with rounded tips in *L. sideroxyloides* (Schltdl.) Bitter, field photo of *Dean 9526* (DAV) **F** mature fruit with calyx nearly lacking appendages (present as short bulges) in *L. ceratocalycia* (Donn.Sm.) Bitter, field photo of *Dean 9532* (DAV). Scale bars: 5 mm.

*Lycianthes* (Dunal) Hassler (Capsiceae, Solanaceae) with approximately 150–200 species (D’Arcy 1991; Hunziker 2001) is the third largest genus in the Solanaceae, after *Solanum* L. and *Cestrum* L. (Hunziker 2001). Although *Lycianthes* is native to both the New and Old Worlds, the center of distribution of the genus, and the majority of *Lycianthes* taxa, are found in the New World (from northern Mexico to Argentina as well as the Caribbean). The genus is recognized by its combination of poricidal anthers and a distinctive calyx morphology in which the five calyx lobes are truncated into a sleeve-like rim; in many *Lycianthes*, appendages (sometimes called calyx teeth) protrude below the calyx rim (D’Arcy 1986a) (Fig. 1). Calyx appendages are also present in *Lycianthes*’s closest relative (and only other member of tribe Capsiceae) *Capsicum* L. (Bitter 1919; Särkinen et al. 2013). When in fruit (and anthers are lacking), *Lycianthes* can be confused with *Capsicum*, as well as other Solanaceous genera with truncated calyces and no appendages (*Cuatresia* Hunz. and *Witheringia* L’Hér.).

The only taxonomic monograph of *Lycianthes* as a whole was written in the early 20^th^ century by the German botanist Friedrich August Georg Bitter (1919); that work includes 189 terminal taxa (species, subspecies, and varieties) divided into subgenera, sections, and series, with no identification keys to assist in distinguishing the species. Although Bitter’s monograph was published in volume 24 of the journal *Abhandlungen herausgegeban vom Naturwissenschaftlichen Verein zu Bremen* with a volume date of 1920, both Band 1 and Band 2 of volume 24 were preprinted in November 1919 and the pagination throughout the volume is continuous (Tropicos 2020), leading to confusion as to the date to cite for the species described in his monograph (1919, 1920, or 1919(1920)). The correct date for publication of all names and combinations published therein is 1919.

### Early taxonomic history of the genus *Lycianthes*

Georg Bitter’s 1919 monograph provides a detailed account of the taxonomic history of *Lycianthes* which was also recounted in an unpublished dissertation thesis by Dean (1995); we are publishing Dean’s 1995 summary here. The genus was included in *Solanum* until the early part of the 20^th^ century. The first person to work with the species as a group was the French botanist Michel Félix Dunal, who monographed *Solanum* in the early 19^th^ century (Dunal 1813, 1816). In his earliest work, he placed a number of present-day Lycianthes species together in his section Inermia, group *Polymeris*, based upon the presence of calyx appendages and the many separate axillary pedicels common in *Lycianthes* (Dunal 1813, 1816; Knapp 1983). His group *Polymeris* was given sectional status in *Solanum* by the German botanist Wilhelm Gerhard Walpers (1844), who added two more sections with future *Lycianthes* species: *Lycioides* and *Holochlaina*. In a much expanded treatment published in De Candolle’s Prodromus, Dunal combined Walpers’ three sections into his subsection Lycianthes (Dunal 1852). That subsection comprised two series (*Meiomeris* and *Polymeris*) and four subseries (*Eulycianthes*, *Pseudolycianthes*, *Gonianthes*, and *Lobanthes*) (Dunal 1852). The Austrian botanist Richard Von Wettstein, in his generic level treatment of *Solanum* for *Die natürlichen Pflanzenfamilien* (Von Wettstein 1895), raised *Lycianthes* to a section of *Solanum*, and Bitter accorded it subgeneric status in 1917 (Bitter 1917).

The Swiss botanist Emil Hassler raised *Lycianthes* to generic level in 1917, transferring three species of Dunal’s subseries *Eulycianthes* (Hassler 1917). The evidence used to justify the creation of the genus came from anatomical work performed by both Hassler and Bitter; all three of the transferred species have fruits with stone cells in their endocarps. In his later comprehensive monograph of *Lycianthes*, Bitter (1919) expanded the circumscription of the genus to include most members of Dunal’s subsection Lycianthes, asserting that the calyx of these species places them closer to *Capsicum* than to *Solanum*. During the latter part of the 20^th^ century, some authors continued to treat *Lycianthes* as part of *Solanum* (Morton 1944, 1976; Hunziker 1979; Rzedowski 1985; Symon 1985a, b, 1987).

Two names for the concept of *Lycianthes* predate the name *Lycianthes* in the literature. The American botanist Constantine Samuel Rafinesque published the name *Otilix* (Rafinesque 1828) based on *Solanum lycioides* L., but this new genus was lost to obscurity and not utilized by subsequent authors. Subsequently, the French botanist Henri Ernest Baillon (1888) published the genus *Parascopolia* based upon a single collection made in Acapulco, Mexico by the phycologist Charles Thiébaut (*P. acapulcensis* Baill.). It is unclear why Baillon published this new genus since, as pointed out by Bitter (1919), Dunal had clearly accounted for similar species in his subsection Lycianthes. In his description of *Parascopolia*, Baillon noted the 8–10 calyx teeth, a feature that clearly indicates that this taxon belongs with other species of *Lycianthes*. His description of the anther dehiscence as “2-rimosis” was interpreted by most workers as meaning longitudinal anther dehiscence. Bitter assumed that *P. acapulcensis* belonged to *Lycianthes* but was not able to see the type at Paris (Bitter 1919). Unable to clear up the anther dehiscence confusion, he delayed synonymizing *Lycianthes* with *Parascopolia* (Bitter 1919). After seeing a photo of *P. acapulcensis*, William D’Arcy proposed the inclusion of this species in *Lycianthes* and the conservation of *Lycianthes* over *Parascopolia* and *Otilix* (D’Arcy 1972); this was approved in 1973 (McVaugh 1973). The anthers of the flowers of the type of *Parascopolia acapulcensis* were finally examined by Dean (1995), and she confirmed that the anthers had small, linear, terminal pores, not longitudinal slits.

### Taxonomic work on New World *Lycianthes* since Bitter (1919)

Since Bitter’s monograph, many additional New World *Lycianthes* taxa have been described (e.g., Bitter 1924; Standley 1927a, b, 1940; Standley and Steyermark 1947; Gentry 1948; Gentry 1973; Dean 1994, 1998; Rodríguez and Vargas 2002; Dean 2004, 2014; Dean et al. 2017b, 2018b, c, 2019a) and transferred to the genus (e.g., Bitter 1922; D’Arcy 1986b). Keys and species-level treatments for the genus have been included in a number of floristic works, including those for parts of Mexico (Rzedowski 1985 [as *Solanum*]; Nee 1986), Guatemala (Gentry and Standley 1974), Nicaragua (D’Arcy 2001), Costa Rica (Standley and Morton 1938; Bohs 2015), Panama (Standley 1928; D’Arcy 1973a), Venezuela (Benítez de Rojas and D’Arcy 1997), Peru (MacBride 1962 [as *Solanum*]), and Argentina (Barboza and Hunziker 1992; Barboza 2013); in addition, an identification key for upcoming species treatments has been published recently for the species of Brazil (Costa-Silva and Agra 2018b), and a summary of the Brazilian species, including lectotypifications, has also been completed (Costa-Silva 2018; Costa-Silva and Agra 2018a).

Morphological revisions have been completed on five of Bitter’s *Lycianthes* sections or series. These are series *Meizonodontae* Bitter (Dean 1995, 2004), series *Microlobae* Bitter (Dean et al. 2007), section Synantheroides Bitter (Reyes Cornejo 2015), series *Tricolores* Bitter (Dean et al. 2017a, 2018c), and series *Piliferae* Bitter (Dean et al. 2019b). Phylogenetic analyses using morphological characters have been completed by Dean (1995) for series *Meizonodontae* and Reyes Cornejo (2015) for section Synantheroides. The published work based on morphology indicates that the species united by the sections and series proposed by Bitter may be closely related in a least two groups (series *Meizonodontae* and series *Tricolores*), while other groups (series *Piliferae* and section Synantheroides) contain a mixture of closely and distantly related species.

To date, a phylogeny of the entire genus has not yet been published, although work on this project using molecular markers is ongoing at the laboratory of Lynn Bohs at the University of Utah, and preliminary results are completed. Research into the evolution of *Lycianthes* series *Meizonodonatae* using molecular markers and an analysis of the biogeography of series *Meizonodonatae* has been completed by the third author at the University of Guadalajara (Anguiano-Constante et al. 2018; Anguiano-Constante 2019). An investigation of the morphology and phylogeny (using molecular markers) of the mostly South American series *Strigosulae* Bitter is also ongoing at the Universidad Nacional Mayor de San Marcos in Peru (Cueva-Manchego no date). In addition, a number of species of *Lycianthes* have been included in phylogenetic studies of the Solanaceae (Särkinen et al. 2013), and this partial analysis of the species indicates that *Lycianthes* may be a paraphyletic group in relation to *Capsicum* (Spalink et al. 2018), and if monophyly is preferred, the genus may need to be broken into smaller groupings or combined with *Capsicum*.

Herbarium collections of many of the species of *Lycianthes* are not numerous, perhaps because many of the species have flowers that close during the day, making them inconspicuous (Nee 1981). In our experience, many *Lycianthes* species are difficult to locate, often occurring singly or in small populations, which may be another reason why they are poorly collected. This lack of material, along with the problems inherent in dealing with an older monograph such as Bitter’s, have perhaps contributed to the lack of attention given to *Lycianthes*. It is likely that there are many new species of *Lycianthes* awaiting discovery in herbaria and in the field.

### Goals of this paper

Over the past four years, the first author has been investigating the *Lycianthes* of Mexico and Central America as part of National Science Foundation-funded research that will produce species descriptions that will be posted at the website Solanaceae Source (http://solanaceaesource.org/). Mexico, known for its high plant species diversity and endemism (Myers et al. 2000; Villaseñor 2016; Sosa et al. 2018), is especially rich in *Lycianthes* taxa (48 recognized here; Table 1), likely the highest number of *Lycianthes* taxa of any country in the Neotropics. However, a floristic treatment of the genus has never been completed for the entirety of Mexico, and a summary of what is known about the Mexican species is overdue. In this paper, we provide species descriptions and synonymy (as well as necessary lectotypifications) for all the *Lycianthes* taxa of Mexico, as well as an identification key, photographs of specimens, illustrations of trichomes, flowers, fruits, and seeds, and distribution maps. As the Mexican state of Chiapas shares many *Lycianthes* species with neighboring Guatemala, we are also treating the Guatemalan species (Table 1) and updating the treatment of *Lycianthes* completed for Guatemala over forty years ago (Gentry and Standley 1974). At the end of the treatment, we also summarize difficult to place specimens and excluded taxa.

**Table 1.** Geographic distribution of the native *Lycianthes* of Mexico and Guatemala, excluding the cultivated species *L. rantonnetii*.

Species	Distribution
*L. acapulcensis* (Baill.) D’Arcy	Mexico to Costa Rica
*L. amatitlanensis* (J.M.Coult. & Donn.Sm.) Bitter	Mexico to South America
*L. anomala* Bitter	Mexico
*L. armentalis* J.L.Gentry	Mexico, Guatemala, Belize
*L. arrazolensis* (J.M.Coult. & Donn.Sm.) Bitter	Mexico to Nicaragua
*L. barbatula* Standl. & Steyerm.	Mexico, Guatemala
*L. breedlovei* E.Dean	Mexico
*L. caeciliae* Bitter	Mexico
*L. ceratocalycia* (Donn.Sm.) Bitter	Mexico, Guatemala
L. chiapensis (Brandegee) Standl. var. chiapensis	Mexico, Guatemala
L. chiapensis (Brandegee) Standl. var. sparsistellata Standl. & Steyerm.	Mexico to Nicaragua
*L. ciliolata* (M.Martens & Galeotti) Bitter	Mexico, Guatemala
*L. connata* J.L.Gentry	Mexico, Guatemala
*L. cuchumatanensis* J.L.Gentry	Guatemala
*L. dejecta* (Fernald) Bitter	Mexico
*L. fredyclaudiae* E.Dean	Guatemala
*L. geminiflora* (M.Martens & Galeotti) Bitter	Mexico
*L. glabripetala* E.Dean	Mexico
*L. gongylodes* J.L.Gentry	Guatemala
*L. gorgonea* Bitter	Mexico, Guatemala, Belize
*L. grandifolia* E.Dean	Mexico
*L. heteroclita* (Sendtn.) Bitter	Mexico to South America
*L. hintonii* E.Dean	Mexico
*L. hypoleuca* Standl.	Mexico to Honduras
*L. inconspicua* Bitter	Guatemala to Panama
*L. jalicensis* E.Dean	Mexico
*L. limitanea* (Standl.) J.L.Gentry	Mexico, Guatemala, Belize
*L. manantlanensis* Aarón Rodr. & O.Vargas	Mexico to El Salvador
*L. mariovelizii* E.Dean	Mexico to Nicaragua
*L. michaelneei* E.Dean	Mexico
L. moziniana (Dunal) Bitter var. margaretiana E.Dean	Mexico
L. moziniana (Dunal) Bitter var. moziniana	Mexico
L. moziniana (Dunal) Bitter var. oaxacana E.Dean	Mexico
*L. nitida* Bitter	Mexico to Panama
*L. ocellata* (Donn.Sm.) C.V.Morton & Standl.	Mexico and Guatemala
*L. orogenes* Standl. & Steyerm.	Mexico and Guatemala
*L. peduncularis* (Schltdl.) Bitter	Mexico
*L. pilifera* (Benth.) Bitter	Mexico
*L. pringlei* (B.L.Rob. & Greenm.) Bitter	Mexico
*L. purpusii* (Brandegee) Bitter	Mexico to Honduras
*L. quichensis* (J.M.Coult. & Donn.Sm.) Bitter	Mexico and Guatemala
*L. rafatorresii* E.Dean	Mexico
*L. rzedowskii* E.Dean	Mexico
L. scandens (Mill.) Nee var. flavicans (Bitter) J.Poore & E.Dean	Mexico to Costa Rica
L. scandens (Mill.) Nee var. scandens	Mexico to South America and the Caribbean
*L. sideroxyloides* (Schltdl.) Bitter	Mexico to Nicargua
*L. starbuckii* E.Dean	Mexico
*L. stephanocalyx* (Brandegee) Bitter	Mexico to Honduras
*L. surotatensis* Gentry	Mexico
*L. textitlaniana* E.Dean	Mexico
*L. tricolor* (Dunal) Bitter	Mexico to El Salvador
*L. venturana* E.Dean	Mexico

## Materials and methods

The circumscriptions of the species treated here are based on examination of herbarium specimens, cultivated plants, and field observations and are supported by morphological evidence. We examined specimens from the following herbaria either in person or as images: A, ANSM, ARIZ, ASU, BIGU, BRIT, BM, BR, BREM, C, CAS, CIIDIR, CR, DAV, DUKE, E, F, G, GBH, GH, GOET, HAL, HBG, HCIB, IBUG, IEB, INBIO, JE, K, LD, LE, LL, M, MA, MEXU, MICH, MO, MPU, MSB, MSC, NDG, NY, P, PH, SERO, TEX, U, UC, UCR, US, VT, W, WIS, WU, XAL, Z, and ZEA (herbarium codes follow Thiers 2019).

Throughout this work, type specimens with a known barcode number are cited with the herbarium code followed by the number (for example: holotype: P [P00070402]). In cases where the specimen has no barcode number (or the herbarium wishes the accession number to be used instead, for example F), the accession number is provided (preceded by “acc. #”). If no number is cited for a type specimen, none was provided on the specimens or was unavailable to the authors. Nearly all type specimens cited were examined as either a digital photo or in person by one of the authors; therefore, herbarium codes are not followed with an exclamation mark. If a specimen was not seen by us, we indicate this. When lectotypes are designated in the nomenclature section of a species treatment, remarks justifying the choice of the lectotype are included in the commentary section of that same treatment.

Specimens examined are listed in Appendix I. We also chose representative specimens from the Mexican/Guatemalan distribution of each taxon (one specimen from each state/department where it occurs) and listed them at the end of each taxon treatment. Specimens in these sections are organized alphabetically first by country, then by state/department, then alphabetically by collector’s surname, and then numerically by collection number. No barcode or accession numbers are cited. Because the first author saw and confirmed the identification of all specimens listed in Appendix I and the “Representative Specimens Examined” sections, we did not use exclamation points to distinguish the specimens that we examined.

In order to create maps of the Mexican species, specimens were georeferenced by using either Geolocate, an online software-mapping package (Rios and Bart 2010), or manually, using Google Earth Pro (Sullivan 2009); for Mexican specimens, the latter was often used in conjunction with location data found in the Mexican Archivo Histórico de Localidades [The Archive of Mexican Historical Locations] (INEGI 2010). In lists of specimens examined, when coordinates were added to a specimen record, we provide those coordinates in brackets; if the coordinates are not in brackets, they were provided on the specimen label.

The georeferenced location data were analyzed in order to provide conservation assessments for each species using GeoCAT (Bachman et al. 2011); we calculated the Extent of Occurrence (EOO) and the Area of Occupancy (AOO) in km^2^, assuming cells of 2 km on a side. Preliminary assessment categories were proposed following the criteria of the International Union for Conservation of Nature (IUCN 2019).

Our species concept is a morphological one (Cronquist 1978), based on measurement of herbarium specimens and field and greenhouse observations. In separating the species, we emphasize discontinuities in habit, pubescence, leaf, floral, fruit, and seed characters that are well preserved on herbarium specimens. The species recognized in this paper are well-defined entities. As discussed below, we are recognizing 53 taxa, including 49 species and seven varieties, from Mexico and Guatemala.

### Morphology and distribution

**Habit**

**Figure 2.**
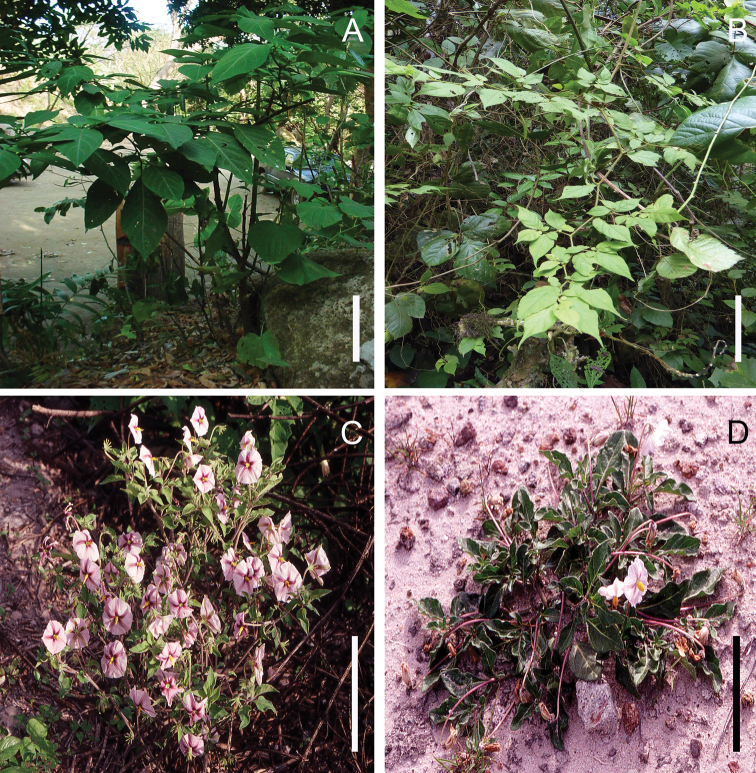
*Lycianthes* habit **A** free-standing shrub habit in *L. heteroclita* (Sendtn.) Bitter, field photo taken by the first author at El Chocoyero, Nicaragua **B** vine habit in *L. gorgonea* Bitter, field photo of *Dean 9528* (DAV) **C** upright herb habit in *L. ciliolata* (M.Martens & Galeotti) Bitter, field photo of *Dean 225* (DAV) **D** prostrate herb habit in *L. peduncularis* (Schlechtd.) Bitter, field photo of *Dean 230* (DAV). Scale bars: 10 cm.

*Lycianthes* life forms (all perennial) include herbs, vines, shrubs, and treelets, with a few species epiphytic (Fig. 2). Most species are shrubs or vines (Fig. 2A, B); many species are intermediate between the two life forms and described as scandent shrubs to vines. Some shrubs, such as *L. jalicensis*, are rhizomatous and form clonal thickets (illustrated in Dean et al. 2017a). A minority of the species are true herbs (Fig. 2C, D). The eight species of series *Meizonodontae* Bitter (*L. acapulcensis*, *L. ciliolata* (Fig. 2C), *L. dejecta*, *L. hintonii*, *L. moziniana*, *L. peduncularis* (Fig. 2D), *L. rzedowskii*, and *L. starbuckii*) are true herbs, arising from and dying back to tuberous roots annually, only appearing above-ground with the commencement of the rainy season (Dean 2004). Depending on climate and habitat, *L. stephanocalyx* can grow as a rhizomatous herb, dying back to rhizomes annually, or it can persist above ground from season to season becoming woody at the base (illustrated in Dean et al. 2019b). *Lycianthes heteroclita* (Fig. 2A), *L. geminiflora*, and *L. ceratocalycia* have green, herbaceous primary stems when young but become shrubs or treelets with age; thus those three species are sometimes described on specimen labels as herbs. *Lycianthes amatitlanensis*, *L. glabripetala*, and *L. inconspicua* are very short subshrubs that are often described as herbs, although they are generally woody and persist above ground. Two species in Mexico and Guatemala are nearly always epiphytic (*L. anomala* and *L. nitida*), and *L. heteroclita* sometimes sprouts on the branches of trees but is usually found rooted in the ground.

The sympodial branching pattern of *Lycianthes* is similar to that of *Solanum* and has been described and illustrated by Child and Lester (1991), Bohs (1994), and Dean (2004). In both herbs and shrubs, initial stem growth begins with an upright sympodial unit (or trunk) with multiple spirally-arranged leaves; the trunk usually terminates in an inflorescence. Growth continues by the production of upper sympodial units that emerge either singly (monochasial branching) or in pairs (dichasial branching) beneath the inflorescence of the prior sympodial unit. If the plant senesces or is damaged, the entire growth cycle can begin again from axillary buds, with the first sympodial unit duplicating the form, leaf number, and leaf arrangement of the trunk. In the species descriptions, we provide information on the length and internode number of the first sympodial unit for the true herbs of series *Meizonodontae*, because it is of significant length and a highly visible portion of the mature plant in most of the species. In contrast, we only provide information on the upper sympodial growth of all the other species (shrubs and vines).

**Indument**

**Figure 3.**
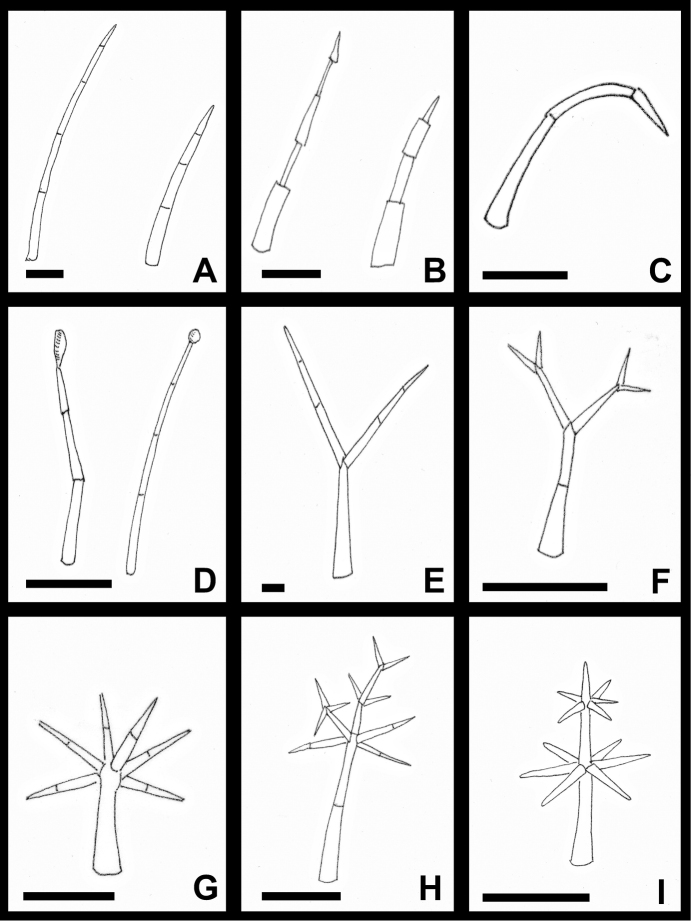
*Lycianthes* trichomes **A** simple trichomes that do not flatten upon drying: left, in *L. purpusii*, from *Beaman 5130* (NY); right, in *L. pilifera*, from *Lorence 4035* (CAS) **B** simple trichomes that flatten upon drying: left, with flattened cells oriented alternately at right angles to one another in *L. quichensis*, from *Breedlove 31746* (CAS); right, with flattened cells not oriented at right angles to one another in *L. acapulcensis*, from *Dean 249* (DAV) **C** simple, curved trichome that does not flatten upon drying in *L. tricolor*, from *Dean 297* (DAV) **D** glandular, simple trichomes: left, glandular trichome with ovoid glandular tip in *L. textitlaniana*, from *Zarate Marcos AZM-274* (MEXU); right, glandular trichome with globose tip in *L. surotatensis*, from *Rzedowski 43384* (DAV) **E** furcate trichome in *L. purpusii*, from *Beaman 5130* (NY) **F** dendritic trichome in *L. dejecta*, from *Dean 261* (DAV) **G** multangulate-stellate trichome in *L. armentalis*, from *Breedlove 56055* (MO) **H** multangulate-stellate trichome with rebranched rays in *L. breedlovei*, from *Breedlove 34793* (MO) **I** geminate-stellate trichome in *L. sideroxyloides*, from *Dean 9526* (DAV). Scale bars: 0.25 mm.

Trichome type is important in the identification of many *Lycianthes* species; trichome density, however, can be quite variable within a species. The trichome types of the *Lycianthes* of Mexico and Guatemala include simple (Fig. 3A–D), furcate (Fig. 3E), dendritic (Fig. 3F), true stellate (uncommon in the species covered in this paper and not illustrated here, however illustrations are available, for example in Payne (1978 [fig. 43]) or Harris and Harris (1994 [fig. 1492]), multangulate-stellate (Fig. 3G, H), and geminate-stellate (Fig. 3I)). All trichomes are uniseriate (one-cell wide). Trichome terms are taken from Roe (1968, 1971) and follow current usage in the Solanaceae (Sampaio et al. 2014), although some current papers use the term *multi*angulate trichome rather than *mult*angulate trichome (used by Roe and used here).

Furcate (forked) trichomes (Fig. 3E) resemble simple trichomes but are forked at the tip. Dendritic trichomes (Fig. 3F) are repeatedly forked with no more than two branches per node. Stellate, multangulate-stellate (Fig. 3G, H), and geminate-stellate (Fig. 3I) trichomes have more than two branches per node, and these whorled branches are called rays. True stellate trichomes are uncommon in the species included here (mainly seen in *Lycianthes hypoleuca*); true stellate trichomes have all rays emerging at just one node, with the rays in one plane. Multangulate-stellate trichomes have all rays emerging at just one node, but the rays are in more than one plane. In several species, the rays of the multangulate-stellate trichomes may be rebranched (Fig. 3H) (Dean et al. 2019a). In the species that have multangulate-stellate trichomes with rebranched rays, trichomes with both unbranched and rebranched rays are present, and the intermediate forms can be seen, which is why we do not refer to these trichomes as dendritic. Geminate-stellate trichomes have rays emerging at numerous consecutive nodes; in the species covered in this paper, geminate-stellate trichomes often have numerous rays per node (more than five) and the rays are relatively short (Fig. 3I). We are using the term geminate-stellate trichomes, as we are following the terminology of Roe (1968, 1971); however, some authors refer to these trichomes as candelabra trichomes (Payne 1978).

In some species with simple trichomes, the cells collapse and flatten upon drying, appearing like a flattened ribbon (Fig. 3B); in some species, the adjoining flattened cells are oriented to one another at 90 degree angles (e.g. *L. moziniana* and *L. quichensis*) (Fig. 3B). In contrast, some species have trichomes that remain cylindrical with a pointed tip (e.g. *L. amatitlanensis*, *L. inconspicua*, *L. glabripetala*, and *L. pilifera*) (Fig. 3A). In most species with furcate, dendritic, and the various types of stellate trichomes, the cells do not collapse, but remain cylindrical upon drying. An exception to this is the dendritic to multangulate-stellate trichomes of *L. dejecta*, which do flatten upon drying.

Only four species included here have glandular trichomes (Fig. 3D) as a major component of stem, leaf, and/or inflorescence pubescence (*L. surotatensis*, *L. textitlaniana*, *L. pringlei*, *L. gorgonea*). In all cases, these species have simple trichomes with a globose (*L. suratotensis*, *L. gorgonea*) to ovoid (*L. pringlei*, *L. textitlaniana*) glandular tip. Minute glandular trichomes are sometimes located inside the calyces or at the tips of the corolla lobes in some species, but these can only be seen with high magnification.

**Leaves**

The upper sympodial units of the *Lycianthes* covered here are difoliate. In many species, the leaves are in geminate pairs, often with one leaf smaller than the other, sometimes with the leaves of very different sizes and shapes. In some species, the smaller leaf fails to develop, and the sympodia appear to be unifoliate. The leaves are simple, usually petiolate, and have mostly entire but undulate margins. In a few populations of a few species the margin can sometimes have irregular, sparse, coarse dentations (e.g. *L. tricolor* and *L. surotatensis*), but this characteristic is uncommon and unpredictable. The texture of the leaf blades ranges from membranaceous to coriaceous.

**Inflorescence**

The sympodial units of *Lycianthes* terminate in inflorescences. The majority of inflorescences are located on the upper sympodia and appear axillary. The peduncle in most *Lycianthes* is reduced and usually not visible, resulting in an umbellate inflorescence of one to many pedicelled flowers; in a minority of species (e.g. *L. amatitlanensis*, *L. nitida*), a short peduncle is present, often covered with the many pedicel scars of fallen flowers. In a minority of the species, the inflorescence is always a solitary flower (e.g. all the herb species of series *Meizonodontae*, *L. stephanocalyx*, *L. textitlaniana*, *L. amatitlanensis*, *L. gorgonea*); all the other species can have one to many flowers. However, usually only one to a few flowers at a time are present at a single axil.

**Pedicels**

The pedicels of the flowers can be erect, ascending, spreading, or reflexed. It is common for the pedicel to be recurved at the tip as the flower is developing (this is the case in the herbs of series *Meizonodontae*). In other species, the pedicel is completely erect, and the flower is oriented upwards as it develops (e.g. *L. heteroclita*). In *L. amatitlanensis* and its close relatives *L. glabripetala* and *L. inconspicua*, the pedicels are usually deflexed and held beneath the leaves with the flowers nodding beneath the plant.

**Calyx**

In all *Lycianthes*, there are no lobes on the synsepalous calyx; rather the calyx is a cup-shaped structure with a truncate rim. Below this rim, calyx appendages may emerge (Fig. 1A, B). In most species, the length of the calyx margin between the rim and the appendages is short, less than 0.5 mm long. In a few species, the margin is conspicuous, as a long as 2 mm long (e.g. *Lycianthes connata* [Fig. 1C]). A very few species have an undulate calyx rim, sometimes with a large papery margin, or a rim that tears with age (e.g. *L. pringlei* [Fig. 1D], *L. manantlanensis*, *L. limitanea*); in these cases, the margin can sometimes resemble unequal calyx lobes. Appendages are usually linear in shape, sometimes subulate (wider at the base and very pointed at the tip, e.g. *L. pilifera* [illustrated in Fig. 8B of Dean et al. 2019b]), more rarely widely obovate with a broadly rounded tip (e.g. *L. sideroxyloides* [Fig. 1E], *L. ocellata*) or linear with an enlarged and bulbous tip (e.g. *L. rafatorresii*). In a minority of the species, appendages are lacking completely (e.g. *L. heteroclita*, *L. geminiflora*, *L. nitida*) or can be reduced to mere bumps on the calyx (e.g. *L. pringlei* [Fig. 1D], *L. ceratocalycia* [Fig. 1F]). In two of the species included here (*L. anomala*, *L. connata*), the appendages are connected to one another at their base, forming a shelf that reflexes as a unit as the flower ages and the fruit develops (Fig. 1C).

**Corolla**

**Figure 4.**
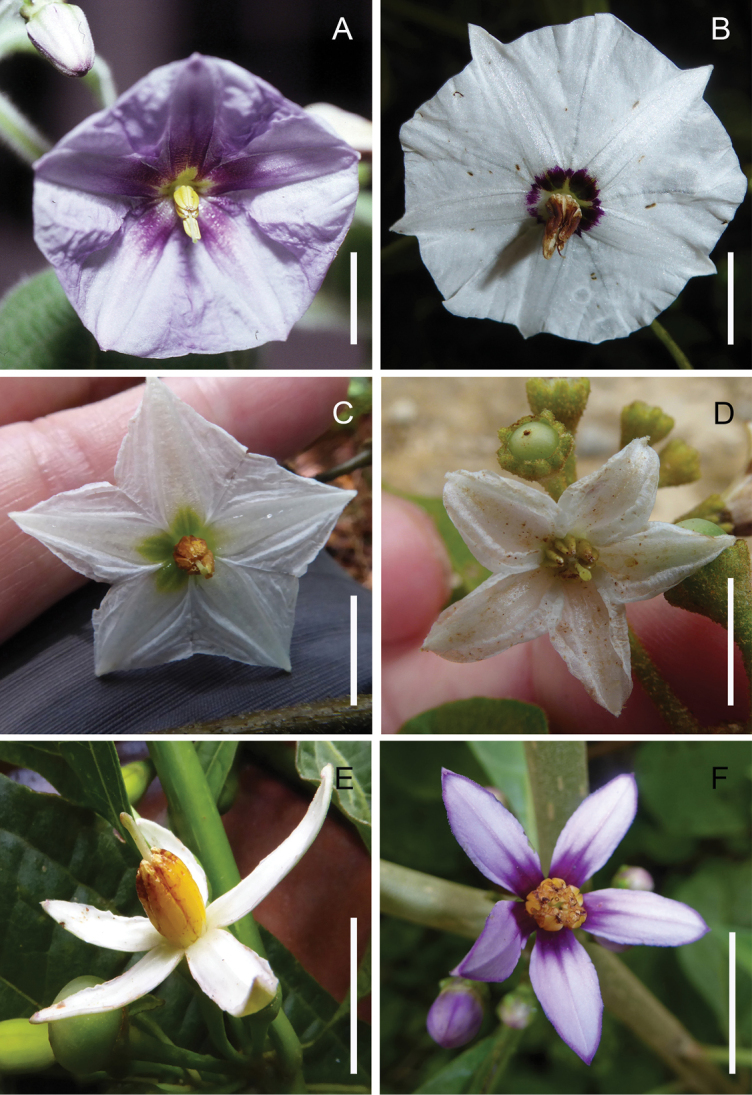
*Lycianthes* corollas and stamens **A** flower of *L. pringlei* with corolla slightly campanulate to rotate in orientation, entire in outline, abundant interpetalar tissue, and green markings at the base of the lobes, the stamens unequal and free; field photo of *Dean 327* (DAV) **B** flower of *L. connata* with corolla rotate in orientation, very shallowly stellate in outline, abundant interpetalar tissue (with the lobes protruding beyond the tissue), and purple markings at the base of the lobes, the stamens unequal and free; field photo of *Dean 9530* (DAV) **C** flower of L. chiapensis (Brandeg.) Standl. var. sparsistellata Standl. & Steyerm. with corolla rotate in orientation, shallowly-stellate in outline, abundant interpetalar tissue, and green markings at the base of the lobes, the stamens unequal and free; field photo of *Dean 9507* (DAV) **D** flower of *L. sideroxyloides* with corolla slightly campanulate to rotate in orientation, stellate in outline, and interpetalar tissue connecting just the lower portions of the lobes, the stamens equal and free; field photo of *Dean 9512* (DAV) **E** flower of *L. heteroclita* with corolla slightly campanulate to rotate in orientation, deeply-stellate in outline, and no interpetalar tissue, the stamens equal with partially connate anthers; field photo of *Dean 9500* (DAV) **F** flower of *L. ceratocalycia* (Donn.Sm.) Bitter with corolla rotate in orientation, deeply-stellate in outline, sparse interpetalar tissue at edges of lobes, and purple markings at the base of the lobes, the stamens equal and free; field photo of *Dean 9532* (DAV). Scale bars: 1 cm.

The corolla in *Lycianthes* is gamopetalous with a short tube that is inserted into the calyx. The limb is five-lobed, with the lobes usually connected to various degrees by thinner “interpetalar” tissue (Fig. 4A–D). In some species, the interpetalar tissue is lacking, and the limb is divided nearly to the tube (Fig. 4E, F). We use the term “stellate” for corollas that are divided shallowly to deeply and the term entire for corollas that are undivided. For consistency with past publications on *Lycianthes* (Dean 2014; Dean et al. 2007, 2017a, 2018c, 2019a, b), we are using the terms rotate, campanulate, and reflexed to indicate the orientation of the corolla limb in relation to the tube. We are using the term rotate as it is defined in Lawrence (1951, pg. 768) and Radford et al. (1974, pg. 104) to mean “… a gamopetalous corolla with a flat and circular limb at right angles to the short obsolete tube.” The illustrations in Lawrence (fig. 315, pg. 754) and Radford et al. (fig. 6.6 pg. 101) show a divided limb, indicating that the limb does not have to be entire to be rotate, and so we use the term rotate to mean a fully open wheel-shaped corolla, even if it is somewhat stellate. This usage of rotate differs from usage of the term in works on the genus *Solanum* to mean an unlobed, entire limb (see for example Spooner et al. 2004). We use the positional term campanulate for limbs that are oriented to the tube at greater than a right angle and the term reflexed for limbs that are oriented to the tube at less than a right angle.

The corollas of *Lycianthes* usually exhibit diurnal movements, opening and closing each day for several days in a row (Dean 2001). The corollas of many Mexican and Guatemalan species open at daybreak and close by late morning; herbarium specimens of species with this pattern often have closed corollas. Exceptions to this pattern are the morphologically similar and perhaps closely-related *L. glabripetala*, *L. inconspicua*, and *L. amatitlanensis*, as well as the horticultural species *L. rantonnetii* (Carr.) Bitter, which have flowers that stay open for most of the day and close at night; herbarium specimens of these species usually have open corollas. As explained elsewhere (Dean et al. 2017a), with only closed corollas available for study, it is often difficult to measure corolla diameter on specimens and most accurate to measure corolla length from the bottom of the calyx tube to the tip of the corolla lobe. It may also be difficult to know the typical corolla orientation (campanulate, rotate, reflexed), which can also affect a diameter measurement. Therefore, corolla size in this paper is usually just given as length. An exception to this is species that were studied extensively in the field and/or greenhouse (such as the herbs of series *Meizonodontae*); in those cases, floral diameter is also given.

The color of the corollas of *Lycianthes* included here range from white to purple on the adaxial side; the abaxial side may be the same color as the adaxial side, or the lobes may be green. As described elsewhere (Dean 2004; Dean et al. 2017a, 2019b), many *Lycianthes* have darker-colored markings on the adaxial side of the corolla. These markings range from purple stripes on the lobes, to a ring of purple color at the base, or green or yellow patches of color on the lobes (Fig. 4).

**Stamens**

Stamens in *Lycianthes* can be equal (Fig. 4D–F) or unequal (Fig. 4A–C). If unequal, differences in length are due to differences in filament length; anther length within a single flower is usually equal, or nearly so. In *Lycianthes* species with unequal stamens, the species native to Mexico and Guatemala have one long stamen and four shorter stamens (Fig. 4A–C); the one species included here that is non-native to the region (the cultivated *L. rantonnetii*) has three long stamens and two short stamens (illustrated in fig. 4 of Barboza and Hunziker 1992).

Anthers can be connate, connivent, or free. They can be glabrous or pubescent; most of the *Lycianthes* with multangulate- or geminate-stellate trichomes that are covered in this paper have sparsely pubescent anthers. Dehiscence is usually by pores at or near the anther tip, sometimes on the inner or outer face of the anther. An exception to this pattern is found in the Mexican endemic *L. anomala*. In *L. anomala*, the anthers are connate and dehisce at their edges, with the pores of one anther being coherent with those of the adjacent anther; these extremely large pores eventually become slits that extend half-way down the anther. This type of dehiscence is also found in the Central American species *L. synanthera*.

The pollen of most *Lycianthes* has not been investigated. As part of her monograph on the perennial herbs of series *Meizonodonatae*, the first author examined the pollen of all the species of the series (Dean 1995, 2004). She found that most species in the series have smooth, triangular, tricolporate pollen. However, two of the species (*L. acapulcensis* and *L. rzedowskii*) are dicolporate, one species (*L. ciliolata*) is dicolporate with a remnant third pore, and *L. starbuckii* has variable pollen ranging from tricolporate to dicolporate with a remnant third pore (Dean 2004). The first author completed an informal survey of the pollen of eight additional taxa included in this paper (*L. anomala*, *L. geminiflora*, *L. jalicensis*, *L. pilifera*, *L. pringlei*, L. scandens var. scandens, *L. stephanocalyx*, and *L. tricolor*). All seven taxa have pollen that is smooth and tricolporate, with the shape ranging from spherical to triangular.

**Gynoecium**

The gynoecium in the *Lycianthes* species included here is bicarpellate with a conic, ovoid, or globose ovary. The single linear style is straight to curved, and the stigma can be truncate, capitate, or oblong.

**Fruits**

**Figure 5.**
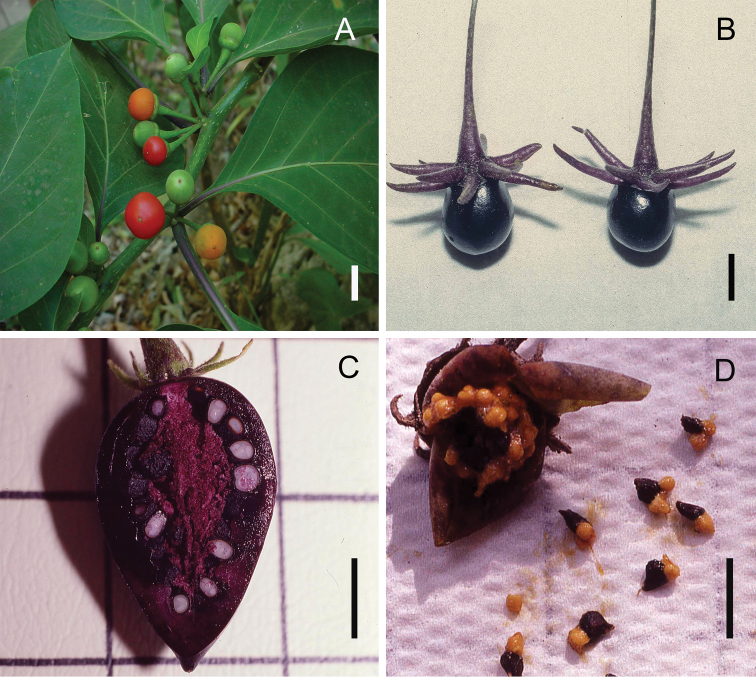
*Lycianthes* fruits **A** immature (green) and mature (orange to red) globose berries of *L. heteroclita* (note the calyx completely lacking appendages), field photo taken by the first author at El Chocoyero, Nicaragua **B** mature, ovoid, dark purple fruits of *L. pilifera*, photo of *Dean 242* collected at the San Francisco Botanical Garden **C** longitudinal section of mature, ovoid, dark purple fruits of *L. ciliolata* showing the light purple, powdery placental area, greenhouse photo of *Dean 295* (DAV) **D** dissected fruit of *L. peduncularis* showing the black seeds and yellow sclerotic granules, field photo of *Dean 303* (DAV). Scale bars: 1 cm.

The fruits of the *Lycianthes* treated here are berries ranging in shape from depressed-globose to ovoid (Fig. 5A, B). The exocarp can be yellow, orange, red, blue, or dark purple (nearly black) (Fig. 5A, B); immature fruits are usually green (Fig. 5A), but in a minority of species the color changes from green to white before changing to the mature color. In *L. barbatula* the mature fruit color is still unknown, but it has been listed as white on labels. In species with simple trichomes, the fruit exocarp is usually glabrous; in species with multangulate- or geminate-stellate trichomes, a few trichomes may be present on the exocarp. The mesocarp and placental areas of the fruits can be dry, powdery, or soft (fleshy or juicy). A minority are dry throughout (e.g. *L. textitlaniana*), while a few of the species in series *Meizonodontae* have a juicy mesocarp and a powdery placental area (*L. acapulcensis*, *L. ciliolata*, *L. rzedowskii*) (Fig. 5C). In two of the species treated here (*L. peduncularis* and *L. rantonnetii*), the mesocarp has numerous yellow sclerotic granules (also called sclereids or stone cells) (Fig. 5D) which sometimes attach to the seeds or are mistaken for seeds.

**Seeds**

Seed shape terminology used in this paper is taken from Radford et al. (1974); seed surface terminology is taken from Gunn and Gaffney (1974). The seeds of 15 of the species covered in this paper have been illustrated in previous publications (Barboza and Hunziker 1992; Dean 2004; Dean et al. 2007, 2017a, b, 2019b). Most *Lycianthes* species covered in this paper have compressed seeds, usually lenticular (completely flattened). A minority of species have seeds that are not compressed at all. This includes some of the species in series *Meizonodonatae* (*L. acapulcensis*, *L. ciliolata*, *L. peduncularis*, *L. rzedowskii*) and *L. rantonnetii*. Others are compressed but not lenticular (e.g. *L. dejecta*, *L. moziniana*, *L. pringlei*, *L. textitlaniana*).

The color of the seeds of the species included here can be tan, yellow, orange, brown, or black. The seed reticulum (surface) has a serpentine pattern formed by the periclinal walls of the surface cells; the luminae of the cells can be shallow to deep. Often, lenticular seeds have a smooth center with an indistinct serpentine pattern and cells with shallow luminae; however, the thickened margin of these seeds have a reticulum with much deeper luminae. Seeds that are more three-dimensional often have a reticulum with a more pronounced serpentine pattern and cells with much deeper luminae. As discussed in previous papers (Dean 2004; Dean et al. 2019b), some seeds have fibrils that protrude from the periclinal walls of the reticulum (e.g. *L. dejecta*, *L. pilifera*) (Lester and Durrands 1984).

**Geographic distribution, habitat and elevation**

Twenty-two taxa of *Lycianthes* are endemic to Mexico, three are endemic to Guatemala, and eight are found in Mexico and Guatemala and no other country (Table 1). The rest of the taxa included here have distributions that extend further into Central America, with a few extending to South America and one to the Caribbean (Table 1). Their elevational ranges include sea level (e.g. Lycianthes scandens var. scandens, *L. armentalis*) to nearly 4000 m in elevation (*L. quichensis*). Based on the vegetation classification of Rzedowski (1978), the habitats where these taxa are found include coniferous forest, deciduous forest (including oak forest), cloud forest, tropical dry forest, tropical moist forest, xerophilous scrub and agricultural areas; the most common habitat is oak forest. In common with many Solanaceae, many of the taxa included here are found in light gaps or edges, rather than dark forest.

**Conservation status**

The preliminary conservation assessment for each species is discussed under each species treatment. The conservation assessments range from Least Concern for common and widespread species to Critically Endangered for species that are only known from one location (Table 2).

**Table 2.** Preliminary conservation assessments for the native *Lycianthes* of Mexico and Guatemala calculated using GeoCAT.

Species	Assessment
*L. acapulcensis*	Least Concern
*L. amatitlanensis*	Least Concern
*L. anomala*	Endangered
*L. armentalis*	Least Concern
*L. arrazolensis*	Least Concern
*L. barbatula*	Endangered
*L. breedlovei*	Endangered
*L. caeciliae*	Endangered
*L. ceratocalycia*	Endangered
L. chiapensis var. chiapensis	Endangered
L. chiapensis var. sparsistellata	Least Concern
*L. ciliolata*	Least Concern
*L. connata*	Least Concern
*L. cuchumatanensis*	Critically Endangered
*L. dejecta*	Least Concern
*L. fredyclaudiae*	Endangered
*L. geminiflora*	Near Threatened
*L. glabripetala*	Vulnerable
*L. gongylodes*	Critically Endangered
*L. gorgonea*	Least Concern
*L. grandifolia*	Endangered
*L. heteroclita*	Least Concern
*L. hintonii*	Critically Endangered
*L. hypoleuca*	Least Concern
*L. inconspicua*	Endangered
*L. jalicensis*	Vulnerable
*L. limitanea*	Least Concern
*L. manantlanensis*	Least Concern
*L. mariovelizii*	Least Concern
*L. michaelneei*	Endangered
L. moziniana var. margaretiana	Near Threatened
L. moziniana var. moziniana	Least Concern
L. moziniana var. oaxacana	Vulnerable
*L. nitida*	Least Concern
*L. ocellata*	Endangered
*L. orogenes*	Endangered
*L. peduncularis*	Least Concern
*L. pilifera*	Endangered
*L. pringlei*	Endangered
*L. purpusii*	Least Concern
*L. quichensis*	Endangered
*L. rafatorresii*	Least Concern
*L. rzedowskii*	Endangered
L. scandens var. flavicans	Least Concern
L. scandens var. scandens	Least Concern
*L. sideroxyloides*	Least Concern
*L. starbuckii*	Endangered
*L. stephanocalyx*	Least Concern
*L. surotatensis*	Least Concern
*L. textitlaniana*	Critically Endangered
*L. tricolor*	Least Concern
*L. venturana*	Endangered

## Taxonomic treatment

### Lycianthes

Taxon classificationPlantae

(Dunal) Hassl., Annuaire Conserv. Jard. Bot. Genève 20: 180. 1917. Nom. conserv.

Otilix Raf., Medical Fl. 2: 87. 1830. Nom. rej. Type: Solanum lycioides L.
Solanum subsect. Lycianthes Dunal, Prodr. [A. P. de Candolle] 13(1): 29. 1852. Type: Solanum lycioides L. (designated by D’Arcy 1972, pg. 211)
Parascopolia Baill., Hist. Pl. 9: 338. 1888. Nom. rej. Type: P. acapulcensis Baill.
Solanum sect. Lycianthes (Dunal) Wettst., Nat. Pflanzenfam. 4(3b): 22. 1891. Type: Based on Solanum subsect. Lycianthes Dunal
Solanum subgenus Lycianthes (Dunal) Bitter, Bot. Jahrb. Syst. 54: 424. 1917. Type: Based on Solanum subsect. Lycianthes Dunal

#### Type.

Based on Solanum subsect. Lycianthes Dunal

#### Description.

Perennial herbs (from stolons, rhizomes, or tuberous roots), shrubs, or vines, sometimes epiphytic. Pubescence of glandular or eglandular, simple, dendritic, or stellate trichomes. Stems with sympodial growth. Leaves alternate, geminate, or solitary, simple, usually entire, usually petiolate, the base often unequal, the leaf pairs sometimes anysophyllous. Inflorescences axillary, the peduncles very short or absent, with one to many pedicelled flowers; calyx with truncate rim, often enlarging in fruit, 10-nerved, often with five to ten (25) appendages protruding from the calyx below the margin; corolla with five lobes, the lobes often connected by interpetalar tissue, the shape entire to stellate, opening and closing daily for several days in a row (sometimes opening only in the very early morning), campanulate, rotate or reflexed when open, white to purple or blue; stamens inserted near the base of the corolla, the filaments equal or not, the anthers free, connivent, or connate, dehiscing by pores (rarely lengthwise); pistil 2-carpellate, ovary spherical, ovoid, or conical, style straight or curved, stigma capitate to oblong, entire to lobed, ovules usually numerous; fruit a berry, round to ovoid, the exocarp purple, red, orange, yellow, or green, some species with sclerotic granules in the outer part of the mesocarp; seeds usually numerous, lenticular compressed to round or angular in outline, tan, yellow, orange, brown or black.

#### Discussion.

Genus name based on the type species *Solanum lycioides*, named, presumably, for its thorny branches that resemble the genus *Lycium* L., which was first described from Lycia, in what is now Turkey.

### Artificial key to Mexican and Guatemalan *Lycianthes*

Key to the Groups

**Table:** 

1	Herbaceous perennial from tuberous roots, dying back to the ground each season; inflorescence one flowered; corollas rotate to reflexed in orientation, mostly entire (not stellate) in outline; stamens unequal	**Group 1**
–	Herbaceous perennial from rhizomes, subshrubs, shrubs, epiphytes, or vines, generally persisting above ground from one season to the next; inflorescence of one to many flowers; corollas campanulate, rotate, or reflexed in orientation, entire to stellate in outline; stamens equal or unequal	**2**
2	Plant with obvious glandular trichomes (to 1 mm long or more), at least on the pedicels and/or calyx, sometimes also on the leaves and stem	**Group 2**
–	Plant lacking obvious glandular trichomes, sometimes with small obscure glandular trichomes inside the calyx or elsewhere	**3**
3	Simple, furcate and/or dendritic trichomes forming a majority of the indument on stems and leaves, or plant completely glabrous	**Group 3**
–	Multangulate- or geminate-stellate trichomes (with more than two rays at a node, the rays sometimes rebranched) forming a majority of the indument on stems and leaves (simple or dendritic trichomes may also be present)	**Group 4**

**Group 1**

Herbaceous perennial from tuberous roots, dying back to the ground each season; inflorescence one flowered; corollas rotate to reflexed in orientation, mostly entire (not stellate) in outline; stamens unequal.

**Table:** 

1	Indument of multangulate stellate trichomes mixed with dendritic and simple trichomes, the dendritic trichomes usually up to 0.5 mm long; species of arid habitats	***L. dejecta***
–	Indument of simple and/or dendritic trichomes (not stellate), if present, the dendritic trichomes often greater than 0.5 mm long; species of both arid and non-arid habitats	**2**
2	Plant prostrate (rarely decumbent or ascending); berry with yellow sclerotic granules in the mesocarp; style often strongly curved downward; ovary rounded to ovoid, less than 2 mm long; indument of simple, antrorsely appressed trichomes	***L. peduncularis***
–	Plants prostrate or not; berry without sclerotic granules; style straight to slightly curved, never strongly curved downward; ovary conical, usually longer than 2 mm (sometimes shorter in *L. starbuckii*); indument of simple or dendritic trichomes, these spreading or retrorsely appressed (rarely, antrorsely appressed in Chiapan or Guatemalan populations of *L. ciliolata*)	**3**
3	Corolla white, with or without maroon to purple nectar guides; corolla lobes usually glabrous abaxially; plant body usually erect, with first sympodial unit well developed above the ground; first two branching points on the plant body usually dichasial	**4**
–	Corolla lilac, violet or light purple, with maroon to purple nectar guides; corolla lobes glabrous or pubescent abaxially; plant body erect, decumbent or prostrate with first sympodial unit sometimes not well developed above ground; first branching point on the plant body dichasial, second sympodial branching point sometimes monochasial	**6**
4	Filament of the longest stamen usually more than twice as long as those of the lateral stamens; pores of the anther of the longest stamen lateral, dehiscing toward the style, usually narrow and linear; stigmas usually deeply bilobed (rarely just capitate); widespread species of the transvolcanic belt, southern Mexico and Central America	***L. acapulcensis***
–	Filament of the longest stamen usually less than twice as long as those of the lateral stamens; pores of the anther of the longest stamen nearly terminal, oval; stigmas not deeply bilobed; species of various regions of Mexico and Guatemala	**5**
5	First sympodial unit usually very well developed (to 90 cm long) with numerous internodes (usually 10–21) and leaves; lateral branching from the nodes of the first sympodial unit usually not present at the time of flowering; subsequent sympodial growth poorly developed; pollen dicolporate; states of Morelos, Michoacán and México, on volcanic soils	***L. rzedowskii***
–	First sympodial unit *c.* 25 cm long, the internodes *c.* 13; lateral branching from the nodes of the first sympodial unit usually present at the time of flowering; subsequent sympodial growth about equal to the length of the first sympodial unit; pollen tricolporate; state of Nuevo León, on limestone soils	***L. hintonii***
6	Berry green to tan at maturity, sometimes with purple blotches; seeds less than 3 mm long, smooth and shiny to the naked eye; second sympodial branching point usually monochasial	**7**
–	Berry purple to black-purple at maturity; seeds greater than 3 mm long, rough-textured and dull to the naked eye; second sympodial branching point usually dichasial	**9**
7	Calyx teeth in flower lax, laying against the corolla; calyx teeth in fruit appressed to the berry, not spreading; abaxial side of corolla lobes densely hairy; leaves of first sympodial unit cuneate at base, not attenuate; leaves sessile or petiole less than 5 mm long; agricultural areas of the transvolcanic belt	**L. moziniana var. moziniana**
–	Calyx teeth in flower slightly spreading; calyx teeth in fruit spreading, not appressed to the berry; abaxial side of the corolla lobes slightly hairy to nearly glabrous (glabrous in Nuevo Leon); leaves of first sympodial unit attenuate at base; petiole to 1.5 cm long; not of the region of the transvolcanic belt (states of San Luis Potosi, Nuevo Leon, Oaxaca) often growing on limestone soil	**8**
8	Filaments glabrous; exocarp of berry green; placental area green and juicy; disturbed clearings and agricultural fields of mountains of state of Oaxaca	**L. moziniana var. oaxacana**
–	Filaments glabrous or pubescent; exocarp of berry green or tan, often with purple blotches; placental area often purplish and powdery; forested areas or more disturbed situations on limestone in northern Mexico (states of San Luis Potosí and Nuevo León)	**L. moziniana var. margaretiana**
9	Calyx teeth at anthesis lax or slightly spreading, not widely spreading or reflexed; corolla lobes usually noticeably pubescent abaxially; first sympodial unit poorly developed above ground, the plant body often prostrate; rare species of the Sierra de Nanchititla (state of México)	***L. starbuckii***
–	Calyx teeth at anthesis widely spreading to reflexed; corolla lobes generally glabrous abaxially (rarely slightly pubescent in Oaxacan plants); first sympodial unit well-developed above ground, the plant body usually erect; widespread in SE Mexico and Guatemala	***L. ciliolata***

**Group 2**

Plants with obvious glandular trichomes (to 1 mm long or more), at least on the pedicels and/or calyx, sometimes also on the leaves and stem

**Table:** 

1	Calyx appendages less than 2 mm long, or reduced to small protuberances, the calyx sometimes tearing, appearing lobed or two-lipped; berry ovoid, orange	***L. pringlei***
–	Calyx appendages usually greater than 2 mm long, the calyx margin truncate, never appearing lobed or two-lipped; berry turbinate, globose, or depressed globose, orange to red	**2**
2	Corolla purple; berry turbinate, the tip pointed, orange; seed surface with widely spaced serpentine cell pattern, noticeably pitted; state of Oaxaca	***L. textitlaniana***
–	Corolla white to lilac, sometimes with purple or green markings; berry globose to depressed globose, the tip round, orange to red; seed surface finely marked with shallow serpentine cell pattern, not noticeably pitted; widely distributed	**3**
3	Leaf pairs of similar shape; inflorescence of 1–5 flowers; calyx appendages in flower 2–10 mm long, to 11 mm long in fruit; stamens unequal with one filament noticeably longer than the other four, the anthers free of one another; from state of Sinaloa to Oaxaca, 670–2200 m in elevation	***L. surotatensis***
–	Leaf pairs usually very different in shape, the smaller leaf blade orbicular to ovate, the larger leaf blade narrowly ovate to lanceolate; inflorescence of a single flower; calyx appendages in flower 7–15 mm long, to 20 mm long in fruit; stamens equal or nearly so, the filaments of approximately the same length, the anthers connate to one another at their edges; Mexico (states of Chiapas, Oaxaca, Tabasco, and Veracruz) to Belize and Guatemala, 200–1000 m in elevation	***L. gorgonea***

**Group 3**

Plants with simple, furcate and/or dendritic trichomes forming a majority of the indument on stems and leaves, or plant completely glabrous

**Table:** 

1	Shrub; stems angular or with prominent striations; corolla dark purple; berries often not developing, the exocarp yellow to light orange when mature, the mesocarp with many sclerotic granules; cultivated plants	***L. rantonnetii***
–	Shrub or other life form; stems angular and striated or not; corolla white to light purple, not usually dark purple; berries often developing, the exocarp yellow, orange, red, or dark purple when mature, the mesocarp lacking sclerotic granules; non-cultivated plants	**2**
2	Leaf blades glabrous except for trichomes restricted to the axils where the primary veins meet the midvein on the abaxial side; stamens usually equal in length	**3**
–	Leaf blades glabrous to pubescent, but trichomes not restricted to the axils where the primary veins meet the midvein on the abaxial side; stamens equal or unequal in length	**4**
3	Shrub or vine, often epiphytic; calyx coriaceous and fleshy, the appendages 0.25–1 mm long, connate at their bases, reflexed as a unit; corolla stellate in outline, blue to purple adaxially; anthers connate at their edges	***L. anomala***
–	Shrub, terrestrial; calyx membranaceous, not fleshy, the appendages 0.5 to 2 mm long, free at their bases and not reflexed; corolla entire to very slightly stellate in outline, white with lavender ring at the base adaxially; anthers free at their edges	***L. barbatula***
4	Stamens equal in length or nearly so	**5**
–	Stamens unequal in length, one filament noticeably longer than the other four	**19**
5	Calyx lacking appendages; corolla shallowly to deeply stellate in outline, sometimes without interpetalar tissue	**6**
–	Calyx with appendages 0.25–20 mm long; corolla entire to stellate in outline, with interpetalar tissue present at least at the base of the corolla lobes	**9**
6	Calyx margin often irregularly notched or torn in flower and/or fruit, sometimes appearing lobed; corolla stellate, divided 1/2 to nearly all the way to the base, with abundant interpetalar tissue; anthers often with dark connective	***L. manantlanensis***
–	Calyx margin entire, truncate, in flower and fruit; corolla stellate, deeply divided to the base, lacking interpetalar tissue; anthers without dark connective	**7**
7	Upper stem nodes remaining round in cross-section upon drying, quickly becoming woody; leaves completely glabrous, coriaceous, the geminate leaf pairs very different in size and shape, the smaller leaf usually 1/4 or less the length of the larger and round to ovate, the larger leaf narrowly ovate to lanceolate; anthers 4 mm long or more and connivent to one another at their edges; woody shrub to vine, usually epiphytic	***L. nitida***
–	Upper stem nodes compressed upon drying, lower stems sometimes woody; leaves glabrous to short pubescent, membranaceous, the geminate leaf pairs unequal in size but similar in shape; anthers less than 4 mm long and free of one another; large herb to shrub, sometimes epiphytic	**8**
8	Calyx up to 1.5 mm long; corolla 0.6–1.2 cm long; anthers 3–3.5 mm long; endemic to Mexico (states of Hidalgo, Oaxaca, Puebla, and Veracruz), usually over 800 m in elevation	***L. geminiflora***
–	Calyx 2 mm long or more; corolla 1–1.6 cm long; anthers 4–7 mm long; widespread in Mexico and Central America, usually up to 1000 m in elevation	***L. heteroclita***
9	Upper stem epidermis with rough texture formed by scurfy horizontal lines; corolla deeply stellate in outline, divided 3/4 of the way to the base, lilac to purple adaxially, the interpetalar tissue only present below the middle of the lobes; Mexico (state of Chiapas) and Guatemala, in cloud forest, usually above 1300 m in elevation	***L. ceratocalycia***
–	Upper stem epidermis smooth, angular or roughened by vertical lenticels, but without scurfy horizontal lines; corolla deeply stellate to rotate in outline, white, pale yellow, or purple adaxially, the sometimes abundant interpetalar tissue not restricted to the lower part of the lobes; widely distributed in Mexico and Central America in various habitats above or below 1300 m in elevation	**10**
10	Number of veins on either side of the leaf blade midvein of largest leaves usually 8 or more, the blades moderately to densely pubescent, the blade base very oblique; calyx teeth in flower up to 4 mm long, very narrow (less than 0.25 mm wide), often withering in fruit; corollas white to pale yellow, stellate in outline, divided 1/2 to nearly all the way to the base; anthers free of one another, up to 3 mm long, abruptly attenuate at the tip; berry globose, orange-red at maturity	**11**
–	Number of veins on either side of the leaf blade midvein of largest leaves usually less than 8 (if 8, the blades glabrous), the blades glabrous to pubescent, the blade base not oblique to somewhat oblique; calyx teeth in flower up to 15 mm long, not very narrow (greater than 0.25 mm wide), not withering in fruit; corollas white to purple, stellate to rotate in outline; anthers free of one another, connivent, or connate, usually greater than 3 mm long, not truncate, rounded, or acute at the tip, not abruptly attenuate; berry globose to ovoid, orange-red or dark purple at maturity	**13**
11	Number of veins on either side of the larger leaf blades usually 10–22, the blade trichomes usually spreading along the midvein on the abaxial side; corolla 0.5–0.8 cm long, the lobes moderately pubescent abaxially, with a tuft of trichomes at the tip; Mexico (southern Veracruz) to Central America, often below 1000 m in elevation	***L. amatitlanensis***
–	Number of veins on either side of the larger leaf blades usually 8–12, the blade trichomes appressed along the midvein on the abaxial side; corolla 0.8–1.2 cm long, the lobes glabrous to sparsely pubescent abaxially, lacking tuft of trichomes at the tip; Mexico (states of Querétaro and Veracruz), Guatemala, and Central America usually above 1000 m in elevation	**12**
12	Trichomes along midvein on abaxial leaf blade surface bent to wavy, appearing woolly; pedicels in flower 9–15 mm long, in fruit 12–20 mm long; corolla 1–1.2 cm long, nearly glabrous abaxially except for sparse hairs near the lobe tip; endemic to Mexico (northern state of Veracruz to Querétaro)	***L. glabripetala***
–	Trichomes along midvein on abaxial leaf blade surface mostly straight and appressed, not appearing woolly; pedicels in flower (11) 15–30 mm long, in fruit 30–36 mm long; corolla 0.5–1 cm long, sparsely pubescent abaxially, densest near the lobe tip; Guatemala to Panama	***L. inconspicua***
13	Perennial herb to woody vine or shrub; mature berry red; anthers connate to connivent; usually occurring at or below 1000 m in elevation	**14**
–	Herb, shrub or treelet; mature berry orange, red or dark purple; anthers free from one another; usually occurring above 1000 m in elevation	**15**
14	Rhizomatous herb to climbing shrub; plant glabrous to sparsely pubescent, the appressed to ascending trichomes to 0.6 mm long, eglandular; calyx appendages 1.5–5 mm long in flower, to 8 mm long in fruit	***L. stephanocalyx***
–	Weak shrub to vine; plant moderately to densely pubescent, the spreading trichomes to 3 mm long, sometimes glandular; calyx appendages 7–15 mm long in flower, to 20 mm long in fruit	***L. gorgonea***
15	Leaf blades glabrous and shiny on both sides, rarely with a few appressed-ascending trichomes to 0.25 mm long; corolla stellate in outline, adaxially white with yellow-green or purple markings near the base; anthers yellow, the connective usually dark in color	***L. manantlanensis***
–	Leaf blades glabrous to moderately pubescent with appressed to spreading trichomes to 1.25 mm long; corolla entire to stellate in outline, adaxially white to pale purple, with or without markings near the base; anthers yellow to purple, the connective usually light in color	**16**
16	Leaf blades sparsely to densely pubescent, the trichomes collapsing when dry; berry orange to red at maturity; Mexico (state of Chiapas) and Guatemala	**17**
–	Leaf blades glabrous to sparsely pubescent, the trichomes remaining conical and acute at the tip when dry; berry dark purple at maturity; Mexico (states of Veracruz and Oaxaca)	**18**
17	Trichomes usually simple (sometimes dendritic), often curling and crisped; calyx appendages to 0.5 mm long; corolla white adaxially, shallowly to deeply stellate in outline, up to 1 cm long; berry globose; Guatemala	***L. gongylodes***
–	Trichomes simple, not curling or crisped; calyx appendages 3–6 mm long; corolla light purple with darker markings at base adaxially, entire to shallowly stellate in outline, up to 3 cm long; berry usually ovoid; Mexico (state of Chiapas) and Guatemala	***L. quichensis***
18	Corolla pale to dark purple with green markings at base adaxially, stellate in outline, divided 1/3 to 2/3 of the way to the base; Mexico (state of Veracruz)	***L. caeciliae***
–	Corolla white to light purple with dark purple ring and green markings at base adaxially, nearly entire in outline; Mexico (state of Oaxaca)	***L. pilifera***
19	Calyx usually nearly glabrous, the calyx appendages less than 2 mm long in flower, less than 3 mm long in fruit; plants glabrous to sparsely pubescent, the trichomes less than 1 mm long; mature berry dark purple (if fruit not present, also try 19b); greater than 1000 m in elevation	**20**
–	Calyx glabrous or pubescent, the calyx appendages often2 mm long or more in flower, 3 mm long or more in fruit; plants glabrous to densely pubescent, the trichomes often at least 1 mm long; mature berry orange to red; greater than or less than 1000 m in elevation	**21**
20	Plant usually glabrous, rarely with occasional tan to brown appressed trichomes to 0.25 mm long; calyx margin often torn, appearing lobed, the appendages in flower up to 1 mm long or absent; corolla stellate in outline, divided 1/2 to all of the way to the base; stamens equal to slightly unequal, the longest filament to 2 mm long, less than twice the length of the short filaments, the anthers 2–3 mm long, often with dark-colored connective; seeds unnotched; Mexico (widespread in the Sierra Madre del Sur from state of Jalisco to Chiapas) south to El Salvador	***L. manantlanensis***
–	Plant glabrous to sparsely pubescent, the trichomes to 0.75 mm long; calyx margin entire, not appearing lobed, the appendages in flower 1–1.5 mm long; corolla entire to shallowly stellate in outline, divided ca. 1/5 of the way to the base; stamens very unequal, the longest filament to 3 mm long, usually twice the length of the short filaments, the anthers 3–4 mm long, usually with light-colored connective; seeds with deep notch on one side; Mexico (state of Chiapas) and Guatemala	***L. orogenes***
21	Shrub to large vine to 10 m tall; trichomes tan, yellow, or red-brown, simple to furcate, 1–4 mm long; calyx often densely pubescent, the appendages in flower 7–17 mm long; mature berry 15–30 mm in diameter	***L. purpusii***
–	Shrub, sometimes scandent, to 5 (7) m tall; trichomes white, off-white, tan, light yellow, brown or light purple, but never red-brown, simple, to 2.5 mm long; calyx glabrous to densely pubescent, the appendages in flower 0.5–9 mm long; mature berry less than 15 mm in diameter	**22**
22	Flowering calyx with well-developed rim 1–3 mm long, the appendages 0.4–4 mm long, connate at their bases, forming a continuous shelf of tissue (this feature especially visible in fruit); Mexico (states of Chiapas and Oaxaca) to Guatemala, above 1500 m	***L. connata***
–	Flowering calyx with rim 0.5 to 1 mm long, the appendages 0.25–11 mm long, free at their bases, not forming a continuous shelf of tissue; Mexico and Central America, 350–3000 m	**23**
23	Calyx glabrous or nearly so; abaxial surface of corolla glabrous or sparsely puberulent with trichomes to 0.1 mm long (difficult to see without magnification); stem trichomes usually appressed-antrorse; Mexico (states of Jalisco, Puebla, and Veracruz)	**24**
–	Calyx and abaxial surface of the corolla lobes usually puberulent with dense trichomes to 2 mm (easily seen without magnification), best seen in bud (sometimes nearly glabrous in *L. arrazolensis* in the state of Oaxaca); stem trichomes spreading to appressed; many different regions of Mexico and Guatemala, south to Nicaragua	**25**
24	Apex of berry sometimes apiculate due to persisting remnant of style base; calyx 1.5–2.5 mm long; Mexico (from northern state of Puebla and adjacent state of Veracruz)	***L. venturana***
–	Apex of berry rounded; calyx 2–4 mm long; Mexico (state of Jalisco)	***L. jalicensis***
25	Corolla lobes and interpetalar membrane purple; stem trichomes usually densely matted; some leaf trichomes to 2 mm long; Mexico (state of Veracruz)	***L. michaelneei***
–	Corolla lobes and interpetalar membrane white to lilac, often with darker purple or maroon stripes on the lobes; stem trichomes often dense but not matted, the individual trichomes spreading and separated from neighboring trichomes; leaf trichomes usually less than 1.5 mm long; southern Mexico (excluding the state of Veracruz), Guatemala, and south to Nicaragua	**26**
26	New growth angled or ribbed, somewhat compressed upon drying; largest leaves usually with blade greater than 16 cm long; longest appendages on calyx 4–7 mm long; 1700–2000 m; Mexico (southeastern state of Chiapas)	***L. grandifolia***
–	New growth terete, not angled or ribbed, not much compressed upon drying; largest leaves usually with blade less than 16 cm long (if largest leaf blade greater than 16 cm, the calyx appendages usually < 4 mm long); 500–3000 m; southern Mexico to Nicaragua	**27**
27	Calyx appendages 5–9 mm long, with at least some appendages on single calyx 7–9 mm long; base of appendages somewhat to very flattened; 700–1000 m	***L. mariovelizii***
–	Calyx appendages usually < 5 mm long (rarely to 5 mm in *L. arrazolensis* in state of Guerrero); base of appendages not flattened; 500–3000 m	**28**
28	Pedicels of the oldest (third day) flowers mostly greater than 1.2 cm long; pedicels of fully developed fruits often greater than 2 cm long; calyx rim above the appendage insertion usually less than 0.5 mm long and covered by the slightly spreading appendages; mature seeds with notch; usually above 2000 m; southern Mexico, Guatemala, El Salvador	***L. tricolor***
–	Pedicels of the oldest flowers mostly less than 1.2 cm long; pedicels of fully developed fruits often less than 2 cm long; calyx rim above the appendage insertion usually greater than 0.5 mm long and exposed by the widely spreading appendages; mature seeds usually lacking notch; 500–3000 m; southern Mexico, Guatemala, to Nicaragua	***L. arrazolensis***

**Group 4**

Multangulate- or geminate-stellate trichomes (with more than two rays at a node, the rays sometimes rebranched) forming the majority of indument on stems and leaves

**Table:** 

1	Stamens equal or nearly so	**2**
–	Stamens obviously unequal, one filament much longer than the other four	**7**
2	Calyx lacking appendages, the rim often undulate, lobed or torn	**3**
–	Calyx with appendages greater than or equal to 0.25 mm long, the rim rarely undulate, lobed or torn	**4**
3	Larger leaf blades 8–15.5 × 4.5–10 cm, the abaxial side densely pubescent with, but not always obscured by, short to long-stalked, red-brown, multangulate- and geminate-stellate trichomes 0.5–1 mm in diameter; calyx 6–7 mm long in flower, 9–17 mm in diameter in fruit; anthers ca. 6 mm long	***L. limitanea***
–	Larger leaf blades 3–13 × 2–5 cm, the abaxial side obscured by a dense tomentum of overlapping, short-stalked, white to tan, stellate or multangulate-stellate trichomes less than 0.25 mm in diameter; calyx 2.5–4.5 mm long in flower, 6–8 mm in diameter in fruit; anthers 3–4 mm long	***L. hypoleuca***
4	Indument of multangulate-stellate trichomes with 3–5 rays at a node, the rays often rebranched, the individual trichomes with a branching tree-like appearance (easily seen on both sides of the leaf); calyx appendages linear, usually narrowed at the tip; corolla stellate in outline, usually divided to 1/2 (rarely 2/3) of the way to the base, with abundant interpetalar tissue connecting the lobes; stamens usually somewhat unequal with one stamen slightly longer than the other four	***L. breedlovei***
–	Indument of geminate-stellate trichomes with 5–8 rays at a node mixed with multangulate-stellate trichomes, the rays sometimes rebranched, the individual trichomes with a bottlebrush appearance (most easily seen on the adaxial leaf surface); calyx appendages usually obovate, rounded at the tip; corolla stellate in outline, divided 1/2 to 2/3 of the way to the base, with scant interpetalar tissue present only at base of corolla lobes; stamens equal	**5**
5	Calyx appendages with large, oblong, glandular area at tip, this area turning black upon drying	***L. ocellata***
–	Calyx appendages lacking glandular area at tip, remaining green upon drying	**6**
6	Upper stem branching divaricate (strongly zigzagging) well below the branch tips; leaf blades 2–10 × 0.5–3.5 cm, narrowly ovate to elliptic, acuminate at the tip, coriaceous, the trichomes often obscuring the abaxial surface; endemic to Guatemala	***L. cuchumatanensis***
–	Upper stem branching only divaricate at the very tips of the branches, not strongly zigzagging below the tip; leave blades 2.5–15 × 1.5–8 cm, broadly ovate to elliptic, acute to acuminate at the tip, thick chartaceous, the trichomes rarely obscuring the abaxial surface; Mexico to Nicaragua	***L. sideroxyloides***
7	Trichomes on leaves and stems often a mixture of colors (off-white to red-brown) and forms (simple, long-stalked furcate, and stalked multangulate-stellate) on the same plant, 1–4 mm long; flowering calyx appendages 7–17 mm long; mature berry 15–30 mm in diameter	***L. purpusii***
–	Trichomes on leaves and stems of various colors (white, off-white, yellow, orange, brown, or red-brown), but markedly different colors not usually present on the same plant and multangulate-stellate trichomes always present, these sessile to stalked, sometimes mixed with furcate trichomes, 0.05–1.5 (2) mm long; flowering calyx appendages 0.25–6 (8) mm long; mature berry 4–20 mm in diameter	**8**
8	Indument of stalked multangulate-stellate trichomes, the rays straight and usually rebranched, sometimes repeatedly; states of Chiapas, Mexico and Baja Verapaz, Guatemala	**9**
–	Indument of sessile to stalked multangulate-stellate and/or geminate-stellate trichomes, the rays rarely rebranched; widely distributed in Mexico and Guatemala	**10**
9	Corolla shallowly stellate in outline, usually divided to 1/2 (rarely 2/3) of the way to the base, white to pale lilac with darker purple color on the lobes adaxially; Mexico (state of Chiapas)	***L. breedlovei***
–	Corolla entire in outline, pale white to lilac, without darker purple color on the lobes adaxially; Guatemala (state of Baja Verapaz)	***L. fredyclaudiae***
10	Trichomes on leaves, stems, and calyx white to tan, not yellow, orange or brown	**11**
–	At least some trichomes on leaves, stems, and calyx yellow, orange, or brown, sometimes mixed with tan trichomes	**13**
11	Multangulate-stellate trichomes of adaxial side of leaf blade sessile to very short-stalked, the rays laying on the leaf surface; corolla white, shallowly stellate in outline	***L. rafatorresii***
–	Multangulate-stellate trichomes of adaxial side of leaf blade sessile to stalked, the rays not laying on the leaf surface; corolla white to purple, entire to shallowly stellate in outline	**12**
12	Vine to scandent shrub; trichomes furcate to multangulate-stellate, those on calyx minute (less than 0.25 mm in diameter) and difficult to see without magnification; leaf blade apex rounded to acute, the leaf veins usually obscure and light green in color; corolla light purple; coastal areas, up to 1000 m in elevation, widespread in Mexico and Central America, especially on the Caribbean slope	**L. scandens var. scandens**
–	Shrub, rarely vine; trichomes multangulate- to geminate-stellate, those on the calyx not minute (usually more than 0.25 mm in diameter), the surface often obscured by indument; leaf blade apex acute to short-acuminate, the leaf veins often prominent and white in color; corolla white to pale lavender; coastal areas and adjacent mountains, up to 1300 m in elevation, Pacific slope of Mexico south to Central America	**L. scandens var. flavicans**
13	Multangulate-stellate trichomes of adaxial side of leaf blade sessile to very short-stalked, the rays laying on the leaf surface; corolla white, shallowly stellate in outline	***L. rafatorresii***
–	Multangulate-stellate trichomes of adaxial side of leaf blade sessile to stalked, the rays not laying on the leaf surface; corolla white, entire to shallowly stellate in outline	**14**
14	Upper dichasial branching widely divaricate (the branches often spreading at a 180 degree angle), not forming a continuous, sinuous axis; epidermis light brown, usually smooth upon drying; multangulate-stellate trichomes usually with 5–8 (10) rays per whorl, the area where they join often becoming enlarged and spherical; corolla white, shallowly stellate in outline; mainly the Yucatán Peninsula of Mexico and adjacent Guatemala and Belize, usually 0–500 m in elevation	***L. armentalis***
–	Upper dichasial branches usually forming a continuous, sinuous axis; epidermis dark brown, often longitudinally wrinkled upon drying; multangulate-stellate trichomes usually with 3–6 rays per whorl, the area where they join just slightly enlarged and not particularly spherical; corolla white, entire to shallowly stellate in outline; away from immediate coast of Mexico and Central America, (500) 900–2500 m in elevation	**15**
15	Calyx 4–5 mm long in flower, 6–8 mm long in fruit	**L. chiapensis var. chiapensis**
–	Calyx 2–3.5 mm long in flower, 2–4 mm long in fruit	**L. chiapensis var. sparsistellata**

### Species descriptions

#### 1 Lycianthes acapulcensis

Taxon classificationPlantae

(Baill.) D’Arcy, Solanaceae Newsl. 2(4): 23. 1986

Fig. 6

Parascopolia acapulcensis Baill., Hist. Pl. (Baillon) 9: 339. 1888. Type: Mexico. Guerrero: Acapulco, Punto Griffon, 1888, *C. Thiébaut 1002* (lectotype, designated here: P [P00070403]).
Lycianthes grandifrons Bitter, Abh. Naturwiss. Verein Bremen 24 [preprint]: 418. 1919. Type: Costa Rica. San José: Llanos de Turrucares, 600 m, 18 Sep 1888, *H. Pittier & T. Durand 478* (holotype: BR [000000552872]; isotypes: CR [mixed collection with Witheringia solanacea L’Hér.], US [00027878]).
Lycianthes guatemalensis Bitter, Abh. Naturwiss. Verein Bremen 24 [preprint]: 419. 1919. Type: Guatemala. Retalhuleu: Retalulëu [Retalhuleu], May 1877, *K. Bernoulli & O. Cario 2384* (lectotype, designated by Dean 2004, pg. 393: GOET [GOET003442]).
Lycianthes somniculenta (Kunze ex Schltdl.) Bitter var. cladotricha Bitter, Abh. Naturwiss. Verein Bremen 24 [preprint]: 413. 1919. Type: Mexico. Morelos: Cuernavaca, in moist copses, 5000 ft, Jun–Jul 1896, *C. Pringle 6399* (lectotype designated by Dean 2004 pg. 395: MEXU [00029023]; isolectotypes: B [not seen, cited by Bitter 1919, probably destroyed], BM [000514912], BR [000000552840], E [E00570140], G [G00343072, G00343073], GH [00021855], GOET [GOET003441, GOET003440], HBG [HBG-511362], JE [JE00004691], K [K000063119], M [M-0166091], MEXU [00029022, 00029023], MO [MO-153222], NDG [NDG45130], NY [00138707], PH [00016314], S [S-G-9982], UC [104211], W [acc. # 1897-4064], WRSL [cited by Bitter 1919, not seen], WU [acc. # 037953], Z [Z-000028495]).
Lycianthes somniculenta (Kunze ex Schltdl.) Bitter var. ramosipila Bitter, Abh. Naturwiss. Verein Bremen 24 [preprint]: 413. 1919. Type: Cultivated in Paris (?) “plaines de terre froide”, *JDP (Jardin des Plantes) 82* (holotype: P [P00070402]).
Lycianthes villosula Bitter, Abh. Naturwiss. Verein Bremen 24 [preprint]: 420. 1919. Type: Costa Rica. Alajuela: El Brazil, gorges of Virilla River, 800 m, 14 Jul 1911, *H. Pittier 3676* (holotype: US [00027904]).

##### Type.

Based on *Parascopolia acapulcensis* Baill.

**Figure 6.**
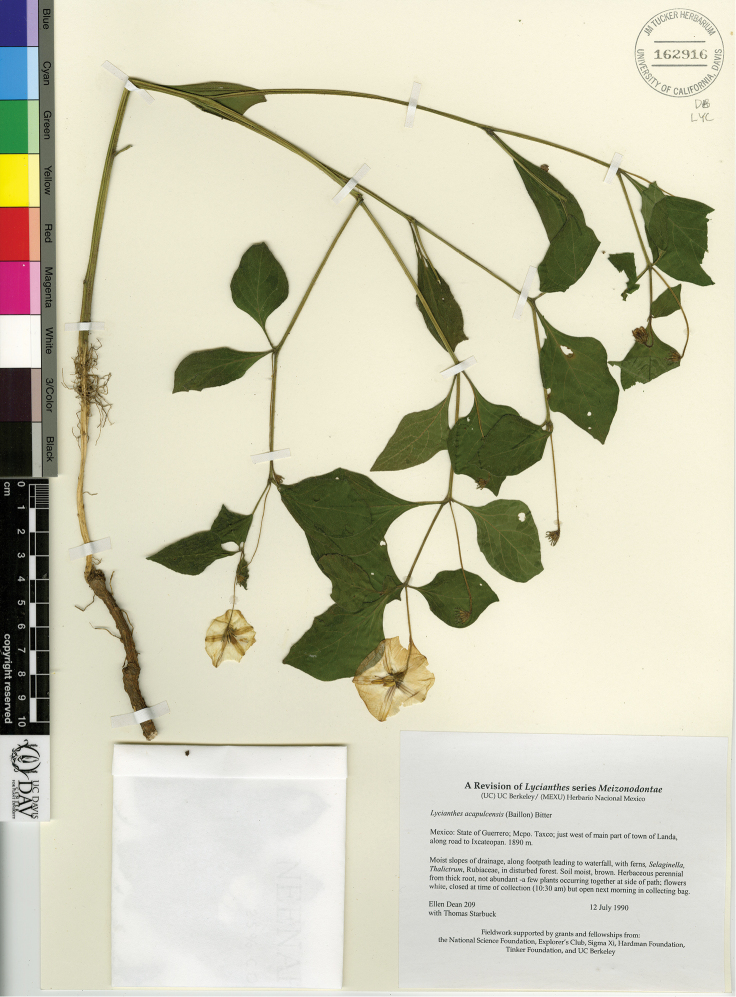
Image of herbarium specimen of *L. acapulcensis*, *Dean 209* (DAV). Image used with permission from the UC Davis Center for Plant Diversity.

##### Description.

Perennial herb from moniliform storage roots, decumbent to erect, 0.1–0.5 (1) m tall, dying back each season. Indument of white, uniseriate, multicellular, simple or dendritically branched, eglandular, spreading to appressed-retrorse trichomes 0.1–1 (2) mm long. Stems green to green-purple, sparsely to moderately pubescent, much compressed upon drying in a plant press, woody with age, especially near the base; first stem (1.5) 5–30 (70) cm long to first inflorescence, the internodes 2–10 (14); first two sympodial branching points dichasial, followed by monochasial branching, this sometimes very extensive (in some Costa Rican and Nicaraguan populations the stems spreading along the ground and rooting at the nodes). Leaves simple, the leaves of the upper sympodia usually paired and unequal in size, the larger ones with blades 3–18 × 1–8 cm, the smaller ones with blades 1/4 to 3/4 the size of the larger, the leaf pairs similar in shape, the blades ovate, elliptic, or obovate, chartaceous to thick chartaceous, glabrous to moderately pubescent, the primary veins 4–7 on each side of midvein, the base cuneate (rarely truncate), short attenuate or decurrent onto the petiole, slightly oblique on smaller leaves, the margin entire, usually irregularly undulate, the apex acute to short-acuminate (rarely long-acuminate), the petioles 0.5–1.5 (2.5) cm long, sometimes absent. Flowers solitary, axillary, oriented horizontally; peduncles absent; pedicels (10) 30–60 (85) mm and erect in flower, 20–70 (90) mm long and deflexed in fruit, sparsely to moderately pubescent; calyx (2) 2.5–5.5 (6.25) mm long, 3.5–5 (6) mm in diameter, obconic, campanulate, or urceolate, glabrous to moderately pubescent, the margin truncate, with (5) 10 linear, spreading to reflexed appendages 1–6.5 (9) mm long emerging 0.5–1 mm below the calyx rim; fruiting calyx enlarged, (1.5) 2–4 (6) mm long, 5–12.5 (14) mm in diameter, the appendages to 10 mm long, usually reflexed (sometimes appressed to fruit), often broken; corolla 1.1–2.7 cm long (2–5 cm in diameter), rotate in orientation, mostly entire in outline (with shallow notches), with abundant interpetalar tissue, white, sometimes with darker maroon to purple stripes along the major veins adaxially, green near the major veins abaxially, glabrous; stamens unequal, straight, the filaments of three lengths, the two shortest filaments 1–2.5 (3.5) mm long, the two medium filaments 1–3.5 (4.5) mm long, the one long filament 4–9 mm long, the length of the long filament nearly always 2–4 times that of medium filaments, glabrous, the anthers 4.5–7.5 mm long, lanceolate to oblong (rarely ovate), free of one another, yellow, glabrous, poricidal at the tips, the pores linear to ovate, dehiscing distally or away from the style, not opening into longitudinal slits; pollen grains dicolporate; pistil with glabrous ovary, the style 9–14 mm, linear, straight to slightly curved, glabrous, the stigma usually strongly bilobed (sometimes weakly bilobed or capitate). Fruit a berry, remaining attached to calyx at maturity, pendent, (10) 15–50 (70) mm long, (4.5) 9–20 mm in diameter, short-ovoid to elongate fusiform, the tip apiculate to long-attenuate, the exocarp glossy blue-black, grey-blue, bright blue, or dull purple, glabrous, the mesocarp ranging from dark purple and juicy to light purple and powdery, lacking sclerotic granules, the placental area light purple and powdery. Seeds (11) 20–80 (90) per fruit, 2.5–3.5 × 3–4.2 mm, not compressed, irregularly depressed obovate to depressed rhombic, ridged and blistered along one side, black, the surface reticulum with a rough, loose serpentine pattern with deep luminae.

##### Chromosome number.

2n = 24 from *Dean 313, 314, 329* (Dean 2004).

##### Distribution and habitat.

**Figure 7.**
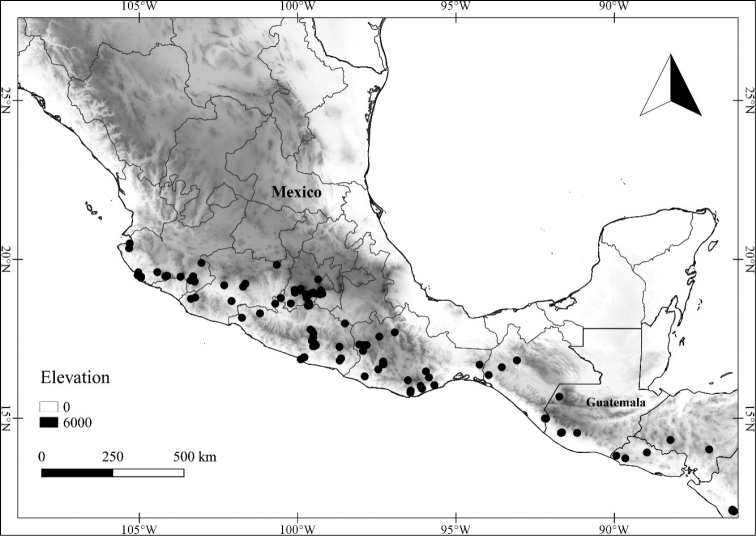
Map of geographic distribution of *L. acapulcensis* based on herbarium specimen data.

Mexico (Chiapas, Colima, Guerrero, Jalisco, México, Michoacán, Morelos, Oaxaca), Guatemala (Huehuetenango, Retalhuleu, Suchitepéquez), El Salvador, Nicaragua, and Costa Rica in clearings and disturbed areas in oak or coniferous forest, shrublands, tropical moist forest, and tropical dry forest, generally on volcanic soils (rarely on limestone, granite, or shale) at 450–2600 m in elevation (Fig. 7).

##### Common names and uses.

Mexico. Fruit edible; maravilla, huevo de cuervo, chimpin, tsibu (Dean 2004).

##### Phenology.

Flowering specimens have been collected from May to September; specimens with mature fruits have been collected between September and November. In the field, the first author has observed that the corollas open in the very early morning and close by late morning. The pollen in this species has a lemony fragrance.

##### Preliminary conservation status.

*Lycianthes acapulcensis* is a widespread species in Mexico and Central America, represented by 116 collections and occurring in seven protected areas. This species was given a preliminary conservation assessment by Anguiano-Constante et al. (2018) of Least Concern (LC).

##### Discussion.

*Lycianthes acapulcensis* is a very variable species, and it may be that some of the local forms deserve varietal status. It is variable in habit, indument (both trichome type and density), leaf shape, presence or absence of purple stripes on the corolla, fruit shape, and fruit coloration. However, the variation extremes are connected by intermediate populations (Dean 2004).

*Lycianthes acapulcensis* may be confused with *L. ciliolata* Bitter and *L. rzedowskii*. It is separated from those species by its combination of white corollas that may or may not have maroon to purple stripes and its pattern of filament lengths (the longest filament nearly always more than twice as long as the adjacent filaments). The anthers have a lemony fragrance, which is unlike that of any other anther (pollen) fragrance in similar Mexican and Guatemalan species of *Lycianthes*. The root shape (moniliform rather than fusiform segments) is helpful if underground parts are available for examination. On dried specimens, the length of the pedicels of the youngest mature flowers relative to their subtending leaves is often a useful character for separating *L. acapulcensis* from *L. ciliolata*. In the former, the length of those pedicels is usually less than that of the subtending leaves, while in *L. ciliolata* the length of the pedicels generally exceeds that of the leaves. *Lycianthes acapulcensis* appears to hybridize with *L. moziniana* and *L. rzedowskii* where the species co-occur (Dean 2004).

When Baillon (1888) published the name *Parascopolia acapulcensis*, he did not specify a specimen or herbarium. Similarly, when D’Arcy (1986b) transferred this species to *Lycianthes*, he did not cite a type specimen. There is only one specimen of this species seen by Baillon, and it is at P [P00070403]. Therefore, we are here designating specimen P00070403 as the lectotype of *P. acapulcensis*.

##### Representative specimens examined.

**Guatemala. Huehuetenango**: Mpio. Jacaltenango, 15.6744, -91.7353, 1627 m, 11 Jul 2006, *M. Véliz 17055* (BIGU). **Retalhuleu**: S. Sebastian, [14.55, -91.65], Sep 1874, *C. Bernoulli 2404* (GOET). **Suchitepéquez**: Patutlul, Finca Los Tarrales, [14.5364, -91.17], 300 m, 30 Jul 2004, *S. Montiel s.n.* (BIGU). **Mexico. Chiapas**: El Ranchito, sobre la carretera de los miradores, Parque Nacional Cañon del Sumidero, 16.8192, -93.0736, 1301 m, 24 Aug 2007, *J.A. Espinosa-Jiménez 306* (MO). **Colima**: Rancho El Jabalí, 22 air km NNW of Colima in the SW foothills of the Volcán de Colima, on border of Colima and Jalisco, [19.45, -103.7], 1300 m, 15 Jul 1991, *L. Vázquez-Villagran 887* (MEXU, DAV). **Guerrero**: Arroyo Cumiapa, a 1.44 km en línea recta al noroeste de la Comisaría de Arroyo Cumpiapa, sobre el camino que va a Cerro Zapote, en el terreno del Sr. Lauro Cortez, 16.8826, -98.6266, 531 m, 2 Aug 2017, *K. Velazco-G 40590* (DAV). **Jalisco**: Sierra del Halo, ca. 2 rd mi along rd to San Isidro (or Jilotlan) that leaves old Colima-Tecalitlán rd c. 7 rd mi S of Tecalitlán, [19.3171, -103.2696], 1340 m, 23 Nov 1991, *E. Dean 329* (DAV, MEXU). **México**: Cruz de los Pozitos, 18.9025, -99.7428, 2340 m, 20 Aug 2011, *F. D. Dorantes-Hernández 408* (MEXU). **Michoacán**: 4 km al sur de Doctor Miguel Silva, sobre la carretera a la Huacana, [19.1368, -101.7215], 500 m, 22 Jul 2001, *J. Rzedowski 53805* (MEXU). **Morelos**: noroeste de La Barranca de Atzingo, [18.9455, -99.2754], 1800 m, 12 Aug 1987, *E. Estrada-Loera 1708* (MEXU). **Oaxaca**: San José del Chilar, 17.7007, -96.9321, 683 m, 10 Nov 2009, *O. Vargas-Ponce 2084* (IBUG).

#### 2 Lycianthes amatitlanensis

Taxon classificationPlantae

(J.M.Coult. & Donn.Sm.) Bitter, Abh. Naturwiss. Verein Bremen 24 [preprint]: 441. 1919

Fig. 8

Solanum amatitlanense J.M.Coult. & Donn.Sm., Bot. Gaz. 37: 420. 1904. Type: Guatemala. Alta Verapaz: Cubilqüitz [Cubilhuitz], [15.6675, -90.4293], 350 m, Feb 1903, *H. von Tuerkheim 8488* (lectotype designated by Dean and Reyes 2018a, pg. 40: US [01269192]; isolectotypes: F [0073066F, acc. # 185826], M [M-0171813], NY [00138963, 00138964], US [01014253]).
Solanum sylvicola Brandegee, Univ. Calif. Publ. Bot. 6: 373. 1917. Type: Mexico. Chiapas: Finca Irlanda, Jun 1914, *C. Purpus 7315* (holotype: UC [acc. # 173378]; isotypes: HBG [HBG-511491], M [M-0171815]).

##### Type.

Based on *Solanum amatitlanense* J.M.Coult. & Donn.Sm.

**Figure 8.**
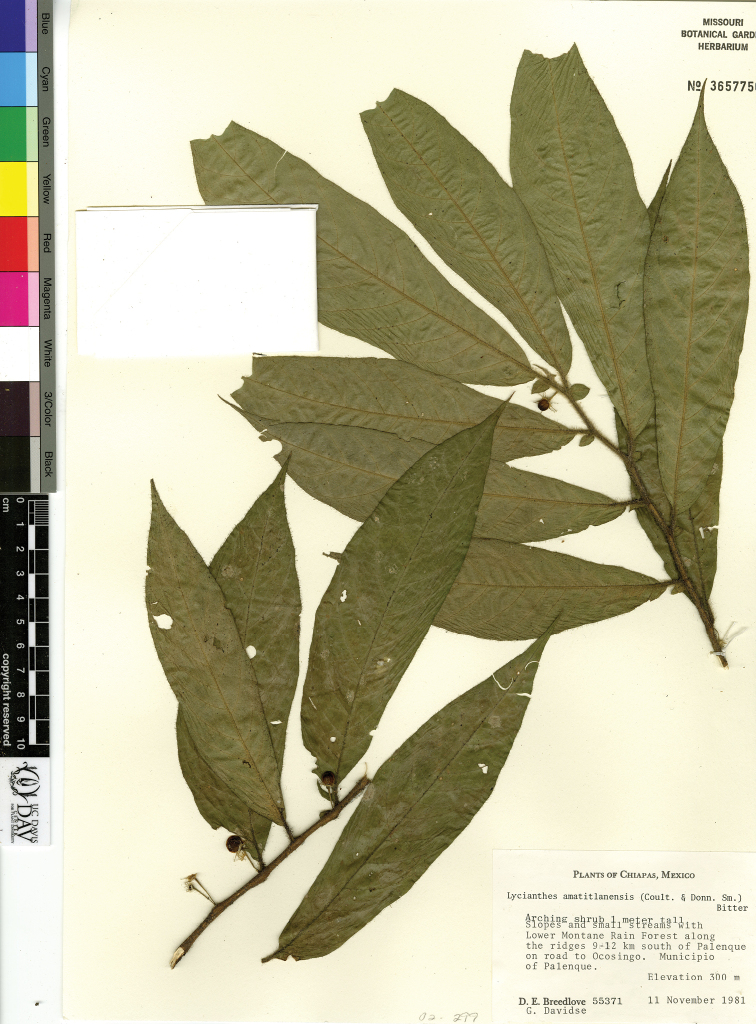
Image of herbarium specimen of *L. amatitlanensis*, *Breedlove 55371* (MO). Specimen used with permission from the Missouri Botanical Garden (http://www.tropicos.org).

##### Description.

Perennial herb to shrub, 0.4–4 m tall. Indument of off-white, tan or purplish (reddish), uniseriate, multicellular, simple, acute, straight to curved, eglandular, spreading or ascending trichomes 0.5–3 mm long. Stems green when young, moderately to densely pubescent, somewhat compressed upon drying in a plant press, light brown and woody with age; upper sympodial branching points usually monochasial with a few dichasial branching points. Leaves simple, the leaves of the upper sympodia usually paired and unequal in size, the larger ones with blades 6.5–26 × 1.5–8.5 cm, ovate, elliptic, or obovate, the smaller ones with blades 0.3–4 (7) × 0.2–2 (3.5) cm, usually ovate, the leaf pairs similar in texture, chartaceous, sparsely to densely pubescent, the trichomes along the midvein of the abaxial side spreading (at a 90 degree angle) to ascending (at a 45 degree angle), the base cuneate (sometimes rounded in the smaller leaves), usually oblique, the margin entire, usually delicately undulate, the apex acute to acuminate, the petiole 0.1–1.5 cm long, sometimes absent, the large leaf blades with (7) 10–22 primary veins on each side of the midvein. Flowers solitary, axillary, oriented horizontally to nodding; peduncles usually absent, sometimes present as a 1–3 mm long peg with overlapping pedicel scars; pedicels 4–12 mm and arching in flower, 6–16 mm long and erect to arching in fruit, moderately to densely pubescent; calyx 1–2 mm long, 2–3 mm in diameter, obconic to narrowly campanulate, moderately pubescent, the margin truncate to undulate, with 5–10 narrow, linear, spreading to reflexed appendages 0.8–4 mm long emerging 0.25–0.5 mm below the calyx rim; fruiting calyx slightly enlarged, widely bowl-shaped to plate-shaped, 1–2.5 mm long, 3.5–6 mm in diameter, the appendages very narrow and weak, to 5 mm long, sometimes withering in age; corolla 0.5–0.8 cm long, campanulate to reflexed in orientation, stellate in outline, divided 1/2 to 2/3 of the way to the base, interpetalar tissue present, white to pale yellow, adaxial markings unknown, moderately pubescent on abaxial surface with tuft of trichomes on distal end of lobe; stamens equal, straight, the filaments 1–2 mm long, glabrous, the anthers 2.5–3 mm long, lanceolate, free of one another, yellowish, glabrous, attenuate and abruptly narrowed at the tip, the narrowed portion ca. 0.5 mm long, poricidal at the tip, the pores ovate, dehiscing distally, not opening into longitudinal slits; pistil with glabrous ovary, the style 4–6 mm long, linear, straight, glabrous, widened distally into the stigma, the stigma capitate, decurrent down two sides. Fruit a berry, 5–8 mm long, 5–8 mm in diameter, globose, orange to red at maturity, glabrous, lacking sclerotic granules. Seeds 25–80 per fruit, 0.9–1.1 × 0.75–1 mm, compressed but not flat, sometimes with one shallow ridge, semi-circular, depressed ovate, triangular, or rhombic in outline, orange, the surface reticulum with tight, shallow serpentine pattern with shallow luminae.

##### Chromosome number.

Unknown.

##### Distribution and habitat.

**Figure 9.**
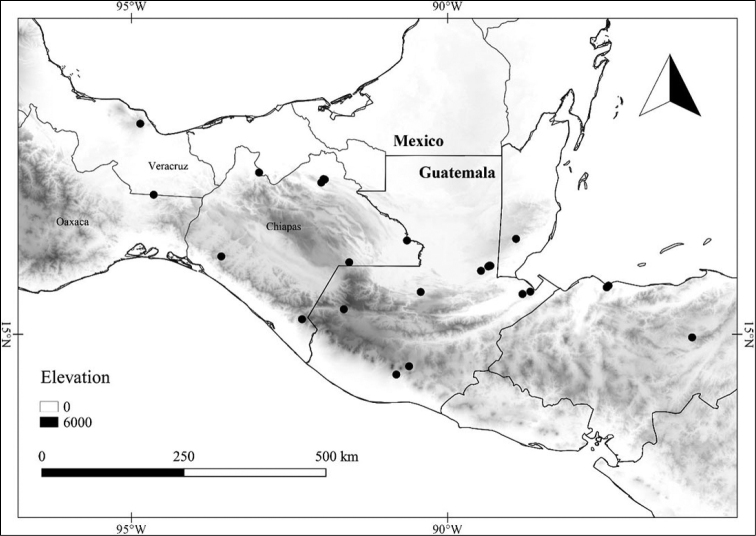
Map of geographic distribution of *L. amatitlanensis* from Mexico to Honduras based on herbarium specimen data.

Southern Mexico (Chiapas, Tabasco, Veracruz), Guatemala (Alta Verapaz, Escuintla, Guatemala, Huehuetenango, Petén), south to Belize, Honduras, Nicaragua, Costa Rica, Panama, and possibly South America. Tall forest, tropical rain forest, tropical moist forest, wet premontane forest, and cloud forest, in shady canyons, slopes, drainages (often near rivers or streams), sometimes in disturbed areas or coffee plantations, sometimes on limestone, usually 100–1000 m in elevation, rarely up to 1800 m (Fig. 9).

##### Common names and uses.

Guatemala: Alta Verapaz: kaki saki maï (*I. Kunkel 186, 398*); same location: maï (*I. Kunkel 211*).

##### Phenology.

Flowering specimens have been collected March through November; fruiting specimens have been collected May through February. De Nevers (1986) documented the pollination of *L. amatitlanensis* in eastern Panama. He described the flowers as being pendulous, positioned below the leaves. Pollinators (halictid bees) were observed visiting the flowers early morning to late afternoon and at 1 a.m. at night. No floral scents, nectar, or diurnal movements were noted. The flowers on most herbarium specimens are open, indicating that if there are diurnal movements, the flowers close for a very short time or only at night.

##### Preliminary conservation status.

*Lycianthes amatitlanensis* is a widespread species ranging from eastern Mexico to Panama, represented by 48 collections and occurring in nine protected areas. The EOO is 687,839.069 km^2^, and the AOO is 192 km^2^. Following the IUCN (2019) criteria, the preliminary assessment category is Least Concern (LC).

##### Discussion.

*Lycianthes amatitlanensis* is a wide ranging species with small white to pale yellow flowers (pubescent on the abaxial side of the corolla lobes and with tufted trichomes at the lobe tips) and long, coarse trichomes that spread away from the midvein on the abaxial side of the leaf (usually with some trichomes at an angle close to ninety degrees). *Lycianthes amatitlanensis* is morphologically similar and perhaps closely related to three other species occurring in Mexico and/or Central America: *L. glabripetala* (endemic to Mexico); *L. inconspicua* (Central America); and *L. inaequilatera* (Rusby) Bitter (Costa Rica, Panama and South America). *Lycianthes inconspicua* differs from *L. amatitlanensis* in having longer pedicels (15–30 mm in flower and 30–35 mm in fruit), appressed trichomes along the midvein of the abaxial side of the leaf, and ovate anthers with a shorter attenuate portion at the tip (ca. 0.25 mm long). *Lycianthes inaequilatera* has pedicels of similar length to those of *L. amatitlanensis*, but it has short, soft, appressed trichomes along the midvein on the abaxial side of the leaf. The Mexican species *Lycianthes glabripetala* has larger, nearly glabrous corollas and appressed, wavy trichomes along the midvein on the abaxial side of the leaf blade; *L. glabripetala* does not overlap in distribution with *L. amatitlanensis*. Where the distribution of *L. amatitlanensis* overlaps with *L. inconspicua* and *L. inaequilatera*, *L. amatitlanensis* tends to occur at lower elevations than the other two species. Intermediates between *L. inaequilatera* and *L. amatitlanensis* occur in Costa Rica and Panama, and these two species may eventually be treated as a single entity. *Lycianthes inaequilatera* is a South American species, originally described from Bolivia that has not been reported further north than Costa Rica. *Lycianthes amatitlanensis* is a Mexican and Central American species, originally described from Guatemala but reported in South America. Further study is needed to understand the ranges and variation of the two species. If united, *L. inaequilatera* is the earlier and correct name.

##### Representative specimens examined.

**Guatemala. Alta Verapaz**: Cubilquitz [Cubilhuitz], [15.6675, -90.4293], 350 m, July 1907, *H. von Tuerkheim 153* (US). **Escuintla**: Río Guacalate, 600 m, 16 Dec 1938, *P.C. Standley 60200* (US). **Guatemala**: Barranca de Eminencia, 1400 ft, Feb 1892, *J. Donnell Smith 1457* (US). **Huehuetenango**: between Ixcan and Finca San Rafael, Sierra des los Cuchumatanes, 200–800 m, 24 Jul 1942, *J.A. Steyermark 49396*, (NY). **Izabal**: Cerro San Gil, 15.6333, -88.8167, 803 m, 8 Feb 2012, *M. Véliz 23523* (BIGU). **Petén**: La Cumbre, in zapotal, on parcela de José León, 3 km east, 14 Aug 1976, *C.L. Lundell 20140* (LL). **Mexico. Chiapas**: Ejido Tres Picos, 16.2272, -93.5808, 1780 m, 19 Apr 2002, *A. Reyes-García 4437* (MEXU). **Tabasco**: vicinity of Teapa, along road between Teapan and Tacotalpa, 3.1 m. E of Teapa along stream and limestone cliffs ca 1/4 mi S of Hwy, 17.55, -92.9833, 150 m, 19 Feb 1987, *T.B. Croat 65349* (MO). **Veracruz**: Mpio. Jesús Carranza, lomas al S de Pob. 2 (ca. 3 km al S de entronque de terracería La Laguna-Sarabia con camino al N a Pob. 2), 17.2, -94.65, 150 m, 8 Jul 1988, *T. Wendt 6064* (MO).

#### 3 Lycianthes anomala

Taxon classificationPlantae

Bitter, Abh. Naturwiss. Verein Bremen 24 [preprint]: 514. 1919

Fig. 10

##### Type.

Mexico. Veracruz: Río Blanco, Orizaba, *Bourgeau 2536* (lectotype designated by Dean and Reyes 2018a, pg. 40: BR [000000552905]; isolectotypes: G [G00415142], GH [00077065], K [K000063121], MPU [MPU310734], P [P00385091, P00385092], S [cited by Bitter (1919), but not seen]).

**Figure 10.**
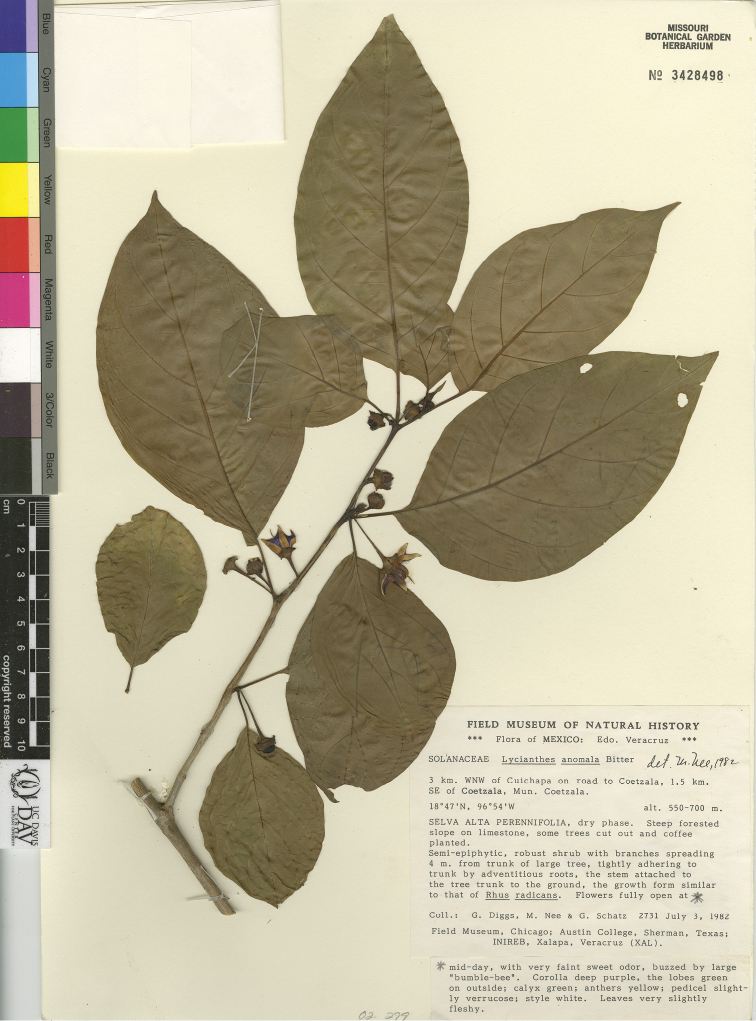
Image of herbarium specimen of *L. anomala*, *Diggs 2731* (MO). Specimen used with permission from the Missouri Botanical Garden (http://www.tropicos.org).

##### Description.

Herb, shrub, to tree, sometimes epiphytic, semi-epiphytic, or vine-like, erect, 2–10 (15) m tall. Indument of tan to brownish, uniseriate, multicellular, simple or dendritically branched, eglandular, spreading trichomes 0.25–0.5 mm long. Stems green when young, glabrous to sparsely pubescent, not compressed upon drying in a plant press, quickly becoming woody (glossy pale grey with longitudinal wrinkles upon drying); upper sympodial branching points mostly monochasial. Leaves simple, the leaves of the upper sympodia usually paired and unequal in size, the larger ones with blades 10–25 × 5–15 cm, ovate, oblong, or elliptic (rarely obovate), the smaller ones with blades 2–10 × 1.5–5 cm, orbicular to ovate, the leaf pairs similar in texture, coriaceous, usually glabrous adaxially, abaxially with tufts of trichomes in the axils of the major veins, the base rounded to cuneate, usually oblique, the margin entire, usually undulate, the apex acute to acuminate, the petiole 1–4 cm long, the larger leaf blades with 6–9 primary veins on each side of the midvein. Flowers solitary or in groups of 2–6, axillary, erect; peduncles absent or present as a short stub with many pedicel scars, 5–10 mm long; pedicels 8–20 mm and erect in flower, to 35 mm long and erect in fruit, glabrous; calyx 4–5 mm long, 6–8 mm in diameter, widely campanulate, glabrous, coriaceous in texture, the margin truncate, very well developed, with 5–10 reflexed appendages, 0.25–1 mm long (sometimes just a bulge), connate at their bases, emerging 1–2 mm below the calyx rim; fruiting calyx enlarged, widely bowl-shaped, 3–4 mm long, 8–10 mm in diameter, the appendages 0.5–2 mm long, reflexed as a connate unit; corolla 1–1.5 cm long, reflexed in orientation, stellate in outline, divided 1/3–1/2 of the way to the base, with scant interpetalar tissue, the lobes purple adaxially, nearly glabrous; stamens equal, straight, the filaments ca. 1 mm long, glabrous, the anthers 4–6 mm long, elliptic, connate at edges to adjacent anther, forming a cone, yellow, glabrous, poricidal at the tips, the pores dehiscing distally and opening into longitudinal slits that extend ca. 1/3 of the way from apex to base, the slit forming between the thecae of adjacent anthers; pistil with glabrous ovary, the style ca. 8 mm long, linear, straight to curved, glabrous, the stigma truncate to capitate. Fruit a berry, 7–10 mm long, 7–10 mm in diameter, globose to depressed globose, green to white when immature, purple at maturity, glabrous, lacking sclerotic granules. Seeds ca. 100 per fruit, 1–1.5 × 1–1.25 mm, flattened, slightly curved, triangular to depressed-ovate in outline, yellow, the surface reticulum with tight, minute serpentine pattern with shallow luminae.

##### Chromosome number.

Unknown.

##### Distribution and habitat.

**Figure 11.**
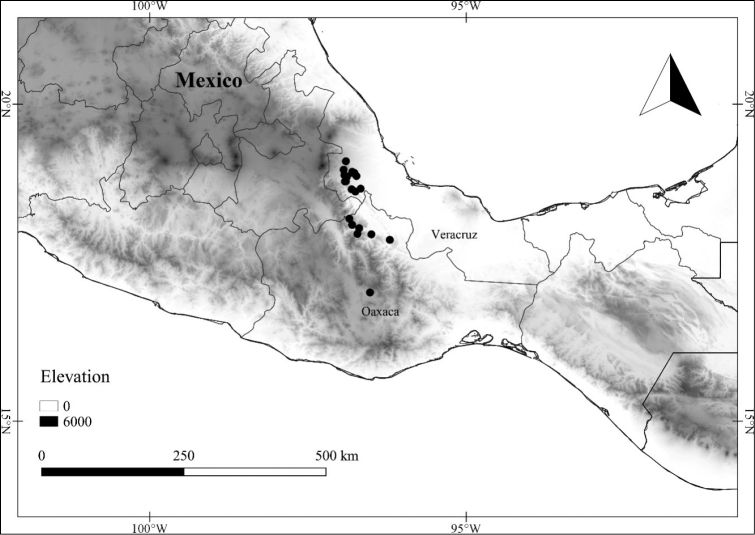
Map of geographic distribution of *L. anomala* based on herbarium specimen data.

Mexico (Oaxaca, Veracruz), in tropical moist forest and cloud forest, sometimes in sandy soil or on limestone, often in disturbed areas, such as secondary forest or coffee plantations, 450–1300 m in elevation (Fig. 11).

##### Common names and uses.

None known.

##### Phenology.

Flowering specimens have been collected June through November. Specimens with immature fruits have been collected June through March. Specimens with mature fruits have been collected June through February. Possibly flowering and fruiting throughout the year in some locations. The diurnal movements of the corolla of this species are unknown, but it has been noted that the flowers are sometimes open at midday (Nee 1986).

##### Preliminary conservation status.

*Lycianthes anomala* is a Mexican endemic, represented by 20 collections, none of which are in protected areas. The EOO is 7,552.355 km^2^, and the AOO is 80 km^2^. Based on the IUCN (2019) criteria, the preliminary assessment category is Endangered (EN).

##### Discussion.

This species is similar to the Central American species *L. synanthera* in having pale woody stems with petioles that darken upon drying, tufts of trichomes in the vein axils of the abaxial side of the leaf, and fused anthers that dehisce by slits formed between adjacent thecae. It differs from that species in having connate, reflexed appendages on the calyx (versus no appendages in *L. synanthera*) and purple fruits (versus yellow/orange fruits in *L. synanthera*). Reyes-Cornejo (2015) indicated that this species is also found in Central America, and we have studied specimens from Costa Rica and Panama that resemble *L. synanthera* but have calyx appendages. These specimens need further study.

##### Representative specimens examined.

**Mexico**. **Oaxaca**: Santa María Tlalixtac, orillas del Río Cóndor, brecha entre Santa María Tlalixtac y Chiquihuitlán de Benito Juárez, [17.9541, -96.7179], 675 m, 25 Nov 2004, *G. Juárez-García 877* (MEXU). **Veracruz**: Ladera de cerro al E de Coetzala, 18.7806, -96.9144, 650 m, 11 Nov 2001, *A. Rincón G. 2811* (IEB, MEXU, XAL).

#### 4 Lycianthes armentalis

Taxon classificationPlantae

J.L.Gentry, Phytologia 26: 269. 1973

Fig. 12

##### Type.

Mexico. Quintana Roo: Cobá, east of the ruins, in advanced deciduous forest, [20.4800, -87.7300], 1 Jun 1938, *C.L. & A.A. Lundell 7800* (holotype: US [00027868]; isotypes: A [00936248], F [0072898F, acc. # 1307280], US [01014254, 01014255]).

**Figure 12.**
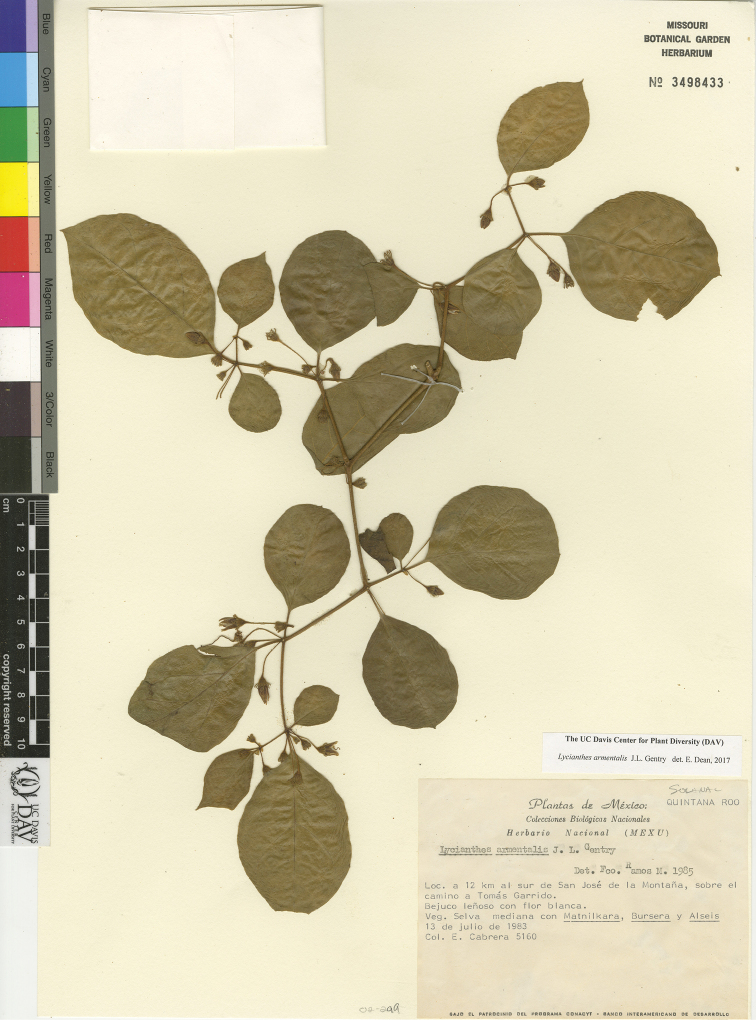
Image of herbarium specimen of *L. armentalis*, *Cabrera 5160* (MO). Specimen used with permission from the Missouri Botanical Garden (http://www.tropicos.org).

##### Description.

Clambering shrub to vine, 0.5–4 (7) m tall. Indument of pale yellow to orange-brown, uniseriate, multicellular, sessile to stalked, multangulate-stellate, eglandular, spreading trichomes 0.1–0.75 mm long, 0.2–0.5 mm in diameter, the rays (3) 5–8 (10) per whorl, straight, rarely rebranched, often with an enlarged sphere where the rays join, rarely some dendritically branched trichomes also present. Stems green to light brown when young, sparsely to densely pubescent (appearing like dense felt), not compressed when dried in a plant press, becoming woody early; upper sympodial branching points a mixture of monochasial and dichasial branching, the branching divaricate (diverging at wide angles). Leaves simple, the leaves of the upper sympodia paired or not, the leaves often appearing like they terminate short shoots with the pairs arranged at 90 degree angles, the pairs unequal in size, the larger ones with blades 3–9 (13) × 2–4.5 (7) cm, the smaller ones (often not developing) with blades 0.9–2.5 × 0.8–2 cm, the leaf pairs similar in shape, the blades ovate, elliptic, obovate, or suborbicular, thick chartaceous, sparsely to moderately pubescent (denser on the abaxial side, especially along the veins, the adaxial side sometimes nearly glabrous), the base cuneate to rounded, sometimes oblique, the margin entire, usually irregularly undulate, the apex rounded, obtuse, acute or acuminate, the petiole 0.3–1.2 cm long, the larger leaf blades with 3–5 primary veins on each side of the midvein. Flowers solitary or in groups of 2–6, axillary, erect; peduncles absent; pedicels 6–13 (20) mm long and erect in flower, 9–25 mm long and erect in fruit, moderately pubescent; calyx 2.5–3 mm long, 3–4 mm in diameter, campanulate, moderately pubescent, the margin truncate, with 10 spreading linear appendages 0.5–3.5 mm long emerging ca. 0.3 mm below the calyx rim; fruiting calyx enlarged, widely bowl-shaped to rotate, 2–4 mm long, 6–10 mm in diameter, the appendages to 5 mm long; corolla 0.8–1.2 cm long, campanulate to rotate in orientation, stellate in outline (divided ca. 1/4–1/2of the way to the base), with abundant interpetalar tissue, white, with a few scattered trichomes on the adaxial side of the lobes near the major veins, sparsely to moderately puberulent on the lobes near the major veins abaxially; stamens slightly to very unequal, straight, the four short filaments 0.5–1 (1.5) mm long, the one long filament 1–2 (3) mm long, glabrous, the anthers 3–4 mm long, elliptic to lanceolate, free of one another, yellow, sparsely pubescent on the inner face, poricidal at the tips, the pores ovate, terminal, dehiscing distally, not opening into longitudinal slits; pistil with glabrous ovary, the style 7–8 mm long, linear, straight to curved, glabrous, the stigma oblong, decurrent down two sides. Fruit a berry, 5–12 mm long, 5–12 mm in diameter, globose, red-orange when mature, glabrous, lacking sclerotic granules. Seeds 20–30 per fruit, 2.5–3 × 2–2.5 mm, flattened, circular to depressed ovate in outline, thickened on the edges, thin and semi-transparent in the center, yellow to dark orange, the surface reticulum in the center nearly smooth, the edges with minute serpentine pattern with shallow luminae.

##### Chromosome number.

Unknown.

##### Distribution and habitat.

**Figure 13.**
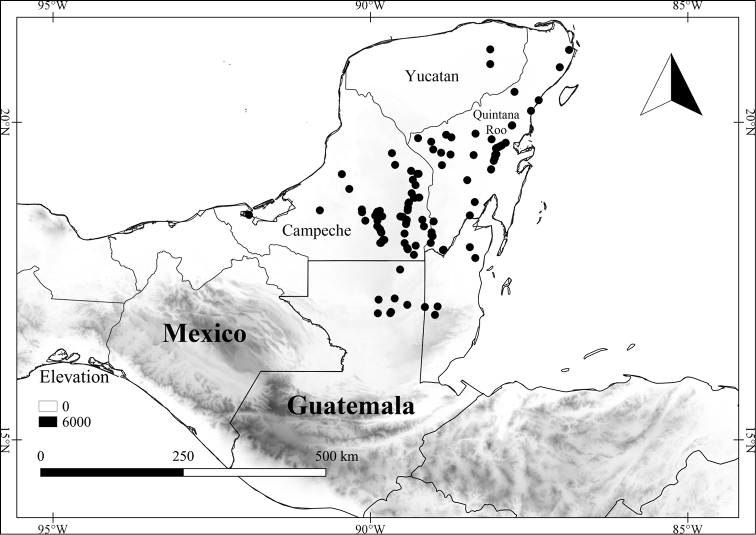
Map of geographic distribution of *L. armentalis* based on herbarium specimen data.

Mexico (the Yucatán Peninsula, including Campeche, Quintana Roo, Yucatán), Guatemala (El Progreso, Petén), and Belize, in forest (often secondary), usually in tropical rain forest, tropical moist forest, or tropical dry forest, sometimes on limestone, 0–500 m in elevation (Fig. 13).

##### Common names and uses.

None known.

##### Phenology.

Flowering specimens have been collected from May to October; specimens with mature fruits have been collected June to March. The timing of the diurnal corolla movements for this species are not known, but many specimens have been collected with closed flowers indicating that the flowers are open for a limited period during the day, probably in the early morning.

##### Preliminary conservation status.

*Lycianthes armentalis* is a widespread species ranging from southeastern Mexico to Belize represented by 116 collections and occurring in seven protected areas; unfortunately, the habitat of this species is vulnerable. The EOO is 122,903.444 km^2^ (LC) and AOO is 404 km^2^ (EN). Based on the IUCN (2019) criteria, the preliminary assessment category is Least Concern (LC).

##### Discussion.

*Lycianthes armentalis* is often confused with *L. sideroxyloides*, *L. rafatorresii*, and L. scandens var. scandens (previously known as *L. lenta* (Cav.) Bitter). *Lycianthes armentalis* differs from those species in its combination of multangulate-stellate (not geminate-stellate) trichomes, widely divaricate branching, calyx appendages that are not enlarged at the tip, white, shallowly stellate corollas, and unequal stamens. *Lycianthes sideroxyloides* has geminate-stellate trichomes, appendages that are enlarged at the tip, deeply stellate corollas, and equal stamens. Lycianthes scandens var. scandens has purple, mostly entire corollas and lacks widely divaricate branching. *Lycianthes rafatorresii* has similar flowers and trichomes to *L. armentalis* but lacks widely divaricate branching and has calyx appendages that are enlarged at the tip. *Lycianthes armentalis* occurs at relatively low elevations on the Yucatán Peninsula, a distribution that overlaps with L. scandens var. scandens, but not the other two species.

##### Representative specimens examined.

**Guatemala. El Progreso**: Tulumaje, [14.9256, -90.0469], 346 m, 23 Nov 2003, *R. Ávila 71* (BIGU). **Petén**: Mpio. Melchor de Mencos, sitio arqueológico El Naranjo, 17.1319, -95.2606, 297 m, 18 Jun 2009, *L. Velásquez 413* (BIGU). **Mexico. Campeche**: a 2 km al E de X-Mejía, 19.2347, -89.3592, 150 m, 24 Jun 2005, *E. Martínez- Salas 38011* (MEXU). **Quintana Roo**: camino a Zafarrancho, 0.73 km al N de Zafarrancho, 19.52, -88.8864, 91 m, 22 Aug 2005, *E. Martínez-Salas 38092* (MEXU). **Yucatán**: carretera Noholal-Sudzal-Chico, 19.75, -89.25, 18 Nov 1992, *F. May 766* (MEXU).

#### 5 Lycianthes arrazolensis

Taxon classificationPlantae

(J.M.Coult. & Donn.Sm.) Bitter, Abh. Naturwiss. Verein Bremen 24 [preprint]: 388. 1919

Fig. 14

Solanum arrazolense J.M.Coult. & Donn.Sm., Bot. Gazette 37: 421. 1904. Type: Guatemala. Guatemala: Arrazola, 5500 ft [1,600 m], Apr 1893 [protologue says Apr, 1893 but writing on label looks like Mar or Mai, 1893], *E.T. Heyde et E. Lux 4736* (holotype: F [0073068F, acc. # 264950, photo negative 49338]; isotypes: B [not seen, cited by Bitter (1919), probably destroyed], G [G00379120], GH [01652206], K [K000585744], US [01014241, 00027461], probably elsewhere).

##### Type.

Based on *Solanum arrazolense* J.M.Coult. & Donn.Sm.

**Figure 14.**
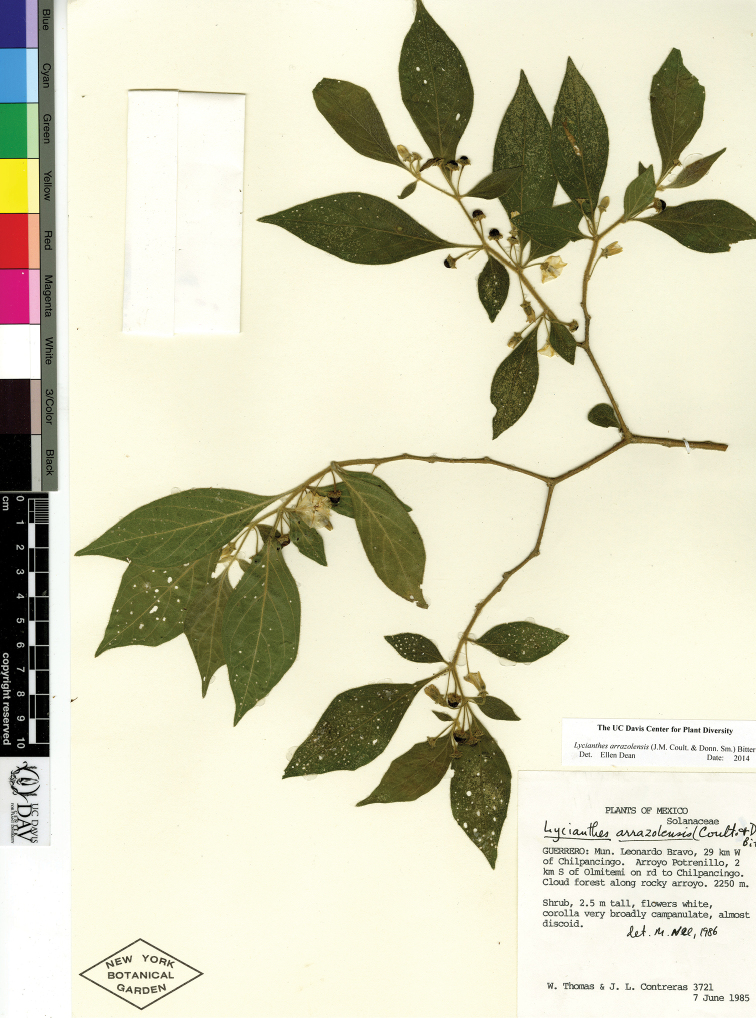
Image of herbarium specimen of *L. arrazolensis*, *Thomas 3721* (NY). Specimen used with permission from the William and Lynda Steere Herbarium, New York Botanical Garden.

##### Description.

Shrub, 0.9–5 m tall, sometimes vining or arching through neighboring vegetation. Indument of light yellow (sometimes appearing tan or off-white), uniseriate, multicellular, simple, eglandular, spreading or appressed-ascending trichomes 0.1–1.5 (1.75) mm long, sometimes the stems with very small appressed trichomes between longer spreading trichomes. Stems green to violet when young (drying tan) with maroon/purple lenticular vertical striations (drying blackish), sparsely to densely pubescent, not much compressed when dried in a plant press, becoming light brown and woody with age; upper sympodial branching points monochasial or dichasial. Leaves simple, the leaves of the upper sympodia usually paired and unequal in size, the larger ones with blades 7.5–22 × (1.8) 3–9 (11) cm, the smaller ones with blades (1) 3–9 (12.5) × (0.4) 1.2–5.7 (7) cm, the leaf pairs similar in shape, the blades ovate, elliptic, or obovate (rarely lanceolate), chartaceous, glabrous to densely pubescent, the trichomes usually densely spreading outward (towards the margins) along the abaxial veins, especially at the base of the main vein, the base cuneate to attenuate, sometimes oblique, the margin entire, usually irregularly undulate, the apex acuminate, the petiole 0.2–3 cm long, sometimes absent, the larger leaf blades with 5–7 primary veins on each side of the midvein. Flowers solitary or in groups of 2–10 (19), axillary, oriented horizontally; peduncles absent; pedicels 4–15 mm long and erect in flower, 6–16 (21) mm long and erect in fruit, sparsely to densely pubescent; calyx (1) 1.5–2.5 (3) mm long, 2–3 (3.5) mm in diameter, obconic to campanulate, sparsely to densely pubescent (sometimes nearly glabrous in Oaxaca), the margin truncate, with 10 spreading, linear appendages 0.5–2.5 mm long (atypically to 5 mm in low elevation Guerrero populations) emerging 0.5–1 mm below the calyx rim; fruiting calyx enlarged, bowl-shaped to rotate, 1–2 mm long, 4–7 mm in diameter, the appendages to 2.5 (4) mm long (probably longer in Guerrero); corolla 0.6–1.2 (1.6) cm long, rotate to campanulate in orientation, mostly entire in outline (with shallow notches), with abundant interpetalar tissue, white to pale violet, adaxially sometimes with purple stripes along the major veins of the lobes or with three green spots located between the short stamens, glabrous, the abaxial side of the lobes green, glabrous to sparsely puberulent near the veins; stamens unequal, straight, the four short filaments 0.5–2 mm long, the one long filament (1.25) 3–4 (5) mm long, glabrous, the anthers 2.5–4 (4.5) mm long, lanceolate, free of one another, yellow, glabrous, poricidal at the tips, the pores ovate, the pores of the longest stamen dehiscing toward the style, the pores of the shorter stamens usually dehiscing away from the style (sometimes dehiscing distally, rarely inward), not opening into longitudinal slits; pistil with glabrous ovary, the style (5) 6.5–9 (10) mm long, linear, straight to curved upward at the tip, glabrous, the stigma oblong, decurrent down two sides. Fruit a berry, 5–10 (11) mm long, 5–10 (11) mm in diameter, globose, orange to red at maturity, glabrous, lacking sclerotic granules. Seeds (3) 10–108 per fruit, 1.2–2.5 (3) × 1–2.5 mm, flattened, elliptic, irregularly triangular, or oval in outline, not obviously notched (if slightly indented, indentation is usually less than 0.3 mm), yellow-orange, surface reticulum rough with indistinct serpentine pattern with shallow luminae.

##### Chromosome number.

2n = 24 (Gentry and Pearce 1977).

##### Distribution and habitat.

**Figure 15.**
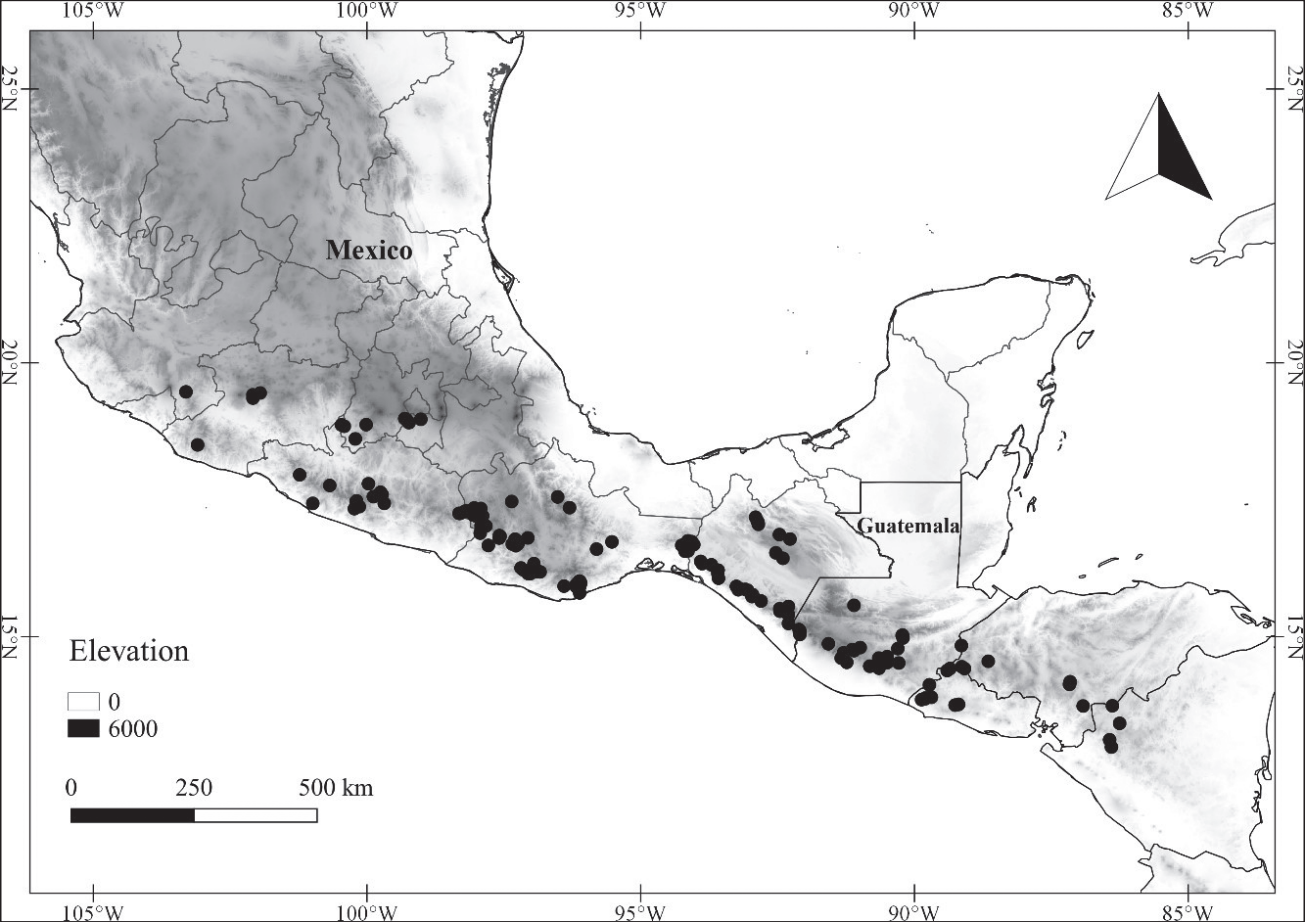
Map of geographic distribution of *L. arrazolensis* based on herbarium specimen data.

Mexico (Chiapas, Guerrero, Jalisco, México, Michoacán, Morelos, Oaxaca), Guatemala (Alta Verapaz, Baja Verapaz, Chimaltenango, El Progreso, Escuintla, Guatemala, Quiché, Sacatepéquez, Sololá, Suchitepéquez), Belize, El Salvador, Honduras, and Nicaragua in wet canyons and drainages, often in riparian forest or disturbed forest, in oak, oak/pine, and tropical dry forest (higher elevation populations are often in hardwood cloud forest; south of Guatemala, it has been collected in high-elevation, dwarf cloud forest and *Cupressus* forest), 500–3000 m in elevation (Fig. 15).

##### Common names and uses.

None known.

##### Phenology.

Flowering specimens have been collected from February through October; specimens with mature fruits have been collected January through December. The corollas of this species are open in the morning and closed by late morning.

##### Preliminary conservation status.

*Lycianthes arrazolensis* is a widespread species ranging from southern Mexico to Nicaragua, represented by 193 collections and occurring in eight protected areas. The EOO is 380,617.675 km^2^, and the AOO is 676 km^2^. Following the IUCN (2019) criteria, the preliminary assessment category is Least Concern (LC).

##### Discussion.

This species is very similar to *Lycianthes tricolor*. It can be easily distinguished from *L. tricolor* by seed shape. The seeds of *L. tricolor* have a definite sharp notch that is usually deeper than 0.5 mm, whereas the seeds of *L. arrazolensis* lack this notch. In some Mexican *L. arrazolensis* populations in the states of Morelos and México, the seeds are shallowly indented on one side, but this indentation is usually less than 0.25 mm and never sharply notched (Dean et al. 2017a). Non-fruiting specimens can be challenging to identify. If a specimen was collected below 1700 m in elevation, it is most likely to be *Lycianthes arrazolensis*. In addition, the following non-seed characters can be helpful: the calyx rim of *L. arrazolensis* tends to be more prominent, often protruding beyond the appendage insertion by over 0.5 mm, while the calyx rim of *L. tricolor* is usually less than 0.5 mm; the appendages of *L. arrazolensis* tend to bend away from the rim, exposing the rim, while the appendages of *L. tricolor* are oriented closer to the rim and corolla, hiding the rim; the pores of the short stamens in *L. arrazolensis* usually face away from the style, while those of *L. tricolor* usually face toward the style; the pedicels of the oldest, largest flowers and the pedicels of the most mature fruits of *L. arrazolensis* are usually shorter than those of *L. tricolor*, although there is overlap in this characteristic; and the leaves of typical *L. arrazolensis* tend to have obvious geminate leaf pairs with elliptic to obovate laminas and leaf bases often attenuate into the petiole, while in *L. tricolor* the small geminate leaf often abscises early, and the laminas are more ovate with a less-attenuate leaf base (Dean et al. 2017a).

There is a wide range of morphological variation within *Lycianthes arrazolensis* as circumscribed here, especially in leaf size, leaf shape, and density of pubescence. This is particularly true of populations in the state of Oaxaca and neighboring Guerrero where small-leaved forms with very dense pubescence as well as large-leaved forms with sparse pubescence are found. The populations with larger leaves and sparser pubescence are usually found below 1,500 m, while those with denser pubescence are usually found above 2,000 m. The degree of variation found in this species is worthy of more study (Dean et al. 2017a).

##### Representative specimens examined.

**Guatemala. Alta Verapaz**: 3 kms de Villa Hermosa, [14.87, -91.57], 1400 m, 16 May 1963, *A. Molina R. 12362* (NY): **Baja Verapaz**: Mpio. San Jerónimo, Santa Elena la Cumbre, 15.0292, -90.2167, 2263 m, 12 Nov 2009, *A. Cóbar 1980* (BIGU). **Chimaltenango**: Volcán Acatenango, Aldea Quisache, 14.5181, -90.2844, 500 m, 19 May 2004, *M. Velíz 15260* (MEXU). **El Progreso**: 15 km N of Morazo, [14.975, -90.2067], 17 Jul 1970, *W.E. Harmon 3207* (MO). **Escuintla**: 2 km W of San Vicente Pacaya, [14.4225, -90.6464], 1500 m, 31 May 1970, *W.E. Harmon 2438* (MO). **Guatemala**: Villa Canales, Fea. San Agustín Las Minas, 14.5264, -90.495, 1839 m, 12 Aug 2010, *L. Velásquez 1460* (BIGU). **Quiché**: Nebaj, Batzchocolá, 15.5714, -91.1035, 1300 m, 1 Aug 2017, *E. Tribouillier 3* (DAV). **Sacatepéquez**: Mpio. Alotenango, astillero municipal, 14.4619, -90.8147, 1332 m, 20 Apr 2011, *M. Véliz 23680* (BIGU). **Sololá**: Patanatic, 6 km to Panajachel [14.7634, -91.1354], 1700 m, 20 Sep 1971, *A. Molina-R. 26664* (MEXU). **Suchitepéquez**: Volcán Santa Clara, 1.5, 2 miles W of Finca El Naranjo [14.6077, -91.3364], 1250 m, 1 Jun 1942, *J.A. Steyermark 46787* (NY). **Mexico. Chiapas**: Ejido Sierra Morena, 16.1522, -93.5902, 1550 m, 30 May 2002, *A. Reyes-García 4788* (MEXU). **Guerrero**: 10 km al suroeste de Puerto del Gallo, sobre el camino a Atoyac, 17.4717, -100.1969 2100 m, 13 Mar 2007, *Y. Ramírez-Amezcua 964* (DAV, IEB, NY). **Jalisco**: Mpio. Tecalitlán, 46 km Carr. Cd. Guzmán-Pihuamo, por brecha Llanitos-Canutillo, a 24 km, [19.4692, -103.3064], 1750 m, 14 May 1988, *V. Pichardo A. 42* (NY). **México**: El Potrero, Cañada de agua fría, 3 km al sur de Tlatlaya, 18.6106, -100.2139, 1650 m, 6 Aug 2004, *I. Martínez de la Cruz 212* (MEXU). **Michoacán**: Mpio. Coalcomán, 4.5 km (en línea recta) al oeste de Las Joyas sobre una brecha maderera, 18.5014, -103.0939, 1970 m, 29 Aug 2008, *V.W. Steinmann 6347* (DAV). **Morelos**: Barranca Tepecapa, 18.9683, -99.0156, 1849 m, 17 Jul 2010, R. *Hernández-Cardenás 445.2* (IEB). **Oaxaca**: Dto. Tlaxiaco. Santiago Yosondua. Paraje El Limón, a 100 m del Río Yutama, 16.8, -97.5833, 1470 m, 17 Jul 2013, *D. Sandoval-Gutiérrez 967* (MEXU).

#### 6 Lycianthes barbatula

Taxon classificationPlantae

Standl. & Steyerm., Publ. Field Mus. Nat. Hist., Bot. Ser. 23(5): 228 1947

Fig. 16

##### Type.

Guatemala. Suchitepéquez: Volcán Santa Clara, between Finca El Naranjo and upper slopes, 1250–2650 m, 23 May 1942, *J.A. Steyermark 46653* (holotype: F [0072901F, acc.# 1148517]; isotypes: NY [00138703], US [00624009]).

**Figure 16.**
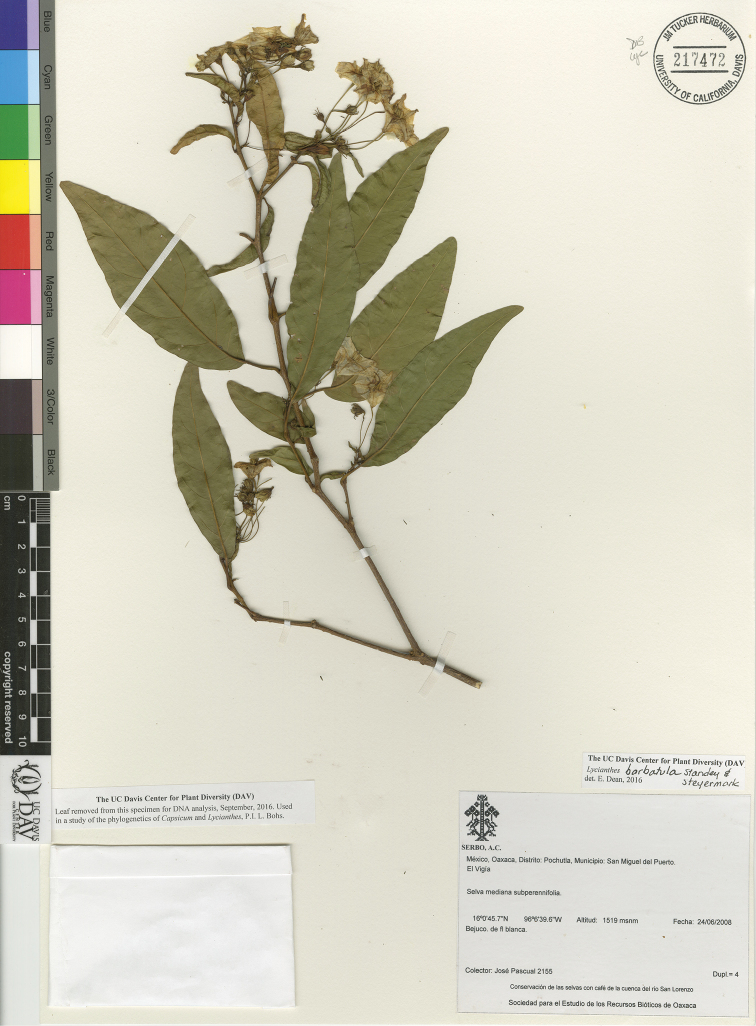
Image of herbarium specimen of *L. barbatula*. *Pascual 2155* (DAV). Image used with permission of the UC Davis Center for Plant Diversity.

##### Description.

Shrub, erect to scandent (sometimes described as a vine), 3–5 m tall. Indument of small white, uniseriate, multicellular, simple, curved, eglandular, appressed-ascending trichomes 0.1–1 mm long. Stems green when young, glabrous to sparsely pubescent, ribbed to angled upon drying in a plant press, woody with age; upper sympodial branching points monochasial or dichasial. Leaves simple, the leaves of the upper sympodia usually paired and unequal in size, the larger ones with blades 4–15 × 1.5–6.5 cm, the smaller ones with blades 1–9 × 0.7–4 cm, the leaf pairs usually similar in shape, the blades narrowly ovate to elliptic or obovate, thick chartaceous, glabrous except for tufts of trichomes located in the axils along the midvein of the abaxial side, the base cuneate to attenuate, sometimes oblique, the margin entire, usually undulate, the apex acute to acuminate, the petiole 0.2–2 cm long, sometimes absent, the larger leaf blades with 5–10 primary veins on each side of the midvein. Flowers solitary or in groups of 2–8, axillary, oriented horizontally to nodding; peduncles absent; pedicels 20–30 mm long and arching in flower, to 40 mm long and spreading to deflexed in fruit, glabrous to sparsely pubescent; calyx 1.5–2.5 mm long, 3–4 mm in diameter, obconic to campanulate, sparsely pubescent, the margin truncate, with 10 linear, erect to spreading appendages 0.5–2 mm long, emerging 0.5 mm below the calyx rim; fruiting calyx usually enlarged, widely campanulate to bowl-shaped, sometimes splitting, 3–4 mm long, 5–8 mm in diameter, the appendages 2.5–4 mm long, spreading; corolla 0.7–2 cm long, rotate to campanulate in orientation, entire to slightly stellate in outline, divided 0–1/5 of the way to the base, with abundant interpetalar tissue, adaxially white with lavender ring near stamen insertion, abaxial color unknown, glabrous; stamens equal or nearly so, the filaments 2–2.5 mm long, glabrous, the anthers 2.5–3 mm long, elliptic, free of one another, brownish-yellow, glabrous, poricidal at the tips, the pores round, large, dehiscing distally, not opening into longitudinal slits; pistil with glabrous ovary, the style 6–7 mm long, linear, straight, glabrous; stigma capitate to oblong, decurrent down two sides. Fruit a berry, 10–12 mm long, 10–12 mm in diameter, globose to ovoid, changing from green to white as it matures, possibly remaining white or changing to blue-grey or purple at maturity, glabrous, lacking sclerotic granules. Seeds ca. 20 per fruit, 3–3.5 × 2–2.5 mm, flattened, triangular to depressed ovate in outline, tan to orange-brown, the surface reticulum with minute serpentine pattern with shallow luminae.

##### Chromosome number.

Unknown.

##### Distribution and habitat.

**Figure 17.**
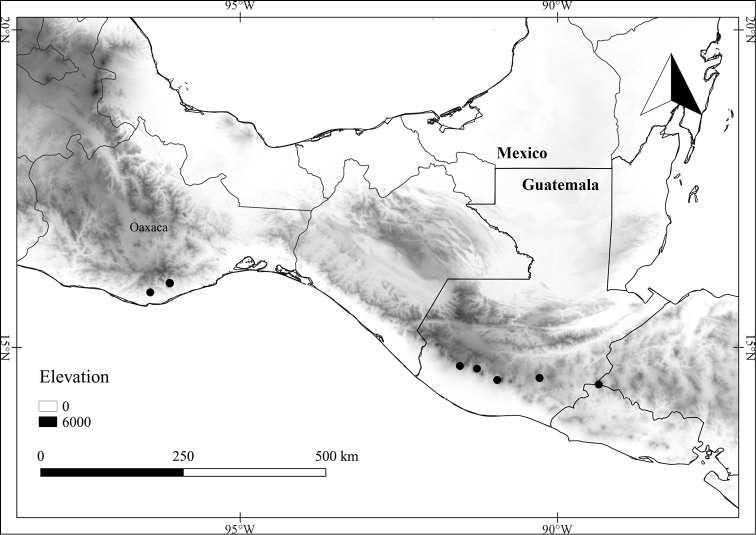
Map of geographic distribution of *L. barbatula* based on herbarium specimen data.

Mexico (Oaxaca, probably also Chiapas) and Guatemala (Chimaltenango, Quezaltenango, Suchitepéquez), in tropical moist forest, cloud forest, and near coffee plantations, 920–1600 m in elevation. [Note: type specimen has a high elevational range of 2650 m, but the specimen may not have been collected that high.] Our knowledge of the distribution and ecology of this species is incomplete due to the paucity of specimens in herbaria (Fig. 17).

##### Common names and uses.

None known.

##### Phenology.

Flowering specimens have been collected in May and June. Specimens with mature fruits have been collected in September and January. Immature fruits have been collected in January. The phenological record is incomplete due to a paucity of specimens. The corollas on the specimens of this species are often open; this indicates that the corollas are open for a substantial amount of time each day.

##### Preliminary conservation status.

*Lycianthes barbatula* is a rarely collected species of Mexico and Guatemala, represented by only 11 collections, only one of which is from a protected area (Cuenca del Lago Atitlán, Guatemala). The EOO is 20,500.215 km^2^, and the AOO is 28 km^2^. Following the IUCN (2019) criteria, the preliminary assessment category is Endangered (EN).

##### Discussion.

*Lycianthes barbatula* is morphologically similar to *L. manantlanensis* and *L. orogenes* with which it shares relatively thick leaves (thick chartaceous to coriaceous), relatively glabrous foliage, long, delicate, arching pedicels, white corollas, and equal to nearly equal stamens with yellowish anthers, sometimes with a brownish connective. *Lycianthes barbatula* differs from the other two species in having longer calyx appendages and tufts of trichomes in the vein axils along the midvein of the abaxial side of the leaf blade. The fruit color of *L. barbatula* has been recorded on several specimen labels as being white, and this is also the color given in Gentry and Standley (1974), but there is uncertainty as to whether this is the final color at maturity or a transitional color, as other specimens mention blue-grey or blue-purple fruits.

##### Representative specimens examined.

**Guatemala. Chimaltenango**: Volcán Acatenanago, Aldea Quisache, 14.51806, -90.2844, 1500 m, 19 May 2004, *M. Véliz 15261* (BIGU, MEXU). **Quetzaltenango**: above Finca Montevideo, along Barranco Espinazo and tributary of Río Pantaleón, lower and middle southwestern slopes of Volcán Fuego, 1200–1600 m, 20 Sep 1942, *J.A. Steyermark 52055* (US). **Suchitépequez**: Volcán Santa Clara, between Finca El Naranjo and upper slopes. 1250–2650 m, 23 May 1942, *J.A. Steyermark 46653* (US). **Mexico. Oaxaca**: Dto. Pochutla, El Vigía, 16.0125, -96.1108, 1519 m, 24 Jun 2008, *J. Pascual 2155* (DAV).

#### 7 Lycianthes breedlovei

Taxon classificationPlantae

E.Dean, Phytotaxa 409: 265. 2019

Fig. 18

##### Type.

Mexico. Chiapas: Mpio. La Independencia, third ridge along logging road from Las Margaritas to Campo Alegre, [16.4756, -91.8234], 2300 m, 6 May 1973, *D. Breedlove 34793* (holotype: CAS [480622]; isotypes: LL [00226970], MO [acc. # 2602916]).

**Figure 18.**
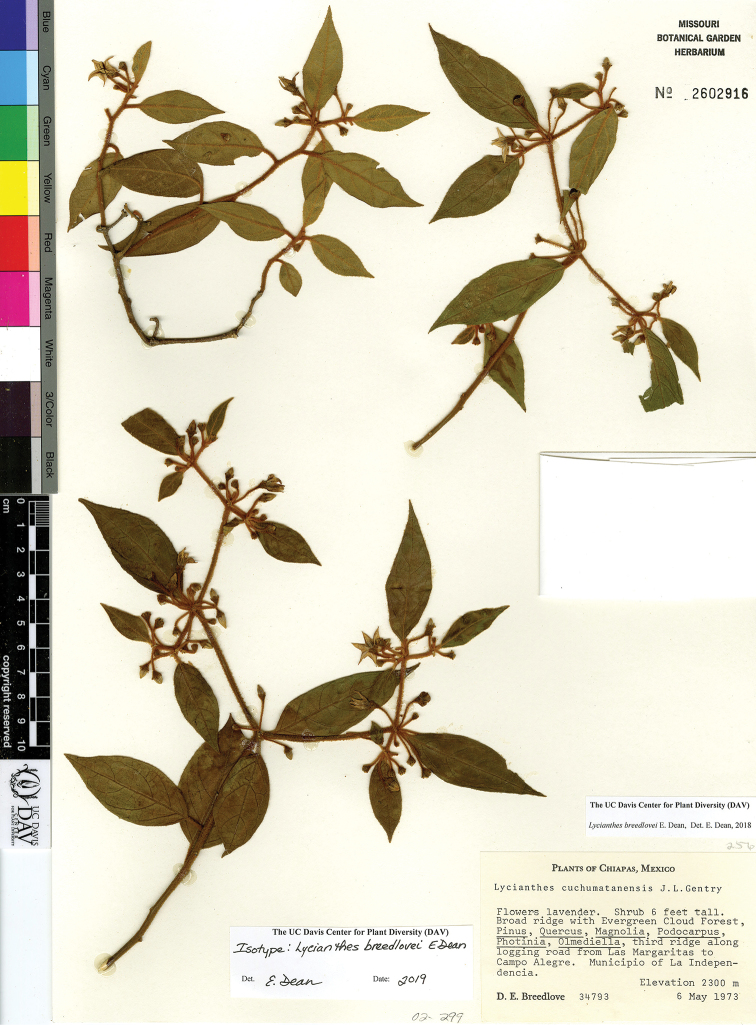
Image of isotype of *L. breedlovei*, *Breedlove 34793* (MO). Specimen used with permission from the Missouri Botanical Garden (http://www.tropicos.org).

##### Description.

Vine to scandent shrub, 2–3.5 (5) m tall (perhaps taller, if a vine). Indument of orange to pale yellow (yellow-grey), uniseriate, multicellular, stalked, multangulate-stellate, eglandular, spreading trichomes 0.25–1.5 (2) mm long, ca. 0.75 in diameter, the rays 3–5 per whorl, straight, often rebranched, sometimes several times. Stems pale green (drying tan) when young, moderately to densely pubescent, not compressed when dried in a plant press, becoming brown and woody with age; upper sympodial branching points a mixture of monochasial and dichasial, the branching divaricate, the branches diverging at wide angles. Leaves simple, the leaves of the upper sympodia usually unpaired, if paired, then unequal in size, the larger ones with blades 3.5–10 × 1.5–4.5 cm, the smaller ones (usually not developing) with blades 1–3.5 × 0.5–2 cm, the leaf pairs similar in shape, the blades ovate, elliptic, or obovate, chartaceous, sparsely to densely pubescent (denser on the abaxial side, especially along the veins), the base cuneate to rounded, sometimes oblique, the margin entire, usually irregularly undulate, the apex acute to acuminate, the petiole 0.3–1 cm long, the larger leaf blades with 4–6 primary veins on each side of the midvein. Flowers solitary or in groups of 2–5, axillary, oriented horizontally; peduncles absent; pedicels 9–16 mm long and erect in flower, 10–25 mm long and erect in fruit, densely pubescent; calyx 2.5–3.5 mm long, 3.5–4.5 mm in diameter, campanulate, pale green (sometimes nearly translucent) with dark ribs, sparsely to densely pubescent, the margin truncate, with 10 spreading linear appendages 1–3 mm long emerging ca. 0.5 mm below the calyx rim; fruiting calyx enlarged, widely bowl-shaped to rotate, 2–3 mm long, 6–8 mm in diameter, the appendages to 5 mm long; corolla 0.9–1.5 cm long, rotate in orientation, shallowly stellate in outline, divided ca. 1/2 of the way to the base, with abundant interpetalar tissue, white to lilac, adaxially with darker purple stripes on the lobes, sparsely pubescent with few scattered trichomes, abaxially usually densely puberulent on the lobes (best seen in bud); stamens slightly unequal, straight, the four short filaments 0.5–1 mm long, the one long filament 1–2 mm long, glabrous, the anthers 3–4 mm long, elliptic, free of one another, yellow, sometimes pubescent on the inner face along the connective, poricidal at the tips, the pores ovate, dehiscing distally, not opening into longitudinal slits; pistil with glabrous ovary, the style 6–8 mm long, linear, straight to curved, glabrous, the stigma oblong, decurrent down two sides. Fruit a berry, 4–10 mm long, 5–11 mm in diameter, depressed globose, orange when mature, glabrous, lacking sclerotic granules. Seeds 5–30 per fruit, 3–3.5 × 2–2.5 mm, flattened, thickened on the edges, reniform to depressed ovate in outline, usually with small notch on one side, orange, the surface reticulum with tight serpentine pattern and shallow luminae, the margin thickened and rougher in texture than the center.

##### Chromosome number.

Unknown.

##### Distribution and habitat.

**Figure 19.**
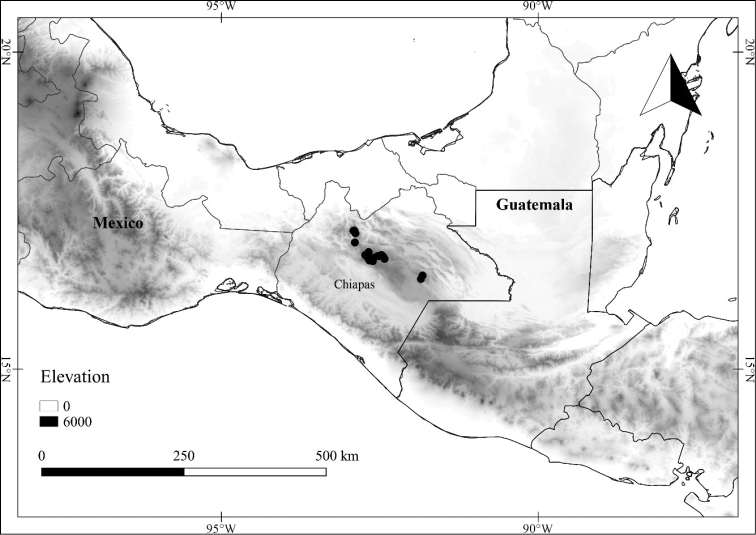
Map of geographic distribution of *L. breedlovei* based on herbarium specimen data.

Mexico (Chiapas), in cloud forest, often in oak forest, sometimes associated with *Pinus*, *Abies*, *Magnolia*, or *Podocarpus*, sometimes near disturbed areas, such as milpas, 2000–3000 m in elevation (Fig. 19).

##### Common names and uses.

Mexico. Chiapas: chichol mut (Tzeltal) (*C. Santíz R. 854*); penko antivo (Tzotzil) (*C. Santíz R. 904*); tunatzak (Tzeltal) (*A. Médez Ton 5054*).

##### Phenology.

Flowering specimens have been collected from April through July; specimens with mature fruits have been collected from August to November. The timing of the diurnal movements of the corolla of this species is not known, but many specimens have been collected with open flowers indicating that the flowers are open for an extended period during the day.

##### Preliminary conservation status.

*Lycianthes breedlovei* is restricted to cloud forest habitat in the state of Chiapas, represented by 18 collections, only one of which is from a protected area. This species was previously given a preliminary assessment by Dean et al. (2019a) of Endangered.

##### Discussion.

*Lycianthes breedlovei* is a shrub to vine with zigzag branching due to widely divaricate branching angles, yellow to orange branched pubescence, white flowers with violet markings, and unequal stamens. It is closely related to *L. hortulana* Standley & L.O.Williams, described from Honduras. The two species are geographically isolated from one another, with no populations of either species known to occur in Guatemala. They have diverged from one another in pedicel length (*L. hortulana* flowers have pedicels 3–9 mm long vs 9–16 mm long), flower size (*L. hortulana* has corollas that are 0.6–1 cm long vs 0.9–1.5 cm), corolla pubescence (*L. hortulana* has very sparse pubescence on the abaxial side of the corolla lobes vs dense), and stamen length (*L. hortulana* has equal stamens vs usually unequal) (Dean et al. 2019a).

##### Representative specimen examined.

**Mexico. Chiapas**: Mpio. Tenejapa, along the road to the town of Matzam, ca. 1.5 km from the eastern outskirts of the town of Las Ollas where the road forks, about 2.6 km from the intersection with the San Cristóbal de las Casas-Tenejapa road, just west and upslope of the settlement of Paraje Cruz Tzibaltic, on ridge where there is an intersection with an undeveloped road, 16.7832, -92.5275, 2484 m, 13 Sep 2017, *E. Dean 9531* (DAV226596).

#### 8 Lycianthes caeciliae

Taxon classificationPlantae

Bitter, Abh. Naturwiss. Verein Bremen 24 [preprint]: 429. 1919

Fig. 20

##### Type.

Mexico. Veracruz: District Cordoba, Cerro de Chocomán, 12 May 1907, *C. Seler & E. Seler 5168* (holotype: B [not seen, cited by Bitter (1919), probably destroyed]; isotype: GH [00936203]).

**Figure 20.**
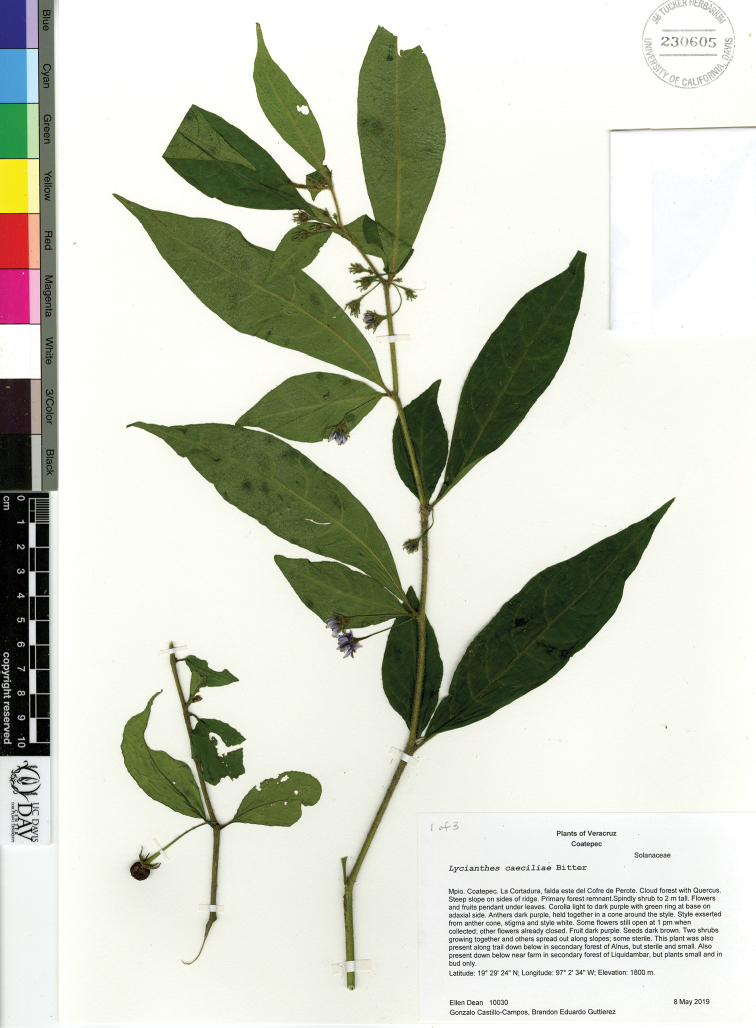
Image of herbarium specimen of *L. caeciliae*, *Dean 10030* (DAV). Image used with permission of the UC Davis Center for Plant Diversity.

##### Description.

Shrub to small tree, 1.5–3 m tall. Indument of off-white to light brown, uniseriate, multicellular, simple (very rarely furcate), acute, eglandular, appressed to spreading trichomes 0.25–1 mm long, these usually remaining cylindrical and acute upon drying. Stems green to purple-green when young, glabrous to moderately pubescent, partly to fully compressed upon drying in a plant press, brown and woody with age; upper sympodial branching points mostly monochasial with a few dichasial branching points. Leaves simple, the leaves of the upper sympodia usually paired and unequal in size, the larger ones with blades 4–16 × 1–5 cm, elliptic to obovate, the smaller ones with blades 1–6 × 0.5–3 cm, ovate, lanceolate, or obovate, the blades of both the large and small leaves chartaceous to thick chartaceous, glabrous to moderately pubescent, denser on the veins, the base cuneate, sometimes oblique, the margin entire, undulate, the apex acute to acuminate, the petiole to 0.8 cm long, sometimes absent, the larger leaf blades with 4–6 primary veins on each side of the midvein. Flowers solitary or in groups of 2–3, axillary, oriented horizontally to nodding; peduncles absent; pedicels 15–30 mm long, arching to deflexed in flower, to 42 mm long, arching to deflexed in fruit, glabrous to moderately pubescent; calyx 3–3.5 mm long, 4–4.5 mm in diameter, campanulate, green, sometimes with a purple hue, glabrous to sparsely pubescent, the margin truncate, with 10 spreading, linear-subulate appendages 2–5 mm long emerging 0.5–1 mm below the prominent, undulating calyx rim; fruiting calyx enlarged, widely bowl-shaped, 2–2.5 mm long, 6–8 mm in diameter, the appendages widening but not significantly elongating, to 7 mm long; corolla 0.7–1.6 cm long, campanulate to reflexed in orientation, stellate in outline, divided 1/3–2/3 of the way to the base, with interpetalar tissue, purple adaxially with green at the base of each lobe near the stamen insertion, purple abaxially, sometimes with a single white line down the middle, nearly glabrous; stamens equal, straight, the filaments 0.5–2 mm long glabrous, the anthers 4–4.5 mm long, ovate to lanceolate, free of one another, purple, glabrous, poricidal at the tips, the pores ovate, dehiscing toward the style, not opening into longitudinal slits; pistil with glabrous ovary, the style 5–7.5 mm long, linear, glabrous; stigma capitate to oblong. Fruit a berry, 7–13 mm long, 6–13 mm in diameter, globose to ovoid, dark purple at maturity, glabrous, lacking sclerotic granules. Seeds 10–50 per fruit, 2.5–3 × 3–4 mm, compressed but not flat, depressed ovate or reniform (with small notch) in outline, brown, the surface reticulum with a tight, serpentine pattern with deep luminae, with fibrils protruding from the cell walls.

##### Chromosome number.

Unknown.

##### Distribution and habitat.

**Figure 21.**
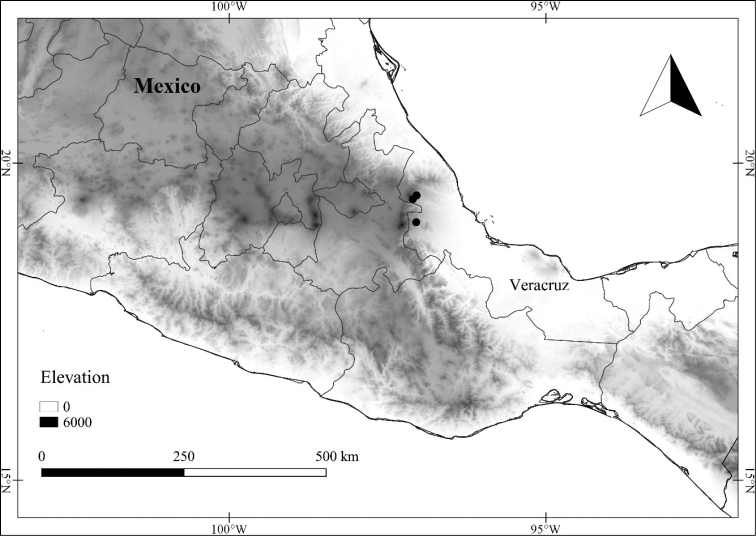
Map of geographic distribution of *L. caeciliae* based on herbarium specimen data.

Mexico (Veracruz), on the eastern slopes of two volcanos, Cofre de Perote and Citlaltépetl [Orizaba], in cloud forest and oak forest, 1750–2250 m in elevation (Fig. 21).

##### Common names and uses.

None known.

##### Phenology.

Specimens have been collected with both flowers and mature fruits April to September. In the field, the first author observed that most of the corollas of this species were closed by 1 pm, but some of the corollas still remained open at that time. Corollas opening for the first time (the smallest on the plant) are a deep purple, while older, larger flowers are a pale purple. The green ring at the base of the corolla is more prominent in older flowers.

##### Preliminary conservation status.

*Lycianthes caeciliae* is a Mexican species of vulnerable cloud forest habitat in the state of Veracruz that is known from only four locations, with only one in a protected area (La Cortadura Ecological Reserve). The EOO is 149.767 km^2^, and the AOO is 12 km^2^. Following the IUCN (2019) criteria, the preliminary assessment category is Endangered (EN).

##### Discussion.

As detailed elsewhere (Dean et al. 2019b), *Lycianthes caeciliae* is a species that was poorly collected until the 21^st^ century. Other than the type collection, it was just collected one other time in the 20^th^ century by Matuda. It was then collected numerous times at La Cortadura on the slopes of Cofre de Perote by Gonzalo Castillo-Campos between 2005 and 2007. The species is most abundant in original oak cloud forest where it is in flower and fruit; although observed by the first author in secondary forest of *Alnus* or *Liquidambar* at lower elevations in the area of La Cortadura, the plants are small and often sterile. *Lycianthes caeciliae* is closely related to *L. pilifera*, a Oaxacan cloud forest endemic, with which it shares simple, straight, spreading trichomes that do not collapse upon drying, corollas with purple and green coloration, purple anthers, purple coloration in the calyx, and dark purple fruit with large brown seeds. *Lycianthes caeciliae* could be confused with *L. stephanocalyx*, a rhizomatous herb to subshrub which also occurs in Veracruz, but at lower elevations. *Lycianthes stephanocalyx* differs from *L. caeciliae* in having small incurved trichomes, yellow connivent anthers, red fruits, and tan seeds (Dean et al. 2019b).

##### Representative specimen examined.

**Mexico. Veracruz**: Mpio. Coatepec, La Cortadura, falda este del Cofre de Perote, 19.4900, -97.0427, 1800 m, 8 May 2019, *E. Dean 10030* (DAV230605).

#### 9 Lycianthes ceratocalycia

Taxon classificationPlantae

(Donn.Sm.) Bitter Abh. Naturwiss. Verein Bremen 24 [preprint]: 498. 1919

Fig. 22

Brachistus ceratocalycius Donn.Sm. Bot. Gaz. 48: 297. 1909. Type: Guatemala. Dept. Alta Verapaz: In silva montana prope Cobán, 1600 m, Jan 1908, *H. von Tuerkheim II 2060* (lectotype designated by Dean and Reyes 2018a, pg.40: US [00624002]; isolectotypes: BM [000514916], BR, E [E00190707], F [0072757F, acc. # 680315], G [G00379123], M [M-0171534], NY [00007074], US [00624001], W [acc. # 1908-5994]).

##### Type.

Based on *Brachistus ceratocalycius* Donn.Sm.

**Figure 22.**
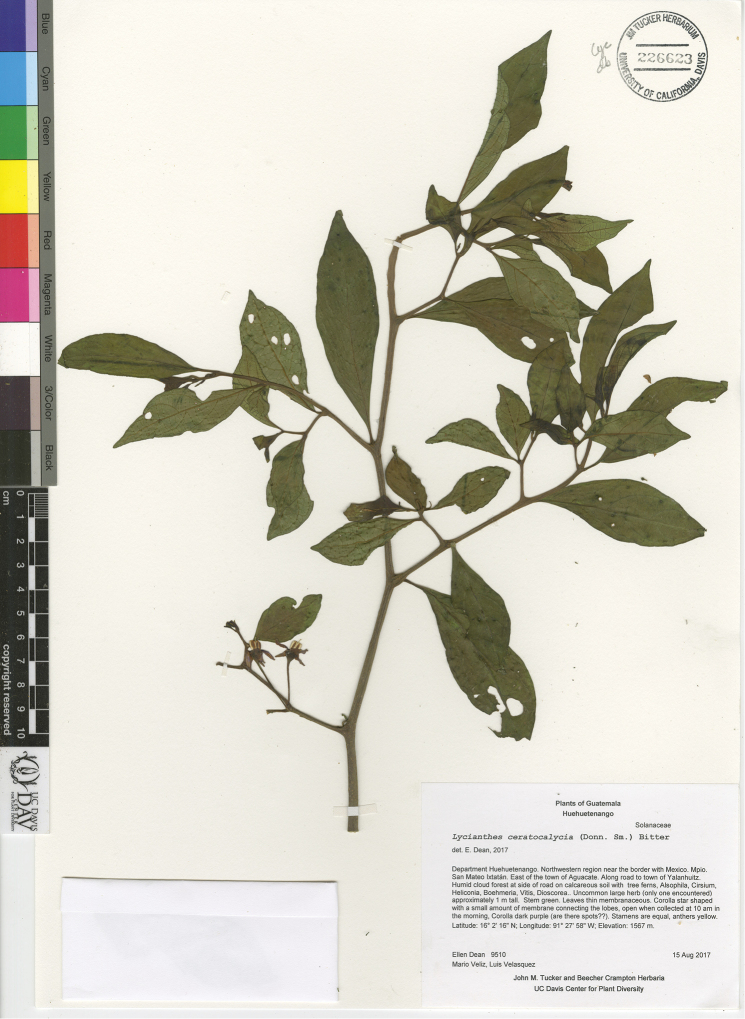
Image of herbarium specimen of *L. ceratocalycia*, *Dean 9510* (DAV). Image used with permission of the UC Davis Center for Plant Diversity.

##### Description.

Herb to shrub, erect, 0.5–2 m tall, sometimes epiphytic. Indument of off-white to tan, uniseriate, multicellular, simple, eglandular, appressed-ascending trichomes 0.1–0.2 mm long. Stems green when young, the surface with brownish scurfy horizontal lines (perpendicular to stem axis), glabrous to moderately pubescent, compressed upon drying in a plant press, woody with age; upper sympodial branching points monochasial or dichasial. Leaves simple, the leaves of the upper sympodia usually paired and unequal in size, the larger ones with blades 4–14 × 2–4 cm, the smaller ones with blades 1.5–6 × 1–2.5 cm, the leaf pairs usually similar in shape, the blades ovate to elliptic (sometimes narrowly), membranaceous to chartaceous, sometimes purple abaxially, glabrous to sparsely pubescent, the base cuneate to attenuate, sometimes oblique, the margin entire, usually undulate, the apex acute to acuminate, the petiole 0.2–0.7 cm long, sometimes absent, the larger leaf blades with 4–6 primary veins on each side of the midvein. Flowers in groups of 2–5 (15), axillary, erect; peduncles absent; pedicels 7–17 mm and erect in flower, to 25 mm long and erect in fruit, glabrous to moderately pubescent; calyx 2.5–4 mm long, 4–5 mm in diameter, campanulate, puberulent with very small trichomes, the margin truncate, undulate, very well developed, with 10 very short, reflexed appendages 0.25–1 mm long emerging 1–1.5 mm below the calyx rim; fruiting calyx enlarged, widely bowl-shaped, 2–2.5 mm long, 6–8 mm in diameter, the appendages not changing in length; corolla 0.5–1.2 cm long, campanulate to reflexed in orientation, stellate in outline, divided at least 3/4 of the way to the base, with scarce interpetalar tissue, lilac to purple adaxially and abaxially, with deeper purple markings adaxially near the stamens, pubescent abaxially with very short trichomes; stamens equal, straight, the filaments ca. 1 mm long, glabrous, the anthers 3–4.5 mm long, lanceolate, free of one another, pale yellow (sometimes with brown shiny pigment on the outer side), glabrous, poricidal at the tips, the pores round, dehiscing distally, not opening into longitudinal slits; pistil with glabrous ovary, the style 6–7 mm long, linear, straight to curved at the tip, glabrous; stigma capitate, decurrent down two sides. Fruit a berry, 5–9 mm long, 5–9 mm in diameter, globose to ovoid, red at maturity, glabrous, lacking sclerotic granules. Seeds 10–60 per fruit, 1.5–2.5 × 1.5–2 mm, flattened with slightly raised and thickened edges, depressed ovate to circular in outline, tan to orange, with the surface reticulum with minute serpentine pattern and shallow luminae.

##### Chromosome number.

Unknown.

##### Distribution and habitat.

**Figure 23.**
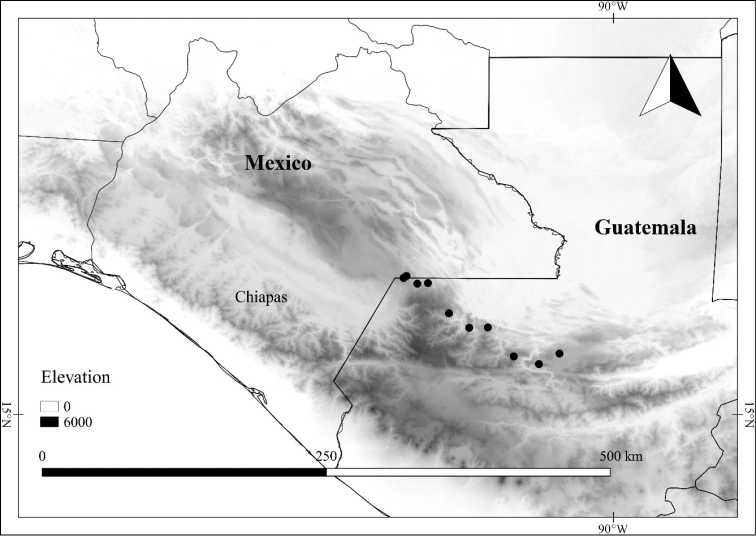
Map of geographic distribution of *L. ceratocalycia* based on herbarium specimen data.

Mexico (Chiapas) and Guatemala (Alta Verapaz, Huehuetenango, and Quiché), in cloud forest, including oak forest, sometimes in disturbed or open areas, often on calcareous soil, 1300–2100 m in elevation (Fig. 23).

##### Common names and uses.

None known.

##### Phenology.

Flowering specimens have been collected April to September. Specimens with mature fruits have been collected in August. In the field in Mexico and Guatemala, the first author observed that the corollas are open in the morning and closed in the afternoon.

##### Preliminary conservation status.

*Lycianthes ceratocalycia* is a species restricted to Guatemala and lands immediately adjacent in Chiapas that is known from only 10 locations, only one of which is from a protected area (Lagunas de Montebello, Mexico). The EOO is 2,169.823 km^2^, and the AOO is 40 km^2^. Following the IUCN (2019) criteria, the preliminary assessment category is Endangered (EN).

##### Discussion.

*Lycianthes ceratocalycia* was rarely collected until recently. It can be distinguished from similar species with stellate corollas and equal stamens by the scurfy horizontal lines on the young stems.

##### Representative specimens examined.

**Guatemala. Alta Verapaz**: San Cristóbal, Finca Pamac II, 15.3986, -90.5883, 2146 m, 16 Aug 2015, *A. Borrayo 5* (BIGU). **Huehuetenango**: northwestern region near the border with Mexico. Mpio. San Mateo Ixtatán, E of the town of Aguacate, along road to town of Yalanhuitz, 16.0377, -91.4662, 1567 m, 15 Aug 2017, *E. Dean 9510* (DAV). **Quiché**: camino a Amachel, 15.6878, -90.9928, 1553 m, 25 Jun 2006, *R. Ávila 3038* (MEXU, MEXU). **Mexico. Chiapas**: Mpio. La Trinitaria, Parque Nacional Lagunas de Montebello, at an outlook (mirador) above and to the E of Dos Lagunas, on the west side of Hwy 307 to the E of Lago Tziscao, ca. 7 km E of the intersection of Hwy 307 and the road to Cinco Lagos, 16.0939, -91.6361, 1461 m, 14 Sep 2017, *E. Dean 9532* (DAV).

#### 10 Lycianthes chiapensis

Taxon classificationPlantae

(Brandegee) Standl., Publ. Field Mus. Nat. Hist., Bot. Ser. 11: 173. 1936

Solanum chiapense Brandegee, Univ. Calif. Publ. Bot. 6: 192. 1914. Type: Mexico. Chiapas: Finca Irlanda, Jun 1914, *C. Purpus 7328* (holotype: UC [UC175061]; isotypes: BM [BM000775080]; GH [00077473]; MO [acc. # 765046]; NY [00138972]; US [00027504]).

##### Type.

Based on *Solanum chiapense* Brandegee.

#### 10a Lycianthes chiapensis var. chiapensis

Taxon classificationPlantae

Fig. 24

Lycianthes nyssifolia Bitter, Abh. Naturwiss. Verein Bremen 24 [preprint]: 366. 1919.

##### Type.

Guatemala. Suchitepéquez: Las Nubes, Nov 1875, *K. Bernoulli & O. Cario 2397* (holotype: GOET [GOET003443]).

**Figure 24.**
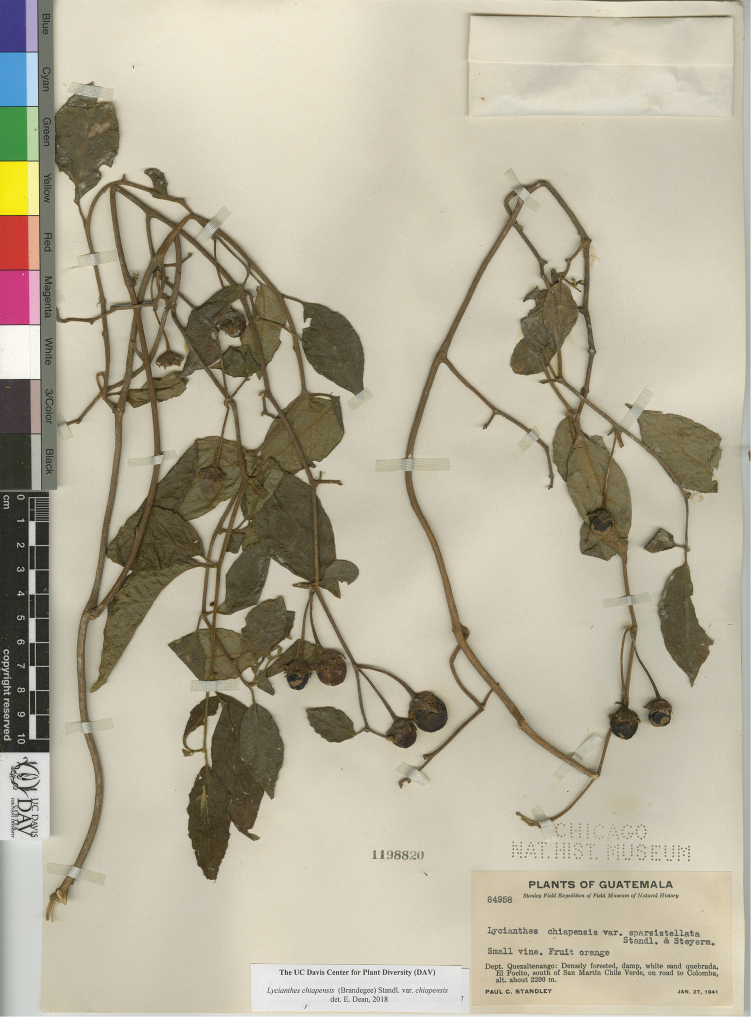
Image of herbarium specimen of L. chiapensis var. chiapensis, *Standley 84958* (F). Specimen used with permission from the Field Museum of Natural History.

##### Description.

Woody vine to 10 m tall, probably taller. Indument of tan, pale yellow to brown, uniseriate, multicellular, stalked, multangulate-stellate (also some simple and dendritic), eglandular, spreading trichomes 0.1–0.5 (1 mm) long, 0.75–1 mm in diameter, the rays of the multangulate trichomes 3–6 per whorl, straight, rarely rebranched. Stems greenish-tan when young, sparsely to moderately pubescent, not compressed when dried in a plant press, becoming woody with age; upper sympodial branching usually monochasial, sometimes dichasial. Leaves simple, the leaves of the upper sympodia sometimes paired and unequal in size, the larger ones with blades 4–14 × 2–4.5 cm, the smaller ones with blades 2–7 × 1.5–3.5 cm, the leaf pairs similar in shape, the blades ovate, elliptic or obovate (sometimes the small geminate leaf nearly orbicular), thick chartaceous, sparsely to moderately pubescent especially along the veins (sometimes nearly glabrous), the base cuneate to rounded, sometimes oblique, the margin entire, usually undulate, the apex acute to acuminate (rarely rounded on smaller leaves), the petiole 0.2–2.5 cm long, the larger leaf blades with 4–6 primary veins on each side of the midvein. Flowers solitary or in groups of 2–3, axillary, oriented horizontally to ascending; peduncles absent; pedicels 10–25 mm and erect to arching in flower, to 40 mm long and erect to arching in fruit, glabrous to sparsely pubescent; calyx 4–5 mm long, 4–5 mm in diameter, campanulate, glabrous to sparsely pubescent, the margin truncate, slightly membranous, wavy or shallowly lobed, with 10 erect to spreading, linear appendages 0.25–3 mm long emerging 0.1–1.5 mm below the calyx rim; fruiting calyx enlarged, campanulate, remaining close to the fruit, 6–8 mm long, 10–13 mm in diameter, the appendages to 5 mm long; corolla 0.7–1.5 cm long, open corolla orientation unknown, stellate in outline, divided ca. 1/3 of the way to the base, with abundant interpetalar tissue, white, additional markings unknown, nearly glabrous to moderately pubescent with short trichomes abaxially near the veins; stamens subequal to unequal, straight, the four short filaments ca. 1 mm long, the long filament 1–2 mm long, glabrous, the anthers 5–5.5 mm long, lanceolate, free of one another, yellow, sometimes sparsely pubescent on inner surface along connective, poricidal at the tips, the pores obovate, dehiscing distally or toward the style, not opening into longitudinal slits; pistil with glabrous ovary, the style 8–10 mm long, linear, straight, glabrous, the stigma ovoid. Fruit a berry, 10–20 mm long, 7–15 mm in diameter, ovoid, orange at maturity, glabrous, lacking sclerotic granules. Seeds 20–30 per fruit, 3.5–4 × 2.5–3 mm, flattened, with slightly thickened rim, depressed ovate in outline, yellow-orange to brown, the surface reticulum with minute, serpentine pattern with shallow luminae.

##### Chromosome number.

Unknown.

##### Distribution and habitat.

**Figure 25.**
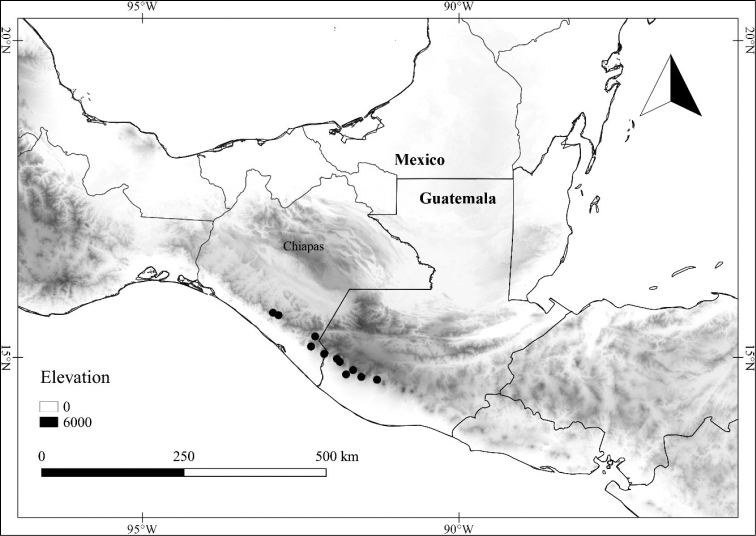
Map of geographic distribution of L. chiapensis var. chiapensis based on herbarium specimen data.

Mexico (Chiapas) and Guatemala (Quetzaltenango, San Marcos, Suchitepéquez), in tropical moist forest or cloud forest, sometimes on sand formations, 1500–2400 m in elevation (Fig. 25).

##### Common names and uses.

None known.

##### Phenology.

Flowering specimens have been collected in June. Specimens with mature fruits have been collected in December and January. The timing of the diurnal movements of the corollas of this species is not known, but the corollas are usually closed on herbarium specimens, indicating that they are probably open for a short time, most likely in the morning.

##### Preliminary conservation status.

Lycianthes chiapensis var. chiapensis is a rarely collected variety of southwestern Guatemala and adjacent Mexico, represented by only 12 collections, three of which are from protected areas. The EOO is 3,020.311 km^2^, and the AOO is 44 km^2^. Based on the IUCN (2019) criteria, the preliminary assessment category is Endangered (EN).

##### Discussion.

Lycianthes chiapensis var. chiapensis is an upper elevation wet forest taxon that is localized in the southern tip of Chiapas and the western region of Guatemala along the Pacific slope. Unlike the more common variety, L. chiapensis var. sparsistellata Standl. & Steyerm., this variety is under-collected, and little is known about its appearance and growth form; it is likely a large liana like var. sparsistellata. The lower sympodial units merge into sinuate woody branches as the plant ages. The mature wood is dark brown and lustrous. This variety differs from var. sparsistellata in having a larger flowering calyx that remains campanulate in fruit and adheres to the fruit as it ages; var. sparsistellata has a much smaller calyx that becomes plate-like in fruit. The fruit of var. chiapensis is also larger, ovoid, and has more seeds. This variety was the first variety to be described, and the type specimens mainly have buds on them, but the larger size of the buds are obvious and different from those of var. sparsistellata. In addition, the leaves of var. chiapensis are somewhat thicker in texture and often more glabrous than those of var. sparsistellata. Although most authors of floras have synonymized the two varieties (for example: Gentry and Standley 1974; Nee 1986), they appear to be very different with regard to calyx morphology and fruit size, and so we are keeping them separate in this treatment. Standley and Steyermark (1940) introduced confusion into the description of the two varieties when describing var. sparsistellata for the first time. The type of var. sparsistellata is clearly that of the small calyx form, but then one of the paratypes cited (*Purpus 7166* from Cerro del Boquerón, Chiapas) is clearly the large calyx form. In describing var. sparsistellata, the authors chose to emphasize pubescence density and ignored the differences in calyx and fruit size.

##### Representative specimens examined.

**Guatemala. Quetzaltenango**: Above Mujuliá, between San Martín Chile Verde and Colomba, 1800 m, 1 Feb 1941, *P.C. Standley 85684* (F). **San Marcos**: near Aldea Fraternidad, between San Rafael Pie de la Cuesta and Palo Gordo, west facing slope of the Sierra Madre Mountains, 1800–2400 m, 10–18 Dec 1963, *L.O. Williams 26281* (NY). **Suchitepéquez**: barranca by Loma Grande, above Finca El Naranjo, on Volcán Santa Clara, 1950–2100 m, 2 Jun 1942, *J.A. Steyermark 46833* (NY). **Mexico. Chiapas**: Reserva de la Biosfera El Triunfo, Poligono III, Cerro Quetzal, 50 km al Sur de la Colonia Independencia, 15.7067, -92.9378, 1856 m, 1 Apr 2001, *G. López-Hernández* (MO).

#### 10b Lycianthes chiapensis (Brandegee) Standl. var. sparsistellata

Taxon classificationPlantae

Standl. & Steyerm., Publ. Field Mus. Nat. Hist., Bot. Ser. 22(4): 274 1940

Fig. 26

##### Type.

Guatemala. Chiquimula: Tixixí (Tishishí), 3–5 miles north of Jocotán, 500–1500 m, 10 Nov 1939, *J.A. Steyermark 31555* (holotype: F [0072902F, acc. # 1039909]; isotype WIS).

**Figure 26.**
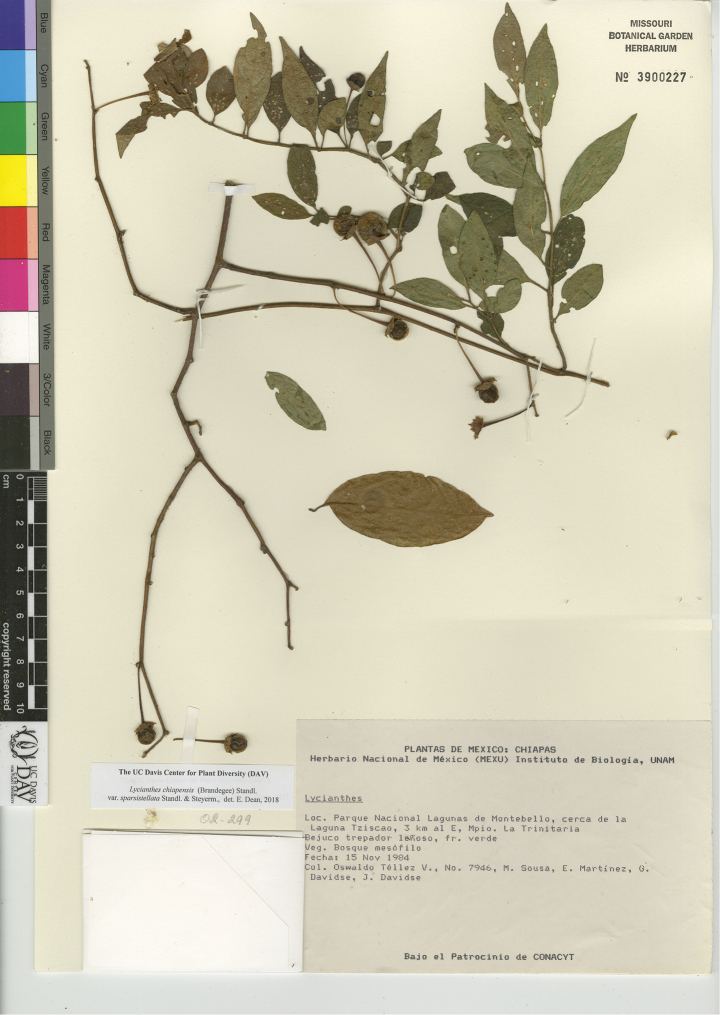
Image of herbarium specimen of L. chiapensis var. sparsistellata, *Téllez 7946* (MO). Specimen used with permission from the Missouri Botanical Garden (http://www.tropicos.org).

##### Description.

Shrub to woody vine, beginning as a shrub, becoming a large liana climbing into tree crowns up to 25 m tall and spreading in the crown up to 10 m wide, the lower stem to 15 cm in diameter. Indument of tannish-yellow to brown, uniseriate, multicellular, stalked, multangulate-stellate, eglandular, spreading trichomes 0.25–1 mm long, 0.75–1.2 mm in diameter, the rays 3–6 per whorl, straight, rarely rebranched. Stems light green when young (drying tan), moderately to densely pubescent, not compressed when dried in a plant press, becoming dark brown and woody with age, the stems sinuous, sometimes glabrate; upper sympodial branching usually monochasial, sometimes dichasial. Leaves simple, the leaves of the upper sympodia sometimes paired and unequal in size, the larger ones with blades 2.5–10 × 1–4 cm, the smaller ones with blades 0.8–4 × 0.5–2 cm, the leaf pairs similar in shape, the blades ovate, elliptic or obovate (sometimes the small geminate leaf nearly orbicular), thin chartaceous to chartaceous, sparsely to moderately pubescent especially along the veins (sometimes nearly glabrous), the base cuneate to rounded, sometimes oblique, the margin entire, usually undulate, the apex acute to acuminate (rarely rounded on smaller leaves), the petiole 0.2–1 cm long, the larger leaf blades with 4–5 primary veins on each side of the midvein. Flowers solitary or in groups of 2–3 (5), axillary, oriented horizontally to ascending; peduncles absent; pedicels (5) 7–24 mm and erect to arching in flower, to 30 mm long and erect to arching in fruit, sparsely to moderately pubescent; calyx (2) 2.5–3.5 mm long, 3–4.5 mm in diameter, campanulate, sparsely to moderately pubescent, the margin truncate, slightly membranous, truncate to wavy or shallowly lobed, with 10 erect to spreading, linear appendages 0.5–2.5 mm long emerging 0.25–0.5 mm below the calyx rim; fruiting calyx enlarged, widely bowl- to plate-shaped, 2–3(4) mm long, 5–8(10) mm in diameter, the appendages not lengthening and often breaking off; corolla 0.6–1.5 cm long, rotate to slightly reflexed in orientation, nearly entire to stellate in outline, divided ca. 1/3–2/3 of the way to the base, with abundant interpetalar tissue, white, the adaxial lobes sometimes with a green spot at the base near the insertion of the shorter stamens, sparsely to moderately pubescent with short trichomes abaxially near the veins; stamens unequal, the four short filaments 0.5–1 mm long, the fifth filament 2.5–4 mm long, glabrous, the anthers 4–5 mm long, lanceolate, free of one another, yellow, pubescent on the inner face, poricidal at the tips, the pores obovate, those of the shorter stamens dehiscing distally or toward the style, those of the long stamen dehiscing toward the style, not opening into longitudinal slits; pistil with glabrous ovary, the style 7–8 mm long, linear, straight to slightly curved, glabrous, the stigma capitate. Fruit a berry, 5–10 (13) mm long, 5–10 (13) mm in diameter, globose, green to white when immature, orange to red at maturity, glabrous to sparsely pubescent, lacking sclerotic granules. Seeds 5–20 per fruit, 3.5–4.5 × 2.5–3.5 mm, flattened, with slightly thickened rim, depressed ovate in outline, yellow-orange to brown, the surface reticulum with minute serpentine pattern and shallow luminae.

##### Chromosome number.

Unknown.

##### Distribution and habitat.

**Figure 27.**
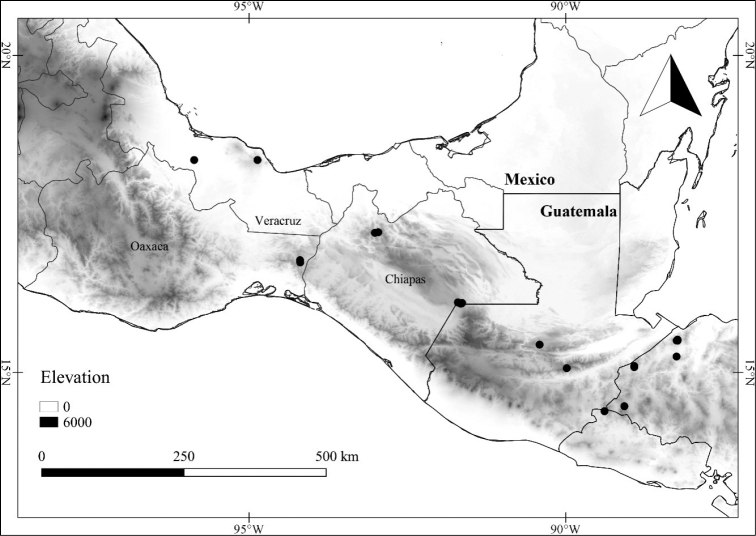
Map of geographic distribution of L. chiapensis var. sparsistellata from Mexico to Honduras based on herbarium specimen data.

Mexico (Chiapas, Oaxaca, and Veracruz), Guatemala (Baja Verapaz, Chiquimula, El Progresso, probably elsewhere), Honduras (Copán, Cortés, Ocotepeque), Nicaragua (Jinotega, Matagalpa), in primary or secondary cloud forest (including oak forest), montane rain forest, and tropical dry forest, (500) 900–2000 m in elevation (Fig. 27).

##### Common names and uses.

None known.

##### Phenology.

In most parts of the range, flowering specimens have been collected July through November (January to March in Nicaragua). Specimens with immature and mature fruits have been collected throughout the year. In the field in Guatemala, the first author observed that the corollas of this species were still open at noon and closed later in the day.

##### Preliminary conservation status.

Lycianthes chiapensis var. sparsistellata is a widespread variety of cloud forest habitat ranging from Mexico to Nicaragua, represented by 23 collections and occurring in five protected areas. The EOO is 156,415.154 km^2^, and the AOO is 84 km^2^. Based on the IUCN (2019) criteria, the preliminary assessment category is Least Concern (LC).

##### Discussion.

Lycianthes chiapensis var. sparsistellata is an upper elevation cloud forest taxon that ranges from Veracruz to Honduras (possibly Nicaragua) mostly along the Caribbean slope. It can grow into a very tall liana that can cover the tree canopy, supported by a very large twining woody stem. The lower sympodial units merge into sinuate woody branches as the plant ages. The mature wood is dark brown and lustrous. This variety is the more common of the two varieties of L. chiapensis and differs from var. chiapensis in having a smaller flowering calyx that becomes plate-like as the plant fruits. The other variety has a larger flowering calyx that adheres to the fruit as it ages and a larger fruit with more seeds. See further discussion of the two varieties under var. chiapensis.

##### Representative specimens examined.

**Guatemala. Alta Verapaz**: at Orchigonia orchid nursery/preserve outside of the city of Cobán along Guatemala Highway 14, 15.4373, -90.4120, 1487 m, 10 Aug 2017, *E. Dean 9507* (DAV). **Chiquimula**: Cerro Tixixí (Tishishí), 3–5 m north of Jocotán, 500–1500 m, 10 Nov 1939, *J.A. Steyermark 31555* (F, WIS). **El Progreso**: Cerro Pinalón, Sierra de las Minas, San Acasaguastlán, 15.0656, 89.9833, 2230 m, 1 Mar 2007, *M. Flores 3548* (MO). **Mexico. Chiapas**: cima del Cerro Salomón, al NO de Benito Juárez, ca. 44 km en línea recta al N de San Pedro Tapantepec, 16.7708, -94.1953, 1770 m, 7 Apr 1986, *M. Ishiki 1451* (NY). **Oaxaca**: Cerro Sabinal, ca. 2 km al SO de Cerro Guayabitos, ca. 3 km en línea recta al NNO de Díaz Ordaz, ca. 40 km en línea recta al N de San Pedro Tapanatepec, al O de la cima del cerro, 16.7333, -94.1917, 1500 m, 21 Dec 1984, *T. Wendt 4678* (NY). **Veracruz**: along trails to base of Volcán Santa Marta, 0–3 km E village of Santa Marta, [18.35, -95.8667], 1100–1200 m, 29 Jun 1982, *M. Nee* 24700 (F, NY, XAL).

#### 11 Lycianthes ciliolata

Taxon classificationPlantae

(M.Martens & Galeotti) Bitter, Abh. Naturwiss. Verein Bremen 24 [preprint]: 410. 1919

Fig. 28

Solanum ciliolatum M.Martens & Galeotti, Bull. Acad. Roy. Sci. Bruxelles 12: 140. 1845. Type: Mexico. Oaxaca: Cordillère orientale de’Oaxaca, dans les bois de la Sierra de Capulalpan et de Llano Verde, Rocheu de Castrasana, de 6,000 à 7,000 pieds, Sep 1840, *H. Galeotti 1230* (lectotype designated by Dean 2004 pg. 399: BR [000000552873]; isolectotypes: BR [000000552906, 000000552842]).
Solanum somniculentum Kunze ex Schltdl., Linnaea 19: 306. 1847. Type: Germany. Grown by G. Kunze in the Leipzig Botanical Garden from seed brought from Mexico by C.A. Ehrenberg, 1845, *G. Kunze s.n.* (lectotype designated by Dean 2004, pg. 399: W [acc. # 0074704]; isolectotype: HAL [acc. # 100603]).
Solanum andrieuxii Dunal, Prodr. [A. P. de Candolle] 13(1): 165. 1852. Type: Mexico. No exact locality (probably from southern Puebla or Oaxaca), 1832?, *G. Andrieux 195* (holotype: G-DC [G00145630]; isotype: M [M0171802, mistakenly listed as the holotype in Dean (2004)]).
Lycianthes ciliolata (M.Martens & Galeotti) Bitter var. pratorum Bitter, Abh. Naturwiss. Verein Bremen 24 [preprint]: 411. 1919. Type: Guatemala. Baja Verapaz: Patal, 1600 m, *H. von Tuerkheim II 2317* (lectotype designated by Dean 2004 pg. 399: W [acc. # 9874]; isolectotypes: BR [000000552875], E [E00570142], F [0072905F, acc. # 574699], G [G00343162], MO [acc. # 3834546], NY [00023650], US [00479453]).
Lycianthes mociniana Dunal var. andrieuxi (Dunal) Bitter, Abh. Naturwiss. Verein Bremen 24 [preprint]: 409. 1919. Type: Based on Solanum andrieuxii.
Lycianthes somniculenta (Kunze ex Schltdl.) Bitter, Abh. Naturwiss. Verein Bremen 24 [preprint]: 411. 1919. Type: Based on Solanum somniculentum Kunze ex Schltdl.
Lycianthes somniculenta (Kunze ex Schltdl.) Bitter var. lanceolata Bitter, Abh. Naturwiss. Verein Bremen 24 [preprint]: 414. 1919. Type: Mexico. Oaxaca: no exact locality, 1842, *Ghiesbrecht 81* (holotype: P [P00070404]).

##### Type.

Based on *Solanum ciliolatum* M.Martens & Galeotti.

**Figure 28.**
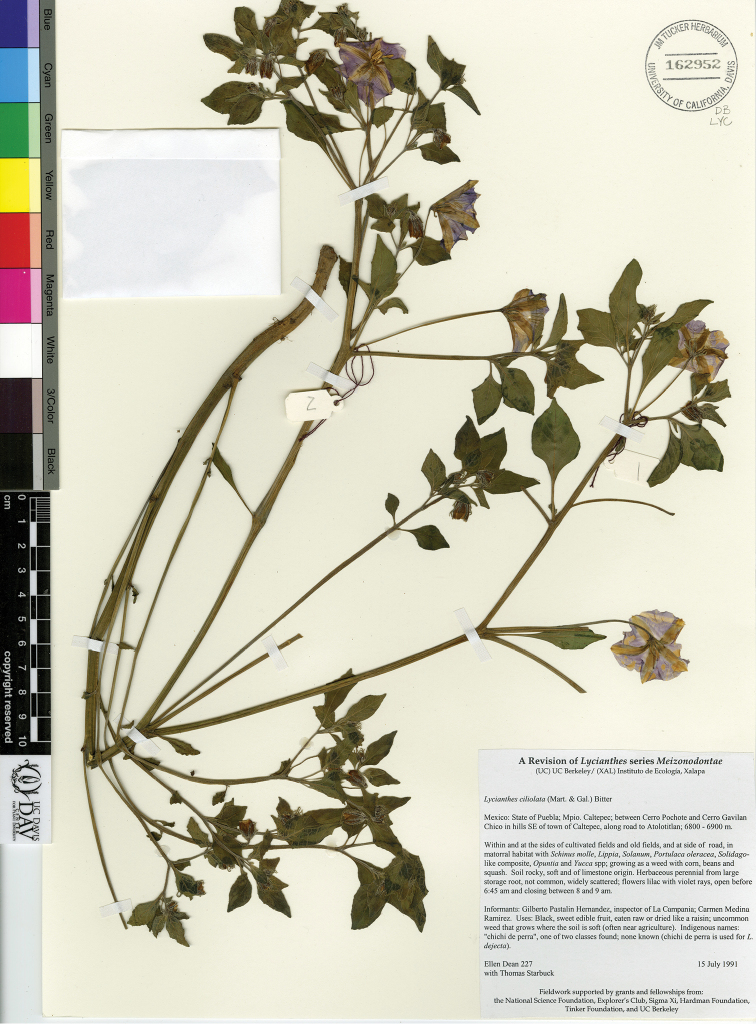
Image of herbarium specimen of *L. ciliolata*, *Dean 227* (DAV). Image used with permission of the UC Davis Center for Plant Diversity.

##### Description.

Perennial herb from fusiform storage roots, usually erect (0.1) 0.2–0.6 (1 m) tall, dying back each season. Indument of white, uniseriate, multicellular, simple or dendritically branched, eglandular, spreading to appressed trichomes 0.1–1 (1.5) mm long. Stems greenish purple, sparsely to moderately pubescent, usually not much compressed upon drying in a plant press, woody with age, especially at the base of the plant; first stem 5–60 cm long to the first inflorescence, the internodes 4–10 (19); first two sympodial branching points dichasial, followed by monochasial branching, this usually extensive. Leaves simple, those of the upper sympodia usually paired and unequal in size, the larger ones with blades 2–14 × 1–7 cm, the smaller ones with blades 1/4 to 3/4 (to equal) the size of the larger, the leaf pairs similar in shape, the blades ovate, lanceolate, or elliptic, (rarely obovate), chartaceous, sparsely to moderately pubescent, the primary veins 4–7 on each side of midvein, the base truncate, obtuse, or cuneate, attenuate onto the petiole, often slightly oblique, the margin entire, usually irregularly undulate, the apex acute to acuminate (rarely long-acuminate), the petiole of larger leaves winged and poorly defined, 0.3–2.5 cm long, sometimes absent. Flowers solitary, axillary, oriented horizontally; peduncles absent; pedicels 30–90 mm and erect in flower, (30) 40.5–80 (110) mm long and deflexed in fruit, sparsely to moderately pubescent with spreading to appressed-retrorse trichomes; calyx 2.5–4.5 mm long, (2.5) 3.5–5.5 (6.5) mm in diameter, obconic to campanulate glabrous to moderately pubescent, the margin truncate, with 10 linear, reflexed appendages 3–9 (11) mm long emerging ca. 0.5–1 mm below calyx rim; fruiting calyx enlarged, (1.5) 2–4 (6) mm long, 5–12.5 (14) mm in diameter, the appendages 3–11 mm long, reflexed, often broken; corolla 1.1–2.7 cm long (2.1–5.3 cm in diameter), rotate in orientation, mostly entire in outline (with shallow notches), with abundant interpetalar tissue, lilac, with maroon to purple stripes along the major veins adaxially, green near the major veins abaxially, usually glabrous; stamens unequal, straight, the filaments of three lengths, the two shortest filaments 1(0.5) 1–3.5 (4.25) mm long, the two medium filaments 1–4.5 (5.5) mm long, the one long filament (2) 3–7 (8) mm long, the length of the long filament nearly always 1.8–3 times that of medium filaments (rarely 1.5–1.8 times), glabrous, the anthers (3) 4–6.25 (8) mm long, lanceolate to oblong, free of one another, yellow, glabrous, poricidal at the tips, the pores linear to ovate, usually dehiscing distally or toward the style, not opening into longitudinal slits; pollen grains dicolporate with remnant third pore; pistil with glabrous ovary, the style 9–12 (13) mm long, linear, straight to slightly curved, glabrous, the stigma round to weakly bilobed (in Oaxaca) to very bilobed (in Guatemala). Fruit a berry, remaining attached to calyx at maturity, pendent, 14–39 mm long, (7) 9–22 mm in diameter, ovoid to conic, the exocarp dark purple to rose-colored, glabrous, the mesocarp rose-colored or dark purple, soft and juicy, lacking sclerotic granules, the placental area light purple and powdery. Seeds (8) 30–80 (102) per fruit, 2.9–4.1 × 2.5–3.9 mm, not compressed, depressed obovate, ridged and blistered along one side, black, the surface reticulum rough in texture with loose serpentine pattern and deep luminae.

##### Chromosome number.

2n = 24 from *Dean 271, 295* (Dean 2004)

##### Distribution and habitat.

**Figure 29.**
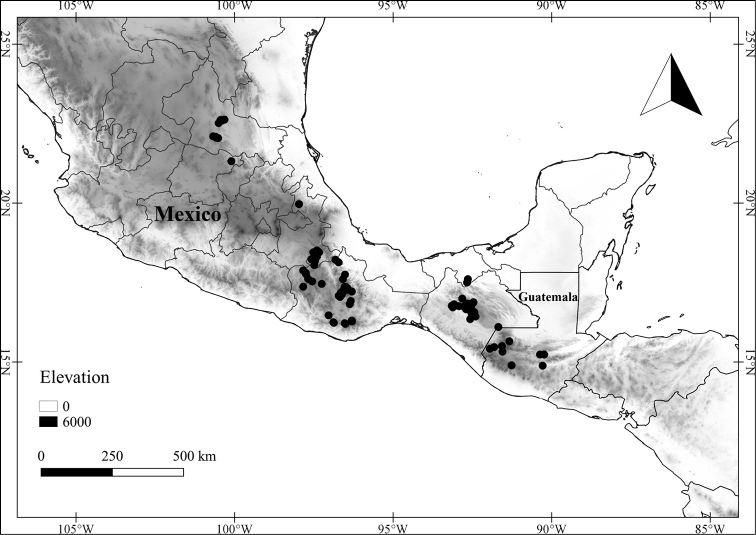
Map of geographic distribution of *L. ciliolata* based on herbarium specimen data.

Mexico (Chiapas, Guanajuato, Oaxaca, Puebla, Querétaro, San Luis Potosí) and Guatemala (Baja Verapaz, Huehuetenango, Totonicapán), in oak or oak/pine forest (that may be intermixed with palms, *Juniperus* or *Yucca*) or in xerophilous scrub in southern Mexico, on slopes, in drainages, in canyons, along paths, and in agricultural fields, on limestone soils, 755–3000 m in elevation (Fig. 29).

##### Phenology.

Flowering specimens have been collected June to October, depending on region. Specimens with mature fruits have been collected between September and November. The first author observed in the field that the corollas are open in the very early morning and closed by late morning. The pollen has a sweet scent. Solitary bees in the genera *Thygator* and *Pseudoaugochloropsis* visit this species (Dean 2001).

##### Common names and uses.

Mexico. Hoh, yich hoh, yichjoj, tintolón, ma’ ‘u’ dsea nuu jgiaa, u dsea niquia, yich balam, guì in-dèm, ngûd-dèm, guizh-dam, kuan xille bekue, campanilla, chichi de vaca, chichi de perro, chichi de venado, binduchi, binduchito, rshtisti katya, tronchichi, tonchicho, manzanillo del campo, shashasto, la pera, chile de ratón. People eat the fruits of this species in the states of Puebla, Oaxaca, and Chiapas. The edible fruits are gathered from wild plants, often from plants growing as volunteers in agricultural fields (Dean 2004).

##### Preliminary conservation status.

*Lycianthes ciliolata* is a widespread species ranging from central Mexico to Guatemala represented by 117 collections and occurring in six protected areas. Anguiano-Constante et al. (2018) provided a preliminary conservation assessment of Least Concern (LC).

##### Discussion.

*Lycianthes ciliolata* is similar to *L. rzedowskii* and *L. acapulcensis*. It differs from those species in having lilac rather than white corollas (or the very pale lilac sometimes found in *L. rzedowskii*). The pattern of filament lengths can also be useful in separating it from *L. rzedowskii*. In *L. ciliolata* the longest stamen filament is often more than twice as long as the medium-short filaments, while in *L. rzedowskii* the longest stamen filament is less than twice as long as the medium-short filaments. The lengths of the pedicels of the youngest mature flowers relative to their subtending leaves is often a useful character for separating *L. ciliolata* from *L. acapulcensis*. In the latter, the length of those pedicels is usually less than that of the subtending leaves, while in *L. ciliolata* the length of the pedicels generally exceeds that of the leaves (Dean 2004).

##### Representative specimens examined.

**Guatemala. Baja Verapaz**: Patal, 1600 m, *H. von Tuerkheim 2317* (W, BR, E, F , G, MO, NY, US). **Huehuetenango**: Cerro Pixpix, above San Ildefonso, Ixtahuacan, [15.4675, -91.8116], 1600–200 m, 15 Aug 1942, *J.A. Steyermark 50597* (NY no #). **Totonicapán**: Cerro María Tecum, Sierra Madre Mountains, 10–20 km east of Totonicapán, [14.8959, -91.2636], 3100–3400 m, 16 Dec 1962, *L.O. Williams 23156* (MO). **Mexico. Chiapas**: Col. Carrizal, 700 m al oriente de la Escuela, 16.6575, -92.6975, 2250 m, 6 Jun 1995, *H. Mejía 429* (XAL). **Guanajuato**: Rancho Beltrán, 10 km al oeste de Xichu, [21.3164, -100.0987], 2000 m, 9 Dec 1990, *E. Ventura 6473* (XAL). **Oaxaca**: Santa María Jaltianguis, [17.361, -96.5282], 7200 ft, 20 Oct 1991, *E. Dean 295* (DAV, IEB, MEXU). **Puebla**: hills to the SW of the city of Tehuacán, up dirt road near El Riego, [18.4433, -97.4235], 1677 m, 27 Sep 1991, *E. Dean 272* (DAV). **San Luis Potosí**: Los Aguajitos, 11 km al NE de Guadalcázar, hacia Pozo de Acuña, 22.625, -100.3167, 2000 m, 17 Nov 1996, *R. Torres C. 14874* (MEXU).

#### 12 Lycianthes connata

Taxon classificationPlantae

J.L.Gentry, Phytologia 26: 271, 1973

Fig. 30

##### Type.

Guatemala. Huehuetenango: Cruz de Limón, between San Mateo Ixtatán and Mucá, Sierra de los Cuchumatanes, 2600–3000 m, 31 Jul 1942, *J.A. Steyermark 49828* (holotype: F [0072906F, acc. # 1199398]).

**Figure 30.**
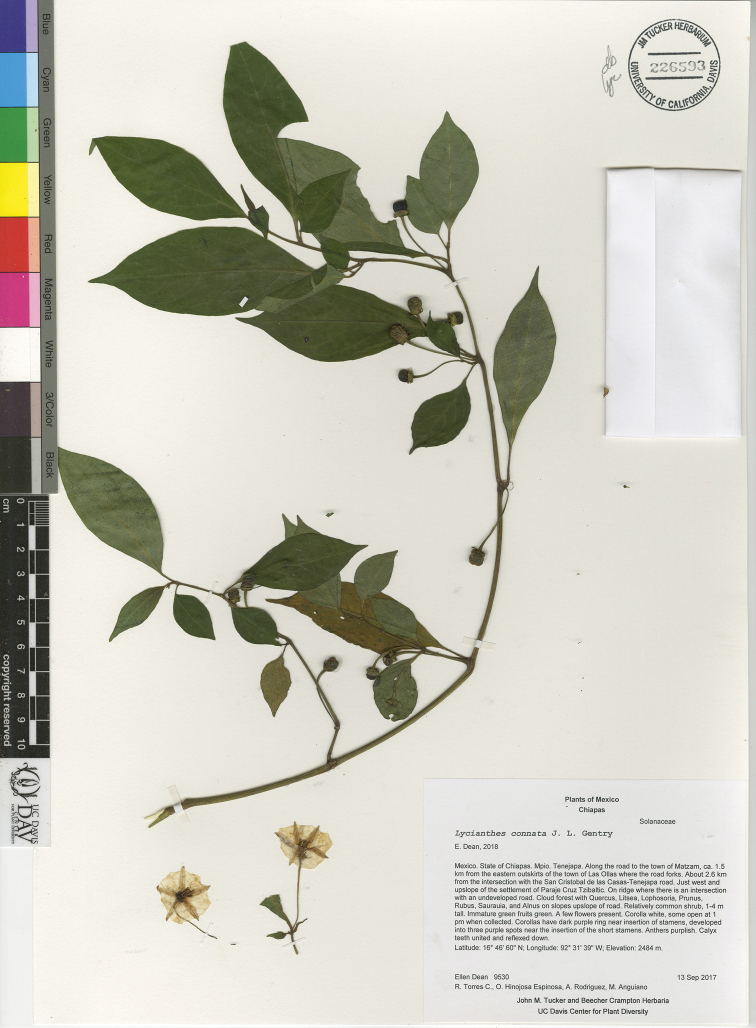
Image of herbarium specimen of *L. connata*, *Dean 9530* (DAV). Image used with permission of the UC Davis Center for Plant Diversity.

##### Description.

Shrub, erect, 0.5–5 (7) m tall. Indument of white, off-white, or brownish, uniseriate, multicellular, simple, curved, eglandular, appressed-ascending trichomes 0.25–1 mm long. Stems green to purplish when young, glabrous to sparsely pubescent, compressed upon drying in a plant press, woody with age; upper sympodial branching points monochasial or dichasial. Leaves simple, the leaves of the upper sympodia usually paired and unequal in size, the larger ones with blades 4.5–16.5 (20) × 1.5–8.5 cm, the smaller ones with blades 1.5–8.5 × 0.5–4 cm, the leaf pairs usually similar in shape, the blades ovate, elliptic, or obovate, membranaceous, glabrous to sparsely pubescent, sometimes with purple veins, the base cuneate to attenuate, sometimes oblique, the margin entire, usually undulate, the apex acute to acuminate, the petiole 0.1–3 cm long, sometimes absent, the larger leaf blades with 4–6 primary veins on each side of the midvein. Flowers solitary or in groups of 2–5, axillary, erect or oriented horizontally; peduncles absent; pedicels 15–35 mm and erect to arching in flower, to 40 mm long, and erect to arching in fruit, usually glabrous; calyx 2–4 mm long, 4–5 mm in diameter, campanulate to widely bowl shaped, sometimes appearing flat-bottomed, usually nearly glabrous, the margin truncate, very well developed, with 10 linear, ascending to reflexed appendages 0.5–4 mm long, connate to each other at the base, emerging 1–3 mm below the calyx rim; fruiting calyx enlarged, widely bowl-shaped, 2–2.5 mm long, 5–7 mm in diameter, the appendages 1.5–3.5 mm long, spreading to reflexed; corolla 0.5–1.7 cm long, rotate to reflexed in orientation, entire to slightly stellate in outline, divided ca. 1/4 of the way to the base, with well-developed interpetalar tissue, white, sometimes with a purple ring in the center adaxially, mostly glabrous; stamens nearly equal to unequal, the four short filaments 1–1.5 mm long, the one long filament 1.5–2.5 mm long, glabrous, the anthers 3.5–5 mm long, lanceolate, free of one another, yellow, glabrous, poricidal at the tips, the pores oval, dehiscing distally, sometimes enlarging by slitting laterally down the side of the anther; pistil with glabrous ovary, the style 6–7 mm long, linear, straight to slightly curved, glabrous, the stigma capitate. Fruit a berry, 5–9 mm long, 5–9 mm in diameter, globose to ovoid, orange at maturity, glabrous, lacking sclerotic granules. Seeds 20–60 per fruit, 1.5–2 × 1.25–1.5 mm, flattened, depressed ovate to reniform in outline, with notch on one side, orange-yellow to orange-brown, the surface reticulum with minute serpentine pattern and shallow luminae.

##### Chromosome number.

Unknown.

##### Distribution and habitat.

**Figure 31.**
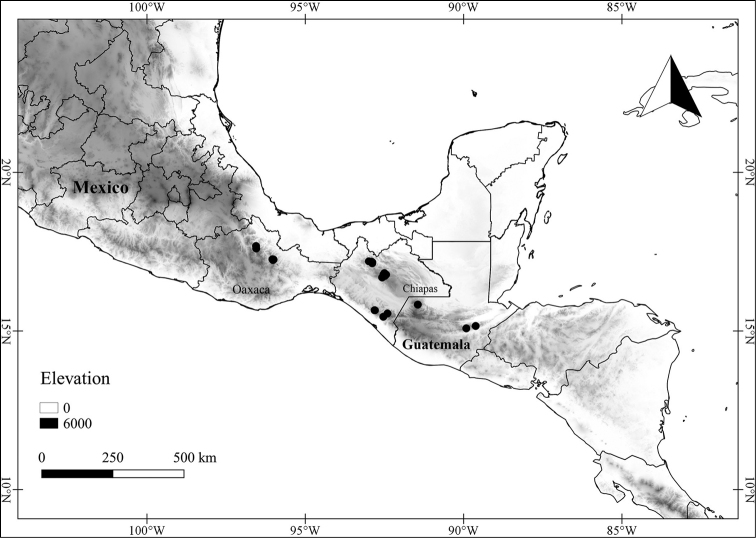
Map of geographic distribution of *L. connata* based on herbarium specimen data.

Mexico (Chiapas, Oaxaca) and Guatemala (El Progreso, Huehuetenango, Zacapa) in cloud forest and montane rain forest, often with *Quercus*, *Pinus*, *Podocarpus*, *Magnolia*, sometimes on slopes, 1600–3000 m in elevation (Fig. 31).

##### Common names and uses.

None known.

##### Phenology.

Flowering specimens have been collected from March through December. Specimens with mature fruits have been collected October through March. The diurnal movements of the corolla are not known, but as some specimens have open corollas, the corollas must stay open at least until late morning.

##### Preliminary conservation status.

*Lycianthes connata* is a cloud forest species of Oaxaca, Chiapas and Guatemala, represented by 27 collections, four of which are from two protected areas. The EOO is 71,681.613 km^2^ and the AOO is 92 km^2^. Based on the IUCN (2019) criteria, the preliminary assessment category is Least Concern (LC).

##### Discussion.

*Lycianthes connata* is a very distinctive species due to the structure of its calyx which has a very long, sleeve-like margin and appendages that are connate at their bases that are reflexed in fruit. The only species that can be confused with *Lycianthes connata* are *L. ceratocalycia* and *L. gongylodes*, both of which have stems that compress upon drying and short, bulging appendages that can give a somewhat similar appearance to the calyx. *Lycianthes ceratocalycia* differs in having stellate, purple corollas, equal stamens, and young stems with scurfy, horizontal lines. *L. gongylodes* differs in having equal stamens and stems that are moderately pubescent with curling trichomes. *Lycianthes connata* was originally described from Guatemala and has been collected often in Chiapas. There are fewer collections from high elevation Oaxaca in the Sierra de Juárez. The Oaxacan populations are very like the Chiapas populations except that the calyx appendages are notably shorter, making the calyx look more like that of *L. gongylodes* or *L. ceratocalycia*.

##### Representative specimens examined.

**Guatemala. El Progreso**: On top of Montaña Piamonte, along Joya Pacayal, 3000 m, 7 Feb 1942, *J.A. Steyermark 43677* (NY). **Huehuetenango**: Cruz de Limón, between San Mateo Ixtatán and Mucá [Nucá], Sierra de los Cuchumatanes, [15.8306, -91.4447], 2600–3000 m, 31 Jul 1942, *J.A. Steyermark 49828* (F). **Zacapa**: Mpio. Río Hondo, 1.5 horas N Finca Alejandra, 30 minutes S Cerro Paloma (P26-Proyecto Deslaves), 15.1569, -89.6178, 2512 m, 17 Mar 2012, *C. Cifuentes 435* (BIGU). **Mexico. Chiapas**: Tzontehuitz. Mpio. Chamula, 16.6856, -92.5714, 2897 m, 28 Aug 1999, *L.Y. Domínguez-Torres 105* (MEXU). **Oaxaca**: Dto. Mixe, Kets tekum, tonun Kux, [17.2523, -96.0292], 17 Jul 1994, *Rivera-Reyes 3156* (IEB, MEXU).

#### 13 Lycianthes cuchumatanensis

Taxon classificationPlantae

J.L.Gentry, Phytologia 26: 273. 1973

Fig. 32

##### Type.

Guatemala: Huehuetenango: between Xoxlac and Nucapuxlac, Sierra de los Cuchumatanes, 1600–2500 m, 17 Jul 1942, *J.A. Steyermark 48925* (holotype: F [0072907F, acc. # 1188543]; isotype: A [00934886]).

**Figure 32.**
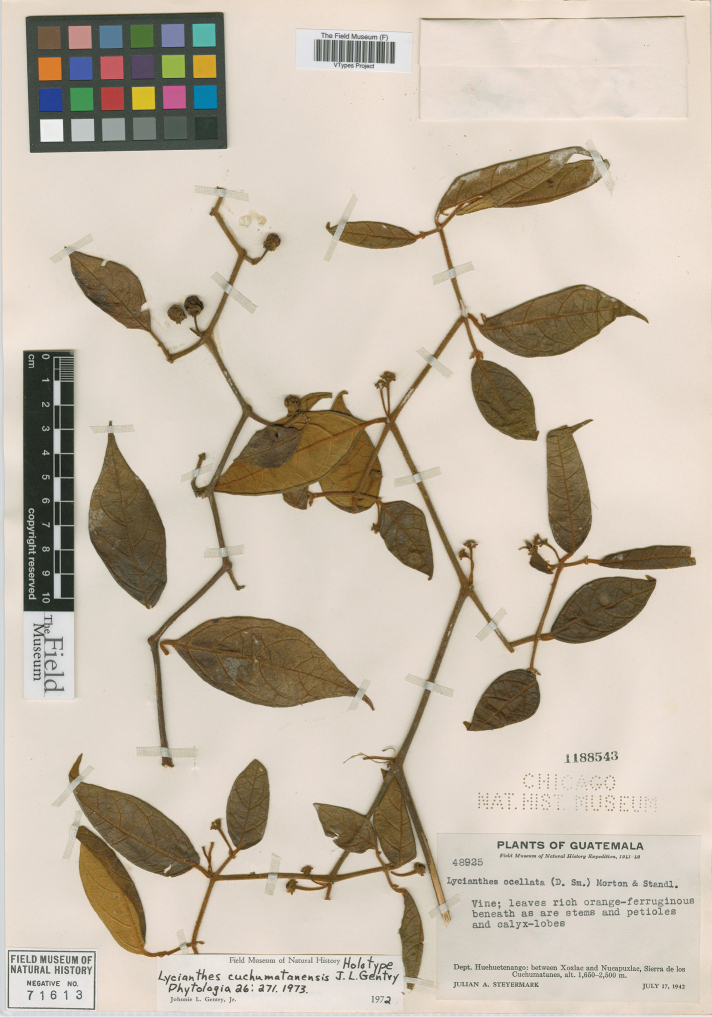
Image of holotype of *L. cuchumatanensis*, *Steyermark 48925* (F). Specimen used with permission from the Field Museum of Natural History.

##### Description.

Scandent shrub to vine, height unknown. Indument of pale yellow to reddish-brown, uniseriate, multicellular, sessile to stalked, multangulate stellate to geminate stellate (multistoried), eglandular, spreading trichomes, 0.25–1 mm long, 0.5–1 mm in diameter, the rays 5–8 per whorl, straight, not rebranched. Stems pale green (drying tan) when young, sparsely to densely pubescent (the surface often obscured), not compressed when dried in a plant press, becoming brown and woody with age; upper sympodial branching points monochasial and dichasial, the branching divaricate (diverging at wide angles). Leaves simple, the leaves of the upper sympodia rarely paired and unequal in size, the blades of the larger ones 5–10 × 2–3.5 cm, the blades of the smaller ones 2–3 × 0.5–1.5 cm, ovate, obovate, lanceolate, or elliptic, subcoriaceous to coriaceous, adaxially sparsely to moderately pubescent (with trichomes concentrated along the veins), abaxially moderately to densely pubescent (with leaf surface sometimes obscured by pubescence), the base cuneate, the margin entire, usually irregularly undulate, the apex acute to acuminate, the petiole 0.5–1.2 cm long, the larger leaf blades with 3–5 primary veins on each side of the midvein. Flowers usually in groups of 2–4, axillary, erect; peduncles absent; pedicels 10–14 mm long and erect in flower, to 15 mm long and erect in fruit, densely pubescent (the surface often obscured); calyx 2.5–3.5 mm long, 2.5–3.5 mm in diameter, campanulate, densely pubescent (the surface usually obscured), the margin truncate, prominent, undulate, scarious (sometimes torn), with 10 obovate, spreading appendages 1–1.5 mm long emerging 1–2 mm below the calyx rim; fruiting calyx slightly enlarged, bowl-shaped, sometimes splitting, 2.5–3 mm long, 4–5 mm in diameter, the appendages to 2 mm long, spreading to reflexing; corolla 0.7–1.1 cm long, open orientation unknown, stellate in outline, divided 1/2 of the way to the base, with scant interpetalar tissue, adaxially purple, glabrous, abaxially densely pubescent on the lobes; stamens equal, straight, the filaments ca. 1 mm long, glabrous, the anthers 3–3.5 mm long, elliptic, free of one another, color uncertain, sparsely pubescent with scattered trichomes, poricidal at the tips, the pores ovate, dehiscing distally, not opening into longitudinal slits; pistil with glabrous ovary, the style 6–7 mm long, linear, straight, glabrous, the stigma capitate. Fruit a berry, ca. 7 mm long, 7 mm in diameter, color unknown, glabrous, lacking sclerotic granules. Seeds number per fruit and seed details unknown, ca. 2.5–3 mm long.

##### Chromosome number.

Unknown.

##### Distribution and habitat.

**Figure 33.**
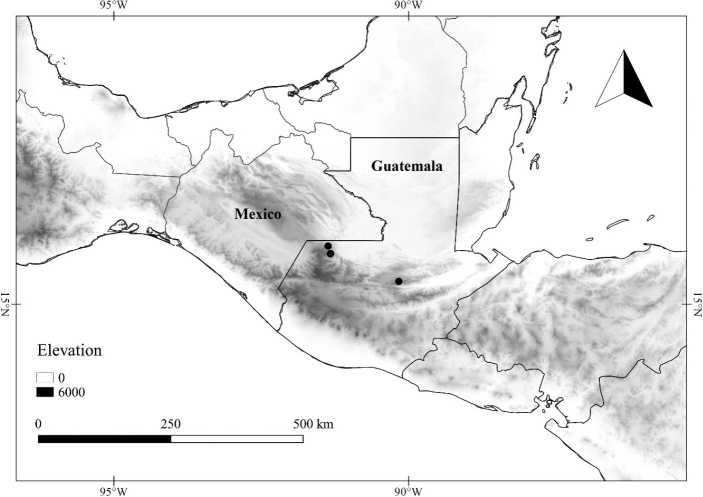
Map of geographic distribution of *L. cuchumatanensis* based on herbarium specimen data.

Guatemala (Alta Verapaz, Huehuetenango), 1500–2600 m in elevation. Nothing is known about the habitat where this species grows, but it may be cloud forest (Fig. 33).

##### Common names and uses.

None known.

##### Phenology.

Flowering specimens have been collected in July and August, and fruiting specimens have been collected in July. Very little is known about this species. The corollas are closed on the few specimens that exist of this species, indicating that the corollas have diurnal movements, but the timing is unknown.

##### Preliminary conservation status.

*Lycianthes cuchumatanensis* is a rarely collected species of Guatemala, represented by only three collections, all outside of protected areas. The EOO is 773.642 km^2^, and the AOO is 12 km^2^. Based on the IUCN (2019) criteria, the preliminary assessment category is Critically Endangered (CR).

##### Discussion.

Although Gentry (1973) thought *Lycianthes cuchumatanensis* to be a close relative of *L. limitanea*, *L. cuchumatanensis* does not resemble *L. limitanea*. It is very similar to *L. sideroxyloides* in its pubescence, branching pattern, solitary leaves, stellate corollas, and equal stamens. It differs from that species in having leaf blades that are usually chartaceous to thick chartaceous, rather than coriaceous, have more rounded bases (rather than cuneate), and have less dense pubescence on the abaxial side. *Lycianthes sideroxyloides* also has a smaller seed size (1.5–2 mm long) than that cited in the protologue for *L. cuchumatanensis* (Gentry 1973). The paratype cited by Gentry in the protologue (*Steyermark 48625*) differs from the holotype in having less dense pubescence and leaf blades that are thinner in texture. Further field work is necessary to locate extant populations at the type locality of *L. cuchumatanensis* to determine if it is conspecific with *L. sideroxyloides*. The name *Lycianthes cuchumatanensis* has been misapplied to *L. breedlovei* and *L. fredyclaudiae* (Dean et al. 2019a).

##### Representative specimens examined.

**Guatemala. Alta Verapaz**: Mpio. San Juan Chamelco, Chicacnab, La Laguna, 15.3844, -90.1639, 2300 m, 4 Aug 1998, *M. Robles 124* (MSB). **Huehuetenango**: Cerro Huitz between Mimanhuitz and Yulhuitz, Sierra de los Cuchumatanes, [15.8550, -91.3244] 1500–2600 m, 14 Jul 1942, *J.A. Steyermark 48617* (G).

#### 14 Lycianthes dejecta

Taxon classificationPlantae

(Fernald) Bitter, Abh. Naturwiss. Verein Bremen 24 [preprint]: 415. 1919

Fig. 34

Solanum dejectum Fernald, Proc. Amer. Acad. of Arts: 35: 569. 1900. Type: Mexico. Durango: near city of Durango, Iron Mountain and vicinity, rare in crevices of rocks, July 1896, *E. Palmer 347* (lectotype designated by Dean 2004 pg. 404: GH [00077482]; isolectotypes: BM [000514923], C, CAS[acc. # 162966], E [E00526483], F [0073089F, acc. # 51446], G [G00343134], K [K000063110], MEXU [MEXU00029058], MO [acc. # 2495231, acc. # 2495232, acc. # 2495233], NY [00214383], S [acc. # S-G-9980], UC [acc. # 104212, acc. # 124634], US [00027543]).
Lycianthes dejecta (Fernald) Bitter var. palmeri Bitter, Abh. Naturwiss. Verein Bremen 24 [preprint]: 416. 1919, nom. illeg. Type: as above, *E. Palmer 347* (holotype: B [not found, cited by Bitter (1919), probably destroyed]; isotypes as listed above).

##### Type.

Based on *Solanum dejectum* Fernald.

**Figure 34.**
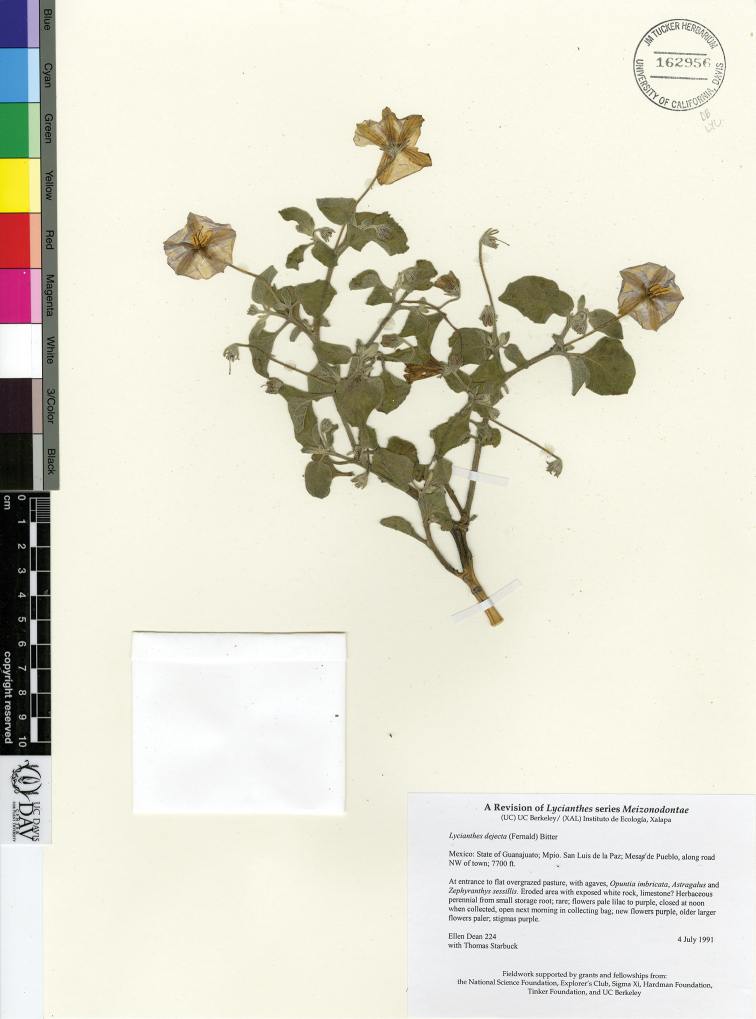
Image of herbarium specimen of *L. dejecta*, *Dean 224* (DAV). Image used with permission of the UC Davis Center for Plant Diversity.

##### Description.

Perennial herb from very large fusiform storage roots, decumbent to erect, decumbent forms to 0.5 m in diameter, tall forms often scrambling, supported by nearby shrubs, to 0.7 m tall, dying back each season. Indument of white, uniseriate, multicellular, simple, dendritically branched, and multangulate-stellate, eglandular, spreading trichomes 0.1–0.5 (0.75) mm long, 0.5–0.75 mm in diameter, the rays of the multangulate trichomes 3–4 per whorl, straight, often rebranched. Stems green with darker green and purple striations, moderately to densely pubescent, much compressed when pressed and dried, becoming woody with age only near the base; first stem 1–35 cm long to the first inflorescence, the internodes 2–7 (13); first sympodial branching point dichasial, followed by a mixture of monochasial and dichasial branching, this branching extensive. Leaves simple, those of the upper sympodia usually paired and unequal in size, the larger ones with blades to 3.5–14.5 × 2–8.5 cm, the smaller ones with blades ca. 1/4–3/4 the size of the larger, the leaf pairs similar in shape, the blades ovate, deltate, or reniform, chartaceous, moderately to densely pubescent, the primary veins 3–5 on each side of the midvein, the base truncate, cuneate, attenuate, decurrent onto the petiole, slightly oblique, the margin entire, usually slightly undulate, the apex acute, the petioles winged and poorly defined, 2–5.5 cm long. Flowers solitary, axillary, oriented horizontally; peduncles absent; pedicels 29–80 (115) mm and erect in flower, (35) 50–110 (125) mm long and deflexed in fruit, moderately to densely pubescent with spreading trichomes; calyx 2.5–4 mm long, 2.5–6 mm in diameter, obconic, the margin truncate, with 10 linear, somewhat reflexed appendages (1) 2–7 (8) mm long emerging ca. 0.5 mm below the calyx rim (usually obscured by trichomes); fruiting calyx enlarged, (5) 6–10 (11.5) mm long, (9) 11–20 (23) mm in diameter, the appendages reflexed to curved, often broken, (3.5) 4–15 (19) mm long; corolla 1–2.2 cm long (1.9–4.1 cm in diameter), rotate in orientation, mostly entire in outline (with shallow notches), with abundant interpetalar tissue, white to lilac, with maroon to purple stripes along the major veins adaxially, green, white, or purple and densely pubescent near the major veins abaxially; stamens unequal, straight to curved, the filaments of three lengths, the two shortest (1.25) 1.5–4 mm long, the two medium filaments (1.5) 2–5 mm long, the one long filament (2) 3–7.5 mm long, the length of the long filament 1.1–1.8 (2) times that of the medium filaments, glabrous; anthers 3–6 mm, ovate to lanceolate, free of one another, yellow, glabrous, poricidal at the tips, the pores ovate, dehiscing distally, not opening into longitudinal slits; pollen grains tricolporate; pistil with glabrous ovary, the style (6) 7–11.5 mm, linear, straight to curved downward, the stigma lobed. Fruit a berry, remaining attached to calyx at maturity, pendent, sometimes near the ground, 17–39 mm long, 12–24 mm in diameter, ovoid to conic, the exocarp light to dark green with purple or black lines (becoming yellowish or brown in age), the mesocarp white to green and juicy, lacking sclerotic granules, the placental area narrow, greenish-white, juicy. Seeds (40) 50–170 (185) per fruit, 2.2–2.8 × 1.5–2.5 mm, rounded, slightly compressed, reniform to depressed-obovate, dark brown to black, surface reticulum with loose serpentine pattern with deep luminae and microscopic fibrils protruding from the cell walls.

##### Chromosome number.

2n = 24, *Dean 276, 309* (Dean 2004)

##### Distribution and habitat.

**Figure 35.**
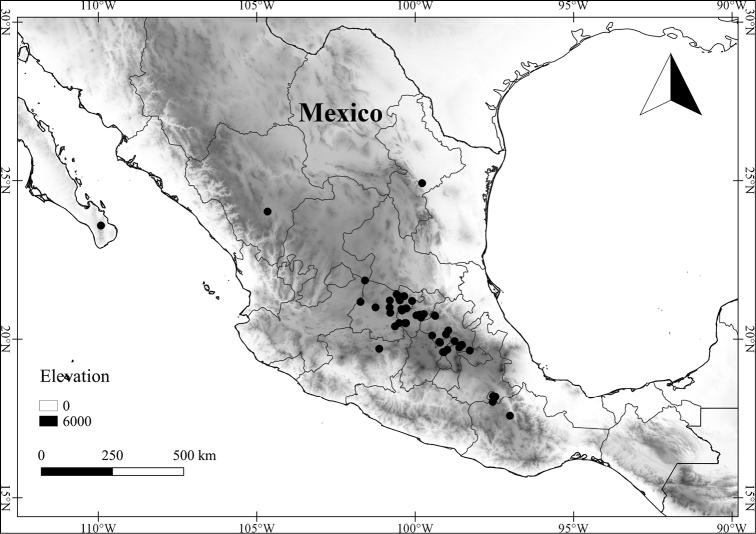
Map of geographic distribution of *L. dejecta* based on herbarium specimen data.

Mexico (Baja California Sur, Distrito Federal, Durango, Guanajuato, Hidalgo, Jalisco, México, Michoacán, Nuevo León, Oaxaca, Puebla, Querétaro) on limestone on either side of the transvolcanic belt, as well as in eroded, ancient agricultural areas within the transvolcanic belt (rarely on rhyolite), usually in xerophilous shrub; it has also been found in disturbed relictual tropical dry forest or oak forest. It has been suggested that eroded volcanic areas within the Valley of Mexico are often home to calciphiles, because erosion has exposed a lower soil layer that is calcium rich (Rzedowski 1986). Habitats include pastures, paths, the sides of agricultural fields, and within abandoned fields at 1800–2900 m in elevation (Fig. 35).

##### Common names and uses.

Mexico. Trompeta, chichi de perra (Dean 2004); used to treat stomachache in Guanajuato (*Ocampo 47*).

##### Phenology.

Flowering specimens have been collected June to August; specimens with mature fruits have been collected September to October. The first author observed in the field that the corollas open after sunrise and close by early afternoon. The pollen in this species has a sweet scent. Solitary bees in the genus *Colletes* visit this species (Dean 2001).

##### Preliminary conservation status.

*Lycianthes dejecta* is a widespread Mexican endemic, represented by 55 collections and occurring in three protected areas (Sierra la Laguna, Sierra Gorda and Tehuacán-Cuicatlan Valley). Anguiano-Constante et al. (2018) provided a preliminary conservation assessment of Least Concern (LC).

##### Discussion.

*Lycianthes dejecta* is a perennial herb recognized by its dense, dendritic to multangulate-stellate trichomes, which cover all parts of the plant, and the truncate bases of its leaf lamina. Its fruits and seed type are similar to those of *L. moziniana*. It differs from that species in having maroon to black lines on its fruits, reflexed to curled calyx teeth, and microscopic fibrils on its seeds. All parts of this plant, including the fruits, have a bitter taste (Dean 2004).

##### Representative specimens examined.

**Mexico. Baja California Sur**: Mpio. La Paz. El Paraje de Cano, Sierra de la Victoria, 23.5833, -109.9167, 1670 m, 30 Sep 1994, *M. Domínquez L. 800* (HCIB). **Distrito Federal**: Sierra de Guadalupe, [19.5908, -99.1203], 7000 ft, *Balls 5073* (BM, K, UC). **Durango**: [24.0237, -104.6580], Apr to Nov 1896, *E. Palmer 347* (BM, C, CAS, G, UC, F, US, MO, NY). **Guanajuato**: cañada a 5 km de Santa Anita, cerro La Meza, [20.9667, -100.3], 2300 m, 22 Sep 2002, *Castillejos-Cruz 1229* (MEXU). **Hidalgo**: Cañon de las Ajuntas, Santa María Macua, 20.1125, -99.4625, 2150 m, 15 Jun 2003, *L. Romero 75* (MEXU). **Jalisco**: carretera Lagos de Moreno-Leon, km 31, [21.1739, -101.7238], 2020 m, 15 Jul 1991, *H. Arreola-Navas 1270a* (MEXU). **México**: N of Huehuetoca along the road to Apaxco, c. 4.2 road mi from building “los arcos” (in dowtown Huehuetoca), W side of rd, [19.8894, -99.2141], 7100 ft, 3 Aug 1991, *E. Dean 243* (DAV). **Michoacán**: Vic. Morelia, Punguato, [19.6954, -101.1381], 2100 m, 20 June 1912, *Arsène 8300* (F, GH, MO, NY). **Nuevo León**: Cerro El Gallo, [24.92, -99.78], 2085 m, 15 Jun 1991, *G. Hinton 21019* (GH, IEB, TEX). **Oaxaca**: a las afueras de Guadalupe Membrillos, 18.0228, -97.5508, 2276 m, 12 Aug 2004, *O. Téllez-V. 17009* (MEXU). **Puebla**: Mpio. Caltepec, between Cerro Pochote and Cerro Gavilán Chico in hills SE of town of Caltepec, along road to Atolotitlan, near small valley called La Laguna, [18.1784, -97.4698], 6800–6900 ft, *E. Dean 228* (DAV). **Querétaro**: 2.09 km al NO de Bernal, Ezequiel Montes, 20.7508, -99.9572, 2240 m, 21 Sep 2012, *O. Rubio-García 263* (IEB).

#### 15 Lycianthes fredyclaudiae

Taxon classificationPlantae

E.Dean, Phytotaxa 409: 268. 1919

Fig. 36

##### Type.

Guatemala. Baja Verapaz: Niño Perdido, Cerro Verde, east of km 150 of Cobán Road, in high forest, elevation not recorded, 3 Dec 1976, *C.L Lundell 20419* (holotype: LL 00490012; isotypes: F [acc. # 1912542], MO [acc. # 3342033], LL [00490006]).

**Figure 36.**
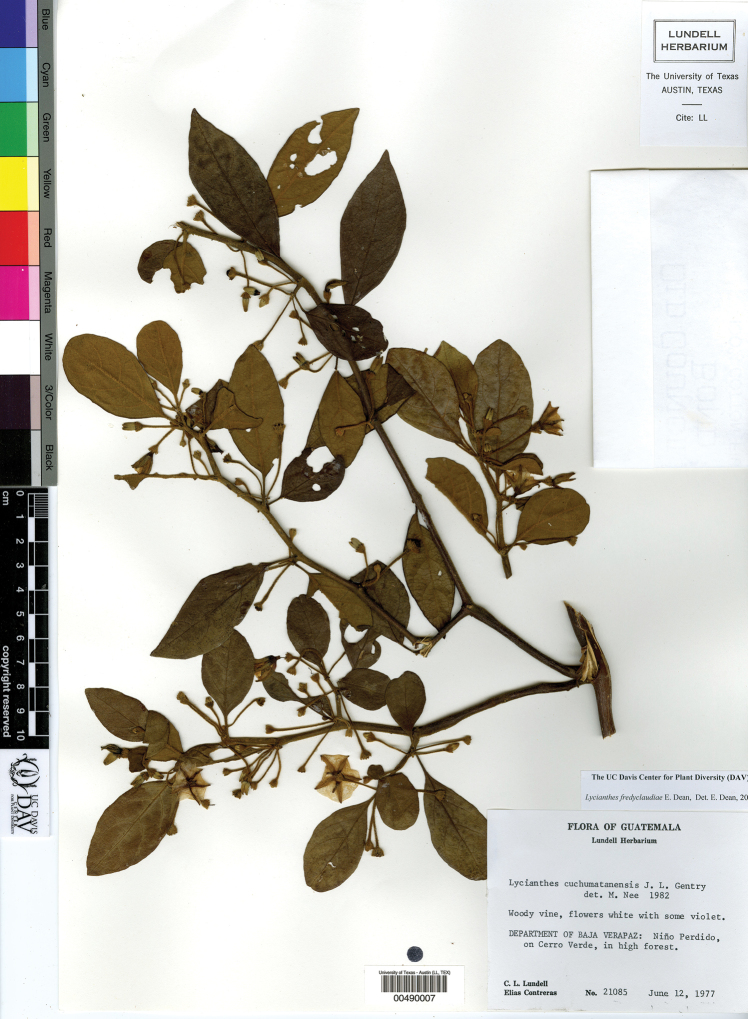
Image of herbarium specimen of *L. fredyclaudiae*, *Lundell 21085* (LL). Specimen used with permission from the Lundell Herbarium, University of Texas at Austin.

##### Description.

Scandent shrub to weak vine, sometimes epiphytic, 2–3 m tall. Indument of tan, pale yellow, or orange, uniseriate, multicellular, stalked, multangulate-stellate, eglandular, spreading trichomes 0.25–1 mm long, 0.75–1.2 mm in diameter, the rays 3–5 per whorl, straight, often rebranched. Stems tan-green to purple-green when young, moderately to densely pubescent, not compressed when dried in a plant press, becoming dark brown and woody with age, the surface of the stems shiny and somewhat longitudinally wrinkled upon drying; upper sympodial branching points mostly monochasial, with some dichasial branching point, the branching not widely divaricate, the adjoining branches often forming straight continuous axes. Leaves simple, the leaves of the upper sympodia usually paired and unequal in size, the larger ones with blades 3–8.5 × 1.5–4 cm, the smaller ones with blades 1–3.5 × 0.5–2.5 cm, the leaf pairs similar in shape, the blades ovate, elliptic, or obovate, the smaller leaf sometimes nearly round, coriaceous, sparsely to densely pubescent, sometimes nearly glabrous adaxially, the trichomes usually dense on the abaxial side, especially along the veins, the base cuneate to rounded, sometimes oblique, the margin entire, usually irregularly undulate, the apex obtuse, acute, or short-acuminate, the petiole 0.2–1.4 cm long, sometimes absent, the larger leaf blades with 3–5 primary veins on each side of the midvein. Flowers in groups of 2–8, axillary, oriented horizontally; peduncles absent; pedicels 8–25 mm long and erect in flower, to 31 mm and erect in fruit, moderately to densely pubescent; calyx 2–3.5 mm long, 3–4.5 mm in diameter, campanulate, moderately to densely pubescent, the margin truncate, with 10 spreading linear appendages 0.5–2 mm long emerging 0.25–0.5 mm below the calyx rim; fruiting calyx enlarged, widely bowl-shaped to rotate, 2–3 mm long, 5–8 mm in diameter, the appendages not elongating; corolla 0.7–1.7 cm long, rotate in orientation, entire in outline, with abundant interpetalar tissue, adaxially white to pale lilac, very sparsely puberulent, abaxially white to pale lilac, the lobes sometimes greenish, moderately to densely puberulent; stamens unequal, straight, the four short filaments 0.5–2 mm long, the one long filament 2–4 mm long, glabrous, the anthers 4–5 mm long, lanceolate, free of one another, yellow to purple, usually with small, white trichomes scattered on either the face of the anther or on the two lobes at the very bottom of the anther, poricidal at the tips, the pores ovate, dehiscing distally, not opening into longitudinal slits; pistil with glabrous ovary, the style 7–8 mm long, linear, straight, glabrous, the stigma oblong, decurrent down two sides. Fruit a berry, 5–13 mm long, 4–12 mm diameter, globose to depressed globose, green to white when immature, yellow to orange when mature, sometimes with a few scattered trichomes, lacking sclerotic granules. Seeds 10–40 per fruit, 2.5–4 × 2–3.5 mm, flattened, thickened on the edges, circular to depressed ovate in outline, sometimes reniform with small notch on one side, yellow-orange to orange-brown, the surface reticulum with loose serpentine pattern and deep luminae, the margin rougher in texture than the center

##### Chromosome number.

Unknown.

##### Distribution and habitat.

**Figure 37.**
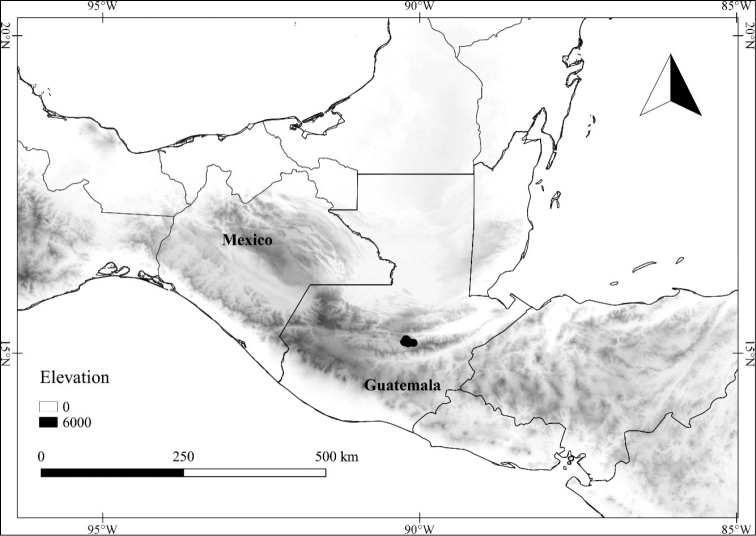
Map of geographic distribution of *L. fredyclaudiae* based on herbarium specimen data.

Guatemala (Baja Verapaz), in cloud forest, “tall forest,” wet forest thickets, and forest floors, sometimes along drainages or on slopes, prefers undisturbed forest, 1500–1800 m in elevation (Fig. 37).

##### Common names and uses.

None known.

##### Phenology.

Flowering specimens have been collected all months of the year except September to November and January to February; specimens with mature fruits have been collected all months of the year except April and May. In the field, the first author observed that the corollas were open in the morning and closed in the afternoon.

##### Preliminary conservation status.

*Lycianthes fredyclaudiae* is a Guatemalan cloud forest endemic, represented by 13 collections from a relatively small area, with six collections from protected areas (Mario Dary Rivera and Sierra de Minas). Dean et al. (2019a) provided a preliminary conservation assessment of Endangered (EN) for this species.

##### Discussion.

*Lycianthes fredyclaudiae* is a species of cloud forest that is known only from the area south of Cobán in the Department of Baja Verapaz. In the 1970s, this species was often collected in primary forest in the vicinity of Union Barrios (collections include those made by the fourth author of this paper), but when this area was revisited in 2017, most of the forest had been cleared, and it was difficult to find the species in the remnants along the highway. One plant was located growing in a roadside thicket, after two days of searching the area, including two reserves. Therefore, it is assumed that the species prefers more undisturbed, lower light habitats, and the species may now be a rare plant (Dean et al. 2019a). *Lycianthes fredyclaudiae* has been misidentified as *L. cuchumatanensis*, a species related to *L. sideroxyloides*. It differs from that species in having rotate, mostly entire corollas (vs stellate corollas), and unequal stamens (vs equal). It is most similar to *L. chiapensis*, *L. breedlovei*, and *L. hortulana*. *Lycianthes chiapensis* differs in being a large vine climbing high into the canopy, having thinner leaves, and having trichomes with rays that are mostly unbranched. *Lycianthes breedlovei* and *L. hortulana* differ from *L. fredyclaudiae* in having stellate corollas and divaricate branching (Dean et al. 2019a).

##### Representative specimen examined.

**Guatemala. Baja Verapaz**: along Guatemala Highway 14 just south of highway marker 158 and La Ram Tzul nature reserve, W side of the road, 15.2064, -90.2065, 1610 m, 11 Aug 2017, *E. Dean 9508* (DAV226622).

#### 16 Lycianthes geminiflora

Taxon classificationPlantae

(M.Martens & Galeotti) Bitter, Abh. Naturwiss. Verein Bremen 24 [preprint]: 497. 1919

Fig. 38

Solanum geminiflorum M.Martens & Galeotti, Bull. Acad. Brux. 12 (1): 142. 1845. Type: Mexico. Oaxaca: Chinantla (Toavela), 3000 ft, *H. Galeotti 1242* (lectotype designated by Dean and Reyes 2018a, pg. 42: BR [000000552845]; isolectotypes: BR [000000552904], LE [LE00016926]).

##### Type.

Based on *Solanum geminiflorum* M.Martens & Galeotti.

**Figure 38.**
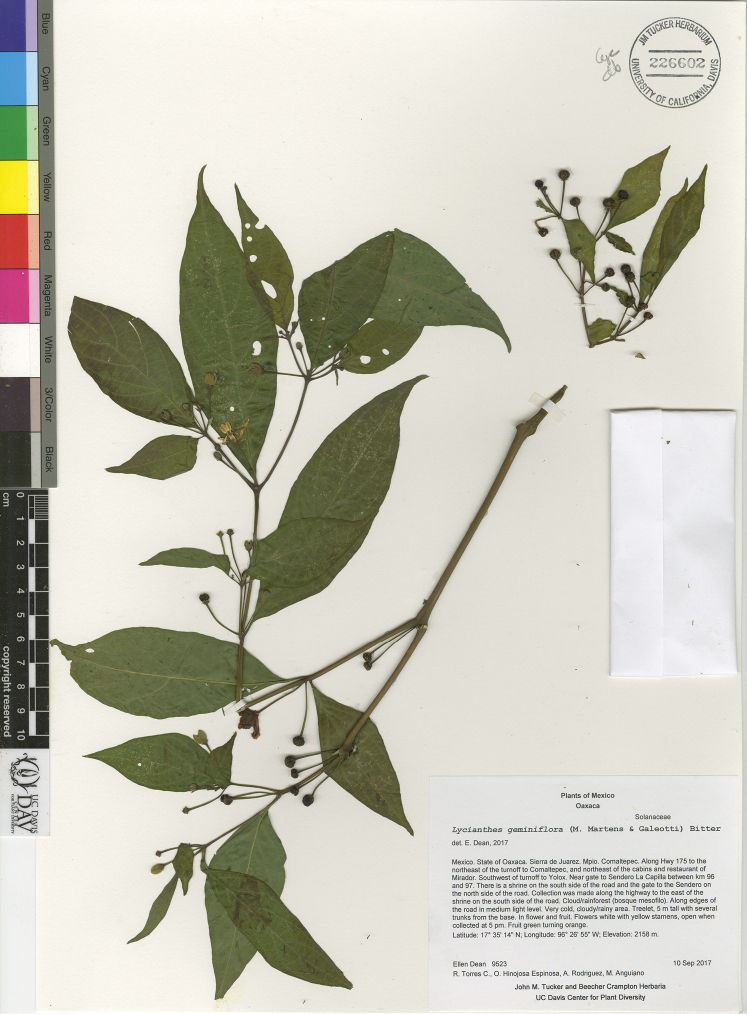
Image of herbarium specimen of *L. geminiflora*, *Dean 9523* (DAV). Image used with permission of the UC Davis Center for Plant Diversity.

##### Description.

Herb, shrub, to treelet, erect, 0.5–5 m tall. Indument of very small, off-white to tan, uniseriate, multicellular, simple, eglandular, appressed-ascending trichomes < 0.1 (0.2) mm long. Stems green when young, sparsely to moderately pubescent, compressed upon drying in a plant press, woody with age; upper sympodial branching points monochasial or dichasial. Leaves simple, the leaves of the upper sympodia usually paired and unequal in size, the larger ones with blades 5–15 × 1.5–6 cm, the smaller ones with blades 2–6 × 0.5–3 cm, the leaf pairs usually similar in shape, the blades ovate, elliptic, or obovate, thin-membranaceous, glabrous to very sparsely pubescent, the base cuneate to attenuate, often oblique, the margin entire, usually undulate, the apex acute to acuminate, the petiole 0.2–0.5 (0.7) cm long, the larger leaf blades with 5–6 primary veins on each side of the midvein. Flowers solitary or in groups of 2–8, axillary, erect; peduncles absent; pedicels 8–20 mm and erect in flower, to 25 mm long and erect in fruit, glabrous to sparsely pubescent; calyx 1–1.5 mm long, 2.5–3 mm in diameter, widely campanulate, puberulent with very small trichomes, the margin truncate, undulate, the appendages lacking; fruiting calyx not very enlarged, widely bowl-shaped, 0.5–1.5 mm long, 3–4 mm in diameter; corolla 0.6–1.2 cm long, campanulate to reflexed in orientation, stellate in outline, divided nearly to the base, interpetalar tissue mostly lacking, white and glabrous adaxially, yellow-green to green and puberulent abaxially; stamens equal, straight, the filaments ca. 1 mm long, glabrous, the anthers 3–3.5 mm long, elliptic, usually partially connivent to the adjacent anther (at least near the middle or base of the anther, not at the tips), yellow, glabrous, poricidal at the tips, the pores round, dehiscing distally, not opening into longitudinal slits; pistil with glabrous ovary, the style 6–8 mm long, linear, straight to curved, glabrous; stigma capitate, decurrent down two sides. Fruit a berry, 4–7 mm long, 4–10 mm in diameter, globose to depressed globose, orange-red at maturity, glabrous, lacking sclerotic granules. Seeds 20–70 per fruit, 1.25–1.75 × 1–1.25 mm, flattened, depressed ovate in outline, tan to orange, the surface reticulum with pitted serpentine pattern with deep luminae.

##### Chromosome number.

Unknown.

##### Distribution and habitat.

**Figure 39.**
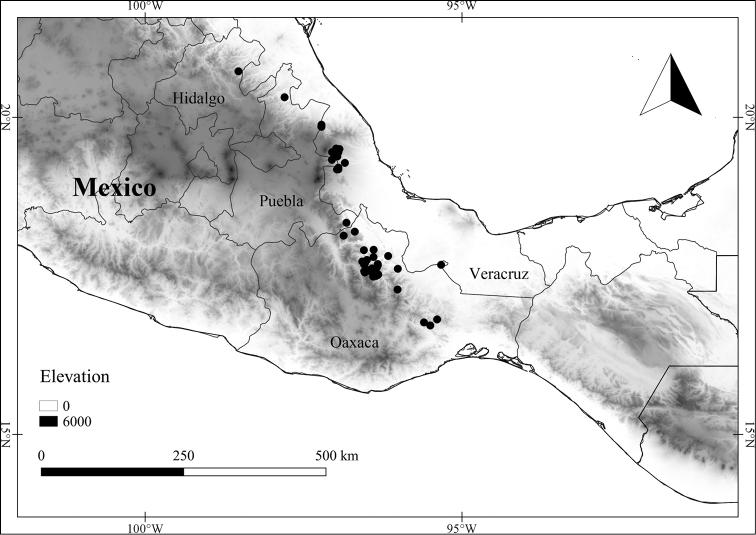
Map of geographic distribution of *L. geminiflora* based on herbarium specimen data.

Mexico (Hidalgo, Oaxaca, Puebla, Veracruz) in cloud forest, tropical dry forest, oak and oak-pine forest, sometimes in disturbed habitats, secondary forest, slopes, coffee plantations, 800–2200 (3000) m in elevation (Fig. 39).

##### Common names and uses.

Mexico. Oaxaca: leaves used as an edible green (*Boyle 2631*); ndia-sku-ya (Mazatec) (*Giovannini 236a*). Puebla: plant used as an edible green;, ndazkjuyoo (Mazateco) and cuajquilitl (Nahuatl) (*C. Mota Cruz 652*). Veracruz: hierba mora (*Dorantes 100*).

##### Phenology.

Flowering and fruits specimens have been collected most months of the year. Based on field observations by the first author, the corollas of this species are open for much of the day in some locations.

##### Preliminary conservation status.

*Lycianthes geminiflora* is a species of threatened cloud forests of central and southern Mexico, represented by 55 collections, all collected outside of protected areas. The EOO is 39,209.944 km^2^, and the AOO is 200 km^2^. Based on the IUCN (2019) criteria, the preliminary assessment category is Least Concern (LC).

##### Discussion.

*Lycianthes geminiflora* is a large herb to treelet, usually found in cloud forest on slopes. It is very similar to *L. heteroclita* (Sendtn.) Bitter, with which it can overlap in distribution in Mexico. The two species differ in the size of the flowering calyx, with that of *L. geminiflora* 1.5 mm long or less, and that of *L. heteroclita* usually 2 mm long or more.

##### Representative specimens examined.

**Mexico. Hidalgo**: Mpio. Tianguistengo, 10 km al E de Tianguistengo, [20.7265, -98.5279], 6 Jul 1995, *M. Sousa Peña 594* (IEB, MEXU). **Oaxaca**: Sierra de Juárez, Mpio. Comaltepec, along Hwy 175 to the NE of the turnoff to Comaltepec, and NE of the cabins and restaurant of Mirador, trail on the southeast side of the road called Sendero Relampago, 17.5918, -96.3999, 2080 m, 10 Sep 2017, *E. Dean 9521* (DAV). **Puebla**: La Guacamaya, [18.3375, -96.8230], 1100 m, Oct 2006, *C. Mota-Cruz 652* (XAL). **Veracruz**: Rancho El Riscal, 19.4544, -96.9964, 2180 m, 26 Sep 2007, *J. Hernández-M. 24* (XAL).

#### 17 Lycianthes glabripetala

Taxon classificationPlantae

E.Dean, Phytologia 100: 28, 2018

Fig. 40

##### Type.

Mexico. Querétaro: Mpio. Landa, 10 km al noreste de Agua Zarca, sobre camino a Neblinas, 1100 m, 23 Jun 1988, *J. Rzedowski 46837* (holotype: DAV [acc. # 217731]; isotypes: IEB [acc. # 193504], TEX [00449100]).

**Figure 40.**
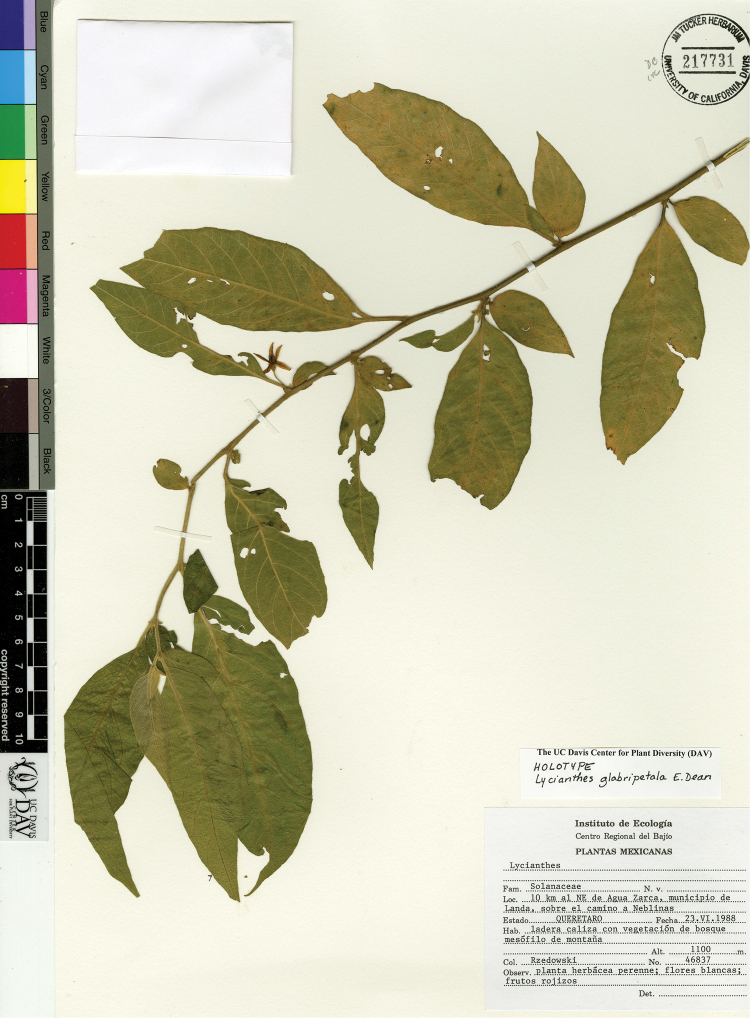
Image of holotype of *L. glabripetala*, *Rzedowski 46837* (DAV). Image used with permission of the UC Davis Center for Plant Diversity.

##### Description.

Perennial herb to small shrub, 0.5–2 m tall. Indument of off-white to tan, uniseriate, multicellular, simple, acute, curved to crisped, eglandular, appressed-ascending (rarely spreading) trichomes, 0.25–1.25 mm long. Stems green when young, moderately to densely pubescent, somewhat compressed upon drying in a plant press, light brown and woody with age; upper sympodial branching points usually monochasial with a few dichasial branching points. Leaves simple, the leaves of the upper sympodia usually paired and unequal in size, the larger ones with blades (4.5) 8.5–14 × (1.8) 2.5–5 cm, ovate to elliptic, the smaller ones with blades 1.3–4.5 × 0.8–2.1 cm, usually ovate, the blades of both the large and small leaves chartaceous, moderately to densely pubescent, the pubescence densest along the veins of the abaxial side, the trichomes along the midvein of the abaxial side appressed and appearing woolly, the base cuneate, usually oblique (sometimes rounded in the smaller leaves), the margin entire, usually delicately undulate, the apex acute to acuminate, the petiole 0.1–1.5 cm long, sometimes absent, the large leaf blades with (6) 8–11 primary veins on each side of the midvein. Flowers often solitary, sometimes in groups of 2–3, axillary, oriented horizontally to nodding; peduncles absent; pedicels 9–15 mm long and arching in flower, 12–20 mm long and arching in fruit, moderately to densely pubescent; calyx 2–2.5 mm long, 2.5–3 mm in diameter, obconic to narrowly campanulate, moderately pubescent, the margin truncate to undulate, with 5–10 narrow, linear, spreading appendages 0.5–2 mm long emerging 0.25–0.5 mm below the calyx rim; fruiting calyx slightly enlarged, widely bowl-shaped to plate-shaped, 1–2 mm long, 4–6 mm in diameter, the appendages withering in age; corolla 1–1.2 cm long, campanulate to reflexed in orientation, stellate in outline, divided nearly to the base, interpetalar tissue present near base, white, adaxial markings unknown, sparsely pubescent on abaxial surface along the midvein; stamens equal, straight, the filaments 0.75–1 mm long, glabrous, the anthers ca. 3 mm long, lanceolate, somewhat narrowed at the tip (the narrowed portion ca. 0.25 mm long), free of one another, color unknown, glabrous, poricidal at the tip, the pores ovate, dehiscing distally, not opening into longitudinal slits; pistil with glabrous ovary, the style ca. 8 mm long, linear, glabrous, widened distally into the stigma; stigma capitate, decurrent down two sides. Fruit a berry, 3.2–8 mm long, 3.1–7 mm in diameter, globose, orange at maturity, glabrous, lacking sclerotic granules. Seeds 30–60 per fruit, 1–1.2 × 0.5–1 mm, compressed but not flat, sometimes with one shallow ridge, semi-circular, depressed ovate, triangular, or rhombic in outline, orange, the surface reticulum with serpentine pattern and shallow luminae.

##### Chromosome number.

Unknown.

##### Distribution and habitat.

**Figure 41.**
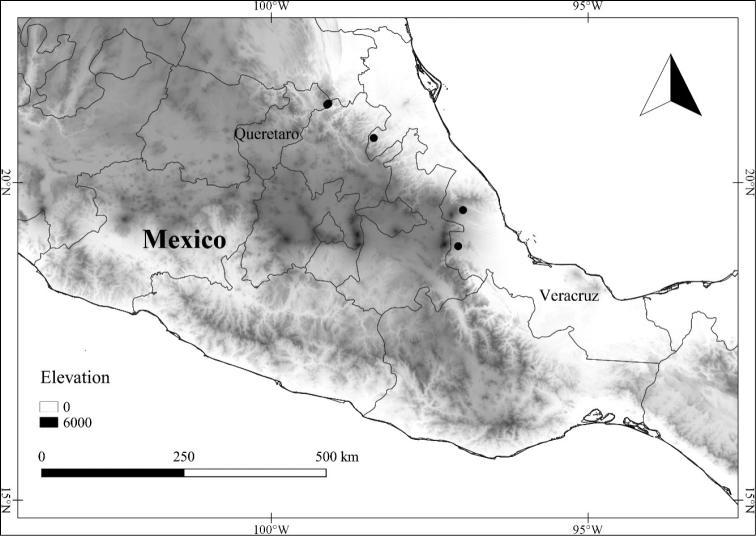
Map of geographic distribution of *L. glabripetala* based on herbarium specimen data.

Mexico (Querétaro, Veracruz) in tropical dry forest and cloud forest, including *Quercus*, *Carpinus*, and *Liquidambar* forest, in shady canyons and on slopes, sometimes on limestone, 1040–1450 m in elevation (Fig. 41).

##### Common names and uses.

None known.

##### Phenology.

Flowering specimens have been collected in June; specimens with mature fruits have been collected in January, July, and October. The timing of the corolla movements is not known, but since the corollas on the specimens of this species are open, the flowers are probably open during the day, as in the morphologically similar *Lycianthes amatitlanensis* (Coult. & Donn.Sm.) Bitter.

##### Preliminary conservation status.

*Lycianthes glabripetala* is a rarely collected species of endangered cloud forest habitat of central Mexico, represented by only six collections, two of which are from a protected area (Sierra Gorda). The EOO is 8,363.379 km^2^, and the AOO is 24 km^2^. Based on the IUCN (2019) criteria, the preliminary assessment category is Vulnerable (VU).

##### Discussion.

*Lycianthes glabripetala* is an endemic Mexican species morphologically similar to *L. amatitlanensis* (of Mexico and Central America), *L. inconspicua* (of Central America), and *L. inaequilatera* (of Central and South America). *Lycianthes glabripetala* differs from those species in combining woolly curved trichomes on the abaxial side of the leaves, a relatively large corolla (to 1.2 cm long), nearly glabrous surfaces on the abaxial side of the corolla lobes, and a pedicel length of 9–15 mm in flower and 12–20 mm in fruit. *Lycianthes amatitlanensis* usually has straight trichomes that project at a 90-degree angle from the midvein of the abaxial leaf surface, corollas 0.5–0.8 cm long, and very evident long trichomes on the abaxial side of the corolla lobes with these trichomes usually tufted at the tip of the lobe. *Lycianthes inconspicua* Bitter can have flowers as long as *L. glabripetala*, and has variable pubescence on the abaxial side of the corolla lobes, but it has longer pedicels (15–30 mm in flower and 30–35 mm in fruit) and delicate straight trichomes that are tightly appressed to the midvein of the abaxial leaf surface; it also differs in having oval anthers. *Lycianthes inaequilatera* has smaller corollas and has pubescence much like *L. inconspicua*, and it occurs far south of the range of *L. glabripetala* (Dean et al. 2018b).

*Lycianthes glabripetala* is known at this time from the highlands of central Mexico in the states of Querétaro and Veracruz in cloud forest vegetation above 1000 m in elevation; this habitat is similar to that of *L. inconspicua* but differs from the most common habitat of *L. amatitlanensis*, a species that is usually found below 1000 m in elevation, often below 500 m, in humid tropical forest.

##### Representative specimens examined.

**Mexico. Querétaro**: 1 km al sureste de El Naranjo, [21.2421, -99.1014], 1050 m, 24 Jul 1989 *H. Rubio 909* (IEB, DAV). **Veracruz**: Mpio. Zontecomatlán, along Huayacocotla-Zontecomatlán road, 1 km NE of San Antonio Ixtatetla, 20.7, -98.3833, 1300 m, 27 Apr 1983, *M. Nee 26820* (NY, XAL).

#### 18 Lycianthes gongylodes

Taxon classificationPlantae

J.L.Gentry, Phytologia 26: 274. 1973

Fig. 42

##### Type.

Guatemala. Huehuetenango: Mpio. San Mateo Ixtatán, 4 miles east of San Mateo Ixtatán on road to Barillas, 8500 ft, 7 Feb 1965, *D. Breedlove 8771* (holotype: F [0072910F, acc. # 1624724]; isotype: CAS [0003289]).

**Figure 42.**
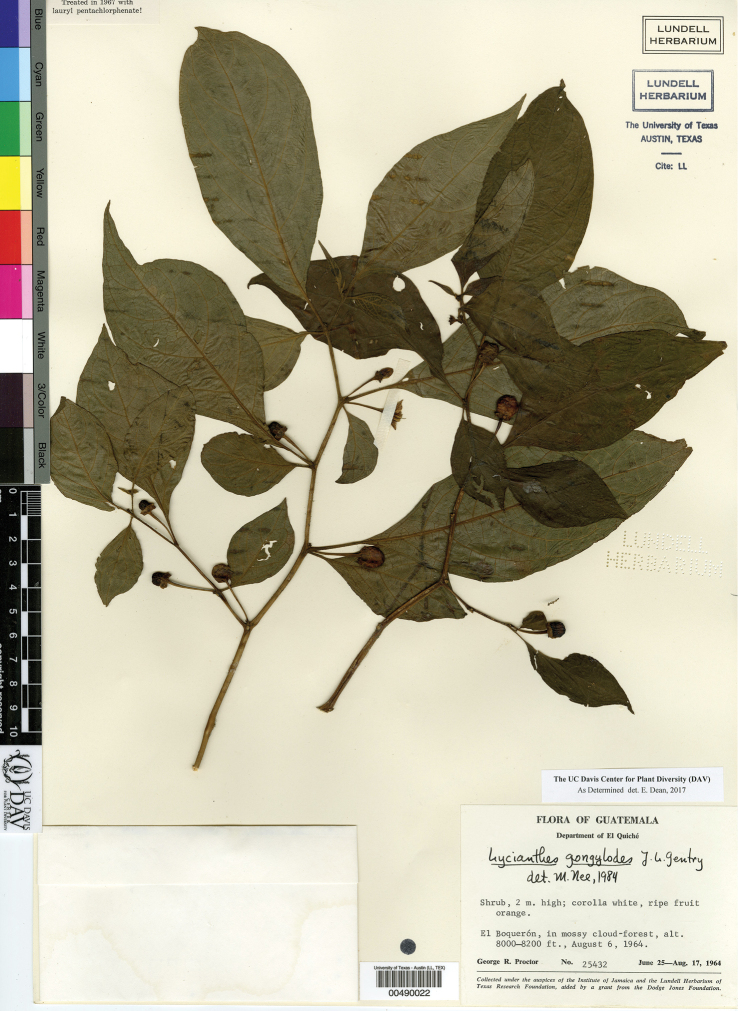
Image of herbarium specimen of *L. gongylodes*, *Proctor 25432* (LL). Specimen used with permission from the Lundell Herbarium, University of Texas at Austin.

##### Description.

Herb to shrub, erect, 1.5–3.5 m tall. Indument of pale yellow to light brown, uniseriate, multicellular, usually simple (sometimes dendritic), curling to crisped, eglandular, spreading to appressed trichomes 0.1–0.75 (1) mm long. Stems green when young, sparsely to moderately pubescent, compressed upon drying in a plant press, woody with age; upper sympodial branching points monochasial or dichasial. Leaves simple, the leaves of the upper sympodia usually paired and unequal in size, the larger ones with blades 7–17.5 × 3.5–6.5 cm, the smaller ones with blades 3.5–10 × 2–4 cm, the leaf pairs usually similar in shape, the blades ovate to elliptic, membranaceous, sparsely pubescent, especially along the veins, the base cuneate to attenuate, sometimes oblique, the margin entire, usually undulate, the apex acute to acuminate, the petiole 0.5–2.5 cm long, sometimes absent, the larger leaf blades with 5–6 primary veins on each side of the midvein. Flowers solitary or in groups of 2–5, axillary, erect or oriented horizontally; peduncles absent; pedicels 10–15 mm and erect to arching in flower, to 30 mm long and erect to arching in fruit; calyx 2–3 mm long, 3.5–4.5 mm in diameter, widely bowl shaped, glabrous to sparsely pubescent, the margin truncate, undulate, very well developed, with 10 very short, reflexed appendages 0.25–0.5 mm long emerging 1–1.5 mm below the calyx rim; fruiting calyx enlarged, widely bowl-shaped, sometimes appearing flat-bottomed, 1.5–3 mm long, 6–9 mm in diameter, the appendages not changing in length; corolla 0.6–1 cm long, rotate to reflexed in orientation, shallowly to deeply stellate in outline, sometimes divided to below the middle, interpetalar tissue present, white adaxially, glabrous, white abaxially, sparsely pubescent with very short trichomes; stamens equal, the filaments ca. 1 mm long, glabrous, the anthers 4–5 mm long, lanceolate, free of one another, pale yellow, glabrous, poricidal at the tips, the pores round, dehiscing distally; pistil with glabrous ovary, the style ca. 7 mm, linear, straight, glabrous, the stigma capitate, decurrent down two sides. Fruit a berry, 7–10 mm long, 7–9 mm in diameter, globose, orange at maturity, glabrous, lacking sclerotic granules. Seeds 20–60 per fruit, 2–2.5 mm × 1.5–2 mm, flattened, depressed ovate to oval in outline, with shallow notch on one side, yellow-orange, the surface reticulum with minute serpentine pattern with shallow luminae.

##### Chromosome number.

Unknown.

##### Distribution and habitat.

**Figure 43.**
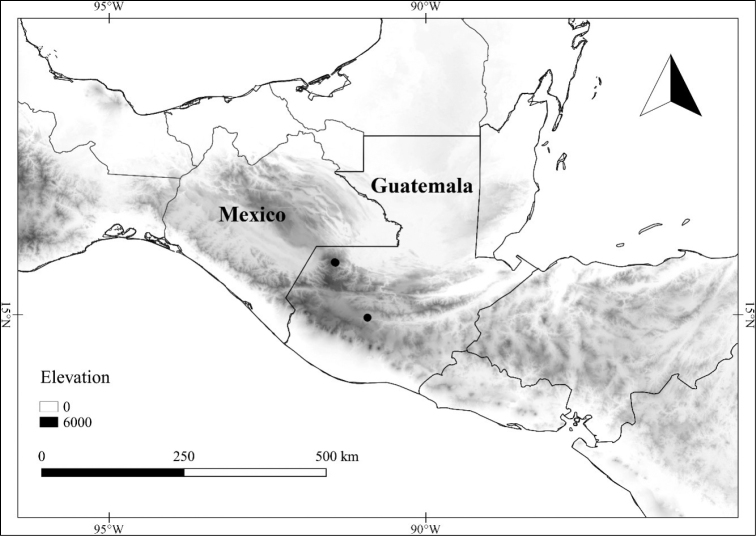
Map of geographic distribution of *L. gongylodes* based on herbarium specimen data.

Guatemala (Huehuetenango, Quiché), in cloud forest, 2400–3000 m in elevation (Fig. 43).

##### Common names and uses.

None known.

##### Phenology.

Flowering specimens and specimens with mature fruits have been collected in February and from June to August. All flowering specimens of this species have open flowers indicating that the flowers are probably open for most of the day.

##### Preliminary conservation status.

*Lycianthes gongylodes* is a rarely collected species of Guatemala, represented by only four collections, all made before 1970, and none from protected areas. The EOO is 43.078 km^2^, and the AOO is 12 km^2^. Based on the IUCN (2019) criteria, the preliminary assessment category is Critically Endangered (CR).

##### Discussion.

*Lycianthes gongylodes* is known from only four collections made by Breedlove, Proctor, and Steyermark. The species is most likely allied to *L. heteroclita*, with which it shares green herbaceous stems that collapse upon drying. The form of the calyx in the two species is similar, although *L. gongylodes* differs in having small calyx appendages which make the calyx appear thicker and more bowl-shaped in flower and flat-bottomed in fruit (a feature it shares with *L. connata*). *Lycianthes heteroclita* usually lacks appendages on the calyx, which looks campanulate in flower and bowl-shaped to plate-like in fruit and, unlike *L. gongylodes*, it usually lacks obvious interpetalar tissue connecting the lobes of the corolla. The curly trichomes that are present on the stems and leaves of *L. gongylodes* are quite distinctive and different than the very small trichomes present in *L. heteroclita*; in addition, *L. gongylodes* lacks the tiny groups of white trichomes that appear like small granules on the calyx of *L. heteroclita*. *Lycianthes gongylodes* could be confused with *L. ceratocalycia*, another allied species that occurs in the same region of Guatemala and adjacent Mexico. *Lycianthes ceratocalycia* differs in having purple, stellate corollas with sparse interpetalar tissue and scurfy horizontal lines on the young branches. A sterile specimen of *L. gongylodes* could be misidentified as *L. tricolor* (Dunal) Bitter, something that was done in Dean et al. (2017a), where *Steyermark 49839* (a paratype of *L. gongylodes*) is cited under *L. tricolor*.

##### Representative specimens examined.

**Guatemala. Huehuetenango**: Sierra de los Cuchumatanes, near the place called Kurus Lemun, 4 miles E of San Mateo Ixtatán along road to Barillas, [15.82, -91.4264], 8500 ft, 7 Aug 1965, *D. Breedlove 11628* (TEX). **Quiché**: El Boquerón, 8000–8200 ft, 6 Aug 1964, *G.R. Proctor 25432* (BRIT, MO, TEX).

#### 19 Lycianthes gorgonea

Taxon classificationPlantae

Bitter, Repert. Spec. Nov. Regni Veg. 20: 364. 1924

Fig. 44

Solanum cuspidatum C.V.Morton, Contr. Univ. Michigan Herb. 4: 25. 1940. Type: Belize, El Cayo District, on Arenal-Valentín road along roadside through high forest, 20–21 Jun 1936, *C. Lundell 6172* (holotype: US [acc. # 1688328]; isotypes: GH [00077476], LL [00372872], MI [1109941], MO [acc. # 1278667], NY [00138976], S [acc. # 04-2899]), TEX [00372873]).
Lycianthes cuspidata (C.V.Morton) Standl. & Steyerm., Publ. Field Mus. Nat. Hist., Bot. Ser. 23: 18. 1943. Type: Based on Solanum cuspidatum C.V.Morton.

##### Type.

Guatemala. Sacluc, Aug 1877, *K. Bernoulli & O. Cario 2357* (holotype: GOET [GOET003446]).

**Figure 44.**
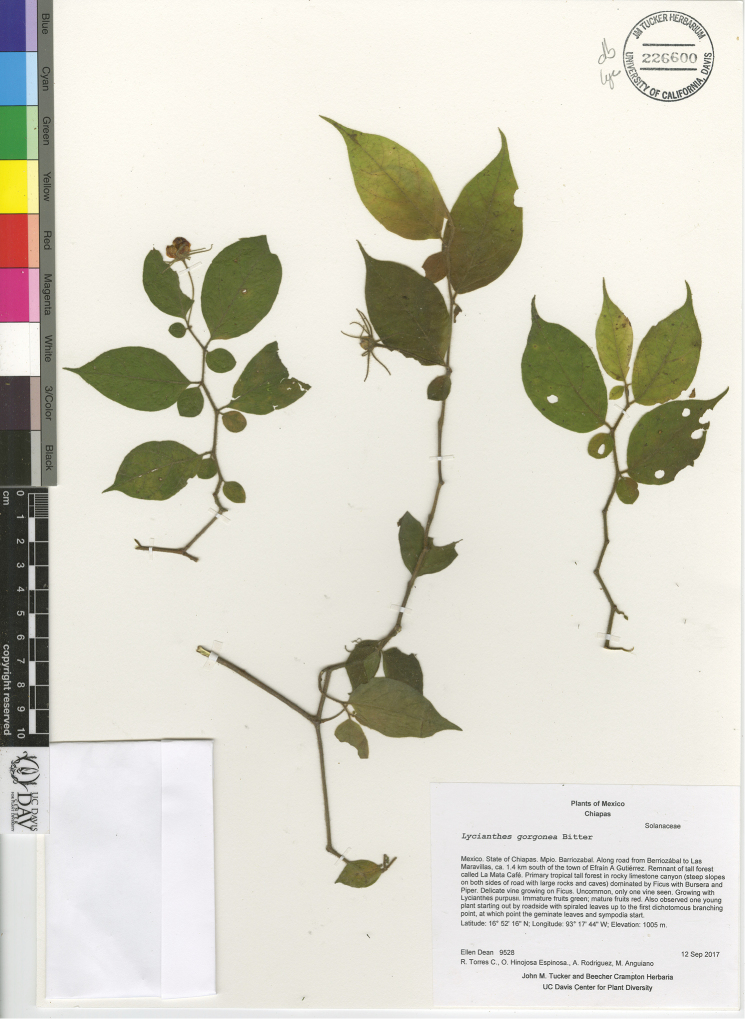
Image of herbarium specimen of *L. gorgonea*, *Dean 9528* (DAV). Image used with permission of the UC Davis Center for Plant Diversity.

##### Description.

Shrub to woody vine, 1–4 m tall. Indument of white to tan, uniseriate, multicellular, simple, eglandular and/or glandular, spreading trichomes 0.5–3 mm long. Stems greenish-tan when young, moderately to densely pubescent, not compressed when dried in a plant press, becoming woody with age; upper sympodial branching points a mixture of dichasial and monochasial, the branching divaricate and zigzagging. Leaves simple, the leaves of the upper sympodia usually paired, the leaf pairs often conspicuously different in size and shape, the larger ones with blades 4–11 × 2–4 cm, ovate to lanceolate, the smaller ones with blades 0.5–4 × 0.5–2 cm, orbicular to ovate, the leaf pairs similar in texture, membranaceous, moderately pubescent (densely pubescent along the midvein of the abaxial side), the base cuneate to rounded, sometimes oblique, the margin entire, usually undulate, the apex acute to acuminate, the petiole to 0.5 (1) cm long, sometimes absent, the larger leaf blades with 5–7 primary veins on each side of the midvein. Flowers solitary, axillary, oriented horizontally; peduncles absent; pedicels 15–25 mm and erect to arching in flower, to 40 mm long and spreading in fruit, densely pubescent; calyx 2–2.5 mm long, 3–3.5 mm in diameter, obconic to campanulate, densely pubescent (sometimes nearly obscured), the margin truncate, with 10 very long spreading linear appendages 7–15 mm long emerging 0.5 mm below the calyx rim; fruiting calyx usually enlarged, widely campanulate to bowl-shaped, 2.5–5 mm long, 6–9 mm in diameter, the appendages to 20 mm long, spreading; corolla 1–1.5 cm long, rotate in orientation, entire in outline, with abundant interpetalar tissue, white to pale blue-violet, glabrous adaxially, with long trichomes near the veins abaxially; stamens equal or nearly so, straight, the filaments 1–2 mm long, glabrous, the anthers 4–4.5 mm long, elliptic, connate to one another at their edges, yellow, glabrous, poricidal at the tips, the pores round, large, dehiscing distally, not opening into longitudinal slits; pistil with glabrous ovary, the style 7–9 mm long, linear, straight to curved, glabrous; stigma capitate, decurrent down two sides. Fruit a berry, 5–8 mm long, 5–9 mm in diameter, globose, red at maturity, glabrous, lacking sclerotic granules. Seeds 15–25 per fruit, 2.25–2.5 × 2.5–3 mm, flattened to unevenly thickened and curved, triangular to depressed ovate in outline, yellow-orange, the surface reticulum with minute serpentine pattern and shallow luminae.

##### Chromosome number.

Unknown.

##### Distribution and habitat.

**Figure 45.**
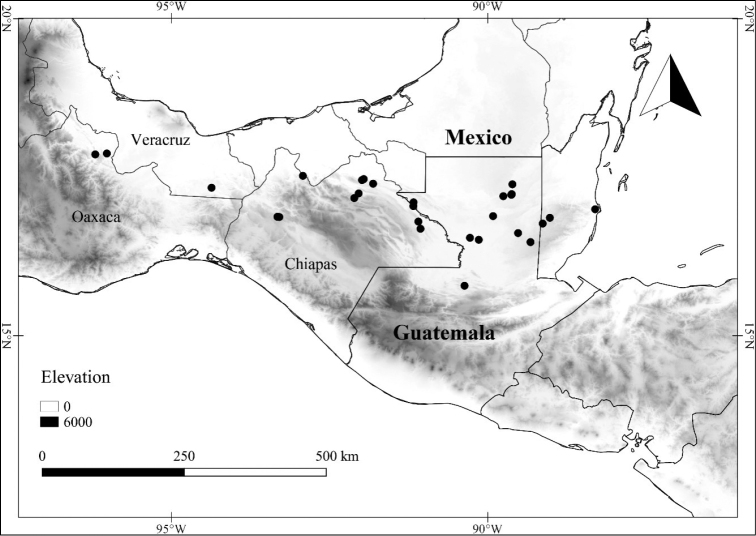
Map of geographic distribution of *L. gorgonea* based on herbarium specimen data.

Mexico (Chiapas, Oaxaca, Tabasco, Veracruz), Guatemala (Alta Verapaz, Petén), and Belize (El Cayo), in high forest, lower montane rain forest, and tropical moist forest, often on limestone ridges or in canyons, sometimes near streams, 200–1000 m in elevation (Fig. 45).

##### Common names and uses.

None known.

##### Phenology.

Flowering specimens have been collected November through August. Specimens with mature fruits have been collected April and July through November. Many specimens have been collected with immature fruits, and these have been collected throughout the year. In Belize, the corollas of this species open at sunrise and close at sunset (Smith and Knapp 2002).

##### Preliminary conservation status.

*Lycianthes gorgonea* is a species of lowlands of southern Mexico, Guatemala, and Belize, represented by 31 collections and occurring in six protected areas. The EOO is 79,973.926 km^2^, and the AOO is 108 km^2^. Based on the IUCN (2019) criteria, the preliminary assessment category is Least Concern (LC).

##### Discussion.

*Lycianthes gorgonea* is a distinctive species of lower elevation tropical rain forest, often found on limestone. The divaricate, zigzag branching of this species, in combination with a distinctive size/shape difference of the paired geminate leaves, soft long, pale trichomes, and very long calyx appendages, is quite different from any other species of *Lycianthes* in Mexico and Central America. The pollination of this species was studied in Belize by Smith and Knapp (2002), and they found that the flowers are visited by the bee genus *Paratetrapedia*.

##### Representative specimens examined.

**Guatemala. Alta Verapaz**: between Limón and Chisec, 200–230 m, 19 Mar 1942, *J.A. Steyermark 45116* (NY, LL). **Petén**: Uaxactun, on Dos Lagunas road, in zapotal/ramonal, 3 km W, 22 Jan 1977, *C. L. Lundell 20534* (MO, LL). **Mexico. Chiapas**: Mpio. Barriozábal, along road from Berriozábal to Las Maravillas, ca. 1.4 km S of the town of Efraín A. Gutiérrez, in remnant of tall forest called La Mata Café, 16.8711, -93.2956, 1005 m, 12 Sep 2017, *E. Dean 9528* (DAV). **Oaxaca**: Dto. Tuxtepec, predio La Joya del Obispo, [17.8599, -96.2060], 12 Aug 1990, *C. H. Ramos 435* (IEB, MEXU, XAL). **Tabasco**: ladera W del Cerro del Madrigal, cerca de la base, Puyacatengo, [17.5213, -92.9225], 25 Mar 1992, *M. A. Guadarrama-O. 1244* (TEX). **Veracruz**: Mpio. Minatitlán, 6.6 km al Norte de la terracería La Laguna-Río Grande, sobre el camino nuevo (no completo) a Ejido Belisario Domínguez, el cual sale de la terracería 14.7 km al E. de La Laguna, 17.3333, -94.3667, 130 m, 13 Jul 1980, *T. Wendt 2557* (MO).

#### 20 Lycianthes grandifolia

Taxon classificationPlantae

E.Dean, Brittonia 70: 479. 2018

Fig. 46

##### Type.

Mexico. Chiapas: [Mpio. Siltepec?] Letrero, near Siltepec [15.5564, -92.3233], 2000 m, 6 Jul 1941, *E. Matuda 4350* (holotype: MEXU [acc. # 82114]; isotypes: A [00934887], [LL [00226934, 00227071], MO [acc. # 1244040]).

**Figure 46.**
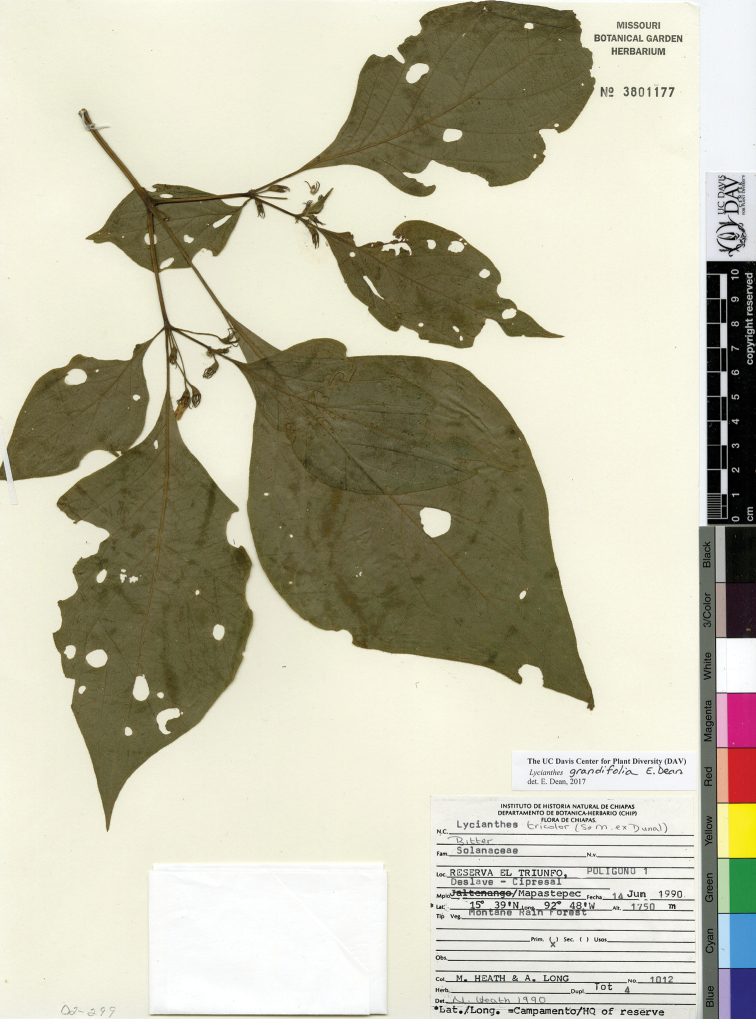
Image of herbarium specimen of *L. grandifolia*, *Heath 1012* (MO). Specimen used with permission from the Missouri Botanical Garden (http://www.tropicos.org).

##### Description.

Shrub, 2–3 m tall, erect. Indument of light yellow (sometimes appearing tan or off-white), uniseriate, multicellular, simple, eglandular, ascending-appressed to spreading trichomes 0.1–1.5 mm long. Stems green, angled when young, sparsely to moderately pubescent, somewhat compressed and ribbed when dried in a plant press, becoming brown and woody with age; upper sympodial branching points monochasial or dichasial. Leaves simple, the leaves of the upper sympodia usually paired and unequal in size, the larger ones with blades 8–22 × 5–12 cm, the smaller ones with blades 3–9 × 2–4.5 cm, the leaf pairs similar in shape, the blades ovate (often widely so), thin chartaceous, sparsely pubescent, ciliate along the margin, the base usually long attenuate (cuneate), sometimes oblique, the margin entire, usually irregularly undulate, the apex acuminate, the petiole 0.2–5 cm long, those of the longer leaves 2 cm long or longer, the larger leaf blades with 5–6 primary veins on each side of the midvein. Flowers in groups of 2–8, axillary, oriented horizontally; peduncles absent; pedicels 10–25 mm long and erect in flower, sparsely to moderately pubescent, mature fruiting pedicels not yet seen; calyx 2.5–3.5 mm long, 3–4 mm in diameter, obconic to campanulate, sparsely to moderately pubescent, the margin truncate, with 10 spreading linear appendages 2–7.5 mm long (at least some appendages on a calyx 4 mm long or longer), emerging 0.25–0.5 mm below the calyx rim; mature fruiting calyx not yet seen; corolla 0.8–1.6 cm long, rotate in orientation, mostly entire in outline (with shallow notches), with abundant interpetalar tissue, white, glabrous adaxially, the abaxial side of the lobes moderately puberulent near the major veins; stamens unequal, straight, the four short filaments 1–2 mm long, the one long filament 3–4.5 mm long, glabrous, the anthers 3–4 mm long, lanceolate, free of one another, yellow, glabrous, poricidal at the tips, the pores ovate, the pores of the longest stamen dehiscing toward the style, the pores of the shorter stamens dehiscing distally or away from the style, not opening into longitudinal slits; pistil with glabrous ovary, the style ca. 7 mm long, linear, glabrous, the stigma capitate, decurrent down two sides, slightly lobed. Fruit a berry, 4–5 mm long, 4–5 mm in diameter, globose to depressed-globose, green when immature (mature fruit not yet seen), glabrous, lacking sclerotic granules. Seeds 10–30 per fruit, only seen when immature, mature size and details not yet known, not notched.

##### Chromosome number.

Unknown.

##### Distribution and habitat.

**Figure 47.**
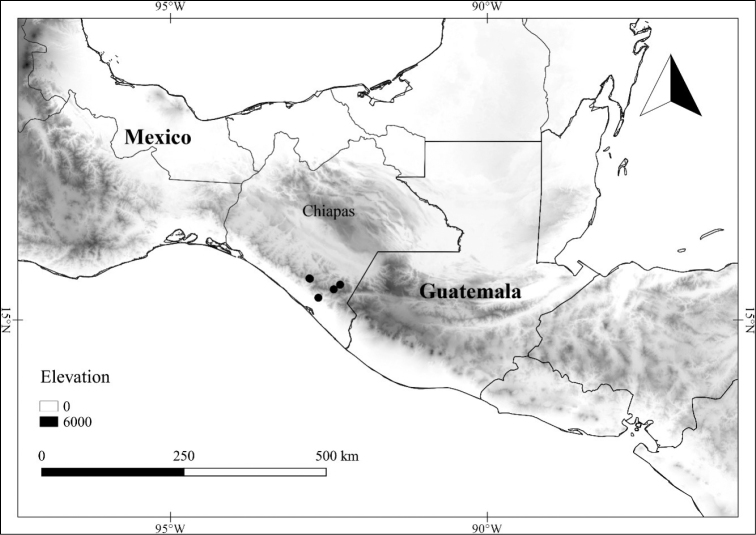
Map of geographic distribution of *L. grandifolia* based on herbarium specimen data.

Mexico (Chiapas), in cloud forest, 1700–2000 m in elevation (Fig. 47).

##### Common names and uses.

None known.

##### Phenology.

Flowering specimens have been collected in June and July, presumably fruiting after that. The corollas on the specimens of this species are usually closed, indicating that the corollas may just open very early in the morning and then close by late morning.

##### Preliminary conservation status.

*Lycianthes grandifolia* is a rarely collected species of southern Mexico, represented by only five collections, two from the protected area El Triunfo. The EOO is 828.005.215 km^2^, and the AOO is 16 km^2^. Based on the IUCN (2019) criteria, the preliminary assessment category is Endangered (EN).

##### Discussion.

This species belongs to series *Tricolores* and is distinguished from most of the other species in the series by long and broad leaves and occurring in cloud forest at elevations close to 2000 m. Every specimen of this species that we have examined has at least one leaf with a length greater than or equal to 18 cm. Other species within series *Tricolores* with leaves that can reach this length (but not typically) are *L. arrazolensis*, *L. jalicensis*, and *L. michaelneei*. *Lycianthes grandifolia* differs from *L. michaelneei* in having white corollas (rather than purple) and having less dense pubescence. It differs from *L. arrazolensis* by usually having longer calyx appendages that are inserted < 0.5 mm from the calyx rim (versus > 0.5 mm). It differs from *L. jalicensis* in having pubescent calyces and corollas and occurring at elevations near 2000 m (versus < 1400 m) (Dean et al. 2018c).

*Lycianthes grandifolia* occurs in habitats similar to those of *L. tricolor* (high elevation forest types), and it has pedicels of similar length. It differs from *L. tricolor* by longer leaves and angled and ribbed young stems that compress when dried in a plant press. We have not seen the mature fruits or seeds of *L. grandifolia*, however the immature seeds resemble those of *L. arrazolensis*, which are unnotched, rather than those of *L. tricolor*, which are notched (Dean et al. 2018c).

This species is poorly known and has been rarely collected. One collection from southeastern Chiapas that may belong to *L. grandifolia*: Mt. Pasitar (Paxtar), 3–4 Aug 1937, *Matuda 1642* (MO-1807983; US-1807982) differs in having the calyx appendages densely pubescent with long, spreading trichomes (2 mm long). Therefore, we did not include it in the species description. The elevation and exact location of this collection is unknown. If from low elevations, it may represent a new species belonging to series *Tricolores*. More fieldwork is necessary to fill out the morphological details of *L. grandifolia* (Dean et al. 2018c)

##### Representative specimen examined.

**Mexico. Chiapas**: Mpio. Mapastepec, Reserva El Triunfo, Deslave-Cipresal, 15.65, -92.8, 1750 m, 14 Jun 1990, *M. Heath 1012* (MO3801177).

#### 21 Lycianthes heteroclita

Taxon classificationPlantae

(Sendtn.) Bitter, Abh. Naturwiss. Verein Bremen 24 [preprint]: 494. 1919

Fig. 48

Solanum heteroclitum Sendtn. Flora 29 (13): 193 [as 177]. 1846. Type: Guatemala. No exact location, *Friedrichsthal s.n.* (lectotype designated here: W [acc. # 0003646]).
Brachistus escuintlensis J.M.Coult., Bot. Gaz. 16: 144. 1891. Type: Guatemala. Escuintla: Escuintla, 1100 ft, Mar 1890, *J. Donnell Smith 2267* (lectotype designated by D’Arcy 1973b, pg. 116: US [00624003, acc. # 1335155]; isolectotypes: F [F0072758F, acc. # 267053], G [G00379121], GH [00076928], K [K000585751], M [M-0171852]).
Bassovia purpusii Brandeg. Univ. Calif. Publ. Bot. 6: 372. 1917. Type: Mexico. Veracruz: Zacuapan [ca. 19°13'N, 96°53'W, 1025 m], Jul 1915, *C. Purpus 7502* (holotype: UC [178570]).
Solanum mitratum Greenm., Bot. Gaz. 37: 211. 1904. Type: Costa Rica. Cartago: Atirro, 600 m, Mar [April on US specimen] 1896, *J. Donnell Smith 6673* (lectotype designated by D’Arcy 1973a, pg. 636: GH [00077517]; isolectotype US [00624007]).
Lycianthes mitrata (Greenm.) Bitter, Abh. Naturwiss. Verein Bremen 24 [preprint]: 500. 1919. Type: Based on Solanum mitratum Greenm.
Lycianthes heteroclita (Sendtn.) Bitter ssp. coalescens Bitter, Abh. Naturwiss. Verein Bremen 24 [preprint]: 496. 1919. Type: Guatemala. Alta Verapaz: Cubilqüitz, [Cubilhuitz], [15.6675, -90.4293], 350 m, Aug 1907, *H. von Tuerkheim II 813* (lectotype designated by D’Arcy (1973a, pg. 636): US [00027880]; isolectotypes: G, NY, W [acc. # 1908-3590, acc. # 1908-3589]).
Lycianthes heteroclita (Sendtn.) Bitter var. gracilis Bitter, Abh. Naturwiss. Verein Bremen 24 [preprint]: 496. 1919. Type: Panama. Canal Zone: Railroad relocation between Gorgona and Gatun, 10–50 m, *Pittier 2281* (lectotype designated by Dean and Reyes 2018a, pg. 42: US [acc. # 676535]; isolectotype US [acc. # 676536]).
Bassovia escuintlensis (J.M.Coult.) Standl., Contrib. U. S. Natl. Herb. 23: 1304. 1924. Type: Based on Brachistus escuintlensis J.M.Coult.
Capsicum escuintlense (J.M.Coult.) Standl., Publ. Field Mus. Nat. Hist., Bot. Ser. 12: 347 1936. Type: Based on Brachistus escuintlensis J.M.Coult.
Solanum escuintlense (J.M.Coult.) Hunz., Kurtziana 5: 166. 1969. Type: Based on Brachistus escuintlensis J.M.Coult.
Lycianthes escuintlensis (J.M.Coult.) D’Arcy, Phytologia 25: 116. 1973. Type: Based on Brachistus escuintlensis J.M.Coult.

##### Type.

Based on *Solanum heteroclitum* Sendtn.

**Figure 48.**
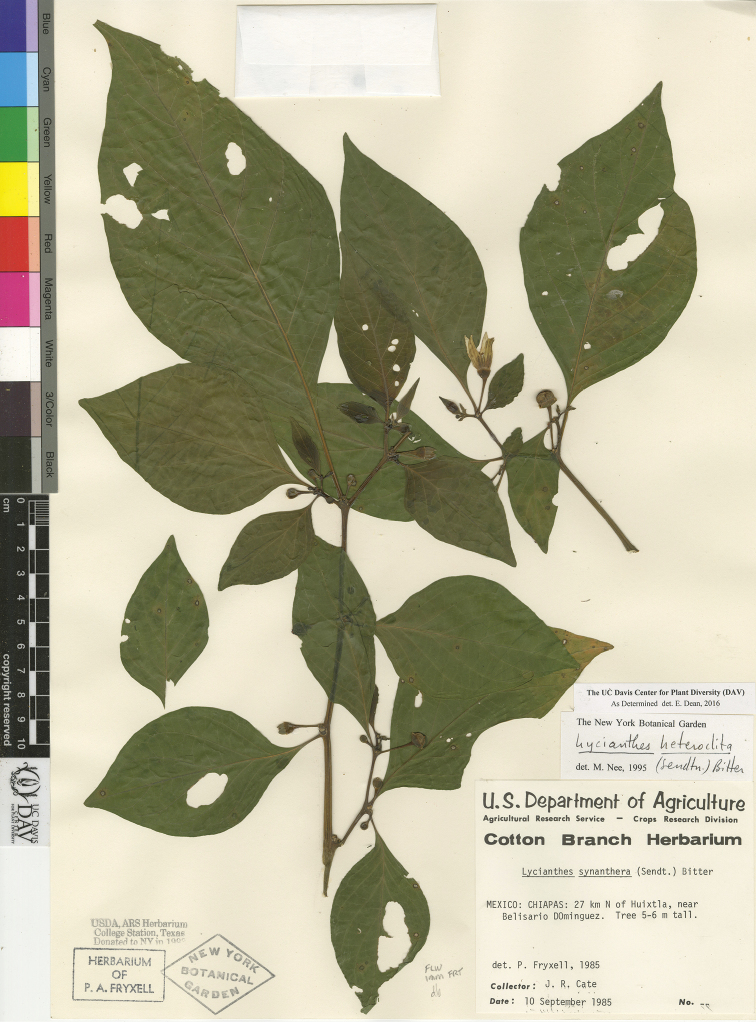
Image of herbarium specimen of *L. heteroclita*, *Cate s.n.* (NY). Specimen used with permission from the William and Lynda Steere Herbarium, New York Botanical Garden.

##### Description.

Herb, shrub, to treelet, sometimes epiphytic, erect, 0.5–5 m tall. Indument of very small, white to tan, uniseriate, multicellular, simple, eglandular, appressed-ascending trichomes < 0.1 (0.2) mm long. Stems green when young, glabrous to sparsely pubescent, compressed (hollowed at the nodes) upon drying in a plant press, woody with age; upper sympodial branching points monochasial or dichasial. Leaves simple, the leaves of the upper sympodia usually paired and unequal in size, the larger ones with blades 7–32 × 2.5–15 cm, the smaller ones with blades 3–12 × 1.5–6 cm, the leaf pairs usually similar in shape, the blades ovate to elliptic (rarely obovate), thin-membranaceous, glabrous to very sparsely pubescent, the base cuneate to attenuate, often oblique, the margin entire, usually undulate, the apex acute to acuminate, the petiole 0.3–5 cm long, the larger leaf blades with 5–8 primary veins on each side of the midvein. Flowers solitary or in groups of 2–12, axillary, erect; peduncles absent; pedicels 8–20 mm and erect in flower, to 25 mm long and erect in fruit, usually glabrous to sparsely pubescent; calyx 2–3 (4) mm long, 3–6 mm in diameter, campanulate, sparsely puberulent, the margin truncate, undulate, the appendages lacking (but ribs sometimes prominent and dark green); fruiting calyx enlarged, widely bowl-shaped to plate-like and slightly reflexed, 1–3.5 mm long, 4.5–10 mm in diameter; corolla 1–1.6 cm long, campanulate to reflexed in orientation, stellate in outline, usually divided 3/4 of the way to all of the way to the base, the lobes usually with scarce interpetalar tissue, white to lilac and glabrous adaxially, white to green abaxially, puberulent abaxially; stamens equal, straight, the filaments 0.5–1.5 mm long, glabrous, the anthers 4–7 mm long, lanceolate, usually partially connivent or connate to the adjacent anther (at least near the middle or base of the anther, not at the tips), white to yellow, glabrous, poricidal at the tips, the pores round, dehiscing distally, not opening into longitudinal slits; pistil with glabrous ovary, the style 8–11 mm long, linear, straight or curved at tip, glabrous, the stigma oblong. Fruit a berry, 6–13 mm long, 8–13 (15) mm in diameter, globose to depressed globose, orange to red at maturity, glabrous, lacking sclerotic granules. Seeds 50–300 per fruit, 1–2.5 × 1–1.5 mm, flattened, circular, ovate, depressed ovate or somewhat triangular in outline, yellow-orange to orange-brown, often lighter in color near the margin, the surface reticulum with minute, serpentine pattern and shallow luminae.

##### Chromosome number.

Unknown.

##### Distribution and habitat.

**Figure 49.**
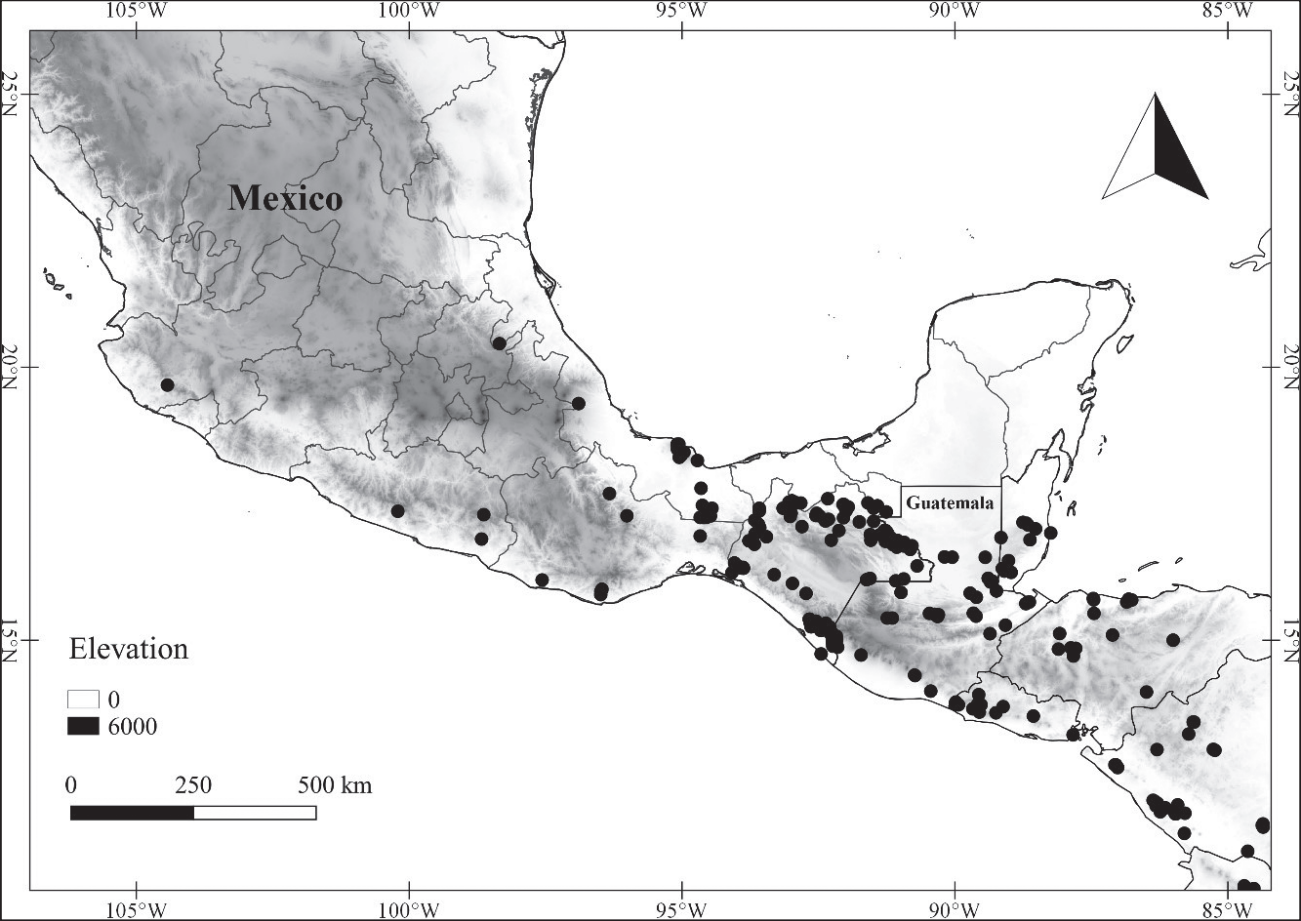
Map of geographic distribution of *L. heteroclita* based on herbarium specimen data.

Mexico (Chiapas, Guerrero, Jalisco, Oaxaca, Tabasco, Veracruz), Guatemala (Alta Verapaz, Escuintla, Huehuetenango, Izabal, Petén, Quetzaltenango, Quiché, Santa Rosa, Zacapan), Belize, El Salvador, Honduras, Nicaragua, Costa Rica, and Panama, in tropical rainforest, tropical moist forest, tropical dry forest, cloud forest, rarely in shrublands, sometimes along streams or on limestone, common in secondary forest, agricultural areas (such as coffee plantations), and along roadsides, 0–1000 (2000) m in elevation, perhaps cultivated in some areas (Fig. 49).

##### Common names and uses.

Mexico. Chiapas: fruits and leaves eaten after cooking in water, yerbabuena (Spanish), ashinte (Tzeltal) (*L. Bohs 3962*); ch’ayok (Maya Lacandon) (*M. González-Espinosa 755*); tumat tez (Tzeltal) (*A. Méndez Ton 5132*); ajchkintez (Tzeltal) (*Alush Méndez Ton 6741*); leaves eaten (*S. Levy T. 43*); quilete (*Matuda 17477*); zajchkintez (Tzeltal) (*A. Méndez T. 5215*); xote nuk (Mayan) (*B. Paniagua 57*); the leaves are eaten boiled, used medicinally for bones (utz’ak abaker), ubojo ch’ayok’ (*B. Paniagua 264*). **Guerrero**: eaten boiled as an edible green (Mixtec) (*K. Velazco-G. 40341*). **Veracruz**: hierba mora, hierba mora cimarrona (*W. Marquez R. 256, 275*).

##### Phenology.

Flowering specimens and specimens with mature fruits have been collected throughout the year in most locations. Corollas usually open in very early morning, closing in late morning.

##### Preliminary conservation status.

*Lycianthes heteroclita* is a widespread species ranging from Mexico to Panama, represented by many collections. The EOO is 1,336,839.05 km^2^, and the AOO is 1,328 km^2^. Based on the IUCN (2019) criteria, the preliminary assessment category is Least Concern (LC).

##### Discussion.

*Lycianthes heteroclita* is one of the most common and wide-ranging *Lycianthes* species in Central America. Its lifeform ranges from a large herb growing epiphytically or on the ground to a treelet, becoming woody at the base. When pressed, its herbaceous stems compress at the nodes. The variability in the flowers of this species deserves more study. From Mexico to Nicaragua, the corolla is usually white on the adaxial side and deeply stellate with very little interpetalar tissue. However, populations with corollas with more interpetalar tissue at the base of the lobes have been observed and collected in Mexico (Chiapas), Guatemala, El Salvador, and Nicaragua; this form of the corolla may be stellate only half-way to the base. In Costa Rica and Panama, the corollas are usually deeply stellate and purple on the adaxial side; this form was first described as *Solanum mitratum* (equal to *L. mitrata*) and later by Georg Bitter as L. heteroclita var. gracilis (both listed as synonyms above). Further study may show that these flower variants are geographically distinct and deserve recognition at the species level.

In Mexico, *Lycianthes heteroclita* is sometimes confused with the Mexican endemic *L. geminiflora*. In general, the two species can be separated by flowering calyx size, with that of *L. heteroclita* usually equal to or greater than 2 mm in length and that of *L. geminiflora* usually up to 1.5 mm in length. Also, *L. heteroclita* is usually found at lower elevations than *L. geminiflora*, which is a high-elevation cloud forest species, but the two species do co-occur in Oaxaca and Veracruz. In the areas of overlap, *L. heteroclita* is usually found below 1000 m, while *L. geminiflora* is usually found above 800 m. In addition, *L. heteroclita* specimens have been collected in Guatemala at elevations of 2000 m or more, which is unusual. In Chiapas, Honduras and Costa Rica, there are many specimens collected from elevations between 1200 and 1700 m, as well as elevations below 1000 m.

Confusion about what name to use for this species exists in the literature. In his treatment of the Solanaceae for Flora of Panama, D’Arcy (1973a) used the name *Lycianthes esquintlensis* for this species, and under that name, he synonymized the names L. heteroclita ssp. coalescens and *Solanum mitratum*. D’Arcy also included the species *L. synanthera* in his treatment. But he then annotated many specimens of *L. synanthera* as *L. esquintlensis*. In addition, in their treatment of *Lycianthes* for the Flora of Guatemala, Gentry and Standley (1974) only recognized *L. synanthera* and synonymized *L. heteroclita*, *L. escuintlensis*, and *S. mitratum* under that name, and their annotations reflected this. This created confusion in the identification of this species for several decades. Nee (1986) in his treatment for The Flora of Veracruz and Bohs (2015) in her recent treatment for the Flora of Costa Rica have corrected these errors.

Where they co-occur, *Lycianthes heteroclita* may be confused with *L. synanthera* and *L. nitida* Bitter, because all three species have calyces lacking appendages and may be epiphytic. Both *L. synanthera* and *L. nitida* have woody upper stems that do not collapse at the nodes upon drying. In addition, *L. synanthera* usually has hairs in the axils of the primary veins on the abaxial leaf surface, while *L. nitida* has coriaceous leaves with geminate pairs in which the minor leaf is much smaller in size and more rounded than the larger leaf.

The name *Solanum heteroclitum* Sendtn. is lectotypified here. In the protologue, Sendtner (1846) cites only one collection: Guatemala. No exact location, *Friedrichsthal s.n.*, but does not specify a particular specimen or herbarium. We were only able to locate one specimen seen by Sendtner at W [acc. # 0003646], and we are choosing that specimen as the lectotype.

##### Representative specimens examined.

**Guatemala. Alta Verapaz**: city of Cobán, garden of residence along 1^st^ St belonging to the family of Fredy Archila, seed of this plant originally collected at Finca Siguanha in forest at 1400 m in elevation, 15.4749, -90.3722, 1335 m, 9 Aug 2017, *E. Dean 9500* (DAV). **Escuintla**: Palín Finca Comunal, El Chilar, 14.3536, -90.7283, 959 m, 10 Nov 2010, *M. Véliz 22280* (BIGU). **Huehuetenango**: Between Ixcan and Finca San Rafael, Sierra de los Cuchumatanes 200–800 m, 24 Jul 1942, *J.A. Steyermark 49399* (NY). **Izabal**: W of Santo Tomás de Castilla, near Guatel antennas on one of the summits of Cerro San Gil, 15.6703, -88.6997, 800–900 m, 21 Sep 1997, *Nee 47320* (MO, NY). **Petén**: La Cumbre. Pusila road, 5 km, 17 Aug 1976, *C.L. Lundell 20194* (MO, LL). **Quetzaltenango**: Colomba, Fca. San Francisco Pie de la Cuesta, 14.7281, -91.7178, 1113 m, 15 Feb 2011, *L. Velásquez 1728* (BIGU). **Quiché**: Nebaj, about 12 km west, [15.4058, -91.1461], 8000 ft, 4 Jul 1964, *E. Contreras 5196* (MEXU, NY, LL, TEX). **Santa Rosa**: Region of Platanares, between Taxisco and Guazacapán, 220 m, 3 Dec 1940, *P.C. Standley 79062* (MO). **Zacapan**: Gualan, 122 m, 28 Dec 1905, *W.A. Kellerman 5678* (LL). **Mexico. Chiapas**: road from Ocosingo to Palenque in pueblo de Bahtaj, about 26 km NE of Ocosingo, roadside, 17.1447, -92.1283, 858 m, 5 Dec 2012, *L. Bohs 3962* (DAV, MEXU). **Guerrero**: Yoloxóchitl, 4.37 km en línea recta al norte de la comunidad, en el terreno del Sr. Enrique Rómulo (tierras de uso común), sobre el arroyo que va al paraje Salto de la Mona, 16.8531, -98.6723, 545 m, 22 Apr 2017, *K. Velazco-G. 40341* (DAV). **Jalisco**: Mpio. Casimiro Castillo, parte poniente del puente “La Calera” por la carretera Guadalajara-Barra de Navidad, [19.6730, -104.4287], 550 m, 17 Aug 1990, *R. López -V. 213* (WIS). **Oaxaca**: Sierra de Juárez, Mpio. Comaltepec, along Hwy 175 between km 66 and 67 just south of the town of Metates, 17.6860, -96.3289, 870 m, 11 Sep 2017, *E. Dean 9524* (DAV). **Tabasco**: Sierra El Madrigal, al E del edificio principal del centro regional Tropical Puyacatengo, [17.5172, -92.9028], 600 m, 6 Jun 1991, *A.M.H. Alipi 440* (MEXU). **Veracruz**: Cerro del Marinero, poblado Adolfo López Mateos, 18 km este de Catemaco, 18.4444, -94.9633, 500 m, 8 Jun 1991, *M. Torres 495* (MEXU).

#### 22 Lycianthes hintonii

Taxon classificationPlantae

E.Dean, Bot. J. Linn. Soc. 145: 407. 2004

Fig. 50

##### Type.

Mexico. Nuevo León: Mpio. Aramberri, Cerro El Viejo, 1200 m, 28 Jul1993, *Hinton et al*. *22882* (holotype: DAV [DAV155244]; isotypes: CIIDIR [CIIDIR022490]; GBH [GBH022882]; TEX [00208091]).

**Figure 50.**
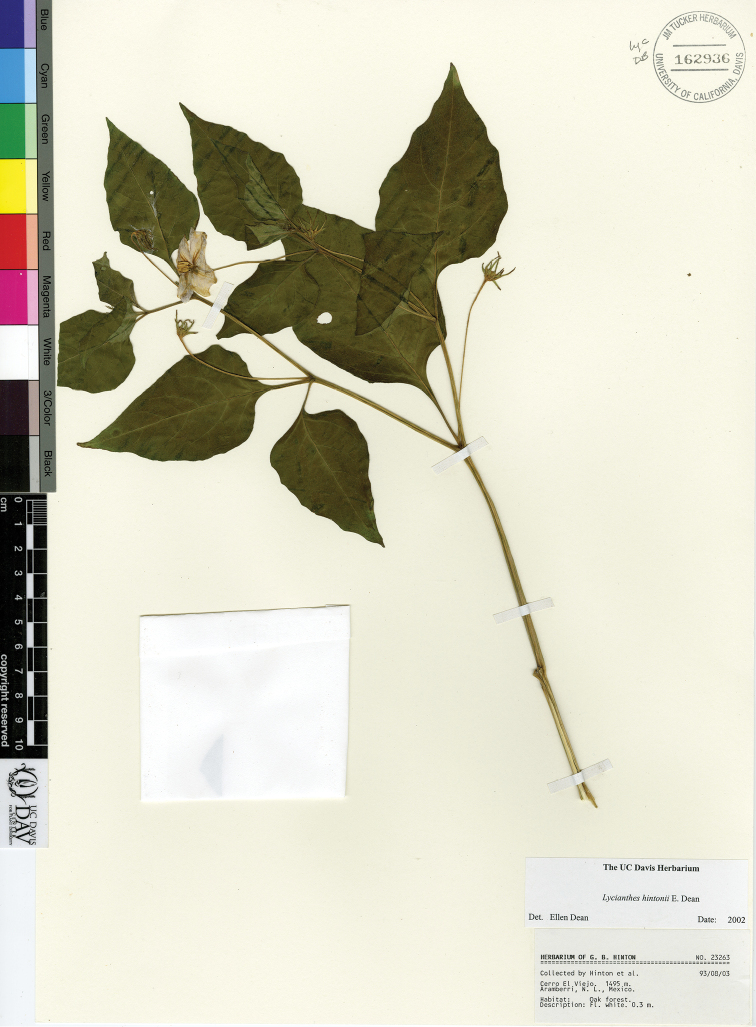
Image of herbarium specimen of *L. hintonii*, *Hinton 23263* (DAV). Image used with permission of the UC Davis Center for Plant Diversity.

##### Description.

Perennial herb from storage roots of unknown shape, usually erect, to ca. 0.5 m tall, dying back each season. Indument of white, uniseriate, multicellular, simple (rarely forked or dendritically branched), eglandular, spreading to appressed-retrorse trichomes 0.1–1 mm long. Stems green with darker green vertical markings, sparsely to moderately pubescent, somewhat compressed upon drying in a plant press, somewhat woody with age, especially at the base of the plant; first stem ca. 25 cm long to the first inflorescence, the internodes ca. 13; first two sympodial branching points dichasial, followed by monochasial branching. Leaves simple, those of the upper sympodia usually paired and unequal in size, the larger ones with blades 8–15 × 5.5–6.5 cm, the smaller ones with blades 1/2 to 2/3 the size of the larger, the leaf pairs similar in shape, the blades deltoid, ovate, or elliptic, thin chartaceous, sparsely pubescent, the primary veins 4–5 on either side of the midvein, the base attenuate or decurrent onto the petiole, slightly oblique on smaller leaves, the margin entire, and usually irregularly undulate, the apex rounded, acute, or short-acuminate, the petioles poorly defined, 1–3 cm long, sometimes absent. Flowers solitary, axillary, oriented horizontally; peduncles absent; pedicels 40–70 mm and erect in flower, ca. 110 mm long (or longer) in fruit (material with mature fruits and fruiting pedicels not yet seen), sparsely pubescent with spreading to appressed trichomes; calyx 3–4 mm long, 4–5 mm in diameter, campanulate, sparsely pubescent, the margin truncate, with 10 linear, spreading to reflexed appendages 4–11 mm long emerging ca. 1 mm below the calyx rim; fruiting calyx not yet seen; corolla 1–2.5 cm long (ca. 2–4 cm in diameter), rotate in orientation, mostly entire in outline (with shallow notches), with abundant interpetalar tissue, white, green near the major veins abaxially, glabrous; stamens unequal, straight, the filaments of three lengths, the two shortest filaments 1.5–3.5 mm long, the two medium filaments 2.5–4 mm long, the one long filament 3–6 mm long, the length of the long filament nearly always 1.2–1.5 times that of the medium filament, glabrous, the anthers 4.5–6 mm long, ovate-lanceolate, free of one another, yellow, glabrous, poricidal at the tips, the pores ovate, dehiscing distally, not opening into longitudinal slits; pollen grains tricolporate; pistil with glabrous ovary, the style 8.5–11 mm long, linear, slightly curved, glabrous, the stigma capitate (sometimes weakly bilobed). Fruits and seeds not yet seen.

##### Chromosome number.

Unknown.

##### Distribution and habitat.

**Figure 51.**
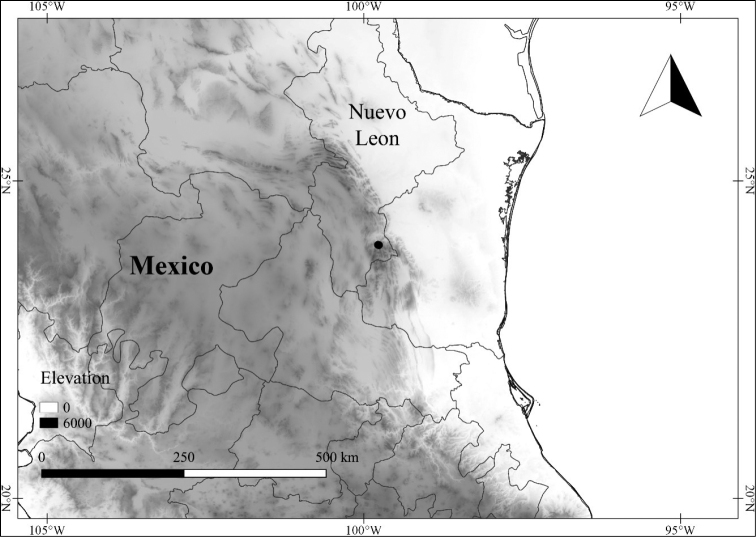
Map of geographic distribution of *L. hintonii* based on herbarium specimen data.

Mexico (Nuevo León), in oak forests on limestone soils in the mountains in the vicinity of Cerro El Viejo, 1200–1500 m in elevation (Fig. 51).

##### Common names and uses.

None known.

##### Phenology.

Flowering specimens have been collected July to August. Fruit not yet seen. The diurnal movements of the corolla have not been observed in the field; the corollas are probably open in the early morning and closed in the late morning. Unlike the other herbs of series *Meizonodonatae*, the scent of the pollen of this species is unknown.

##### Preliminary conservation status.

*Lycianthes hintonii* is a rarely collected species of northern Mexico, represented by only two collections made before 1993, both from the same location (Cerro El Viejo, Nuevo León), which is not a protected area. Anguiano-Constante et al. (2018) provided a preliminary assessment of Critically Endangered (CR).

##### Discussion.

Although this species has not been observed in the field, it is obviously related to the species of series *Meizonodontae* and it is assumed that it has the characteristic tuberous roots. The fruit type is unknown and could be either green, like *Lycianthes moziniana*, or dark purple like *L. ciliolata*. This species is similar to *L. rzedowskii* in its white flowers, but it differs from that species in having fewer, larger leaves on the first stem to emerge from the ground, triangular, tricolporate pollen, and in growing in basic limestone soils. Its distribution is quite disjunct from the other populations of *L. rzedowskii* (Dean 2004).

##### Representative specimen examined.

**Mexico. Nuevo León**: Mpio. Aramberri, Cerro El Viejo, [23.9885, -99.7612], 1495 m, 3 Aug 1993, *Hinton 23263* (DAV, GBH, TEX, UC).

#### 23 Lycianthes hypoleuca

Taxon classificationPlantae

Standl., Trop. Woods 9: 12. 1927

Fig. 52

Solanum hypoleucum (Standl.) C.V.Morton, Contr. Univ. Michigan Herb. 4: 27. 1940. Type: Based on Lycianthes hypoleuca Standl.

##### Type.

Belize. Orange Walk: 10 Oct 1926, *H. W. Winzerling V-14* (holotype: US [00027883]; isotypes: F [0072913F, acc. # 573777]), G [G00379122], WIS [00000961MAD]).

**Figure 52.**
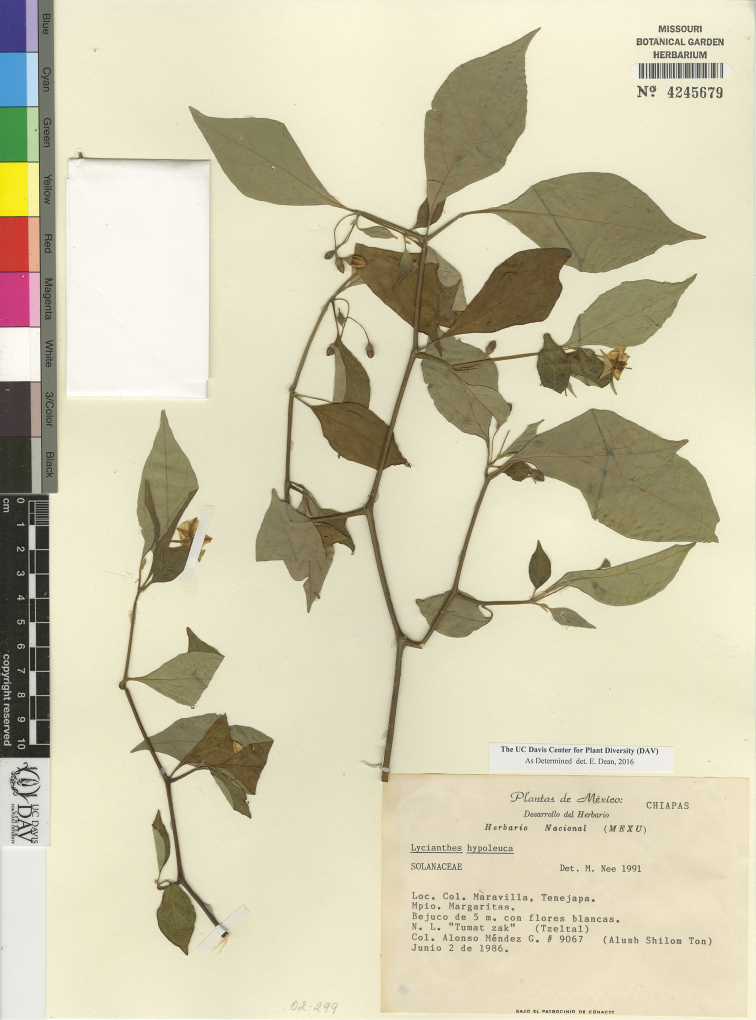
Image of herbarium specimen of *L. hypoleuca*, *Mendéz 9067* (MO). Specimen used with permission from the Missouri Botanical Garden (http://www.tropicos.org).

##### Description.

Scandent shrub to twining woody liana, 2.5–5 m tall (or taller, described as climbing high into the tree canopy). Indument white to tan, uniseriate, multicellular, sessile to short-stalked, stellate or multangulate-stellate, eglandular, spreading trichomes 0.1–0.2 mm long, 0.1–0.25 mm in diameter, the rays 3–6 per whorl, straight, not rebranched. Young stems greenish, sparsely to densely pubescent, compressed at the nodes when dried in a plant press, becoming dark reddish brown and woody with age; upper sympodial branching points monochasial and dichasial. Leaves simple, the leaves of the upper sympodia paired or not, the pairs unequal in size, the larger ones with blades 3–11 (12.8) × (1.8) 1.5–5 cm, the smaller ones (often not developing) with blades 2.5–6.2 × 1–3.6 cm, the leaf pairs similar in shape, the blades ovate, elliptic, or obovate, chartaceous, the two sides of the blade very different in color, the adaxial side green, glabrous, the abaxial side pale, densely pubescent with overlapping trichomes, the base cuneate to attenuate, sometimes oblique, the margin entire, usually irregularly undulate, the apex acute to acuminate, the petiole 0.2–2.5 cm long, sometimes absent, the larger leaf blades with 3–6 primary veins on each side of the midvein. Flowers solitary or in groups of 2–3, axillary, oriented horizontally; peduncles absent; pedicels 12–33 mm long and erect to arching in flower, 20–35 mm long (probably longer) and erect in fruit; calyx 2.5–4.5 mm long, 2.5–4 mm in diameter, campanulate, moderately to densely pubescent, the margin truncate, undulate or lobed, the appendages lacking; fruiting calyx enlarged, bowl-shaped to rotate, 2.5–4 mm long, 6–8 mm in diameter; corolla 0.8–1.2 cm long, rotate in orientation, entire to shallowly stellate in outline, divided ca. 1/4 of the way to the base), with abundant interpetalar tissue, white, adaxially sometimes with green markings at the base of the lobes, glabrous, abaxially moderately puberulent near the major veins; stamens equal to slightly unequal, straight, the four short filaments ca. 1 mm long, the one long filament 1–2 mm long, glabrous, the anthers 3–4 mm long, ovate to oblong, the tips narrowed, free from one another, yellow (drying brownish perhaps due to glandular exudate), bumpy in texture, poricidal at the tips, the pores ovate, dehiscing distally, not opening into longitudinal slits; pistil with glabrous ovary, the style 7–9 mm long, linear, straight to curved, glabrous, the stigma capitate, slightly bilobed, decurrent down two sides. Fruit a berry, 6–12 mm long, 8–13 mm in diameter, depressed globose, orange to red when mature, glabrous, lacking sclerotic granules. Seeds 20–40 per fruit, 2.5–3 × 2–2.5 mm, flattened, oval in outline, with slightly thickened margin, yellow-orange to brown, the surface reticulum rough with indistinct serpentine pattern and deep luminae.

##### Chromosome number.

Unknown.

##### Distribution and habitat.

**Figure 53.**
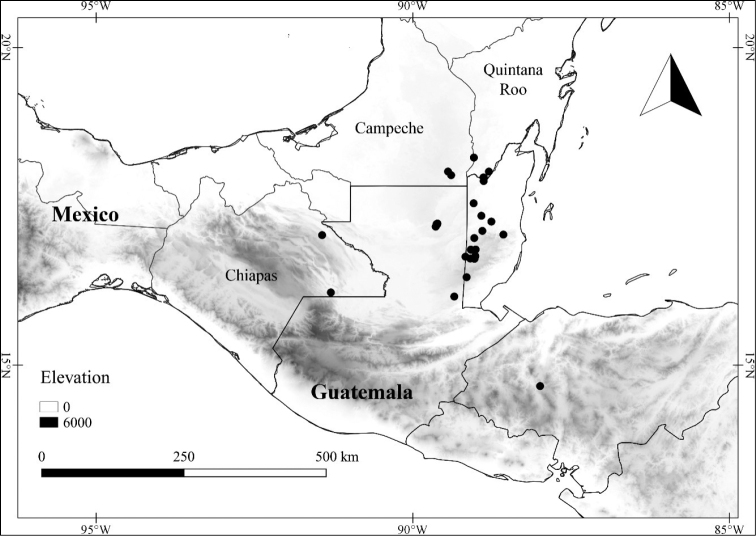
Map of geographic distribution of *L. hypoleuca* based on herbarium specimen data.

Mexico (Campeche, Chiapas, Quintana Roo), Guatemala (Petén), Belize, and Honduras, in primary or disturbed tropical moist forest and tropical dry forest, on slopes and ridges, in ravines, often on limestone, 0–800 m in elevation (Fig. 53).

##### Common names and uses.

Mexico. Chiapas: tumat zak (Tzeltal) (*A. Méndez G. 9067*).

##### Phenology.

Flowering specimens have been collected from May through October; specimens with mature fruits have been collected from June through October. In Belize, corollas open in the early morning (sometimes before sunrise) and close by sunset (Smith and Knapp 2002).

##### Preliminary conservation status.

*Lycianthes hypoleuca* is a species ranging from southern Mexico to Honduras, represented by 33 collections and occurring in four protected areas. The EOO is 78,970.5 km^2^, and the AOO is 124 km^2^. Based on the IUCN (2019) criteria, the preliminary assessment category is Least Concern (LC).

##### Discussion.

*Lycianthes hypoleuca* is a distinctive species of lowland Caribbean forest. It is easily identified based on its leaves. The whitish, tomentose, stellate pubescence of the underside of the leaf surface makes the underside much paler than the upper side. The pollination of this species was studied in Belize by Smith and Knapp (2002) and they found that a number of different bee species visit the flowers.

##### Representative specimens examined.

**Guatemala. Petén**: 2 mi S of entrance of Tikal National Park, [17.2107, -89.6247], 500 ft, 19 Jun 1973, *T.B. Croat 24707* (MO). **Mexico. Campeche**: Mpio. Calakmul, N de Rancho Ek Sacrificio, camino a nuevo centro de población Ejidal Ley de Fomento Agropecuario, 17.9897, -89.3944, 61 m, 5 Aug 1997, *E.M. Martínez S. 28113* (NY). **Chiapas**: al SW de Santo Domingo, [17.0458, -91.4317], 30 Jul 1982, *J.M. Quintanilla 14* (MO). **Quintana Roo**: a 2 km al norte de Estero Franco, sobre la carretera La Unión-Ucum, [17.9512, -88.8769], 20 Aug 1983, *E. Cabrera-C. 5444* (MO, NY).

#### 24 Lycianthes inconspicua

Taxon classificationPlantae

Bitter, Repert. Spec. Nov. Regni Veg. 20: 368, 1924

Fig. 54

Lycianthes storkii C.V.Morton & Standl., Publ. Field Mus. Nat. Hist., Bot. Ser. 18: 1061. 1938. Type: Costa Rica, 0.5 mile south of Santa María, 5700 ft, 8 Aug 1932, *H. Stork 3138* (holotype: F [0072922F, acc. # 672865]).

##### Type.

Guatemala. [Quetzaltenango]: [Volcán] Santa María, [14.7559, -91.5537], Dec 1877, *K. Bernoulli & O. Cario 2373* (holotype: GOET [GOET003447]).

**Figure 54.**
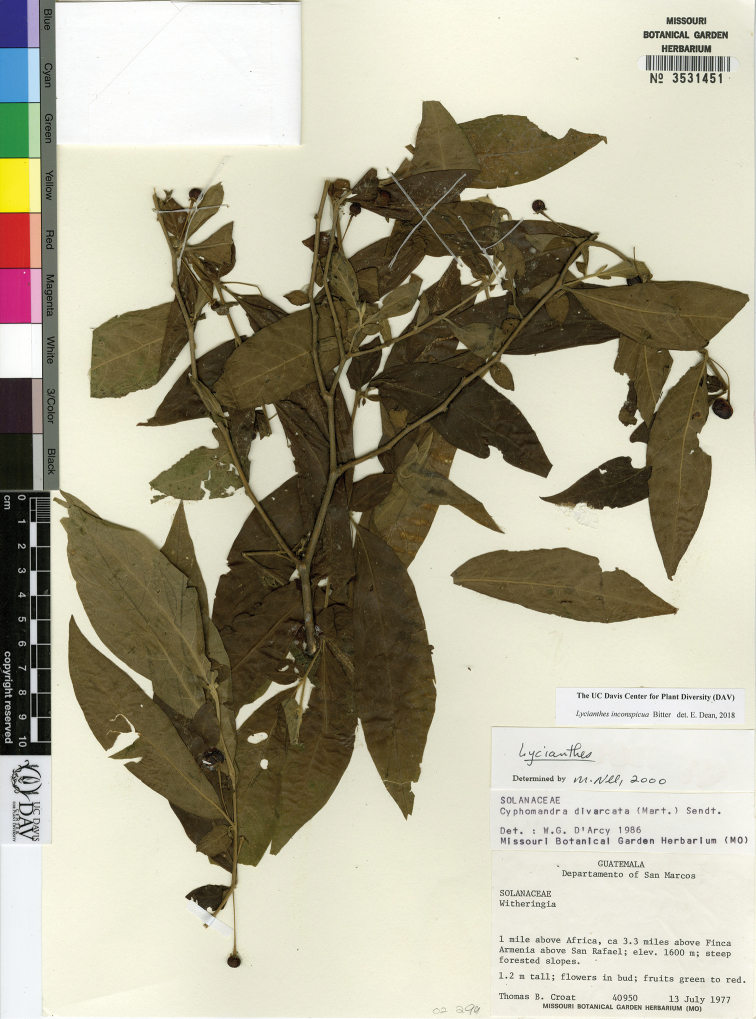
Image of herbarium specimen of *L. inconspicua* in Guatemala, *Croat 40950* (MO). Specimen used with permission from the Missouri Botanical Garden (http://www.tropicos.org).

##### Description.

Subshrub, shrub or treelet, erect, 0.3–6 m tall. Indument pale yellow, uniseriate, multicellular, simple, eglandular, ascending-appressed to ascending trichomes 0.3–1.5 mm long, these usually remaining cylindrical and acute upon drying, the trichomes sometimes becoming less appressed on older stems. Stems green and slender when young, moderately to densely pubescent, sometimes glabrate with age, often compressed upon drying in a plant press, becoming woody with age; upper sympodial branching points usually monochasial with a few dichasial branching points, the branching angles not particularly apparent. Leaves simple, the leaves of the upper sympodia usually paired, usually conspicuously different in size and shape, the larger ones with blades 6–19.5 × 2–7 cm, narrowly elliptic, lanceolate, or oblanceolate, sometimes appearing slightly falcate, the smaller ones with blades 0.6–3.5 × 0.4–2.1 cm, ovate, the blades of both the large and small leaves chartaceous, moderately pubescent, the pubescence densest along the veins of the abaxial side, the trichomes along the midvein of the abaxial side appressed, the base of large blades cuneate, the base of small blades rounded, oblique, the margin entire, usually undulate, the apex acute to acuminate, the petiole to 0.6 (1.1) cm long, sometimes absent, the large leaf blades with (6) 8–12 primary veins on each side of the midvein. Flowers solitary or in groups of 2–3, axillary, nodding; peduncles absent; pedicels very slender, (11) 15–30 mm and straight to arching or deflexed in flower, to 36 mm long, arching or deflexed in fruit, moderately pubescent; 1.5–2.5 mm long, 2–3 mm in diameter, obconic to campanulate, moderately pubescent, the margin truncate, with 5–10 narrow, erect appendages 0.5–3 mm long, emerging ca. 0.25 mm below the calyx rim; fruiting calyx usually enlarged, widely campanulate to bowl-shaped, 1–2 mm long, 3.5–7 mm in diameter, the appendages 1–4 mm long, erect, sometimes withering; corolla 0.5–1 cm long, campanulate to rotate in orientation, stellate in outline, divided 1/2 to nearly all the way to the base, interpetalar tissue present near base, white, adaxial markings unknown, sparsely pubescent abaxially especially near the lobe tips; stamens equal, straight, the filaments 1–2 mm long, glabrous, the anthers 2–3 mm long, ovate, somewhat narrowed at the tip (the narrowed portion ca. 0.25 mm long), free of one another, yellow, glabrous, poricidal at the tips, the pores round, dehiscing distally, not opening into longitudinal slits; pistil with glabrous ovary, the style 3.5–5 mm long, linear, straight, glabrous, the stigma capitate. Fruit a berry, 3.5–9 mm long, 3.5–11 mm in diameter, globose to ovoid, orange to red at maturity, glabrous, lacking sclerotic granules. Seeds 50–100 per fruit, 0.9–1.5 × 1 mm, compressed, but not flat, ridged, rhombic to triangular in outline, tan to orange, the surface reticulum with minute serpentine pattern and shallow luminae.

##### Chromosome number.

Unknown.

##### Distribution and habitat.

**Figure 55.**
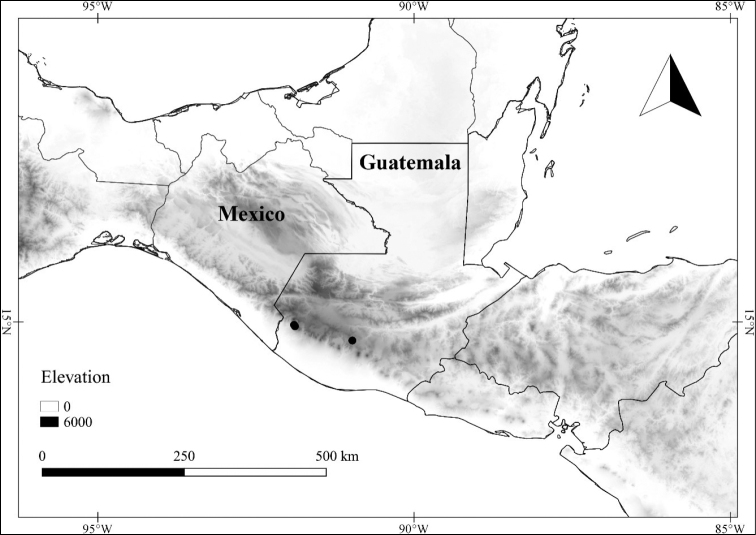
Map of geographic distribution of *L. inconspicua* based on herbarium specimen data.

Guatemala (Quetzaltenango, San Marcos, Suchitepéquez), Costa Rica, and Panama, in cloud forest, montane forest, and oak forest, sometimes on slopes in canyons and drainages, often near streams, 1250–1870 m in elevation (Fig. 55).

##### Common names and uses.

Guatemala. Chiltepe de montaña (Gentry and Standley 1974).

##### Phenology.

Flowering specimens have been collected in January, February, June, and July. Specimens with mature fruits have been collected in July, August, and February. Information about the diurnal movements of the corolla of this species has not been determined; the flowers on specimens range from fully open to somewhat closed (campanulate).

##### Preliminary conservation status.

In Guatemala, *Lycianthes inconspicua* is represented by only three collections, although it is more common in Costa Rica and Panama, and present in one protected area in Panama (Cerros de La Carpintera, Panama). The EOO is 27,148.801 km^2^, and the AOO is 32 km^2^. Based on the IUCN (2019) criteria, the preliminary assessment category for Guatemala is Endangered (EN).

##### Discussion.

*Lycianthes inconspicua* is an uncommon species of high elevation areas in Central America. It is uncommon in Guatemala, but more common in high elevation areas of Costa Rica and Panama. It is morphologically similar to three other Mexican and Central American species: *L. glabripetala*, *L. amatitlanensis* and *L. inaequilatera*. *Lycianthes amatitlanensis* differs from *L. inconspicua* in having shorter pedicels (4–12 mm in flower and 6–16 mm in fruit), long, coarse trichomes that spread away from the midvein on the abaxial side of the leaf (usually with some trichomes at an angle close to ninety degrees), and lanceolate anthers with a longer attenuate portion at the tip (ca. 0.5 mm long). *Lycianthes inaequilatera* and *L. glabripetala* are more similar to *L. inconspicua* in terms of having appressed trichomes along the leaf blade midvein, but they tend to have shorter pedicels (15 mm or less in flower); in addition, *L. glabripetala* has larger corollas (1–1.2 cm long).

##### Representative specimens examined.

**Guatemala. [Quetzaltenango**]: [Volcán] Santa María, [14.7559, -91.5537], Dec 1877, *K. Bernoulli & O. Cario 2373* (GOET). **San Marcos**: Finca Armenia, Rafael de Cuesta, San Marcos, 5000 ft, 6–7 Jul 1977, *J.D. Dwyer 14408* (LL , MO). **Suchitepéquez**: Mpio. San Francisco Zapotitlán, Reserva Natural Privada Las Nubes, 14.7061, -90.9744, 1600 m, 3 Jul 2014, *B. Escobar 171* (BIGU).

#### 25 Lycianthes jalicensis

Taxon classificationPlantae

E.Dean, Novon 8: 133. 1998

Fig. 56

##### Type.

Mexico. Jalisco: S of Puerto Vallarta and N of El Tuito, along hwy. 200, 20.3 road km S of Playa Mismaloya, W side of the road, along footpath that follows small drainage, 500 m, 13 Aug. 1991, *E. Dean 248* (holotype: DAV [DAV158081]; isotypes: IEB [000183677], MEXU [MEXU01195794], NY [00687933], UC [1797878], XAL [XAL0106678]).

**Figure 56.**
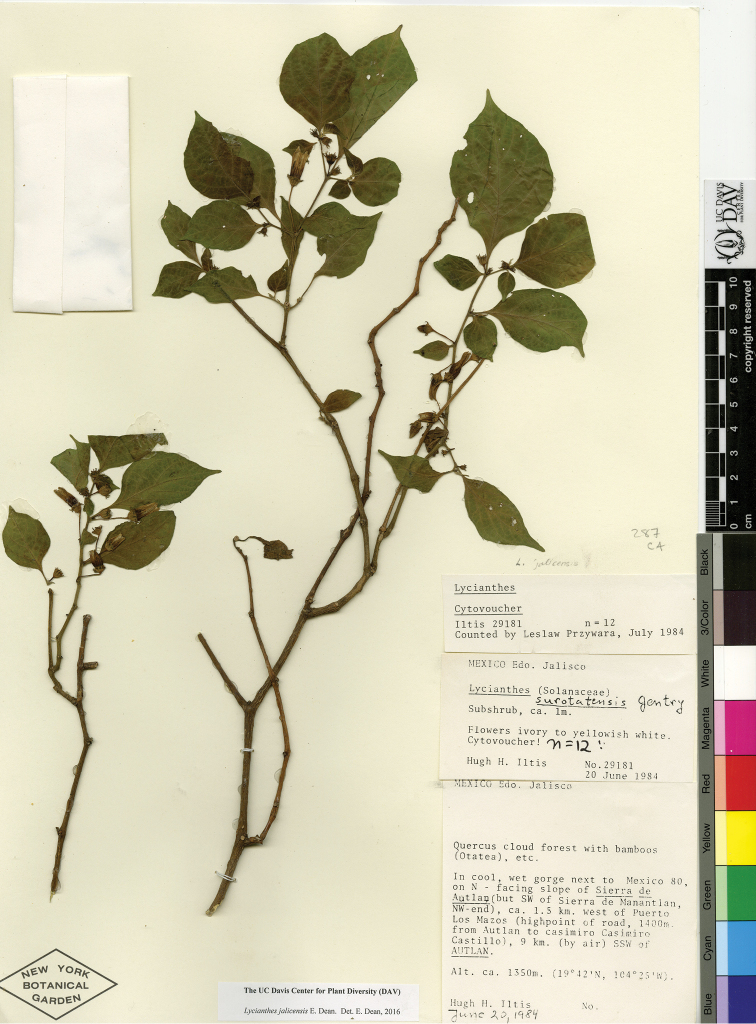
Image of herbarium specimen of *L. jalicensis*, *Iltis 29181* (NY). Specimen used with permission from the William and Lynda Steere Herbarium, New York Botanical Garden.

##### Description.

Shrub, 0.3–2.2 m tall, from horizontal rhizomes. Indument white to tan, uniseriate, multicellular, simple, eglandular, appressed (usually appressed-ascending) trichomes 0.1–1.25 mm long. Stems green to purple (drying greenish tan) with purple (drying blackish) lenticular vertical striations and purplish nodes when young, glabrous to sparsely pubescent, not much compressed when dried in a plant press, becoming brown and woody with age; upper sympodial branching points mostly monochasial, sometimes dichasial. Leaves simple, the leaves of the upper sympodia usually paired and unequal in size, the larger ones with blades 5.5–15 (22) × 2.9–8.7 (11) cm, the smaller ones with blades 2–9 (12) × 1.3–4.9 (7) cm, the leaf pairs similar in shape, the blades ovate, elliptic, or obovate, chartaceous, glabrous to sparsely pubescent, the base cuneate to truncate (rounded on small leaves), sometimes oblique, the margin entire, usually irregularly undulate, the apex acuminate, the petiole 0.1–1.5 (2.5) cm long, the larger leaf blades with 5–7 primary veins on each side of the midvein. Flowers solitary or in groups of 2–7, axillary, oriented horizontally; peduncles absent; pedicels 5–30 mm long and erect in flower, 11–29 mm long and erect in fruit, glabrous (rarely sparsely pubescent); calyx 2.5–4 mm long, 2.5–5.5 mm in diameter, campanulate, glabrous to sparsely puberulent, the margin truncate, with 10 spreading, linear appendages 1–5.5 mm long emerging 0.5–1 mm below calyx rim; fruiting calyx enlarged, widely bowl-shaped to rotate, 1.5–3.5 mm long, (3.5) 5–8 mm in diameter, the appendages to 6 mm long; corolla (1) 1.4–2.3 cm long, campanulate in orientation (sometimes opening wider by tearing), mostly entire in outline (with shallow notches), with abundant interpetalar tissue, adaxially white and glabrous with no markings, the abaxial side of the lobes green, glabrous to sparsely puberulent; stamens unequal, straight, the four short filaments 0.5–2 mm long, the one long filament 4–7 mm long, glabrous, the anthers 4–6 mm long, lanceolate, free of one another, yellow, glabrous, poricidal at the tips, the pores ovate, the pores of the longest stamen dehiscing toward the style, the pores of the shorter stamens usually dehiscing away from the style, not opening into longitudinal slits; pistil with glabrous ovary, the style 6–11 mm long, linear, slightly curved downward, glabrous, the stigma oblong, decurrent down two sides. Fruit a berry, 6–10 mm long, 7–12 mm in diameter, globose, red at maturity, glabrous, lacking sclerotic granules. Seeds 20–40 per fruit, 2–3 × 1.5–2 mm, flattened, depressed ovate in outline, tan to light brown, the surface reticulum with minutely pitted serpentine pattern and shallow luminae.

##### Chromosome number.

n = 12, from *Iltis 29181*, count reported on herbarium label as done by Leslaw Przywara, July 1984, apparently unpublished.

##### Distribution and habitat.

**Figure 57.**
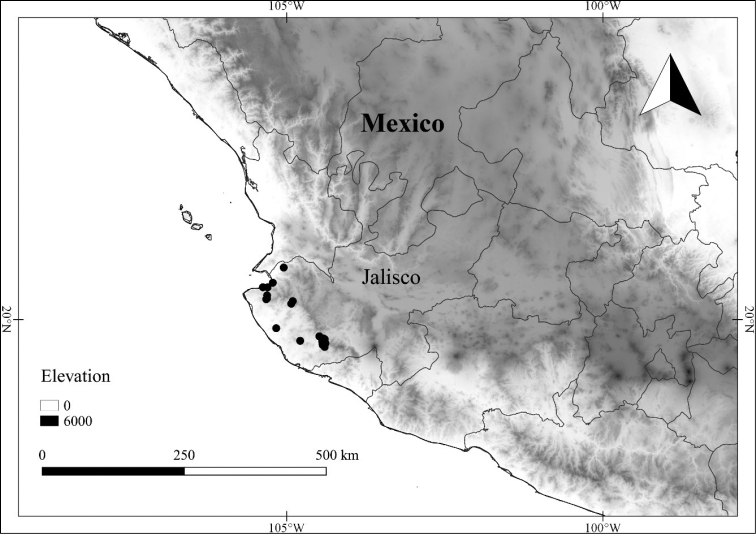
Map of geographic distribution of *L. jalicensis* based on herbarium specimen data.

Mexico (Jalisco) in tropical moist forest, tropical dry forest or in oak forest, often near drainages, 350–1350 m in elevation (Fig. 57).

##### Common names and uses.

None known.

##### Phenology.

Flowering specimens have been collected from June through November (and March); specimens with mature fruits have been collected August through December (and March). Field observation of the corollas indicates that they are open in the early morning and closed by late morning (Dean et al. 2017a).

##### Preliminary conservation status.

*Lycianthes jalicensis* is currently only known from the state of Jalisco, Mexico, represented by 27 collections, only two from protected areas. The EOO is 6,855.367 km^2^, and the AOO is 92 km^2^. Unfortunately, the lands where this species grows are vulnerable due to recent land use changes. Based on the IUCN (2019) criteria, the preliminary assessment category is Vulnerable (VU).

##### Discussion.

*Lycianthes jalicensis* occurs at the low elevations and in habitats that can also be inhabited by the widely distributed *L. arrazolensis*; some populations of *L. arrazolensis* also share the white, campanulate corollas and seeds found in *L. jalicensis*. The two species differ in that *L. jalicensis* has longer pedicels (the length more similar to that of *L. tricolor*), has glabrous calyces and corollas, and usually has larger floral dimensions than *L. arrazolensis. Lycianthes jalicensis* can be distinguished from *L. tricolor* by having unnotched seeds, more glabrous calyces and flowers, and lower elevational range (Dean et al. 2017a).

##### Representative specimen examined.

**Mexico. Jalisco**: streamside bottoms and steep lower erosive wooded slopes of La Calera, a deep narrow valley cut into the SW-facing slope of Sierra de Manantlán Occidental, just NW of km 188 marker on Autlán-Manzanillo hwy (Mex 80), 9 km (by air) NNE of La Resolano (Casimiro Castillo) and ca 16 km SSE of Autlán, [19.6778, -104.4084], 800–1100 m, 10 Mar 1992, *Iltis 31037* (DAV, WIS).

#### 26 Lycianthes limitanea

Taxon classificationPlantae

(Standl.) J.L. Gentry, Phytologia 26: 275. 1973

Fig. 58

Solanum limitaneum Standl., Publ. Carnegie Inst. Wash. 461 (4): 85. 1935. Type: Belize. Camp 33 (on Guatemala-Belize boundary), 2850 feet [869 m], 24 Apr 1934, *W.A. Schipp S-681* (holotype: F [0073115F, acc. # 733642]).

##### Type.

Based on *Solanum limitaneum* Standl.

**Figure 58.**
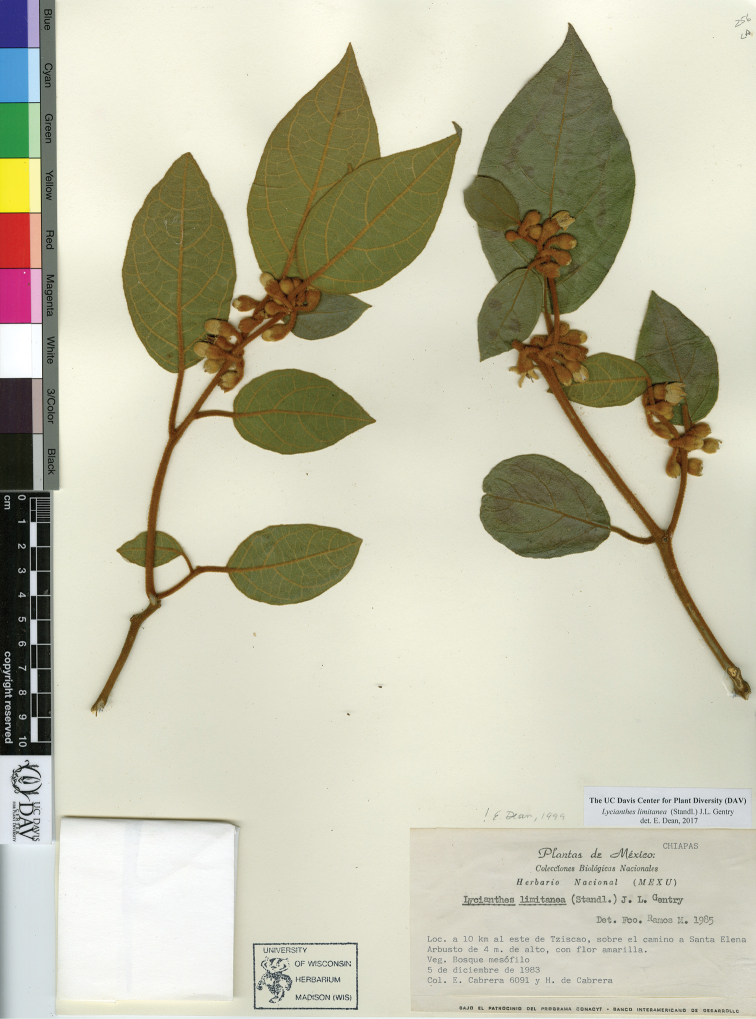
Image of herbarium specimen of *L. limitanea*, *Cabrera 6091* (WIS). Specimen used with permission from Wisconsin State Herbarium, University of Wisconsin, Madison.

##### Description.

Scandent shrub to woody vine, 3–4 (10) m tall, the lower stem to 2.5 cm in diameter. Indument of tan to orange, uniseriate, multicellular, short- to long-stalked, multangulate-stellate to geminate-stellate, eglandular, spreading trichomes 0.5–0.75 (1.2) mm long and in diameter, the rays 5–8 per whorl, straight, rarely rebranched. Stems greenish when young, densely pubescent, not compressed when dried in a plant press, becoming light brown and woody with age; upper sympodial branching usually monochasial, sometimes dichasial. Leaves simple, the leaves of the upper sympodia sometimes paired and unequal in size, the larger ones with blades 8–15.5 × 4.5–10 cm, the smaller ones with blades 1.5–4.5 (9.5) × 1–3 (4) cm, the leaf pairs similar in shape, the blades ovate to elliptic (often widely so), coriaceous, adaxially sparsely pubescent, abaxially densely pubescent, the base truncate to rounded (rarely cuneate or slightly cordate), the margin entire, usually undulate, the apex acute to acuminate, the petiole (0.5) 1–3.7 cm long, the larger leaf blades with 4–6 primary veins on each side of the midvein. Flowers solitary or in groups of 2–12, axillary, nodding; peduncles absent; pedicels 7–12 mm long and reflexed in flower, to 28 mm long and erect in fruit, densely pubescent; calyx 6–7 mm long, 6–7 mm in diameter, campanulate, densely pubescent, the margin truncate, undulate, or lobed, the appendages not present; fruiting calyx enlarged, bowl-shaped, unevenly torn and lobed, 3–10 mm long, 9–17 mm in diameter; corolla 1–1.5 cm long, open corolla orientation not known, entire to shallowly stellate in outline, divided ca. 1/4 of the way to the base, with abundant interpetalar tissue, white, adaxially glabrous, abaxially densely puberulent on the lobes; stamens equal, the filaments ca. 1 mm long, glabrous, the anthers 5–6 mm long, lanceolate, free of one another, yellow, glabrous to sparsely pubescent, poricidal at the tips, the pores ovate, dehiscing distally, not opening into longitudinal slits; pistil with glabrous ovary, the style 8–9 mm long, linear, straight to curved, glabrous, the stigma oblong, sometimes slightly bilobed, decurrent down two sides. Fruit a berry, 8–21 mm long, 7–22 mm in diameter, globose to depressed globose, green to white when immature, orange to red when mature, glabrous to sparsely pubescent, lacking sclerotic granules. Seeds 20–50 per fruit, 2.9–3.8 × 2.5–3.2 mm, flattened, with thickened margin, circular to depressed ovate in outline, yellow-orange to brown-orange, the surface reticulum in the center nearly smooth with indistinct serpentine pattern and shallow luminae, the texture on the margin wrinkled and rough.

##### Chromosome number.

Unknown.

##### Distribution and habitat.

**Figure 59.**
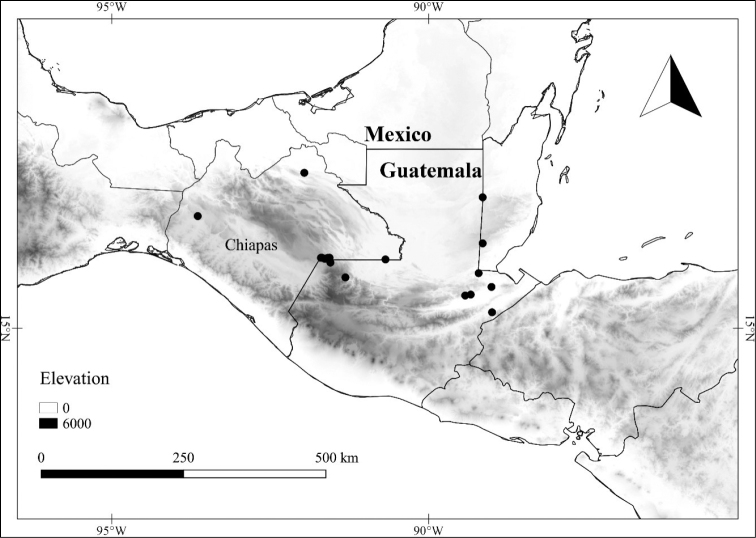
Map of geographic distribution of *L. limitanea* based on herbarium specimen data.

Mexico (Chiapas), Guatemala (Huehuetenango, Izabal, Petén), and Belize, in tropical rain forest, pine forest, pine-oak forest, cloud forest, and tropical moist forest, both in primary forest and along road edges, on slopes and ridges, 80–1500 m in elevation (Fig. 59).

##### Common names and uses.

None known.

##### Phenology.

Flowering specimens have been collected December through May; specimens with mature fruits have been collected from March through October. The diurnal movements of the corolla of this species are unknown. The five flowering collections that we examined had closed flowers, indicating that the flowers must open and close very early in the morning or at night.

##### Preliminary conservation status.

*Lycianthes limitanea* is an uncommon species of southern Mexico, Guatemala, and Belize, represented by 17 collections and occurring in two protected areas (Columbia River, Belize and Río Dulce, Guatemala). The EOO is 69,835.282 km^2^, and the AOO is 68 km^2^. Based on the IUCN (2019) criteria, the preliminary assessment category is Least Concern (LC).

##### Discussion.

*Lycianthes limitanea* is a distinctive, but poorly known and rarely collected, species of the Caribbean slope ranging from southern Mexico to Belize and Guatemala. It is easily identified based on its dense, tan to orange, multangulate-stellate trichomes, relatively large leaves, large calyces which lack appendages, equal stamens, and large, round fruits. Until this paper, the flowers had not been described in the literature, and of the 17 collections we examined, only five collections were in bud and three collections were in flower. All the other collections were in fruit. Therefore, the flower measurements in this description are based on dissection of a single flower.

##### Representative specimens examined.

**Guatemala. Huehuetenango**: Nentón, along the road from Nuevo San José Frontera to Las Palmas, 16.0333, -91.55, 900–1200 m, 17 Mar 2009, *M.J.M. Christenhusz 5619* (NY). **Izabal**: El Estor, La Llorona, 15.5142, -89.4236, 500 m, 30 Aug 1998, *M. Véliz 6652* (BIGU). **Petén**: on Melchor de Mencos Road, 8 May 1967, *E. Contreras 6873* (MO, NY, LL). **Mexico. Chiapas**: Mpio. Palenque, 6–12 km south of Palenque on road to Ocosingo, [17.4468, -91.9623], 300 m, 10 May 1973, *D. Breedlove 35007* (MO).

#### 27 Lycianthes manantlanensis

Taxon classificationPlantae

Aarón Rodr. & O.Vargas, Novon 12: 245. 2002

Fig. 60

##### Type.

Mexico. Jalisco: Mpio. Guautitlán de García Barragán, Majada de las Avellanas, comunidad indígena de Cuzalapa, 3–4 km al NNW de El Durazno, 800–1000 m, 6 Nov 1995, *R. Cuevas 5009* (holotype: IBUG (not seen); isotypes: ENCB (not seen), IBUG [IBUG0157395], MEXU (not seen), MO (not seen), WIS (not seen), ZEA).

**Figure 60.**
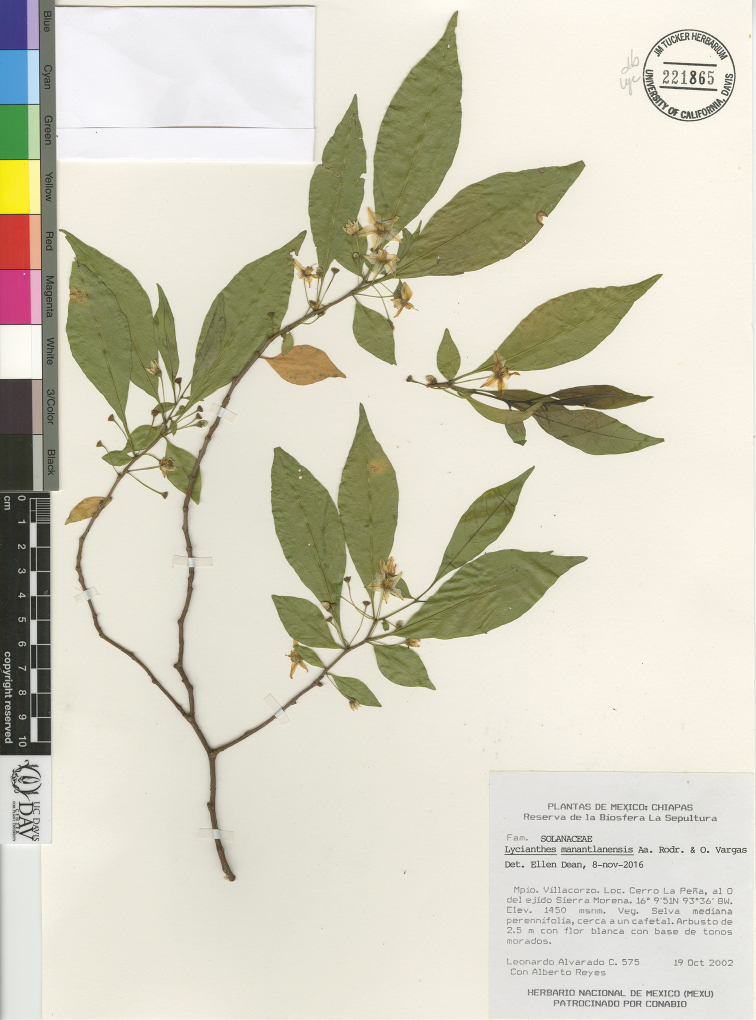
Image of herbarium specimen of *L. manantlanensis*, *Alvarado 575* (DAV). Image used with permission of the UC Davis Center for Plant Diversity.

##### Description.

Shrub to tree, 1.5–7 (10) m tall. Indument mostly lacking, rarely with a few tan to brown, uniseriate, multicellular, simple, eglandular, appressed-ascending trichomes 0.1–0.25 mm long. Stems green when young, usually glabrous, not compressed, but sometimes slightly angled, upon drying in a plant press, woody with age; upper sympodial branching points monochasial or dichasial. Leaves simple, the leaves of the upper sympodia usually paired and unequal in size, the larger ones with blades 3–15 × 0.75–6 cm, the smaller ones with blades 1–6 × 0.3–3 cm, the leaf pairs usually similar in shape, the blades ovate (sometimes narrowly), elliptic, or obovate, coriaceous, glabrous and shiny on both sides, the base cuneate to attenuate, sometimes oblique, the margin entire, usually undulate, the apex obtuse to acute or acuminate, the petiole 0.2–1.5 cm long, the larger leaf blades with 5–8 primary veins on each side of the midvein. Flowers solitary or in groups of 2–5, axillary, oriented horizontally to nodding; peduncles absent; pedicels slender, 14–27 mm long and erect to arching in flower, to 35 mm long, arching to deflexed in fruit, usually glabrous; calyx 2–3.5 mm long, 3–4.5 mm in diameter, campanulate, glabrous or with a few small scattered trichomes, the margin truncate to shallowly lobed, often irregularly notched or torn, with 0–5 knob-like appendages 0.5–1 mm long, emerging 0.25 mm below the calyx rim; fruiting calyx enlarged, widely bowl-shaped, often torn, 1–3 mm long, 3.5–5.5 mm in diameter, the appendages usually not visible; corolla 0.6–1.3 cm long, rotate to campanulate in orientation, stellate in outline, divided 1/2 to nearly all of the way to the base, the lobes with interpetalar tissue, white both abaxially and adaxially, often with yellow-green or purple markings near the stamen insertion on the adaxial side, glabrous except for tiny trichomes along the margins of the lobes; stamens equal to unequal, straight, when unequal the four shorter filaments 1–1.5 mm long, the fifth filament 1.5–2 mm long, glabrous, the anthers 2.5–3 mm long, lanceolate, free of one another, yellow, sometimes with a brown connective, glabrous, poricidal at the tips, the pores round, large, dehiscing distally, not opening into longitudinal slits; pistil with glabrous ovary, the style 6–7 mm long, linear, straight, glabrous, the stigma capitate. Fruit a berry, 5–12 mm long, 5–13 mm in diameter, globose to depressed globose, green when immature, purple at maturity, glabrous, lacking sclerotic granules. Seeds 10–50 per fruit, 2.5–3 × 2–2.5 mm, flattened, depressed ovate to circular in outline, sometimes folded, brown, the surface reticulum with minute serpentine pattern and shallow luminae.

##### Chromosome number.

Unknown.

##### Distribution and habitat.

**Figure 61.**
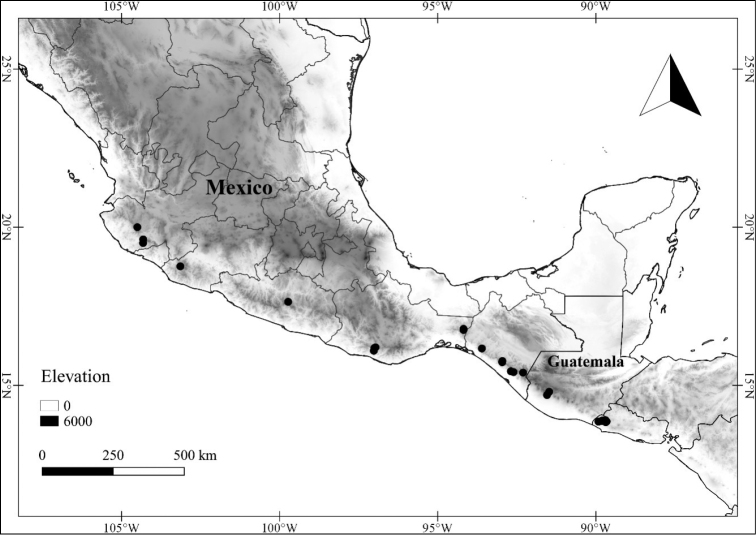
Map of geographic distribution of *L. manantlanensis* based on herbarium specimen data.

Mexico (Chiapas, Guerrero, Jalisco, Michoacán, Oaxaca), Guatemala (Quetzaltenango), and El Salvador, in primary or secondary forest, often in the transition between tropical dry forest and cloud forest, including tropical moist forest, *Liquidambar*, oak, or pine-oak forest, rarely in fir forest, in canyons or on slopes, 1200–2500 m in elevation (Fig. 61).

##### Common names and uses.

Mexico. Naranjillo (Rodríguez and Vargas 2002). El Salvador. Chiltepe morado (*Reyna 1492*).

##### Phenology.

Flowering specimens have been collected June through December. Specimens with mature fruits have been collected December through June. The corollas on the specimens are often open; this indicates that the corollas must be open for a substantial amount of time each day.

##### Preliminary conservation status.

*Lycianthes manantlanensis* is a widespread species ranging from western Mexico to El Salvador, represented by 29 collections and occurring in three Mexican protected areas (Sierra Manantlán, La Sepultura, and El Triunfo). The EOO is 198,390.562 km^2^, and the AOO is 104 km^2^. Based on the IUCN (2019) criteria, the preliminary assessment category is Least Concern (LC).

##### Discussion.

*Lycianthes manantlanensis* is sometimes confused with *L. orogenes* and *L. barbatula*, and it is probable that the three are closely related, because they share similarities in floral structure, such as white, stellate corollas and yellow anthers that sometimes have a brownish connective. *Lycianthes barbatula* differs from the other two species in having tufts of trichomes in the vein axils on the abaxial leaf side. *Lycianthes manantlanensis* differs from *L. orogenes* in having a calyx that nearly lacks calyx appendages and often tears in fruit, as well as having equal to slightly unequal stamens. It should be noted that the original description of *L. manantlanensis* incorrectly stated that the mature fruits are green (Rodríguez and Vargas 2002). The mature fruits are purple; the immature fruits are green. In addition, the original description states that the stamens are equal. However, the stamens are not always equal; they are often slightly unequal, with one stamen slightly longer than the other four. In the preparation of this treatment, we realized that this species is not endemic to Mexico but ranges to El Salvador. The specimens collected in El Salvador and Guatemala had been identified originally as *L. orogenes*, and many of the specimens were collections made by Standley or Steyermark, the original authors of that species. However, examination of the type material of *L. manantlanensis* and *L. orogenes* shows differences in the calyx structure that allows us to assign some of the Guatemalan and all of the El Salvadorian material to *L. manantlanensis*.

##### Representative specimens examined.

**Guatemala. Quezaltenango**: along road between Finca Pirineos and Patzulín, 1200–1400 m, 9 Feb 1941, *P.C. Standley 87013* (US). **Mexico. Chiapas**: 5 km del camino Ejido Las Golondrinas a Rosario Sacatonal, 15.4436, -92.6856, 1400 m, 9 Mar 2006, *R. Martínez-Camilo 936* (MO). **Guerrero**: 33.3 km SW of Filo de Caballo, on Chilpancingo-Atoyac road, [17.6405, -99.7304], 2000 m, 7 Nov 1999, *T. Yahara 1925*, (MEXU). **Jalisco**: Mpio. Cuautitlán de García Barragán, Majada de las Avellanas, comunidad indígena de Cuzalapa, 3–4 km al NNW de El Durazno, [19.4988, -104.3182], 800–1000 m, 6 Nov 1995, *R. Cuevas 5009* (IBUG). **Michoacán**: Dto. Coalcoman, Naranjillo, [18.7624, -103.1397], 1400 m, 2 Aug 1941, *Hinton 15942* (F, NY). **Oaxaca**: cañada al N de Cerro de la Leona (cerro al NE de Cerro Quetzal y ca. 7–9 km al N de Cerro Guayabitos), ca. 46 km en línea recta al N de San Pedro Tapantepec, 16.7833, -94.1833, 1300 m, 28 Feb 1987 *S. Maya J. 4226* (MEXU).

#### 28 Lycianthes mariovelizii

Taxon classificationPlantae

E.Dean, Brittonia 70: 482. 2018

Fig. 62

##### Type.

Guatemala. Huehuetenango: Mpio. Santa Ana Huista, N of Parque Victoria aquatic center, Aldea El Tabacal, bank above the Río Santa Ana, upstream of the sumidero, 15.6949, -91.8721, 753 m, 15 Aug 2017, *E. Dean 9509* (holotype: BIGU [acc. # 76960]; isotypes: BIGU [acc. # 76959], DAV [acc. # 221941, acc. # 221937], MEXU, NY).

**Figure 62.**
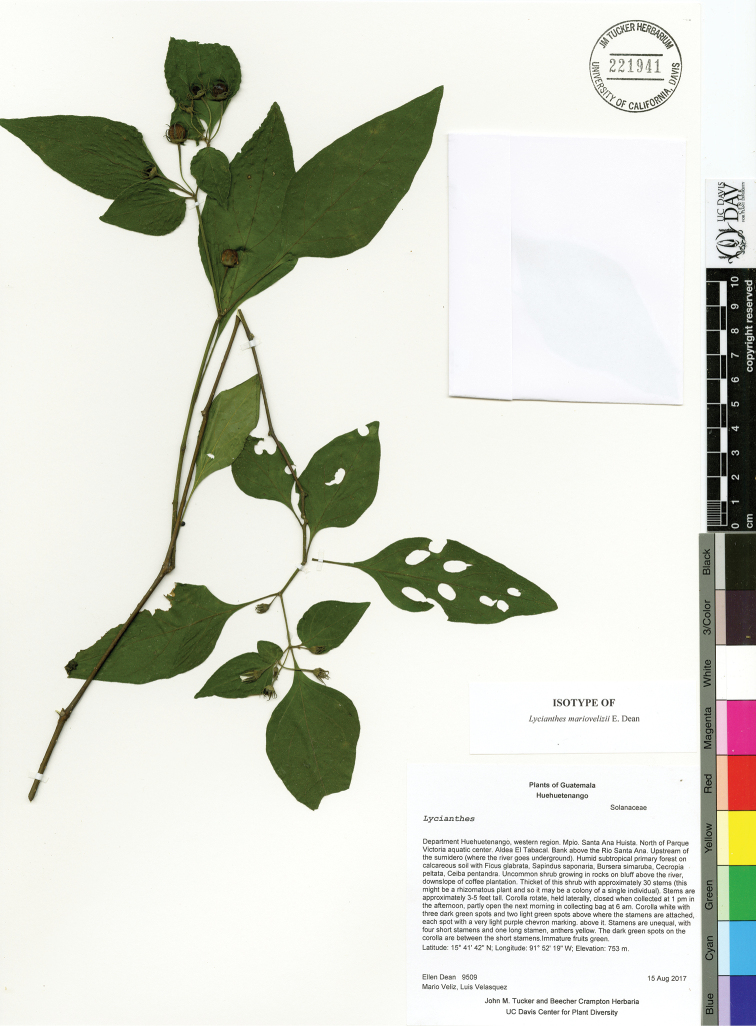
Image of isotype of *L. mariovelizii*, *Dean 9509* (DAV). Image used with permission of the UC Davis Center for Plant Diversity.

##### Description.

Shrub, 0.75–2 m tall, erect. Indument of light yellow (sometimes appearing tan or off-white), uniseriate, multicellular, simple, eglandular, appressed-ascending trichomes 0.1–1 (1.5) mm long. Stems green with small light green lenticular vertical striations when young, sparsely to moderately pubescent, not much compressed when dried in a plant press, becoming brown and woody with age; upper sympodial branching points monochasial or dichasial. Leaves simple, the leaves of the upper sympodia usually paired and unequal in size, the larger ones with blades 5.5–14 × 2–7 cm, the smaller ones with blades 2–7 × 1–4.5 cm, the leaf pairs similar in shape, the blades ovate to elliptic, chartaceous, glabrous to sparsely (moderately) pubescent, the trichomes usually densely spreading outward (towards the margins) along the abaxial veins, especially at the base of the main vein, the base cuneate to attenuate, sometimes oblique, the margin entire, usually irregularly undulate, the apex acuminate, the petiole 0.2–1.5 cm long, sometimes absent, the larger leaf blades with 5–6 primary veins on each side of the midvein. Flowers solitary or in groups of 2–3, axillary, oriented horizontally; peduncles absent; pedicels (8) 10–18 mm long and erect in flower, 15–25 mm long and erect in fruit, sparsely to moderately pubescent; calyx 2–3 mm long, 4–5 mm in diameter, obconic to campanulate, sparsely to moderately pubescent, the margin truncate, with 10 spreading linear appendages 5–11 mm long (appendages on the same calyx of varying lengths, but at least some appendages on the same calyx > 7 mm long), the base of the appendages flattened and 0.5–1 mm wide, emerging ca. 0.25–0.5 mm below the calyx rim; fruiting calyx enlarged, bowl-shaped to rotate, 2–3 mm long, 5–7 mm in diameter, the appendages 5–14 mm long, to 1.5 mm wide at the flattened base; corolla 0.9–1.8 cm long, rotate in orientation, mostly entire in outline (with shallow notches), with abundant interpetalar tissue, white, the adaxial side with three green spots located between the short stamens, glabrous, the abaxial side of the lobes green, sparsely to moderately puberulent near the major veins; stamens unequal, straight, the four short filaments 1–2 mm long, the one long filament 3–5 mm long, glabrous, the anthers 4–6 mm long, lanceolate, free of one another, yellow, glabrous, poricidal at the tips, the pores ovate, the pores of the longest stamen dehiscing toward the style, the pores of the shorter stamens dehiscing distally, not opening into longitudinal slits; pistil with glabrous ovary, the style 8–9 mm long, linear, glabrous, the stigma oblong, decurrent down two sides, slightly lobed. Fruit a berry, 10–11 mm long, 10–12 mm in diameter, globose to depressed globose, orange (red) at maturity, glabrous, lacking sclerotic granules. Seeds 10–40 per fruit, 2.5–3 × 2–2.5 mm, flattened, nearly circular to oval in outline, not obviously notched (if slightly indented, indentation is usually less than 0.3 mm), yellow-orange, the surface reticulum with indistinct serpentine pattern and shallow luminae.

##### Chromosome number.

Unknown.

##### Distribution and habitat.

**Figure 63.**
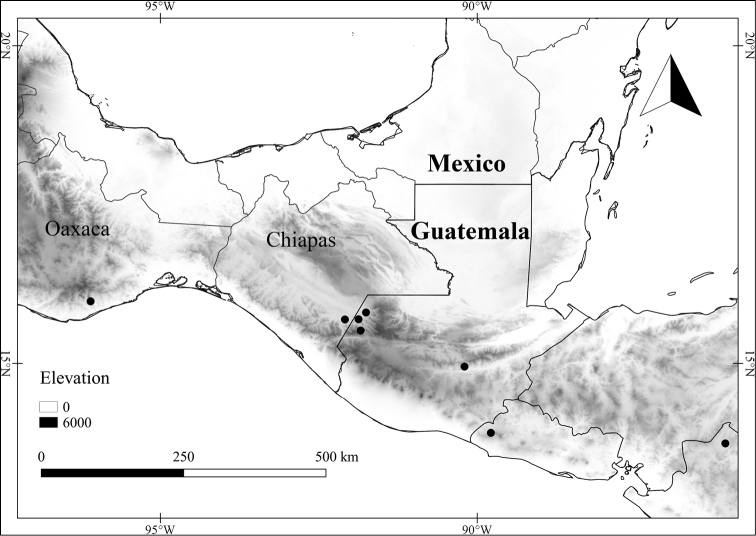
Map of geographic distribution of *L. mariovelizii* based on herbarium specimen data.

Mexico (Chiapas, Oaxaca), Guatemala (Huehuetenango, El Progreso), El Salvador, and Nicaragua, in oak or oak-pine forest, tropical moist forest, tropical dry forest, often on calcareous soils, sometimes near drainages, 700–1000 m in elevation (1600 m in El Salvador) (Fig. 63).

##### Common names and uses.

None known.

##### Phenology.

Flowering specimens have been collected from July through November; specimens with mature fruits have been collected September through December. The corollas of this species are open in the early morning and closed by late morning (Dean et al. 2018c).

##### Preliminary conservation status.

*Lycianthes mariovelizii* is a widespread but poorly collected species ranging from southern Mexico to Nicaragua, represented by only nine collections and occurring in eight protected areas. The EOO is 87,389.609 km^2^, and the AOO is 32 km^2^. Based on the IUCN (2019) criteria, the preliminary assessment category is Least Concern (LC).

##### Discussion.

*Lycianthes mariovelizii* belongs to series *Tricolores* and is distinguished from most of the other species in the series by long calyx appendages (to 11 mm in flower and 14 mm in fruit) that are somewhat flattened and relatively wide at the base (appearing very flattened when dried). Within a calyx, the appendages often vary in length, but at least some of the appendages on a calyx are greater than 7 mm long and 1 mm wide at the base. The only species in series *Tricolores* with appendages similarly long and wide is *L. surotatensis*, a species with glandular trichomes (Dean et al. 2018c). Within the series, *L. mariovelizii* is most similar to *L. arrazolensis*, *L. jalicensis*, and *L. surotatensis*, three species with which *L. mariovelizii* shares the following characters: unnotched seeds, white corollas, and occurrence mostly below 1000 m. *Lycianthes mariovelizii* differs from *L. surotatensis* by eglandular trichomes. It differs from *L. arrazolensis* and *L. jalicensis* by longer calyx appendages inserted < 0.5 mm below the calyx rim, versus > 0.5 mm. The appendages of *L. arrazolensis* are typically < 2.5 mm long, rarely reaching 5 mm in Guerrero; those of *L. jalicensis* are typically < 5 mm long. Also, the calyx and corolla of *L. jalicensis* are usually glabrous or nearly so, whereas those of *L. mariovelizii* are pubescent (Dean et al. 2018c). A collection of *L. mariovelizii* (*Steyermark 51177*) was listed by Gentry and Standley (1974) as a difficult to place collection and unnamed taxon at the end of their treatment of *Lycianthes* in Flora of Guatemala; Dean et al. (2017a) listed a different specimen of *L. mariovelizii* in their Specimen Group E in their section on difficult to place collections at the end of their paper on series *Tricolores* (*E. M. Martínez S. 23237* – listed in the paper as *Droege 23237*).

##### Representative specimens examined.

**Guatemala. Huehuetenango**: Paso del Boquerón, Río Trapichillo, below La Libertad [15.5174, 91.8385], 1200–1300 m, 21 Aug 1942, *J.A. Steyermark 51177* (F); **El Progreso**: a 2 km al N de Los Leones, camino El Rancho-Cobán, 14.95, -90.2, 4 Aug 1988, *E.M. Martínez S. 23237* (MEXU, MO, NY). **Mexico. Chiapas**: Mpio. Frontera Comalapa, 6–8 km E of Frontera Comalapa along road to Ciudad Cuahtémoc, [15.6916, -92.0871], 1000 m, 15 Aug 1972, *D. Breedlove 27009* (CAS, MEXU, MO). **Oaxaca**: Dto. Pochutla, Mpio. San Miguel del Puerto, en el Cafetal Arroyo Arena, 15.9778, -96.1006, 700 m, 16 Nov 2003, *A. Nava-Zafra 205* (DAV).

#### 29 Lycianthes michaelneei

Taxon classificationPlantae

E.Dean, Phytoneuron 2014–42: 4 (2 Apr 2014)

Fig. 64

##### Type.

Mexico. Veracruz: Mpio. Calcahualco: 4.2 km W of Escola on road to Jacal, 17.5 km by road NW of Coscomatepec, 2,200 m, 12 Jan 1981, *M. H. Nee & G. Schatz 19791* (holotype: WIS; isotypes: CAS [483750, acc. # 648091, MEXU [acc. # 303241]).

**Figure 64.**
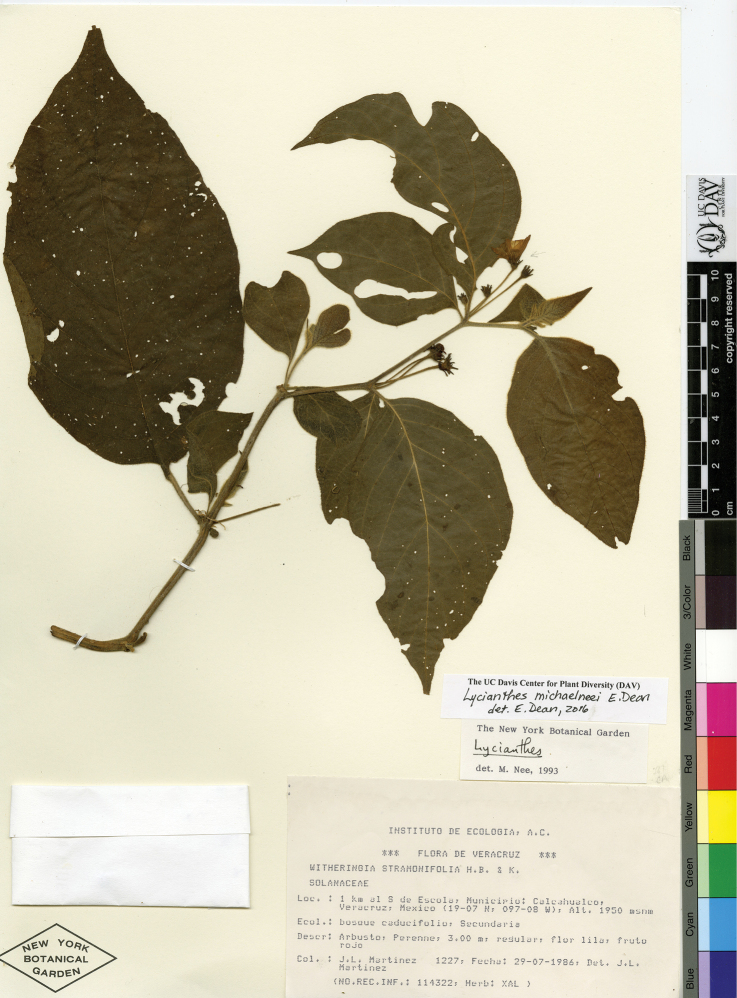
Image of herbarium specimen of *L. michaelneei*, *Martinez 1227* (NY). Specimen used with permission from the William and Lynda Steere Herbarium, New York Botanical Garden.

##### Description.

Shrub, 2–4 m tall. Indument of light yellow, uniseriate, multicellular, simple, eglandular, spreading to appressed, weak, sometimes matted (on stem) trichomes, 0.25–2 mm long. Stems tan to purplish with vertical ridges when young (dark striations not evident on dried material), moderately to densely pubescent, not compressed when dried in a plant press, becoming brown and woody with age; upper sympodial branching points mostly monochasial, sometimes dichasial. Leaves simple, the leaves of the upper sympodia usually paired and unequal in size, the larger ones with blades 13.9–23.5 × 5.5–10.5 cm, the smaller ones with blades 5.7–8.5 × 2.6–4.7 cm, the leaf pairs similar in shape, ovate, elliptic, or obovate, chartaceous, densely pubescent, the base rounded to cuneate, sometimes oblique, the margin entire, usually undulate, the apex acuminate, the petiole 0.2–3.5 cm long, the larger leaf blades with 5–7 primary veins on each side of the midvein. Flowers solitary or in groups of 2–6 (10), axillary, oriented horizontally; peduncles absent; pedicels 15–28 mm long and erect in flower, 26–42 mm long and erect in fruit, densely pubescent; calyx 2–3 mm long, 2.5–3.5 mm in diameter, urceolate to campanulate, densely pubescent, the margin truncate, with 10 spreading, linear appendages 1–4 mm long emerging ca. 0.5 mm below the calyx rim; fruiting calyx enlarged, widely bowl- to plate-shaped, 1.5–3 mm long, 5.5–9 mm in diameter, the appendages to 6 mm long; corolla 1.1–1.6 cm long mm long, rotate in orientation, mostly entire in outline (with shallow notches), with abundant interpetalar tissue, purple adaxially, greenish-purple and densely pubescent near the major veins abaxially; stamens unequal, straight, the four short filaments 0.5–1 mm long, the one long filament 3.5–4 mm long, glabrous, the anthers 3–4 mm long, lanceolate, free of one another, yellow, glabrous, poricidal at the tips, the pores ovate, the pores of the longest stamen dehiscing toward the style, the pores of the short stamens dehiscing away from the style, not opening into longitudinal slits; pistil with glabrous ovary, the style 7–8.5 mm long, linear, straight to slightly curved, glabrous, the stigma capitate, unlobed. Fruit a berry, 6–9 mm long, 6–8 mm in diameter, globose, red at maturity, glabrous, lacking sclerotic granules. Seeds 20–30 per fruit, 2–3 × 2–2.5 mm, flattened, reniform in outline with notch on one side, brown to orange, the surface reticulum with minute serpentine pattern and shallow luminae.

##### Chromosome number.

Unknown.

##### Distribution and habitat.

**Figure 65.**
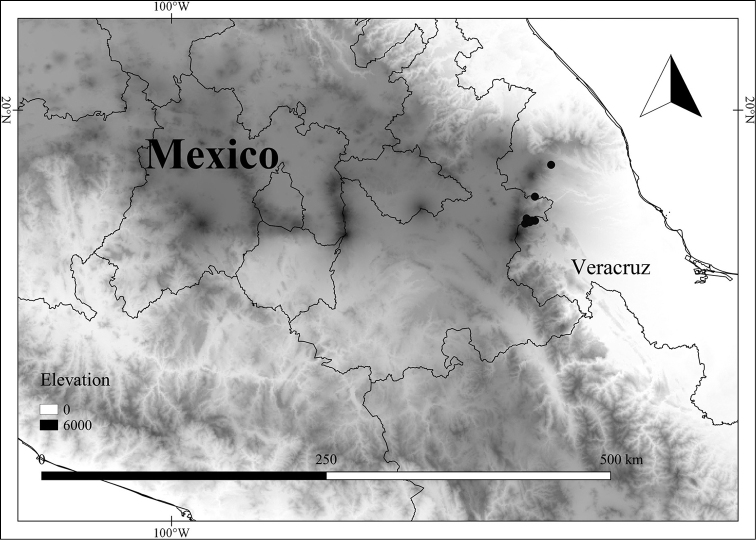
Map of geographic distribution of *L. michaelneei* based on herbarium specimen data.

Mexico (Veracruz), in oak-pine forest, tropical moist forest, or cloud forest, often in disturbed or secondary forest, in shady canyons and ravines, 1750–2600 m in elevation (Fig. 65).

##### Common names and uses.

None known.

##### Phenology.

Flowering specimens have been collected from January to July; specimens with mature fruits have been collected June to January. The diurnal corolla movements of this species are not known. The corollas on specimens are usually closed, indicating that the flowers are probably only open in the early morning.

##### Preliminary conservation status.

*Lycianthes michaelneei* is a rarely collected species of eastern Mexico, represented by only six collections, only one from a protected area (Pico de Orizaba). The EOO is 181.413 km^2^, and the AOO is 24 km^2^. Based on the IUCN (2019) criteria, the preliminary assessment category is Endangered (EN).

##### Discussion.

*Lycianthes michaelneei* is a member of series *Tricolores* (Dean et al. 2017a) and was provisionally identified as *L. pilosissima* (M.Martens & Galeotti) Bitter (a synonym of *L. tricolor*) in the *Flora of Veracruz* (Nee 1986). *Lycianthes michaelneei* is similar to *L. tricolor* in having notched seeds. It differs from *L. tricolor* in having an entirely purple corolla (lobes and membrane) rather than a white membrane with some purple on the lobes, and it may lack the green, glandular spots on the corolla (although more fieldwork is needed to determine this). In general, this species is more robust than similar species such as *L. tricolor* and *L. arrazolensis*. Another distinctive difference is the matted stem trichomes, which are not found in closely related species (Dean et al. 2017a).

##### Representative specimen examined.

**Mexico. Veracruz**: Mpio. Calcahualco, 1 km al S de Escola, 19.1167, -97.1333, 1950 m, 24 Jul 1986, *J. L. Martínez 1227* (NY, XAL).

#### 30 Lycianthes moziniana

Taxon classificationPlantae

(Dunal) Bitter, Abh. Naturwiss. Verein Bremen 24 [preprint]: 408, 1919

Solanum mozinianum Dunal, Encycl. [J. Lamarck & al.] Suppl. 3: 757. 1814. Type: Painting made during the Royal Botanical Expedition to New Spain (1787–1803) under the direction of Martín de Sessé y Lacasta (lectotype designated by Dean 1997, pg. 193: Hunt Institute for Botanical Documentation, catalogue number 6331.0121).

##### Type.

Based on *Solanum mozinianum* Dunal.

#### 30a Lycianthes moziniana var. moziniana

Taxon classificationPlantae

Fig. 66

Solanum uniflorum Sessé ex Lag., Gen. Sp. Pl. [Lagasca]: 10. 1816. Type: Painting made during the Royal Botanical Expedition to New Spain (1787–1803) under the direction of Martín de Sessé y Lacasta (lectotype designated by Dean 1997, pg. 196: Hunt Institute for Botanical Documentation, catalogue number 6331.0025).
Solanum monanthum Roem. & Schult., Syst. Veg., ed. 15 bis [Roemer & Schultes]. 4: 608. 1819. Type: Based on Solanum uniflorum Sessé ex Lag.
Solanum mocinianum Dunal forma luteiflorum Dunal, Prodr. [A. P. de Candolle] 13(1): 164. 1852. Type: Based on Solanum uniflorum Sessé ex Lag.
Solanum uniflorum Moçiño & Sessé, Pl. Nov. Hisp.: 35. 1888. Type: Based on Solanum uniflorum Sessé ex Lag.

##### Description.

Perennial herb from fusiform storage roots, decumbent, ascending, to erect, usually recumbent with age, ca. 0.1–0.5 (0.9) m tall, dying back each season far beneath the soil surface. Indument of white, uniseriate, multicellular, simple, eglandular, spreading to appressed trichomes (0.1) 0.5–2 mm long, (in the state of Michoacán, these sometimes intermixed with forked or dendritically branched trichomes). Stems green to purple-green, moderately pubescent (rarely glabrate in age), compressed and ribbed when dried in a plant press, usually with little woody tissue; first stem (2) 5–35 (40) cm long to the first inflorescence, the internodes (3) 6–14 (21); first sympodial branching point usually dichasial, the subsequent sympodial branching points usually monochasial. Leaves simple, those of the upper sympodia usually paired and unequal in size, the larger ones with blades 2–10.5 × 0.8–4.2 cm, the smaller ones with blades 1/4–7/8 the size of the larger, the leaf pairs similar in shape, the blades obovate, elliptic, ovate, or lanceolate, chartaceous, moderately pubescent, the primary veins 5–7 on either side of the midvein, the base rounded to cuneate, the margin entire, usually undulate to irregularly angled, the apex rounded to acute, the petioles 0.1–0.5 cm long, sometimes absent. Flowers solitary, axillary, oriented horizontally; peduncles absent; pedicels (30) 50–150 (180) mm and erect in flower, 54–170 mm long (probably longer) and deflexed in fruit, moderately pubescent with trichomes of two distinct lengths, the smaller 0.1–0.3 mm and appressed, the longer 0.5–1.5 mm and mostly spreading, rarely only the longer trichomes present; calyx 3–5.5 mm long, 3.5–7 mm in diameter, campanulate, densely pubescent, the margin truncate, with 10 linear, lax appendages laying closely against the corolla 2–10 mm long emerging ca. 0.5–1 mm below the calyx rim; fruiting calyx enlarged, 4–11 mm long, 9–22.5 mm in diameter, the appendages appressed to fruit, often broken, to 11.5 mm long; corolla 1.3–3.6 cm long (2.9–6.8 cm in diameter), rotate in orientation, mostly entire in outline (with shallow notches), with abundant interpetalar tissue, lilac to dark purple (very rarely white or very pale lilac), with darker purple stripes along the major veins adaxially, green and moderately pubescent near the major veins abaxially; stamens unequal, straight, the filaments of three lengths, the two shortest filaments 1–4.5 mm long, the two medium filaments 1.5–5.5 mm long, the one long filament (2.5) 3–8.25 mm long, the length of the longest filament 1.2–2 times that of the medium filaments, glabrous, the anthers 4.5–8.5 mm long, elliptic to ovate, free of one another, yellow, glabrous, poricidal at the tips, the pores obovate, dehiscing distally or toward the style, not opening into longitudinal slits; pollen grains tricolporate; pistil with glabrous ovary, the style 8–14.5 mm, linear, straight to slightly curved, glabrous, the stigma round to slightly bilobed. Fruit a berry, remaining attached to calyx at maturity, pendent or lying on the ground, 14–41.5 mm long, 12–28 mm diameter, round to ovoid, the exocarp green, glabrous, the mesocarp area green, soft and juicy, lacking sclerotic granules, placental area green, soft and juicy. Seeds (10) 40–139 per fruit, 2.3–2.8 × 1.7–2.5 mm, rounded, slightly compressed, reniform to depressed-obovate brownish-black, the surface reticulum with minute serpentine pattern and shallow luminae.

**Figure 66.**
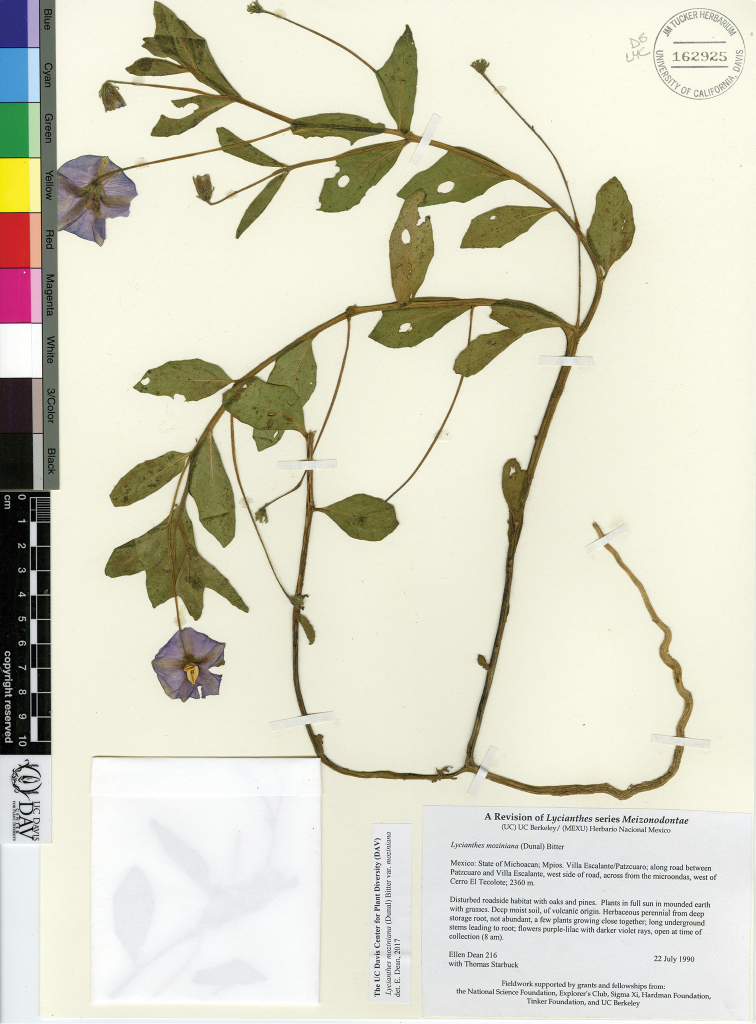
Image of herbarium specimen of L. moziniana var. moziniana, *Dean 216* (DAV). Image used with permission of the UC Davis Center for Plant Diversity.

##### Chromosome number.

2n = 24 (Williams 1993); 2n = 24, *Dean 300, 306* (Dean 2004).

##### Distribution and habitat.

**Figure 67.**
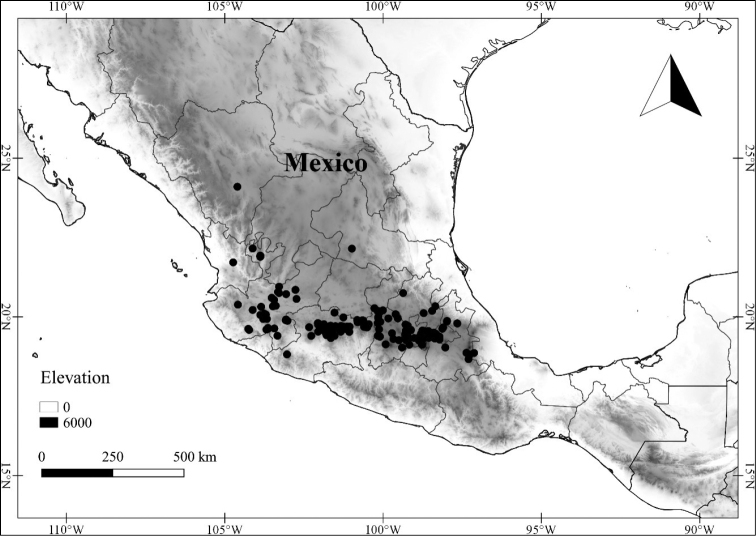
Map of geographic distribution of L. moziniana var. moziniana based on herbarium specimen data.

Mexico (Distrito Federal, Durango, Guanajato, Hidalgo, Jalisco, México, Michoacán, Nayarit, Puebla, Querétaro, San Luis Potosí, Tlaxcala, Veracruz, perhaps Zacatecas), mainly restricted to the volcanic soils of the transvolcanic belt in disturbed areas such as pastures, agricultural fields, along paths and roadsides, and in clearings in xerophilous schrub, oak and coniferous forest, 1600–3000 m in elevation (Fig. 67).

##### Common names and uses.

Mexico. Berenjena, berenjito, chumpin, chimpina, huevo de gato, tintolón, tilindon, tlanoxtle, tlanochtle, tlalnonochtle, shimpe, tilapó, tochin, la chichi, chochocuero, coyotomate, purga de las animas, xipes, mazatlatlaixtli (Dean 2004). Used medicinally as a purgative in the state of México (*Altimirano 72*).

##### Phenology.

Flowering specimens have been collected June to October; specimens with mature fruit have been collected September to December. The first author observed in the field that the corollas open in the very early morning and close by noon. The pollen of this variety has a sweet scent. Solitary bees in the genus *Thygator* visit this species (Dean 2001).

##### Preliminary conservation status.

Lycianthes moziniana var. moziniana is a common variety of the Trans-Mexican Volcanic Belt of Mexico, represented by 181 collections and occurring in 12 Mexican protected areas. The EOO is 264,912.814 km^2^, and the AOO is 688 km^2^. Based on the IUCN (2019) criteria, the preliminary assessment category is Least Concern (LC).

##### Discussion.

Lycianthes moziniana var. moziniana is separated from closely related taxa by its combination of green fruits, small smooth seeds (without fibrils on cell walls), a tendency toward having only one dichasial or no dichasial branching points, very elongate pedicels, and abaxially pubescent corolla lobes. Variety *moziniana* is separated from the other varieties of the species in having fruiting calyx teeth that are lax in flower and stay appressed to the fruit, cuneate (rather than attenuate) leaf bases, relatively dense and long stem and leaf pubescence, consistently pubescent abaxial corolla lobes, and an affinity for the soils of the transvolcanic belt (Dean 2004).

This “weedy” variety has had an intimate relationship with the people of Mexico and may owe its current distribution to humans, who are probably its primary dispersal agents. The fruit of L. moziniana var. moziniana is edible and in past decades was gathered for sale at markets. Some researchers believe this variety was once a domesticated plant that has since reverted to a weed of agricultural areas (Williams 1993). It persists under traditional agricultural practices by sprouting from its underground root system as well as from seed dispersed by humans (Williams 1993). With the advent of herbicide usage in agricultural fields, changes in field preparation techniques, and lack of usage by humans, this once plentiful variety is becoming rare in Mexico (Dean 2004).

In some areas of Jalisco and Michoacán, where this variety grows with *L. rzedowskii* or *L. acapulcensis*, plants with intermediate trichome, leaf and floral color characteristics have been collected or observed. Limited crossing experiments indicate that L. moziniana var. moziniana is capable of crossing with L. moziniana var. oaxacana, although with reduced fertility; crosses between this variety and mature accessions of L. moziniana var. margaretiana have not yet been performed (Dean 2004).

##### Representative specimens examined.

**Mexico. Distrito Federal**: Limbo, delegación de Álvaro Obregón, [19.3247, -99.2633], 2700 m, 4 Aug 1979, *Á. Ventura A. 3477* (ENCB, F, MEX). **Durango**: 1 km al noroeste de Santa María Ocotán, Mezquital, [24.1, -104.6], 13 Jul 1984, *M. González Espinosa 1378* (MEXU). **Hidalgo**: Mpio. Acatlán, small town of Los Reyes, ca. 9 rd. mi from Acatlán, along road to Huasca, NW of Tulancingo, [20.1888, -98.4489], 7200 ft, 21 Sep 1991, *E. Dean 259* (DAV, IEB). **Jalisco**: 1 km al suroeste de Nueva Colonia, Santa Catarina, [22.1554, -104.1165], 2200 m, 20 Jul 1992, *J.J. Reynoso-Dueñas 929* (IBUG, IEB). **México**: Valle de México, Cuajimalpa a Río Hondo, [19.4315, -99.2919], 2400 m, 9 Sep 1951, *Matuda 21819* (MEXU). **Michoacán**: lado sureste del Cerro El Aguila, subiendo por el poblado de Huatzanguio, 19.6064, -101.3792, 2530 m, 14 Aug 2008, *G. Cornejo-Tenorio 2840* (IEB). **Nayarit**: Sierra Madre, territory of Tepic, between Sta. Gertrudis and Sta. Teresa, [21.7167, -104.7333], 8 Aug 1897, *Rose 2068* (GH). **Puebla**: Mpio. Xochiapulco, Rosa Chica, en un sitio llamado El Plan, cerca de la escuela primaria, 19.7950, -97.6568, 2041 m, 25 Jun 2015, *M. Jiménez -Chimil 30760* (DAV). **Querétaro**: El Picacho, desviación San Pedro Tenango, 3 km al sureste de Amealco, [20.1374, -100.1152], 2650 m, 20 Jul 2003, *V. Serrano-Serrano 121* (IEB). **San Luis Potosí**: chiefly in the region of San Luis Potosí, 6000–8000 ft, 1878, *Parry 662* (GH, MO, NY [the K duplicate of this collection number is L. moziniana var. margaretiana]). **Tlaxcala**: Mpio. Espanita, 0.95 rd mi from intersection with Hwy 136, along road to Espanita, [19.4931, -98.4599], 8920 ft, 28 Oct 1991, *E. Dean 302* (DAV). **Veracruz**: camino a Zacatonal, 18.7869, -97.2767, 9 Jul 2013, *A.F. Vargas-Rueda 637* (MEXU). **Zacatecas**: 2 km al oeste de Monte Escobedo, 22.3253, -103.5829, 2376 m, 28 Aug 2005, *A. Rodríguez 4462* (IBUG).

#### 30b Lycianthes moziniana (Dunal) Bitter var. margaretiana

Taxon classificationPlantae

E.Dean, Bot. J. Linn. Soc. 145: 413. 2004

Fig. 68

##### Type.

Mexico. Nuevo León: Mpio. Zaragoza, Cerro El Viejo, 2085 m, 17 Jun 1993, *Hinton et al. 22937* (holotype: DAV [DAV155246]; isotypes: GBH [GBH022937], TEX [00208090], XAL [XAL0106696]).

**Figure 68.**
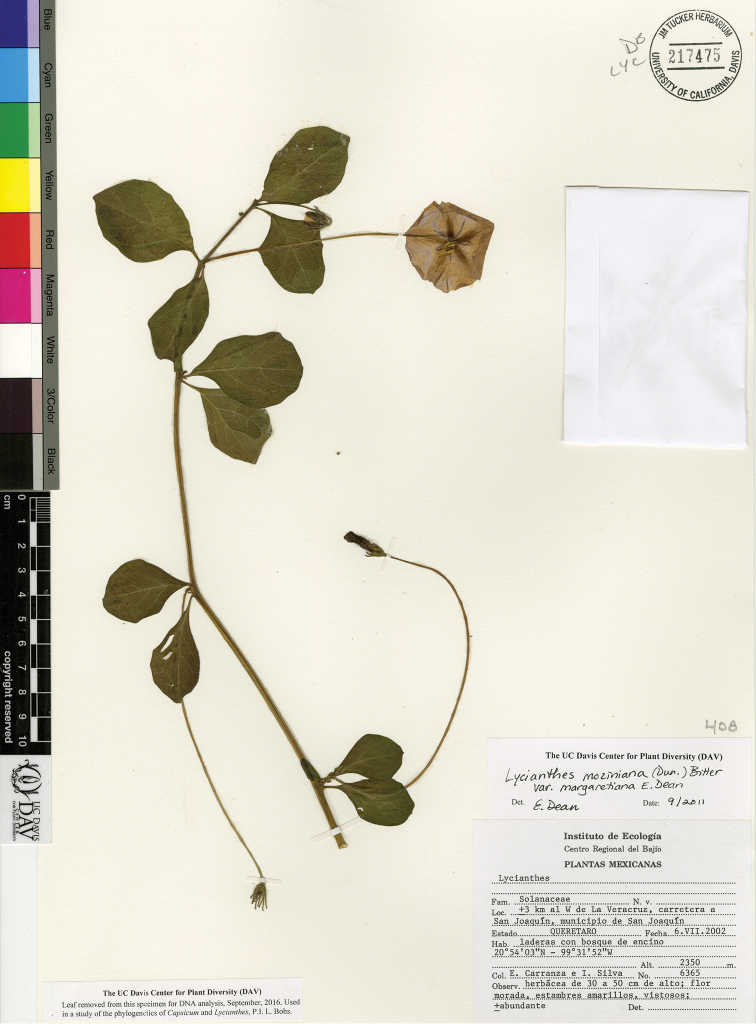
Image of herbarium specimen of L. moziniana var. margaretiana, *Carranza 6365* (DAV). Image used with permission of the UC Davis Center for Plant Diversity.

##### Description.

Perennial herb, from fusiform storage roots, erect, 0.1–0.6 m tall, dying back each season. Indument of white, uniseriate, multicellular, simple, eglandular, spreading to appressed trichomes 0.1–1.5 mm long, these often of two distinct lengths, the shorter more numerous, 0.1–0.25 mm long and appressed, the longer less numerous, 0.5–1.5 mm long and spreading. Stems green to purple-green, sparsely to moderately pubescent, compressed and ribbed when dried in a plant press, usually with very little woody tissue except at the base; first stem 9–30 cm long to the first inflorescence, internodes (3) 6–14 ; first sympodial branching point usually dichasial, the subsequent sympodial branching points usually monochasial. Leaves simple, those of the upper sympodia usually paired and unequal in size, the larger ones with blades 1.5–10 × 0.5–4.5 cm, the smaller ones with blades 1/3–9/10 the size of the larger, the leaf pairs similar in shape, the blades ovate, elliptic, or obovate, chartaceous, sparsely pubescent with trichomes similar to those of the stem, the primary veins 4–6 on either side of the midvein, the base cuneate, attenuate onto the petiole, the margin entire, usually irregularly undulate, the apex rounded to acute, the petioles to 1.5 cm long, sometimes absent. Flowers solitary, axillary, oriented horizontally; peduncles absent; pedicels 30–130 (190) mm and erect in flower, 60–100 mm (probably longer) and deflexed in fruit, often looped or curved, moderately pubescent with trichomes of two distinct lengths, the smaller 0.1–0.3 mm and appressed-retrorse, the longer 0.5–1.5 mm and mostly spreading (slightly retrorse), rarely only the longer trichomes present; calyx 3–5 mm long, 4–7 mm in diameter, campanulate (conic), moderately pubescent (densest on the ribs), the margin truncate, with 10 linear, slightly spreading appendages 3–8 (12.5) mm long emerging ca. 0.5 mm below the calyx rim; fruiting calyx enlarged, 8–9 mm long, 16–17.5 mm in diameter, the teeth spreading slightly, often broken, 3.5–9 mm long; corolla 1–2.8 cm long (2–4.6 cm in diameter), rotate in orientation, mostly entire in outline (with shallow notches), with abundant interpetalar tissue, lilac, with darker purple stripes on the major veins adaxially, green and glabrous to very sparsely pubescent near the major veins abaxially; stamens unequal, straight, the filaments unequal, the two shortest filaments 2–3 mm long, the two medium filaments 2–3.5 mm long, the one long filament 2.5–5.5 mm long, the length of the longest filament 1.5–2 times the length of the medium filaments, glabrous to pubescent; anthers 4–5 (6) mm long, elliptic to ovate, free of one another, yellow, glabrous, poricidal at the tips, the pores ovate, dehiscing distally, not opening into longitudinal slits; pollen grains tricolporate; pistil with glabrous ovary, the style 9–12 mm, linear, straight to slightly curved, glabrous, the stigma round to slightly bilobed. Fruit a berry, remaining attached to calyx at maturity, pendent, 22–30 mm long, 11–19 mm in diameter, ovoid, the exocarp green with rose or tan blotches, glabrous, the mesocarp soft, juicy, lacking sclerotic granules, the placental area variable in texture, sometimes green and juicy, other times purplish and slightly powdery. Seeds ca. 50–100 per fruit per fruit, 2.3–2.8 × 1.7–2.5 mm, rounded, slightly compressed, reniform to depressed-obovate, brownish-black, the surface reticulum with minute serpentine pattern and shallow luminae.

##### Chromosome number.

Unknown.

##### Distribution and habitat.

**Figure 69.**
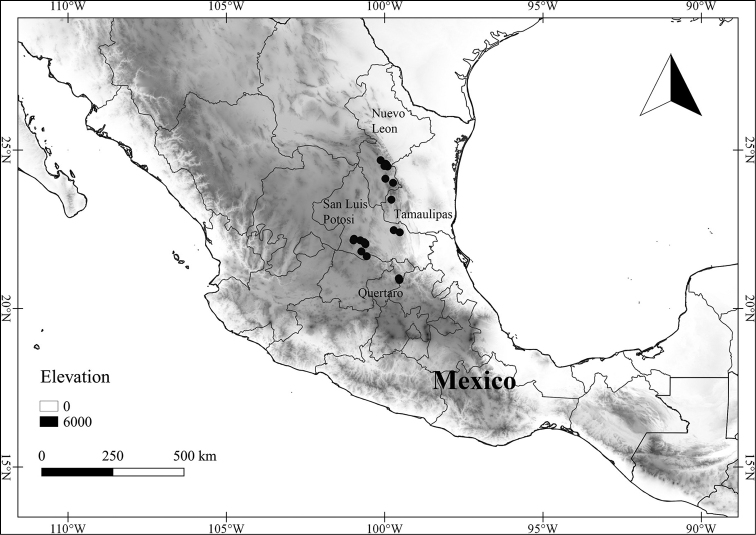
Map of geographic distribution of L. moziniana var. margaretiana based on herbarium specimen data.

Sierra Madre Oriental of Mexico (Nuevo León, Querétaro, San Luis Potosí), in oak and pine forest that may be mixed with xerophilous scrub, on limestone soils, 900–2700 m in elevation (Fig. 69).

##### Common names and uses.

None known.

##### Phenology.

Flowering specimens have been collected July and August (May in Tamaulipus); mature fruits of Lycianthes moziniana var. margaretiana have not yet been collected from the field. As discussed below, intermediates between L. moziniana var. margaretiana and *Lycianthes ciliolata* are known from several locations, and specimens with mature fruits have been collected from those populations in November and December. The diurnal corolla movements of this variety were observed in the greenhouse by the first author; the corolla opens in the very early morning and close by late morning. The pollen has a sweet scent.

##### Preliminary conservation status.

Lycianthes moziniana var. margaretiana is an uncommon variety of the Sierra Madre Oriental of Mexico, represented by 19 collections, two of which are from the protected area of the Sierra de Álvarez. The EOO is 36,938.334 km^2^, and the AOO is 76 km^2^. Based on the IUCN (2019) criteria, the preliminary assessment category is Near Threatened (NT).

##### Discussion.

Lycianthes moziniana var. margaretiana is closely related to L. moziniana var. oaxacana E. Dean based on DNA sequence data, and it shares the attenuate leaf bases and spreading calyx teeth (in fruit) of that variety. However, its distribution is disjunct from var. oaxacana and presumably the two varieties have been separated for quite some time. Variety *margaretiana* has several characteristics that differ from the other two varieties of *L. moziniana*: 1) upon maturity, the fruit exocarp can have tan or purple patches of color; 2) the placental area of the fruits may be purple, sometimes even of a powdery texture similar to the fruits of *L. ciliolata*; 3) the pollen is somewhat larger than that of the other two varieties; 4) the stamen filaments can sometimes be pubescent; 5) the abaxial sides of the corolla lobes can sometimes be glabrous; and 6) this variety does not appear to be a weed of agricultural situations (Dean 2004).

The fruits of L. moziniana var. margaretiana in Nuevo León are green with tan or rose blotches with seeds typical in shape and size for L. moziniana var. moziniana. The placental area of the Nuevo León fruits can be intermediate between L. moziniana var. moziniana and *L. ciliolata*. Lycianthes moziniana var. margaretiana may be an evolutionary transition between *L. ciliolata* and L. moziniana var. moziniana. Intermediates between var. margaretiana and *L. ciliolata* are found in San Luis Potosí, Guanajuato, and Querétaro. The intermediate plants resemble var. margaretiana in vegetative and floral characters, however the branching pattern and pollen morphology are intermediate between the two taxa. The fruits of the intermediates are large, up to 50 mm long, the exocarp is rose-colored with the grainy light purple placental area typical of *L. ciliolata*, but the fruit shape and the shape of the fruiting calyx is that of L. moziniana var. margaretiana. Finally, the seeds of the intermediates have the texture and shape of L. moziniana var. margaretiana but are much larger (4–5 mm long) than usually found in that variety. More study is needed to understand the relationship between L. moziniana var. margaretiana and *L. ciliolata* in northern Mexico (Dean 2004).

##### Representative specimens examined.

**Mexico. Nuevo León**: Mpio. Galeana, E of the town of Pablillo, San Francisco Canyon, [24.5666, -99.9666], 4 Sep 1993, *Dean 360* (DAV, XAL, ANSM). **Querétaro**: aproximadamente al oeste de La Veracruz, carretera a San Joaquín, 20.9008, -99.5311, 2350 m, 6 Jul 2002, *E. Carranza 6365* (DAV, IEB, IBUG). **San Luis Potosí**: Hwy 86, 25 mi from Juárez Circle, beyond Xoconostle, 22.2, -100.9667, 9000 ft, 5 Jul 1971, *M. Andreasen 544* (MO).

#### 30c Lycianthes moziniana (Dunal) Bitter var. oaxacana

Taxon classificationPlantae

E.Dean, Bot. J. Linn. Soc. 145: 415. 2004

Fig. 70

##### Type.

Mexico. Oaxaca: Mpio. Santa María Jaltianguis, along hwy 175, ca. 5.0–7.2 rd mi N of Ixtlán de Juárez, N of turnoff to Sta. María Jaltianguis, W side of road, downslope along footpaths, 2439 m, 11 Oct 1991, *E. Dean 285* (holotype: DAV [DAV172076]; isotypes: NY [00687932], XAL [XAL0106697]).

**Figure 70.**
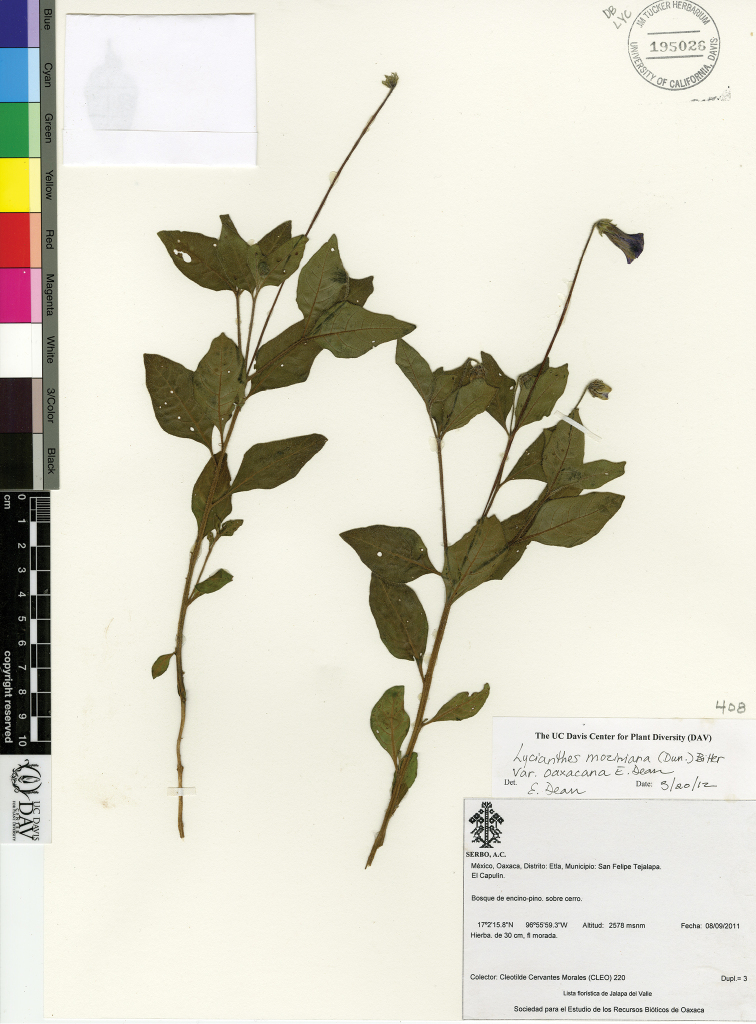
Image of herbarium specimen of L. moziniana var. oaxacana, *Cervantes 220* (DAV). Image used with permission of the UC Davis Center for Plant Diversity.

##### Description.

Perennial herb from fusiform roots, usually erect, ca. 0.1–0.4 m tall, dying back each season. Indument of white, uniseriate, multicellular, simple, eglandular, spreading to appressed trichomes 0.1–1 mm long, these often of two distinct lengths, the shorter more numerous, 0.1–0.25 mm long and appressed retrorse, the longer less numerous, 0.5–1 mm long and spreading, some populations lacking the longer trichomes. Stems green to purple-green, sparsely to moderately pubescent, only the youngest stems compressed and ribbed when dried in a plant press, woody with age; first stem 2.5–25 cm long to the first inflorescence, internodes 3–8, the first sympodial branching point dichasial or monochasial, the subsequent sympodial branching points monochasial. Leaves simple, those of the upper sympodia usually paired and unequal in size, the larger ones with blades 2.5–7 × 1.5–3.5 cm, the smaller ones with blades 3/4 the size of the larger, the leaf pairs similar in shape, the blades ovate to obovate, chartaceous, the primary veins 4–6 on either side of the midvein, the base rounded to cuneate to shortly attenuate onto the petiole, the margin entire, usually irregularly undulate, the apex acute to acuminate, the petioles 0.5–1.5 cm long, sometimes absent. Flowers solitary, axillary, oriented horizontally; peduncles absent; pedicels 50–140 mm and erect in flower, 50–90 mm long (probably longer) and deflexed in fruit, sparsely to moderately pubescent with trichomes of two distinct lengths, the smaller 0.1–0.25 mm long and appressed-retrorse, the longer 0.5–1 mm long and spreading; calyx 4–5 mm long, 4–5.5 mm in diameter, campanulate, the ribs pubescent with spreading trichomes, the margin truncate, with 10 linear, slightly spreading appendages 2–10 mm long emerging ca. 0.5 mm below the calyx rim; fruiting calyx enlarged, 5.5–9 mm long, 11–15 mm diameter, the appendages spreading slightly, not lax, often broken, 7–10 mm long; corolla 1.2–2.3 cm long (2.3–4.5 cm in diameter), rotate in orientation, mostly entire in outline (with shallow notches), with abundant interpetalar tissue, lilac, with darker purple stripes along the major veins adaxially, green and moderately pubescent near the major veins abaxially; stamens unequal, straight, the filaments unequal, the two shortest filaments 1.5–3 mm long, the two medium filaments 2–3.5 mm long, the one long filament 3–5.5 mm long, the length of the longest filament 1.5–2 times the length of the medium filaments, glabrous; anthers 4–5.5 mm, ovate, free of one another, yellow, glabrous, poricidal at the tips, the pores ovate, dehiscing distally, not opening into longitudinal slits; pollen grains tricolporate; pistil with glabrous ovary, the style 8–12 mm, linear, straight to slightly curved, glabrous, the stigma round. Fruit a berry, remaining attached to calyx at maturity, pendent, 22–28 mm long, 13–17 mm diameter, ovoid, the exocarp green, glabrous, the mesocarp and placental area soft and juicy, lacking sclerotic granules, the placental area soft and juicy. Seeds ca. 50–90 per fruit per fruit, 2.3–2.8 × 1.7–2.5 mm, rounded, slightly compressed, reniform to depressed-obovate, brownish-black, the surface reticulum with minute serpentine pattern and shallow luminae.

##### Chromosome number.

Unknown.

##### Distribution and habitat.

**Figure 71.**
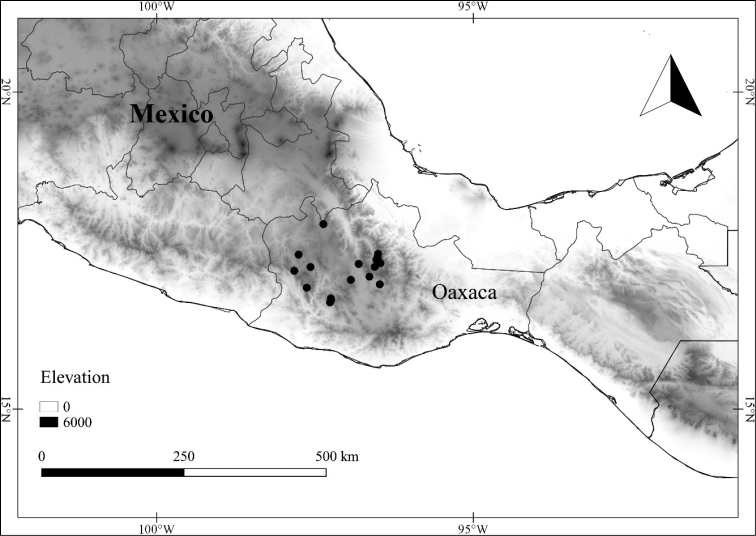
Map of geographic distribution of L. moziniana var. oaxacana based on herbarium specimen data.

Mexico (Oaxaca), in oak, oak-pine, and pine forest, typically found in anthropogenically disturbed habitats such as roadsides, pastures, old fields and corn fields, 2100–2900 m in elevation (Fig. 71).

##### Common names and uses.

Mexico. Oaxaca: chichi de venado (Dean 2004).

##### Phenology.

Flowering specimens have been collected June to July; specimens with mature fruits have been collected in October. The first author observed in the field that the corollas open in the very early morning and close by late morning. The pollen of this variety has a sweet scent.

##### Preliminary conservation status.

Lycianthes moziniana var. oaxacana is an uncommon variety of Oaxaca, Mexico, represented by 19 collections, none of which are from protected areas. The EOO is 12,876.905 km^2^, and the AOO is 72 km^2^. Based on the IUCN (2019) criteria, the preliminary assessment category is Vulnerable (VU).

##### Discussion.

Lycianthes moziniana var. oaxacana differs from var. moziniana in having spreading calyx appendages in fruit and in having attenuate leaf bases. It is closely related to L. moziniana var. margaretiana (a northern variety found in the Sierra Madre Oriental) based on unpublished DNA sequence data, however it differs from var. margaretiana in lacking tan or purple blotches on the fruit exocarp, not having a purple, powdery placental area, and in always having glabrous stamen filaments (Dean 2004). This variety was described from the Sierra de Juárez in Oaxaca. Since that time, many more populations of L. moziniana var. oaxacana have been discovered in Oaxaca, and it is possible that some of them are the more widespread var. moziniana. Lycianthes moziniana var. oaxacana is similar to *L. ciliolata*, with which it overlaps in distribution. It differs from *L. ciliolata* in having pubescence on the abaxial side of the corolla lobes (versus no pubescence), having the length of the longest stamen filament 1.5–2 times the length of the medium filaments (vs 1.5–3 times), having trichomes of two distinct lengths on the pedicels (vs trichomes of one length), and having tricolporate pollen grains (vs grains with two pores and a remnant third pore) (Dean 2004).

##### Representative specimen examined.

**Mexico. Oaxaca**: Llano de las flores, on the Oaxaca-Valle Nacional Highway, 20 km E of Ixtlán, 2870 m, 22 Jul 1960, *J. Beaman 3703* (GH, LL).

#### 31 Lycianthes nitida

Taxon classificationPlantae

Bitter, Abh. Naturwiss. Verein Bremen 24 [preprint]: 501. 1919

Fig. 72

Solanum calochromum S.F.Blake, Contr. U.S. Natl. Herb. 24: 21. 1922. Type: Honduras. Between Hacienda El Limon and El Paraiso, 12 May 1919, *S. Blake 7370* (holotype: US [00027489]).

##### Type.

Guatemala. [Alta Verapaz]: Cubilqüitz, [Cubilhuitz], [15.6675, -90.4293], 350 m, Aug 1907, *H. von Tuerkheim II 59* (lectotype designated by Dean and Reyes 2018a, pg. 43: BR [000000552878]; isolectotypes E [E00190704], GH [00936250], GH [00936251], M, NY [00007318, 00007334], U [U-0113931], US [00027489, 00624006], WIS).

**Figure 72.**
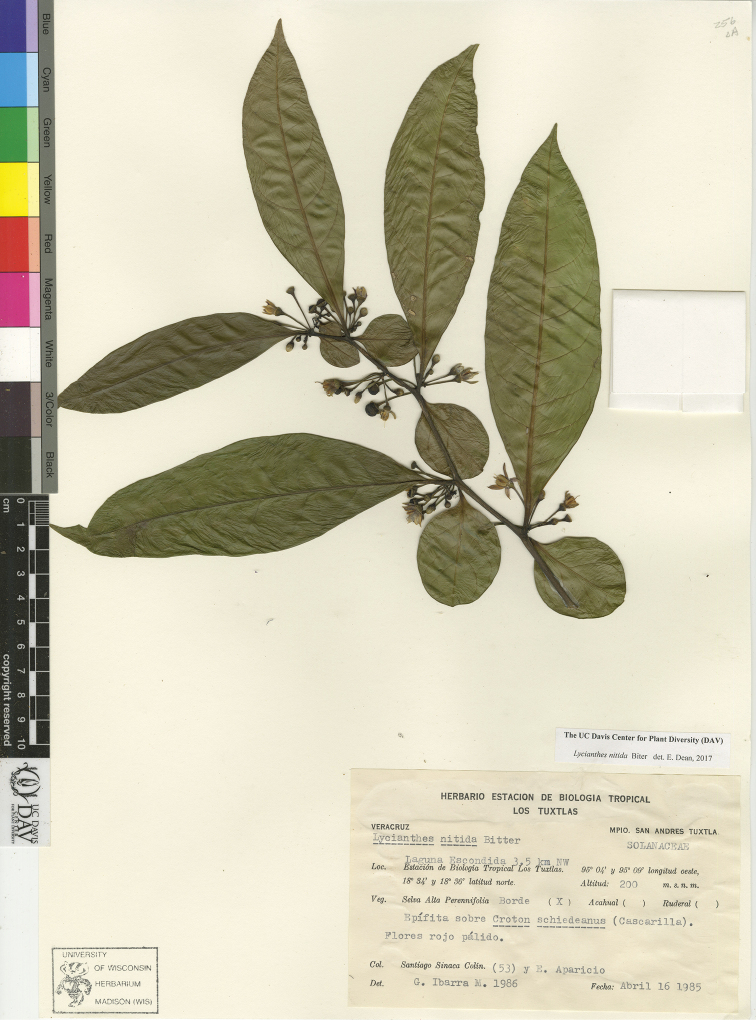
Image of herbarium specimen of *L. nitida*, *S. Sinaca C. 53* (WIS). Specimen used with permission from Wisconsin State Herbarium, University of Wisconsin, Madison.

##### Description.

Shrub, treelet, or woody vine, sometimes epiphytic, 2–6 m tall. Indument of tan to brownish, uniseriate, multicellular simple, eglandular, curved or spreading trichomes 0.1–0.5 mm long (mostly glabrous). Stems green when young, glabrous to very sparsely pubescent, not compressed upon drying in a plant press, quickly becoming woody (glossy pale grey with longitudinal wrinkles upon drying); upper sympodial branching points mostly monochasial. Leaves simple, the leaves of the upper sympodia usually paired, the leaf pairs often conspicuously different in size and shape, the larger ones with blades 8–25 × 2–9 cm, ovate (usually narrowly so), lanceolate, elliptic, or oblanceolate, the smaller ones with blades 1.75–8 × 1.3–6.7 cm, suborbicular, ovate or obovate, the leaf pairs similar in texture, coriaceous, usually glabrous, the base rounded to cuneate (usually oblique on larger leaves), the margin entire, usually undulate, the apex acute to acuminate on larger leaves, acute to rounded on smaller leaves, the petiole to 3 cm long, sometimes absent, the larger leaf blades with 6–10 primary veins on each side of the midvein. Flowers solitary or in groups of 2–11 (30), axillary, erect; peduncles absent or present as a short stub 3–5 mm long, with many pedical scars; pedicels 4–18 mm and erect in flower, to 28 mm long and erect in fruit, glabrous; calyx 1.75–4 mm long, 2.5–5 mm in diameter, widely campanulate, glabrous, the margin truncate, the appendages lacking; fruiting calyx enlarged, widely bowl-shaped, 1–4 mm long, 5–9 mm in diameter; corolla 0.6–1.3 cm long, rotate to reflexed in orientation, stellate in outline, deeply divided to the base, lacking interpetalar tissue, adaxially blue to purple and glabrous, abaxially creamy white, pinkish, or pale green and glabrous, sometimes with a linear appendage to 1 mm long at the lobe tips; stamens equal, straight, the filaments 1–2 mm long, glabrous, the anthers 5–6.5 mm long, ovate, connivent at edges to adjacent anther, forming a cone, yellow, glabrous, poricidal at the tips, the pores ovate, dehiscing distally, not opening into longitudinal slits; pistil with glabrous ovary, the style 5–10 mm long, linear, straight, glabrous, the stigma capitate. Fruit a berry, 4–12 mm long, 5–10 mm in diameter, globose to depressed globose, green to white when immature, orange to red at maturity, glabrous, lacking sclerotic granules. Seeds 50–250 per fruit, 1–2 × 1–2 mm, flattened to slightly curved, triangular, rectangular, or depressed ovate in outline, yellow to yellow orange, sometimes the margin lighter in color than the center, the surface reticulum with minute serpentine pattern and shallow luminae.

##### Chromosome number.

Unknown.

##### Distribution and habitat.

**Figure 73.**
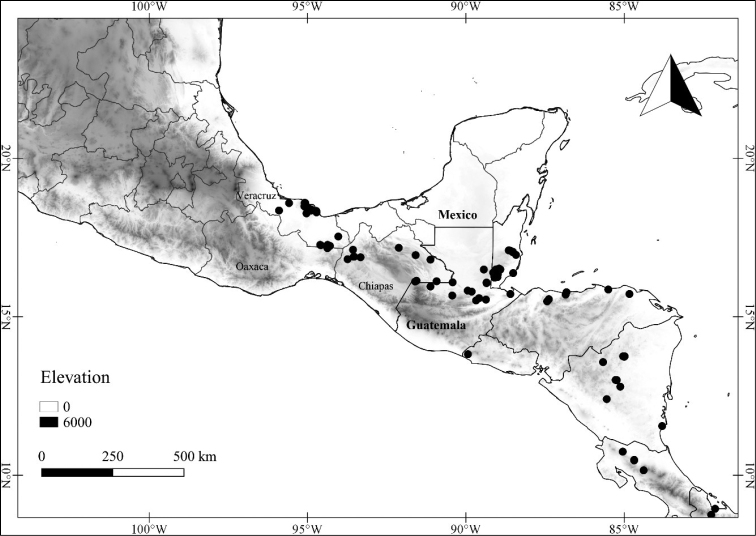
Map of geographic distribution of *L. nitida* from Mexico to Costa Rica based on herbarium specimen data.

Mexico, (Chiapas, Oaxaca, Veracruz), Guatemala (Alta Verapaz, Huehuetenango, Izabal, Petén), Belize, El Salvador, Honduras, Nicaragua, Costa Rica, and Panama in high forest, tropical moist forest, tropical rain forest, cloud forest, montane rain forest, tropical dry forest, and *Liquidambar* forest, sometimes in forest clearings or disturbed areas, including agricultural areas, or along drainages or on slopes or ridges, sometimes on limestone, 200–1000 m in elevation (Fig. 73).

##### Common names and uses.

None known.

##### Phenology.

Flowering specimens and specimens with mature fruits have been collected March through December. Possibly flowering and fruiting throughout the year in some locations. Corollas opening at night (Nee 1986) or in the morning, closed in the afternoon (from *Nee 18808*).

##### Preliminary conservation status.

*Lycianthes nitida* is a widespread species ranging from southern Mexico to Costa Rica, represented by 94 collections and occurring in 10 protected areas. The EOO is 564,238.851 km^2^, and the AOO is 352 km^2^. Based on the IUCN (2019) criteria, the preliminary assessment category is Least Concern (LC).

##### Discussion.

*Lycianthes nitida* is a relatively common and widely distributed (southern Mexico through Central America) epiphytic herb or shrub with calyces lacking appendages, purple, stellate corollas, and equal stamens. Its distinctive shiny, glabrous, coriaceous leaves, in which the geminate leaf pairs are of very different shapes and sizes (the smaller leaf much shorter and rounder than the larger) makes this species difficult to confuse with similar species that lack calyx appendages, such as *L. heteroclita* and *L. synanthera*. *Lycianthes nitida* may be confused with *L. anomala*, another epiphyte with stellate corollas and equal stamens, but *L. anomala* has short appendages on its calyx and tufts of trichomes in the vein axils of the underside of the leaves.

##### Representative specimens examined.

**Guatemala. Alta Verapaz**: 7 miles up road to Oxec along road which turns off Highway 7E between Tucúru and El Estor CA 6 km NE of Panzós, 700 m, 20 Jul 1977, *T.B. Croat 41622* (MO). **Huehuetenango**: between Ixcan and Río Ixcan, Sierra de los Cuchumatanes, bordering Río Lacandón, 150–200 m, 23 Jul 1942, *J.A. Steyermark 49352* (NY). **Izabal**: Mpio. Puerto Barrios, en la torre de Guatel, Sierra del Mico, 940 m, 8 Sep 1988, *E.M. Martínez S. 23554* (MO). **Petén**: El Petén, La Cumbre on las Cañas, on 142/143 km of El Petén/ Izabal road, 6 Mar 1975, *C.L. Lundell 19056* (DUKE, MO). **Mexico. Chiapas**: Mpio. Ocosingo, al N de la Estación Chajul, 16.0833, -90.4167, 180 m, 23 Jun 2000, *S. Sinaca-C. 2548* (XAL). **Oaxaca**: Mpio. Santa María Chimalapa, San Antonio Nuevo Paraíso, a 3 km al W, Plan de la Ceiba, 17.1625, -94.3711, 250 m, 21 Sep 1997, *E. Torres 1353* (IEB, BIGU, XAL). **Veracruz**: Rancho “El Milagro,” 5 km en línea recta al sureste de la colonia Nueva Tabasquenia, 17.53, -94.0289, 115 m, 5 Aug 2002, *E. López 195* (XAL).

#### 32 Lycianthes ocellata

Taxon classificationPlantae

(Donn.Sm.) C.V.Morton & Standl., Publ. Field Mus. Nat. Hist., Bot. Ser. 22: 274. 1940

Fig. 74

Solanum sideroxyloides Schltdl. var. ocellatum Donn.Sm., Bot. Gaz. 14(2): 28. 1889. Type: Guatemala. Department Alta Verapaz: Pansamalá, 3800 ft, May 1887, *H. von Tuerkheim 1155* (holotype: US [00027797]; isotypes: GH [00934885], NY [00139029], LE [LE0017035], US [00027798].
Lycianthes sideroxyloides (Schltdl.) Bitter ssp. ocellata Bitter, Abh. Naturwiss. Verein Bremen 24 [preprint]: 405. 1919. Type: Based on Solanum sideroxyloides Schltdl. var. ocellatum Donn.Sm.

##### Type.

Based on Solanum sideroxyloides Schltdl. var. ocellatum Donn.Sm.

**Figure 74.**
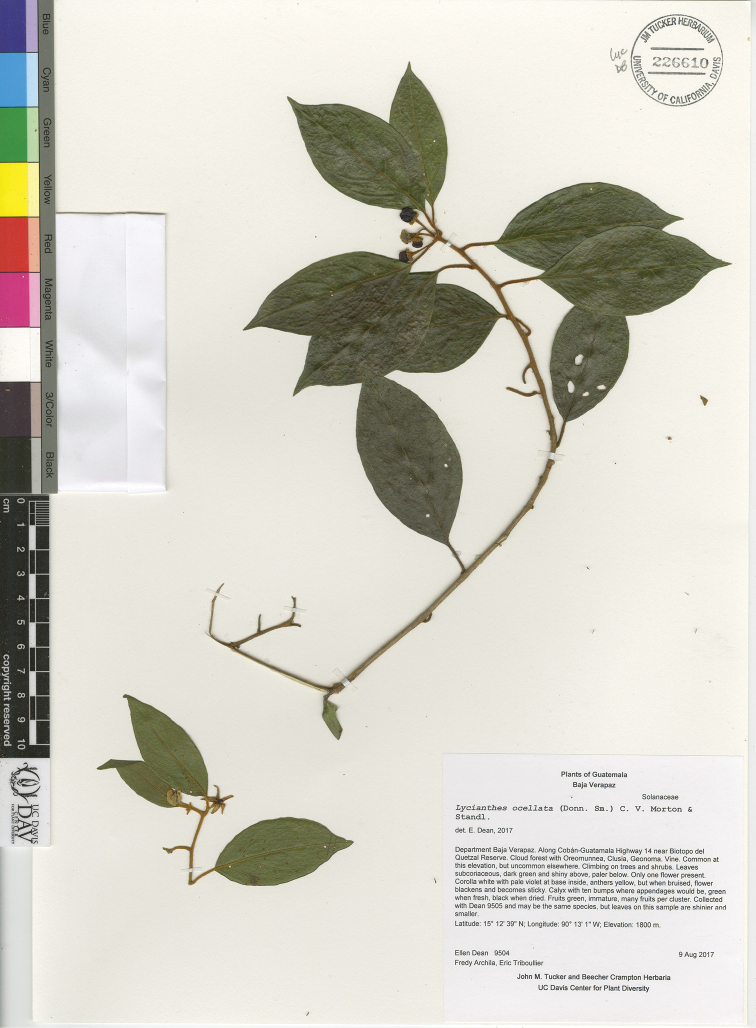
Image of herbarium specimen of *L. ocellata*, *Dean 9504* (DAV). Image used with permission of the UC Davis Center for Plant Diversity.

##### Description.

Scandent shrub to vine, climbing to 10 m (or more) into the tree canopy. Indument of pale-yellow to reddish-brown, uniseriate, multicellular, sessile or stalked, multangulate-stellate to geminate-stellate, eglandular, spreading trichomes 0.1–0.5 (0.75) mm long, 0.5–0.75 mm in diameter, the rays 5–8 per whorl, straight, not rebranched. Stems pale green (drying tan) when young, sparsely to densely pubescent, not compressed when dried in a plant press, becoming brown and woody with age; upper sympodial branching points a mixture of monochasial and dichasial, the branching near the tips of the plant not divaricate. Leaves simple, the leaves of the upper sympodia usually unpaired, the blades 3–11 × 1.5–5 cm, ovate, elliptic, or obovate, chartaceous to thick chartaceous, glabrous (especially adaxially) to moderately pubescent, the base cuneate to rounded, sometimes oblique, the margin entire, usually irregularly undulate, the apex acute to acuminate, rarely obtuse, the petiole 0.5–2.5 cm long, the larger leaf blades with 3–5 primary veins on each side of the midvein. Flowers usually in groups of 4–20, axillary, erect; peduncles absent; pedicels 4–10 mm long and erect in flower, to 15 mm long and erect in fruit, moderately to densely pubescent (the surface often obscured); calyx 2–3.5 mm long, 3–4.5 mm in diameter, campanulate, densely pubescent, the margin truncate, with 10 small obovate appendages 0.5–1 mm long emerging 0.5–1 mm below the calyx rim, the appendages sticky glandular when fresh, drying black; fruiting calyx enlarged, widely bowl-shaped, 2–2.5 mm long, 5–6 mm in diameter, the appendages not enlarging; corolla 0.6–1.2 cm long, rotate to reflexed in orientation, stellate in outline, divided 2/3 of the way to the base, with scant interpetalar tissue present at the sides of the lobes, white (lilac) and glabrous to sparsely pubescent adaxially, densely and evenly pubescent on the lobes abaxially; stamens equal, straight, the filaments 0.5–1 mm long, glabrous or with scattered trichomes, the anthers 3–4 mm long, lanceolate, free of one another, yellow to reddish-yellow, glabrous or with scattered trichomes, poricidal at the tips, the pores ovate, dehiscing distally, not opening into longitudinal slits; pistil with glabrous to sparsely puberulent ovary, the style 6–8 mm long, linear, straight, glabrous, the stigma capitate, decurrent down the sides. Fruit a berry, 5–7 mm long, 5–7 mm in diameter, globose, green to whitish when immature, orange-red when mature, glabrous or with scattered trichomes, lacking sclerotic granules. Seeds 5–10 per fruit, ca. 2 × 3 mm, flattened, thickened on edges, circular, depressed ovate, or reniform in outline, yellow-orange to dark orange, surface reticulum with minute serpentine pattern and shallow luminae.

##### Chromosome number.

Unknown.

##### Distribution and habitat.

**Figure 75.**
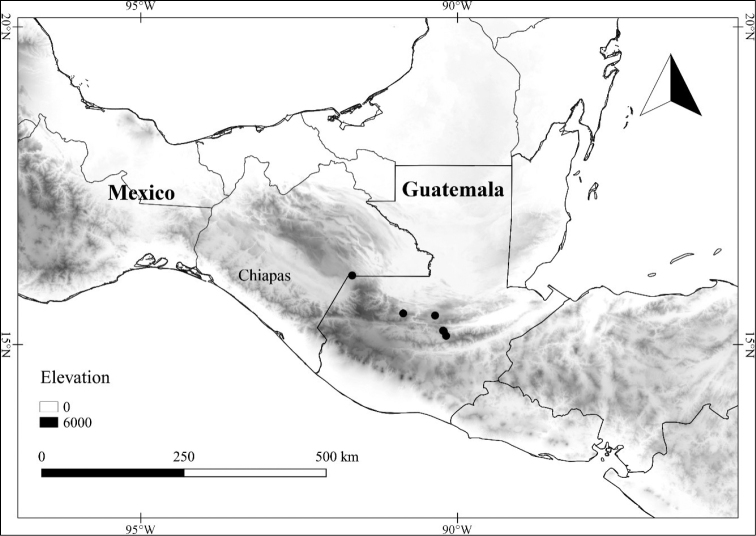
Map of geographic distribution of *L. ocellata* based on herbarium specimen data.

Mexico (Chiapas), Guatemala (Alta Verapaz, Baja Verapaz, Quiché), in montane rainforest, cloud forest, high forest, on slopes, 1300–1800 m in elevation (Fig. 75).

##### Common names and uses.

None known.

##### Phenology.

Flowering specimens have been collected May to August; specimens with mature fruits have been collected June to August. Many specimens have closed corollas, indicating that the corollas are open for a short time during the day, probably during the morning. The first author observed that the corollas were closed in the afternoon in Guatemala.

##### Preliminary conservation status.

*Lycianthes ocellata* is an uncommon species of Guatemala and immediately adjacent areas in Mexico represented by only six collections, two of which are from protected areas (Lagos de Moreno, Mexico and Mario Dary Rivera, Guatemala). The EOO is 2,567.916 km^2^, and the AOO is 24 km^2^. Based on the IUCN (2019) criteria, the preliminary assessment category is Endangered (EN).

##### Commentary.

*Lycianthes ocellata* is closely related to *L. sideroxyloides* with which it shares obovate calyx appendages, stellate corollas, and yellow to orange, geminate-stellate trichomes. It differs from that species in having glandular appendages that are shiny and sticky when fresh and turn black upon drying. The corolla and anthers are also somewhat glandular and bruise easily when fresh, turning a reddish color. *Lycianthes ocellata* is mainly restricted to the cloud forests of Guatemala; however, there is at least one collection from Chiapas at the border with Guatemala that has calyx appendages with a black area.

##### Representative specimens examined.

**Guatemala. Alta Verapaz**: Cobán, 1350 m, Jun 1907, *H. von Tuerkheim 1810* (GH, NY). **Baja Verapaz**: along Cobán-Guatemala Highway 14 near Biotopo del Quetzal Reserve, 15.2107, -90.2169, 1800 m, 9 Aug 2017, *E. Dean 9505* (DAV). **Quiché**: Cerro Putul, “Zona Reyna,” 5300 ft, 3 Dec 1934, *A.F. Skutch 1835* (A). **Mexico. Chiapas**: E of Laguna Tzikaw, Monte Bello National Park, [16.0873, -91.6625], 1300 m, 13 May 1973, *D. Breedlove 35135* (MEXU, MO).

#### 33 Lycianthes orogenes

Taxon classificationPlantae

Standl. & Steyerm., Publ. Field Mus. Nat. Hist., Bot. Ser. 23(5): 229. 1947

Fig. 76

##### Type.

Guatemala. Chimaltenango: southwestern slopes of Volcán de Fuego, above Finca Montevideo, along Barranco Espinazo, 1200–1600 m, 20 Sep 1942, *J.A. Steyermark 52104* (holotype: F [0072921F, acc. # 1148518]; isotype: US [00027892]).

**Figure 76.**
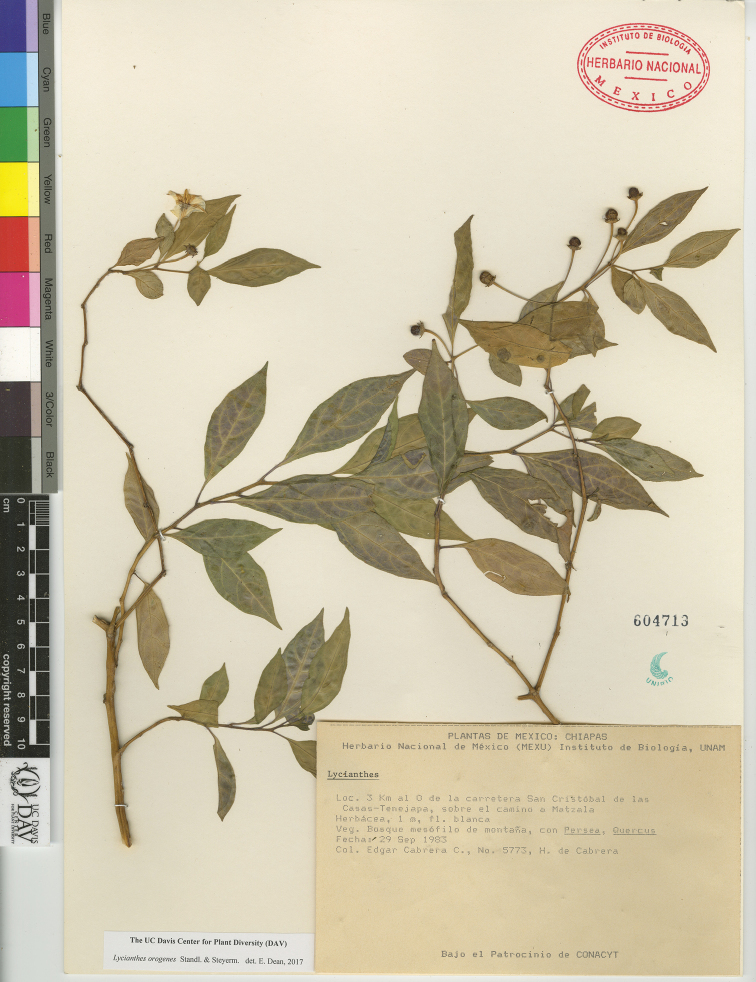
Image of herbarium specimen of *L. orogenes*, *Cabrera 5773* (MEXU). Specimen used with permission from the Herbario Nacional de México, Universidad Autónoma de México.

##### Description.

Shrub, erect, 1–3 m tall. Indument of white to light brown, uniseriate, multicellular, simple, eglandular, patent to curved and appressed-ascending trichomes 0.25–0.75 mm long. Stems green when young, glabrous to sparsely pubescent, compressed upon drying in a plant press, woody with age; upper sympodial branching points monochasial or dichasial. Leaves simple, the leaves of the upper sympodia usually paired and unequal in size, the larger ones with blades 8–18 × 2–6 cm, the smaller ones with blades 2–6.5 × 1–3 cm, the leaf pairs usually similar in shape, the blades ovate (sometimes narrowly) to elliptic, membranaceous to thin chartaceous, sometimes with purple color along the veins, glabrous to sparsely pubescent, the base cuneate to attenuate, sometimes oblique, the margin entire, usually undulate, the apex acute to acuminate, the petiole 0.2–3.3 cm long, sometimes absent, the larger leaf blades with 4–6 primary veins on each side of the midvein. Flowers solitary or in groups of 2–7, axillary, erect to nodding; peduncles absent; pedicels 15–30 mm and erect to arching in flower, to 40 mm long, arching to deflexed in fruit, usually glabrous; calyx 1.5–2.5 mm long, 3–4 mm in diameter, campanulate, glabrous, the margin truncate, with 10 erect to slightly spreading, slightly flattened appendages 1–1.5 mm long emerging 0.25–0.5 mm below the calyx rim; fruiting calyx enlarged, widely bowl-shaped, 1.5–2.5 mm long, 6–7 mm in diameter, the appendages 1–2.5 mm long, spreading; corolla 0.7–1.6 cm long, campanulate to reflexed in orientation, entire to slightly stellate in outline, divided ca. 1/5 of the way to the base, with interpetalar tissue, white, sometimes with purple markings on the adaxial side near the stamen insertion, glabrous; stamens unequal, the four shorter filaments 1–1.5 mm long, the one long filament 2–3 mm long, glabrous, the anthers 3–4 mm long, lanceolate, free of one another, yellow, glabrous, poricidal at the tips, the pores round, dehiscing distally or towards the pistil, not opening into longitudinal slits; pistil with glabrous ovary, the style 6–7 mm long, linear, straight, glabrous, the stigma capitate to oblong, decurrent down two sides. Fruit a berry, 5–8 mm long, 5–10 mm in diameter, ovoid, globose, or depressed globose, green when immature, purple at maturity, glabrous, lacking sclerotic granules. Seeds 10–50 per fruit, 2–2.5 × 1.5–2 mm, flattened, reniform in outline with deep notch on one side, orange-brown in center with yellow-orange margin, the surface reticulum with minute serpentine pattern and shallow luminae.

##### Chromosome number.

Unknown.

##### Distribution and habitat.

**Figure 77.**
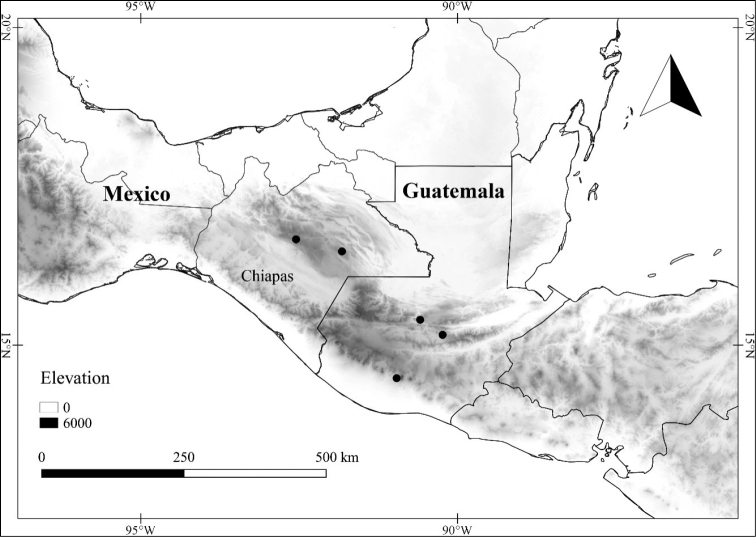
Map of geographic distribution of *L. orogenes* based on herbarium specimen data.

Mexico (Chiapas) and Guatemala (Alta Verapaz, Baja Verapaz, Chimaltenango) in cloud forest, including oak forest, 1200–2300 m in elevation (Fig. 77).

##### Common names and uses.

None known.

##### Phenology.

Flowering specimens have been collected in August and September. Specimens with mature fruits have been collected in February. Immature fruits have been collected in August. The phenological record is incomplete, due to the paucity of specimens, and there is very little information on the diurnal movements of the corollas.

##### Preliminary conservation status.

*Lycianthes orogenes* is a rarely collected species of Mexico and Guatemala, represented by only five collections, none of which is from a protected area. The EOO is 19,783.690 km^2^, and the AOO is 20 km^2^. Based on the IUCN (2019) criteria, the preliminary assessment category is Endangered (EN).

##### Discussion.

*Lycianthes orogenes* is an undercollected species of cloud forests in Chiapas and Guatemala that is morphologically similar to *L. manantlanensis* and *L. barbatula*, with which it shares slender elongate pedicels and white flowers. *Lycianthes orogenes* has calyx appendages that are intermediate in length between the two other species, and it lacks the trichomes in the leaf axils that are present in *L. barbatula*. Although the mature fruits have been reported in the literature as green (Gentry and Standley 1974; Rodríguez and Vargas 2002), they are reported as purple on specimen labels, and the immature fruits are green. Specimens of *L. manantlanensis* from some areas of Guatemala and El Salvador have sometimes been misidentified as *L. orogenes*.

##### Representative specimens examined.

**Guatemala. Alta Verapaz**: San Cristóbal, Finca Pamac II, 15.3980, -90.5883, 2179 m, 16 Aug 2015, *E. Car 35* (BIGU). **Baja Verapaz**: Unión Barrios, top of hill, W of km 153/154, 16 Aug 1975, *C.L. Lundell 19655* (LL, F, MO). **Chimaltenango**: lower and middle southwestern slopes of Volcán Fuego, above Finca Montevideo, along Barranco Espinazo and tributary of Río Pantaleón, 1200–1600 m, 20 Sep 1942, *J.A. Steyermark 52104* (US). **Mexico. Chiapas**: 3 km al O de la carretera San Cristóbal de las Casas-Tenejapa, sobre el camino a Matzala, [16.6651, -92.5487], [2500 m], 29 Sep 1983, *E. Cabrera C. 5773* (MEXU).

#### 34 Lycianthes peduncularis

Taxon classificationPlantae

(Schltdl.) Bitter, Abh. Naturwiss. Verein Bremen 24 [preprint]: 416. 1919

Fig. 78

Solanum pedunculare Schltdl., Linnaea 19: 305. 1847. Type: Germany. Leipzig Botanical Garden, 1842, *G. Kunze s.n.* (neotype designated by Dean 2004, pg. 416: W [acc. # 292213] see discussion in commentary below).

##### Type.

Based on *Solanum pedunculare* Schltdl.

**Figure 78.**
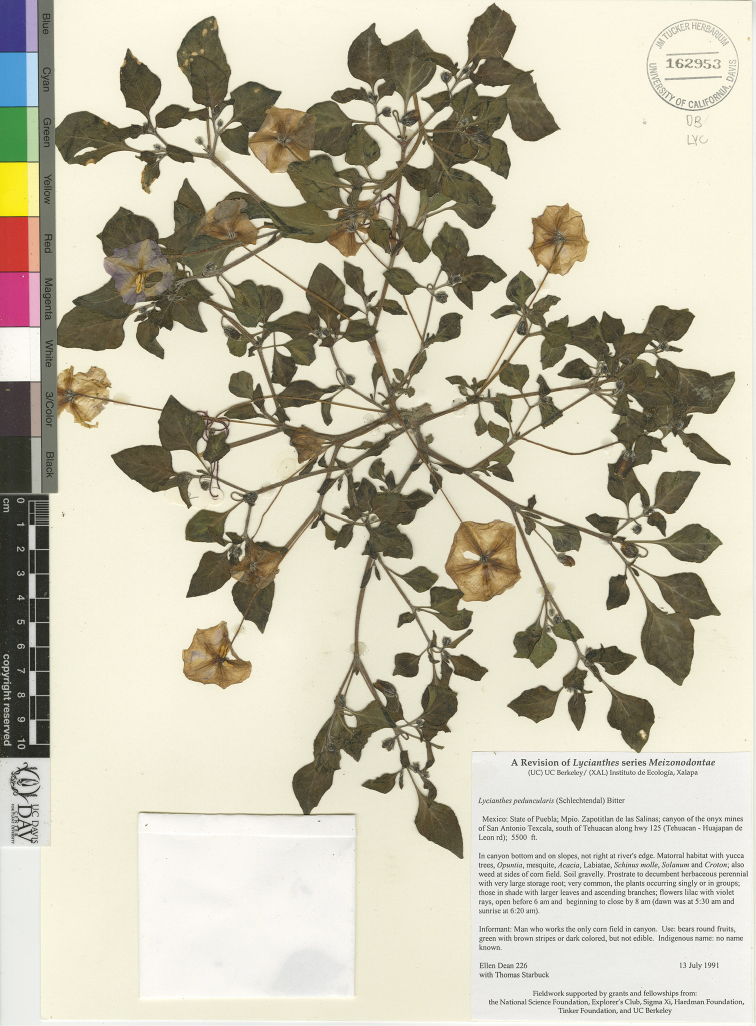
Image of herbarium specimen of *L. peduncularis*, *Dean 226* (DAV). Image used with permission of the UC Davis Center for Plant Diversity.

##### Description.

Perennial herb from large fusiform storage roots, prostate to decumbent, to 0.25 m tall and 0.5 m in diameter, dying back each season. Indument of white, uniseriate, multicellular, simple (rarely a few dendritically branched), curved, eglandular, usually appressed-ascending trichomes, 0.1–1.25 mm long. Stems green with darker green and purple striations, moderately pubescent, much compressed upon drying in a plant press, usually nonwoody; first stem 0.5–7 cm long to the first inflorescence, the internodes 4–6 (10); first sympodial branching point dichasial, followed by a mixture of monochasial and dichasial branching, this branching extensive, usually resting on the soil surface. Leaves simple, those of the upper sympodia usually paired and unequal in size, the larger ones with blades (1) 1.5–9 (14) × (0.5) 0.7–4 (7) cm, the smaller ones with blades 1/8–3/4 the size of the larger, the leaf pairs similar in shape, the blades ovate, elliptic, or obovate, thick chartaceous, sparsely to moderately pubescent, the primary veins 3–5 on either side of the midvein, the base truncate to cuneate, short to long attenuate onto the petiole, sometimes oblique, the margin entire, usually slightly undulate, the apex short acuminate to rounded, the petioles winged and poorly defined, to 2 cm long, sometimes absent. Flowers solitary, axillary, oriented horizontally; peduncles absent; pedicels (20) 30–115 mm and erect in flower, 33–180 mm long, deflexed and undulate in fruit, sparsely to moderately pubescent; calyx (1.5) 2–3.5 (4.5) mm long, (2.5) 3.25–4.5 mm in diameter, broadly cupulate, moderately pubescent, the margin truncate, with 10 linear, spreading to reflexed appendages (0.5) 1–4.5 (6) mm long emerging ca. 0.5 mm below the calyx rim; fruiting calyx enlarged, 5.5–12.5 mm long, (9.5) 12–25 mm in diameter, the teeth recurved, often making a complete loop, often broken, 1–12.5 mm long; corolla 1.1–2.5 cm long (2.2–4.9 cm in diameter) , rotate in orientation, mostly entire in outline (with shallow notches), with abundant interpetalar tissue, white to lilac, with violet stripes along the major veins adaxially, green and moderately pubescent near the major veins abaxially; stamens unequal, curved, the filaments of three different lengths, the two shortest filaments (1.25) 1.75–3 (4) mm long, the two medium filaments (1.5) 2.25–3.75 (5) mm long, the one long filament 2.5–5.5 (8.5) mm long, the length of the long filament 1.2–1.8 (2.5) times that of the medium-short filaments, glabrous; anthers (2.5) 3–5.5 (6.5) mm long, elliptic to ovate, rarely lanceolate or oblong, free of one another, yellow, glabrous, poricidal at the tips, the pores ovate to slit-like, dehiscing distally or toward the style, not opening into longitudinal slits; pollen tricolporate; pistil with glabrous ovary, the style 6–10.5 (12) mm, linear, curved downward, the stigma round to shallowly lobed. Fruit a berry, remaining attached to the calyx at maturity (the fruit matures while lying on the ground), 9–24 mm long, 9–26 mm in diameter, round to ovoid, the exocarp green with purple or black lines (becoming yellowish in age), the mesocarp thin, green and juicy, with profuse sclerotic granules, 32–83 per fruit, round to angular, yellow, the placental area narrow, greenish-white, juicy. Seeds (12) 20–100 (143) per fruit, (2.5) 3–4 × 2.2–3.1 (3.7) mm, not compressed, oblong to depressed-ovate, smooth, ridged, dark brown to black, surface reticulum with loose serpentine pattern with deep luminae and microscopic fibrils protruding from the cell walls.

##### Chromosome number.

2n = 24, *Dean 303* (Dean 2004)

##### Distribution and habitat.

**Figure 79.**
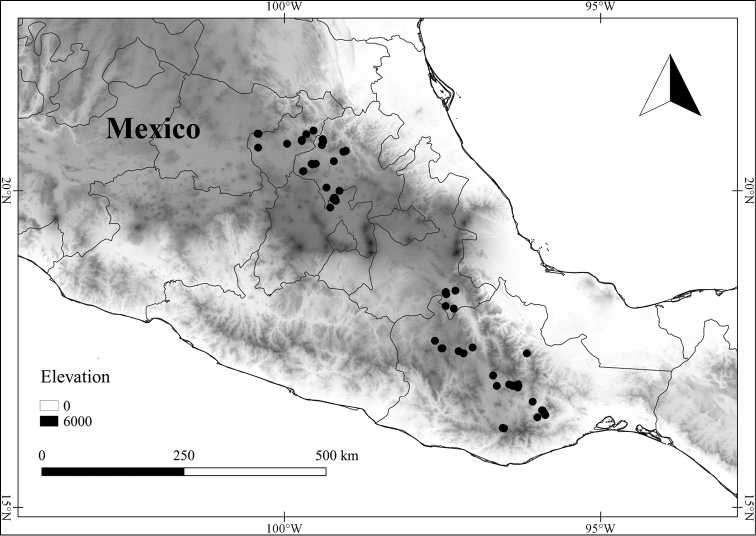
Map of geographic distribution of *L. peduncularis* based on herbarium specimen data.

Mexico (Guanajuato, Hidalgo, México, Oaxaca, Puebla, Querétaro), in xerophilous scrub, rarely pine/juniper woodland, on rocky, limestone soils, often near a drainage or in a wash or canyon, often on an eroded floodplain, sometimes within or at the sides of agricultural fields or pastures, 770–2500 m in elevation (Fig. 79).

##### Common names and uses.

Mexico. Trompeta, berenjena, chichi de perra, ojo de venado, tonchichi, tomatillo del monte (Dean 2004).

##### Phenology.

Flowering specimens have been collected June through August; specimens with mature fruits have been collected August to October. The first author has observed that the corollas open in the very early morning and close by noon. The pollen has a sweet scent. Solitary bees in the genera *Exomalopsis* and *Ptiloglossa* visit this species (Dean 2001).

##### Preliminary conservation status.

*Lycianthes peduncularis* is a common species ranging from northern to southern Mexico, represented by 64 collections and occurring in two protected areas (Yagul and Tehuacán-Cuicatlán Valley). The conservation status of this species was evaluated by Anguiano-Constante et al. (2018) and found to be Least Concern (LC).

##### Discussion.

*Lycianthes peduncularis* is recognized by its combination of prostrate to decumbent habit, simple, ascending-appressed trichomes, small calyces, and round, green fruit with maroon to black striations. The fruits have yellow sclerotic granules in the mesocarp. This species may once have had a broader and more continuous distribution on limestone soils. It is currently restricted to limestone soils on either side of the transvolcanic belt and to some eroded volcanic areas within the transvolcanic belt (rarely on rhyolite) (Rzedowski 1986). In addition, this species may tolerate other, more unusual, substrates. In Oaxaca, *L. peduncularis* has been collected near onyx and marble quarries. Several other localities are in or near mining areas. Some of the populations in Oaxaca are atypical in the size and shape of their leaves, the long length of the longest stamen filament, and the straight style (Dean 2004).

This species was widely cultivated in German botanical gardens in the 19^th^ and early 20^th^ centuries, and an interesting article was written by the German botanist Purpus on how to cultivate the species (Purpus 1923). Currently, the type of *Solanum pedunculare* is a neotype at W from the Leipzig Botanical Garden designated by Dean (2004). In his monograph of *Lycianthes*, Bitter (1919) mentioned that the type material that he studied of *S. pedunculare* were mixed collections representing both *L. moziniana* and *L. peduncularis* (Bitter 1919). As previously detailed in Dean (2004), Schlechtendal cites three syntypes. One is based on cultivated material from the Halle Botanical Garden (which he indicates is of primary importance, because he cites it after the species epithet as H. Hal) and two herbarium specimens from B, cited near the end of the protologue: *Ehrenberg 81* from Hidalgo, Mexico; *Schiede s.n.* from Michoacan, Mexico.

At the time Dean published the neotypification for *S. pedunculare*, no authentic material seen by Schlechtendal was available. Recently, the Ehrenberg specimen seen by Schlechtendal has been located at HAL and seen by the first author, and the specimen matches L. moziniana var. moziniana. This specimen is annotated by Schlechtendal as “*S. pedunculare*, *S. mocinianum*?” This indicates that he was uncertain that the specimen was *S. pedunculare*. The specimen is also annotated by Bitter as *L. mociniana*, indicating that this is the syntype he saw. The entire Solanaceae collections at GOET, HAL, M, and W were searched in 2019, and no other material of *Solanum pedunculare* seen by Schlechtendal was located. However, the original material from the Halle Botanical Garden existed at the time Bitter (1919) completed his monograph, and he was clear that that the Halle Botanical Garden material belonged to a different taxon than *S. moziniana* and matched other material that he annotated as *L. peduncularis*; the specimens and material he examined may have been lost in the destruction of the Berlin herbarium during World War II (Hiepko 1978, 1987; Vorontsova and Knapp 2010).

Because Schlechtendal placed primary importance on the horticultural syntype from the Halle Botanical Garden, we assume that he took his description from that material, material that was shared with other botanical gardens, such as the Leipzig Botanical Garden from which the neotype was collected. The characters in the protologue description of *S. pedunculare* do not match the Ehrenberg specimen in several important ways. First, the protologue says that *S. pedunculare* is a branching, prostrate plant, while the Ehrenberg specimen is clearly erect and not highly branched. Second, the protologue describes the root as thick, whereas only narrow underground stems (which lead to a root far beneath the soil surface) are visible on the Ehrenberg specimen. Third, the protologue mentions rhombic-shaped leaves with cuneate bases which are not present on the Ehrenberg specimens. In addition, although the protologue does not mention the density of pubescence, Schlechtendal adds a comment at the end of the protologue that says that the plants in their natural habitat have denser pubescence. By this comment, Schlechtendal acknowledges that the pubescence of the syntypes from Mexico do not match that of the horticultural material from the Halle Botanical Garden. *Lycianthes peduncularis* as recognized by Bitter (1919), by Dean (2004), and in this treatment is a prostrate, highly branched plant with a very thick root that is very close to the soil surface, rhombic to widely oblanceolate leaves with cuneate bases, and short, sometimes sparse trichomes. In the future, if more authentic material seen by Schlechtendal becomes available, the name *S. pedunculare* may need to be retypified, however we are not doing so at this time.

##### Representative specimens examined.

**Mexico. Guanajuato**: Mpio. San José Iturbide, near Rancho El Guajolote, SW of San José Iturbide, one hwy exit S of exit to San José, dirt rd that goes W to large drainage, farm of Margarita Vargaz Fuentes de Acosta, [20.9019, -100.4215], 6000 ft, 31 Oct 1991, *E. Dean 308* (DAV). **Hidalgo**: cañada de Arrollo Hondo, 25.9 km al noreste de Ixmiquilpan, carretera a Tolatongo, 20.6320, -99.0268, 1870 m, 17 Jun 2000, *R. Cruz-Durán 4674* (MEXU). **México**: N of Huehuetoca along the road to Apaxco, ca. 4.2 road mi from building Los Arcos in dowtown Huehuetoca, E side of rd, near where RR tracks come close to rd, [19.8896, -99.2083], 2134 m, 3 Aug 1991, *E. Dean 244* (DAV, MEXU). **Oaxaca**: Mpio. Mitla, La Colorada, 16.9261, -96.3950, 1767 m, 2 Jun 2009, *H. Hernández O. 137* (DAV). **Puebla**: Mpio. Zapotitlán de las Salinas, San Antonio Texcala, along hwy 125 S of Tehuacán, canyon with onyx mine just N of town, [18.4004, -97.4465], 1677 m, 22 Oct 1991, *E. Dean 298* (DAV). **Querétaro**: alrededores de Bernal, [20.7423, -99.9567], 2200 m, 5 Jun 1992, *R. Hernández-Magaña 9905* (MEXU).

#### 35 Lycianthes pilifera

Taxon classificationPlantae

(Benth.) Bitter, Abh. Naturwiss. Verein Bremen 24 [preprint]: 427. 1919

Fig. 80

Solanum piliferum Benth., Pl. Hartw. 68. 1840. Type: México. Oaxaca: Llano Verde, 1839, *C. T. Hartweg 499* (lectotype designated by Dean and Reyes 2018a, pg. 44: K [K000585745]); isolectotype: LD [1212266]).
Solanum pilosiusculum M.Martens & Galeotti, Bull. Acad. Brux. 12(1): 136. 1845. Type: México. Oaxaca: Cerro del Malacate (Pelado Capulalpan and Llano Verde), near Villa Alta, 7500 ft, Nov–Apr 1840, *H. Galeotti 1171* (lectotype designated by Dean and Reyes 2018a, pg. 44: BR [000000552882]; isolectotypes: BR [000000552849, 000000552911], G [G00343182], LE [LE00017009], NY [00139019], US [00027745], W [acc. # 1889-156335, acc. # 0004160]).
Lycianthes pilifera (Benth.) Bitter var. pilosiuscula (M.Martens & Galeotti) Bitter, Abh. Naturwiss. Verein Bremen 24 [preprint]: 428. 1919. Type: Based on Solanum pilosiusculum M.Martens & Galeotti.

##### Type.

Based on *Solanum piliferum* Benth.

**Figure 80.**
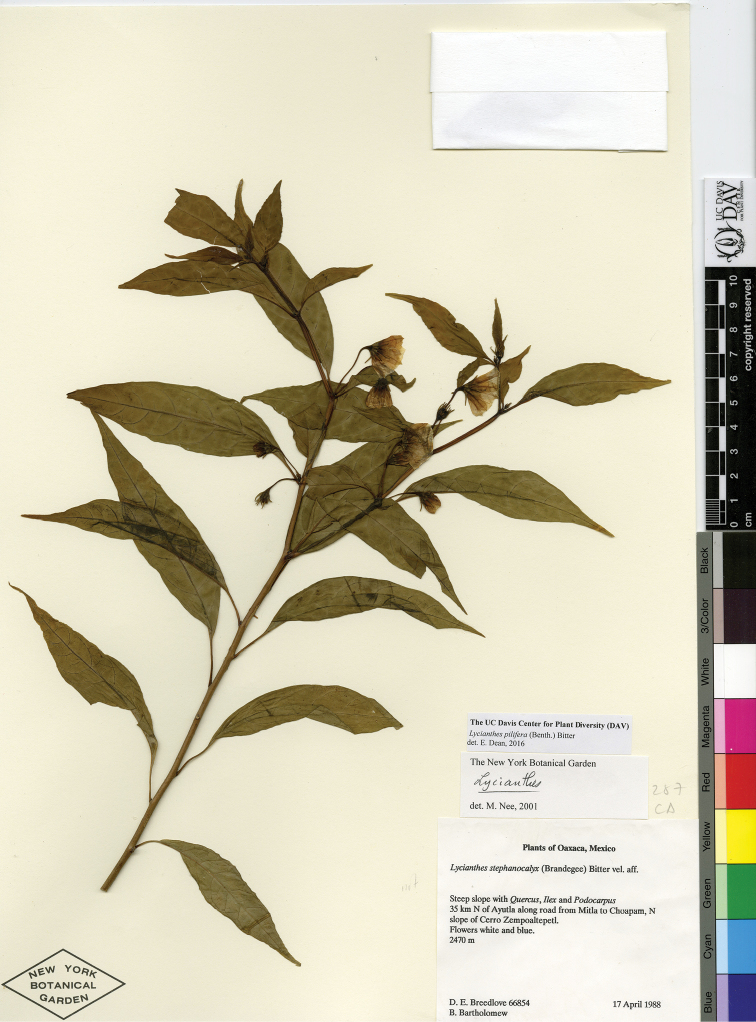
Image of herbarium specimen of *L. pilifera*, *Breedlove 66854* (NY). Specimen used with permission from the William and Lynda Steere Herbarium, New York Botanical Garden.

##### Description.

Shrub, 1–4 m tall. Indument of brown, uniseriate, multicellular, simple, acute, eglandular, appressed to spreading trichomes to 1.25 mm long, these usually remaining cylindrical and acute upon drying. Stems green to purple-green, glabrous to densely pubescent, not much compressed upon drying in a plant press, brown and woody with age; upper sympodial branching points mostly monochasial, some dichasial. Leaves simple, the leaves of the upper sympodia usually paired and unequal in size, the larger ones with blades (3) 6–15 × (1) 2–6.5 cm, elliptic to obovate (sometimes narrowly so), the smaller ones with blades 1–6 × 0.6–3 cm, suborbicular, ovate, elliptic or obovate, the blades of both the large and small leaves chartaceous to subcoriaceous, glabrous to moderately pubescent (denser on the veins), the base cuneate (sometimes rounded in smaller leaves), sometimes oblique, the margin entire, usually undulate, the apex acute to acuminate, the petiole to 1 (3) cm long, sometimes absent, the larger leaf blades with 4–7 primary veins on each side of the midvein. Flowers solitary or in groups of 2–6, axillary, oriented horizontally to nodding; peduncles absent; pedicels 15–60 mm long and arching in flower, to 30–55 mm long (probably longer) and arching in fruit, glabrous to densely pubescent; calyx 2–3 mm long, 3–4.5 mm in diameter, campanulate, often purplish in color, glabrous to densely pubescent, the margin truncate, with 10 spreading, linear-subulate appendages 2–9 mm long emerging 0.5–1 mm below the calyx rim; fruiting calyx enlarged, widely bowl-shaped, 1.5–4 mm long, 7–9 mm in diameter, the appendages up to 15 mm long; corolla 0.8–2.1 cm long, campanulate in orientation, entire to shallowly stellate in outline, with abundant interpetalar tissue, adaxially white to light purple with darker purple ring near the base (sometimes with a green ring or spots at base below the purple ring), glabrous, abaxially white to purple, sometimes green near the major veins, nearly glabrous; stamens equal, straight, the filaments 1–2 mm long, glabrous, the anthers 5–6 mm long, ovate to lanceolate, free of one another, yellow-purple to purple, glabrous, poricidal at the tips, the pores ovate, dehiscing toward the style, not opening into longitudinal slits; pistil with glabrous ovary, the style 10–11 mm long, linear, glabrous, the stigma capitate. Fruit a berry, (6) 12–18 mm long, 9–15 mm in diameter, ovoid, dark purple at maturity, glabrous, lacking sclerotic granules. Seeds 10–30 per fruit, 2.5–4 × 2–2.5 mm, compressed but not flat, ridged on one side or near the center, irregular in outline (shallowly crescent-shaped, semi-circular, depressed ovate, rhombic, or reniform with small notch), medium-brown to nearly black, the surface reticulum with a serpentine to honeycomb pattern with deep luminae, appearing pitted, with fibrils protruding from the cell walls.

##### Chromosome number.

Unknown.

##### Distribution and habitat.

**Figure 81.**
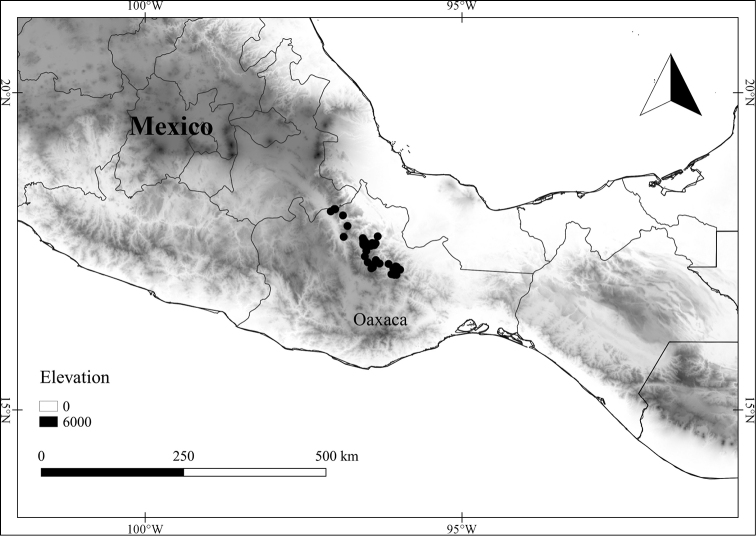
Map of geographic distribution of *L. pilifera* based on herbarium specimen data.

Mexico (Oaxaca), in cloud forest, tropical moist forest, including pine-oak, oak, and mixed forest with *Ilex*, *Podocarpus*, *Weinmannia*, *Persea*, *Ocotea*, *Oreomunnea*, *Taxus* and/or *Cupressus*, in shady canyons, slopes, and drainages, 1800–3050 m in elevation (Fig. 81).

##### Common names and uses.

Mexico. Oaxaca: monte agua zapote (*J. Rivera-Reyes 2609*); rojo monte papel (*J. Rivera-Reyes 3141*).

##### Phenology.

Flowering specimens and specimens with mature fruits have been collected most months of the year. The corollas are at least partially open on many specimens, indicating that they are open for much of the day.

##### Preliminary conservation status.

*Lycianthes pilifera* is a common species of the cloud forests of Oaxaca, represented by 61 collections, none of which is from a protected area. The EOO is 4,808.353 km^2^, and the AOO is 192 km^2^. Based on the EOO and AOO areas, and following the IUCN (2019) criteria, the preliminary assessment category is Endangered (EN).

##### Discussion.

*Lycianthes pilifera* is extremely variable in terms of width of leaves, size of flowers and calyx appendages, as well as amount of pubescence. The type material has nearly glabrous, relatively narrow leaves and relatively short calyx appendages. Morphological forms with longer calyx appendages are found in Oaxaca below 2000 m, and forms with shorter appendages are found above that elevation. Very small-leaved, small-flowered, and small-fruited forms have been collected from near Conception Papalo, Oaxaca, at 2700 m (Dean et al. 2019b). This species has been sometimes confused with *L. stephanocalyx* and *L. quichensis*, both of which can have one to two-flowered inflorescences and flowers with equal stamens. Unlike *L. pilifera*, both of those species have red fruits. *Lycianthes quichensis* is only found in Chiapas and Guatemala and does not overlap in distribution with *L. pilifera*. *Lycianthes stephanocalyx* does overlap in distribution with *L. pilifera* and differs in having red fruit, connate anthers (which are usually yellow), and small, whitish, curved trichomes. *Lycianthes pilifera* also resembles *L. caeciliae*, an endemic of Veracruz, in having purple flowers with equal stamens and dark purple fruit with large, dark seeds, however *L. caeciliae* differs in having dark purple, stellate corollas and dark purple anthers (Dean et al. 2019b).

##### Representative specimen examined.

**Mexico. Oaxaca**: Sierra de Juárez, Mpio. San Pedro Yólox, along Hwy 175 to the NE of the turnoff to Comaltepec and NE of the cabins and restaurant of Mirador, along old undeveloped road to Yólox (just E of new turnoff to Yólox), 17.6028, -96.4175, 2022 m, 10 Sep 2017, *E. Dean 9522* (DAV225278).

#### 36 Lycianthes pringlei

Taxon classificationPlantae

(B.L.Rob. & Greenm.) Bitter, Abh. Naturwiss. Verein Bremen 24 [preprint]: 421. 1919

Fig. 82

Solanum pringlei B.L.Rob. & Greenm., Amer. J. Sci. ser. 3, 50: 160. 1895. Type: México. Jalisco: mountain cañons near Guadalajara [isotypes say Sierras near L. Chapala], 18 Nov 1892, *C. Pringle 5343* (holotype: GH [00077531]; isotypes: MEXU [MEXU00028862, MEXU00029069], US [00027755], VT [UVMVT026445]).

##### Type.

Based on *Solanum pringlei* B.L.Rob. & Greenm.

**Figure 82.**
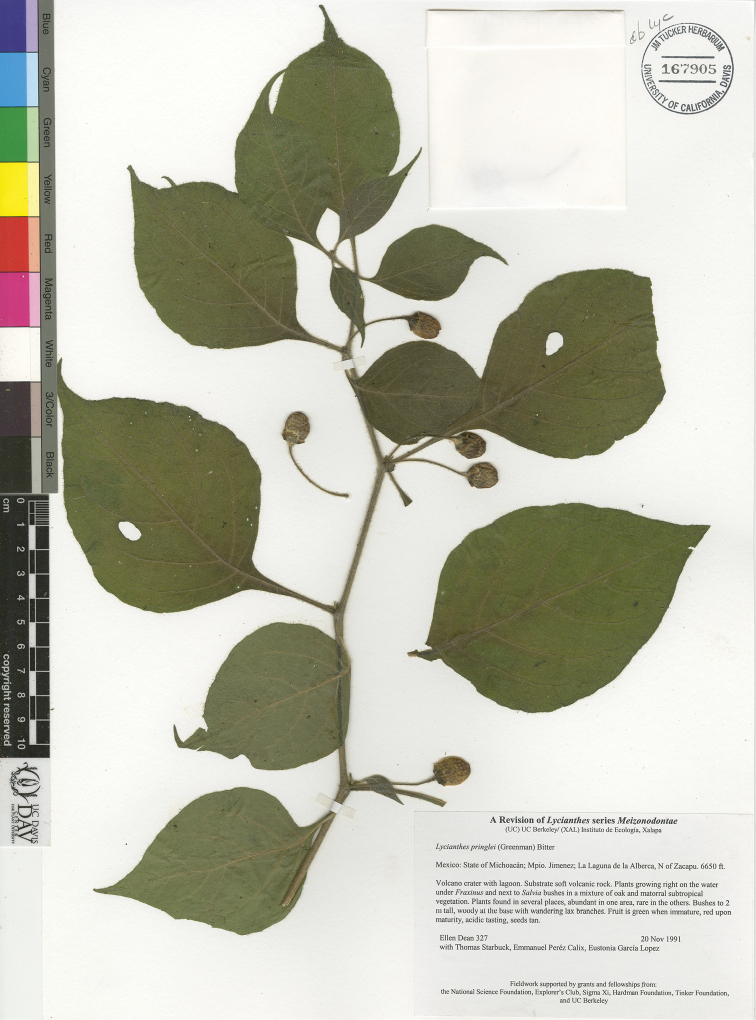
Image of herbarium specimen of *L. pringlei*, *Dean 327* (DAV). Image used with permission of the UC Davis Center for Plant Diversity.

##### Description.

Shrub, to 2 m tall. Indument of grey, uniseriate, multicellular, simple, glandular and eglandular, spreading trichomes (0.25) 0.5–1.5 (2.5), often glabrate with age. Stems pale green-brown and herbaceous when young, moderately pubescent, not much compressed when dried in a plant press, becoming brown and woody with age, often glabrate; upper sympodial branching points dichasial and monochasial. Leaves simple, the leaves of the upper sympodia usually paired and unequal in size, the larger ones with blades (6) 8–14 (18.5) × 3–9 cm, the smaller ones with blades 3–10 × 1.9–5.6 cm, the leaf pairs similar in shape, the blades widely ovate, elliptic, or obovate, chartaceous, moderately pubescent, the base truncate to short-attenuate, sometimes oblique, the margin entire, usually irregularly undulate, the apex acute to acuminate, the petiole (0.2) 1–4 (5) cm long, the larger leaf blades with 4–6 (7) primary veins on each side of the midvein. Flowers solitary or in groups of 2–3, axillary, oriented horizontally; peduncles absent; pedicels (5) 20–35 (45) mm long and erect to arching in flower, 12–35 mm long and erect to arching in fruit, moderately pubescent; calyx 3–7.5 mm long, 3–7.5 (11) mm in diameter, urceolate to campanulate, mostly glabrous except for spreading trichomes at the base near the juncture with the pedicel, the margin undulate, sometimes torn and the calyx appearing lobed, with 10 small triangular appendages 0.25–1.5 mm emerging 1–2 mm below calyx rim, these often reduced to oval protuberances 0.25–1 mm in diameter; fruiting calyx enlarged, widely bowl-shaped, 3–4 mm long, 7–12 mm in diameter, the appendages not enlarging, darkening in color; corolla 0.7–2 cm long (1.4–2.5 cm in diameter), slightly campanulate to rotate in orientation, entire in outline, with abundant interpetalar tissue, adaxially pale lilac to blue-purple with darker purple markings on the lobes, glabrous, abaxially pale lilac to blue-purple, glabrous; stamens unequal, straight, the four short filaments 1–4 mm long, the one long filament 3–7 mm long, glabrous, the anthers 3–3.5 mm long, elliptic, free of one another, yellow, glabrous, poricidal at the tips, the pores ovate, dehiscing distally, not opening into longitudinal slits; pistil with glabrous ovary, the style 6–10 mm long, linear, slightly curved, glabrous, the stigma capitate, slightly bilobed. Fruit a berry, 7–20 mm long, 5–12 mm in diameter, ovoid, orange to red at maturity, glabrous, lacking sclerotic granules. Seeds 90–200 per fruit, 0.8–1.7 × 0.6–1.5 mm, flattened but not flat, rounded on the edges, depressed ovate to round in outline, tan-orange to reddish brown, the surface reticulum pitted with loose serpentine pattern and deep luminae.

##### Chromosome number.

Unknown.

##### Distribution and habitat.

**Figure 83.**
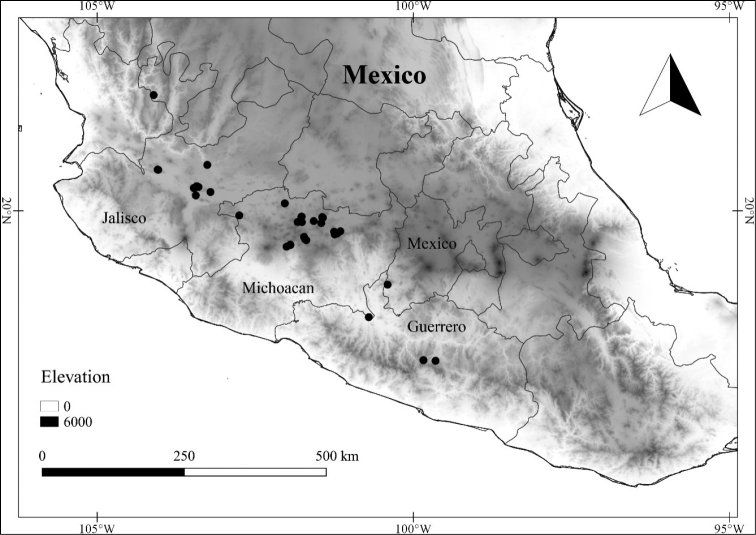
Map of geographic distribution of *L. pringlei* based on herbarium specimen data.

Mexico (Guerrero, Jalisco, México, Michoacán), in oak, oak-pine, tropical dry forest, and xerophilous scrub, often in moist or seasonally wet habitat, on volcanic soils, 1750–2100 m in elevation (Fig. 83).

##### Common names and uses.

None known.

##### Phenology.

Specimens with flowers have been collected from June through January; specimens with mature fruits have been collected from August through March. The first author observed in the field that the corollas are open in the morning and closed by afternoon.

##### Preliminary conservation status.

*Lycianthes pringlei* is a rarely collected species of western Mexico, represented by 36 collections, none of which is from a protected area. The EOO is 49,603.413 km^2^, and the AOO is 128 km^2^. Based on the IUCN (2019) criteria, the preliminary assessment category is Endangered (EN).

##### Discussion.

The calyx of *Lycianthes pringlei*, with its undulate margin that may look bilabiate and poorly developed appendages which often appear as elongated dark bumps, is uncommon in the genus. This feature, in combination with the very glandular pubescence and reddish-orange ovoid fruits, differentiate this shrub from all other species of *Lycianthes* in the flora area (Dean et al. 2007).

##### Representative specimens examined.

**Mexico. Guerrero**: 4 km al sur de Tetipac, sobre el camino Tetipac-Taxco, [17.6355, -99.6487], 1820 m, 5 Dec 1982, *E.M. Martínez-Salas 2863* (MEXU, NY). **Jalisco**: Área Natural Protegida Piedras Bola, 20.6489, -104.0435, 1947 m, 29 Oct 2011, *M.A. García Martínez 128* (IBUG, MEXU). **México**: Nanchititla, [18.8346, -100.4071], 27 Nov 1935, *Hinton 8750* (CAS, GH, NY). **Michoacán**: La Alberca de Teremendo de los Reyes, 19.8064, -101.4556, 2072 m, 15 Oct 2013, *J. Contreras L. 93* (MEXU).

#### 37 Lycianthes purpusii

Taxon classificationPlantae

(Brandegee) Bitter, Abh. Naturwiss. Verein Bremen 24 [preprint]: 382. 1919

Fig. 84

Solanum purpusii Brandegee, Univ. Calif. Publ. Bot. 6: 62. 1914. Type: México. Chiapas: Finca Mexiquito, *C. Purpus 7011* (holotype: UC [acc. # 173078]; isotypes: F [0073141F, acc. # 415779]; NY [00139025]; US [00027766]).
Lycianthes purpusii (Brandegee) Bitter var. extensidentata Bitter, Repert. Spec. Nov. Regni Veg. 20: 365. 1924. Type: Guatemala. San Francisco Miramare, Apr 1878, *K. Bernoulli & O. Cario 2334* (holotype: GOET [GOET003449]).
Lycianthes vulpina Standl., Publ. Field Mus. Nat. Hist., Bot. Ser. 4: 321. 1929. Type: Honduras, Atlántida: Lancetilla Valley, 11 Jan 1928, *P. Standley 54356* (holotype: F [0072925F, acc. # 583595]; isotypes: A [00934883], US [00027906]).

##### Type.

Based on *Solanum purpusii* Brandegee.

**Figure 84.**
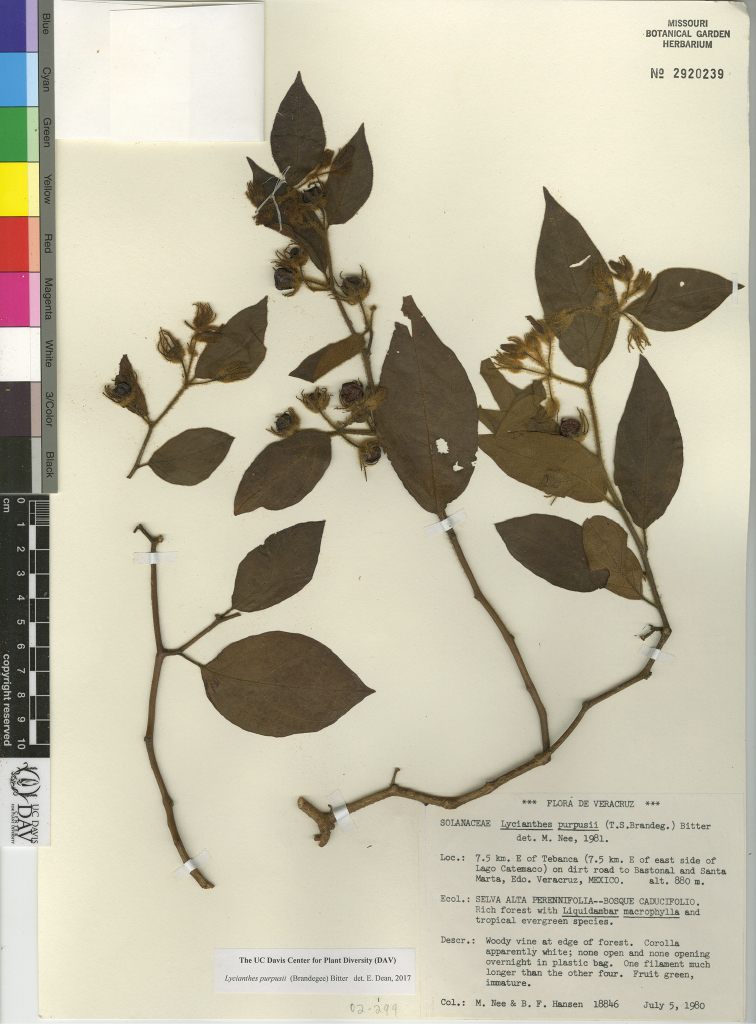
Image of herbarium specimen of *L. purpusii*, *Nee 18846* (MO). Specimen used with permission from the Missouri Botanical Garden (http://www.tropicos.org).

##### Description.

Shrub to woody vine, 2–10 m tall. Indument of long, pale yellow, orange, or red-brown, uniseriate, multicellular, simple, dendritically branched or long-stalked multangulate-stellate, eglandular, spreading trichomes 1–4 mm long, 0.75–1.4 mm in diameter, the rays of the multangulate trichomes 3–5 per whorl, straight, rarely rebranched, sometimes with an enlarged sphere where the rays join, the trichomes on an individual sometimes a mixture of colors and textures. Stems greenish-tan when young, moderately to densely pubescent, not compressed when dried in a plant press, becoming woody with age; upper sympodial branching points usually monochasial, sometimes dichasial, the branching sometimes zigzagging but not strongly divaricate. Leaves simple, the leaves of the upper sympodia sometimes paired and unequal in size, the larger ones with blades 3.5–15 × 2–8 cm, the smaller ones with blades 1.5–4.5 × 0.5–3 cm, ovate, elliptic or obovate (sometimes the small geminate leaf nearly orbicular), thick chartaceous, moderately to densely pubescent, the base cuneate to rounded, sometimes oblique, the margin entire, usually undulate, the apex acute to acuminate (rarely rounded on smaller leaves), the petiole 0.3–1.5 cm long, the larger leaf blades with 4–6 primary veins on each side of the midvein. Flowers solitary or in groups of 2–3, axillary, oriented horizontally to ascending; peduncles absent; pedicels (4) 8–20 (30) mm and erect to arching in flower, to 30 mm long and erect to arching in fruit, moderately to densely pubescent; calyx 3–4 mm long, 3.5–4.5 mm in diameter, campanulate, moderately to densely pubescent (sometimes nearly obscured), the margin truncate, with 10 very long spreading linear appendages 7–17 mm long emerging 0.5 mm below the calyx rim; fruiting calyx enlarged, widely bowl-shaped, 5–6 mm long, 11–14 mm in diameter, the appendages to 20 mm long, often curling over the fruit as it develops and then spreading when the fruit is mature; corolla 1.1–1.6 (2) cm long, rotate in orientation, nearly entire in outline, with very shallow lobes (divided about 3 mm toward the base), with abundant interpetalar tissue, white to pale blue-violet, glabrous adaxially, moderately pubescent with short trichomes abaxially near the veins; stamens unequal, the four short filaments 1–1.5 mm long, the one long filament 4–5 mm long, glabrous, the anthers 5–6 mm long, narrowly oblong to lanceolate, the anthers of some of the short anthers sometimes connivent at their edges to adjacent anther, yellow, glabrous, poricidal at the tips, the pores obovate, those of the shorter stamens dehiscing away from the style, those of the long stamen dehiscing toward the style, not opening into longitudinal slits; pistil with glabrous ovary, the style 10–12 mm long, linear, straight to slightly curved, glabrous, the stigma oblong, decurrent down two sides. Fruit a berry, 15–30 mm long, 15–30 mm in diameter, globose to depressed globose, green to white when immature, orange to red at maturity, glabrous, lacking sclerotic granules. Seeds 20–50 per fruit, 3.5–4 × 3–3.5 mm, flattened, with thickened rim, depressed ovate in outline, tan to dark brown, the surface reticulum with minute serpentine pattern with shallow luminae.

##### Chromosome number.

Unknown.

##### Distribution and habitat.

**Figure 85.**
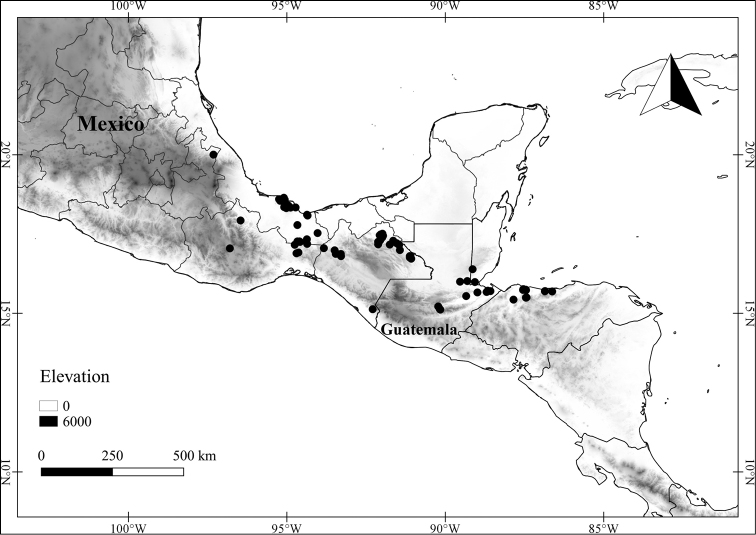
Map of geographic distribution of *L. purpusii* based on herbarium specimen data.

Mexico (Chiapas, Oaxaca, Puebla, Veracruz), Guatemala (Baja Verapaz, Izabal, Petén), Belize, and Honduras, in primary or secondary high forest or tropical dry forest, very rarely in cloud forest at the upper part of its elevational range, often on limestone, 80–1000 (1500) m in elevation (Fig. 85).

##### Common names and uses.

None known.

##### Phenology.

Flowering specimens have been collected February to October. Specimens with mature fruits have been collected throughout the year. The corollas have been reported as opening at night (Nee 1986), and all flowering specimens have closed corollas.

##### Preliminary conservation status.

*Lycianthes purpusii* is a widespread species ranging from southern Mexico to Honduras, represented by 92 collections and occurring in eight protected areas. The EOO is 91,196.026 km^2^, and the AOO is 328 km^2^. Based on the IUCN (2019) criteria, the preliminary assessment category is Least Concern (LC).

##### Discussion.

*Lycianthes purpusii* is a wide-ranging species of tall tropical forest and relatively low elevations. It is distinguished by very long calyx appendages (7–17 mm long in flower) and dendritically branched or long-stalked multangulate-stellate trichomes. The species is variable in leaf arrangement; the leaves are usually paired (geminate) in Honduras but unpaired in many places in Mexico. It is also variable in the length of the pedicels (sometimes becoming unusually long in Guatemala) and the color and density of the pubescence. This species is somewhat similar to *L. furcatistellata* Bitter of Costa Rica but differs from that species in habitat preference (*L. furcatistellata* occurs in upper elevations, often in cloud forest), length of the pedicels (*L. purpusii* often has flowering pedicels less than 20 mm long, while *L. furcatistellata* usually has pedicels greater than 20 mm long), and calyx appendage length (*L. furcatistellata* has appendages on the flowering calyxes of 4 mm or less). The two species do not have overlapping distributions. Several specimens with dense, soft, very branched calyx trichomes, very short pedicels, and flowers in very tight groupings are included in this species circumscription, and they might prove to be a separate species.

##### Representative specimens examined.

**Guatemala. Baja Verapaz**: Purulhá, Biótopo del Quetzal, 15.21306, -90.22, 400 m, 18 Oct 1995, *A. Cahuec s.n.* (BIGU). **Izabal**: Montañas del Mico, 11 km W of Santo Tomás de Castillas, microwave tower, [15.6719, -88.6929], 940 m, 8 Sep 1988, *W. Stevens 25496* (NY). **Petén**: 19 km N of Modesto Méndez, 200 m, 21 Jun 1971, *W.E. Harmon 5855* (MO). **Mexico. Chiapas**: Mpio. Barriozábal, along road from Berriozábal to Las Maravillas, ca. 1.4 km S of the town of Efraín A. Gutiérrez, remnant of tall forest called La Mata Café, 16.8711, -93.2956, 1005 m, 12 Sep 2017, *E. Dean 9527* (DAV). **Oaxaca**: Nueva Santa Flora, 17.9275, -96.4608, 700 m, 22 Dec 1992, *G. Ibarra-Manríquez 3781* (XAL). **Puebla**: Mpio. Hueytamalco, 1 km hacia el Oeste de las instalaciones del Campo Experimental “Las Margaritas,” Instituto Nacional de Investigaciones Forestales, Agrícolas y Pecuarias (INIFAP), 20.0044, -97.3167, 550 m, 19 Nov 2007, *B. Gómez-Chagala 349* (IEB, MEXU). **Veracruz**: Rancho “El Milagro,” 5 km en línea recta al sureste de la colonia Nueva Tabasquenia, 17.53, -94.0289, 115 m, 5 Aug 2002, *E. López 192* (IEB, XAL).

#### 38 Lycianthes quichensis

Taxon classificationPlantae

(J.M.Coult. & Donn.Sm.) Bitter, Abh. Naturwiss. Verein Bremen 24 [preprint]: 428. 1919

Fig. 86

Solanum quichense J.M.Coult. & Donn.Sm., Bot. Gaz. 37: 422. 1904. Type: Guatemala. Quiché: Chiul, Apr 1892, *E. T. Heyde & E. Lux 3450* (holotype: F [0073142F, acc. # 264727]; isotypes: G [G00379126], GH [GH00077532], M [M-0171931], NY [00139026], US [00624010], US [00027769].
Lycianthes obliquifolia Standl., Field Mus. Nat. Hist., Bot. Ser. 22: 101. 1940. Type: México. Chiapas: Volcán de Tacaná, 30 Mar 1939, *E. Matuda 2938* (holotype: F [0072919F, acc. # 980247]; isotypes: A [00077523], MEXU [MEXU00082111], MICH [1109852], NY [00138706]).

##### Type.

Based on *Solanum quichense* Coult. & Donn.Sm.

**Figure 86.**
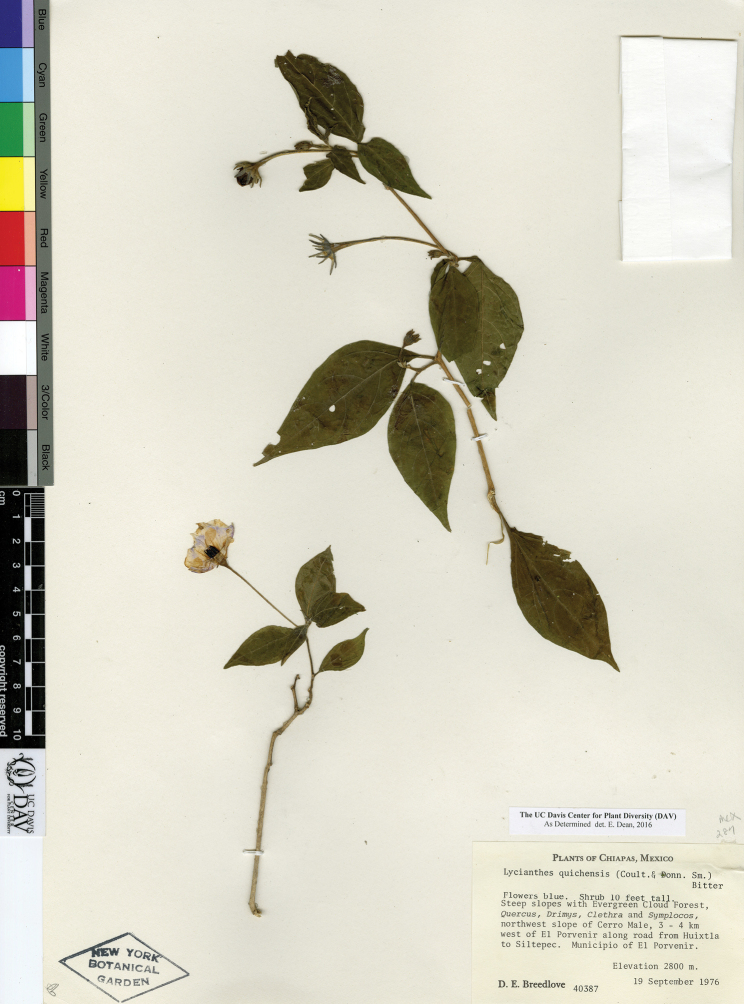
Image of herbarium specimen of *L. quichensis*, *Breedlove 40387* (NY). Specimen used with permission from the William and Lynda Steere Herbarium, New York Botanical Garden.

##### Description.

Shrub, 1–5 m tall. Indument of white to pale yellow, uniseriate, multicellular, simple, acute, eglandular, appressed to spreading trichomes 0.1–1 mm long, the larger trichomes becoming flattened upon drying. Stems green when young, sparsely to densely pubescent, not much compressed upon drying in a plant press, brown and woody with age; upper sympodial branching points mostly monochasial, some dichasial. Leaves simple, the leaves of the upper sympodia usually paired and unequal in size, the larger ones with blades 4–15 (25) × 2.5–9 (14) cm, ovate to elliptic (sometimes very widely so), rarely lanceolate, the smaller ones with blades 1.5–9 × 1–5 cm, ovate, the blades of both the large and small leaves chartaceous, sparsely to moderately pubescent, denser along the veins and in the abaxial leaf axils, the base cuneate to truncate or rounded, sometimes oblique, the margin entire, usually undulate, the apex acute to acuminate, the petiole to 4 (6) cm long, sometimes absent, the larger leaf blades with 4–7 primary veins on each side of the midvein. Flowers solitary or in groups of 2, axillary, oriented horizontally; peduncles absent; pedicels 20–60 mm long, erect to arching in flower, to 70 mm long (probably longer), deflexed and arching in fruit, sparsely to densely pubescent; calyx 3–4 mm long, 3.5–6 mm in diameter, campanulate, often purplish in color, sparsely to densely pubescent, the margin truncate, with 10 spreading, linear-subulate appendages 3–6 mm long emerging 0.5 mm below the calyx rim; fruiting calyx enlarged, widely bowl-shaped, 2–5 mm long, 7–13 mm in diameter, the appendages not greatly enlarging; corolla 1.2–3 cm long, campanulate to rotate in orientation, entire to shallowly stellate in outline, with abundant interpetalar tissue, adaxially light purple with darker purple ring near the base and green markings at very base, glabrous, abaxially light purple, sometimes paler or white on the lobes, sparsely to densely pubescent with small trichomes near the veins; stamens equal, straight, the filaments 1.5–2 mm long, glabrous, the anthers 5–6 mm long, lanceolate, free of one another, purple, glabrous, poricidal at the tips, the pores ovate, dehiscing toward the style, not opening into longitudinal slits; pistil with glabrous ovary, the style 10–12 mm long, linear, glabrous, the stigma capitate. Fruit a berry, 9–17 mm long, 7–15 mm in diameter, usually ovoid (round), red at maturity (drying dark purple on herbarium sheets), glabrous, lacking sclerotic granules. Seeds 20–40 per fruit, 2.5–3 × 2–2.5 mm, compressed but not flat, ridged on one side or near the center, irregular in outline (shallowly crescent-shaped, triangular, or semi-circular), orange-brown, the surface reticulum with a serpentine to honeycomb pattern with deep luminae, appearing pitted, with fibrils protruding from the cell walls.

##### Chromosome number.

Unknown.

##### Distribution and habitat.

**Figure 87.**
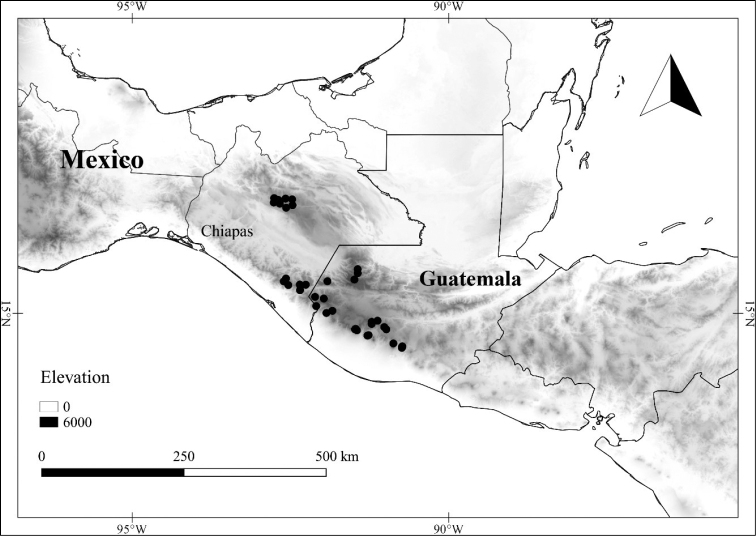
Map of geographic distribution of *L. quichensis* based on herbarium specimen data.

Mexico (Chiapas) and Guatemala (Chimaltenango, Huehuetenango, Quetzaltenango, Quiché, Sacatepéquez, San Marcos, Sololá, Totonicapán), tropical moist forests or thickets in cloud forest and oak-pine forest with *Chiranthodendron*, *Symplocos*, *Drimys*, and *Clethra* (in Guatemala often in *Cupressus* or *Abies* forests), 2200–3900 m in elevation (Fig. 87).

##### Common names and uses.

Guatemala. Chilete, choshel, coxel, flor de rosa, quilete, tomatillo blanco (Gentry and Standley 1974)

##### Phenology.

Flowering specimens and specimens with mature fruits have been collected during most months of the year. Many herbarium specimens of this species have flowers with open corollas, indicating that the flowers must stay open until at least noon, if not after.

##### Preliminary conservation status.

*Lycianthes quichensis* is a rare species of Mexico and Guatemala, represented by 38 collections and occurring in seven protected areas. The EOO is 22,627.703 km^2^, and the AOO is 148 km^2^. Based on the IUCN (2019) criteria, the preliminary assessment category is Endangered (EN).

##### Discussion.

*Lycianthes quichensis* is an attractive shrub that horticulturalists are trying to bring into cultivation and is sometimes available in seed catalogues. It has relatively large purple flowers with distinctive dark purple and green markings and widely ovate leaves. It has sometimes been confused with *L. pilifera*, a species of Oaxaca, Mexico. The two species differ in fruit color (*L. pilifera* has purple-black fruits) and pubescence (the trichomes of *L. pilifera* are darker and more tubular-conical upon drying than those of *L. quichensis* which are pale yellow to white and flattened upon drying) (Dean et al. 2019b).

##### Representative specimens examined.

**Guatemala. Chimaltenango**: Volcán de Acatenango, [14.5255, -90.8753], 2800 m, 22 Apr 1999, *M. Véliz 7020* (BIGU, MEXU). **Huehuetenango**: Mpio. La Libertad, Peña Blanca, 15.5075, -91.9158, 3193 m, 14 Dec 2000, *M. Véliz 10846* (BIGU, CAS). **Quetzaltenango**: Cumbre de Alaska, [14.7560, -91.4705], 3100 m, 10 Sep 1999, *M. Véliz 7285* (BIGU, MEXU). **Quiché**: Chiul, Apr 1892, *E.T. Heyde & E. Lux 3450* (F, GH, M, NY, US). **Sacatepéquez**: Volcán de Agua, 14.4769, -90.7322, 2852 m, 6 Feb 2006, *M. Véliz 16671* (BIGU, TEX). **San Marcos**: Mpio. San José Ojetenam, [15.2342, -91.9736], 3100 m, 26 Nov 2009, *F. Pérez 18* (BIGU). **Sololá**: San Pedro La Laguna, Volcán San Pedro, ladera noreste del volcán, 14.6572, -91.2664, 3006 m, 28 Jan 2005, *P. Pardo 27* (CAS). **Totonicapán**: María Tecún, [14.8341, -91.2174], 3000–3600 m, 12-–23 Jan 1966, *A. Molina R. 16399* (CAS-DS, NY). **Mexico. Chiapas**: Mpio. Chamula, Tzontehuitz, 16.8108, -92.5775, 2740 m, 20 Apr 1999, *L.Y. Domínguez-Torres 70* (DAV, MEXU).

#### 39 Lycianthes rafatorresii

Taxon classificationPlantae

E. Dean sp. nov.

urn:lsid:ipni.org:names:77213136-1

Fig. 88

##### Type.

México. Oaxaca: Distrito Juchitán, Mpio. Santa María Chimalapa, San Antonio Nuevo Paraiso, a 2 km en línea recta al W, en Cerro Camedor, 17.1444, -94.3577, *C. Perret 352* (holotype: MEXU [acc. # 1143589]; isotypes: IEB [acc. # 210462, acc. # 209686], SERO).

**Figure 88.**
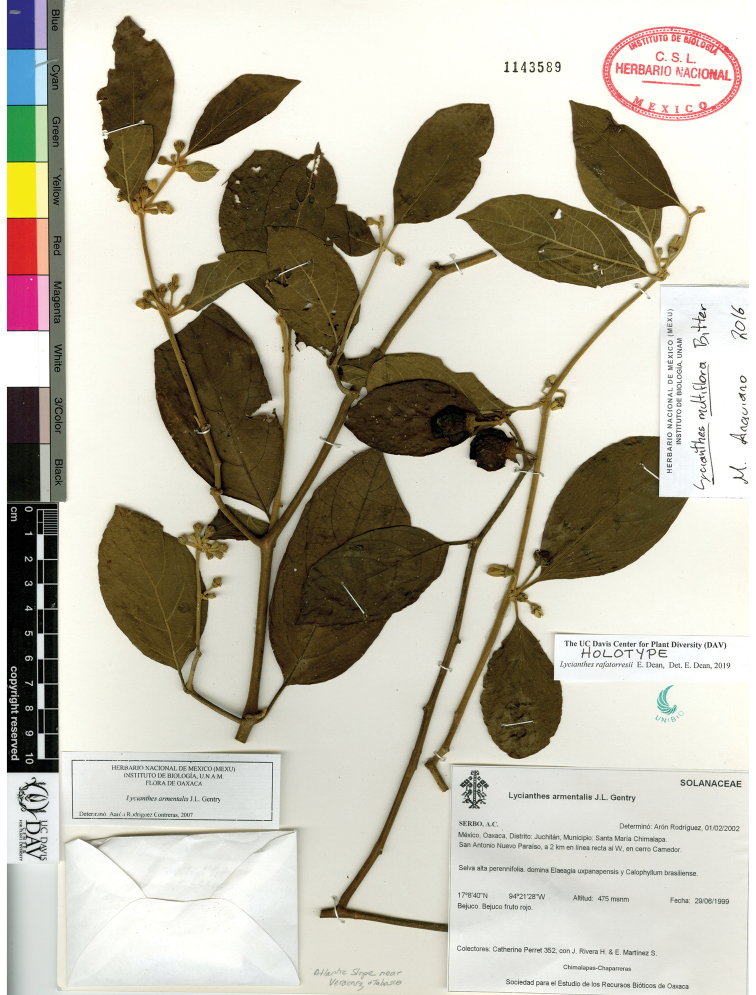
Image of herbarium specimen of *L. rafatorresii*, *Perret 352* (MEXU). Specimen used with permission from the Herbario Nacional de México, Universidad Autónoma de México.

##### Diagnosis.

Very similar to *Lycianthes multiflora* Bitter of Central America, *L. sideroxyloides* of Mexico and Central America, and *L. armentalis* of the Yucatán Peninsula. Differing from those species in having the following combination of characters: multangulate-stellate (not geminate-stellate) trichomes with straight (not crisped) trichome rays, branching not widely divaricate, inflorescences that are not restricted to the last few terminal, leafless, sympodial units of the plant, calyx appendages with a widened bulbous apex (rather than acute), stellate corollas divided one quarter to approximately half way to the base with abundant interpetalar tissue, unequal stamens, and few, large fruits, 8–15 mm in diameter with 40–100 seeds per fruit, the seeds usually slightly notched on one side.

##### Description.

Shrub (sometimes scandent), treelet, to vine, 1–5 (20) m tall. Indument of off-white, grey, pale yellow, or yellow-orange, uniseriate, multicellular, sessile to short-stalked, multangulate-stellate, eglandular, spreading trichomes 0.1–0.25 mm long, 0.25–0.75 mm in diameter, the rays 5–8 per whorl, usually straight (not crisped), not rebranched, the center of the trichome where the rays meet sometimes enlarged and spherical, the trichomes of the adaxial leaf surface often sessile and appressed to the leaf surface. Stems light green when young, (drying tan to brown), moderately to densely pubescent (appearing like dense felt), slightly compressed and ribbed when dried in a plant press, becoming brown and woody with age; upper sympodial branching points a mixture of monochasial and dichasial branching (often monochasial), the branching not divaricate. Leaves simple, the leaves of the upper sympodia usually paired, the pairs unequal in size, the larger ones with blades 4.5–13 × 2.5–6 cm, the smaller ones with blades 1.5–7.6 × 1–3.8 cm, the leaf pairs similar in shape, the blades ovate to elliptic, chartaceous to subcoriaceous, sparsely to moderately pubescent, the base cuneate (sometimes widely so), rarely rounded, sometimes oblique, the margin entire, usually irregularly undulate, the apex acute to acuminate, the petiole 0.3–2.5 cm long, the larger leaf blades with 4–6 primary veins on each side of the midvein. Flowers solitary or in groups of 2–6 (–10), axillary, oriented horizontally; peduncles usually absent, sometimes a small pad forming with numerous pedicel scars, to 2 mm long; pedicels 4–10 mm long and erect in flower, to 17 mm long and erect in fruit, moderately to densely pubescent; calyx 2–4 mm long, 3–4 mm in diameter, campanulate, moderately to densely pubescent, the margin truncate, with 10 erect linear appendages with widened, bulbous apex, 1–4 mm long, emerging 0.5–1 mm below the calyx rim, the rim membranaceous and papery; fruiting calyx enlarged, widely bowl-shaped to rotate, 2–4 mm long, 6–10 mm in diameter, the appendages to 5 mm long; corolla 0.7–1.5 cm long, orientation of open corolla unknown (most likely campanulate or rotate), shallowly stellate in outline, divided 1/4 to 1/2 of the way to the base (the division increasing by tearing as the flower ages), with abundant interpetalar tissue, the adaxial side white, glabrous, the abaxial side of the lobes of unknown color, densely pubescent with multangulate-stellate, appressed trichomes; stamens unequal, straight, the four short filaments 0.5–1 mm long, the one long filament 2–3.5 mm long, glabrous, the anthers 4–5 mm long, lanceolate, narrowed at the tip, free of one another, yellow, sparsely pubescent on the inner face, poricidal at the tips, the pores ovate, terminal, dehiscing distally, not opening into longitudinal slits; pistil with glabrous ovary, the style 7–8 mm long, linear, straight, glabrous, the stigma oblong, decurrent down two sides. Fruit a berry, 8–15 mm long, 8–15 mm in diameter, globose, red-orange when mature, glabrous, lacking sclerotic granules. Seeds 40–100 per fruit, 2–3 × 2–3 mm, flattened, circular to depressed ovate in outline, sometimes shallowly indented on one side (< 0.5 mm), thickened on the margin, yellow-orange to orange-brown, the margin darker in color than the center, the surface reticulum nearly smooth in the center with indistinct serpentine pattern and shallow luminae, the luminae much deeper on the margin.

##### Chromosome number.

Unknown.

##### Distribution and habitat.

**Figure 89.**
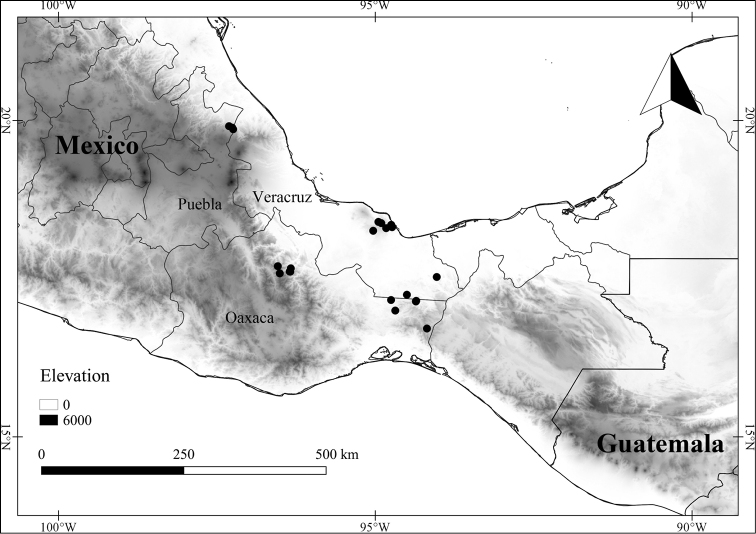
Map of geographic distribution of *L. rafatorresii* based on herbarium specimen data.

Mexico, Caribbean slope (Oaxaca, Puebla, Veracruz), in tropical moist forest, tropical dry forest, and cloud forest, in both primary forest and disturbed areas, such as coffee plantations and along roadsides, often growing with *Quercus* or *Liquidambar*, sometimes on steep slopes or along drainages, sometimes on limestone, 200–1600 m in elevation (Fig. 89).

##### Common names and uses.

Mexico. Oaxaca: paniui poj (Zoque) (*Hernández G. 1234*).

##### Phenology.

Flowering specimens have been collected March through September; specimens with mature fruits have been collected May through November. The corollas on specimens of this species are usually closed, indicating that the corollas are open for only a short time period during the day, most likely in morning.

##### Preliminary conservation status.

*Lycianthes rafatorresii* is a rare shrub with a discontinuous distribution represented by 22 collections, and it is only present in one protected area (Los Tuxtlas). The size of the EOO area (51,517.851 km^2^) suggests a preliminary conservation assessment of Least Concern (LC). In contrast, the size of the AOO area (76 km^2^) suggests an Endangered (EN) status.

##### Etymology.

This species is named for Mexican botanist Rafael Torres Colín (Rafa), an expert on the flora of Oaxaca, who led us into the field in 2017, when we attempted to locate this species.

##### Discussion.

*Lycianthes rafatorresii* is a Mexican endemic of the Caribbean slope of Oaxaca, Puebla, and Veracruz. Morphologically, it is close to *L. multiflora* Bitter of Central America (which does not occur in Guatemala or Mexico), and it has been misidentified as *L. sideroxyloides* (with which it overlaps in distribution) and *L. armentalis* (with which it does not overlap) (Nee 1986). It differs from *L. multiflora* in having straight to curved rays on the trichomes (rather than crisped rays in *L. multiflora*), having the inflorescences and fruits on leafy stems that are not restricted to the last few terminal sympodial units of the plant (rather than having flowers and fruits on mostly leafless terminal sympodia (so that they appear to be in a large panicle), having calyx appendages with a widened bulbous apex (rather than acute appendages), and having stellate corollas (rather than entire corollas). *Lycianthes rafatorresii* differs from *L. sideroxyloides* in lacking geminate-stellate trichomes, by having a corolla divided just half way to the base (or less) with abundant interpetalar tissue (versus a deeply stellate corolla with scant interpetalar tissue), by having unequal stamens (versus equal stamens), and by having fewer, larger mature fruits (8–15 mm in diameter versus 2–7 mm in diameter in *L. sideroxyloides*). It differs from *L. armentalis* in lacking widely divaricate branching, having appendages with a widened bulbous apex (versus acute appendages), having more seeds per fruit (40–100 in *L. rafatorresii* versus 20–30 in *L. armentalis*), occurring at higher elevations (to 1600 m in *L. rafatorresii* versus to 500 m in *L. armentalis*), and not occurring in the lowlands of the Yucatán Peninsula. More field study of *L. rafatorresii* is needed to better separate it from these similar species and understand its distribution, which currently does not appear to be continuous. Attempts by the first and third authors to locate this species in the field in Oaxaca in 2017 were unsuccessful.

##### Representative specimens examined.

(full list of paratypes in Appendix I). **Mexico. Oaxaca**: Dto. de Ixtlán, 3 km al S de Metates, carr. Tuxtepec-Oaxaca, [17.5872, -96.5065], no elevation, 10 Sep 1985, *R. Torres C. 7270* (MO). **Puebla**: Limonateno, [19.9110, -97.3076], 1000 m, 12 May 1970, *F. Ventura A. 1077* (IEB). **Veracruz**: Bastonal, 3–5 km adelante, camino a la Sierra de Santa Marta, 18.40, -94.95, 25 Nov 1985, *G. Castillo-Campos 4417* (XAL).

#### 40 Lycianthes rantonnetii

Taxon classificationPlantae

(Carrière) Bitter, Abh. Naturwiss. Verein Bremen 24 [preprint]: 332. 1919

Fig. 90

Solanum rantonnetii Carrière, Rev. Hort. [Paris] 32: 135. 1859, as rantonnei. Type: Illustration (Rev. Hort. [Paris] 32: fig. 32, page 135 (lectotype designated here). Orthographic variant correcting epithet to “*rantonnetii*” published by Lescuyer, Hort. Français, sér. II. i (9): 197. Late 1859 or 1860.
Solanum corniculatum Hiern, Vidensk. Meddel. Naturhist. Foren. Kjøbenhavn (1877–78): 45. 1877. Type: Brazil, Río de Janeiro, 1867. *A. Glaziou 1078* (lectotype designated here: C [10019192]; isolectotypes: BR [00000552267, 00000552234]).
Solanum urbanum Morong, Ann. New York Acad. Sci. 7: 177. 1893. Type: Paraguay. Asunción, streets of Asunción, Nov 1888, *T. Morong 147* (lectotype designated by Barboza 2013, pg. 29: NY [00172225]; isolectotypes: MO [acc. # 2495263], NDG [NDG45160], PH [00030498], US [00027839], WIS [v0004256WIS]).
Solanum muticum N.E.Br., Bull. Misc. Inform. Kew 85: 6. 1894. Type: Uruguay. Montevideo “Originaire du Paraguay, cultivé à Montevideo comme plante d’ornament,” Mar 1858, *Gibert 56* (lectotype designated by Barboza 2013, pg. 29: K [K000585755]).
Solanum urbanum Morong var. foliosum Chodat, Bull. Soc. Bot. Genève, ser. 2, 8: 152, fig. 47. 1916. Type: Paraguay. Cerros de Paraguarí, *R. Chodat & W. Vischer 60* (holotype: G [G00392293]).
Solanum urbanum Morong var. nervosum Chodat, Bull. Soc. Bot. Genève, ser. 2, 8: 152, fig. 47. 1916. Type: Paraguay. No exact location, Jan 1900, *É. Hassler 7024* (lectotype designated here: G [G00390048]; isolectotypes: G [G00392288, G00392285, G00392290], P [P03852955], W [acc. # 1904-804].
Solanum urbanum Morong var. subtomentosum Chodat, Bull. Soc. Bot. Genève, ser. 2, 8: 152, fig. 47. 1916. Type: Paraguay. Misiones: San Ignacio, Oct 1914, *R. Chodat & W. Vischer 61* (holotype: G [G00392295]).
Solanum urbanum Morong var. typicum Chodat, Bull. Soc. Bot. Genève, ser. 2, 8: 151. 1916. Nom. illeg. Type: Paraguay. Dans les jardines de l’Assumption, 9 Apr 1875, *B. Balansa 2104* (lectotype designated here: G [G00392296]; isolectotype: G [G00392297]).

##### Type.

Based on *Solanum rantonnetii* Carrière

**Figure 90.**
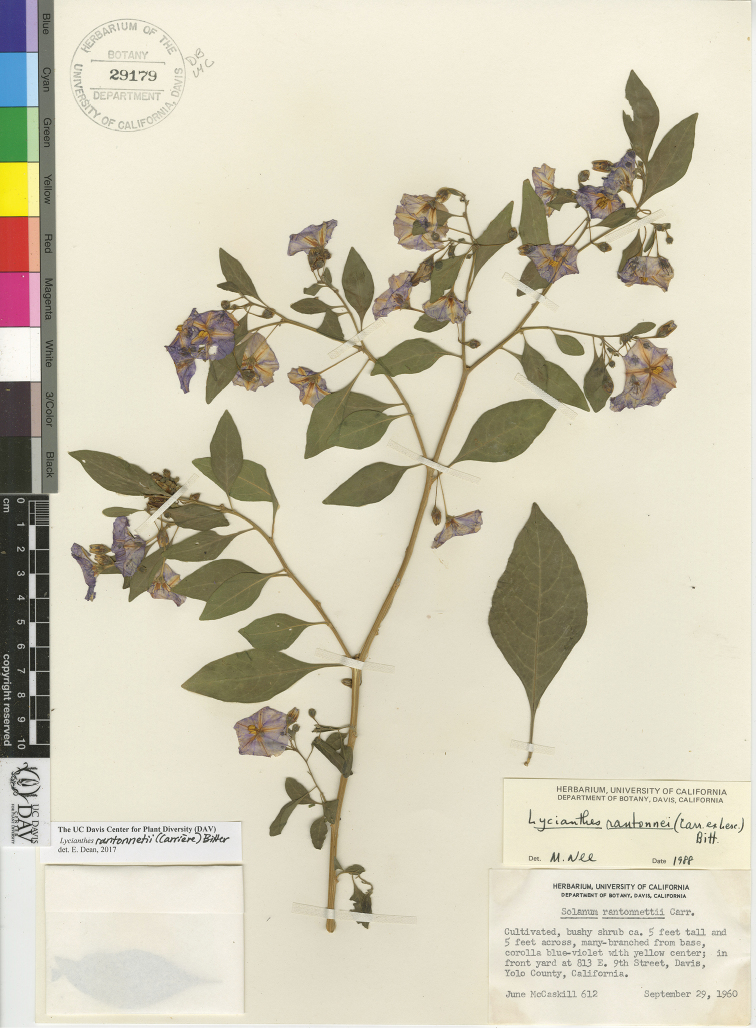
Image of herbarium specimen of *L. rantonnetii*, *McCaskill 612* (DAV). Image used with permission of the UC Davis Center for Plant Diversity.

##### Description.

Shrub, 1–3 (4) m tall, multiple stems emerging at the soil level. Indument of whitish, multicellular, simple, furcate, or dendritically branched, eglandular, spreading trichomes to 0.5 mm long, as well as sparse simple, glandular hairs. Stems green with yellow striations when young, sparsely to moderately pubescent, not compressed, but sometimes angled, upon drying in a plant press, brown and woody with angled ridges with age, becoming glabrate; upper sympodial branching usually dichasial, sometimes monochasial. Leaves simple, the leaves of the upper sympodia usually unpaired, when paired often equal in size, the blades 1–15.5 × 0.5–7.5 cm, widely ovate, elliptic, rhombic-lanceolate, or narrowly lanceolate, chartaceous, moderately pubescent, the base cuneate, attenuate into the petiole, sometimes oblique, the margin entire, irregularly undulate, the apex acute or acuminate (sometimes obtuse at the very tip), the petiole (0.1–) 0.8–2.5 (–4) cm long, winged toward the apex, the larger leaf blades with 3–7 primary veins on each side of the midvein. Flowers solitary or in groups of 2–7, axillary, erect to ascending; peduncles absent; pedicels slender, 5–25 mm long and erect to arching in flower, to 25 mm long (probably longer), arching to deflexed fruit, moderately pubescent; calyx 1.5–4 mm long, 2.5–4.5 mm in diameter, obconic to campanulate, puberulent, the margin truncate, with 5–10 spreading, linear-subulate appendages of two different lengths, the five longer appendages 2–5.2 mm long, emerging at the calyx rim, the five shorter appendages 0.25–2 mm long (these sometimes lacking), emerging ca. 0.25 mm below the calyx rim; fruiting calyx slightly enlarged, widely bowl- or plate-shaped, 3–5 mm long, 9–11 mm in diameter, the appendages not elongating, often withering; corolla 1–1.2 (2) cm long, rotate in orientation, mostly entire in outline (with shallow notches), with abundant interpetalar tissue, adaxially deep violet-purple with yellow center, glabrous, abaxially deep violet-purple, glabrous; stamens unequal, curved, the two short filaments 0.8–1.5 mm long, the three long filaments 2–3 mm long, sparsely to densely pubescent on inner face at juncture with corolla, the anthers 2.5–4 mm long, elliptic to oblong, free of one another, yellow to orange, glabrous, poricidal at the tips, the pores opening horizontally, dehiscing toward the style, not opening into longitudinal slits; pistil with glabrous ovary, the style 3.5–5.5 mm long, linear, slightly curved, widening near the stigma, glabrous, the stigma truncate, slightly bilobed. Fruit a berry, 10–20 (35) mm long, 8–15 (35) mm in diameter, subglobose to ellipsoid, green (sometimes with dark lines when immature), yellow to orange when mature, glabrous, with abundant (often more than 20) tan, irregularly shaped sclerotic granules, 1–2 (3) mm long, these sometimes attached to the seeds. Seeds ca. 3–12 per fruit in horticultural plants, possibly more in native habitat, (1.75) 2.25–3.5 × (1.5) 3–3.3 mm, compressed but not flat, round elliptic to reniform in outline, dark brown, the surface reticulum with loose serpentine pattern with deep luminae.

##### Chromosome number.

2n = 24, Gerasimenko and Reznikova 1968, cited in D’Arcy (1974). Acosta et al. (2005).

##### Distribution and habitat.

Native to South America (Argentina, Bolivia, Paraguay, and southern Brazil) in thickets and woodlands, weedy in disturbed areas along roadsides, 100–2000 m in elevation. Horticulturally, widely distributed worldwide, including Mexico and Guatemala (no distribution map completed for this species).

##### Common names and uses.

Argentina. Meloncillo del aire (Barboza and Hunziker 1992).

##### Phenology.

This species flowers most of the year in cultivation and may be similar in its native habitat. Fruiting period not known. Corollas open in the morning, closing by late afternoon or evening.

##### Preliminary conservation status.

As this species is a horticultural plant in our floristic region, we are not providing a conservation assessment.

##### Discussion.

*Lycianthes rantonnetii* is a popular horticultural plant, often flowering prolifically throughout the year (depending on climate) and dying back to near the ground in cold temperatures. The species is widely planted in Mexico and California. Viable seeds are produced in Mexican plants, but in California, the fruits are often sterile, without viable seeds; it has been documented as persisting in the wild in southern California, but it is unknown whether viable seeds are being produced (Dean in press). The species was named for M. Victor Rantonnet, 19^th^ century French horticulturist of Hyères, a town on the Mediterranean coast of southern France (Carrière 1859). The spelling of the epithet was first published as “*rantonnei*” in March of 1859 (Carrière 1859) and changed to *rantonnetii* by Lescuyer in the later months of 1859 (D’Arcy 1974). Retaining the spelling as *L. rantonnetii* was upheld by recent editors of the International Plants Name Index citing Art. 60.7 of the International Code of Botanical Nomenclature (Shaw and Shaw 2004).

In the protologue, Carrière (1859) described *Solanum rantonnetii* from horticultural material grown in Hyères. He says that the seeds were brought to France in ca. 1849 by a naval officer from the region of La Plata [assumed to be Argentina], and after being grown at Toulon, plants were then shared with Rantonnet. No herbarium specimens were cited by Carrière, but the protologue does have a detailed illustration (Carrière 1859, fig. 32, pg. 135) which can serve as a type, and we are lectotypifying that illustration here.

The protologue for *Solanum corniculatum* is part of an article edited by Warming but published by Hiern (a British botanist) on the Solanaceae of Brazil (Hiern 1877). The protologue cites one collection (*Glazou 1078*) but does not mention where the collection was seen or deposited. We located one duplicate of *Glazou 1078* at C [10019192] and two at BR [00000552267, 00000552234]. The specimen at C is from the Warming Herbarium. Those at BR are from the Martius herbarium. Therefore, the duplicate from the Warming Herbarium at C was chosen as the lectotype: C [10019192].

In his protologue for Solanum urbanum Morong var. nervosum, Chodat and Vischer (1916) cite three collections from Paraguay which we located at G: *É. Hassler 7024* [G00390048, G00392288, G00392285, G00392290], *B. Balansa 2121* [G00390049], and *É. Hassler 6728* [G00392289]. From these specimens we chose a duplicate of *É. Hassler 7024* [G00390048] as the lectotype.

In his protologue for Solanum urbanum Morong var. typicum, Chodat and Vischer (1916) cite one collection from Paraguay at G: *B. Balansa 2104*. We located two duplicate syntypes of the collection at G [G00392296, G00392297]. From these we chose G00392296 as the lectotype.

#### 41 Lycianthes rzedowskii

Taxon classificationPlantae

E.Dean, Novon 4: 327, 1994

Fig. 91

##### Type.

México. Michoacán: Mpio. Charo, along hwy 15, 20 rd km E of Morelia, just E of Pontezuelas, 2165 m, 13 Nov 1991, *E. Dean 322a* (holotype: UC [UC1797879]; isotypes: DAV [DAV158015], NY [00687931], XAL [XAL0106679]).

**Figure 91.**
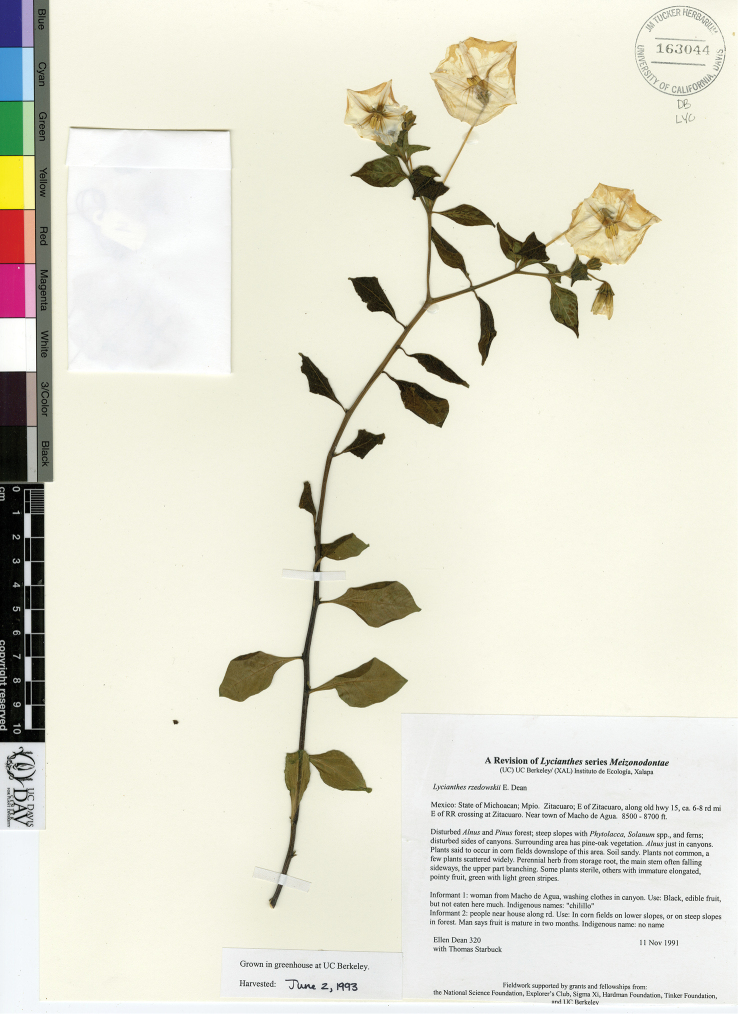
Image of herbarium specimen of *L. rzedowskii*, *Dean 320* (DAV). Image used with permission of the UC Davis Center for Plant Diversity.

##### Description.

Perennial herb from fusiform storage roots, usually erect, often recumbent with age, 0.17–1.1 m tall, dying back each season. Indument of white, uniseriate, multicellular, simple or dendritically branched, eglandular, spreading to appressed, sometimes crisped, trichomes, 0.1–1.5 (2) mm long. Stems green to reddish-purple, glabrous to sparsely pubescent, usually compressed and ribbed when dried in a plant press, somewhat woody with age, especially at base of plant; first stem 7–90 cm long to first inflorescence, the internodes (6) 10–21; first two sympodial branching points usually dichasial, usually followed by monochasial branching, this branching usually limited. Leaves simple, those of the upper sympodia usually paired and unequal in size, the larger ones with blades (2.5) 5–10 (15) × 1–6 cm, the smaller ones with blades 1/4 to 3/4 the size of the larger, the leaf pairs similar in shape, the blades ovate, elliptic, or obovate, chartaceous, sparsely to moderately pubescent, the primary veins 5–7 on either side of the midvein, the base truncate or cuneate, attenuate onto the petiole, sometimes oblique, the margin entire, usually irregularly undulate, the apex acuminate, the petioles of larger leaves winged and poorly defined, 0.1–2.8 cm long, sometimes absent. Flowers solitary, axillary, oriented horizontally, and somewhat nodding; peduncles absent; pedicels 19–86 mm and erect in flower, 30–110 mm long and deflexed in fruit, glabrous to moderately pubescent; calyx 3–6 mm long, 3–6.5 mm in diameter, campanulate, glabrous to moderately pubescent, the margin truncate, with 10 knob-like to linear, slightly spreading or erect appendages 1–7.25 mm long emerging ca. 1 mm below the calyx rim; fruiting calyx enlarged, 2–10 mm long, 5–14.5 mm in diameter, the appendages stout, stiff, remaining appressed to fruit or somewhat spreading, often broken, 1–7.5 mm long; corolla 1–2.5 cm long (2–4.7 cm in diameter), rotate in orientation, mostly entire in outline (with shallow notches), with abundant interpetalar tissue, white (rarely tinged lilac), with violet stripes near the major veins adaxially, green near the major veins abaxially, usually glabrous; stamens unequal, the filaments of three lengths, the two shortest filaments 1–4 mm long, the two medium filaments 2–4 mm long, the one long filament 2.5–6.25 mm long, the length of the long filament always less than 2 times that of the medium filaments, glabrous, the anthers 3–5.5 mm long, lanceolate to elliptic, free of one another, yellow, glabrous, poricidal at the tips, the pores round to oval, dehiscing distally, not opening into longitudinal slits; pollen grains dicolporate; pistil with glabrous ovary, the style 7–11 mm, linear, straight to slightly curved, the stigma round, rarely somewhat lobed. Fruit a berry, usually remaining attached to calyx at maturity, pendent, 19–75 mm long, 8–19 mm in diameter, turbinate, elongate, the exocarp dull light purple to black at maturity (green with light or dark longitudinal lines when immature), glabrous, the mesocarp dark purple, soft and juicy, lacking sclerotic granules, the placental area light purple, powdery in texture. Seeds 3–38 per fruit, 3.5–5 × 3.2–4.7 mm, not compressed, depressed obovate, ridged and blistered along one side, black in color, the surface reticulum rough in texture with loose serpentine pattern with deep luminae.

##### Chromosome number.

2n = 24, *Dean 212, 317, 336* (Dean 2004).

##### Distribution and habitat.

**Figure 92.**
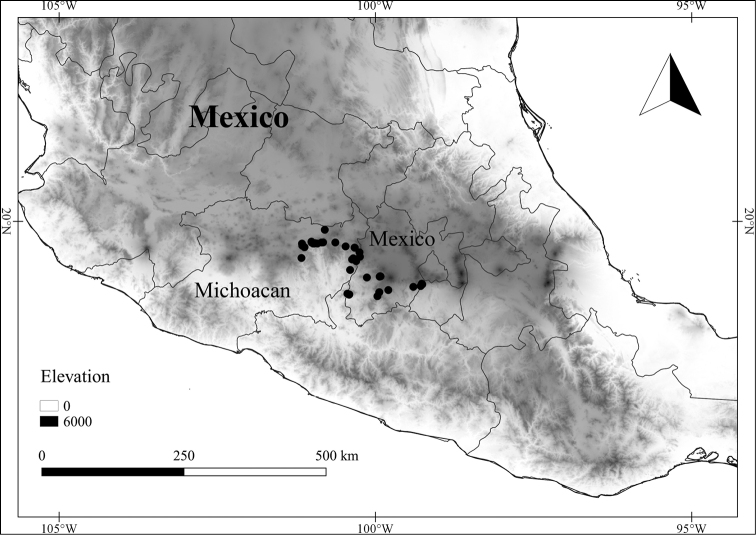
Map of geographic distribution of *L. rzedowskii* based on herbarium specimen data.

Mexico (México, Michoacán, Morelos) in oak, oak-pine, pine-oak, and fir forests, often on forested slopes near drainages, on volcanic soils, 1794–2645 m in elevation (Fig. 92).

##### Common names and uses.

Mexico. Chilillo (Dean 2004).

##### Phenology.

Flowering specimens have been collected June to October; specimens with mature fruits have been collected in late November and December. The first author has observed in the field that the corollas open in very early morning and closed in the late morning. The pollen of this species has a sweet, powdery fragrance.

##### Preliminary conservation status.

*Lycianthes rzedowskii* is a locally common species of central/western Mexico, represented by 43 collections and occurring in six protected areas. Unfortunately, the habitat of this species is vulnerable due to urban development. The conservation status of this species was assessed by Anguiano-Constante et al. (2018) and their preliminary assessment was Least Concern. The EOO is 24,120.227 km^2^, and the AOO is 160 km^2^. Based on the IUCN (2019) criteria, the preliminary assessment category is now Endangered (EN).

##### Discussion.

*Lycianthes rzedowskii* may be confused with *L. acapulcensis* and *L. ciliolata*. It can be distinguished from those species by the many internodes on its first stem, the usually poorly developed sympodial branching in flowering plants, smooth, white (rarely pale lilac) corollas, and broad sweetly scented anthers with terminal, round anther pores. In addition, one of the best ways to distinguish *L. rzedowskii* from the other two species is to look at the relative lengths of the stamen filaments. In *L. rzedowskii* the length of the longest filament is never more than twice that of the medium-short filaments, while in the other two species, the length of the longest filament is almost always more than twice that of the medium-short. *Lycianthes rzedowskii* may hybridize with *L. acapulcensis* and *L. starbuckii* E.Dean where they occur together (Dean 2004).

##### Representative specimens examined.

**Mexico. México**: Avandaro, Cerro Gordo, 19.1142, -100.1341, 2301 m, 22 Jun 2011, *L. Corral 1801* (MEXU). **Michoacán**: San Miguel Chichimequillas La Mesa, 19.4065, -100.3631, 2018 m, 15 Jul 2007, *L. Corral 376* (MEXU). **Morelos**: Sierra de Morelos, Cuernavaca, [18.9794, -99.2841], 2050 m, 26 Jul 1969, *Hinton 17221* (NY).

#### 42 Lycianthes scandens

Taxon classificationPlantae

(Mill.) M. Nee comb. nov.

urn:lsid:ipni.org:names:77213137-1

Solanum scandens Mill., Gard. Dict. ed. 8, no. 19. 1768. Type: Mexico. Vera Cruce [Veracruz], 1730, *W. Houstoun s.n.* (holotype: BM [BM000514911].

##### Type.

Based on *Solanum scandens* Mill.

#### 42a Lycianthes scandens var. scandens

Taxon classificationPlantae

Fig. 93

Solanum lentum Cav., Icon. [Cavanilles] 4: 4, tab. 308. 1797. Type: Spain. ex h. r. Mat., Oct 1795, *A. Cavanilles s.n.* (lectotype designated by Knapp 2007, pg. 198: MA [MA 476355]).
Solanum axilliflorum Dunal, Hist. Solan. 238. 1813. Nom. Illeg. New name for Solanum scandens Mill. Type: Based on same type as Solanum scandens Mill.
Solanum affine Dunal, in Prodr. [A. P. de Candolle] 13(1): 168. 1852. Type: Cuba, 1829, *R. de la Sagra 231* (lectotype designated here: G-DC [G00145629]).
Solanum fugax Bert. ex Dunal, in Prodr. [A. P. de Candolle] 13(1): 170. 1852. Type: Naranjilla en S. Martha, 1822, *C. Bertero s.n.* (holotype: G-DC [G00145637]).
Solanum neglectum Bert. ex Dunal, in Prodr. [A. P. de Candolle] 13(1): 170. 1852. Type: [Colombia]. Santa Martha, 1822, *C. Bertero s.n.* (holotype: G-DC [G00145631]).
Solanum decemfidum Pav. ex Dunal, Prodr. [A. P. de Candolle] 13(1): 173. 1852. Type: Nueva España [Mexico]. No date, *Herb. Pavón s.n.* (holotype: G [G00379128]).
S. lentum Cav. var. echinatum Dunal, Prodr. [A. P. de Candolle] 13(1): 173. 1852. Type: Nueva España [Mexico]. No date, *Herb. Pavón s.n.* (holotype: G [G00379129]).
Solanum nocturnum Fernald, Proc. Amer. Acad. Arts 35: 570. 1900. Type: Mexico. Guerrero: Acapulco, Jan 1895, *E. Palmer 533* (lectotype designated here: GH [00077521]; isolectotype: US [00027708]).
Solanum virgatum Lam. var. lentum (Cav.) O. Schulz, in Urb., Symb. Antill. (Urban) 6: 189. 1909. Type: Based on Solanum lentum Cav.
Lycianthes lenta (Cav.) Bitter, Abh. Naturwiss. Verein Bremen 24 [preprint]: 364. 1919. Type: Based on Solanum lentum Cav.
Lycianthes lenta (Cav.) Bitter var. utrinquemollis Bitter, Abh. Naturwiss. Verein Bremen 24 [preprint]: 366. 1919. Type: Guatemala [Nicaragua]. Grenada [Granada], 1841, *E. Von Friedrichsthal 940* (lectotype designated here: W [acc. # 0003076]; isolectotype: F [barcode F0072916F, acc. # 875961]).
Lycianthes nocturna (Fernald) Bitter, Abh. Naturwiss. Verein Bremen 24 [preprint]: 368. 1919. Type: Based on Solanum nocturnum Fernald.
Lycianthes recticarpa Rusby, Bull. Torrey Bot. Club 53: 210. 1926. Type: Colombia. Santa Marta: Quebrada del Cabo, 100 ft, 26 Aug 1876, *H. H. Smith 1876* (holotype: NY [00007274]).
Lycianthes variifolia Standl., Publ. Field Mus. Nat. Hist. Chicago, Bot. Ser. 4: 259. 1929. Type: Belize. [Orange Walk or Corozal]: Tower Hill, 1928, *J. Karling 13* (holotype F [acc. # 579929]; isotype G [G00379131]).

##### Description.

Scandent shrub to vine, 0.5–3 (6) m tall. Indument of white to tan, uniseriate, multicellular, sessile to stalked, forked to multangulate-stellate, eglandular, spreading trichomes 0.05–0.25 (0.7) mm long and in diameter, the rays 3–4 per node, straight, not rebranched (sometimes mixed with simple trichomes). Stems green to light brown when young, sparsely to densely pubescent, rarely compressed when dried in a plant press, becoming woody with age; upper sympodial branching points mostly monochasial, the branching not widely divaricate, the branch segments shallowly zigzagging or sinuate. Leaves simple, the leaves of the upper sympodia sometimes paired, the pairs equal or unequal in size, the larger ones with blades 2.5–10 (11.2) × 1.6–5 (8.4) cm, the smaller ones with blades 1.6–7 (10.5) × 0.9–3.4 (7.5) cm, the leaf pairs similar in shape, the blades ovate to elliptic, chartaceous, sparsely to moderately pubescent (denser abaxially, sometimes nearly glabrous adaxially), the base cuneate, rounded, or truncate, sometimes oblique, the margin entire, usually irregularly undulate, the apex usually rounded to acute, the petiole 0.3–2 (2.6) cm long, the larger leaf blades with 3–5 primary veins on each side of the midvein, these not usually whitish or prominent. Flowers solitary or in groups of 2–6, axillary, erect; peduncles absent; pedicels 8–20 (26) mm long and erect in flower, 10–26 (30) mm long and erect in fruit, sparsely to moderately pubescent; calyx 2.5–4 mm long, 3–6 mm in diameter, campanulate, sparsely pubescent (the individual trichomes often difficult to see with the naked eye, up to 0.1 mm long), the margin truncate, with 10 spreading linear appendages (0.75) 1–3.5 (5) mm long emerging 0.5–1 mm below the calyx rim; fruiting calyx enlarged, widely bowl-shaped to rotate, 3–4 mm long, 6–9 mm in diameter, the appendages to 9 mm; corolla 0.7–1.9 cm long, campanulate to rotate in orientation, entire in outline, with abundant interpetalar tissue, adaxially light purple, sometimes with green at the base of the lobes, nearly glabrous, abaxially green and sparsely to moderately puberulent on the lobes, especially at the distal end, this more evident in bud; stamens unequal, the four short filaments 0.5–1 (2) mm long, the one long filament (1) 2–4 (6.5) mm long, glabrous, the anthers 4–6 mm long, narrowly ovate to lanceolate, free of one another, yellow, sparsely pubescent on the inner face, poricidal at the tips, the pores ovate, dehiscing distally or the short stamens dehiscing away from the style, not opening into longitudinal slits; pistil with glabrous ovary, the style 8.5–9 (11) mm long, linear, straight to curved, glabrous, the stigma oblong, decurrent down two sides. Fruit a berry, 6–14 mm long, 7–17 mm in diameter, globose to depressed globose, red-orange at maturity, glabrous to sparsely pubescent, lacking sclerotic granules. Seeds 50–70 per fruit, 2–3 × 1.8–2 mm, flattened, circular, triangular, depressed ovate, or slightly notched and reniform in outline, yellow-orange to tan, the surface reticulum smooth, with indistinct, tight serpentine pattern with shallow luminae, the seed margin slightly thickened and lighter in color.

**Figure 93.**
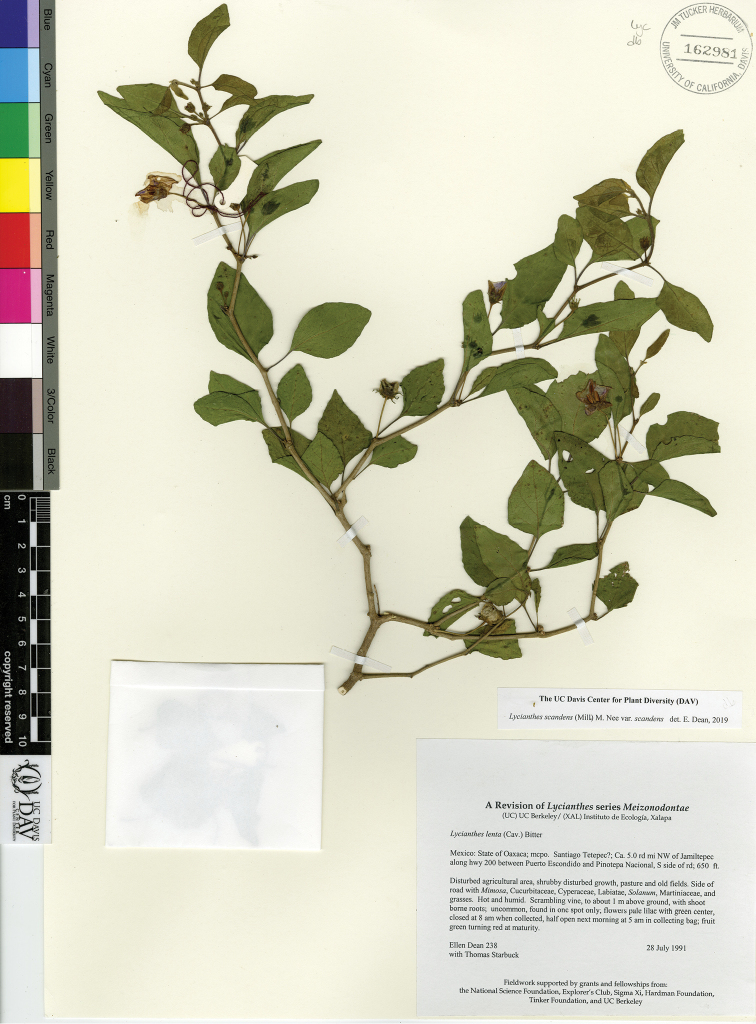
Image of herbarium specimen of L. scandens var. scandens, *Dean 238* (DAV). Image used with permission of the UC Davis Center for Plant Diversity.

##### Chromosome number.

Unknown.

##### Distribution and habitat.

**Figure 94.**
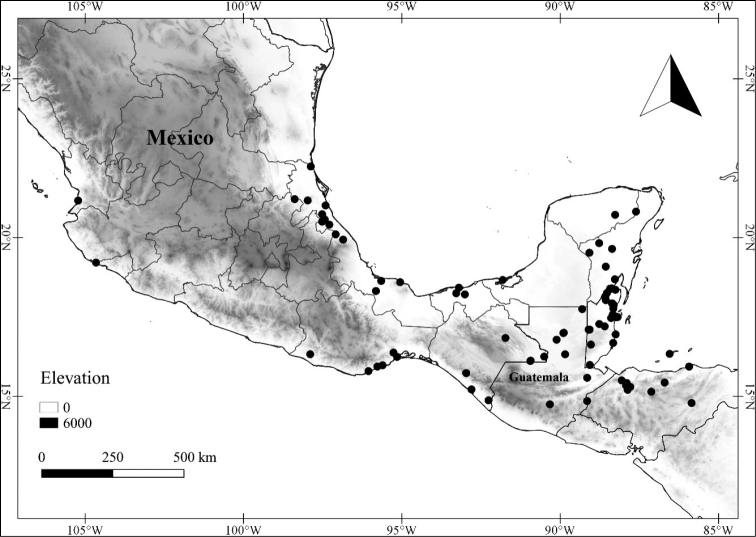
Map of geographic distribution of L. scandens var. scandens from Mexico to Honduras based on herbarium specimen data.

Mexico (Campeche, Chiapas, Jalisco, Nayarit, Oaxaca, Quintana Roo, Tabasco, Tamaulipas, Veracruz, Yucatán), Guatemala (Chiquimula, Izabal, Petén, Sacatepéquez), Belize, Honduras, the Caribbean, and northern South America, in open or disturbed areas, roadsides, fencerows, thickets, dunes or forest edges, sometimes seasonally flooded (in mangroves), sometimes on limestone, within or near tropical dry forest, 0–400 (915) m in elevation (Fig. 94).

##### Common names and uses.

None known.

##### Phenology.

Flowering specimens have been collected most months of the year (from January to November); specimens with mature fruits have been collected all months of the year. The first author observed the corolla open in the field between 5 am and 5:30 am. The corollas closed at 8 am.

##### Preliminary conservation status.

Lycianthes scandens var. scandens is a widespread variety ranging from Mexico to the Caribbean and northern South America, represented by many collections and occurring in ten protected areas in our region. The EOO is at least 1,162,412.966 km^2^. Based on the size of the EOO, and following the IUCN (2019) criteria, the preliminary assessment category is Least Concern (LC).

##### Discussion.

In this treatment, we are replacing the widely used name *L. lenta* with the new combination L. scandens var. scandens; this name change is due to the discovery of an older name for this taxon made during relatively recent work on the genus *Solanum* by the fourth author. Lycianthes scandens var. scandens is distinguished from the less common var. flavicans by having smaller sparser trichomes (usually less than 0.25 mm in diameter versus more than 0.25 mm in diameter in var. flavicans); the trichomes on the calyx are often so small that they cannot be seen with the naked eye. In addition, the leaf veins are obscure in var. scandens (versus prominent and whitish in var. flavicans), the corolla is purple (versus pale violet to white in var. flavicans), and the habit is usually a trailing vine (often an upright shrub in var. flavicans). This variety is also found at lower elevations (usually below 500 m) than var. flavicans (to 1300 m), and it is very common along the Caribbean slope of Mexico and Central America, while var. flavicans is more common along the Pacific slope. The two varieties can be difficult to distinguish in certain regions.

In his protologue for *Solanum affine*, Dunal (1852) cites two syntypes from the De Candolle herbarium, both of which were located and examined at G: *R. de la Sagre 231* (G-DC [G00145629]) and *R. de la Sagra 394* (G-DC [G00145619]). Both specimens match the protologue description, but only the *R. de la Sagre 231* specimen has location information (Cuba), and it has better vegetative and fertile material. Therefore, we have chosen *R. de la Sagre 231* (G-DC [G00145629]) as the lectotype.

In his protologue for *Solanum nocturnum*, Fernald (1900), cites two syntypes both of which have been located and examined at GH: *E. Palmer 533* from Guerrero, Mexico (GH [00077521]) and *E. Seler 1625* from Oaxaca, Mexico (GH [00077522]). As the *Palmer 533* collection has both flowers and fruits and matches the protologue description more fully, we have chosen the GH specimen of *Palmer 533* (GH [00077521]) as the lectotype.

In his protologue for Lycianthes lenta var. utrinquemollis, Bitter (1919) cites two syntypes both from Grenada [Granada], Nicaragua, and these have been located and examined at W and G: *E. von Friedrichsthal 940* (W [acc. # 0003076]) and *P. Levy 239* (G [G00379125]). We have chosen the W specimen of *E. von Friedrichsthal 940* (W [acc. # 0003076]) as the lectotype.

##### Representative specimens examined.

**Guatemala. Chiquimula**: vicinity of Chiquimula town, 16.1227, -90.9299, 400 m, 4 Dec 1969, *A. Molina R. 25111* (NY). **Izabal**: Finca Mucielago [Murciélago], 22 Jun 1967, *S.C. Snedaker 178* (NY). **Petén**: NW-Ufer des Lago Petén Itzá, 16.9917, -89.8983, 120–140 m, 30 Nov 1994, *B. Wallnöfer 9577* (NY). **Sacatepéquez**: Agua Caliente, 28 Mar 1922, *J.M. Greenman 5944* (MO). **Mexico. Campeche**: en la Ciudad del Carmen, sobre Avenida Camarón, [18.6632, -91.8145], 23 Nov 1987, *E. Cabrera C. 14856* (MEXU, MO). **Chiapas**: Mpio. Ocosingo, Estación Chajul, sobre el Río Lacantun, [16.1227, -90.9300], 150 m, 9 Sep 1992, *E. Martínez S. 25292* (MEXU). **Jalisco**: Arroyo Seco, [19.211, -104.6577], 30 m, 28 May 1990, *A. Rodríguez 2077* (WIS). **Nayarit**: vicinity of Chacala, ca. 5 miles west of Las Varas, 25–50 m, 14 Sep 1960, *R. McVaugh 19019* (NY). **Oaxaca**: Dto. Tehuantepec, Hacienda del Rosario, Río Seco, 15.9367, -95.7719, 33 m, 22 Aug 2010, *R. García S. (ROG) 226* (DAV). **Quintana Roo**: Mpio. José María Morelos, a 3.4 km al SE de Sabana San Francisco, 19.52, -89.075, 86 m, 3 Sep 2004, *D. Álvarez 10473* (DAV). **Tabasco**: Mpio. Comalcalco, Reyes Hernández, [18.2445, -93.2829], 0 m, 10 Sep 1984, *F. Ventura A. 21240* (NY). **Tamaulipas**: vicinity of Tampico, 15 m, 27–30 Apr 1910, *E. Palmer 334* (MO, NY). **Veracruz**: vicinity of Pozarica, [20.5669, -97.4332], 6 June 1987, *T.B. Croat 66115* (NY). **Yucatán**: Pixoy, camino rumbo a San Lorenzo, Valladolid, 20.7147, -88.2625, 22 m, 13 Jul 1988, *G. Remmers 30* (MO).

#### 42b Lycianthes scandens (Mill.) M.Nee var. flavicans

Taxon classificationPlantae

(Bitter) J.Poore & E.Dean comb. nov.

urn:lsid:ipni.org:names:77213138-1

Figs 95 , 96

Solanum lambii Fernald, Botanical Gazette 20: 536. 1895. Type: Mexico. Sinaloa: Villa Union, Jan 1895, *F. Lamb 446* (holotype: GH [00077501]; isotypes: CAS [0004496], G [G00442761], K [K000063068], MSC [MSC0092874], NDG [NDG45061], NY [00138999], US [00027643]).
Lycianthes lenta (Cav.) Bitter var. flavicans Bitter, Abh. Naturwiss. Verein Bremen 24 [preprint]: 366. 1919. Type: Mexico. Jalisco: Tequila, Mar 184X (year is missing the last digit), *H. Galeotti 1225F* (holotype: BR [000000563130]; isotypes: NY [00138704], US [01107998]).
Lycianthes lenta (Cav.) Bitter ssp. lambii (Fernald) Bitter, Abh. Naturwiss. Verein Bremen 24 [preprint]: 368. 1919. Type: Based on Solanum lambii Fernald.

##### Type.

Based on Lycianthes lenta (Cav.) Bitter var. flavicans Bitter.

**Figure 95.**
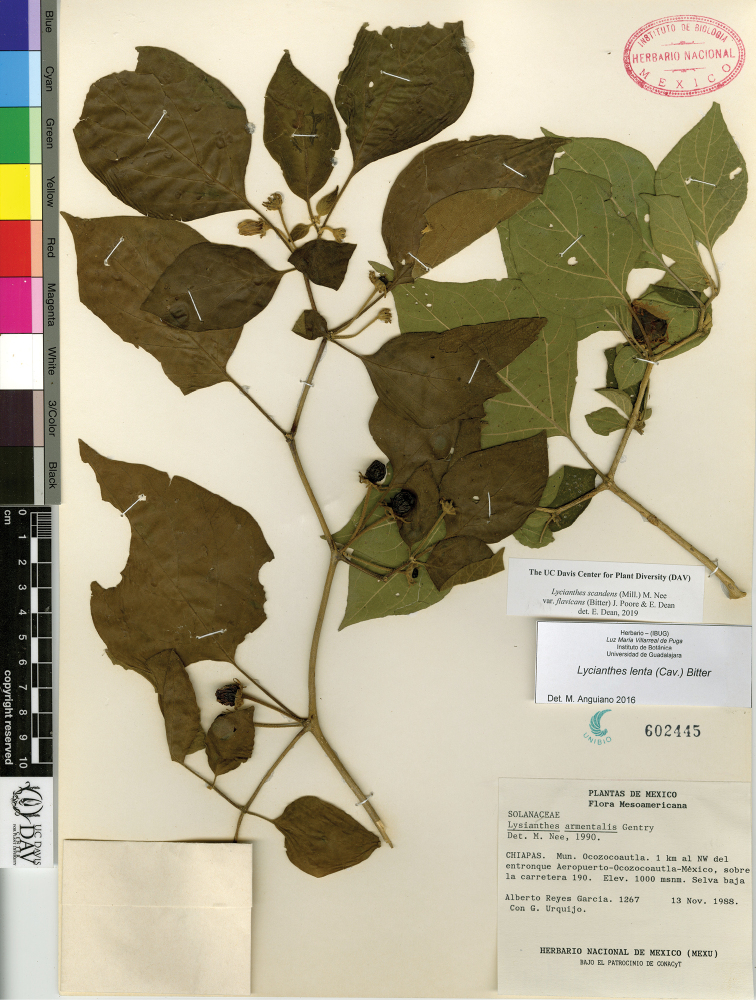
Image of herbarium specimen of L. scandens var. flavicans, *Reyes-Garcia 1267* (MEXU). Specimen used with permission from the Herbario Nacional de México, Universidad Autónoma de México.

##### Description.

Clambering or erect shrub, sometimes a vine, 1–4 m tall. Indument of white to tan, uniseriate, multicellular, sessile to stalked, furcate, multangulate-stellate, and geminate-stellate, eglandular, spreading trichomes 0.05–0.75 mm long and 0.2–0.75 in diameter, the rays of the stellate trichomes 3–5 per whorl, straight, rarely rebranched. Stems green to light brown when young, sparsely to densely pubescent, not compressed when dried in a plant press, becoming woody with age; upper sympodial branching points dichasial or monochasial, the branching not widely divaricate, the segments shallowly zigzagging. Leaves simple, the leaves of the upper sympodia usually paired, the pairs equal or unequal in size, the larger ones with blades (2.3) 4–13.2 × (1.5) 3–9 cm, the smaller ones with blades (1.3) 2.7–7.5 × (0.7) 1.2–5 cm, the leaf pairs similar in shape, the blades ovate to elliptic, chartaceous, moderately to densely pubescent (denser abaxially, sometimes nearly glabrous adaxially), the base cuneate, rounded, or truncate, sometimes oblique, the margin entire, usually irregularly undulate, the apex acute to short acuminate, the petiole 0.3–5.4 (6) cm long, the larger leaf blades with 3–5 primary veins on each side of the midvein, these usually pale in color and prominent. Flowers solitary or in groups of 2–7, axillary, erect; peduncles absent; pedicels 7–31 mm long and erect in flower, 8–38 mm long and erect in fruit, moderately to densely pubescent; calyx 2.5–4.5 mm long, 3–7 mm in diameter, campanulate, moderately to densely pubescent (the surface sometimes obscured), the margin truncate, with 10 spreading linear appendages 1.5–6 (8) mm long emerging 0.5–1.0 mm below the calyx rim; fruiting calyx enlarged, widely bowl-shaped to rotate, 2.5–4.5 mm long, 7–12 mm in diameter, the appendages to 9 mm; corolla 1–1.8 (2.1) cm long, campanulate to rotate in orientation, entire in outline, with abundant interpetalar tissue, adaxially white to pale lavender and nearly glabrous, abaxially green and sparsely to moderately puberulent on the lobes, especially at the distal end, this more evident in bud; stamens unequal, the four short filaments 1–3 mm long, the one long filament 3–7 mm long, glabrous, the anthers 4–5.5 mm long, narrowly ovate to lanceolate, free of one another, yellow, very sparsely pubescent on the inner face, poricidal at the tips, the pores ovate, dehiscing distally or the short stamens dehiscing away from the style, not opening into longitudinal slits; pistil with glabrous ovary, the style 7–12 mm long, linear, straight to curved, glabrous, the stigma oblong, decurrent down two sides. Fruit a berry, 7–17 mm long, 8–17 mm diameter, usually globose to depressed globose, red-orange at maturity, glabrous to very sparsely pubescent, lacking sclerotic granules. Seeds 40–80 per fruit, 2–3 × 1.5–2.5 mm, flattened, circular, triangular, depressed ovate, or very slightly notched in outline, yellow to yellow-orange, the surface reticulum with tight serpentine pattern with shallow lumina, the seed margin slightly thickened, lighter or darker in color.

##### Chromosome number.

Unknown.

##### Distribution and habitat.

Mexico (Chiapas, Colima, Jalisco, Michoacán, Oaxaca, Sinaloa), Guatemala (El Progreso, Huehuetenango, Zacapa), El Salvador, Honduras, Nicaragua, and Costa Rica, in oak forest, pine forest, and tropical dry forest, in open disturbed areas, riparian areas, road edges, canyons and ravines, thickets and hedges, sometimes on limestone, 0–1300 m in elevation (Fig. 97).

**Figure 96.**
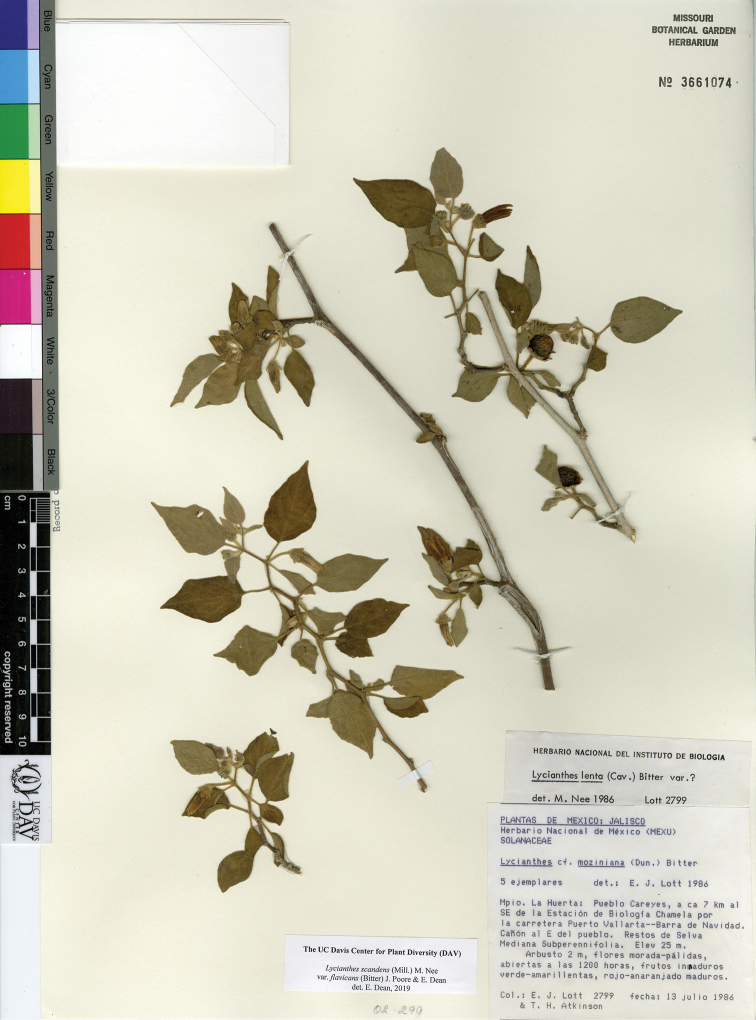
Image of herbarium specimen of L. scandens var. flavicans, *Lott 2799* (MO). Specimen used with permission from the Missouri Botanical Garden (http://www.tropicos.org).

**Figure 97.**
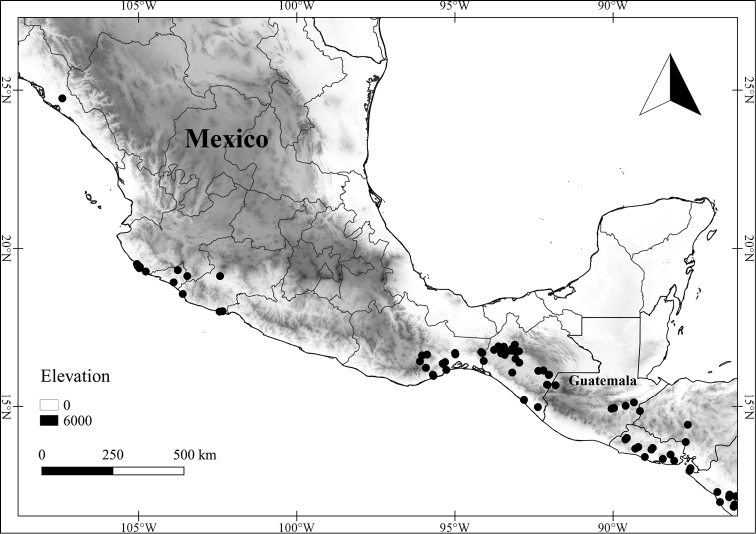
Map of geographic distribution of L. scandens var. flavicans from Mexico to Honduras based on herbarium specimen data.

##### Common names and uses.

None known.

##### Phenology.

Flowering specimens and specimens with mature fruits have been collected all months of the year. The corollas of this variety have not been observed in the field by the authors, but it is probable that the diurnal movements are similar to those of var. scandens, opening and closing in the very early morning.

##### Preliminary conservation status.

Lycianthes scandens var. flavicans is a widespread variety of western Mexico and Central America, represented by 111 collections and occurring in four protected areas. The EOO is 1,500,273.062 km^2^, and the AOO is 408 km^2^. Based on the IUCN (2019) criteria, the preliminary assessment category is Least Concern (LC).

##### Discussion.

Lycianthes scandens var. flavicans is distinguished from the more common var. scandens by having larger and denser trichomes (usually greater than 0.25 mm in diameter versus less than 0.25 mm in diameter in var. scandens) which often make the calyx look woolly; in addition, the leaf veins are often prominent and white in color (versus obscure in var. scandens), the corolla is often white (versus purple in var. scandens), and the habit is often more of a shrub than a trailing vine (the common habit in var. scandens). This variety is also found at higher elevations (to 1300 m) than var. scandens (usually less than 500 m), and it is usually along the Pacific slope of Mexico and Central America, while var. scandens is more common along the Caribbean slope. The two varieties can be difficult to distinguish in certain regions, especially Nicaragua.

The type collection from Jalisco, Mexico upon which this variety is based (*H. Galeotti 1225F*) has very small leaves, a form which is found in certain areas of the Pacific slope of Mexico. Other populations have much larger leaves. To illustrate this morphological variation, we have provided two figures (Figs 93, 94) for this taxon.

##### Representative specimens examined.

**Guatemala. El Progreso**: Tulumaje, [14.9286, -90.0313], 346 m, 23 Oct 2003, *R. Avila 71* (MO, MEXU). **Huehuetenango**: Between Santa Ana Huista and woods of Rancho Lucas, Sierra de los Cuchumatanes, 800–900 m, 26 Aug 1942, *J.A. Steyermark 51365* (MO1294998). **Zacapan**: San Agustín Acasaguastlan, [14.9480, -89.9647], 300 m, 8 Feb 2003, *F. Ramírez 254* (MEXU). **Mexico. Chiapas**: roadside on road MEX190 from Tuxtla Gutiérrez to San Fernando, about 8 km NW from the center of Tuxtla Gutiérrez (in a straight line), 16.7944, -93.1836, 733 m, 29 Nov 2012, *L. Bohs 3920* (DAV, MEXU). **Colima**: 17–18 km al NW de Colima; 1 km al sur de Campo Cuatro, [19.3138, -103.7604], 1300 m, 15 Aug 1991, *F.J. Santana Michael 5281* (MEXU). **Jalisco**: Rancho Cuixmala, along the Río Cuitzmala near the ranch headquarters, from the road crossing to Zapata downstream about 1 km, [19.38, -104.98], 10 m, 16 Mar 1991, *A. Sanders 10509* (MO, NY). **Michoacán**: Cuatrocaminos, 4 km al N de Playa Azul, rumbo a la desviación a Lázaro Cárdenas-Tecomán, 18.0196, -102.3414, 50 m, 21 Jun 1998, *P. Tenorio L. 19750* (MEXU). **Oaxaca**: Dto. Yautepec, Toma de Agua, 16.4433, -90.0044, 1036 m, 24 May 2012, *D. López P. 3039* (DAV). **Sinaloa**: Culiacán, 27 Aug–15 Sep 1981, *E. Palmer 1502* (NY).

#### 43 Lycianthes sideroxyloides

Taxon classificationPlantae

(Schltdl.) Bitter, Abh. Naturwiss. Verein Bremen 24 [preprint]: 403. 1919

Fig. 98

Solanum sideroxyloides Schltdl., Linnaea 8: 253. 1833. Type: México. Veracruz: Hacienda de la Laguna, July 1829, *C. J. W. Schiede 135* (lectotype designated here: HAL [acc. # 100610]; isolectotypes: E [E00190770], G [G00379130], GOET [GOET003590], LE [LE00017034], MO [acc. # 2090811], NY [00139028], P [00371472], W [acc. # 1889-292190]).

##### Type.

Based on *Solanum sideroxyloides* Schltdl.

**Figure 98.**
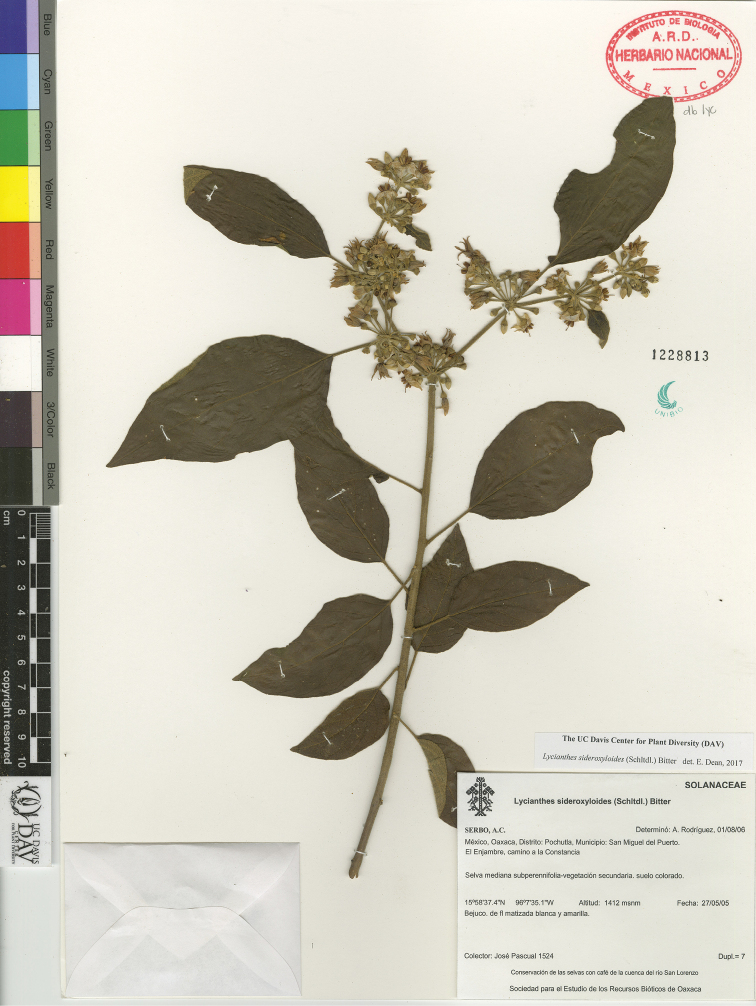
Image of herbarium specimen of *L. sideroxyloides*, *Pascual 1524* (MEXU). Specimen used with permission from the Herbario Nacional de México, Universidad Autónoma de México.

##### Description.

Scandent shrub to vine, 1–10 m tall. Indument of pale yellow to reddish-brown, uniseriate, multicellular, stalked or sessile, multangulate-stellate or geminate-stellate, eglandular, spreading trichomes 0.1–0.5 (0.75) mm long, 0.25–0.75 mm in diameter, the rays 5–8 rays per whorl, straight, not rebranched. Stems pale green (drying tan) when young, sparsely to densely pubescent (the surface often obscured), not compressed when dried in a plant press, becoming brown and woody with age; upper sympodial branching points a mixture of monochasial and dichasial, the branching near the tips of the plant divaricate (diverging at wide angles). Leaves simple, the leaves of the upper sympodia usually unpaired, the blades 2.5–15 × 1.5–8 cm, ovate to elliptic, chartaceous, thick chartaceous, or subcoriaceous, sparsely pubescent adaxially (often shiny, with trichomes just concentrated along the veins), moderately to densely pubescent abaxially with surface sometimes obscured, the base cuneate to rounded, sometimes oblique, the margin entire, usually irregularly undulate, the apex acute to acuminate, rarely obtuse, the petiole 0.5–3 cm long, the larger leaf blades with 4–7 primary veins on each side of the midvein. Flowers usually in groups of 4–30, (the densest groupings spherical in shape), axillary, erect; peduncles absent; pedicels 3–10 mm long and erect in flower, to 15 mm long and erect in fruit, densely pubescent (the surface often obscured); calyx 1–2 mm long, 2.5–3.5 mm in diameter, campanulate, densely pubescent, the margin truncate, with 10 obovate appendages, sometimes just small protuberances, 0.5–1.5 (2) mm long emerging 0.3–0.5 mm below the calyx rim; fruiting calyx enlarged, widely bowl-shaped to rotate, 1.5–2.5 mm long, 4–6 mm in diameter, the appendages not enlarging; corolla 0.5–1.1 cm long, campanulate to reflexed in orientation, stellate in outline, divided 1/2 to 2/3 of the way to the base, with scant interpetalar tissue present connecting the base of the lobes, white (lilac) and glabrous to sparsely pubescent adaxially, pale green to whitish and densely and evenly pubescent on the lobes abaxially; stamens equal, straight, the filaments ca. 0.5 mm long, glabrous, the anthers 2.5–3 mm long, elliptic to lanceolate, free of one another, yellow to purple-yellow, glabrous or with scattered trichomes, poricidal at the tips, the pores ovate, dehiscing distally, not opening into longitudinal slits; pistil with glabrous ovary, the style 5–6 mm long, linear, straight to curved, glabrous, the stigma truncate, decurrent down the sides. Fruit a berry, 2–7 mm long, 2–7 mm in diameter, globose, green to whitish when immature, orange-red when mature, glabrous or with scattered trichomes, lacking sclerotic granules. Seeds 15–40 per fruit, 1.5–2 × 1.5 mm, flattened, thickened on edges, circular, depressed ovate, or reniform in outline, yellow-orange to dark orange, the surface reticulum with minute serpentine pattern and shallow luminae.

##### Chromosome number.

Unknown.

##### Distribution and habitat.

**Figure 99.**
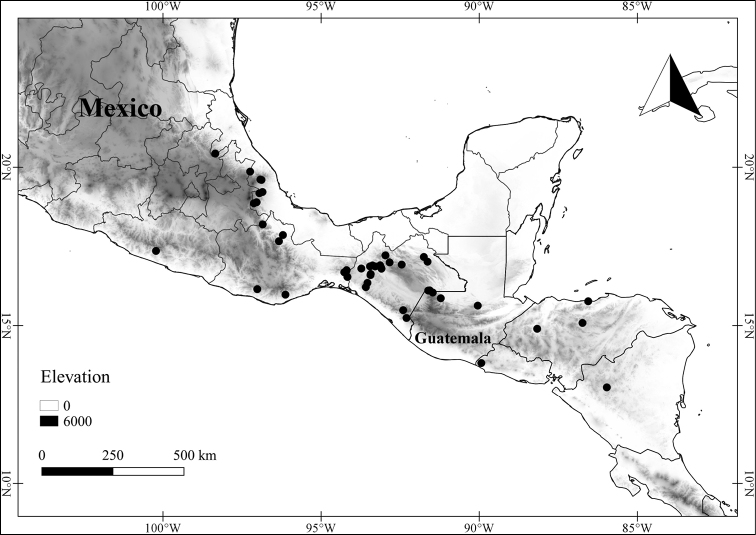
Map of geographic distribution of *L. sideroxyloides* based on herbarium specimen data.

Mexico (Chiapas, Guerrero, Oaxaca, Veracruz), Guatemala (Alta Verapaz, Huehuetenango), El Salvador, Honduras, and Nicaragua, in montane rainforest, tropical moist forest, tropical dry forest, oak forest, and pine-oak forest, sometimes on slopes or in disturbed forest, along roadsides or in coffee plantations, often on limestone, 500–1850 m in elevation (Fig. 99).

##### Common names and uses.

None known.

##### Phenology.

Flowering specimens have been collected from March to November; specimens with mature fruits have been collected May to December. Many specimens have closed flowers, indicating that the flowers are open for a short time during the day, probably during the morning. In the field in Guatemala, in cloud forest on an overcast day, the first author observed that the newest flowers were open midday, while older flowers were already closed.

##### Preliminary conservation status.

*Lycianthes sideroxyloides* is a widespread species ranging from southern Mexico to Nicaragua, represented by 49 collections and occurring in seven protected areas. The EOO is 534,220.001 km^2^. Based on the EOO and the number of locations, and following the IUCN (2019) criteria, the preliminary assessment category is Least Concern (LC).

##### Discussion.

*Lycianthes sideroxyloides* is a wide-ranging species with obovate calyx appendages that are noticeably thickened at the tips. It has yellow to orange, geminate-stellate (multistoried) trichomes with 5–8 rays in a whorl. The obovate calyx appendages are similar to those of the wide-ranging *L. pauciflora* (Vahl) Bitter of Central and South America (Benítez de Rojas and D’Arcy 1997) and its relatives. *Lycianthes sideroxyloides* exhibits variation in flower size and flower number per axil, length of calyx appendages, as well as fruit size. Most populations have very short calyx appendages, however there are populations with appendages as long as 2 mm long. In populations with small flowers, the number of flowers per axil can be as many as 30 with the inflorescence appearing spherical. In populations with few flowers per axil, the flower size is larger. Similarly, in populations with few, large flowers, the fruits are larger. Seed size, however, is consistent throughout the range and is the same in fruits of varying sizes; the number of seeds per fruit is greater in larger fruits, with larger fruits having twice as many seeds as small fruits. *Lycianthes sideroxyloides* is morphologically very similar to *Lycianthes ocellata*, which differs in having glandular appendages that dry black, and to *L. cuchumatanensis*, which differs in having thicker leaves with denser pubescence and larger seeds (2.5–3 mm, according to the protologue).

In the protologue for *Solanum sideroxyloides*, Schlechtendal (1833) cited one collection, *Schiede 135*, but did not cite a specific specimen or herbarium. We are designating the duplicate at HAL [acc. # 100610] as the lectotype, chosen from the many duplicates of *Schiede 135* at multiple institutions listed above.

The status of L. sideroxyloides var. transitoria Bitter (Abh. Naturwiss. Verein Bremen 24 [preprint]: 405. 1919. Type: Guatemala, Depto. Alta Verapaz, Pansamalá, 1200 m, Jun, *H. von Tuerkheim 923* [holotype: B]) is unknown, as the one specimen at B upon which it was based is lost and no photo negative is available at F. In the protologue, Bitter (1919) commented that the pubescence of the underside of the leaf in var. transitoria is between the densely felted pubescence of typical *L. sideroxyloides* in Mexico and the sparsely hairy leaf underside of L. sideroxyloides ssp. ocellata, however, var. transitoria lacks the dark spots at the upper end of the short calyx, which defines L. sideroxyloides ssp. ocellata. Therefore, Bitter’s description of the variety matches a sparsely pubescent *L. sideroxyloides*. Gentry and Standley (1974) synonymized var. transitoria with *L. ocellata*, because they did not recognize that *L. sideroxyloides* occurs in Guatemala.

##### Representative specimens examined.

**Guatemala. Alta Verapaz**: on Cobán Road, between Chiracte and Chapultepec Farm, in clearing betweeen km 284/285, 19 May 1964, *E. Contreras 4726* (MO); **Huehuetenango**: northern region, along road from San Ramón to Barillas, 15.8626, -91.2147, 790 m, 15 Aug 2017, *Dean 9515* (DAV). **Mexico. Chiapas**: Mpio. Tuxtla Gutiérrez, Mirador El Roblar, Parque Nacional Cañón del Sumidero, 16.7972, -93.0897, 940 m, 23 Aug 2007, *J.A. Espinosa J. 273* (MEXU). **Guerrero**: Mpio. Atoyac, cuenca del Río Balsas y Sierra Madre del Sur, a 15 km al NE de El Paraíso, [17.3574, -100.2194], 1100 m, 19 Aug 1985, *J.C. Soto N. 10103* (MEXU). **Oaxaca**: Mpio. San Miguel del Puerto, El Enjambre, camino a la Constancia, 15.9771, -96.1264, 1412 m, 27 May 2005, *J. Pascual 1524* (IEB, MEXU). **Veracruz**: Cerro Buenavista, 18.8944, -97.0375, 1255 m, 31 Aug 1995, *B. Juárez L. 719* (MEXU, XAL).

#### 44 Lycianthes starbuckii

Taxon classificationPlantae

E.Dean, Novon 4: 324, 1994

Fig. 100

##### Type.

México. México: Sierra de Nanchititla, oak forest across the reservoir from the town of Nanchititla, 1945 m, 8 September 1991, *E. Dean 315* (holotype: UC [UC1862224]; isotypes: BM [001000924], DAV [DAV158254, DAV158084, DAV158083, DAV158082], ENCB, MEXU [MEXU01195795], MO [2246353], NY [00687930], XAL [XAL0106673, XAL0106672].

**Figure 100.**
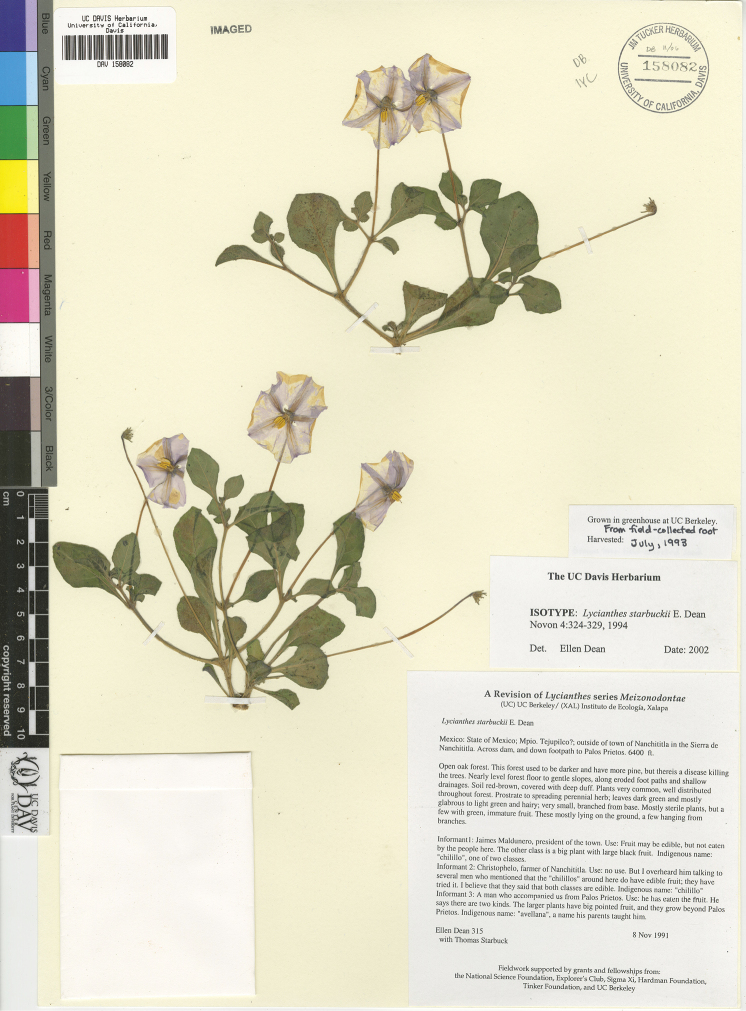
Image of isotype of *L. starbuckii*, *Dean 315* (DAV). Image used with permission of the UC Davis Center for Plant Diversity.

##### Description.

Perennial herb, from fusiform storage roots, usually prostrate to ascending, to 0.15 m tall, dying back each season. Indument of white, uniseriate, multicellular, simple, eglandular, spreading to appressed trichomes, 0.1–0.5 mm long. Stems greenish-purple, moderately to densely pubescent, usually compressed when dried in a plant press, with very little woody tissue; first stem 0.5–3 cm long to the first inflorescence, the internodes 2–5; first sympodial branching point usually dichasial, followed by a mixture of monochasial and dichasial branching, this branching extensive, usually resting on the soil surface. Leaves simple, those of the upper sympodia usually paired and unequal in size, the larger ones with blades 3–6 × 1.25–3 cm, the smaller ones with blades 1/4 to 1/2 the size of the larger, the leaf pairs similar in shape, the blades obovate, oblanceolate, ovate, broadly elliptic, or rhombic, thick chartaceous, sparsely to moderately pubescent, the primary veins 3–5 on either side of the midvein, the base cuneate, sometimes attenuate onto the petiole, sometimes oblique, the margin entire, usually irregularly undulate, the apex broadly acute to rounded, the petioles winged and poorly defined, to 1.8 cm long, sometimes absent. Flowers solitary, axillary, oriented horizontally; peduncles absent; pedicels 42–86 mm and erect in flower, 52–122 mm long, deflexed, and undulate in fruit, moderately pubescent with spreading trichomes of two distinct lengths, the shorter to 0.25 mm long and the longer to 0.5 mm long; calyx 3–4 mm long, 4–5.5 mm in diameter, narrowly to broadly conic, moderately pubescent, the margin truncate, with 10 linear appendages lying laxly near the corolla surface 2–6.5 mm long emerging ca. 0.5 mm below the calyx rim; fruiting calyx enlarged, 2–5.5 mm long, 6–12.5 mm in diameter, the appendages spreading to reflexed, often broken, 2–6.5 mm long; corolla 1–2 cm long (2.1–3.8 cm in diameter), rotate in orientation, mostly entire in outline (with shallow notches), with abundant interpetalar tissue, lilac, with violet stripes near the major veins adaxially, green and moderately pubescent near the major veins abaxially; stamens unequal, the filaments of three lengths, the two shortest filaments 1.25–3.25 mm long, the two medium filaments 1.5–3.75 mm long, the one long filament 2–5.25 mm long, the length of the long filament usually less than 2 times that of the medium filaments, glabrous, the anthers 3.25–5.25 mm, lanceolate to elliptic, free of one another, yellow, glabrous, poricidal at the tips, the pores round, dehiscing distally, not opening into longitudinal slits; pollen grains irregular in shape and number of pores; pistil with glabrous ovary, the style 7–10 mm, linear, straight to slightly curved, glabrous, the stigma round, shallowly lobed. Fruit a berry, separating from calyx at maturity and matures lying on the ground, 17–22 mm long, 7–17 mm in diameter, ovoid, the exocarp dull dark purple at maturity, glabrous, the mesocarp dark purple, soft and juicy, lacking sclerotic granules, the placental area light purple, powdery in texture. Seeds 2–29 per fruit, 3.5–4.5 × 3–4.3 mm, not compressed, depressed obovate, ridged and blistered along one side, black, the surface reticulum rough in texture with loose serpentine pattern and deep luminae.

##### Chromosome number.

2n = 24, *Dean 315* (Dean 2004)

##### Distribution and habitat.

**Figure 101.**
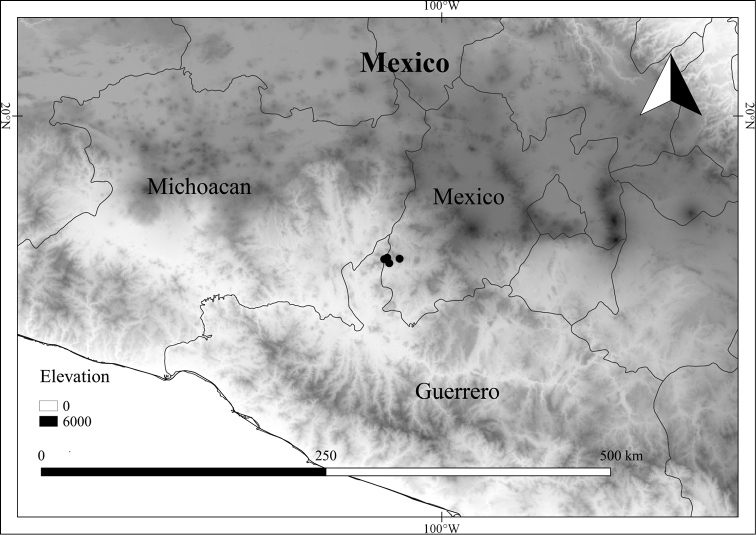
Map of geographic distribution of *L. starbuckii* based on herbarium specimen data.

Mexico, endemic to southwestern state of México, Sierra de Nanchititla, on level oak forest ﬂoor, 1945 m in elevation (Fig. 101).

##### Common names and uses.

Mexico. México: chilillo (Dean 2004).

##### Phenology.

Flowering specimens have been collected July and August; specimens with mature fruits have been collected in October and November. The first author has observed in the field that the corollas open in the very early morning and close by late morning. The pollen of this species has a sweet fragrance.

##### Preliminary conservation status.

*Lycianthes starbuckii* is a rare species of central Mexico, represented by only four collections from the type location, which is not a protected area. The conservation status of *L. starbuckii* was investigated by Anguiano-Constante et al. (2018), and their preliminary conservation assessment for this species was Endangered.

##### Discussion.

*Lycianthes starbuckii* can be distinguished from other species of series *Meizonodontae* by its combination of prostrate to ascending habit, densely pubescent stems, thick-chartaceous leaves, lax calyx teeth at anthesis, moderately pubescent corolla lobes, and dark purple fruits with large brown to black seeds. *Lycianthes starbuckii* is unusual in the *L. ciliolata* complex in its habit and pubescent corolla lobes (Dean 2004). In these characteristics, it is much closer to such species as *L. moziniana* and *L. peduncularis*. *Lycianthes starbuckii* may hybridize with *L. rzedowskii*, which grows in drainage areas near Nanchititla (Dean 2004). There are specimens collected in southern Guerrero that strongly resemble *L. starbuckii*, but they have a more erect habit, rather than prostrate; these collections are discussed below under Difficult to Place Specimens. Field work is needed to investigate these populations.

##### Representative specimen examined.

**Mexico. México**: km 15 carretera El Estado-cañadas de Nanchititla, camino Torrecillas, 18.874, -100.3326, 1934 m, 27 Jul 2010, *A. Rodríguez 6083* (IBUG, IEB, MEXU).

#### 45 Lycianthes stephanocalyx

Taxon classificationPlantae

(Brandegee) Bitter, Repert. Spec. Nov. Regni Veg. 18: 315. 1922

Fig. 102

Solanum stephanocalyx Brandegee, Univ. Calif. Publ. Bot. 6: 374. 1917. Type: Mexico. Veracruz: Zacuapan, Jul 1915, *C. Purpus 7519* (holotype: UC [UC178649]; isotypes: GH [00077535], NY [00139030]).
Lycianthes symphyandra Bitter, Abh. Naturwiss. Verein Bremen 24 [preprint]: 430. 1919. Type: Mexico. Veracruz: Mirador, 1842, *F. Liebmann 1456* (lectotype designated by Dean and Reyes 2018a, pg. 44: C [C10022133]).
Solanum solitarium S.F.Blake, Contr. U.S. Natl. Herb. 24: 21. 1922. Type: Guatemala. Izabal: Río Mojanales, 17 May 1919: *S. F. Blake 7489* (holotype: US [00027803]).
Lycianthes solitaria (S.F.Blake) Standl., J. Wash. Acad. Sci. 17: 15. 1927. Type: Based on Solanum solitarium S.F.Blake.
Lycianthes luisana Standl., Publ. Field Mus. Nat. Hist., Bot. Ser. 22: 101. 1940. Type: Mexico. San Luis Potosí: Tamazunchale, Jul 1937, *M. Edwards 913* (holotype: F [0072917F, acc. # 918327]; isotypes: ARIZ [ARIZ-BOT-0005035], CAS [0003290], MO [503464]).
Solanum symphyandrum (Bitter) C.V.Morton, Contr. Univ. Michigan Herb. 4: 27. 1940. Type: Based on Lycianthes symphyandra Bitter.
Solanum hondurense C.V.Morton, Contr. Univ. Michigan Herb. 4: 26. 1940. Type: Belize. El Cayo: Chalillo Crossing, 15 Jul 1936, *C. Lundell 6512* (holotype: US [00027603]; isotypes: LL [00372878], MICH [1109936]).
Lycianthes hondurensis (C.V.Morton) Standl. & Steyerm., Publ. Field Mus. Nat. Hist., Bot. Ser. 23: 18. 1943. Type: Based on Solanum hondurense C.V.Morton.

##### Type.

Based on *Solanum stephanocalyx* Brandegee.

**Figure 102.**
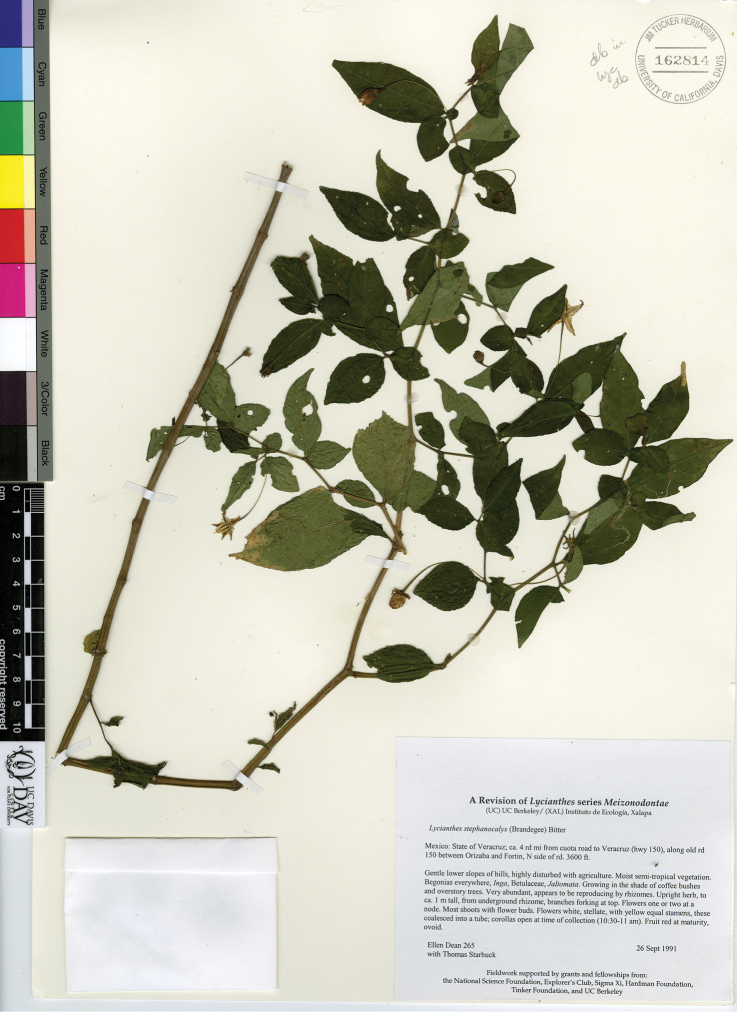
Image of herbarium specimen of *L. stephanocalyx*, *Dean 265* (DAV). Image used with permission of the UC Davis Center for Plant Diversity.

##### Description.

Perennial herb to climbing shrub, erect, often recumbent with age, to 2 (3) m tall, dying back to rhizomes. Indument of small, white, uniseriate, multicellular, simple, curved, eglandular, appressed-ascending trichomes 0.1–0.6 mm long. Stems green when young, glabrous to sparsely pubescent, compressed and ribbed upon drying in a plant press, brown and woody with age; upper sympodial branching points monochasial or dichasial. Leaves simple, the leaves of the upper sympodia usually paired and unequal in size, the larger ones with blades 3.5–15 × 1.5–6.2 cm, the smaller ones with blades 0.7–6.5 (10.5) × 0.5–3.1 (5) cm, the leaf pairs similar in shape, the blades ovate (sometimes narrowly), elliptic, or obovate, the blades of both the large and small leaves chartaceous to thick chartaceous, glabrous to sparsely pubescent, the base truncate, cuneate, or attenuate, sometimes oblique, the margin entire, the apex acute to acuminate, the petiole 0.1–0.9 (2) cm long, sometimes absent, the large leaf blades with 3–6 primary veins on each side of the midvein. Flowers solitary, axillary, pendent; peduncles absent; pedicels 12–45 mm and arching to deflexed in flower, to 53 mm long and deflexed in fruit, glabrous to sparsely pubescent; calyx 1.5–3 (4) mm long, 3–4 mm in diameter, obconic to campanulate, glabrous to sparsely pubescent, the margin truncate, with 10 linear, spreading to reflexed appendages 1.5–5 mm long emerging 0.5 mm below the calyx rim; fruiting calyx usually enlarged, widely campanulate to bowl-shaped, 1.5–4 mm long, 3–8 mm in diameter, the appendages 2–8 mm long, spreading; corolla 0.5–1.4 cm long, campanulate to reflexed in orientation, stellate in outline, divided 1/3–2/3 of the way to the base, (lobes shallow on first day that the flower opens, becoming deeper each subsequent day that the flower opens), with interpetalar tissue, adaxially and abaxially white to light purple, glabrous; stamens equal, straight, the filaments 1–1.5 mm long, glabrous, the anthers 4.5–7 mm long, lanceolate, connivent to connate at edges to adjacent anther, yellow, glabrous, poricidal at the tip, the pores round, dehiscing distally, not opening into longitudinal slits; pistil with glabrous ovary, the style 6–9 mm long, linear, straight, glabrous, the stigma truncate. Fruit a berry, 3–10 (17) mm long, 3–9 (12) mm in diameter, globose to ovoid, orange to red at maturity, glabrous, lacking sclerotic granules. Seeds 7–60 per fruit, 1.5–3 × 1.5–2 mm, flattened, depressed ovate in outline, tan to orange, the surface reticulum with a tight serpentine pattern with shallow luminae.

##### Chromosome number.

Unknown.

##### Distribution and habitat.

**Figure 103.**
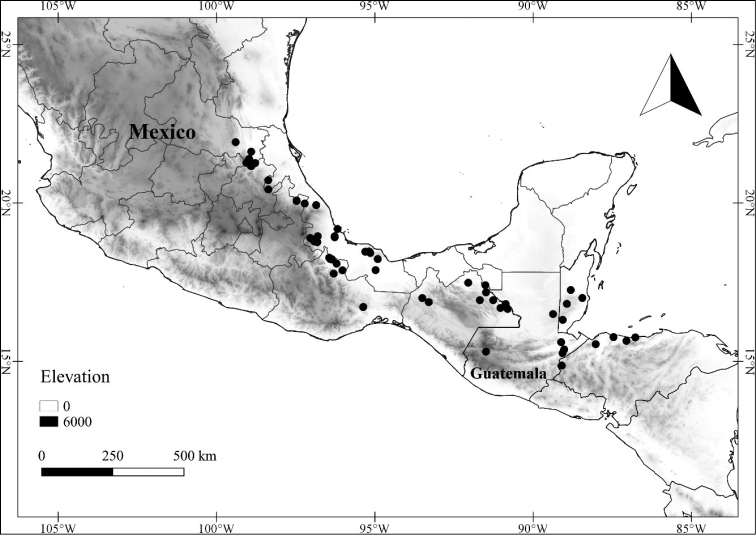
Map of geographic distribution of *L. stephanocalyx* based on herbarium specimen data.

Mexico (Chiapas, Hidalgo, Oaxaca, Puebla, Querétaro, San Luis Potosí, Tabasco, Veracruz), Guatemala (Huehuetenango, Izabal, Petén), Belize, and Honduras (and possibly further south in Central America), in tropical rainforest, tropical dry forest, tropical moist forest, and cloud forest, sometimes in coffee plantations or disturbed forest, near rivers or waterfalls, in gorges, or on the sides of canyons, 30–1050 m in elevation (Fig. 103).

##### Common names and uses.

Mexico. San Luis Potosi: tomatillo, arrete de la virgin, flor de mariposa (Standley 1940); Veracruz: masan ay (from herbarium specimen *M. Leonti 71*).

##### Phenology.

Flowering specimens have been collected May to September. Fruiting specimens have been collected September to December. It is possible this species flowers and fruits throughout the year in some locations. The first author observed in the field in Mexico that the corollas are open in the very early morning and closed by late morning.

##### Preliminary conservation status.

*Lycianthes stephanocalyx* is a widespread species ranging from western Mexico to Honduras, represented by 68 collections and occurring in six protected areas. The EOO is 361,720.394 km^2^, and the AOO is 260 km^2^. Based on the IUCN (2019) criteria, the preliminary assessment category is Least Concern (LC).

##### Discussion.

*Lycianthes stephanocalyx* is a rhizomatous herb (that can sometimes produce above-ground woody growth) with white stellate flowers and equal, connate anthers. Its closest relatives are not yet fully known, but they are probably other species with equal stamens and stellate corollas such as *L. heteroclita* and *L. geminiflora*. It was placed by Georg Bitter in his series *Pilifera* (Bitter, 1919), but it is probably not closely related to the other species he placed in the series, such as *L. pilifera* and *L. quichensis* , both of which are shrubs occurring at relatively high elevations (Dean et al. 2019b). *Lycianthes stephanocalyx* is sometimes confused with *L. pilifera* in herbaria, because both species have flowers with equal stamens, and *L. pilifera* sometimes has one-flowered inflorescences. *Lycianthes stephanocalyx* does overlap in distribution with *L. pilifera* and differs in having red fruit (rather than dark purple), connivent yellow anthers (rather than free purplish anthers), and small whitish curved trichomes (rather than straight brown pointed trichomes) (Dean et al. 2019b).

##### Representative specimens examined.

**Guatemala. Huehuetenango**: Sierra de los Cuchumatanes, between Xoxlac and Nucapuxlac, [15.3094, -91.4894], 1650–2500 m, 17 Jul 1942, *J.A. Steyermark 48960* (NY). **Izabal**: Chickasaw Farm of the United Fruit Company, about 15 km north of Quirigua, 70 m, 28 May 1922, *P.C. Standley 24628* (GH). **Petén**: Dolores, bordering Río Mopan, in clearing 6 km SE, [16.5089, -89.4065], 29 Jun 1961, *E. Contreras 2566* (CAS, MO). **Mexico. Chiapas**: Mpio Ocosingo, 5 km al S de Campamento COFOLASA, el cual está a 24 km al SE de Crucero Corozal, camino Palenque-Boca Lacantum, [16.6556, -90.8063], 220 m, 24 Sep 1984, *E. Martínez S. 7847* (NY). **Hidalgo**: 53 km al noreste de Zimapan, [21.1662, -98.9166], 1000 m, 7 Nov 1979, *R. Hernández Magaña 3898* (MEXU). **Oaxaca**: Dto. Tehuantepec, 3 km al norte de Santa María Guienagati, carretera a Guevea de H, 16.7167, -95.3667, 460 m, 27 Aug 1991, *A.D. Campos-Villanueva 3849* (MEXU). **Puebla**: road (575) Cuetzalan to San Antonio Rayón [Santiago Yancuictalpan], 20.0617, -97.4706, 592 m, 10 Nov 2014, *P. Acevedo-Rodríguez 16044* (DAV). **Querétaro**: 2 km al sureste de Neblinas, Río Tancuilín, 21.2662, -99.0537, 610 m, 12 Sep 1990, *H. Rubio 1954* (DAV, IEB). **San Luis Potosí**: San Antonio, [21.6180, -98.9039], 7 Sep 1978, *J. Alcorn 1649* (TEX). **Tabasco**: a orillas del Chinilkija en el ejido Linda Vista, [17.4058, -91.5075], 2 Aug 1990, *M.A. Magaña 2299* (MEXU). **Veracruz**: 6 km en línea recta al sureste de Zontecomatlán, ejido Cabellete, 20.7172, -98.3667, 800–1100 m, 8 Sep 2000, *A. Rincón G. 1869* (IEB, MEXU).

#### 46 Lycianthes surotatensis

Taxon classificationPlantae

Gentry, Brittonia 6 (3): 323. 1948

Fig. 104

##### Type.

Mexico. Sinaloa: Sierra Surotato, Las Mesas, 15 Sep 1941, *H. S. Gentry 6620* (holotype: MICH [1109850]; isotypes: ARIZ [ARIZ-BOT-0005037], DES [DES00041687], MEXU, MO [acc. # 1217396], GH [00934884], NY [00138708]).

**Figure 104.**
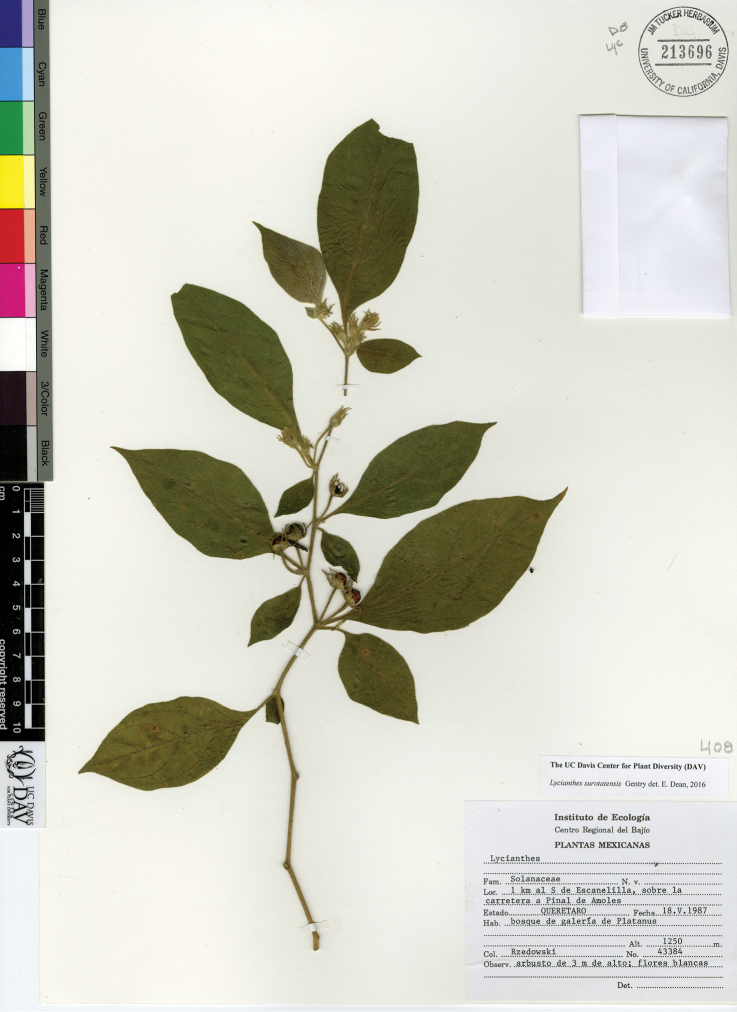
Image of herbarium specimen of *L. surotatensis*, *Rzedowski 43384* (DAV). Image used with permission of the UC Davis Center for Plant Diversity.

##### Description.

Shrub, (0.6) 1–3 m tall. Indument of off-white to tan (purple), uniseriate, multicellular, simple, glandular and eglandular, spreading to appressed trichomes 0.2–2.1 mm long. Stems pale green (drying tan) with dark lenticular vertical striations when young, moderately pubescent, not much compressed when dried in a plant press, becoming brown and woody with age; upper sympodial branching points dichasial and monochasial. Leaves simple, the leaves of the upper sympodia usually paired and unequal in size, the larger ones with blades 8.1–16.5 × 3.4–9.5 cm, the smaller ones with blades 1.6–7.8 × 0.6–4.2 cm, the leaf pairs similar in shape, the blades ovate to elliptic, thin-chartaceous, moderately pubescent, the base cuneate to attenuate, sometimes oblique, the margin entire, usually irregularly undulate (rarely remotely coarsely dentate), the apex acuminate, the petiole 0.7–2.2 cm long, sometimes absent, the larger leaf blades with 5–6 primary veins on each side of the midvein. Flowers solitary or in groups of 2–3 (5), axillary, oriented horizontally; peduncles absent; pedicels 9–33 mm long and erect in flower, 11–29 mm long and erect in fruit, moderately pubescent; calyx 2–4 mm long, 2.5–6 mm in diameter, widely obconic to widely campanulate, densely puberulent, the margin truncate, with 10 spreading linear appendages 2–10 mm long, emerging ca. 0.5 mm below the calyx rim; fruiting calyx enlarged, widely bowl-shaped to rotate, 1–2 (4) mm long, (3) 5–9 mm in diameter, the appendages 6–11 mm long (probably longer), 0.5–1 mm wide at the base, spreading; corolla 0.9–1.8 cm long, rotate in orientation, mostly entire in outline (with shallow notches), with abundant interpetalar tissue, adaxially white, sometimes with three green spots near the insertion of the stamen filaments, abaxially green and puberulent with purple trichomes near the major veins; stamens unequal, straight, the four short filaments 1–2 mm long, the one long filament 3.5–4.5 mm long, glabrous, the anthers 3.5–5 mm long, elliptic, lanceolate, or oblong, free of one another, yellow, glabrous, poricidal at the tips, the pores ovate, dehiscing distally, not opening into longitudinal slits; pistil with glabrous ovary, the style 7–10 mm long, linear, slightly curved, glabrous, the stigma capitate, decurrent down two sides, sometimes slightly bilobed. Fruit a berry, 4–11 mm long, 4–12 mm in diameter, globose to depressed globose, red at maturity, glabrous, lacking sclerotic granules. Seeds 40–137 per fruit, 1.75–2.25 × 1.5 mm, flattened, reniform to triangular in outline, tan, the surface reticulum with minute serpentine pattern and shallow luminae.

##### Chromosome number.

Unknown.

##### Distribution and habitat.

**Figure 105.**
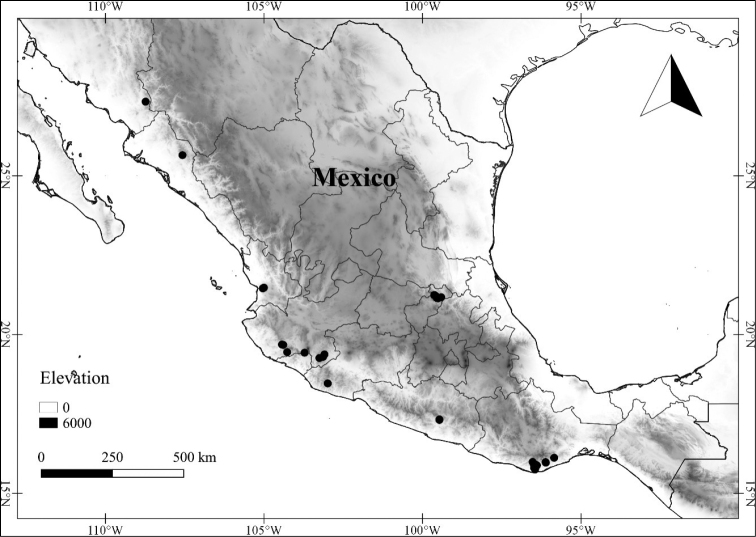
Map of geographic distribution of *L. surotatensis* based on herbarium specimen data.

Mexico (Colima, Guerrero, Jalisco, Michoacán, Nayarit, Oaxaca, Quéretaro, Sinaloa, and Sonora) in broadleaved forest, oak forest, pine-oak forest, tropical dry forest, and riparian forest (including *Platanus* gallery forest), 670–2200 m in elevation (Fig. 105).

##### Common names and uses.

None known.

##### Phenology.

Flowering specimens have been collected from March to December; specimens with mature fruits have been collected February to December. The first author observed in the field that the corollas are open in the morning and closed by afternoon.

##### Preliminary conservation status.

*Lycianthes surotatensis* is a locally common species with a disjunct distribution, ranging from central and western Mexico to southern Mexico, represented by 38 collections and occurring in two protected areas. The EOO is 497,854.619 km^2^, and the AOO is 132 km^2^. Based on the IUCN (2019) criteria, the preliminary assessment category is Least Concern (LC).

##### Discussion.

*Lycianthes surotatensis* is very similar to *L. tricolor* in its pedicel length and corolla size. It differs from that species in having unnotched seeds and glandular pubescence at least on the pedicels or calyx. In addition, the calyx length and calyx appendage length are usually longer in *L. surotatensis*. In the protologue for *L. surotatensis*, Gentry (1948) did not note the presence of glandular trichomes on the type specimen and concentrated instead on the dentate margins that are present on some of the leaves of the type specimen. However, the trichomes on the leaves, pedicels, and calyx of the type specimen are clearly glandular, and the character of the dentate leaf margins is not consistently present within the species and sometimes can occur in other species of series *Tricolores* (Dean et al. 2017a). The disjunct distribution of this species, with an isolated group of populations in Querétaro, needs investigation.

##### Representative specimens examined.

**Mexico. Colima**: southwestern foothills of the Nevado de Colima, 1–1.5 miles above (S of) Hacienda San Antonio, [19.4285, -103.7194], 1200–1250 m, 11 Aug 1957, *R. McVaugh 16084* (MEXU). **Guerrero**: Agua de Obispo, 17.3139, -99.4667, 1050 m, 17 Oct 1963, *Kruse 1032* (IEB, MEXU, MO). **Jalisco**: Sierra del Halo, cañada La Jabalina, 12 km en línea recta al este de Pihuamo, 3.5 km al oeste de Alotitlan, 1750 m, 23 Feb 2012, *A. Castro-Castro 2665* (XAL). **Michoacán**: Mpio. Coalcomán, approx. 4 km (en línea recta) al sur de Puerto La Bufa, 18.4622, -102.9881, 1750 m, 25 May 2008, *Y. Ramírez-Amézcua 1320* (DAV). **Nayarit**: Mpio. Tepic, 1 km al SW de El Cuarenteño, camino a El Cora, o 4 km al N del entronque del camino El Cora-Palapitas, [21.4544, -105.0357], 820 m, 16 May 1994, *G. Flores-Franco 3457* (MEXU). **Oaxaca**: Cerro Zapote, a 88 km en LR (325 N) de Santa María Zapotitlán, 16.12025, -95.8475, 1600 m, 15 Mar 2006, *K. Velasco-Gutíerrez 1248* (MEXU). **Querétaro**: Mpio. Pinal de Amoles, La Cuesta, 3 km al S de Escanelilla, [21.1411, -99.5089] 1100 m, 14 Dec 1994, *R. Fernández N. 2109* (MEXU). **Sinaloa**: Las Mesas, Sierra Surotato, [25.6496, -107.5702], 15 Sep 1941, *H.S. Gentry 6620* (ARIZ, DES, MICH, MO, NY). **Sonora**: Río Mayo region, Arroya Tepopa, about 35 km NE of Alamos, “main fork”of canyon above confluence with “waterfall fork,” above old ranch site, 27.3333, -108.7333, 1100 m, 16 Mar 1993, *M. Fishbein 1040* (NY).

#### 47 Lycianthes textitlaniana

Taxon classificationPlantae

E.Dean, Phytologia (Dec 18, 2017) 99: 242. 2017

Fig. 106

##### Type.

Mexico: State of Oaxaca, Dto. Sola de Vega, Mpio. Santiago Textitlán, Colonia Nueva, 18 Aug 2006, *Alma Zarate Marcos AZM-274* (holotype: MEXU [acc. # 1229513]; isotype: SERBO [acc. # 115451].

**Figure 106.**
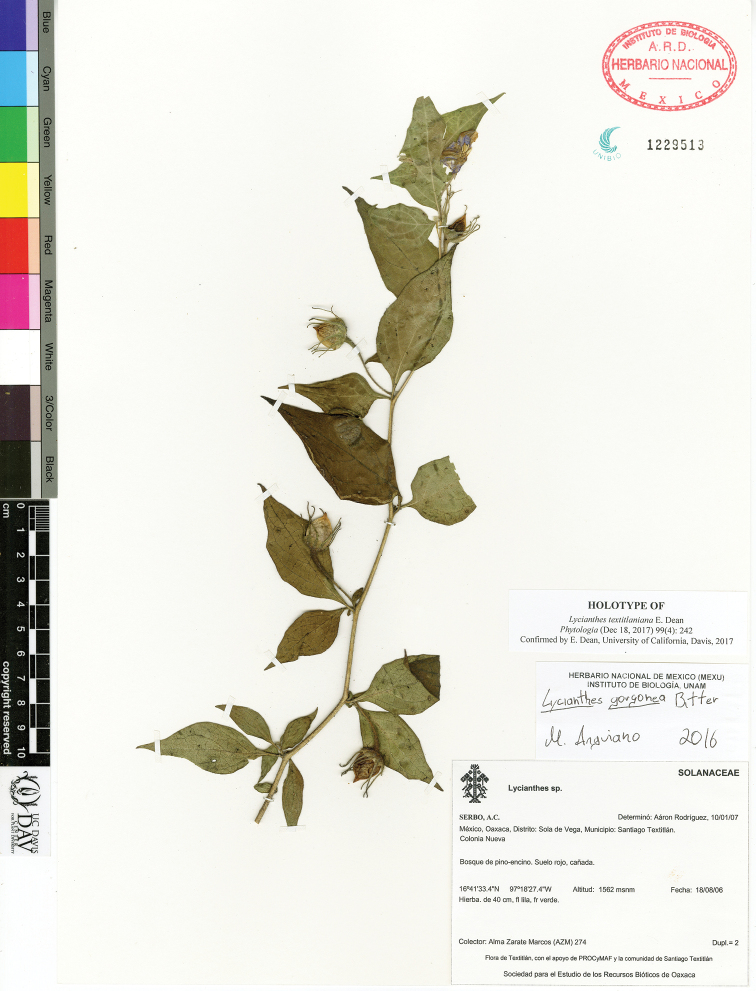
Image of holotype of *L. textitlaniana*, *Zarate 274* (MEXU). Specimen used with permission from the Herbario Nacional de México, Universidad Autónoma de México.

##### Description.

Small shrub, ca. 0.5 m tall. Indument of clear to white, uniseriate, multicellular, simple, crisped, glandular (glandular tip golden-yellow to grey), spreading trichomes 0.25–1 (1.5) mm long. Stems pale green (drying tan) when young, moderately to densely pubescent, not much compressed when dried in a plant press, becoming brown and woody with age; upper sympodial branching points dichasial and monochasial. Leaves simple, the leaves of the upper sympodia usually paired and unequal in size, the larger ones with blades 3–7 × 1.5–3 cm, the smaller ones with blades 1.5–5 × 0.5–2 cm, the leaf pairs similar in shape, the blades ovate, to elliptic, chartaceous, moderately to densely pubescent, the base truncate, cuneate, or attenuate, sometimes oblique, the margin entire, usually irregularly undulate, the apex acuminate, the petiole 0.1–1 cm long, sometimes absent, the larger leaf blades with 4–5 primary veins on each side of the midvein. Flowers solitary, axillary, oriented horizontally; peduncles absent; pedicels ca. 20–25 mm long and erect in flower, 20–30 mm long and erect to arching in fruit, moderately to densely pubescent; calyx 3–3.5 mm long, 3.5–4 mm in diameter, campanulate, moderately to densely puberulent, the margin truncate, with 10 spreading linear, basally flattened appendages 7–10 mm long emerging ca. 0.5 mm below the calyx rim; fruiting calyx enlarged, widely bowl-shaped, 4–6 mm long, 9–12 mm in diameter, appendages to 15 mm long; corolla 1.2–1.5 cm long, rotate in orientation, mostly entire in outline (with shallow notches), with abundant interpetalar tissue, purple and glabrous adaxially, color of abaxial side unknown, glabrous; stamens unequal, straight, the four short filaments 1–2 mm long, the one long filament 2–3 mm long, glabrous, the anthers 3.5–4 mm long, elliptic to lanceolate, free of one another, yellow, glabrous, poricidal at the tips, the pores ovate, dehiscing distally, not opening into longitudinal slits; pistil with glabrous ovary, the style ca. 7 mm long, linear, glabrous, the stigma capitate, decurrent down two sides. Fruit a dry berry lacking juicy mesocarp, 13–15 mm long, 9–12 mm in diameter, turbinate, the tip apiculate, pale greenish orange when mature, glabrous, lacking sclerotic granules. Seeds 50–80 per fruit, 1.75–2 × 1.25–1.5 mm, somewhat compressed, round-edged, reniform in outline, tan-orange, surface pitted.

##### Chromosome number.

Unknown.

##### Distribution and habitat.

**Figure 107.**
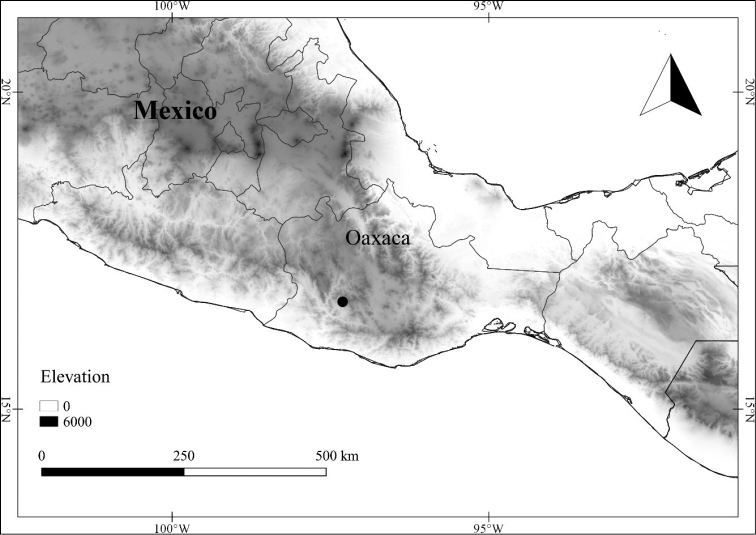
Map of geographic distribution of *L. textitlaniana* based on herbarium specimen data.

Mexico (Oaxaca), in pine-oak forest, ca. 1500 m in elevation (Fig. 107).

##### Common names and uses.

None known.

##### Phenology.

A flowering and fruiting specimen has been collected in July. The timing of the diurnal corolla movements is unknown; however, the corollas are open on the two specimens of the type collection, indicating that the flowers are open for part of the day.

##### Preliminary conservation status.

*Lycianthes textitlaniana* is a rare plant of Oaxaca, Mexico, represented by only one collection, which is not from a protected area and was burned in recent fires (Dean et al. 2017b). The EOO is only 4 km^2^. Based on the very small EOO, and following the IUCN (2019) criteria, the preliminary assessment category is Critically Endangered (CR).

##### Discussion.

*Lycianthes textitlaniana* is unlike any other *Lycianthes* species in its combination of glandular pubescence, relatively long calyx appendages, and dry turbinate berry with round-edged seeds with pitted surface.

##### Representative specimens examined.

This species is only known from the one type specimen cited above.

#### 48 Lycianthes tricolor

Taxon classificationPlantae

(Dunal) Bitter, Abh. Naturwiss. Verein Bremen 24 [preprint]: 385. 1919

Fig. 108

Solanum tricolor Dunal, Encycl. [J. Lamarck & al.] Suppl. 3: 756. 1814 [1813] (official date of publication of volume 3 is 1813, but pages 369–780 were published in 1814 [Stafleu and Cowan 1979]). Type: Painting made during the Royal Expedition to New Spain (1787–1803) under the direction of Martin de Sessé y Lacasta (lectotype designated by Dean et al. 2017a, pg. 205: Hunt Institute for Botanical Documentation HI Art acc. # 6331.04610).
Solanum monodynamum Visiani, Sem. Hort. Patav. (1841): 3. 1841. Type: cultivated plant in the botanical garden at Padua (Padova), Italy, seed source from Mexico (neotype designated by Dean et al. 2017a, pg. 205: La Mexique, Jardin de Padoue, 2 Oct 1844, *collector unknown s.n.* (G-DC [G00145628]).
Solanum pilosissimum M.Martens & Galeotti, Bull. Acad. Roy. Sci. Bruxelles 12(1): 139. 1845. Type: Mexico, Oaxaca, Yavezia, 7000 ft, Nov–Apr, 1840, *H. Galeotti 1228* (lectotype designated by Dean et al. 2017a pg. 205: BR [00000552342]; isolectotypes: BR [00000552309], G [G00359732], P [P00368470], W [acc. # 3080]).
Solanum quadriflorum M.Martens & Galeotti, Bull. Acad. Roy. Sci. Bruxelles 12(1): 139. 1845. Type: Mexico, Oaxaca, Cordillera, 7000 ft, Nov–Apr 1840, *H. Galeotti 1231* (lectotype designated by Dean et al. 2017a pg. 206: BR [00000552205]; isolectotypes: BR [00000552238], P [P00369232, two sheets]).
Solanum nyctaginoides Dunal, Prodr. [A. P. de Candolle]13(1): 172. 1852. Type: Hort. Mexican (also on another duplicate Jardín Bot. D. Mexico), 12/7 (12 Jul) 1827, *J. Berlandier 553* (lectotype designated by Dean et al. 2017a, pg. 205: G-DC [G00145660]; isolectotypes: G [G00359731, G00301639], P [P00385102, P00385101], W [acc. # 0003084].
Lycianthes pilosissima (M.Martens & Galeotti) Bitter, Abh. Naturwiss. Verein Bremen 24 [preprint]: 378. 1919. Type: Based on Solanum pilosissimum M.Martens & Galeotti.
Lycianthes tricolor (Dunal) Bitter var. flavidipila Bitter, Abh. Naturwiss. Verein Bremen 24 [preprint]: 387. 1919. Type: Guatemala, Vulcán de Agua, 2,500 m, 31 May 1882, *F. C. Lehmann 1486* (lectotype designated by Dean et al. 2017a, pg. 205: G [G00414712, 2 sheets]; isolectotypes: US [00027898], K [K000585741], B [cited by Bitter (1919), not found, likely destroyed]).
Lycianthes tricolor (Dunal) Bitter var. primoaurata Bitter, Abh. Naturwiss. Verein Bremen 24 [preprint]: 387. 1919. Type: Guatemala, Volcán de Fuego, ridges above calderas, 8300 ft, Sep 1873, *O. Salvin s.n.* (holotype: W [acc. # 1886–8331]).
Lycianthes tricolor (Dunal) Bitter var. hirsutior Bitter, Abh. Naturwiss. Verein Bremen 24 [preprint]: 388. 1919. Type: Mexico, Castresana [locality likely Cuatresana near San Pedro Nolasco, Oaxaca],1842, *F. Liebmann 1438* (lectotype designated by Dean et al. 2017a, pg. 205: C [10021746]; isolectotypes: C [10021745], US [00027899]).
Lycianthes arrazolensis (J.M.Coult. & Donn.Sm.) Bitter var. patentipila Bitter, Abh. Naturwiss. Verein Bremen 24 [preprint]: 391. 1919. Type: Mexico. Oaxaca, 1750 m, *Conzatti & González 1071* (holotype: GH [00936247]).

##### Type.

Based on *Solanum tricolor* Dunal.

**Figure 108.**
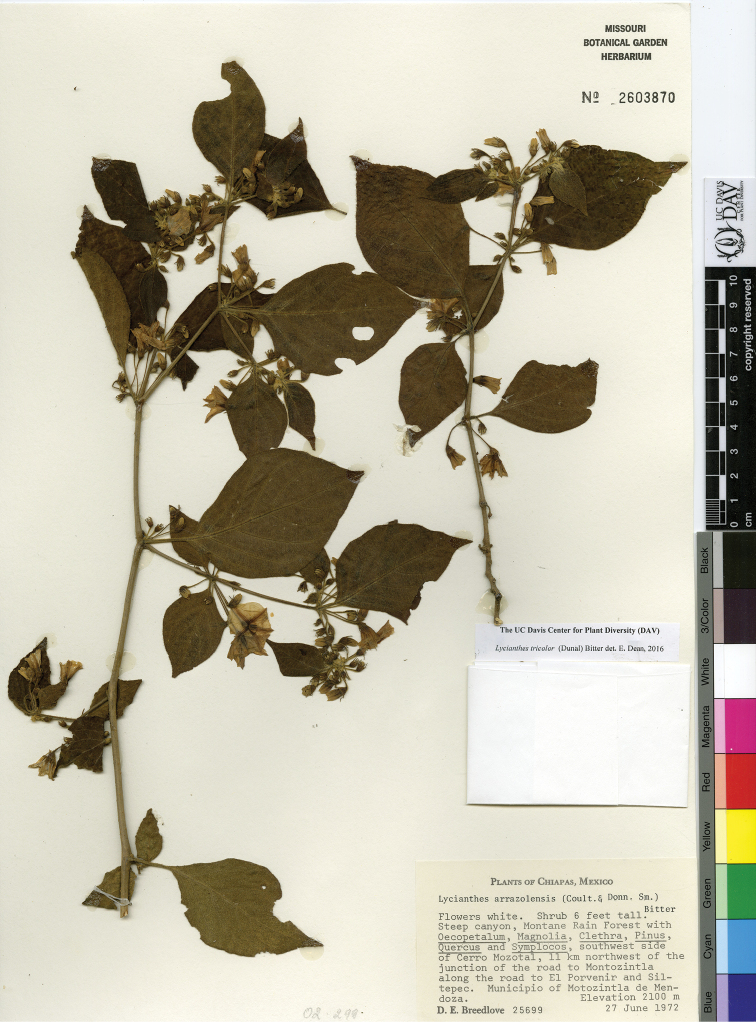
Image of herbarium specimen of *L. tricolor*, *Breedlove 25699* (MO). Specimen used with permission from the Missouri Botanical Garden (http://www.tropicos.org).

##### Description.

Shrub, 1.2–4.5 m tall, sometimes vining or arching through neighboring vegetation. Indument of light yellow, uniseriate, multicellular, simple, often curly, eglandular, spreading to appressed trichomes 0.2–1.75 mm long, sometimes with very small glistening bumps between longer trichomes. Stems green to purple (drying tan) with dark lenticular vertical striations when young, sparsely to densely pubescent, not much compressed when dried in a plant press, becoming light brown and woody with age; upper sympodial branching points monochasial and dichasial. Leaves simple, the leaves of the upper sympodia usually paired and unequal in size, the larger ones with blades (4.6) 6.3–11.8 (15) × (1.9) 2.5–6.5 (8.8) cm, the smaller ones with blades 1.7–5.2 (8.5) × 0.9–3.9 (6.3) cm, the leaf pairs similar in shape, the blades ovate to elliptic, chartaceous, moderately pubescent, the trichomes usually densely spreading outward (towards the margins) along the abaxial veins, especially at the base of the main vein, the base usually cuneate (rarely attenuate), sometimes oblique, the margin entire, usually irregularly undulate (rarely coarsely dentate), the apex acuminate, the petiole 0.5–3.3 cm long, the larger leaf blades with 4–6 primary veins on each side of the midvein. Flowers solitary or in groups of 2–7 (10), axillary, oriented horizontally; peduncles absent; pedicels (8) 13–33 mm long and erect in flower, (15) 18–33 (40) mm long and erect in fruit, moderately pubescent; calyx 2.3–3.5 (4.1) mm long, 2.5–3.5 (4) mm in diameter, obconic to campanulate, moderately to densely pubescent, the margin truncate, with 10 erect, linear appendages (0.5) 1–4.3 mm long emerging 0.25–0.5 mm below the calyx rim; fruiting calyx enlarged, bowl-shaped to rotate, 1.5–3.2 mm long, 5–8.5 mm in diameter, the appendages to 6 mm long; corolla (0.7) 0.9–1.8 cm long, rotate in orientation, entire in outline, with abundant interpetalar tissue, adaxially white to pale violet with purple stripes along the major veins, with three green spots located between the short stamens, glabrous, abaxially green on the lobes, moderately puberulent near the veins; stamens unequal, straight, the four short filaments 1–2 (3) mm long, the one long filament (2.75) 3.5–4.2 (5) mm long, glabrous, the anthers 2.5–4.5 mm long, lanceolate, free of one another, yellow, glabrous, poricidal at the tips, the pores ovate, dehiscing toward the style or distally, not opening into longitudinal slits; pistil with glabrous ovary, the style 6–9 mm long, linear (sometimes curved upward at the tip), glabrous, the stigma oblong, decurrent down two sides. Fruit a berry, 6–11 mm long, 6–10 (15) mm in diameter, globose, red at maturity, glabrous, lacking sclerotic granules. Seeds 10–30 per fruit, (2) 2.5–4 × 2–3 mm, flattened, reniform in outline, with deep notch on side (usually more than 0.5 mm deep), yellow-orange, the surface reticulum with minute serpentine pattern and shallow luminae.

##### Chromosome number.

Unknown.

##### Distribution and habitat.

**Figure 109.**
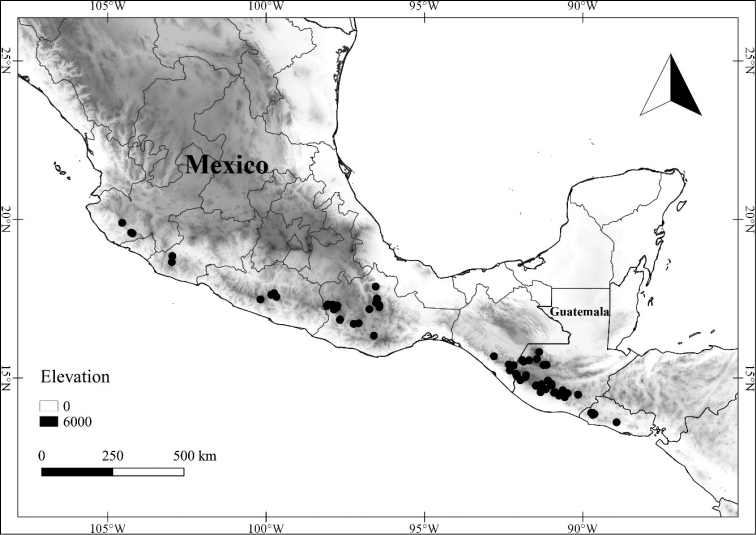
Map of geographic distribution of *L. tricolor* based on herbarium specimen data.

Mexico (Chiapas, Guerrero, Jalisco, Michoacán, Oaxaca), Guatemala (Chimaltenango, Escuintla, Guatemala, Huehuetenango, Quetzaltenango, Quiché, Sacatepéquez, San Marocis, Sololá), and El Salvador on steep slopes, in ravines, and in semi-disturbed areas such as roadside thickets, path edges, and disturbed agricultural areas in mixed broadleaved forest, oak forest, oak/pine forest, fir forest, and cloud forest, 2100–3000 m in elevation (Fig. 109).

##### Common names and uses.

None known.

##### Phenology.

Flowering specimens have been collected from February through October; specimens with mature fruits have been collected January through December. Field observation of the corollas indicates that the corollas are open in the early morning and closed by late morning (Dean et al. 2017a).

##### Preliminary conservation status.

*Lycianthes tricolor* is a widespread species ranging from southwestern Mexico to El Salvador, represented by 82 collections and occurring in seven protected areas. The EOO is 22,404.475 km^2^, and the AOO is 308 km^2^. Based on the IUCN (2019) criteria, the preliminary assessment category is Least Concern (LC).

##### Discussion.

This species is very similar to *Lycianthes arrazolensis* from which it can be distinguished by seed shape. The seeds of *L. tricolor* have a definite sharp notch that is usually deeper than 0.5 mm, whereas the seeds of *L. arrazolensis* lack this notch. In some Mexican *L. arrazolensis* populations in the states of Morelos and México, the seeds are shallowly indented on one side, but this indentation is less than 0.5 mm and usually less than 0.25 mm, and never sharply notched (Dean et al. 2017a). Non-fruiting specimens can be challenging to identify. The following non-seed characters can be helpful: the calyx rim of *L. arrazolensis* tends to be more prominent, often protruding beyond the appendage insertion by over 0.5 mm, while the calyx rim of *L. tricolor* is usually less than 0.5 mm; the appendages of *L. arrazolensis* tend to bend away from the rim, exposing the rim, while the appendages of *L. tricolor* are oriented closer to the rim and corolla, hiding the rim; the pores of the short stamens in *L. arrazolensis* usually face away from the style, while those of *L. tricolor* usually face toward the style; the flowering pedicels of *L. tricolor* are usually 13–33 mm long, becoming much longer in fruit, while those of *L. arrazolensis* are usually less than 15 mm in flower and 21 mm in fruit (however shorter pedicels in *L. tricolor* are found in Guatemala and Chiapas); and the leaves of typical *L. arrazolensis* tend to have obvious geminate leaf pairs with elliptic to obovate blades and leaf bases often attenuate into the petiole, while in *L. tricolor* the small geminate leaf often abscises early, and the laminas are more ovate with a less-attenuate leaf base (Dean et al. 2017a).

##### Representative specimens examined.

**Guatemala. Chimaltenango**: Reserva Natural Privada El Encanto de Tecpán, Caserlo Panimachavac, Aldea Chajaljya, Tecpán Chimaltenango, 14.8142, -90.9872, 2317 m, 8 Apr 2014, *B. Escobar 87* (BIGU). **Escuintla**: Volcán de Agua of Finca Rosario de La Vista Hermosa, [14.4411, -90.7506], 6000–8000 ft, 22 May 1971, *R.L. Wilbur 14748* (DUKE). **Guatemala**: along old road to San Lucas, vicinity of San Rafael, 1800 m, 27 Sep 1972, *A. Molina 27614* (MEXU, TEX). **Huehuetenango**: Chanximil, Aldea San Martín, todos Santos Cuchumatán, 15.5531, -91.6986, 2185 m, 16 Sep 2006, *J. Morales 3889* (MO). **Quetzaltenango**: Mpio. Zunil, road to Fuentes Georgiñas, 4 km S of Zunil, northwest slopes of Volcán Zunil, [14.7542, -91.4828], 2442 m, 4 Jan 2009, *T. Sultan Quedensley 7013* (BIGU). **Quiché**: Nebaj, about 4 km W, 6700 ft, 11 Jun 1964, *E. Contreras 4953* (MO, LL). **Sacatepéquez**: Volcán de Agua, [14.4708, -90.7392], 2500 m, 15 Aug 2003, *P. García 26* (BIGU, MEXU, TEX). **San Marcos**: 20 miles S of San Marcos along road from San Raphael, 2100 m, 13 Jul 1977, *T. B. Croat 41006* (MO). **Sololá**: Mpio. San Pedro La Laguna, Volcán San Pedro, [14.6564, -91.2672], 2500 m, 27 Jul 2005, *P. Pardo 665* (BIGU). **Mexico. Chiapas**: Reserva de la Biosfera El Triunfo, sendero Finca Prusia-Campamento El Triunfo, 22.15, -99.5667, 1900 m, 12 Nov 2004, *N. Martínez- Meléndrez 587* (MEXU). **Guerrero**: Omiltemi, Barranca Potrerillos, [17.5595, -99.6759], 2170 m, 28 Nov 1993, *C. González 241* (MEXU). **Jalisco**: parte alta del ejido de Pabelo, cerca del predio Las Iglesias, 4.4 km al sur-suroeste de Santa Monica (Mpio. Ayutla) y 4 km al este-noreste de Plaza de Gallos, 19.8922, -104.5464, 2120–2160 m, 12 Aug 2012, *P. Carrillo-Reyes 6743* (IBUG, IEB, MEXU). **Michoacán**: Mpio. Coalcomán, 14 km al SE de Varaloso, sobre el camino a Barranca Seca, 18.6536, -102.9769, 2000 m, 22 Dec 2007, *V. Steinmann 6121* (DAV). **Oaxaca**: Dto. Sol de Vega, Pena Ahumada, 16.7256, -97.0781, 2 Sep 2006, *A. Zárate Marcos 407* (IEB).

#### 49 Lycianthes venturana

Taxon classificationPlantae

E.Dean, Phytoneuron 2014–42: 1 (2 Apr 2014)

Fig. 110

##### Type.

México. Puebla. Mpio. Teziutlán: Agua de Obispo, bosque de encino en cañada, [19.815, -97.36], 1350 m, 25 Nov 1976, *F. Ventura A. 13635* (holotype: MEXU [acc. # 918864]; isotype: IEB [acc. # 114957]).

**Figure 110.**
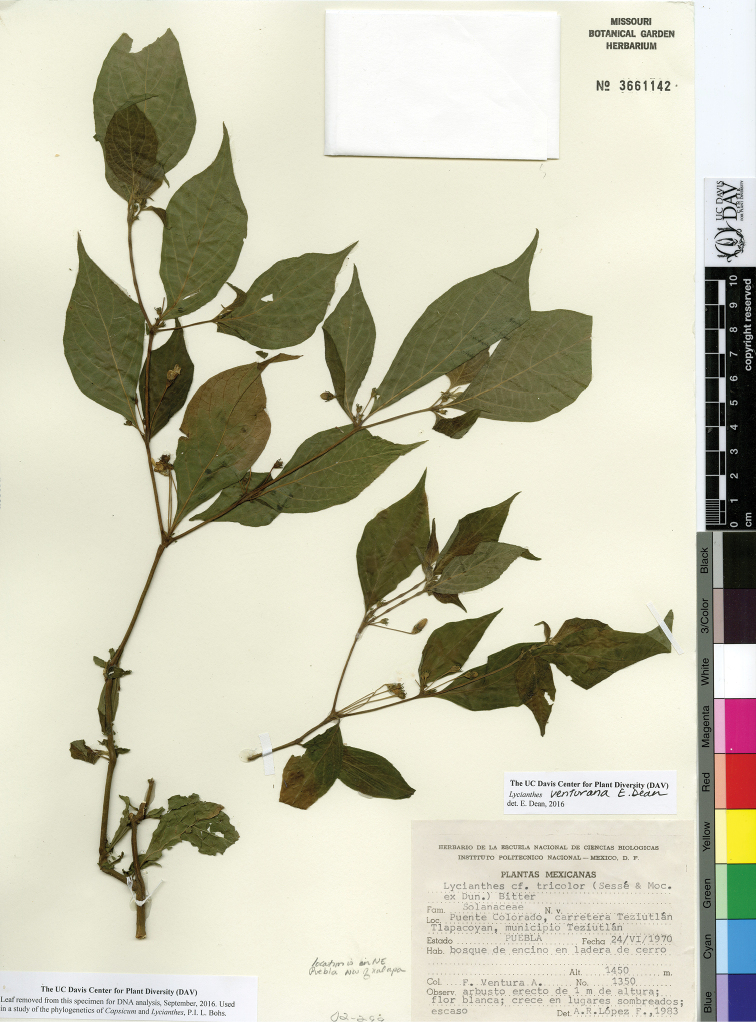
Image of herbarium specimen of *L. venturana*, *Ventura 1350* (MO). Specimen used with permission from the Missouri Botanical Garden (http://www.tropicos.org).

##### Description.

Shrub, 0.5–1.8 m tall. Indument of tan, uniseriate, multicellular, simple, eglandular, appressed-ascending to ascending trichomes 0.25–1.25 mm long. Stems green with pale vertical lenticular streaks when young, glabrous to sparsely pubescent, not much compressed when dried in a plant press, becoming brown and woody with age; upper sympodial branching points mostly monochasial, some dichasial. Leaves simple, the leaves of the upper sympodia usually paired and unequal in size, the larger ones with blades 6.5–13.3 × 3.7–6.5 cm, the smaller ones with blades 4–8.5 × 1.9–4.9 cm, the leaf pairs similar in shape, the blades ovate, elliptic, or obovate, thin chartaceous, glabrous to sparsely pubescent (densest along the veins), the base cuneate to attenuate, sometimes oblique, the margin entire, usually undulate, the apex acuminate, the petiole 0.2–1.5 (2) cm long, the larger leaf blades with 4–6 primary veins on each side of the midvein. Flowers solitary or in groups of 2–6 (10), axillary, oriented horizontally; peduncles absent; pedicels 13–28 mm long and erect in flower, 18–25 mm long (probably longer) and erect in fruit, glabrous to sparsely pubescent; calyx 1.5–2.5 mm long, 1.75–2.5 mm in diameter, urceolate to campanulate, glabrous to sparsely pubescent, the margin truncate, with 10 spreading linear appendages 1–3.5 mm long emerging 0.3–0.5 mm below the calyx rim; fruiting calyx enlarged, widely bowl-shaped, ca. 2 mm long, 4 mm in diameter, the appendages to 4 mm long (possibly longer); corolla (0.5) 0.9–1.4 cm long, campanulate to reflexed in orientation, mostly entire in outline (with shallow notches), with abundant interpetalar tissue, sometimes the interpetalar tissue tearing as the corolla opens, adaxially white, glabrous, abaxially color unknown, sparsely pubescent near the major veins; stamens unequal, straight, the four short filaments 1.25–1.5 mm long, the one long filament 4–5 mm long, glabrous, the anthers 3.5–4 mm long, lanceolate, free of one another, yellow, glabrous, poricidal at the tips, the pores ovate, all dehiscing toward the style, not opening into longitudinal slits; pistil with glabrous ovary, the style 7–9 mm long, linear, curved upward at tip, glabrous, the stigma oblong-capitate, slightly bilobed. Fruit a berry, ca. 6 mm long, 4 mm in diameter (probably larger), ovoid, usually apiculate due to persistent style base, color unknown, glabrous, lacking sclerotic granules. Seeds not yet seen.

##### Chromosome number.

Unknown.

##### Distribution and habitat.

**Figure 111.**
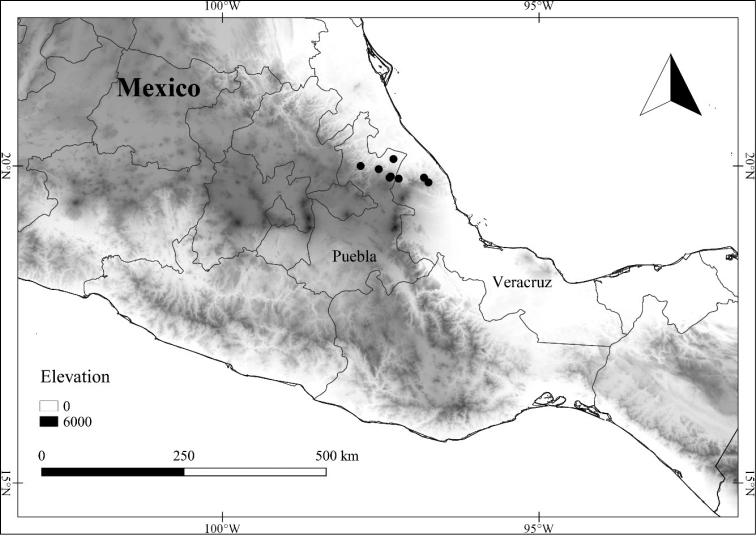
Map of geographic distribution of *L. venturana* based on herbarium specimen data.

Mexico (Puebla, Veracruz) in disturbed broadleaved cloud forest and oak forest, in shady canyons and on mountainsides, 1250–1450 m in elevation (Fig. 111).

##### Common names and uses.

None known.

##### Phenology.

Flowering specimens have been collected from April to November; specimens with mature fruit have been collected in November. The diurnal corolla movements are not known, however the corollas on specimens are usually closed, indicating that the flowers are probably only open in the early morning.

##### Preliminary conservation status.

*Lycianthes venturana* is a rarely collected species of eastern Mexico, represented by 8 collections, none of which is from a protected area. The EOO is 2,022.587 km^2^, and the AOO is 32 km^2^. Based on the IUCN (2019) criteria, the preliminary assessment category is Endangered (EN).

##### Discussion.

*Lycianthes venturana* is known from limited material from which mature fruit size and color, seed type, and corolla color and shape cannot be exactly ascertained. It is morphologically similar and most likely related to the species of series *Tricolores* (Dean et al. 2017a). Of those species, it is most similar to *L. jalicensis* in being nearly glabrous throughout, especially on the calyx and corolla, but it is distinguished from that species by having a smaller calyx as well as being geographically disjunct. The corolla is likely white and probably has greenish coloration along veins of the lobes on the abaxial side. Whether or not the corolla has the small greenish spots or glands present in related species, such as *L. tricolor*, is not known, because they do not persist well on pressed material, and the information is not included in label data. The fruits of the type specimen are dark in color, usually apiculate, 6 mm long and 4 mm wide. Whether these fruits are mature is not known. The apiculate tip to the fruits is formed from the persistent base of the style (Dean et al. 2017a).

##### Representative specimens examined.

**Mexico. Puebla**: San Juan Tahitic Village, trail to Zapotepic, locality Hueyaktepet, next to Arroyo de Huaxkonta, 19.9503, -97.5311, 1165 m, 2 Nov 2017, *T.B. Croat 107297* (DAV); **Veracruz**: “El Siete,” 19.8, -97.2167, 1445 m, 10 Jul 2008, *T. Krömer 2556* (MO).

### Excluded taxa

#### Lycianthes multiflora

Taxon classificationPlantae

Bitter, Abh. Naturwiss. Verein Bremen 24 [preprint]: 361. 1919

Lycianthes brevipes Bitter, Abh. Naturwiss. Verein Bremen 24 [preprint]: 369. 1919. Type: Guatemala. No location given, [1840?], *E. von Friedrichsthal s.n. (541?)* (holotype: W [acc. # 0074705; F photo negative “Types of the Vienna Herbarium” no. 33125]).
Lycianthes ferruginea (Vahl) Bitter var. firmior Bitter, Abh. Naturwiss. Verein Bremen 24 [preprint]: 340. 1919. Type: Costa Rica. [San José]: Descente de la Ardilla près San Marcos, 900–1355 m, Mar 1893, *A. Tonduz 7666* (lectotype designated by Dean and Reyes 2018a pg. 41: BR [000000552844]; isolectotype BR [000000552877]).
Lycianthes multiflora Bitter var. plicitomentosa Bitter, Abh. Naturwiss. Verein Bremen 24 [preprint]: 363. 1919. Type: Costa Rica. [Alajuela?]: Naranjo, no date, *A. Oersted 1384* (lectotype designated by Dean and Reyes 2018a pg. 43: C [C0021744]; isolectotype: C [C10021743]).
Lycianthes multiflora Bitter var. extustomentosa Bitter, Abh. Naturwiss. Verein Bremen 24 [preprint]: 363. 1919. Type: Costa Rica. Cartago: Turrialba, no date, *A. Oersted 1386* (holotype: C [C10021742]).
Lycianthes pittieri Bitter, Abh. Naturwiss. Verein Bremen 24 [preprint]: 360. 1919. Type: Costa Rica. Cartago: by San Lorenzo de Dota, 1250 m, 3 Apr 1890, *H. Pittier & T. Durand 2277* (holotype: BR [BR0000024940924]).
Lycianthes dominicana C.V.Morton & Standl., Publ. Field. Mus. Nat. Hist. Bot. Ser. 18: 1057. 1938. Type: Costa Rica. Santa Domingo de Vara Blanca, 2200 m, Feb 1937, *M. Valerio 1550* (holotype: F [0072909F, acc. # 888370])
Lycianthes hawkesiana D’Arcy, Ann. Missouri Bot. Gard. 60: 641. 1974 (1973). Type: Panama. Bocas del Toro: Changuinola Valley, 14 Jan 1924, *V. Dunlap 327* (holotype: US [cited by D’Arcy, not seen]; isotype F [0072911F, acc. # 635688]).
Lycianthes luteynii D’Arcy, Ann. Missouri Bot. Gard. 60: 643. 1974 (1973). Type: Panama. Chiriquí: 5.2 miles northwest of El Hato del Volcán on the road to Costa Rica, 5500 ft, 22 Jun 1970, *J. Luteyn 836* (holotype: MO [acc. # 2140705]).

##### Type.

Costa Rica. Cartago: Las Vueltas, Tucurrique, 635 m, Jan 1899, *A. Tonduz 13173* (lectotype designated by Dean and Reyes 2018a, pg. 43: M [M-0165975]; isolectotypes: B [F photo negative # 2584], BM [BM000775268], ENCB [ENCB003797], G [G00343135], GH [00934882], K [K000585740], LD [1688216], US [00604490, 00027891], W [acc. # 1903-12658]).

We have not been able to confirm the existence of the shrub/vine *L. multiflora* in Guatemala; we have only seen specimens of *L. multiflora* from Belize, Nicaragua, Costa Rica, and Panama. The species was originally described from Costa Rica (Bitter 1919), and most of the names we have placed in synonymy are based on specimens from Costa Rica or Panama. An exception to this is the name *L. brevipes*, which Bitter (1919) based on a collection made by the Austrian botanist Emanuel von Friedrichsthal (*Friedrichsthal s.n*.). Although the preprinted label with the specimen says Guatemala, Hemsley (1887) wrote that all of the Friedrichsthal collections that Hemsley encountered at the Kew Herbarium were actually collected in Nicaragua and Costa Rica. In fact, Bitter, in his protologue for the species, wrote the location as “Guatemala? Nicaragua? No special location”; therefore, it is probable that the type material of *L. brevipes* was not collected in Guatemala.

#### Lycianthes synanthera

Taxon classificationPlantae

(Sendtn.) Bitter, Abh. Naturwiss. Verein Bremen 24 [preprint]: 499. 1919

Solanum synantherum Sendtn., Flora 29 (13): 194 [as 178]. 1846. Type: Guatemala [Costa Rica. Province Alajuela], Monte Aguacate, *E. von Friedrichsthal 1292* (lectotype designated here: W [acc. # 0003083]; isolectotypes: W [acc. # 0003081, acc. # 0003082], F [F0073153F, acc. # 871594]).

##### Type.

Based on *Solanum synantherum* Sendtn.

We have not been able to confirm the existence of the usually epiphytic shrub/vine *L. synanthera* in Guatemala, and it likely does not occur there; we have located and examined many specimens of *L. synanthera* from Nicaragua, Costa Rica, and Panama. The type material of *Solanum synantherum* was collected by Friedrichsthal at Monte Aguacate. His labels were preprinted with the word “Guatemala,” however Monte Aguacate is located in Costa Rica, Province Alajuela. In her thesis, Reyes Cornejo (2015) cited one specimen of *L. synanthera* from Guatemala, *Steyermark 41657* (US), which is available as an online image, and this specimen is not *L. synanthera*; it likely is *Cuatresia*. All the Guatemalan specimens at herbarium MEXU that were previously identified as *L. synanthera* have been reidentified by the first author as *L. nitida*, *L. heteroclita*, or *Witheringia*.

In his protologue for *Solanum synantherum*, Sendtner (1846) just cited the one collection of Friedrichsthal (*Friedrichsthal 1292*) with no specific herbarium cited. We located duplicates of this collection (syntypes) at both W and F: W [acc. # 0003083, acc. # 0003081, acc. # 0003082] and F [F0073153F, acc. # 871594]. We selected one of the specimens at W (acc. # 0003083) as the lectotype.

### Difficult to place specimens.

**Specimen Group A**

**Mexico. Chiapas**: Near summit of Chuchil ton, northeast of Bochil, Mpio. San Andrés Larráinzar, 2700 m, 3 Aug 1972, *D. Breedlove 26814* (MO); Mpio. San Cristóbal de Las Casas, Santa Cruz en San Felipe, 15 Nov 1986, *A. Méndez Ton & de Lopez 9845* (NY, MO); Mpio. La Independencia, third ridge along logging road from Las Margaritas to Campo Alegre, 2300 m, 18 Feb 1973, *D. Breedlove 33679* (MO). This group of collections somewhat resemble *L. sideroxyloides*, but they lack calyx appendages (represented by bumps on the calyx), and the calyx rim is membranaceous and undulate, resembling sepal lobes. The trichomes are a mixture of multangulate-stellate and geminate-stellate, the stamens are equal, and the corolla is stellate and deeply divided, similar to the features of *L. sideroxyloides*. This group of specimens may represent a new species.

**Specimen Group B**

**Guatemala. San Marcos**: west facing slope of the Sierra Madre Mountains, near Aldea Fraternidad, between San Rafael Pie de la Cuesta and Palo Gordo, 1800–2400 m, 10–18 Dec 1963, *L.O. Williams 26281* (NY, US2542562); same location and date, *L.O. Williams 25998* (NY); Zacapa: slopes of Monte Virgen, around of summit of mountain, 2200–2400 m, 12–13 Jan 1942, *J.A. Steyermark 42683* (NY). This group of fruiting collections somewhat resemble L. chiapensis var. sparsistellata, but they lack calyx appendages (represented by bumps on the calyx), and the calyx rim is membranaceous and undulate, and somewhat torn. This group of specimens may represent a new species or they may just be L. chiapensis var. sparsistellata with atypical calyces.

**Specimen Group C**

**Mexico. San Luis Potosi**: km 14.5 carretera Ciudad Valles-Rio Verde, 1157 m, 9 Jul 2000, *E. M. Lira Charco 1458* (MEXU1277353). This fruiting collection matches Lycianthes scandens var. scandens but it is out of range for that species.

**Specimen Group D**

**Mexico. Guerrero**: Mpio. General Heliodoro Castillo, 35 km N of El Paraiso on road to Puerto del Gallo, 2000–2100 m, 9 Jun 1985, *W. Thomas 3750* (NY). This flowering collection resembles *L. fredyclaudiae*, and it shares the rebranched multangulate-stellate trichome type shared by *L. fredyclaudiae*, *L. hortulana*, and *L. breedlovei*, but it is out of range of any of those species.

**Specimen Group E**

**Mexico. Veracruz**: Mpio. Catemaco. Bastonal, 10 km NE de Tebanca, carretera Catemaco-Tebanca, 1600 m, *S. Sinaca C. 863* (MEXU422574, MEXU424171). This fruiting collection resembles *L. rafatorresii*, but the leaves have rounded apices that differ from that species, and the inflorescence arrangement resembles that of *L. multiflora*, with the inflorescences in the axils of leafless terminal sympodia.

In addition to the above specimen groups, there are difficult to place specimens among the herbs of series *Meizonodontae* that were assigned to three Specimen Groups (A, B, C) by the first author in Dean (2004). Group C of Dean (2004) is a particularly interesting group of collections that occur in the mountains of Guerrero ringing the Cuenca del Balsas (Sierra Madre del Sur and Sierra de Cacahuamilpa), growing in oak, oak-pine or pine forest, 1750–2240 m. These specimens resemble *L. starbuckii* but are erect in habit, rather than prostrate, have more attenuate leaf bases, and sometimes have glabrous corolla lobes; the relationship of these populations to *L. starbuckii* deserves more study. *Dean 278a* and *278b* from the Sierra de Cacahuamilpa north of Taxco, Guerrero, were collected with *L. acapulcensis* and may be part of a hybrid complex at that site. Similarly, the Wagenbruth specimens cited below were collected in an area (Malitepec) where *L. acapulcensis* has been collected. We encountered more collections belonging to this specimen group in preparing this paper and we list all the collections here: **Mexico. Guerrero**: Mpio. Tetipac, 2.4 rd mi from the town of Tetipac along the Tetipac-Taxco rd, 5800–6200 ft, 4 Oct 1991, *Dean 278a* (DAV, XAL), *278b* (DAV, XAL); District Mina, Yesceros, 2200 m, 7 Jul 1939, *Hinton et al. 14403* (GH no #, MEXU29068, MO1208513, LL no #); Mpio. Xalpatláhuac, 32 km al S de Tlapa, camino a Malinaltepec, 2240 m, 26 Jun 1982, *E. Martínez S. 1101* (NY); 18 km south of Taxco, carretera a Ixcateopan, 1880 m, 7 Jul 1982, *J. C. Soto Nùñez 4028* (MEXU); a 16 km al E de Atlixtac, camino de Chilapa a Tlapa, no elevation, 7 Dec 1982, *O. Téllez V. 6097* (MEXU1051891); Mpio. Malinatepec, Malinatepec, 1900 m, 24 Jun 1991, *Wagenbreth 667* (MEXU).

Finally, at the end of their paper on series *Tricolores*, Dean et al. (2017a) assigned difficult to place Mexican and Guatemalan specimens belonging to series *Tricolores* into six collection groups. Two of those groups (A and E) have since been described as new species and are covered in this paper: *L. grandifolia* (Group A) and *L. mariovelizii* (Group E). The other collections remain unplaced.

## Supplementary Material

XML Treatment for Lycianthes

XML Treatment for Lycianthes acapulcensis

XML Treatment for Lycianthes amatitlanensis

XML Treatment for Lycianthes anomala

XML Treatment for Lycianthes armentalis

XML Treatment for Lycianthes arrazolensis

XML Treatment for Lycianthes barbatula

XML Treatment for Lycianthes breedlovei

XML Treatment for Lycianthes caeciliae

XML Treatment for Lycianthes ceratocalycia

XML Treatment for Lycianthes chiapensis

XML Treatment for Lycianthes chiapensis var. chiapensis

XML Treatment for Lycianthes chiapensis (Brandegee) Standl. var. sparsistellata

XML Treatment for Lycianthes ciliolata

XML Treatment for Lycianthes connata

XML Treatment for Lycianthes cuchumatanensis

XML Treatment for Lycianthes dejecta

XML Treatment for Lycianthes fredyclaudiae

XML Treatment for Lycianthes geminiflora

XML Treatment for Lycianthes glabripetala

XML Treatment for Lycianthes gongylodes

XML Treatment for Lycianthes gorgonea

XML Treatment for Lycianthes grandifolia

XML Treatment for Lycianthes heteroclita

XML Treatment for Lycianthes hintonii

XML Treatment for Lycianthes hypoleuca

XML Treatment for Lycianthes inconspicua

XML Treatment for Lycianthes jalicensis

XML Treatment for Lycianthes limitanea

XML Treatment for Lycianthes manantlanensis

XML Treatment for Lycianthes mariovelizii

XML Treatment for Lycianthes michaelneei

XML Treatment for Lycianthes moziniana

XML Treatment for Lycianthes moziniana var. moziniana

XML Treatment for Lycianthes moziniana (Dunal) Bitter var. margaretiana

XML Treatment for Lycianthes moziniana (Dunal) Bitter var. oaxacana

XML Treatment for Lycianthes nitida

XML Treatment for Lycianthes ocellata

XML Treatment for Lycianthes orogenes

XML Treatment for Lycianthes peduncularis

XML Treatment for Lycianthes pilifera

XML Treatment for Lycianthes pringlei

XML Treatment for Lycianthes purpusii

XML Treatment for Lycianthes quichensis

XML Treatment for Lycianthes rafatorresii

XML Treatment for Lycianthes rantonnetii

XML Treatment for Lycianthes rzedowskii

XML Treatment for Lycianthes scandens

XML Treatment for Lycianthes scandens var. scandens

XML Treatment for Lycianthes scandens (Mill.) M.Nee var. flavicans

XML Treatment for Lycianthes sideroxyloides

XML Treatment for Lycianthes starbuckii

XML Treatment for Lycianthes stephanocalyx

XML Treatment for Lycianthes surotatensis

XML Treatment for Lycianthes textitlaniana

XML Treatment for Lycianthes tricolor

XML Treatment for Lycianthes venturana

XML Treatment for Lycianthes multiflora

XML Treatment for Lycianthes synanthera

## Appendix I

**Additional specimens examined**

**1. *Lycianthes acapulcensis***

**Costa Rica. Guanacaste**: Cantón de La Cruz, Cordillera de Guanacaste, 9.9708, -85.5831, 350–450 m, 15 Jul 1996, *Morales 5512* (NY); Puntarenas: Monteverde, 1–2 km downstream from the village of San Luis along the Río Guacimal, 10°16'N, 84°50'W, 550–820 m, 30 May 1992, *Haber & Joyce 11197* (INB); **San José**: El Brazil, rd between El Brazil and the electric substation of the River Virilla, along tributary of the Virilla River, 9°56'N, 84°14'W, 800 m, 29 Sep 1993, *Dean 361* (CR, DAV, INBIO, NY); along rd between Guacimal and Ciruelas, at the crossing of the Ciruelas River, near railroad station, 9°59'N, 84°16'W, 700 m, 29 Sep 1993, *Dean 362* (CR, DAV, INBIO, UC); Villa Colon, Finca San Luis, 800 m, 26 May 1965, *Jiménez-M. 3214* (CR, F, MO, NY); Cantón de Acosta, Valle del Candelaria, 9.7756, -84.1725, 700 m, 22 Apr 1995, *Morales 3957* (NY).

**El Salvador. Department unknown**: Finca San Nicolás, 371 m, May 1923, *Calderón 1643* (US); **Ahuachapán**: San Benito, al E del pie del semillerón, 13.8167, -89.9333, 100 m, 26 Apr 1993, *Sandoval 1202* (MO); same location, 18 Apr 1995, *Sandoval ISB893* (MO, NY); **Sonsonate**: vicinity of Izalco, [13.7333, -89.6500], 391 m, 19–24 Mar 1922, *Standley 21850* (GH, US).

**Guatemala. Department unknown**: S. Sebastian, Sep 1874, *Bernoulli & Cario 2404* (B destroyed, photos NY, GH, G); **Huehuetenango**: Mpio. Jacaltenango, 15.6744, -91.7353, 1627 m, 11 Jul 2006, *Véliz 17055* (BIGU); **Schitepéquez**: Mpio. Patutlul, Finca Los Tarrales, 14.5363, -91.170, 300 m, 30 Jul 2004, *Montiel s.n.* (BIGU).

**Honduras. Intibuca**: Mpio. Yamaranguila, 3.7 mi WNW of La Esperanza along the road from La Esperanza to Gracias, Sierra de Opalaca, 14.3119, -88.2253, 1860 m, 22 Jun 1994, *Davidse 35237* (DAV, MO, NY).

**Mexico. Chiapas**: Mpio. Tuxtla Gutiérrez, El Ranchito, sobre la carretera de los miradores, Parque Nacional Cañon del Sumidero, 16.8191, -93.0736, 1301 m, 24 Aug 2007, *Espinosa-Jiménez 306* (MO); Mpio. Jiquipilas, no exact location, 16.60, -93.55, 580 m, 26 Jun 1991, *Ferrera S. 186* (MEXU); Mpio. Tuxtla Chico, a 3 km al W de Cacahoatan, camino al Río Suchiate, [14.99, -92.19], 380 m, 27 Apr 1987, *Martínez-Salas 20348* (MEXU, MO); Mpio. Arriaga, Ejido López Mateos, 16.3558, -93.9661, 500 m, 10 Jun 2002, *Reyes-García 5122* (DAV, MEXU); Mpio. Cacahoatán, Monte Bello, 450 m, 19 Apr 1985, *Ventura 1541* (ENCB, G, UC, XAL); **Colima**: Mpio. Comala, Rancho El Jabalí, ca. 22 km NNW of city of Colima, near Jalisco state line, S and E sides of Lago Calabozo (near 19°26.25'N, 103°40.5'W), 1475 m, 29 Aug 1988, *Sanders 8542* (NY); Mpio. Comala, Rancho El Jabalí, 22 air km NNW of Colima in the SW foothills of the Volcán de Colima, on the Colima/Jalisco border,19°26.65'N, 103°41.9'W, 1350 m, 12 Jul 1991, *Vázquez-V. 837* (NY); Mpio. Comala, Rancho El Jabalí, 22 air km NNW of Colima in the SW foothills of the Volcán de Colima, on the Colima/Jalisco border, along road from headquarters to Lago Epazote, 19°27.2'N, 103°41.6'W, 1300 m, 14 Jul 1991, *Vázquez-V. 876* (CAS, NY); Mpio. Comala, Rancho El Jabalí, 22 air km NNW of Colima in the SW foothills of the Volcán de Colima, on the Colima/Jalisco border, 19°26.8'N, 103°41.9'W, 1300 m, 15 Jul 1991, *Vázquez-V 887* (DAV, MEXU, NY, UC, UCR); SW foothills of the Volcán de Colima; Rancho El Jabalí, 22 km (airline) NNW of Colima, Colima/Jalisco line passes through ranch. Western side of Lago Calabozo, 19.3383, -103.6792, 1400 m, 8 Aug 1991, *Vázquez-V 1082* (UCR); SW foothills of the Volcán de Colima, Rancho El Jabalí, 22 km (airline) N of Colima, Colima/Jalisco line passes through the ranch, at the edge of the river near Hacienda San Antonio, 19.4433, -103.7266, 1200 m, 3 Oct 1991, *Vázquez-V 1317* (UCR); **Guerrero**: S of Chilpancingo, 24 Jun 1935, *Clark 7196* (MO, NY); 1 mile west of Acahuizotla, 2800 ft, 9 Jun 1952, *Cooper 2528* (BRIT); 5 mi NE of Ocotito, [17.2894, -99.4247], 2900 ft, 13 Jun 1954, *Crisman 194* (TEX, UC); Mpio. Taxco, Just west of main part of town of Landa, along road to Ixcateopan, [18.5627, -99.6234], 1890 m, 12 Jul 1990, *Dean 209* (DAV, MEXU); Mpio. Tetipac, 2.4 rd mi from the town of Tetipac along the Tetipac-Taxco rd, [18.6291, -99.64834], Oct 1991, *Dean 278c* (XAL); Mpio. Mochitlan, along hwy 95 between Chilpancingo and Tierra Colorada, ca. 5.0 rd mi N of El Ocotito, W side of rd, [17.2593, -99.5151], 3000 m, 7 Oct 1991, *Dean 279* (DAV, MEXU, XAL); Mpio. Chochihualco, along rd between hwy 95 and Filo de Caballo, N of Chilpancingo, ca. 9 rd mi W of Xochipala, N side of rd, [17.7921, -99.5935], 6500 m, 7 Oct 1991, *Dean 280* (DAV, MEXU, NY, XAL); Mpio. Chilpancingo, Botanical Garden of the Universidad Autónoma de México at Chilpancingo, [17.5388, -99.5047], 4400 m, 8 Oct 1991, *Dean 281* (DAV, ENCB, MEXU, MO, NY, UC, XAL); same location, 5 Nov 1991, *Dean 312* (DAV, MEXU, NY, UC, XAL); Mpio. Taxco, Town of Landa, just W of main part of town, along rd to Ixcateopan, 1890 m, 6 Nov 1991, *Dean 313* (DAV, ENCB, MEXU, NY, UC, XAL); 3300 ft, *Gray 2986* (BRIT); Dist. Montes de Oca, Capiral, [16.9166, -99.7833], 780 m, 13 Jun 1937, *Hinton et al. 10315* (G, GH, K, LL, NY, S, UC); Dist. Mina, Manchón, [17.4333, -99.5500], 6 Jul 1937, *Hinton et al. 10517* (BM, DS, GH, K, LL, MEXU, NY, UC); Agua del Obispo, 3300 ft, 30 Jun 1952, *Mockford 2857* (BRIT); Mpio. Chilpancingo, Rincon de la Via, 17.2875, -99.4819, 700 m, 15 Jul 1960, *Kruse 306* (MEXU); Mpio. Zirandaro, en La Saiba Amarilla a 7 km al E de Guayameo, camino Guayameo-Los Placeres del Oro, 18.2975, -101.1870, 1150 m, 14 Jul 1982, *Martínez-S. 1387* (MEXU, NY); Mpio. Cutzamala de Pinzon, Cerro La Mesa del Caballo, 1 km al S del Aguacate al suroeste de la cañada Vieja, [18.6018, -100.7041], 7 Jul 1973, *Medrano 6149* (MEXU); Limón Mt, [17.4333, -99.55], 4000 ft, 2 Jul 1910, *Rusby s.n.* (NY); Landa, 5 km al SW de Taxco, camino a Ixcateopan, [18.5391, -99.6726], 1790 m, 7 Jul 1982, *Soto-N. 4000* (CAS, ENCB, MEXU, WIS); 11 km al S de Acahuizotla, Microondas El Fresno, 17.2608, -99.4819, 920 m, 18 Jul 2005, *Soto-N. 27083* (MEXU); 20.6 km al N de La Unión, 18.1608, -101.7549, 565 m, 21 Jul 2005, *Soto-N. 27147* (MEXU); road from Milpillas to Atoyac de Alvarez, 9 mi WSW of Xochipala, [17.75, -99.55], 5400 ft, 30 Jun 1982, *Thomas et al. 2845* (NY); Chilpancingo, Jardín Botánico de la Universidad Autónoma de Guerrero, 1330 m, 29 Jun 1978, *Toledo-C. 386* (XAL); Mpio. San Luis Acatlán, Arroyo Cumiapa, a 1.44 km en línea recta al noroeste de la Comisaría de Arroyo Cumpiapa, sobre el camino que va a Cerro Zapote, en el terreno del Sr. Lauro Cortez, 16.8825, -98.6266, 531 m, 2 Aug 2017, *Velasco G. 40590* (DAV); Mpio. San Luis Acatlán, Yoloxóochitle, Arroyo Naranja, a 1.45 km en línea recta, a 500 m de la carretera que va a Cuanacaxtitlán, en la parcela del finado Sr. Celestino Castellanos Bautista, a 10 m del arroyo, 16.8134, -98.6721, 636 m, 1 Sep 2017, *Velasco G. 40647* (DAV); Mpio. Acapulco, La Venta, 10 Jul 1966, *Villanueva-O. 10* (ENCB, F); Mpio. Malinaltepec, Malinaltepec, [17.2457, -98.6784], 1800 m, 4 Jun 1989, *Wagenbreth 35* (MEXU); **Jalisco**: Mpio. La Huerta, camino antiguo Chachalaca-Arroyo Zarco, [19.50, -105.05], 238 m, 28 Jul 1982, *Bullock 1217* (BH, MEXU); Mpio. Casimiro Castillo, 7–8 km NNE of Casimiro Castillo, Arroyo La Calera, [19.5962, -104.4284], 800–1000 m, 14 Jul 1988, *Cuevas 3066* (WIS); Mpio. Tecalitlán, 1.2 to 3.7 rd km along dirt rd to San Isidro that leaves old Colima-Tecalitlán road about 7 rd mi S of city of Tecalitlán, [19.4666, -103.3], 1200 m, 15 Aug 1991, *Dean 249* (DAV, XAL); Mpio. Tecalitlán, Sierra del Halo, ca. 2 rd mi along rd to San Isidro (or Jilotlan) that leaves old Colima-Tecalitlán rd ca. 7 rd mi S of Tecalitlán, [19.3171, -103.2695], 1340 m, 23 Nov 1991, *Dean 329* (DAV, MEXU, NY, UC, XAL); Mpio. El Tuito, About 3 rd mi N of El Tuito along hwy 200, footpath enters woods above a running river on W side of hwy, [20.3496, -105.3201], 2400 m, 27 Nov 1991, *Dean 332* (DAV, MEXU, NY, UC, XAL); Sierra de Manantlán, 3 km al SE de Lagunillas, La Mochota, Cuautitlán, 19°28'N, 104°11'W, 850 m, 1 Jul 1990, *De Niz 173* (WIS, ZEA); entre el Tuito y Puerto Vallarta, a 20 km de Puerto Vallarta y a 20 km de El Tuito, [20.5046, -105.3007], 19 Jul 1976, *Delgado-S. 358* (same as *Hernández 2609*) (MEXU); no exact locality, Diquet s.n. (NY); Mpio. Tecalitlán, alrededores de Plan de Lego, 51 km directamente al SSE de Ciudad Guzmán, al E de P. de Lego, brecha a Las Animas, [19.2981, -103.2524], 8 Aug 1988, *Fuentes-O. 496* (IEB, NY); Antiguo camino a Nacastillo, en la parte alta de la Estación, a 9.1 km al E de la carretera Puerto Vallarta-Barra de Navidad, [19.5880, -105.0307], 190 m, 7 Jul 1985, *Guadalupe-Ayala 21* (MEXU); 4 km S of Mazamitla, [19.8879, -103.03679], 1600 m, 7 Jul 1967, *Hernández-M. 407* (GH, MEXU); steep N-facing hills, 0.5 km S of Puente San Pedro, on road from Colima to Ciudad Guzmán, 19°20'N, 103°23'W, 1300–1400 m, 31 Jul 1960, *Iltis 609* (ENCB, WIS); Mpio. La Huerta, Rancho Cuixmala and environs, S side of Río Cuixmala, dirt road heading upriver (E), ca 3 km inland from Puerto Vallara-Barra de Navidad (Mex. 200) hwy, 19°25'N, 104°57'W, 9 Jul 1991, *Lott et al. 3655* (CAS, NY, TEX); Sierra del Halo, near a lumber road leaving the Colima hwy 7 miles SSW of Tecalitlán and extending SE toward San Isidro, about 2 mi from the hwy, [19.3151, -103.2514], 1400 m, 13 Aug 1957, *McVaugh 16154* (TEX; duplicate specimens at MEXU, NY, and TEX are *L. moziniana*); Sierra de Manantlán, 40.5 km al SE de Autlán, 1–2 km al NE de Telcruz, Cuautitlán, 19°29'N, 104°7'W, 1100 m, 18 Jul 1987, *Vázquez & López-V.* 4405 (DAV, UC, ZEA); **México**: Mpio. Ixtapan de la Sal, 2 rd km NE of the NE gates of the town of Ixtapan de la Sal, along hwy 55 (to Toluca), [18.8627, -99.6695], 1920 m, 12 Jul 1990, *Dean 210* (DAV, MEXU); same location and date, *Dean 211* (DAV, MEXU, UC, XAL); same location but 1830 m, 2 Nov 1991, *Dean 310* (BM, DAV, ENCB, MEXU, MO, NY, UC, XAL); Mpio. Temascaltepec, ca. 4–4.5 rd mi S of Temascaltepec along hwy 134, E and W sides of hwy, [18.9864, -100.0582], 2030 m, 7 Nov 1991, *Dean 314* (DAV, ENCB, MEXU, NY, UC, XAL); Cruz de los Pozitos, 18.9025, -99.7428, 2340 m, 20 Aug 2011, *Dorantes-Hernandez 408* (MEXU); Mpio. Ixtapan de la Sal, Barranca de Malinaltenango, cerca del Río, [18.7866, -99.7157], 1450 m, 14 Jul 1998, *García-Mendoza 6631* (MEXU); District Temascaltepec, Vigas, [19.0229, -99.9214], 1080 m, 30 Jul 1932, *Hinton et al. 1211* (BM, F, G); District Temascaltepec, Rincón, [19.0722, -99.9034], 1960 m, 10 Sep 1932, *Hinton et al. 1548* (GH, K, NY); Plaza del Gallos, [19.3667, -99.35], 1200 m, 27 Aug 1933, *Hinton et al. 4593* (BM, F, G, MO); Mpio. Malinalco, cañada San Miguel, paraje el Rincon de San Miguel, Barrio Santa Monica, [18.9527, -99.5008], 1855 m, 7 Jul 2013, *López-Zamora 85* (MEXU); Los Bejucos, Tejupilco, [18.7866, -100.5283], 700 m, 27 Aug 1954, *Matuda 31392* (MEXU); 2 km N of Ixtapan de la Sal along Hwy 55, [18.8627, -99.6694], 1650 m, 28 Jul 1964, *Mick 348* (WIS); Mpio. Tenancingo, Cerro de La Malinche, alrededores de petroglifo, 18.9213, -99.5934, 2302 m, 13 Jul 2011, *Rodríguez-Contreras 6229* (IBUG, XAL); 6 km S of Temascaltepec on Hwy 130, 18°57'N, 100°05'W, 2000 m, 3 Sep 1965, *Roe 1676* (ENCB, UC, WIS); 4 km S of Temascaltepec, 18°58'N, 100°05'W, 1800 m, 4 Sep 1965, *Roe 1739* (ENCB, UC, WIS); 2 km N of Ixtapan de la Sal, at km 145 on hwy 55, 18°50'N, 99°25'W, 1930 m, 9–10 Sep 1965, *Roe 1902* (ENCB, UC, WIS); Temascaltepec, cañada al oeste de la población, [19.0376, -100.0943], 1800 m, 4 Sep 1965, *Rzedowski 20836* (DS); **Michoacán**: District Coalcomán, Pto. Zarzamora, [18.8, -103.2333], 3 Aug 1939, *Hinton et al. 15037* (F, GH, LL, MO, NY, TEX); Mpio. Tancítaro, rd from Tancítaro to Apatzingán, [19.1833, -102.3167], 5000 ft, 30 Jul 1940, *Leavenworth 386* (F); Mpio. Ario de Rosales, just above Tacámbaro on highway to Pátzcuaro, [19.2333, -101.65], 1700 m, 20 Jul 1948, *Moore 4020* (A, GH, TEX); Mpio. Ario de Rosales, 4 km al S de Doctor Miguel Silva, sobre la carretera a la Huacana, [19.1368, -101.7215], 1500 m, 22 Jul 2001, *Rzedowski 53805* (MEXU); Cerca de Laguna verde, [19.8253, -100.6581], 7 Jul 1985, *Soto-Nuñez 9086* (MEXU); Mpio. Chinicuila, en Villa Victoria, [18.7556, -103.3678], 600 m, 15 Jul 1985, *Soto-Nuñez 9441* (IBUG, MEXU); Mpio. La Huacana, Sierra Las Cruces, 7.5 km (en línea recta) al SE de Los Ranchos, alrededores de la Ciénega del Plátano, 18.6875, -102.0853, 800 m, 9 Jul 2005, *Steinmann 5140* (DAV); **Morelos**: Mpio. Cuernavaca, Noroeste de La Barranca de Atzingo, [18.9454, -99.2754], 1800 m, 12 Aug 1987, *Estrada Loera 1708* (MEXU); Mpio. Huitzilac, Huitzilac, [19.0167, -99.2667], 1 Jul 1930, *Lyonnet 713* (MEXU – mixed collection with *L. rzedowskii*, NY, duplicate specimens at BM, GH, MO AND MEXU are *L. rzedowskii*); Cuernavaca, Tetela del Monte, Lomas, [18.9725, -99.2670], Aug 1955, *Lyonnet 550800023* (MEXU); barranca of Cuernavaca, [18.9, -99.2833], 5000 ft, 17 Jun 1904, *Pringle 13137* (BH, BRIT, C, CAS, K, GH, L, MO, S); Cuernavaca, *Schmitz s.n.* (GH, W); Cuernavaca, *Schmitz 1091* (W); **Oaxaca**: Mpio. Sn. S. Tecomoxtlahuaca, Sabino Solo, 5 km de San Sebstian Tecomoxtlahuaca, carretera Coicoyan de las Flores, 17.3167, -98.0500, 1710 m, 6 Oct 1994, *Calzada 19383* (NY); Sierra Madre del Sur, Mpio. Textitlán, Colonia Nueva, Ferreria Providencia, a settlement to the west of the town of Textitlán above the Río Tigre, along old undeveloped road to the west of the settlement above a canyon with drainages leading down to the Río Tigre, 16.6930, -97.3059, 1560 m, 7 Sep 2017, *Dean 9520* (DAV, MEXU); 13 mi E of Pinotepa Nacional on Mex Hwy 200, NW of Puerto Escondido, [16.3137, -97.87914], 27 May 1973, *Hansen 1525* (WIS, MEXU); Dto. Tehuantepec, Mpio. San Pedro Huamelula, cerro donde trabaja Francisco Siraco, 16.0388, -95.6800, 337 m, 24 May 2009, *Leyva-Marquez 124* (MO, NY); Dto. Yautepec, Mpio. San Bartolo Yautepec, El Zapote, 16.4717, -95.9464, 945 m, 17 Jun 2012, *López P. 3217* (DAV); Dto. Yautepec, Mpio. San Bartolo Yautepec, no exact location, 16.4017, -95.9981, 1016 m, 7 Apr 2012, *López P. 3298* (DAV); Mpio. San Miguel Chimalapa, Cabecera del arroyo de paso por el Mamey, al oeste-noreoeste del Cerro El Reten, ca. 24 km en línea recta al norte-NE de Zanatepec, 16.6833, -94.2500, 1600–1800 m, 2–4 Jul 1986, *Maya-Jimenez 3512* (MEXU); Dto. Yautepec, Mpio. Santa María Ecatepec, en el crucero a San Lorenzo, 16 km al suroeste de la Reforma, camino a Santa María Ecatepec, [16.2799, -95.8505], 15 Jul 1988, *Martínez-Ramírez 1490* (IBUG, MEXU); Dto. Pochutla, Mpio. San Miguel del Puerto, camino al Achiote, a 100 m de la entrada para el Tapesco, [15.9286, -96.0688], 443 m, 11 Jun 2002, *Pascual 479* (TEX); Dto. Pochutla, El Vigia, 16.0152, -96.1147, 680 m, 20 Jun 2006, *Pascual 1906* (IBUG, MEXU); Dto. Juxtlahuaca, Mpio. San Juan Mixtepec, cañada de San Lucas, 8 km al oeste de Cabecera Municipal de San Juan Mixtepec, [17.3007, -97.9089], 9 Nov 1988, *Reyes-Santiago 116* (MEXU); Mpio. San Juan Mixtepec, San Pedro Centro a 0.5 km sureste de San Juan Mixtepec, 17.3000, -97.8167, 1740 m, 10 May 1988, *Reyes-Santiago 240* (MEXU); Mpio. San Andres Paxtlan, La Venta, km 127–128 de la carretera Mex. 175, 26 km al sureste de Mihuatlan, camino a San Marcial Ozolotepec y San Sebastian Río Hondo, 16.1931, -96.5160, 2389 m, 21 Oct 2005, *Rodríguez-Contreras 4757* (IBUG, IEB); Dto. Pochutla, Mpio. San Miguel del Puerto, Cascadas del Llano de Horno, 15.9913, -96.1035, 720 m, 29 May 2006, *Sanchez-Martínez 1494* (IBUG); Dto. Sola de Vega, Mpio. Santiago Textitlán, Paraje Tierra de Yunta, 16.7836, -97.2976, 2330 m, 18 Jul 2006, *Sanchez-Martínez 1707* (IBUG, IEB, MEXU); Dto. Putla, 13 km al S de Copala o 2 km al S de la desviación a Tlaxiaco, hacia Putla, [17.566, -97.4166], 2600 m, 19 Jun 1982, *Torres-C. 598* (MEXU, MO, NY); Cerro Espinoso, Finca Montecristo entrado por Chacalapa 5 km al N de Pochutla carretera a Oaxaca, [15.7992, -96.4360], 31 May 1984, *Torres-C. 5242* (MEXU); 6 km al NE de Chacalapa en la brecha a la finca Ojo de Agua, [15.8649, -96.4291], 14 Jun 1985, *Torres-C. 6826* (MEXU, MO, NY, XAL); Dto. Pochutla, La Cueva, 31.6 km al N de Putla, [17.1156, -97.9326], 1470 m, 10 Aug 1985, *Torres-C. 7207* (MEXU); Dto. Sola de Vega, Mpio. Santiago Textitlán, Paraje Río Tigre, 16.6960, -97.2849, 1857 m, 16 Jun 2006, *Trujillo-Olazo 24* (MEXU); Dto. Pochutla, 6 km al NE de Chacalapa en la brecha a la finca Ojo de Agua, 14 Jun 1985, *Torres-C. 6826* (MEXU, MO, NY); Mpio. Miahuatlan, San José del Chilar, 17.7007, -96.9321, 683 m, 10 Nov 2009, *Vargas-Ponce 2084* (IBUG); Dto. Sola de Vega, Mpio. Santa Cruz Zenzontepec, Communidad de El Carrizal, 16.5333, -97.4500, 1040 m, 8 Jul 1993, *Weiss 236* (TEX); Dto. Sola de Vega, Río Tigre, 16.6944, -97.2843, 1562 m, 13 Aug 2006, *Zarate-Marcos 255* (MEXU). **Puebla**: Sierra de Chalchi, Jun 1945, *Miranda 3551* (MEXU) [This location is out of range for this species and this specimen needs research].

**Nicaragua. Managua**: Managua, [12.0833, -86.2667], 29 Jun 1927, *Chaves 278* (F, US); vicinity of Managua, [12.0833, -86.2667], Aug 1932, *Garnier 1179* (US); km 48 carretera al Ingenio Julio Guitrago (Montelimar), ca. 11°50'30"N, 86°30'10"W, 100 m, 25 Sep 1982, *Grijalva 1250* (MO); Esquipulas, 10 km SE of Managua, 12.0667, -86.2167, 13 Jun 1977, *Neill 2122* (MO); campus of the University Centroamericana, Managua, 12.1167, -86.2667, 125 m, 7 Sep 1981, *Stevens 20625* (MO).

**2. *Lycianthes amatitlanensis***

**Belize. Toledo**: southern Maya Mountains, Bladen Nature Reserve, N of “AC Camp,” ca. 2 airline km N of upper Bladen Branch, 16.5042, -88.9186, 260 m, 11 May 1996, *Davidse 35822* (MO).

**Costa Rica. Alajuela**: Reserva Biológica Monteverde, Río Peñas Blancas, Finca Wilson Salazar, 10.3000, -84.7167, 800–900 m, 20 Aug 1987, *Haber 7400* (MO); Peñas Blancas river valley NE of San Carlos, 10.3667, -84.5500, 350 m, 29 Jun 1985, *Hammel 14066* (NY, TEX); **Guanacaste**: Tilarán, Río Chiquito de Quebrada Grande, Zona Monteverde, 10.4167, -84.8833, 700–900 m, 6 Jun 1987, *Haber 7432* (MO); **Limón**: along the road between BriBri and Bratsi and along the Río Sixaola, 9.5833, -82.8833, 10–50 m, 12 Feb 1977, *Gentry 3739* (NY); same location and date, *Gentry 3743* (MO); Cordillera de Talamanca, along Quebrada Cañabral, from Río Barbilla to ca. 1.5 km upstream, 10.0333, -83.4083, 100–200 m, 8 Sep 1988, *Grayum 8877* (MO); **Puntarenas**: Reserva Forestal Golfo Dulce Aguabuena. sector oeste, 8.7056, -83.5250, 50–150 m, 22 Nov 1991, *Aguilar 698* (MO); Cantón de Oso, Rancho Quemado, Rincón, Río Riyito, 50 m aguas abajo del puente, 8.6875, -83.5639, 150 m, 15 Jul 1990, *Herrera 4267* (MO); Cantón de Osa Rincón, Aguabuena, fila divisoira entre Quebrada Orito y Quebrada Baneguitas, 8.6944, -83.5333, 450 m, 26 Oct 1990, *Herrera 4513* (MO); Cantón de Golfito Jiménez, Dos Brazos de Río Tigre, siguiendo el sendero que desciende hasta la unión de la Quebrada Patemazo con Quebrada Porsillego, 8.5139, -83.4500, 500 m, 22 Nov 1990, *Herrera 4623* (MO); Cantón de Osa Filas Estero Guerra. Sierpe, 8.5750, -83.5750, 300 m, 27 Sept 1991, *Marín 198* (MO); **San José**: Zona Protectora La Cangreja, along Quebrada Grande and on adjacent ridges, ca. 2 km NNE of Mastatal de Puriscal, 9.7000, -84.3667, 400–540 m, 23 Jul 1988, *Grayum 8637* (MO, TEX); Z.P. La Cangreja, Santa Rosa de Puriscal, en las faldas de Fila La Cangreja, 9.7194, -84.3889, 500 m, 10 Sep 1992, *Morales 636* (MO).

**Guatemala. Alta Verapaz**: [previously] Dept. Amatitlán, Barranca de Eminencia, 1400 ft, Feb 1892, *Donnell-Smith 1457* (US); Sacté, 15.5000, -90.4500, 900–1050 m, 4 Apr 1975, *Kunkel 186* (BR); same location, 27 Nov 1974, *Kunkel 211* (BR); same location, 4 Apr 1975, *Kunkel 398* (BR); Cubilquitz [Cubilhuitz], [15.6675, -90.4293], 350 m, Jul 1907, *von Tuerkheim 153* (US); same location, Jul 1901, *von Tuerkheim 7753* (US); **Chimaltenango**: lower and middle southwestern slopes of Volcán Fuego, above Finca Montevideo, along Barranco Espinazo and tributary of Río Pantaleón, [14.4679, -90.9792], 1200–1600 m, 20 Sep 1942, *Steyermark 52098* (G); **Escuintla**: Río Guacalate, [14.3643, -90.8127], 600 m, 16 Dec 1938, *Standley 60200* (US); **Huehuetenango**: between Ixcan and Finca San Rafael, Sierra de los Cuchumatanes, [15.3937, -91.6425], 200–800 m, 24 Jul 1942, *Steyermark 49396* (NY); **Izabal**: Montañas del Mico, 7–8 km W of Santo Tomás de Castilla on road to microwave tower, [15.6719, -88.6929], 600–650 m, 19 Aug 1988, *Stevens 25592* (MO); Cerro San Gil, [15.6333, -88.8167], 803 m, 8 Feb 2012, *Véliz 23523* (BIGU); **Petén**: Cadenas Road, west of km 142, [16.0682, -89.3573], Nov 1966, *Contreras 6519* (LL); La Cumbre, on Cadenas Road, km 143, [16.0791, -89.3454], 30 Jul 1969, *Contreras 8801* (LL); La Cumbre, in zapotal, on Parcela de José León, 3 km E, [16.0794, -89.3243], 14 Aug 1976, *Lundell 20140* (LL); Yaltutú, entre Cristo Rey y Poptún, [16.56, -89.4727], 433 m, 11 Nov 1965, *Molina-R. 15565* (NY).

**Honduras. Atlántida**: Lancetilla Valley, near Tela, [15.7657, -87.4569], 20–600 m, 6 Dec 1927 to 20 Mar 1928, *Standley 52626* (US); same location and date, *Standley 53113* (US); same location and date, *Standley 54181* (US); near bank of Tela River, above Lancetilla, [15.7391, -87.4844], 100 ft, 17 Jul 1934, *Yuncker 4625* (MO, NY); **Olancho**: on road from San Francisco de La Paz to Gualaco, 11.5 km S of Gualaco, 14.9500, -86.1333, 1000 m, 29 May 1992, *D’Arcy 18034* (MO).

**Mexico. Chiapas**: Mpio. Palenque, 6–12 km S of Palenque on the road to Ocosingo, [17.4460, -91.9642], 300 m, 12 Oct 1972, *Breedlove 28830* (MO); Mpio. Palenque, 9–12 km S of Palenque on road to Ocosingo, [17.4398, -91.9440], 300 m, 11 Nov 1981, *Breedlove 55371* (MO, LL); Mpio. La Trinitaria, 15 km E NE of Dos Lagos above Santa Elena, [16.1343, -91.5572], 1000 m, 29 Dec 1981, *Breedlove 56618* (MO, LL); 19 km S of Palenque, Cascada Mizola [Misol Ha], 17.3909, -91.9993, 250 m, 2 Oct 1974, *Madison 1763* (GH); Mpio. Ococingo: a 4 km al S de ejido Benemérito de las Américas, camino a Flor de Cacao, [16.4779, -90.6441], 120 m, 18 Feb 1985, *Martínez 10836* (NY); Chiapas, 1913, *Purpus 6994* (MO, NY); Mpio. Villaflores, Ejido Tres Picos, 16.2272, -93.5808, 1780 m, 19 Apr 2002, *Reyes-García 4437* (MEXU); **Tabasco**: Vicinity of Teapa, along road between Teapa and Tacotalpa, 3.1 mi E of Teapa, along stream and limestone cliffs ca 1/4 mi S of Hwy, 17.5500, -92.9833, 150 m, 19 Feb 1987, *Croat 65349* (MO); **Veracruz**: Mpio. Jesús Carranza, Lomas al S de Pob. 2 (ca. 3 km al S de entronque de terracería La Laguna-Sarabia con Camino al N a Pob. 2), 17.2000, -94.6500, 150 m, 8 Jul 1988, *Wendt 6064* (MO).

**Nicaragua. Chontales**: Cerro Oluma, near top of Cordillera Amerisque, 12.3000, -85.4000, 840 m, 3 Jan 1984, *Gentry 43890* (MO); **Matagalpa**: falda N del Cerro Musún, frente a trocha a Wanawás, [13.0100, -85.2319], 200–500 m, 16 May 1980, *Araquistain 2753* (MO); **Zelaya**: Cerro Saslaya, [13.7592, -85.0343], 1100 m, 3 May 1978, *Neill 3836* (MO).

**Panama. Bocas Del Toro**: Rivera del Río Changuinola en la Comunidad de Guayabal en la Quebrada Guararí, [9.3064, -82.5448], 144 m, 16 May 2007, *Daguerre 9140-ND* (US); **Coclé**: 2 miles N of Cerro Pilon, [8.6412, -80.1321], 900 m, 16 Mar 1973, *Liesner 733* (NY, US); **Comarca de San Blas**: Nasagandi El Llano-Carti road, along a creek on the Atlantic slope, [9.3167, -78.2500], 300 m, 10 Aug 1984, *de Nevers 3658* (G, NY, TEX); Nasagandi El Llano-Carti road, 18 km from Interamerican Hwy, headwaters of Atlantic-draining creeks, 9.3167, -78.9167, 300 m, 7 Sep 1984, *de Nevers 3865* (G); **Darién**: Río Uruceca, [8.1104, -77.7003], Nov 1967, *Bristan 1438* (US); Parque Nacional de Darién. ridge between Río Topalisa and Río Pucuro ca. 17 km E of Pucuro, Mi Casita to La Laguna, 8.0667, -77.2833, 600–850 m, 15 Oct 1987, *de Nevers 8339* (MO); bank above Río Paca, [8.1104, -77.5892], 26 Jun 1959, *Stern 599* (US); **San Blas**: forest SE of Puerto Obaldia, [8.6667, -77.4198], 18 Aug 1971, *Croat 16766* (MO); Pemasky, sendero lateral a Nergan Igar, km 15 de la carretera Llano-Cartí, 9.0333, -78.9667, 350 m, 2 Jul 1994, *Galdames 1274* (NY); **Veraguas**: Parque Nacional Cerro Hoya, Restingue, 7.2454, -80.8719, 729 m, 19 Jul 2011, *Flores 647 RF* (MO).

**3. *Lycianthes anomala***

**Mexico. Oaxaca**: Mpio. San Lucas Ojitlan, Loma de Naranjo, 21 Jul 1989, *Calzada 14874* (MEXU); Mpio. San Felipe Usila, a 2 km del poblado, Cerro Verde, carretera al campamento Arroyo Tambor, [17.9469, -96.4971], 550 m, 1 Nov 1990, *Calzada 16578* (MEXU); Mpio. Santa María Tlalixtac, orillas del Río Cóndor, brecha entre Santa María Tlalixtac y Chiquihuitlán de Benito Juárez, [17.9540, -96.7179], 675 m, 25 Nov 2004, *Juárez-García 877* (MEXU); Mpio. Teotitlan de Flores Magon, San Bartolome Ayautla, [17.0289, -96.5193], 1300 m, 10 Jun 1973, *Luis-Diaz 11* (MEXU); Mpio. Sta. María Chilchotla, 6.3 km del Puente de Fierro por la terracería Sta. María Chilchotla, 18.1939, -96.8453, 1226 m, 4 Jul 2001, *Munn-Estrada 1315* (MEXU, XAL); Mpio. Sta. María Chilchotla, aprox. 6.3 km del Puente de Fierro por la terracería a Sta. María Chilchotla, Sierra Mazateca, 18.1939, -96.8453, 1226 m, 6 Mar 2002, *Munn-Estrada 2135* (XAL); Dto.Tuxtepec, Mpio. Santa María Jacatepec, predio La Joya del Obispo, 17.8599, -96.2060, 12 Aug 1990, *Ramos 425* (MEXU, XAL); Dto. Teotitlan, Mpio. San Bartolome Ayautla, 2 km al E de Campo Aereo la Carlota, carretera Huautla-Jalapa de Díaz, [18.0422, -96.6910], 1300 m, 20 Jul 1982, *Torres-C. 825* (MEXU); **Veracruz**: Atoyac, Ejido La Esperanza, 18.8667, -96.7333, 750 m, 21 Jun 1985, *Acevedo-R. 272* (MEXU, XAL); Mpio. Atoyac, Vara Negra, 3 km al nor-noroeste de Atoyac, 18.9167, -96.7667, 700 m, 17 Jul 1985, *Acevedo-R. 304* (XAL); Mpio. Atoyac, 3.5 km al S de Miraflores, 18.9167, -96.8000, 700 m, 19 Sep 1985, *Acevedo-R. 486* (IEB, XAL); Mpio. Atoyac, Miraflores, 5 km al NW del poblado de Atoyac, 18.9333, -96.8000, 850 m, 20 Sep 1985, *Acevedo-R. 536* (XAL); Mpio. Huatusco, Barranca al W de Capulapa, 19.1000, -96.9000, 800 m, 8 Nov 1979, *Avendaño-R. 572* (XAL); Valle de Cordova, 14 Jan 1865, *Bourgeau 1753* (US); Mpio. Coetzala, 3 km WNW of Cuichapa on the road to Coetzala, 1.5 km SE of Coetzala, 18.7833, -96.9000, 550–700 m, 3 Jul 1982, *Diggs 2731* (BR, MEXU, MO, XAL); Rancho Ojo de Agua, Cordoba, [18.9656, -96.9358], 31 Oct 1948, *Miranda-González 4831* (MEXU); Mpio. Amatlán de Los Reyes, Camino Peñuela Amatlán (Ejido Peñuela), 7 Aug 1985, *Oseguera-C. 44* (XAL); Mpio. Coetzala, Ladera del cerro al E de Coetzala, 18.7806, -96.9144 650 m, 11 Nov 2001, *Rincón-Gutiérrez 2811* (IEB, MEXU, XAL); Mpio. Tezonapa, a 4 km aproximadamente al suroeste de Motzorongo, 18.6667, -96.6667, 600 m, 9 Feb 1986, *Robles-G. 185* (MEXU, XAL); Mpio. Tezonapa, a 3 km aproximadamente al suroeste de Motzorongo, 18.6667, -96.6667, 450 m, 9 Feb 1986, *Robles-G. 226* (MEXU, XAL); Mpio. Tezonapa, a 4 km aproximadamente al suroeste de Motzorongo, 18.6667, -96.6667, 600 m, 10 Feb 1986, *Robles-G. 256* (XAL); Mpio. Tezonapa, a 3 km aproximadamente al suroeste de Motzorongo, 18.6667, -96.6667, 450 m, 10 Jun 1986, *Robles-G. 763* (MEXU, XAL); Mpio. Tezonapa, a 4 km al suroeste de Vazquez Vela, 850 m, 20 Nov 2019, *Robles.-G. 1080* (XAL).

**4. *Lycianthes armentalis***

**Belize. Belize**: Prospecto, Northern River, [17.862704, -88.347653], 30 Nov 1933, *Gentle 905* (MO, NY); **Cayo**: Cayo, [17.098525, -88.941742], Jun–Aug 1936, *Lundell 6126* (A, NY); Mountain Pine Ridge, San Agustin, [16.966438, -88.983291], July 1936, *Lundell 6814* (MEXU, NY); Xunantonich, [17.09, -89.1416], 29 Aug 1980, *Whitefoord 2262* (MO); **Orange Walk**: Honey Camp, [18.033187, -88.433268], Oct 1929, *Lundell 548* (MO).

**Guatemala. Petén**: Dos Lagunas, on Ixcanrio road, about 3 km NE of the village, 9 Nov 1960, *Contreras 1611* (TEX); Mpio. Tikal, 19 Feb 1959, *Lundell 15656* (MO, LL); Aguada Terminos, 13 Jan 1962, *Lundell 17064* (LL); Tikal National Park, Remate Road, 15 Jan 1962, *Lundell 17111* (LL); Tikal, Parque Nacional, km 58, [17.2249, -89.6176], 16 Dec 1969, *Ortíz 466* (NY); Santa Elena, en orilla del camino para El Remate, a km 19, lado noroeste del camino, 20 Jan 1972, *Ortíz 2200* (G, MO); Mpio. Melchor de Mencos, Petén, sitio arqueológico El Naranjo, 17.2453, -98.6718, 297 m, 18 Jun 2009, *Velásquez 413* (BIGU); Mpio. San José, Petén, NW-Ufer des Lago Petén Itzá, 1–1.5 km NNE-NE San José, 16.9917, -89.8833, 120–140 m, 30 Nov 1994, *Wallnöfer 9577* (MO); NW-Umgebung des Lago Petén Itzá, zwischen San José und dem Anwesen von R.O. Frisch am Chakmamantok-Felsen, da ist 0.3–0.5 km NNE Zentrum von San José, 16.9864, -89.9000, 140–160 m, 2 Dec 1994, *Wallnöfer 13612* (W).

**Mexico. Campeche**: Mpio. Champotón, a 120 km al SO de Xpujil en los alrededores de la zona arqueológica de Calakmul, 18.1000, -89.8333, 150 m, 9 Jul 1996, *Pascual-Alvarado 357* (MEXU); Mpio. Hopelchén, en Ejido Dos Lagunas, a 45 km al N de Xpujil, sobre el camino a Dzibalcén, 18.8830, -89.3500, 250 m, 30 Jul 1996, *Pascual-Alvarado 451* (MEXU); Mpio. Champotón, a 120 km al suroeste de Xpujil, sobre el camino a la zona arqueológica de Calakmul, 18.1000, -89.7833, 11 Sep 1996, *Pascual-Alvarado 569* (MEXU); Mpio. Calakmul, a 1 km al N del Poblado Narciso Mendoza, 18.2472, -89.4625, 240 m, 26 Jun 1997, *Alvarez-M. 112* (BIGU, MEXU); Mpio. Calakmul, a 10 km al S del poblado Ley de Fomento Agropecuerio, camino a Dos Naciones, 18.0000, -89.4117, 210 m, 14 Oct 1997, *Alvarez-M. 411* (MEXU); Mpio. Calakmul, a 500 m al N del Ejido 11 de Mayo, 18.0978, -89.4617, 225 m, 15 Oct 1997, *Alvarez-M. 437* (MEXU); Mpio. Calakmul, a 2.1 km al N del poblado Zoh-Laguna, 18.6136, -89.4133, 272 m, 28 Jun 2002, *Alvarez-M. 1542* (MEXU); Mpio. Calakmul, a 1 km al N del poblado La Lucha, 18.4308, -89.4348, 304 m, 1 Jul 2002, *Alvarez-M. 1637* (MEXU); Mpio. Calakmul, a 7.6 km al E de La Mancolona (Unión 20 de Junio), 18.8153, -89.2300, 160 m, 19 Aug 2002, *Alvarez-M. 1861* (MEXU); Mpio. Calakmul, a 1.1 km al NEE de Conhuás, camino a la Zona Arqueológica Nedzcaan, 18.5431, -89.9125, 193 m, 20 Aug 2002, *Alvarez-M. 1869* (MEXU); Mpio. Calakmul, a 0.9 km al E de Conhuás, carretera Xpujil-Escárcega, camino a Nadzcaan, 18.5408, -89.9144, 178 m, 4 Oct 2002, *Alvarez-M. 2147* (MEXU); Mpio. Calakmul, a 6 km al E del poblado “Unión 20 de Junio” (antes La Mancolona), camino a Flores Magón, 18.8139, -89.2344, 198 m, 24 Oct 2002, *Alvarez-M. 2229* (MEXU); Mpio. Calakmul, a 5.2 km al NE de Conhuás, camino a la Zona Arqueológica Nadzcaan, 18.5803, -89.8972, 201 m, 8 Jan 2003, *Alvarez-M. 3117* (MEXU); Mpio. Calakmul, a 8 km al S de Ejido Ley de Fomento Agropecuario, camino a Dos Naciones, 18.0197, -89.4211, 294 m, 30 Jan 2003, *Alvarez-M. 3381* (MEXU); Mpio. Calakmul, a 12 km al N del poblado Zoh-Laguna, 18.7197, -89.4019, 283 m, 3 Jun 2003, *Alvarez-M. 5197* (MEXU); Mpio. Calakmul, a 2.84 km al sureste de la comunidad El Chichonal, 18.5158, -89.5283, 294 m, 29 Sep 2003, *Alvarez-M. 6545* (MEXU); Mpio. Calakmul, a 6.82 km al NE de Conhuas, camino a Zona Arqueológica Nadzcaan, 18.5931, -89.8889, 172 m, 2 Dec 2003, *Alvarez-M. 7367* (MEXU); Mpio. Hopelchén, a 32 km al SE de Chan-chen, 19.1883, -89.2406, 113 m, 13 Jun 2004, *Alvarez-M. 9022* (MEXU); Mpio. Hopelchén, a 1.7 km al ENE de X-mejia, camino a Ukum, 19.2347, -89.3586, 152 m, 15 Jun 2004, *Alvarez-M. 9208* (MEXU); Mpio. Hopelchén, a 2.3 km al S de Chan-chen, camino a Pachuitz, 19.1875, -89.2617, 136 m, 16 Jun 2004, *Alvarez-M. 9231* (MEXU); Mpio. Hopelchén, a 9.66 km al N de Bel-ha, 19.0103, -89.2894, 161 m, 3 Aug 2004, *Alvarez-M. 10165* (MEXU); near ruins of Becán and Chicaná, 144 km E of Escárcega, [18.5100, -89.4900], 100 m, 12 Dec 1981, *Breedlove 56055* (MO); 3 km al S de Constitución, a 70 km al E de Escárcega, [18.5823, -90.1315], 10 Jul 1983, *Cabrera-C. 5046* (MEXU); a 12 km al E de Conhuas, sobre la carretera Chetumal-Francisco Escárcega, [18.5190, -89.8138], 1 Aug 1984, *Cabrera-C. 6994* (MEXU); 10 km al S de Conhuas, sobre el camino al Centro Ceremonial de Calakmul, entrando por el km 98 de la carretera Escárcega-Chetumal, [18.4503, -89.8892], 27 Jul 1986, *Cabrera-C. 11853* (BIGU, MEXU); Mpio. Calakmul, a 0.9 km al NE de Conhuás, 18.5411, -91.9139, 210 m, 20 Feb 2002, *Calónico 21786* (DAV); Mpio. Champotón, camino entre Pusyunich y San Antonio Yacasay, 19.1833, -90.4500, 10 m, 24 Jul 1981, *Chan 740* (XAL); Mpio. Hopelchén, camino blanco entre Kankabchén y Chunchintok, 19.3292, -89.6111, 30 m, 25 Sep 1984, *Chan 4002* (MEXU); Mpio. Hopelchén, camino entre Dzibalchén y Vicente Guerrero, 19.5139, -89.6611, 30 m, 17 Sep 1985, *Chan 5650* (MEXU); campo experimental forestal tropical El Tormento, km 5 carretera Escárcega a Candelaria, [18.6129, -90.7967], 4 Jan 1966, *Chavelas-P. ES2586* (MEXU); Mpio. Champotón, km 30 del entronque hacia Calakmul, 18.3181, -89.8500, 2 Dec 1996, *Durán 2847* (GH, NY); Mpio. Champotón, 9 km entre Conhuas y Calakmul, 18.5283, -89.9244, 150 m, 1 Aug 1995, *Gutiérrez-B. 4528* (XAL); Mpio. Champotón, Calakmul, 18.1419, -89.8047, 240 m, 08 Aug 1995, *Gutiérrez-B. 4548* (MEXU, XAL); Reserva de la Biosfera de Calakmul, a 35 km de la entrada a la reserva, (depresión producida por extracción de tierra para la contrucción de la carretera), 18.2631, -89.8275, 160 m, 18 Oct 1997, *Lira-C. 266* (MEXU); Mpio. Calakmul, a 9 km al S de La Nueva Vida, por la carretera Xpujil-Campeche, entrada en el km 25 de esta, 18.7342, -89.3947, 230 m, 22 Nov 1997, *Lira-C. 357* (MEXU); Mpio. Calakmul, a 17 km al S de la Nueva Vida, entrada en el km 16 de la carretera Xpujil-Campeche, 18.6533, -89.4083, 230 m, 23 Nov 1997, *Lira-C. 416* (MEXU); Mpio. Calakmul, a 20 km al S de la caseta de vigilancia de la Reserva de la Biosfera de Calakmul, antigua caseta de vigilancia, 18.3642, -89.8922, 204 m, 24 Nov 1997, *Lira-C. 449* (MEXU); Tuxpeña, [18.4500, -90.0800], 22 Nov 1931, *Lundell 971* (A, G, GH, MO, NY); Mpio. Calakmul, a 6 km al S de la entrada a Calakmul, 18.4442, -89.8886, 120 m, 17 Oct 1997, *Madrid 205* (MEXU); Mpio. Calakmul, a 34 km al S de la caseta de entrada a Calakmul, 18.2631, -89.8275, 160 m, 18 Oct 1997, *Madrid 278* (MEXU); Mpio. Champotón, a 55 km al S del km 98 de la carretera Escársega a Chetumal, sobre el camino a las ruinas de Calakmul, [18.1483, -89.7808], 200 m, 4 Feb 1983, *Martínez-S. 2950* (BIGU, MEXU, MO); Mpio. Calakmul, a 8 km al N de Zon-Laguna, camino a La Nueva Vida, 18.6567, -89.4083, 217 m, 13 May 1997, *Martínez-S. 27186* (M, MEXU); Mpio. Hopelchén, a 1 km al S de Xcan-ha, camino a Xpujil, 19.0944, -89.3317, 80 m, 29 Jun 1997, *Martínez-S. S. 27467* (BIGU, MEXU, NY); Mpio. Calakmul, a 1 km al W del Poblado Unión 20 de Junio (Mancolona), camino a La Nueva Vida, [18.8106, -89.2983], 200 m, 2 Jul 1997, *Martínez-S. 27521* (MEXU); Mpio. Calakmul, a 8 km al N de Calakmul, 18.1464, -89.7833, 199 m, 13 Jul 1997, *Martínez-S. 27719* (MEXU, NY); Mpio. Calakmul, a 10 km al E de Dos Naciones, camino a El Civalito, 17.9128, -89.3153, 147 m, 29 Jul 1997, *Martínez-S. 27869* (MEXU); Mpio. Calakmul, a 4 km al S de La Nueva Vida, camino a Xpujil, en el puente Papagayo, [18.7375, -89.3922], 297 m, 2 Aug 1997, *Martínez-S. 27973* (MEXU); Mpio. Calakmul, a 32 km al S de la Caseta de entrada a Calakmul, 18.2897, -89.8394, 250 m, 9 Aug 1997, *Martínez-S. 28270* (MEXU, NY); Calakmul, a 2 km al N de Nuevo Campanario, camino a Xpujil, 18.0995, -89.8454, 11 Aug 1997, *Martínez-S. 28348* (MEXU, NY); Mpio. Calakmul, en Puente el Papagayo, a 10 km al S de La Nueva Vida, 18.7353, -89.3947, 240 m, 12 Oct 1997, *Martínez-S. 28764* (MEXU, TEX); Mpio. Calakmul, a 34 km al S de la caseta de entrada a Calakmul, 18.2631, -89.8275, 160 m, 16 Oct 1997, *Martínez-S. 28957* (MEXU, TEX); Mpio. Calakmul, a 2 km al W de La Mancolona, camino a La Nueva Vida, 18.8100, -89.3139, 230 m, 25 Oct 1997, *Martínez-S. 29352* (TEX); Mpio. Calakmul, a 1 km al SE de Eugenio Echeverria Castellot II antes (Carrizal), 18.3972, -89.4389, 220 m, 28 Oct 1997, *Martínez-S. 29540a* (MEXU); Mpio. Calakmul, a 2 km al E de Plan de Ayala (5 de Mayo), 18.0569, -89.2867, 485 m, 6 Dec 1998, *Martínez-S. 31555a* (MEXU); Mpio. Calakmul, a 2 km al oeste de La Mancoloma, camino a la Nueva Vida, 18.8114, -89.3044, 231 m, 7 Dec 1998, *Martínez-S. 31631* (MEXU, TEX); Mpio. Calakmul, en el km 34 del camino a Calakmul, 18.2625, -89.8278, 225 m, 9 Dec 1998, *Martínez-S. 31663* (MEXU); Mpio. Calakmul, Nadzcaan a 10. 5 km al NE de Conhuás, 18.6058, -89.8511, 215 m, 17 Aug 2002, *Martínez-S. 35885* (MEXU); Mpio. Hopelchén, a 7.3 km al SO de Othón P. Blanco, 18.3609, -89.1569, 92 m, 13 Jun 2005, *Martínez-S. 37677* (MEXU); Mpio. Hopelchén, a 2 km al E de X-Mejía, 19.2347, -89.3592 150 m, 24 Jun 2005, *Martínez-S. 38011* (MEXU); Mpio. Hopelchén, no exact location, 18.5500, -89.4333, 30 m, 9 Jul 1988, *Ortega-T. 586* (XAL); Mpio. Hopelchén, no exact location, 18.5500, -89.4333, 30 m, 9 Jul 1988, *Ortega-T. 576* (XAL); Mpio. Champotón, Yohaltun rumbo a Chaechaito, 18.9500, -90.3333, 8 m, 16 Oct 1981, *Ucan 1617* (XAL); Mpio. Champotón, Rancho Constitución, carretera Escárcega – Chetumal, 100 m, 9 Aug 1984, *Vazquez-B. 1899* (XAL); **Quintana Roo**: Mpio. José María Morelos, Ejido Othón P. Blanco, a 5.6 km al S del poblado, 19.5717, -89.0106, 03 Sep 2004, *Alvarez-M. M. 10533* (MEXU); Mpio. José María Morelos, a 5.5 km al SSE del poblado Pozo, [19.7627, -88.7237], 9 Sep 2004, *Alvarez-M. 10623* (MEXU); Mpio. José María Morelos, a 5.5 km al S de pozo Pirata camino a Zafarrancho, 19.5681, -88.8736, 102 m, 17 Jun 2005, *Alvarez-M. 11243* (MEXU); en la desviación a Laguna Ocum, a 10 km al S de F. Carrillo Puerto, [19.4911, -88.0334], 13 Nov 1980, *Cabrera-C. 279* (MEXU); a 3 km al oeste de F. Carrillo Puerto, rumbo a Vigía Chico, [19.5990, -88.0062], 16 Nov 1980, *Cabrera-C. 412* (MEXU); en la brecha que une Divorciados con la Pantera, [19.0885, -88.4744], 13 Dec 1980, *Cabrera-C. 733* (MEXU); Mpio. Felipe Carrillo Puerto, a 12 km al E de Carrillo Puerto, rumbo a Vigía chico, [19.6362, -87.9306], 14 Dec 1980, *Cabrera-C. 785* (MEXU); sobre brecha a Chanca Ver, a 9 km al S de Carrillo Puerto, [19.4840, -88.0283], 19 Jan 1981, *Cabrera-C. 1075* (MEXU); a 20 km a S de Carrillo Puerto, [19.3921, -88.0570], 17 Mar 1981, *Cabrera-C. 1568* (MEXU); a 20 km al E de Carillo Puerto, rumbo a Vigía Chico, [19.6779, -87.8685], 20 Sep 1982, *Cabrera-C. 3571* (MEXU, MO); a 7 km al N de Bacalar, [18.7440, -88.3567], 21 Dec 1982, *Cabrera-C. 4135* (MEXU); Mpio. Felipe Carrillo Puerto, a 4 km al S de Kampocolche, [19.8211, -88.3440], 26 Jul 1982, *Cabrera-C. 4284* (MEXU); a 12 km al S de San José de la Montaña, sobre el camino a Tomás Garrido, [18.2667, -89.0351], 13 Jul 1983, *Cabrera-C. 5160* (MEXU, MO, NY); a 2 km al oeste de Chanca Veracruz a 8 km al S de Felipe Carrillo Puerto, [19.4973, -88.0155], 21 Aug 1983, *Cabrera-C. 5459* (MEXU, MO, NY); 10 km al noroeste de Estero Franco, entrando por Rancho El Danto, sobre la carretera Ucum – La Unión, [17.9926, -88.8480], 29 Mar 1988, *Cabrera-C. 16070* (MEXU, MO); Mpio. Felipe Carrillo Puerto, 5–8 km al E de Felipe Carrillo Puerto sobre el camino a Vigía Chico, [19.6115, -87.9760], 19 Jan 1989, *Cabrera-C. 16133* (MEXU); Felipe Carrillo Puerto, 10 km al noroeste de Carrillo Puerto, sobre la carretera a Valladolid, [19.7324, -88.0909], 16 Feb 1989, *Cabrera-C. 16312* (MEXU); Felipe Carrillo Puerto, Las Palmas aprox. 36 km al S de F. Carrillo Puerto, [19.2559, -88.0992], 16 Sep 1989, *Cabrera-C. 16557* (MEXU); Othon P. Blanco, en brecha a 8 km al NO de estero Franco sobre carr. Ucum – La Unión, [17.9876, -88.8574], 19 Sep 1989, *Cabrera-C. 16646* (MEXU); Mpio. Las Palmas, a 36 km aprox. al S de F. Carrillo Puerto, [19.2546, -88.0994], 23 Oct 1989, *Cabrera-C. 16766* (MEXU); sobre el camino a Vigia Chico 10 km al NO de F. Carrillo Puerto, [19.6281, -87.9451], 24 Oct 1989, *Cabrera-C. 16784* (MEXU); Mpio. Chetumal, km 20 Carretera Chetumal-Escárcega, 18.5333, -88.4333, 20 Jul 1983, *Chan 2545* (XAL); E side of Lake Chicnancanab between Presumida and San Francisco, [19.8031, -88.8067], 1 Oct 1982, *Darwin 2391* (MEXU, MO); Mpio. Felipe Carrillo Puerto, Reserva Sian Kaan, 1 km al sureste del crucero de Chumpón, [19.9529, -87.7661], 23 Oct 1983, *Duran 586* (MEXU); Mpio. Felipe Carrillo Puerto, a 2 km al SE del Crucero Chumpon, [19.9484, -87.7720], 10 Nov 1983, *Duran 622* (MEXU); Mpio. José María Morelos, a 1.7 km al E de Gavilanes, 19.4922, -88.7381, 81 m, 14 Jun 2005, *Martínez-S. 37771* (MEXU); Mpio. José María Morelos, a 2 km al S de Tigre Grande, [19.6944, -89.0422], 90 m, 16 Jun 2005, *Martínez-S. 37789* (MEXU); Mpio. José María Morelos, a 1.7 km al E de San Isidro Poniente, 19.3253, -88.8731, 145 m, 18 Jun 2005, *Martínez-S. 37888* (MEXU); Mpio. José María Morelos, camino a Zafarrancho, 0.73 km al N de Zafarrancho, 19.5200, -88.8864, 91 m, 22 Aug 2005, *Martínez-S. 38092* (MEXU); Mpio. Chetumal, roadcuts through primary (and some secondary) forest on gentle limestone hills, with chicle tree, 1.5 km south of main road from Chetumal to Escárcega (hwy 186) 1 km E of La Moza, [18.4639, -89.1822], 28 Aug 1997, *Nee 47167* (MEXU); camino a Vallarta, [20.8667, -87.0167], 16 Oct 1985, *Olmsted 50* (MEXU); Mpio. Felipe Carrillo Puerto, 3 km al N de Yactun, [19.7684, -87.6818], 21 Aug 1971, *Pérez-S. 397* (IEB, MEXU); Mpio. Felipe Carrillo Puerto, Rancho Las Palmas km 33 carretera F. Carrillo Puerto a Chetumal, km 2 de Palmas a Vallarta, [19.2607, -88.0981], 14 Nov 1990, *Salazar-C. 40* (MEXU); Mpio. Chetumal, 6.5–7 km N of Tomás Garrido on the road which joins Hwy 186 west of Nicolas Bravo, 18.1000, -89.0500, 150 m, 15 Mar 1990, *Sanders 9983* (NY); a 7 km al S de Tulum, [20.1800, -87.4700], 9 Jan 1980, *Téllez-V. 1175* (MO); a 6 km al W de Cancún, sobre la carretera a Mérida, [21.1400, -86.8700], 2 Feb 1980, *Téllez-V. 1322* (NY); a 15 km al S de F. Carrillo Puerto, [19.4350, -88.0410], 6 May 1980, *Téllez-V. 2078* (MEXU, MO, NY); a 30 km al S del Ejido Laguna OM, [18.2059, -89.0213], 8 Jun 1980, *Téllez-V. 2432* (MEXU, MO); a 2 km al S de Ehido Laguna OM, sobre camino a Tomás Garrido, [18.4386, -89.0034], 6 Jul 1980, *Téllez-V. 2666* (MEXU, MO, NY); a 1 km al E de F. Carrillo Puerto, sobre camino a Vigía Chico, [19.5889, -88.0221], 10 Jul 1980, *Téllez-V. 2817* (MEXU, MO); a 17 km al S de San José, rumbo a Tomás Garrido, [18.2232, -89.0373], 5 Sep 1980, *Téllez-V. 3415* (MEXU, MO); a 3 km al N de Xel-Ha, [20.3474, -87.3488], 11 Sep 1980, *Téllez-V. 3539* (MEXU, MO); a 2 km al E de F. Carrillo Puerto, hacia Vigía Chico, [19.5939, -88.0141], 15 Oct 1980, *Téllez-V. 3718* (MEXU); **Yucatán**: a 24 km del Mpio. Temozón, en las cercanias del Ejido de Actuncoh, [20.9167, -88.1119], 18 Jun 1986, *Aguilar-Zepeda 249* (MEXU); Tizimin-tixkankal, [21.1480, -88.1122], 12 Oct 1955, *Enriquez 180* (MEXU); Yucatán, (No exact location given), 1917–1921, *Gaumer 24212* (A, G, MO, NY); Mpio. Tkak, carretera Noholal-Sudzal-Chico, 19.7500, -89.2500, 18 Nov 1992, *May 766* (MEXU).

**5. *Lycianthes arrazolensis***

**El Salvador. State Unknown**: E side of Cerro del Aguila, 13.9000, -89.7000, 1710 m, 28 Apr 1942, *Tucker 1316* (G, LL); **Ahuachapán**: paredones humedos a la orilla de la carretera de Ataco al Canton El Naranjito, 3 km al S de Ataco, 1200 m, 21 Jul 1993, *Linares 475* (MEXU); camino a la Laguna de las Ninfas, [13.8789, -89.7967], 1500 m, 28 May 1959, *Montalvo 3493* (MO); Apaneca, camino a la Laguna Verde, 13.8667, -89.8000, 1100 m, 17 Sep 1997, *Renderos MR-00273* (NY); Apaneca, a orilla de la carretera. 13.8667, -89.8167, 1600 m, 4 Apr 1995, *Villacorta 2267* (MO); **Morazán**: Mpio. Chilanga, Canton Joya del Matazano, A.N.P. Cerro Cacahuatique, Sendero El Cutal, 13.7622, -89.1911, 1442 m, 7 May 2014, *Rodríguez 4832* (NY); **San Salvador**: complejo Volcán de San Salvador, Cerro El Picacho, A.N.P. Santa María, calle principal hacia la cumbre, 13.7497, -89.2569, 1798 m, 13 Jun 2012, *Rodríguez 3122* (NY); **Santa Ana**: 7 mi NE of Metapán between administrative office and Los Planes, along dirt road between Metapán and Cerro Monte Cristo, [14.3800, -89.4008], 1200 m, 30 Jul 1977, *Croat 42295* (DAV, MO); entre El Olimo y la Cumbre del Cerro El Aguila, 1700–1900 m, 23 May 1993, *Linares 401* (MEXU); San José Ingenio, P. N. Montecristo, el plan del cedro, 14.4167, -89.3500, 1000 m, 23 Aug 2001, *Martínez 115* (MO); Chalchuapa, Canton El Paste, Volcán Chingo, sendero al crater, cumbre de cerro. [14.1186, -89.7242], 1724 m, 9 Jul 2012, *Rodríguez 3197* (NY); Chalchuapa, San Isidro, Volcán Chingo, vereda aledana a la Brecha que conduce a la cima del crater, 14.1192, -89.7250, 1746 m, 16 Apr 2013, *Rodríguez 4029* (NY).

**Guatemala. Alta Verapaz**: 3 km de Villa Hermosa, [14.8700, -91.5700], 1400 m, 16 May 1963, *Molina-R. 12362* (NY); **Baja Verapaz**: Mpio. San Jeronimo, Santa Elena la Cumbre, 15.0292, -90.2167, 2263 m, 12 Nov 2009, *Cóbar 1980* (BIGU); along Highway CA 14 to Cobán, about 1 mi S of turnoff to Salamá, [15.0186, -90.2236], 1300 m, 16 Jul 1977, *Croat 41144* (MO); **Chimaltenango**: Chichavac, 8000–9000 ft, 10 Jul 1933, *Skutch 379* (A); Volcán Acatenango, Aldea Quisache, 14.5181, -90.2844, 1500 m, 19 May 2004, *Véliz 15260* (MEXU); **El Progreso**: 15 km N of Morazo, [14.9750, -90.2067], 17 Jul 1970, *Harmon 3207* (MO); **Escuintla**: 2 km W of San Vicente Pacaya, 1500 m, 31 May 1970, *Harmon 2438* (MO); **Guatemala**: 12 miles NE of Guatemala City, along Hwy CA 9 to Puerto Barrios, [14.7817, -90.3019], 1100 m, 23 Jul 1977, *Croat 41911* (MO); same location and date, *Croat 41926* (MO); Guatemala, Jardín Botánico USAC, 14.6144, -90.5128, 1500 m, Mar 2011, *López s.n.* (BIGU); Zona 12, Ciudad Universitaria, 14.6347, -90.4956, 1500 m, 15 Jun 2015, *Ordoñez-Sayle 81* (BIGU); Mpio. Villa Canales, Fea. San Agustín Las Minas, 14.5264, -90.4950, 1839 m, 12 Aug 2010, *Veláquez 1460* (BIGU); **Quiché**: Nebaj, Batzchocolá, 15.5714, -91.1035, 1300 m, 1 Aug 2017, *Tribouillier 3* (DAV); **Quetzaltenango**: along road above Santa María de Jesús, 1680 m, 25 Jan 1941, *Standley 84876* (US); along road between Finca Pirineos and Patzulí, 1200–1400 m, 9 Feb 1941, *P. Standley 86812* (US); **Sacatepéquez**: On road to Zorzoya, [14.6078, -90.6500], 29 Jun 1994, *Castillo-M. 2220* (NY); 2.3 miles SW of Alotenango, on road from Antigua to Escuintla, [14.4589, -90.8133], 1300 m, 26 Jul 1977, *Croat 42006* (MO); Mpio. Alotenango, astillero municipal, 14.4619, -90.8147, 1332 m, 20 Apr 2011, *Véliz 23680* (BIGU); **Sololá**: common at river thickets near San Andres, 2000 m, 20 Sep 1971, *Molina-R. 26679* (MEXU); Patanatic, 6 km to Panajachel, [14.7634, -91.1354], 1700 m, 20 Sep 1971, *Molina-R. 26664* (MEXU); trail between village of San Pedro, via San Juan, Cristóbal Buena Vista, and northwestern slopes of Volcán Santa Clara, [14.7044, -91.2936], 1800–2300 m, 8 Jun 1942, *Steyermark 47320* (MO); **Suchitepéquez**: Finca Moca, 4800 ft, 29 Oct 1934, *Skutch 1554* (A); Volcán Santa Clara, 1.5–2 miles W of Finca El Naranjo, [14.6078, -91.3364], 1250 m, 1 Jun 1942, *Steyermark 46787* (NY).

**Honduras. Copán**: above Antigua Copán, [14.8368, -89.1402], 8 May 1970, *Cuellar 5185* (DAV, GH); **El Paraíso**: Quebrada Tapahuasca, [13.7333, -86.9167], 1300 m, 14 Aug 1964, *Molina-R. 14659* (NY, W); **Francisco Morazán**: Aldea El Hatillo, [14.1297, -87.1644], 1500 m, 3 Aug 1978, *Espinal 56* (MO, NY); Los Chorritos, 15 km NE of Tegucigalpa, [14.1758, -87.1542], 1400 m, 3 Oct 1978, *Nelson 4853* (MO, NY); El Hatillo, [14.1297, -87.1644], 1400 m, 21 Oct 1978, *Turcios 241* (MO); El Hatillo, Tegucigalpa, [14.1297, -87.1644], 1200 m, 3 Aug 1978, *Zelaya 67* (MO); Lempira, Montaña de Celaque, SE portion of massif, repressa trail between Gracias and top of Cerro Celaque, 14.5500, -88.6500, 1290–1400 m, 23 May 1991, *Davidse 34569* (NY); Montaña de Celaque, [14.5670, -88.6500], 1700 m, 4–6 Jun 1978, *Hazlett 2824* (NY); **Ocotepeque**: 10 km from Nueva Ocotepeque, road to El Portillo, [14.4167, -89.0833], 1500 m, 26 Aug 1968, *Molina-R. 22188* (NY); 6 mi NE of Ocotepeque, San Rafael Las Mataras Creek, [14.4500, -89.1333], 1300 m, 1 Sep 1975, *Molina-R. 30969* (MO);

**Mexico. Chiapas**: Mpio. Villaflores, 0.5 km al N del poblado Nuevo Independencia, 16.2144, -93.5842, 1324 m, 7 Sep 2004, *Alvarez 9922* (MEXU); Mpio. Venustiano Carranza, 3 miles S of Aguacatenango along road to Pinola Las Rosas, 5600 ft, 25 Jun 1965, *Breedlove 10574* (US); Mpio. Tenejapa. Paraje of Kotol Te’, 4300 ft, 16 Jul 1965, *Breedlove 11079* (LL); Mpio. Venust. Carranza, 3 mi S of Aguacatenango along road to Pinola Las Rosas, 5600 ft, 22 Jul 1965, *Breedlove 11195* (LL); Mpio. Jitotol, on the bank of the Río Hondo, 4 miles N of Jitotol on the road to Pueblo Nuevo Solistahuacán, 5500 ft, 20 Aug 1965, *Breedlove 12023* (US); Mpio. Jitotol, along the road to Río Hondo, 6.5 km N of Jitotol, [17.1000, -92.8600], 1700 m, 27 Oct 1971, *Breedlove 21373* (MO, LL); SE side of Volcán Tacaná above Talquian, [15.1053, -92.0925], 2200 m, 5 Mar 1972, *Breedlove 24392* (MO); Mpio. Cintalapa, SE of Cerro Baul on the border with the state of Oaxaca, 16 km NW of Rizo de Oro, along the logging road to Colonia Figaroa, [16.5661, -94.1342], 1600 m, 21 Apr 1972, *Breedlove 24755* (MO); Mpio. Villa Corzo, E base of Cerro Tres Picos near Cerro Bola along a logging road SW of Colonia Agronomos Mexicanos, [16.2106, -93.5961], 1500 m, 4 May 1972, *Breedlove 25055* (MO); same location, 1500–1800 m, 27 May 1972, *Breedlove 25349* (MO, NY); Mpio. Motozintla de Mendoza, SW side of Cerro Mozotal, 11 km NW of the junction of the road to Motozintla along the road to Porvenir and Siltepec, [15.4000, -92.3000], 2100 m, 27 Jun 1972, *Breedlove 25729* (MO); Mpio. Nuevo Solistahuacán, 3 km NW of Pueblo Nuevo Solistahuacán, near Clinica Yerba Buena, [17.1800, -92.9000], 1700 m, 11 Jul 1972, *Breedlove 26045* (MO, LL); Mpio. Totolapa, 6–8 km W of Teopisca on the side of Cerro Chenek’ultik, [16.5300, -92.5300], 2150 m, 16 Aug 1972, *Breedlove 27075* (MO); Mpio. Cintalapa, ridge SE of Cerro Baul on the border with the state of Oaxaca, 17 km NW of Rizo de Oro, along a logging road to Colonia Figaroa, [16.5600, -94.1900], 1600 m, 12 Oct 1979, *Breedlove 44412* (MEXU, MO); Mpio. Angel Albino Corzo, above Finca Cuxtepec, [15.7664, -92.9550], 1380 m, 21 Oct 1980, *Breedlove 46712* (MO); Mpio. Cintalpa, on Oaxaca-Chiapas border near La Cienega de León, [16.6800, -94.0300], 1080–1230 m, 1 Dec 1980, *Breedlove 48149* (MO); Mpio. Angel Albino Corzo, above Finca Cuxtepec, [15.7300, -92.9600], 1380 m, 24 Jun 1981, *Breedlove 51193* (MO); 1 km al suroeste del entronque a Simajovel de Allende sobre la carretera Escopetazo-Pichucalco, [17.0503, -92.8497], 28 Jun 1982, *Cabrera 3028* (MO, NY); Mpio. Mapastepec, Cañada Honda, camino para el Arenal, 1360 m, 15 May 1983, *Calzada 8984* (XAL); Mpio. Jiquipilas, cerro frente a Cerro La Palmita, [16.3727, -93.9019], 1130 m, 08 Jul 1994, *Castillo 79* (MEXU); Mpio. Oxchuc, orilla de camino 1 km antes de Rancho El Cura, 16.7819, -92.2722, 1730 m, 28 Jun 1994, *Chame 61* (MEXU); Mpio. La Concordia, 1700 m al O de Arroyo Negro-Neuva Linda, [15.8455, -93.0449], 1250 m, 28 Jul 2014, *Diaz-M. 1079* (MEXU); Mpio. Villa Corzo, Nueva Reforma Agraria, 15.9172, -93.2506, 938 m, 26 Jan 2002, *Gómez-Domínguez 45* (MO); Mpio. Mpio. La Concordia, Finca Custepec. trail NW from Finca, 1–3 km along trail [15.7330, -92.9670], 1180 m, 12 Jul 1990, *Hampshire 1217* (MEXU); Mpio. Angel Albino Corzo, Reserve El Triunflo (Campamento/HQ); Cañada Honda, 15.6500, -92.8000, 1400 m, May 1989, *Heath MA103* (MEXU); Mpio. Cintalapa, La Carraca, 17.7 km N Rizo de Oro por camino a Col. Rodolfo Figueroa, [16.5700, -94.1900], 1420 m, 23 Jun 1984, *Hernández 365* (DAV, MEXU, MO); Mpio. Villa Corzo, Reserva de la Biosfera El Triunfo, Poligono V, desvio al camino Plan de Higo, 15.8617, -93.0844, 1303 m, 9 Jul 2002, *López-Hernández 123* (MEXU); Mpio. Villa Corzo, Cerro Miramar, Plan de Ayala, 15.8569, -93.2167, 1565 m, 26 May 1999, *López-Molina 397* (NY); Mpio. Villacorzo, Reserva de la Biosfera El Triunfo, Cerro La Angostura, Polígono Zona Núcleo v La Angostura, 15.8803, -93.1958, 1147 m, 31 Mar 2005, *Martínez-Meléndez 737* (MO); Mpio. Unión Juárez, en el Volcán Tacaná, a 500 m al E de Talquián, [15.1290, -92.1051], 1700 m, 26 Apr 1987, *Martínez-S. 20309* (BIGU); Mpio. Unión Juárez, Volcán Tacaná, entre Talquián y Chiquihuite, [15.1000, -92.1000], 800 m, 23 Apr 1987, *Martínez-S. 20481* (MEXU, MO); Reserva El Triunfo, [15.6500, -92.8000], 1900 m, 1 Jun 1987, *Martínez-S. 21495* (MEXU); Siltepec, [15.5508, -92.3013], 7 Aug 1937, *Matuda 1672* (NY); Letrero, near Siltepec, [15.5100, -92.4600], 2000 m, 6 Jul 1941, *Matuda 4364* (MO, NY, LL); San Luis Siltepec, [15.4647, -92.4544], 1300 m, 20 Jun 1945, *Matuda 5975* (LL); Mpio. Arriaga, Cañada al Españadal, Rancho Bonito, 16.3328, -93.8761, 23 May 2002, *Meléndez-L. 261b* (DAV); Cerro del Boqueron, [15.2400, -92.3000], Aug 1913, *Purpus 7106* (GH, NY); Cerro del Boqueron, [15.2400, -92.3000], Jun 1914, *Purpus 7316* (G, GH, M, MO, NY); Mpio. Tonala. Ejido Las Palmas, en el paraje Santa Cruz, 16.0658, -93.5783, 180 m, 28 Apr 2002, *Reyes-García 4583* (MEXU); Mpio. Villa Flores, Ejido El Paraíso, 16.3114, -93.7069, 1500 m, 29 May 2002, *Reyes-García 4714* (DAV); Mpio. Villa Corzo, Ejido Sierra Morena, 16.1522, -93.5903, 1550 m, 30 May 2002, *Reyes-García 4788* (MEXU); Mpio. Cintalapa, “La Carraca,” 17.7 km al N de Rizo de Oro hacia Rodolfo Figueroa, [16.5700, -94.1900], 28 Jun 1984, *Torres-C. 5402* (MEXU); Mpio. Unión Juárez, Línea Divisoria, 10 km al S de Unión Juárez, [15.0400, -92.0903], 2500 m, 16 Apr 1988, *Ventura 5171* (MO, NY, TEX); Mpio. Unión Juárez, Chiquihuite, 8 km al S de Unión Juárez, [15.0700, -92.0900], 3000 m, 9 Aug 1988, *Ventura 5466* (NY); **Guerrero**: Mpio. Metlatónoc, Xatu Yahta, al W de Coicoyán, terreno de Atzompa, 17.2500, -98.3167, 2700 m, 13 Feb 1988, *de Ávila 209* (MEXU, NY); 10.9 km al E de Vallecitos, 17.9553, -101.2294, 1626 m, 22 Jul 2005, *Calonico-S. 27171* (MEXU); 36.7 km from Atoyac de Alvarez on road to Puerto de Gallo, [17.4800, -100.1900], 3050 ft, 9 Aug 1977, *Davis IV 817* (MEXU); Mpio. Atoyac de Alvarez, Las Golondrinas, carretera Paraíso-Filo de Caballos, [17.3368, -100.2302], 1600 m, 06 Jul 1985, *Espejo-Serna 1843* (MEXU); Dto. Montes de Oca, Mpio. Petatlan, El Mameyal, NW of Acapulco, I. R. F. Coyquilla, [17.4333, -99.6833], 700 m, 15 Oct 1937, *Hinton 10806* (NY); Dto. Galeana, Sierra Madre del Sur, Alcaparosa, [17.4333, -99.6833], 560 m, 23 Oct 1937, *10849* (MEXU, MO, NY, LL); Dto. Mina, Chilacayote, [17.7600, -100.6800], 1650 m, 16 Apr 1939, *Hinton 14179* (GH, MO, NY, WIS); Mpio. Leonardo Bravo, 3 km al NE de Cruz de Ocote, [17.5560, -99.8826], 2180 m, 23 Mar 1985, *Loera-H. 3473* (IEB, MEXU, TEX, XAL); Mpio. Chilpancingo de los Bravos, 6 km al W de Omiltemi, camino a la Soledad Las Hoyas, [17.5950, -99.7239], 27 Mar 1982, *Martínez-S. 271* (MEXU, MO, NY); Mpio. Tlacotepec, en el Cerro Teotepec, a 8 km al N de Puerto del Gallo, camino a Chichihualco, [17.7917, -99.9733], 3000 m, 11 Mar 1984, *Martínez-S. 6233* (IEB, MEXU, MO); 10 km al suroeste de Puerto del Gallo, sobre el camino a Atoyac, 2100 m, 13 Mar 2007, *Ramírez-Amézcua 964* (DAV, IEB, NY); Mpio. Chichihualco, 2 km al SO de La Hierbabuena, [17.6357, -99.7520], 1950 m, 22 Apr 1985, *Soto-Nuñez 8394* (MEXU); Mpio. Atoyac de Alvarez, a 15 km al NE de El Paraíso, [17.3740, -100.1400], 1100 m, 19 Aug 1985, *Soto-Nuñez 10104* (MEXU); vicinity of Omiltemi, 20 miles W of Chilpancingo on logging road, [17.5540, -99.7393], 8000 ft, 31 Jul 1957, *Straw 1084* (MEXU); Mpio Leonardo Bravo, 29 km W of Chilpancingo, [17.5897, -99.7957], 2250 m, 7 Jun 1985, *Thomas 3721* (NY); **Jalisco**: Mpio. Tecalitlán, 46 km carr. Cd. Guzmán-Pihuamo, por brecha Llanitos-Canutillo, a 24 km, [19.4692, -103.3064], 1750 m, 14 May 1988, *Pichardo-A. 42* (NY); **México**: Mpio. Tlatlaya, El Potrero, cañada Agua Fría, 1.5 km al S de Tlatlaya, 18.6111, -100.2117, 1650 m, 2 Jun 2001, *Aguilera-Gómez 946* (MEXU); Mpio. Tejupilco de Hidalgo, 22.7 km W of Estanco on the road to Cañada de Nanchititla, top of Mesa de Nanchititla, [18.8410, -100.4136], 1950 m, 11 Oct 1985, *Bartholomew 2937* (GH, MEXU, NY); Dto.Temascaltepec, Cumbre de Tejupilco, [19.1322, -99.9176], 2000 m, 20 Jul 1932, *Hinton 1089* (G); Dto. Temascaltepec, Rincón, [18.9017, -100.1200], 27 Dec 1933, *Hinton 3029* (MEXU, US); Dto. Temascaltepec, Pantoja, [18.8711, -100.0146], 6 May 1933, *Hinton 4080* (NY, US); Dto. Temascaltepec, Ypericones, [18.8720, -100.1844], 26 Jul 1933, *Hinton 4371* (G); Dto. Temascaltepec, Nanchititla, [18.8667, -100.4667], 14 Jun 1934, *Hinton 6159* (G, MO); Mpio. Tlatlaya, El Potrero, Cañada de Agua Fría, 3 km al S de Tlatlaya, 18.6106, -100.2139, 1650 m, 6 Aug 2004, *Martínez de la Cruz 212* (MEXU); **Michoacán**: Mpio. Uruapan, Hwy 37, km 85, 19.4167, -102.0833, 1500 m, 26 Jun 1981, *Hahn 597* (MO); Cascada de Tzararacua, cerca de Uruapan, [19.3550, -102.0831], 1500 m, 4 May 1966, *Rzedowski 22327* (IEB); Mpio. Uruapan, 85 km on Hwy 37, 1500 m, 26 Jun 1981, *Hahn 597* (MO); Mpio. Coalcomán, 4.5 km (en línea recta) al oeste de Las Joyas, 18.5014, -103.0939, 1970 m, 29 Aug 2008, *Steinmann 6347* (DAV); Mpio. Ziracuaretiro, Malpais de San Andres Coru, 19.4492, -101.9497, 1667 m, 25 Sep 2012, *Valentin-Martínez 309* (MEXU); **Morelos**: Mpio. Tlayacapan, Barranca Tepecapa, 18.9683, -99.0156, 1849 m, 17 Jul 2010, *R. Hernández-Cardenás 445.2* (IEB); Sierra de Ocuila, rumbo Mexicapa, 18.9100, -99.2300, 16–18 Dec 1938, *Lyonnet 2886* (US); Sierra de Ocuila, Apr 1948, *Lyonnet 480400003* (MEXU); Cuernavaca, [18.9085, -99.2362], Aug–Sep 1950, *Lyonnet 500800043* (MEXU); above Cuernavaca, 18.9100, -99.2300, 6500 ft, 24 Sep 1896, *Pringle 6505* (GH, MEXU, MO, NY); wet mountain canyon above Cuernavaca, [18.9583, -99.2750], 6500 ft, 26 Jun 1898, *Pringle 6877* (G, GH, GOET, MEXU, MO, NY, US, W); Barranca Atzingo, [18.9583, -99.2750], 1850 m, 23 Apr 1971, *Vázquez 3146* (MEXU); Cuernavaca, Barranca del Tecolote, 18.9500, -99.2736, 1750 m, 7 May 2005, *Zamudio Ruiz 13085* (TEX); **Oaxaca**: Mpio. Coicoyán, Yuvi Ndoso Itia, al SW de Coicoyán, 17.2500, -98.3000, 2200 m, 16 Mar 1988, *de Ávila 214* (MEXU, NY); Mpio. Coicoyán, Nami Yoo, al SW de Coicoyán, 17.2500, -98.3000, 2200 m, 3 Jun 1989, *de Ávila 630* (MEXU, NY); Dto. Juxtlahuaca, Mpio. San Martin Peras, a 2.7 km adelante de la desviacion de San Martin Peras, carretera para Coyoacan de las Flores, 17.2833, -98.2167, 2490 m, 20 Jun 1993, *Calzada 18444* (MEXU); Mpio. San Martín Peras, 2 km de la desviación a San Martín Peras, carretera Coicoyán de Las Flores-Santiago Juxtlahuaca, 17.3000, -98.1833, 2565 m, 10 Oct 1994, *Calzada 19461* (NY); Mpio. Santiago Juxtlahuaca, km 14 de San Miguel Cuevas a El Manzanal, deviación en Santa Rosa, 17.2167, -98.0500, 2300 m, 18 Feb 1995, *Calzada 19763* (IEB, MEXU, NY); Mpio. Santiago Juxtlahuaca, 3 km de San Pedro Chayuco, carretera por el aserradero, 17.2000, -98.0000, 1855 m, 19 Feb 1995, *Calzada 19781* (MEXU, NY, XAL); Mpio. Santiago Juxtlahuaca, Puerta de Luz, hacia la torre de microondas, entrada por Santa Rosa, vía San Miguel Cuevas a El Manzanal, 17.2167, -98.0500, 2350 m, 18 Apr 1995, *Calzada 19813* (IEB, MEXU, NY); 4–5 km del poblado El Manzanal, carretera a Infiernillo, 17.3500, -98.0400, 30 Jan 1996, *Calzada 20742* (MEXU, NY); Mpio Santiago Juxtlahuaca, faldas y cima del cerro de la torre de microondas de El Manazanal, 2395 m, 12 Sep 1996, *Calzada 21348* (BIGU, MEXU); Mpio. Santiago Juxtlahuaca, a 1 km de El Manzanal, carretera a Infiernillo, 17.2267, -98.0606, 2170 m, 2 Apr 1997, *Calzada 21632* (BIGU, MEXU, NY); Mpio. Santiago Juxtlahuaca, 2 km de Agua Fría, desviación por la carretera a Hierba Santa, 17.2092, -97.9781, 1740 m, 20 Apr 1997, *Calzada 21897* (MEXU, NY); Mpio. San Jeronimo Coatlan, 11 km al suroeste de San Jeronimo Coatlan, brecha a San Gabriel Mixtepec, 16.3333, -96.9500, 1990 m, 20 Feb 1988, *Campos-Villanueva 1280* (MEXU); Mpio. San Jeronimo Coatlan, 17.9 km al suroeste de San Jeronimo Coatlan, brecha a Piedra Larga, 16.2000, -96.9667, 1890 m, 17 May 1988, *Campos-Villanueva 1842* (MEXU); Mpio. San Jeronimo Coatlan, 11.5 km al suroeste de San Jeronimo Coatlan, brecha a Piedra Larga, 16.3333, -96.9500, 2060 m, 13 Aug 1988, *Campos-Villanueva 2222* (MEXU); Mpio. San jeronimo Coatlan, Cerro La Neblina, 18 km al NE de la entrada a Progreso, brecha San Jeronimo Coatlan, 16.1833, -96.9333, 1900 m, 19 Apr 1990, *Campos-Villanueva 3049* (MEXU); Dto. de Putla, Barranca del Pájaro, 20 km al N de Putla, [17.2067, -97.9292], 1400 m, 10 Jun 1985, *García-Mendoza 1556* (MEXU); Mpio. San jeronimo Coatlan, 40 km al SE de San Jerónimo Coatlán, camino a San Gabriel Mixtepec, a 3 km de la desv. a Progreso, [16.1539, -97.0586], 1300 m, 18 Apr 1990, *García-Mendoza 4611* (MEXU); Mpio. Santiago Yosondua, a la orilla del Río La Esmeralda, 50 m abajo del mirador, 16.8449, -97.5801, 1898 m, 6 Aug 2012, *García-Mendoza 9876* (MEXU); Mpio. Putla Villa de Guerrero, Río El Pajaro, [17.0236, -97.9167], 7 Jul 2008, *González 499* (IEB, MEXU); Mpio. Comaltepec, S. Comaltepec, 17.5500, -96.5167, 2000 m, 16 Apr 1988, *Hernández-García 278* (MEXU, NY); Distrito Juxtlahuaca, camino a Coicoyan de las Flores, desviacion a San Martin Peras, 17.2940, -98.1909, 2745 m, 8 Jul 2008, *Hernández 505* (IEB); Mpio. Putla Villa de Guerrero, Yucunicoco, 17.2047, -97.8864, 2142 m, 6 Sep 2008, *Hernández 541* (IEB); Mpio. Santiago Textitlán, en el arroyo de Providencia, 16.6901, -97.3109, 1431 m, 8 Aug 2006, *Jacobo-Salinas 309* (MEXU); Mpio. Santiago Textitlán, Paraje La Toma de Agua, rumbo a pueblo viejo, 16.6895, -97.2438, 1966 m, 31 Aug 2006, *Jacobo-Salinas 477* (MEXU); Mpio. Santiago Textitlán, 16.6895, -97.2438, 1966 m, 31 Aug 2006, *Jacobo-Salinas 482* (MEXU); Mpio. Santiago Textitlán, arriba de Barranca Nube, 16.6916, -97.2466, 1801 m, 28 Feb 2007, *Jacobo- Salinas 1726* (IEB, MEXU); Mpio. San Miguel del Puerto, Finca Montecarlo, 15.9140, -96.1326, 900 m, 15 Apr 2000, *López 36* (MEXU); Mpio. San Miguel del Puerto, 15.9828, -96.1036, 645 m, 11 May 2000, *López 122* (BIGU, DAV, IEB, MEXU); Mpio. San Miguel del Puerto, 15.9358, -96.1156, 990 m, 17 May 2000, *López 163* (IEB, MEXU); Mpio. San Miguel del Puerto, las Trancas de Sta. Rosa, 16 Jun 2000, *López 196* (MEXU); Mpio. San Miguel del Puerto, Pleito de Perro, 15.9911, -96.1153, 1030 m, 21 Jun 2000, *López 225* (IEB); Mpio. San Miguel del Puerto, Arroyo Arena, 15.9697, -96.0997, 835 m, 19 Jul 2000, *López 318* (IEB, MEXU); Mpio. Santos Reyes Topala, a 194 km al sur-sureste de Oaxaca camino a Puerto Escondido, 16.2183, -97.1428, 1905 m, 11 May 2001, *Martínez 33847* (MEXU); Mpio. Pluma Hidalgo, Finca La Cabaña, a 5 km al SE de Pluma Hidalgo, 15.9222, -96.3994, 753 m, 7 Aug 2001, *Martínez-Salas 34431* (MEXU); Mpio. San Miguel Chimalapa, 3 km al SE de Congregación Benito Juárez, 16.7000, -94.1167, 1100 m, 3 Sep 1984, *Maya-J. 562* (NY); Mpio. Santa María Chimalapa, Loma de Las Yeguas, ca. 3 km al NE de Benito Juárez, en el límite con el Mpio. de San Juan Chimalapa, ca. 41 km en línea recta al N de San Pedro Tapanatepec, 16.7500, -94.1356, 1100–1200 m, 20 May 1985, *Maya-Jimenez 1643* (MEXU); Mpio. Santa María Chimalapa, Cabecera del Cañón Hierba Santa, faldas S del Cerro Guayabitos, ca. 4 km en línea recta al NO de Benito Juárez, ca. 39 km en línea recta al N de San Pedro Tapanatepec, 16.7333, -94.1667, 1500–1700 m, 24 Jun 1985, *Maya-Jimenez 1804* (MEXU); Mpio. Santa María Chimalapa, cerca de Chocomanantlán, en el crucero de caminos que van a Los Pericos y a Benito Juárez, ca, 42 km en línea recta al NNE de San pedro Tapanatepec, 16.7333, -94.0667, 1100 m, 27 Jun 1986, *Maya-Jimenez 3482* (MEXU); Mpio. Santa María Chimalapa, Cerro El Retén, cerca del Paraje Palmero “El Progreso” y la vereda El Progreso-El Salto ca. 23 km al NE de Zanatepec, 16.6667, -94.2500, 10 Sep 1986, *Maya-Jimenez 3858* (MEXU); Mpio. Zimatlan de Álvarez, Paraje Río Salinas, 7 km al S de La Cofradia. Comunidad de San Pedro El Alto, [16.8025, -97.0613], 2120 m, 24 Oct 1998, *Andres Miranda 452* (MEXU); San Miguel del Puerto, Distrito Pochutla; Cafetal “Arroyo Arena,” 15.9777, -96.0989, 680 m, 19 Apr 2002, *Nava-Zafra 293* (IBUG, MEXU); Mpio. Santiago Juxtlahuaca, 19 km al SW de Santa Rosa sobre la carretera a San Miguel Cuevas, 17.2253, -98.0567, 2050 m, 28 Feb 1995, *Panero 5530* (NY); Mpio. San Miguel del Puerto, Corral de Piedra, carretera que va a San Miguel del Puerto, 15.9110, -96.1745, 22 Jun 2002, *Pascual 520* (IBUG); Mpio. San Miguel del Puerto, Placeton, 16.0041, -96.1274, 1773 m, 7 May 2004, *Pascual 1075* (IEB, MEXU); Mpio. San Miguel del Puerto, El Vigia, 16.0136, -96.1128, 1625 m, 15 Jun 2011, *Pascual 2383* (DAV); Mpio. San Miguel del Puerto, 4.7 km al N hacia Yuviaga, El Mirador, 15.9519, -96.1783, 630 m, 26 Oct 1999, *Perret 732* (IEB); Mpio. San Juan Mixtepec, Cava Ntataán, a 2 km al NO de Tinuma de Zaragoza, [17.3394, -97.9200], 2100 m, 19 Mar 1989, *Reyes-S. 1499* (MEXU); Mpio. Santa María Huatulco, aprox. 10 km al N de Santa María Huatulco, en la ranchería La Jabalina, junto al Río Tangolunda, [15.7981, -96.1142], 30 m, 22 Jan 1999, *Reyes-García 2931C* (MEXU); Mpio. San Miguel del Puerto, finca Montecarlo, 15.9942, -96.1094, 500 m al N por el camino de la zanja, 15.9942, -96.1094, 905 m, 19 May 2000, *Rivera-H. 2409* (MEXU); Mpio. San Miguel del Puerto, Rancho del Istmo, 700 m al S de la desviación a Rancho Sn. Agustín, hacia Xadani, 15.9881, -96.1011, 815 m, 20 May 2000, *Rivera-H. 2494* (IEB, MEXU); Mpio Juquila, 4 km al S de Lachao, km 183 carretera Oaxaca-Puerto Escondido, [16.2539, -97.1856], 1850 m, 14 Apr 1965, *Rzedowski 19597* (IEB, MEXU, US, XAL); Mpio. Santiago Textitlán, Paraje El Limón, 16.6897, -97.3456 1253 m, 18 May 2006, *Salas-M. 1253* (MEXU); Mpio. San Miguel del Puerto, La Hamaca, Copalita, 4.7 km al E, 15.9011, -96.1658, 325 m, 27 Jul 2000, *Salas 3064* (IEB); Mpio. San Miguel del Puerto, brecha Xandani-San Miguel del Puerto, camino a Las Blas, 15.9214, -96.1094, 500 m, 28 Sep 2001, *Salas-M. 4141* (IEB, MEXU); Mpio. Santiago Textitlán, Paraje Río Comal, 16.6921, -97.2936, 1553 m, 18 May 2006, *Salas-Morales 5797* (IEB, MEXU); Mpio. San Miguel del Puerto, Cafetal Arroyo Arena, 15.9778, -96.0982, 700 m, 28 May 2006, *Salas 5978* (MEXU); Mpio. San Miguel del Puerto, Orilla del Río San Lorenzo, 15.9804, -96.0956, 520 m, 28 May 2006, *Salas 5983* (IEB, MEXU); Mpio. San Miguel del Puerto, camino hacia el Punto Trino, 16.0119, -96.1214, 1720 m, 27 Apr 2005, *Sanchez-Martínez 739* (IEB); Mpio. Santiago Textitlán, Sola de Vega, S de Tierra de Yunta, 16.7798, -97.2935, 2310 m, 18 Jul 2006, *Sanchez-Martínez 1709* (IBUG, IEB, MO); Dto. Tlaxiaco. Santiago Yosondua. Cueva El Expresidente, debajo de la Cascada La Esmeralda, 16.8333, -97.5667, 1876 m, 8 Aug 2012, *Sandoval-Gutiérrez 142* (MEXU); Dto. Tlaxiaco. Santiago Yosondua, Paraje El Limón, a 100 m del Río Yutama, 16.8000, -97.5833, 1470 m, 17 Jul 2013, *Sandoval-Gutiérrez 967* (MEXU); Mpio. San Miguel del Puerto, Rancho San Agustín, entrada al Rancho, 15.9919, -96.1072, 860 m, 30 May 2001, *Saynes-V. 2150* (MO, MEXU); Mpio. San Miguel del Puerto, 800 m al SW de la Finca el Faro, sobre brecha a la Finca la Constancia, 15.9960, -96.1181, 1050 m, 29 Jun 2001, *Saynes 2280* (IEB); Mpio. San Miguel del Puerto, 600 m línea recta al NE del Rancho San Agustin; rumbo a el Encinal, 15.9957, -96.1019, 880 m, 04 Jun 2002, *Saynes-V. 2966* (IBUG); Mpio. Putla, ca. 4 km al SE de Pueblo Nueva, [16.8886, -97.9314], 920 m, 18 Jun 1988, *Solano-C. 345a* (MEXU, MO); Dto. Santiago Juxtlahuaca, 19 km al SW de Juxtlahuaca, 5 km en la misma dirección de Sta. Ma. Asunción, [17.1970, -97.9561], 1900 m, 4 Apr 1982, *Tenorio-L. 178* (MEXU, NY); Mpio. Totontepec Villa de Morelos, Santa María Tiltepec, [17.4675, -97.3555], 2300 m, 20 Jan 1984, *Tenorio-Lezama 5361* (MEXU); Dto. Tehuantepec; 8.9 km al N de Lachiguti, [16.7299, -95.5245], 10 ec 1983, *Torres-C. 4310* (MEXU); 7 km al n de Putla carr. a Tlaxiaco, [17.0419, -97.8841], 820 m, 3 Jul 1984, *Torres-C. 5491* (MEXU); Sierra de Miahuatlan, 19 km al NW de Piedra Larga carr. Puerto Escondido, [16.5983, -95.8050], 1 Aug 1984, *Torres-C. 5814* (MEXU); Mpio. Juchatengo, 9.6 km al SE del Cerro de Vidrio carr. Oaxaca-Puerto Escondido, [17.3550, -96.2983], 1850 m, 7 Aug 1984, *Torres-C. 5827* (MEXU); Mpio. Concepción Progreso, 15 km al E de Putla, en el Cerro Cacao, [17.0222, -97.8264], 1020 m, 9 Jun 1985, *Torres-C. 6722* (MEXU); Mpio. San Sebastian Coatlan, 11 km al E de Piedra Larga, pasando el campamento de la Papelera Tuxtepec, camino a Miahuatlán, [16.1832, -96.8360], 1250 m, 16 Jun 1986, *Torres-C. 8589* (MEXU); Mpio. San Pedro Tapanatepec, 16 km al N de Rizo de Oro, carretera a Diaz Ordaz, 16.5667, -94.1833 1490 m, 4 Nov 1987, *Torres-C. 10580* (IBUG, MEXU); Mpio. San jeronimo Coatlan, 17.9 km al suroeste de San Jeronimo Coatlan, carretera Mihuatlan-Piedra Larga, 16.2000, -96.9500, 1800 m, 13 Dec 1987, *Torres-C. 10825* (MEXU); Mpio. San Jeronimo Coatlan, 2 km al noroeste de la entrada a Progreso la cual se encuentra 41 km al oeste de San Jeronimo Coatlan o 12.3 km al NE de Piedra Larga, 16.1500, -97.0167, 1390 m, 20 Mar 1988, *Torres-C. 11883* (MEXU); Mpio. Santiago Textitlán, La Cieneguilla, 16.7350, -97.2289, 2541 m, 7 Aug 2006, *Trujillo-Vásquez 252* (MEXU); Mpio. Santiago Textitlán, Paraje Comen Tierra, 16.7751, -97.2927, 2202 m, 2 Mar 2007, *Trujillo-Olazo 1748* (MEXU); Mpio. Santiago TextitlánTextitlán, , 16.6889, -97.2628, 1649 m, 19 Mar 2007, *Trujillo-Olazo 1854* (MO); Santiago Textitlán, Banco de Jardin, 16.6613, -97.2793, 1937 m, 23 Mar 2007, *Trujillo-Olazo 1877* (IEB, MO); Mpio. San Miguel del Puerto, ribera del Arroyo Piñanona, 15.9300, -96.1718, 365 m, 17 May 2002, *Vazquez 1240* (DAV); Mpio. San Miguel del Puerto, El Placeton a km en línea recta de la finca El Faro, 16.0043, -96.1274, 1760 m, 17 Apr 2002, *Velasco-Gutiérrez 514* (IBUG); Mpio. Santa Cruz Itundujia, Predio de Sr. Tomás Riano, Agencia de Zaragoza, 16.6650, -97.7822, 1715 m, 19 May 2008, *Velasco-Gutiérrez 2624* (IEB); Mpio. Santiago Textitlán, Paraje el Boludo, sobre la brecha, 16.7147, -97.2579, 2053 m, 15 Jun 2006, *Zarate-Marcos 26* (MEXU); Mpio. Santiago Textitlán, comunidad de Río Humo, Paraje La Peña, 16.7219, -97.2433, 31 Jul 2006, *Zarate-Marcos 217* (MEXU); Mpio. Santiago Textitlán, Río Tigre, 16.6944, -97.2843, 1562 m, 13 Aug 2006, *Zarate-Marcos 242* (MEXU); same location and date, *Zarate-Marcos 248* (MEXU).

**Nicaragua. Estelí**: Cerro Quiabú, situado unos 8 km al noroeste de la ciudad de Estelí, [13.1167, -86.4333], 1500–1600 m, 19 Oct 1979, *Grijalva 611* (MO); Cerro Quiabú, al NO de Estelí, 13.1167, -86.4333, 1500 m, 25 Jul 1980, *Moreno 1371* (MO); Cerro Quiabú, 13.1167, -86.4333, 1604 m, 14 Jan 1981, *Moreno 6071* (MO); Quetzalcayán, al E del Cerro Tisey, 900–1100 m, 15 Oct 1982, *Moreno 17866* (MO, NY); N Slope of Cerro El Fraile, 13.4167, -86.2500, 1160–1200 m, 28 Sep 1980, *Stevens 18071* (MO); same location and date, *Stevens 18101* (MO); same location and date, *Stevens 18102* (MO); **Nueva Segovia**: 0.7 km W of Hacienda Las Brisas, S slope of Cerro Mogotón, 13.7372, -86.3825, 1456 m, 31 Aug 2015, *Stevens 36930* (MO).

**6. *Lycianthes barbatula***

**Guatemala. Chimaltenango**: above Finca Montevideo, along Barranco Espinazo and tributary of Río Pantaleón, lower and middle southwestern slopes of Volcán Fuego, 1200–1600 m, 20 Sep 1942, *Steyermark 52055* (US); Volcán Acatenango, Aldea Quisache, 14.5181, -90.2844, 1500 m, 19 May 2004, *Véliz 15261* (BIGU, MEXU); **Quetzaltenango**: slopes and ridges between Quebrada Chicharro and Montaña Chicarro, on SE-facing slopes of Volcán Santa María, 1300–1400 m, 18 Jan 1940, *Steyermark 34354* (US).

**Mexico. Oaxaca**: Dto. Pochutla, Mpio. Pluma Hidalgo, Cerro Espino, 4 km al E de Concordia, brecha a finca cafetalera Monte Cristo, 15.8667, -96.4167, 920 m, 21 Jan 1988, *Campos-V. 1171* (DAV221866, MEXU); Dto. Pochutla, Mpio. San Miguel del Puerto, Las Lobas, 16.0125, -96.1056, 1482 m, 18 May 2005, *Pascual 1501* (MEXU); Dto. Pochutla, Mpio. San Miguel del Puerto, El Vigia, 16.0136, -96.1128, 1625 m, 5 Jun 2006, *Pascual 1888* (MEXU, MO); Dto. Pochutla, Mpio. San Miguel del Puerto, El Vigía, 16.0125, -96.1108, 1519 m, 24 Jun 2008, *Pascual 2155* (DAV); Dto. Pochutla, Mpio. San Miguel del Puerto, limite entre El Faro y Montecarlo, 16.0092, -96.1100, 1200 m, 13 Jan 2010, *Salas M. 6870* (DAV); Dto. Pochutla, Mpio. San Miguel del Puerto, La Merde, 16.0139, -96.1128, 1595 m, 21 Jan 2010, *Salas-M. 7072* (DAV); Dto. Pochutla, Mpio. San Miguel del Puerto, Finca Montecarlo, 600 m al N, 16.0081, -96.1086, 1170 m, 31 May 2001, *Saynes-Vazquez 2178* (MEXU).

**7. *Lycianthes breedlovei***

**Mexico. Chiapas**: Mpio. San Cristóbal de las Casas, near summit of Hueitepec near Las Casas, [16.7506, -92.6735], 22 Apr 1945, *Alexander 1212* (MEXU, NY); Mpio. Tenejapa, in Colonia ‘Ach’lum, [16.7753, -92.4467], 9100 ft, 23 Aug 1966, *Breedlove 15206* (NY); Mpio. Jitotol, about 7 miles N of Jitotol along a side road to an oil well, [17.1417, -92.8842], 6700 ft, 28 Aug 1966, *Breedlove 15412* (DUKE); Mpio. San Andrés Larrainzar, near the summit of Chuchil Ton, NE of Bochil [16.9958, -92.8932], 2700 m, 3 Aug 1972, *Breedlove* 26811 (MO); Mpio. La Independencia, third ridge along logging road from Las Margaritas to Campo Alegre, [16.4756, -91.8234], 2300 m, 18 Feb 1973, *Breedlove 33583* (CAS); Mpio. La Independencia, third ridge along logging road from Las Margaritas to Campo Alegre, [16.4756, -91.8234], 2300 m, 6 May 1973, *Breedlove 34793* (CAS, MEXU, LL); Mpio. La Independencia, third ridge along logging road from Las Margaritas to Campo Alegre, [16.4756, -91.8234], 2300 m, 24 Oct 1976, *Breedlove 41074* (CAS, MEXU, MO); Mpio. La Independencia, Third ridge along logging road from Las Margaritas to Campo Alegre, [16.4756, -91.8234], 2300 m, 3 Jul 1981, *Breedlove* 51326 (CAS, MEXU, MO); Mpio. Tenejapa, Near Paraje Banabil, [16.7803, -92.5108], 2713 m, 8 Oct 1981, *Breedlove 53375* (LL); Mpio. La Independencia, 6–10 km NE of La Soledad along logging road from Las Margaritas to Campo Alegre, [16.42, -91.85], 1600, 19 Nov 1981, *Breedlove 55673* (CAS, NY); Mpio. Tenejapa, 25 km al NE de San Cristóbal de las Casas, sobre el camino a Matzala [16.7365, -92.4239], 2350 m, 25 Nov 1982, *Cabrera-C. 3800* (MEXU, MO, NY); Mpio. Tenejapa, 3 km al oeste de la carretera San Cristóbal de Las Casas-Tenejapa, sobre el camino a Matzala [16.758936, -92.58676], 29 Sep 1983, *Cabrera-C. 5757* (MEXU); Mpio. Tenejapa, along the road to the town of Matzam, ca. 1.5 km from the eastern outskirts of the town of Las Ollas, 16.78323, -92.52748, 2484 m, 13 Sep 2017, *Dean 9531* (DAV); Mpio. San Cristóbal de las Casas, Estación Biológica Huitepec-PRONATURA, [16.7471, -92.6826], 2550 m, 3 Jun 1991, *González-Espinosa 1489* (MEXU); Mpio. Pueblo Nuevo Solistahuacán, Clínica La Yerbabuena [17.1830, -92.900], 2100 m, 23 Oct 1989, *Heath 2062* (MEXU); Mpio. Tenejapa, 22 km from San Cristóbal de las Casas on the road to Tenejapa, then right 3 km on the road to Matzam, [16.7988, -92.4728], 2400 m, 29 Sep 1984, *Huft 2195* (MEXU); Mpio. Huixtán, Rancho Merced Bazom, 16.7014, -92.6097, 2450 m, 2 Jul 1994, *Ico 26* (MEXU); Mpio. Pueblo Nuevo Solistahuacán, northern highlands of Chiapas, Jitotol Ridge, 3 km NW of Pueblo Nuevo Solistahuacán, 17.5, -92.67, 6700 ft, 1 Jun 1971, *Lathrop 7487* (CAS); Mpio. Tenejapa, Paraje Navil, [16.8417, -92.5108], 2000 m, 15 Nov 1982, *Méndez-Ton 5054* (MEXU, MO); Mpio. Tenejapa, Rancho Banabil, [16.7833, -92.5139], 2200 m, 26 Apr 1983, *Méndez-Ton 5918* (MEXU, MO, WIS, XAL); Mpio. San Cristóbal de Las Casas, Santa Cruz en San Felipe, [16.7134, -92.6640], 15 Nov 1986, *Méndez-Ton 9846* (CAS, GH, MEXU, MO, NY, TEX); Mpio. Chamula, Yaal Ichin (yoc sac lum), 16.846389, -92.67583, 2180 m, 15 Jun 1993, *Ruíz-Díaz 116* (CAS); Mpio. San Juán Chamula, Paraje Nab ta Kokontik, 7900 ft, 20 May 1988, *Santíz-Ruíz 854* (CAS, MO, NY, TEX); Mpio. San Juán Chamula, Bautista Grande, [16.797778, -92.72944444], 13 Jun 1988, *Santíz- Ruíz 904* (CAS, MEXU, MO, TEX, WIS); Mpio. Tenejapa, Colonia of ‘Ach’lum, [16.7753, -92.4467], 9100 ft, 15 May 1967, *Shilom-Ton 2340* (CAS).

**8. *Lycianthes caeciliae***

**Mexico**. **Veracruz**: Mpio. Coatepec, La Cortadura, falda este del Cofre de Perote, 19.49, -97.04, 2100 m, 2 May 2005, *Castillo-Campos 21514* (IEB, XAL); same location, 6 May 2005, *Castillo-Campos 21543* (XAL); same location, 6 May 2005, *Castillo-Campos 21552* (XAL); same location, 10 Aug 2005, *Castillo-Campos 21766* (MEXU, XAL); same location, 27 Feb 2005, *Castillo-Campos 22271* (XAL); same location, 18 Apr 2007, *Castillo-Campos 22711* (XAL); same location, 25 Apr 2007, *Castillo-Campos 22754* (XAL); same location, 25 Apr 2007, *Castillo-Campos 22773* (XAL); same location, 23 May 2007, *Castillo-Campos 22828* (IEB, XAL); Mpio. Coatepec, La Cortadura, falda este del Cofre de Perote, 19.4900, -97.0427, 1800 m, 8 May 2019, *E. Dean 10030* (DAV, MEXU, XAL); Mpio. Coscomatepec, [19.0679, -97.0473], 10 May 1987, *Matuda 1309* (MEXU); Mpio. Xico, terracería, 1 km al NE de Oxtlapa, rumbo a Tonalco, faldas del Cofre de Perote, 19.4305, -97.1013, 2250 m, 27 Sep 2001, *Rincón-G. 2604* (MEXU, XAL); Acajete, Plan de Cedeño, [19.5934, -97.0289], 1750 m, 23 Jun 1976, *Ventura-A. 12881* (IEB).

**9. *Lycianthes ceratocalycia***

**Guatemala. Alta Verapaz**: Mpio. San Cristóbal, Finca Pamac II, 15.3986, -90.5883, 2146 m, 16 Aug 2015, *Borrayo MGC-5* (BIGU); Montaña Sacranix: Cooperativa Sanimtaca (Anexo Cooperativa Samac), etwa 12 km Luftlinie WNW Cobán, alter Maya-Weg am oberen Rand des Karstkraters, 15.4833, -90.4667, 1400–1550 m, 5 Apr 2001, *Förther 11146* (MSB, W); **El Quiché**: Mpio. Chajul, Pombaltzé, 15.6851, -91.1396, 1900 m, Nov 2016, *Tribouillier 1* (DAV); **Huehuetenango**: Mpio. Nentón, along road from Nuevo San José Frontera to Las Palmas, 16.0333, -91.5500, 900–1200 m, 26 Mar 2009, *Christenhusz 5628* (MO); Mpio. San Mateo Ixtatán, E of the town of Aguacate, along road to town of Yalanhuitz, 16.0377, -91.4662, 1567 m, 15 Aug 2017, *Dean 9510* (DAV); Mpio. Barillas, San José Maxbal, camino a Barillas, [15.8000, -91.3000], m, 20 Sep 2006, *Pérez 1355* (MEXU1433300); **Quiché**: Mpio. Chajul, camino a Amachel, 15.6878, -90.9928, 1553 m, 25 Jun 2006, *R. Ávila 3038* (MEXU); Uspantan Montaña Chimel, 15.4592, -90.7875, m, 25 Apr 2007, *Morales 4670* (MEXU).

**Mexico. Chiapas**: Mpio. La Trinitaria, slopes E of Laguna Tziscao, Monte Bello National Park, [16.0786, -91.6589], 1300 m, 13 May 1973, *Breedlove 35229* (MO, NY); Mpio. La Trinitaria, Parque Nacional Lagunas de Montebello, at an outlook (mirador) above and to the E of Dos Lagunas, the mirador is on the W side of Hwy 307 to the E of Lago Tziscao, ca. 7 km E of the intersection of Hwy 307 and the road to Cinco Lagos, 16.0939, -91.6361, 1461 m, 14 Sep 2017, *Dean 9532* (DAV, MEXU).

**10a. Lycianthes chiapensis var. chiapensis**

**Guatemala. Quetzaltenango**: Montaña Chicharro, on lower SE-facing slopes of Volcán Santa María, 2–4 miles south of Santa María de Jesús, [14.693888, -91.542336], 1400–1500 m, 17 Jan 1940, *Steyermark 34284* (F1043138); El Pocito, south of San Martín Chile Verde, on road to Colomba, [14.801692, -91.672212], 2200 m, 27 Jan 1941, *Standley 84958* (F1198820); above Mujuliá, between San Martín Chile Verde and Colomba, [14.739365, -91.686351], 1800 m, 1 Feb 1941, *Standley 85684* (F1124852); **San Marcos**: above Finca El Porvenir on “Todos Santos Chiquitos,” lower facing slopes of Volcán Tajumulco, [14.978158, -91.93612], 1300–1500 m, 7 Mar 1940, *Steyermark 37164* (F1035062); near Aldea Fraternidad, between San Rafael Pie de la Cuesta and Palo Gordo, west facing slope of the Sierra Madre Mountains, [14.935493, -91.872523], 1800–2400 m, 10–18 Dec 1963, *Williams 26281* (NY); **Suchitepéquez**: Barranca by Loma Grande, above Finca El Naranjo, on Volcán Santa Clara, [14.623345, -91.353277], 1950–2100 m, 2 Jun 1942, *Steyermark 46833* (G, NY); **Mexico. Chiapas**: Motozintla de Mendoza, 45–50 km NE of Huixtla along road to Motozintla, [15.3317, -92.2714], 1900 m, 28 Dec 1972, *Breedlove 31037* (MO2610142); Mpio. La Concordia, El Triunfo Reserve, trail WSW from Palo Gordo towards Finca Catarina, 15.6667, -92.8500, 1850 m, 25 Feb 1990, *Hampshire 671* (MO5755435); Mpio. La Concordia, Reserva de la Biosfera El Triunto, Poligono III, Cerro Quetzal, 50 km al S de la Colonia Independencia, 15.7067, -92.9378, 1856 m, 1 Apr 2001, *López-Hernández 1* (MO4793866); Cerro del Boqueron, [15.1735, -92.3363], Jun 1914, *Purpus 7166* (MO765044, NY); Mpio. Unión Juárez, La Esperanza, 10 km al S del Toquian, [15.0558, -92.1294], 2000 m, 12 Jun 1987, *Ventura 4564* (IEB113835, MEXU629065, NY, TEX barcode # 00226959).

**10b. Lycianthes chiapensis var. sparsistellata**

**Guatemala. Alta Verapaz**: at Orchigonia orchid nursery/preserve outside of the city of Cobán along Guatemala Hwy 14, 15.4373, -90.4120, 1487 m, 10 Aug 2017, *Dean 9507* (DAV); **El Progreso**: Cerro Pinalón, Sierra de las Minas, San Acasaguastlán, 15.0656, -89.9833, 2230 m, 1 Mar 2007, *Flores 3548* (MO).

**Honduras. Copán**: Al NE de Quebrada Cañón Oscuro, 10.8 km al noroeste de Florida, en el Parque Nacional Cerro Azul, 15.0833, -88.9166, 1680 m, 11 Feb 1992, *Darío 45* (NY); S slopes of Montaña Cerro Azul, 14 km NE of Florida, Cerro Azul National Park, 15.10, -88.9166, 1500 m, *Thomas 236* (MO); **Cortés**: filo entre Cerro Cantiles y Cerro Jilinco, 20 km al oeste de San Pedro Sula, en el Parque Nacional Cusuco, 15.50, -88.2333, 2000 m, 19 Mar 1993, *Darío 338* (NY); along trail from new park station building to Cerro Cusuco, ca. 24 km W of San Pedro Sula, ca. 15 km S of Cuyamel, Parque Nacional Cusuco, 15.5166, -88.2333, 1940 m, 22 Mar 1993, *Evans 1506* (MO); Montaña Idalfonso N de Cofradía, 2100 m, 17–18 Apr 1957, *Molina-R. 8216* (NY); nuclear zone of the Cusuco National Park, Cuenca Qbr. Cantiles. Quebrada Cantiles watershed, [15.25, -88.25], 1875 m, 22 Mar 1993, *Thomas 706* (MO, NY); **Ocotepeque**: Cordillera Merendón vicinity of El Portillo, 2000 m, 2 Sep 1975, *Molina-R. 31004* (MO); Cordillera Merendón, above El Portillo, 20 km E of Nuevo Ocotepeque, [14.4667, -89.0667], 2000 m, 17 Jan 1976, *Molina-R.* 31310 (MO).

**Mexico. Chiapas**: Mpio. La Trinitaria, slopes E of Laguna Tzikaw [Tziscao], Monte Bello National Park, [16.0873, -91.6625], 1300 m, 16 Nov 1972, *Breedlove 29617* (MO, NY); Mpio.La Trinitaria, on slopes adjacent to Dos Lagos, Lagos de Montebello National Park, [16.0932, -91.6368], 1500 m, 28 Nov 1976, *Breedlove 41909* (MO); Mpio. La Trinitaria, Montebello National Park, slopes E of Laguna Tziscao, [16.0873, -91.6624], 1380 m, 18 Dec 1980, *Breedlove 48764* (MO3657748, NY); La Trinitaria, near Lago Montebello, Lagos de Montebello National Park, [16.1044, -91.7028], 1370 m, 14 Aug 1981, *Breedlove 52227* (MO, NY); Mpio. Pantepec, slope above Rayón along road to Pantepec and Tapalapa, [17.2018, -93.0126], 1770 m, 22 Sep 1981, *Breedlove 53009* (MO); Mpio. La Trinitaria, between Lago Tziscao and Dos Lagos, Lagos de Montebello National Park, 1372 m, 13 Oct 1981, *Breedlove 53416* (MO); along Highway 195 between Bochil and Pichucalco, 8 miles NW of Pueblo Nuevo Solistahuacan, on steep slopes below lookout, [17.2100, -92.9600], 1900–1950 m, 25 Jan 1979, *Croat 46360* (MO); San Miguel Chimalapa, Cima del Cerro Salomón, al NO de Benito Juárez, ca. 44 km en línea recta al N de Sn Pedro Tapantepec, 16.7708, -94.1953, 1770 m, 7 Apr 1986, *Ishiki 1451* (NY); Mpio. La Trinitaria, Parque Nacional Lagunas de Montebello, cerca de la Laguna Tziscao, 3 km al E, [16.0889, -91.6435], 15 Nov 1984, *Téllez-V. 7946* (MO); **Oaxaca**: Mpio. San Miguel Chimalpa, Cerro Sabinal, ca. 2 km al SO de Cerro Guayabitos, ca. 3 km en línea recta al NNO de Díaz Ordaz, ca. 40 km en línea recta al N de San Pedro Tapanatepec, al O de la cima del cerro, 16.7333, -94.1917, 1500 m, 21 Dec 1984, *Wendt 4678* (NY); **Veracruz**: Mpio. Soteapan, Cerro Campanario, 950 m, 14 Jul 1987, *Acosta-P. 1662* (NY); Mpio. Soteapan, along trails to base of Volcán Santa Marta, 0–3 km E village of Santa Marta, 18.3500, -95.8667, 1100–1200 m, 29 Jun 1982, *Nee 24700* (NY); Mpio. Soteapan, 2 km SW del Ejido G. Victoria, 500 m, 18 May 1986, *Vázquez-T. 3505* (WIS).

**Nicaragua. Jinotega**: Kilambé, “Paricutín,” 4 km al SE de Cerro Kilambé, 13.5833, -85.6666, 800–1000 m, 25 Mar 1981, *Moreno 7447* (MO); Cerro Kilambé, falda E del Pico Piedra Pelona, 13.5666, -85.6666, 1300–1400 m, 28 Mar 1981, *Moreno 7763* (MO); **Matagalpa**: Fuente Pura, km 142, carretera Matagalpa-Jinotega, 13.00, -85.9166, 1400–1450 m, 26 Aug 1982, *Moreno 17058* (MO); NW slope of Cerro El Picacho, 13.00, -85.9166, 1420–1520 m, 25 May 1983, *Stevens 22144* (MO).

**11. *Lycianthes ciliolata***

**Field Origin Unknown**: *Bernhardi herbarium s.n*, (MO); Leipzig Botanic Garden, Germany, no date or coll. number, tag on specimen says 1939 (M); probably a European Botanic Garden specimen, *Beer 853* (W); probably from a German botanical garden, *ex herbario Bitter s.n.* (GOET); H. B. Leipzig, (Leipzig Botanic Garden), Germany, 1857, *Kunze? s.n.* (BR); Leipzig Botanic Garden, Germany, *Kunze s.n.* (W); Horto. Bot. Turionor (Zurich Botanic Garden), *Regel 478*? (ZT); cultivated in the garden of Van Houtte, France, 17 Sep 1849, *Van Houtte 390* (K); H. B. Berol, (Berlin Botanic Garden), Germany, 1854?, *Herb. Vocke. s.n.* (GOET).

**Guatemala. Baja Verapaz**: Agua Caliente, [14.8833, -90.2833], 755 m, 25 May 1906, *Cook 38* (US); Patal, zwischen Santa Rosa und Tastic, [15.2333, -90.3667], 5000 ft, Sep 1888, *von Tuerkheim 1434* (F, GH, K, US); **Huehuetenango**: Vicinity of Chinacho, 10 km W of Zacaleu Ruins, 1900 m, 14 Sep 1971, *Molina-R. 26471* (F); Trail between Ixteapoc and Soloma, Sierra de los Cuchumatanes (near Soloma), [15.6500, -91.3333], 2400–2500 m, 8 Jul 1942, *Steyermark 48444* (F); Cerro Pixpix, above San Ildefonso, Ixtahuacan, [15.4675, -91.8116], 1600–2000 m, 15 Aug 1942, *Steyermark 50597* (F, NY); Cumbre Papal, on S-facing slopes between Cuilco and Ixmoqui, [15.4167, -91.9500], 1400–3000 m, 19 Aug 1942, *Steyermark 50925* (F, G); Slopes above La Libertad on Cerro Pueblo Viejo, [15.50, -91.5667], 1900 m, 20 Aug 1942, *Steyermark 50981* (F); **Totonicapán**: Cerro María Tecum, Sierra Madre Mountains, 10–20 km E of Totonicapán, [14.8959, -91.2636], 3100–3400 m, 16 Dec 1962, *Williams 23156* (MO).

**Mexico. State unknown**: 1833, *Andrieux, 163* (G); *Liebmann 1451* (C); Vermultl. Provinz Veracruz oder Oaxaca, Jul 7 1961, *Schwabe s.n.* (B); 1787–1795–1804, *Sessé, Moçiño, Castillo et Maldonado 1529* (photo NY – of Madrid specimen, F negative 48221; another photo at NY of a duplicate specimen is *L. moziniana*); *1787–1795–1804, Sessé, Moçiño, Castillo et Maldonado 5389* (photo NY – of Madrid specimen – F negative 4822); 1787–1795–1804, *Sessé, Moçiño, Castillo et Maldonado 5389* (F – newer label says 5389, small old label says 239 and *S. uniflorum*); Toluca? (probably in error), *Wawra 1214* (W); **Chiapas**: Mpio. Tenejapa, near the schoolhouse of Pokolum in the Paraje of Sibanil Ha’, [16.8751, -92.4662], 5100 ft, 10 Jul 1964, *Breedlove 6100* (DS); Mpio. Teopisca, NW edge of Teopisca along Mexican hwy 190, [16.5566, -92.5301], 5900 ft, 25 Jun 1965, *Breedlove 10493* (DS); Mpio. Tuxtla Gutiérrez, 15 km N of Tuxtla Gutiérrez along rd to El Sumidero, [16.8143, -93.07339], 3800 ft, 2 Jul 1965, *Breedlove 10644* (DS, F); Mpio. Tenejapa, near the schoolhouse of Pokolum, paraje of Sibanilha’, [16.8751, -92.4662], 5200 ft, 15 Jul 1965, *Breedlove 11014* (DS, ENCB, F); Mpio. Venustiano Carranza, 3 mi S of Aguacatenango along rd to Pínola Las Rosas, [16.4316, -92.39972], 5600 ft, 22 Jul 1965, *Breedlove 11183* (DS); Mpio. Tenejapa, Paraje of Kulak’tik, [16.8343, -92.4971], 5500 ft, 18 Jul 1966, *Breedlove 14588* (DS, F, LL); Mpio. La Trinitaria, Lagos de Montebello, 42 km NE of La Trinitaria, along the Comitán River at its sumidero, 1300 m, 23 Oct 1971, *Breedlove 21144* (DS); Mpio. Totolapa, Rancho Ch’ a ha’, 5–6 km W of Teopisca, [16.5388, -92.5280], 1500 m, 26 Nov 1971, *Breedlove 22870* (DS); Mpio. San Andrés Larrainzar, near the summit of Chuchil Ton, NE of Bochil, [16.9935, -92.8176], 2700 m, 17 Oct 1972, *Breedlove 29298* (DS, MO); Mpio. San Cristóbal de Las Casas, on road to San Lucas Zapotal, 2–4 km from Mex hwy 190, [16.7266, -92.6858] 2400 m, 8 Sep 1974, *Breedlove 37317* (DS, MO); a 5 km al N de la carretera San Cristóbal de las Casas-Comitán, con rumbo a Ococingo, [16.6976, -92.6046], 19 Jun 1982, *Cabrera 2813* (NY); Mpio. San Cristóbal de las Casas, just outside city of San Cristóbal de las Casas, along rd to hwy 190 which diverges from rd to Tenejapa, nearly opposite trailer park, near Almolonga River, [16.6991, -92.6148], 2210–2240 m, 16 Jul 1990, *Dean 213* (DAV, MEXU); Mpio. Zinacantán, hills SE of the town of Zinacantán, along footpath to Paraje Sok’on, several km uphill from Zinacantán, [16.7501, -92.7029], 2463 m, 17 Jul 1990, *Dean 214* (DAV, MEXU, NY, UC, XAL); Mpio. Teopisca, Ca. 8 rd km NW of Teopisca along hwy 190, NE side of road, [16.5844, -92.5021], 2103 m, 18 Jul 1990, *Dean 215* (DAV, MEXU); no exact locality, *Ghiesbrecht 58* (GH – mixed collection with *L. pilifera*); terre froide, Jul 1864–1867, *Ghiesbrecht 827* (BM, GH, MO); Yochib Ja’, 5 km al N de Amatenango del Valle, [16.5701, -92.4372], 5500 ft, 15 Apr 1988, *Gómez-L. 421* (MO); Mpio. San Cristóbal de Las Casas, Estación Biológica Huitepec-PRONATURA, 16.7439, -92.6708, 2400 m, 13 Jun 1989, *González-Espinoza 668* (MEXU); Ixtapa, carretera 190 en el km 75 entre Tuxtla Gutiérrez y San Cristóbal de las Casas, [16.7552, 92.9233], 2250 m, 2 Jul 1991, *González-V. 3951* (WIS); 32 mi SE of San Juan Cristóbal de las Casas, [16.6569, -92.5659], 7100 ft, 23 Jun 1950, *Johnston 53-666* (TEX); Mpio. Zinacantán, valley floor in Zinacantán Center, [16.7706, -92.7293], 6700 ft, 7 Jun 1966, *Laughlin 1012* (DS); Mpio. Zinacantán, Paraje Vo’bits, [16.7379, -92.7659], 4300 ft, 7 Jun 1966, *Laughlin 1016* (DS); Mpio. Venustiano Carranza, Venustiano Carranza, [16.3502, -92.5676], 800 m, 13 Jun 1945, *Matuda 30154* (MEXU); Mpio. San Cristóbal de Las Casas, Col. Carrizal, 700 m al oriente de la escuela, 16.6575, -92.6975, 2250 m, 6 Jun 1995, *Mejia 429* (XAL); Mpio. Tenejapa, Pocolum, [16.8798, -92.4635], 1600 m, 15 Sep 1982, *Méndez-T. 4604* (NY); Mpio. Tenejapa, Shishitonil, [16.8483, -92.4844], 1600 m, 5 Oct 1982, *Méndez-T. 4746* (BIGU, MEXU); Mpio. San Cristóbal de Las Casa, Mitziton, 16.6700, -92.5325, 2430 m, 5 Aug 1993, *Ramírez-Marcial 404* (MEXU); Tak’i, 3 km al S del poblado, [16.7167, -93.1167], 4050 ft, 12 Jul 1988, *Santíz-C. 686* (MEXU, MO, TEX); Mpio. San Juan Chamula, Jipil Vinik, 6400 ft, 2 Oct 1987, *Santíz Ruíz 207* (TEX); Paraje Ya’al Ichin, 18 km al S de Chamula, [16.8400, -92.6700], 6800 ft, 21 Jul 1988, *Santíz Ruíz 955* (MO, TEX); Mpio. Tenejapa, paraje of Kulak’tik, [16.8230, -92.5092], 5800 ft, Jan-March 1964, *Shilom-T. 250* (DS); Mpio. Teopisca, 5 mi NW of Teopisca, 16°38'N, 92°32'W, 6100 ft, 20 Aug 1972, *Webster 17975* (DS, MEXU); Mpio. Pueblo Nuevo Solistahuacán, 3 km NW of Pueblo Nuevo Solistahuacán, 92°40'W, 17°30'N, 5400 ft, 19 Jul 1970, *Zuill 99* (DS); Mpio. Pueblo Nuevo Solistahuacán, 3 km NW of Pueblo Nuevo Solistahuacán, [17.1736, -92.9159], 5400 ft, 18 Sep 1970, *Zuill 362* (DS). **Guanajato**: Rancho Beltrán, 10 km al oeste de Xichu, 21.3164, -100.0987, 2000 m, 9 Dec 1990, Ventura 6473 (XAL); **Oaxaca**: Mpio. Santo Domingo Yodohino, Custeni, en orilla S de la localidad, [17.6144, -97.6797], 1645 m, 17 Jul 2003, *López-Moreno 7* (MEXU); Mt. San Felipe, Jul 1834, *Andrieux 182* (G-DC, GH, photos NY, GH, WIS); Dto. Miahuatlan, cañada de San Jeronimo Coatlan (Río Trapiches), [16.2333, -96.8667], 28 Jun 1990, *Campos-Villanueva 3215* (IBUG); Dto. Miahuatlan, cañada del Río Trapiches, lado N del poblado de San Juan Coatlan, [16.2500, -96.8833], 1500 m, 10 Aug 1990, *Campos-Villanueva 3343* (MEXU); near Oaxaca, at San Felipe del Agua, 1650 m, 1 Sep 1895, *Conzatti 132* (GH); same location, Jun 1901, *Conzatti 1217* (GH); same location, Jun 1901, *Conzatti 1218* (GH); Mpio. Huajuapan de León, ca. 5.5 rd mi NW of Huajuapan de León along hwy 190, just S of tourist turnout, SW side of the road, [17.7619, -97.7454], 1920 m, 16 Jul 1991, *Dean 229* (DAV, XAL); Mpio. Tlalixtac, along hwy 175, NE of the city of Oaxaca, ca. 4.3 rd mi E of the Juárez monument, where electrical wires cross rd, SE side of road, across river, [17.0986, -96.6454], 1740 m, 19 Jul 1991, *Dean 231* (DAV, UC, XAL); Las Animas, along hwy 175, NE of the city of Oaxaca, [17.2500, -96.5333], 2380 m, 19 Jul 1991, *Dean 232* (DAV, NY, XAL); Mpio. Calpulalpan, Calpulalpan, [17.3071, -96.4388], 2040 m, 20 Jul 1991, *Dean 233* (DAV, MEXU, MO, NY, XAL); Mpio. Tlacochahuaya, a very short distance N of turnoff to Dainzu along hwy 190, S of the city of Oaxaca, in the Oaxaca Valley, W side of road, [17.0471, -96.6715], 1585 m, 22 Jul 1991, *Dean 234* (DAV, UC, XAL); Mpio. Tamazulapan, along hwy 175 between Miahuatlán and Puerto Angel in the Sierra Madre del Sur, 0.7 rd mi NW of the town of San Andrés, ca. 12.6 rd mi SE of Miahuatlán, [16.2129, -96.5657], 2286 m, 25 Jul 1991, *Dean 237* (DAV, UC, XAL); Mpio. Matatlán, along cobblestone road to radio tower, ca. 3.6 rd mi S of town of Matatlán, S of city of Oaxaca along hwy 190, ca. 1 mi S of km 595, [16.8207, -96.3659], 1006 m, 29 Jul 1991, *Dean 241* (DAV, XAL); Mpio. Huajuapan de León, 5.5 rd mi NW of Huajuapan de León along hwy 190, just S of tourist turnoff, SW side of road, [17.8025, -97.7519], 1920 m, 9 Oct 1991, *Dean 282* (XAL); Mpio. Nochixlan, NW side of town of Nochixlan, near large drainage that crosses hwy 190, [17.4575, -97.2505], 2073 m, 9 Oct 1991, *Dean 284* (DAV, XAL); Mpio. Santa María Jaltianguis, upper areas of Sta. María Jaltianguis, along dirt road, in front of residence, [17.3631, -96.5191], 2287 m, 11 Oct 1991, *Dean 286* (DAV, XAL); Mpio. Calpulalpan, Calpulalpan, 2042 m, 12 Oct 1991, *Dean 287* (BM, DAV, F, MEXU, MO, NY, UC, XAL); Mpio. Matatlán, along cobblestone road to radio tower, about 3.6 rd mi S of Matatlán, along hwy 190 between Oaxaca City and Tehuantepec, [16.8207, -96.3659], 1006 m, 13 Oct 1991, *Dean 290* (DAV, NY, UC, XAL); Mpio. Tlacochahuaya, a very short distance N of turnoff to Dainzu, along hwy 190, between Mitla and Oaxaca City, W side of the rd, [17.0471, -96.6715], 1585 m, 14 Oct 1991, *Dean 291* (DAV, XAL); Mpio. Tamazulapan, along hwy 175 between Miahuatlán and Puerto Angel, ca. 12.6 rd mi SE of Miahuatlán, near turnoff to town of San Andrés Paxtla, [16.2197, -96.5244], 2287 m, 18 Oct 1991, *Dean 294* (DAV, MEXU, MO, NY, UC, XAL); Mpio. Sta. María Jaltianguis, Sta. María Jaltianguis, [17.3611, -96.5282], 2195 m, 20 Oct 1991, *Dean 295* (DAV, MEXU, MO, NY, UC, XAL); Oaxaca Valley, on the western foothills of Sierra Oaxacana, along hwy 175, 7.2 rd km E of hwy 190 (at Juárez Monument), 17°07'N, 96°37'W, 1799 m, 29 Jul 1978, *Diggs 2148* (WIS); along hwy 131 between Oaxaca and Puerto Escondido, 15 km by road S of Sola de Vega, 16°28'N, 97°02'W, 2012 m, 25 Jun 1986, *Diggs et al. 3997* (NY, TEX); km 7.21 carretera Ixtlán-Capulalpam por la curva de las Margaritas, 17.3166, -96.4333, 2030 m, 28 Aug 2002, *Figueroa-Brito 154a* (MEXU); cordillera de Oaxaca, 6500 ft, Nov-Apr 1840, *Galeotti 1166* (BR, G, W); San Pedro Nolasco (note by M. Nee, ca. 60 km NE of city of Oaxaca), [17.2740, -96.4208], Jul 1844, *Galeotti 1225L* (BR, NY); Mpio. Santiago Comáltepec, Santiago Comáltepec, 17°33'N, 96°31'W, 2000 m, 21 Jun 1987, *Hernández-G. 31* (MEXU, MO, NY, UC); Dto. Miahuatlan, Mpio. San Juan Mixtepec, near town, 16.3003, -96.3003, 2000 m, 8 Jun 1997, *Hunn OAX1213* (MEXU); same location, 1 Jul 1997, *Hunn 1310* (MEXU); same location, 2050 m, 27 Jul 1997, *Hunn 1640* (MEXU); Dto. Miahuatlan, E 0.5 km, 16°16.6'N, 96°17.8'W, 2000 m, 17 Jul 1998, *Hunn 1799* (MEXU); Dto. Miahuatlan, 16.2633, -96.3067, 2280 m, 21 Jul 1998, *Hunn 1832* (MEXU); San Pedro Nolasco, Talea, & c, [17.2740, -96.4208], 1843–1844, *Jurgensen 695* (BM, G); Mpio. Santiago Comáltepec, Santiago Comáltepec, 17°33'N, 96°31'W, 2000 m, 18 Jun 1988, *López-L. 132* (DAV, MEXU, NY, UC); Dto. Ixtlán, Sierra de Juárez, Rancho Vivero Teja a 3 km al N de Ixtlán, [17.3581, -96.4891], 2250 m, 28 Jul 1985, *Lorence 4603* (MEXU); region of Tamazulapan-Chilapa, on a small dirt road along a stream, 17.6056, -92.6408, 1740 m, 3 Jul 2014, *Lott 6032* (DAV); Mpio. Santiago Laxopa, Santiago Laxopa, 17°13'N, 96°18'W, 2000 m, 26 May 1986, *Maldonado-V. 19* (DAV, MEXU, UC); 15 km (by road) SE of Miahuatlán on road to Puerto Angel in high mountains of Sierra Madre de Sur, 16°12'N, 96°30'W, 2400 m, 6 Jul 1969, *Marcks 1031* (MO, WIS); Mpio. Santa Catarina Ixtepji, a 1 km al E de Corral de Piedra, camino a La Cumbre, 17.1747, -96.6492, 3184 m, 5 Jun 2001, *Martínez S. 34157* (MEXU); Dto. Ixtlán, Cañada del Estudiante, [17.2049, -96.5958], Jul 2001, Martínez 302 (MEXU); Mpio. San Juan Mixtepec, Ladera del Río Azucena, a 17 km al noroeste de San Juan Mixtepec, 17.3667, -97.8333, 1800 m, 22 Jul 1989, *Reyes-Santiago 1736* (MEXU); Dto. Teposcolula, Rancho El Colibri, a 2 km al noroeste de San Pedro Teposcolula, [17.5333, -97.5500], 2210 m, 19 Oct 1990, *Reyes-Santiago* 2442 (MEXU); E slope of El Cumbre Mtns, 16°55'N, 96°20'W, 21 Jul 1966, *Schoenwetter JSOX25* (US); Tenango, 5 Jul 1895, *Smith 400* (GH); along road to microwave tower, ca. 3.6 mi S of Matatlán on hwy 190, about 1 mi S of km 595, [16.8207, -96.3659], 22 Jul 1971, *Stevens 1297* (ENCB, MO, WIS); Dto. Ixtlán, Mpio. Ixtepeji, a 9 km al N de Cerezal, 17.2616, -96.5687, 1900 m, 26 Sep 1982, *Téllez-V. 6050* (MEXU); Dto. Ixtlán, Rancho Arriero, recorrido de Santa María Totomoxtla, 17.6050, -96.5833, 1600 m, 25 Jun 2002, *Torres C. 16667* (MEXU); **Puebla**: cerros calizos al NE de Tehuacán, vecinos al campo tiro del ejercito, 2 Jul 1981, *Chiang C. F2031a* (MEXU); Mpio. Tehuacán, Barranca Cruz de Quiote, in the hill to the NE of city of Tehuacán, NE of the army rifle range and cemetery, [18.4667, -97.3500], 1768–1800 m, 9 Jul 1990, *Dean 206* (DAV, IEB, MEXU); Mpio. Tehuacán, El Riego Mesa on the W side of the city of Tehuacán [18.4391, -97.4224], 1750 m, 9 Jul 1990, *Dean 207* (DAV, MEXU); Mpio. Caltepec, 0.85 road km E of junction of Atecoxco-San Pedro Atzumba road with Acatepec-Caltepec road, along road to Caltepec, fields on S side of road, [18.2269, -97.5734], 2165 m, 10 Jul 1990, *Dean 208* (DAV, MEXU); Mpio. Tehuacán, E [W] side of city of Tehuacán, just W[E] and SW [SE] of El Riego mesa, below El Riego cave and by road leading up to hills, [18.4391, -97.4224], 1677 m, 13 Jul 1991, *Dean 225* (DAV, MEXU, NY, UC, XAL); Mpio. Caltepec, between Cerro Pochote and Cerro Gavilán Chico in hills SE of town of Caltepec, along road to Atolotitlan, [18.1667, -97.4667], 2073–2104 m, 15 Jul 1991, *Dean 227* (DAV, XAL); Mpio. Tehuacán, old field just to the E of the mesa at El Riego, and drainage that crosses dirt road to the SE of El Riego, [18.4431, -97.4252], 1677 m, 27 Sep 1991, *Dean 271* (DAV, MEXU, NY, UC, XAL); Mpio. Tehuacán, hills to the SW of the city of Tehuacán, up dirt road near El Riego, [18.4433, -97.4235], 1677–1982 m, 27 Sep 1991, *Dean 272* (DAV, XAL); Mpio. Caltepec, 0.85 km E of junction of Acatepec-Caltepec and Atecoxco-San Pedro Atzumba roads, along road to Caltepec, [18.2269, -97.5734], 2164 m, 30 Sep 1991, *Dean 275* (DAV, NY, XAL); Mpio. Caltepec, hills SE of the town of Caltepec, between Cerro Gavilán Chico and Cerro Pochote, as well as an area called Rancho Gavilán, near rd to Atolotitlan, [18.4500, -97.5167], 2225 m, 30 Sep 1991, *Dean 277* (DAV, UC, XAL); Mpio. Zapotitlán de las Salinas, San Antonio Texcala, along hwy 125 S of Tehuacán, canyon with onyx mine just N of town, [18.3833, -97.4333], 1677 m, 22 Oct 1991, *Dean 299* (DAV, XAL); 3 mi N of the city limits of Tehuacán, [18.5105, -97.4068], 5500 ft, 7 Jun 1973, *Hansen. 1722* (MEXU, WIS); Chila Zapotitlán [perhaps between Chila and Zapotitlán], [18.3167, -97.4000], 15 Jul 1943, *Miranda 2845* (MEXU); Tehuacán, [18.4500, -97.3333], 5000 ft, 6 Aug 1897, *Pringle 6776* (BM, BR, G, GH, GOET, HBG, JE, LL, M, MEXU, MO, NY, S, TEX, UC, W, WU, Z); El Riego, [18.4530, -97.4258], Jun 1905, *Purpus 1279* (MO, UC); in the vicinity of San Luis Tultitlanapa, near Oaxaca, [18.0500, -97.4833], Jul 1908, *Purpus 3561* (MO, UC); same location and date, *Purpus 3562* (BM, UC); W of Tehuacán on the Mesa above El Riego, [18.4433, -97.4317], 1500 m, Jul 1961, *Smith et al. 3804* (F, G, MEXU, NY); Mpio. Atzingo, Tlacuilosto al S de Atzingo, [19.9667, -97.9667], 1900 m, 22 Jul 1985, *Tenorio-L. 9464* (TEX); 9.6 km al NW de Huajuapan de León, carr. a Acatlán, antes de la torre de microondas, [17.8811, -97.8343], 1770 m, 29 Jul 1983, *Torres-C. 3321* (MEXU, MO). **San Luis Potosí**: Mpio. Zaragoza, ca. 9.54 rd mi W of outskirts of town of Santa Catarina along hwy 70 (Río Verde-San Luis Potosí hwy), [22.0993, -100.6739], 5500–5700 ft, 3 Jul 1991, *Dean 222* (DAV, XAL); Mpio. Zaragoza, ca. 1.6 rd mi E of little town of San Francisco, along hwy 70 (Río Verde-San Luis Potosí hwy), W of Santa Catarina, large turnout at site of small shrine, [22.0539, -100.5744], 2043–2058 m, 3 Jul 1991, *Dean 223* (DAV, NY, XAL); Mpio. Zaragoza, 6.1 rd mi E of turn off to Sierra de Alvarez at town of San Francisco, along hwy 70 between city of San Luis Potosí and Río Verde, [22.0443, -100.5181], 1680 m, 15 Sep 1991, *Dean 251* (DAV, MEXU, NY, XAL); El Leoncito, km 207 de la carretera entre la ciudad de San Luis Potosí y Río Verde, 22.5167, -100.5000, 1520 m, 4 Aug 1995, *Rodríguez C. 2721* (IEB, MEXU); 56 km E of San Luis Potosí or 18 km W of Santa Catarina on Hwy 86, 22°05'N, 100°35'W, 1100 m, 10 Jul 1965, *Roe 75* (ENCB, WIS); Los Aguajitos, 11 km la NE de Guadalcázar, hacia Pozo de Acuña, 22.6250, -100.3167, 2000 m, 17 Nov 1996, *Torres C.* 14874 (MEXU); Aguaje de Los García, por la Cueva del Gato, 22.6089, -100.4456, 1750 m, 18 Jul 1998, *Torres C. 15243* (MEXU); Cerro El Calvario, cerros al suroeste de Guadalcazar al final de la calle Ocampo, 22.6000, -100.3833, 1068 m, 31 Jul 2000, *Torres C. 15858* (MEXU); Curva del Gato, 6 km al noroeste de Guadalcazar hacia crucero carreterea San Luis Potosí – Matehuala, 22.6167, -100.4333, 1686 m, 15 Oct 2000, *Torres C.* 15975 (MEXU).

**12. *Lycianthes connata***

**Guatemala. El Progreso**: on top of Montaña Piamonte, along Joya Pacayal, 3000 m, 7 Feb 1942, *Steyermark 43677* (NY); **Huehuetenango**: Sierra de los Cuchumatanes, Cruz de Limón, between San Mateo Ixtatán and Nuca, 2600–3000 m, 31 Jul 1942, *Steyermark 49828* (G); **Zacapa**: Mpio. Río Hondo, 1.5 horas N Finca Alejandra, 30 minutes S Cerro Paloma (P26-Proyecto Deslaves), 15.1569, -89.6178, 2512 m, 17 Mar 2012, *Cifuentes 435* (BIGU65648).

**Mexico. Chiapas**: Mpio. Tenejapa, on the NE side of the hill called Matsab. Paraje of Matsab, [16.7443, -92.4905], 9200 ft, 25 Aug 1966, *Breedlove 15296* (DUKE); Mpio. Jitotol, about 12 km N of Jitotol along a side road to an oil well, [17.1417, -92.8842], 2000 m, 28 Sep 1971, *Breedlove 19947* (MEXU, MO); Mpio. Pueblo Nuevo Solistahuacán, crest of ridge, watershed preserve for Clinica Yerba Buena, [17.1797, -92.9047], 2000 m, 16 Dec 1971, *Breedlove 23209* (MO); same location and elevation, 5 Dec 1972, *Breedlove 29964* (MEXU); Mpio. Tenejapa, near Colonia Ach’lum, [16.7753, -92.4467], 2700 m, 10 Feb 1981, *Breedlove 49775* (MEXU, MO, NY); Mpio. Tenejapa, near Paraje Banabil, 2713 m, 8 Oct 1981, *Breedlove 53374* (MO, NY); Mpio. Tenejapa, Paraje Banabil, 2590 m, 14 Nov 1981, *Breedlove 55552* (MO, NY); Mpio. Tenejapa, near Paraje Banabil, 2680 m, 12 Jan 1982, *Breedlove 57055* (MEXU, MO); a 25 km al NE de San Cristóbal de las Casas sobre el camino a Matzala, [16.8228, -92.4986], 25 Nov 1982, *Cabrera 3811* (MO); Mpio. Pueblo Nuevo Solistahuacán, Reserva Ecologica de la Yerbabuena, 3 km pasando Pueblo Nuevo Solistahuacán por carretera Pichucalco, [17.1856, -92.9050], 2100 m, 03 Jan 1988, *Chazaro-Basañez 5337* (WIS, XAL); Mpio. Tenejapa, along the road to the town of Matzam, ca,.1.5 km from the eastern outskirts of the town of Las Ollas where the road forks, about 2.6 km from the intersection with the San Cristóbal de las Casas-Tenejapa road, just west and upslope of the settlement of Paraje Cruz Tzibaltic, on ridge where there is an intersection with an undeveloped road, 16.7832, -92.5275, 2484 m, 13 Sep 2017, *Dean 9530* (DAV); Mpio. Chamula, Tzontehuitz, 16.6856, -92.5714, 2897 28 Aug 1999, *Domínguez-Torres 105* (MEXU); al NE de Pueblo Nuevo Solistahuacán, [17.1647, -92.8809], 2050 11 Aug 1967, *Gómez-Pompa 2532* (MEXU); Mpio. Huixtan, Rancho Merce-Bazom, [16.7347, -92.4864], 2450 m, 29 Oct 1988, *González-Espinosa 573* (MEXU, XAL); Mpio. Pueblo Nuevo Solistahuacán, ca. 3 km sureste Selva Negra, [17.1941, -92.9903], 28 Mar 1952, *Harrel 437* (MEXU); Mpio. Pueblo Nuevo Solistahuacán, Clinica La Yerbabuena, [17.1796, -92.9042], 2100 m, 23–26 Oct 1989, *Heath AM28* (MEXU880470); Mpio. Jaltenango, Reserva El Triunfo, poligono 1, Cerro El Triunfo, 15.6500, -92.8000, 2200 m, 12 Jun 1990, *Heath 955* (MEXU); Mpio. Pueblo Nuevo Solistahuacán, Clinica La Yerbabuena, 17.1833, -92.9000, 2100 m, 23 Oct 1989, *Heath 2028* (MEXU); 22 km from San Cristóbal de las Casas on the road to Tenejapa, then right 3 km on the road to Matzam, [16.7988, -92.4728], 2400 m, 29 Sep 1984, *Huft 2212* (MEXU); Mpio. Santa Rosa, near Escuintla, [15.4472, -92.5339], 1600 m, 20 Jun 1941, *Matuda 4249* (A, MEXU, NY); Mpio. Siltepec, Fraylesca, [15.5508, -92.4042], 2000 m, 13 Mar 1945, *Matuda 5256* (MEXU); Mpio. Pueblo Nuevo Solistahuacán, La Yerbabuena, [17.1827, -92.9064], 2000 m, 15 Nov 1988, *Palacios-Espinosa 1103* (MEXU); Mpio. Angel Albino Corzo, Reserva de la Biosfera El Triunfo, [15.6529, -92.8096], 2000 m, 17 Jun 1994, *Ramírez-Marcial 511* (MEXU); Mpio. Pueblo Nuevo Solistahuacán, Reserva Natural Yerbabuena, frente a la clinica Yerbabuena, 2 km al noroeste de Pueblo Nuevo Solistahuacán, [17.1856, -92.9050], 1850 2150 m, 15 Feb 1990, *Reyes-García 1665* (MEXU, MO, XAL); Mpio. Pueblo Nuevo Solistahuacán, 3 km N of Pueblo Nuevo, near Clinica Yerba Buena, [17.1797, -92.9047], 2420 m, 12 Aug 1965, *Roe 1222* (WIS); a 4 km al S de Matsab, [16.7443, -92.4905], 8 Sep 1981, *Téllez-Valdes 4670* (MEXU); Jitotol Ridge, c. 3 km N of Pueblo Nuevo Solistahuacán, 17.1333, -92.8833, 6600–6700, 9 Aug 1972, *Webster 17778* (MEXU); **Oaxaca**: San Pedro Yólox, Dto. de Ixtlán, 9.3 km N of the ‘desviacion’ to Yólox, [17.5989, -96.5479], 2400 m, 7 Apr 1981, *Martin 488* (MEXU, MO, NY, US); Dto. Mixe, Mpio. Totontepec Villa de Morelos, Kets tekum, tonun Kux, [17.2523, -96.0292], 17 Jul 1994, *Rivera-Reyes 3156* (IEB, MEXU); Mpio. San Felipe Usila, Cuenca del Río Perfume (ladera O), 6.7 km en línea recta al S de Santa Cruz Tepetoutla, 17.6769, -96.5542, 2120 m, 3 Apr 1994, *Rincon-G. 351* (IEB, MEXU, XAL); Dto. Mixe, Mpio. Totontepec Villa de Morelos, Totontepec, 17.2500, -96.0000, 1900 m, 8 Sep 1986, *Rivera-Reyes 452* (MEXU, NY); Mpio. Totontepec Villa de Morelos, Kets tekum, na payi, 17 Jul 1994, *Rivera-Reyes 3143* (IEB, IEB); Dto. Mixe, Mpio. Totontepec Villa de Morelos, 9.4 km al S de Tototepec, [17.2357, -96.0213], 2170 m, 24 Feb 1987, *Torres-C. 9333* (MEXU).

**13. *Lycianthes cuchumatanensis***

**Guatemala. Alta Verapaz**: Mpio. San Juan Chamelco, Chicacnab. La Laguna, 15.3844, -90.1639, 2300 m, 4 Aug 1998, *Robles 124* (MSB); **Huehuetenango**: Cerro Huitz between Mimanhuitz and Yulhuitz, Sierra de los Cuchumatanes, [15.8550, -91.3244] 1500–2600 m, 14 Jul 1942, *Steyermark 48617* (G); same location and date, *Steyermark 48625* (F); same location and date, *Steyermark 48645* (A).

**14. *Lycianthes dejecta***

**Mexico. State Unknown**: no exact location, *Schmitz s.n*. (W) and *482* (BM, W); **Baja California Sur**: Mpio. La Paz, El Paraje de Cano, Sierra de la Victoria, 23.5833, -109.9167, 1670 m, 30 Sep 1994, *Domínquez-L. 800* (HCIB); **Dto. Federal**: Sierra de Guadalupe, [19.5907, -99.1203], 7000 ft, 21 Jul 1938, *Balls B5073* (BM, K, UC); **Guanajuato**: Mpio. San José de Iturbide, cañada a 5 km de Santa Anita, cerro La Meza, 20.9667, -100.3000, 2300 m, 22 Sep 2002, *Cruz 1229* (MEXU), Mpio. San Luis de la Paz, Mesas de Pueblo, along road NW of town, [21.4139, -100.5840], 2348 m, 4 Jul 1991, *Dean 224* (DAV, XAL); Mpio. San José Iturbide, near Rancho El Guajolote, SW of San José Iturbide, one hwy exit S of exit to San José, on W side of hwy 57, take dirt rd E and cross river, [20.9031, -100.4153], 1829 m, 16 Sep 1991, *Dean 253* (DAV, MEXU, NY, UC, XAL); same location, 31 Oct 1991, *Dean 309* (DAV, XAL); hwy 51 near San Miguel de Allende, [20.8304, -100.7850], 2200 m, 7 Jul 1971, *Genelle 922* (MO, WIS); cerca de San Miguel Allende, Río Laja, Atotonilco, [20.9988, -100.8055], 1850 m, 8 Aug 1980, *Kishler 923* (MEXU); Mpio. Guanajato, cañada de la Virgen, [21.0000, -101.2500], 2000 m, 24 Aug 1994, *Mares 5* (MEXU); Mpio. Apaseo el Alto, Camino a Talayote, 20.4015, -100.6360, 1990 m, 14 Oct 1996, *Martínez 6103* (IEB, MEXU); Mpio. San Luis de la Paz, Predio El Cortijo, a 16 km al NE de la ciudad de Dolores Hidalgo sobre la carretera a San Luis de la Paz, 21.2189, -100.7983, 1906 m, 17 Aug 1996, *Ocampo 47* (IEB, MEXU, TEX); San José Iturbide, cerca de el Guajolote, 2200 m, 22 Aug 1988, *Rzedowski 47099* (IEB); Mpio. San José de Iturbide, El Zorrillo, 20.9706, -100.2739, 2277 m, 4 Jul 2003, *Monroy 27* (MEXU); Rancho La Misión, 8 km al NE de San Luis de la Paz, [21.3386, -100.4654], 2900 m, 11 Oct 1988, *Ventura 6129* (IEB, MEXU, XAL); Mesas del Pueblo, 8 km al N de San Luis de la Paz, [21.3554, -100.3435], 17 Jul 1989, *Ventura 6871* (IEB, MEXU, XAL); La Mina Grande de Pozos, [21.2217, -100.4830], 2100 m, 17 Jul 1990, Ventura 8291 (MEXU, MO); Mpio. San Luis de la Paz, Santa Brigada, camina a Pozo, [21.2667, -100.5000], 2100 m, 18 Jul 1991, Ventura 9284 (MEXU); Mpio. Victoria, Cerro del Xoconoshtle, frente al Nogal, [21.2045, -100.0950], 2150 m, 28 Jun 1986, *Zamudio-Ruiz 4007* (IEB, MEXU, XAL); **Hidalgo**: Mpio. Tepealpulco, outskirts of Tepealpulco, base of Cerro Tres Peñas, near ruin of Jihuingo and rock quarry, [19.8167, -98.5333], 2530 m, 21 Sep 1991, *Dean 261* (DAV, MEXU, UC, XAL); Rincon del Gato, barranca al N del poblado Emiliano Zapata, Sierra de Chicavasco, ejido Emiliano Zapata, 20.1583, -99.0250, 2280 m, 20 Aug 1988, *Diaz-Vilchis 27* (MEXU); 11 mi up mine rd W of Mex 85, 2 mi N of Posada del Rey, Zimapán, on ridge-like plateau, [20.7801, -99.3303], 5 Jul 1966, *Mears 254a* (TEX); trail from Zimapán to mines of El Monte, N of Zimapán, [20.7500, -99.3833], 7500–7800 ft, 11 Aug 1948, *Moore 4460* (A, GH); Mpio. Tula de Allende, Cañón de las Ajuntas, Santa María Macua, 20.1125, -99.4625, 2150 m, 15 Jun 2003, *Romero 75* (MEXU); prope Zimapán, Jun 1828–1833, *Schiede s.n.* (HAL); Mpio. Tepeapulco, Cerro Tres Peñas, [19.8121, -98.5499], 2500 m, 9 Sep 1975, *Ventura-A. 248* (CAS, F, IEB, MEXU, XAL); Mpio. Tepealpulco, terrenos de Tepeapulco, 2600 m, 14 Oct 1975, *Ventura-A. 402* (F, MEXU); Mpio. Zempoala, Tlaquilpan, [19.9362, -98.7546], 2500 m, 22 Jun 1976, *Ventura-A. 1650* (F, IEB, MEXU); **Jalisco**: Mpio. Lagos de Moreno, carretera Lagos de Moreno-León, km 31, [21.1739, -101.7238], 2020 m, 15 Jul 1991, *Arreola-Navas 1270a* (MEXU); Matanzas, along road from Ojuelos de Jalisco, 21.85000, -101.583, *Rodríguez-C. s.n.* (IBUG); **México**: Valley of Mexico, fields, near Guadalupe, Mt. Zacualco, 10 Jul 1865, *Bourgeau 543* (BR, F, G, M) (Syntype of *Solanum dejectum*, cited by Fernald – but as *Hahn 543*); Actopan [near Temascalapa], on high plateau by city of Mexico, [19.8362, -98.8552], June 1827, *Karwinski s.n.* (M); Mpio. Huehuetoca, N of Huehuetoca along the road to Apaxco, ca. 4.2 road mi from building “Los Arcos” in downtown Huehuetoca, W side of rd, [19.8894, -99.2141], 2200 m, 1 Jul 1990, *Dean 202* (BM, DAV, MEXU, MO, NY, UC, XAL); Mpio. Huehuetoca, N of town of Huehuetoca along the rd to Apaxco, ca. 4.2 rd mi from building “Los Arcos” (in downtown Huehuetoca), W side of rd, [19.8894, -99.2141], 2165 m, 3 Aug 1991, *Dean 243* (DAV, MEXU, UC, XAL); same location, 29 Oct 1991, *Dean 304* (DAV, MEXU, XAL); Valley of Mexico, ca. San Cristóbal, [19.5868, -99.086], 2800 m, 25 May 1951, *Matuda 21145* (MEXU); Mpio. Ecatepec, Rancho el Copal, cerca de San Juan Ixhuatepec, [19.5273, -99.1089], 2400 m, 14 Sep 1982, *Rzedowski 37903* (ENCB); Lomas de Jaral [perhaps El Jaral in Atizapan, but not georeferenced], 18 Jul 1886, *Schumann 998* (JE); **Michoacán**: Morelia, Punguato, [19.6975, -101.1334], Aug 1910, *Arsène s.n.* (F); same location, 16 Aug 1909, *Arsène s.n.* (F); same location, 1950 m, 16 May 1909, *Arsène s.n.* (G); same location, 2000 m, 26 Jun 1909, *Arsène 2714* (GH, L, MEXU, MO); same location, 2200 m, 11 Aug 1910, *Arsène 5233* (CAS, GH, MO); same location, 2100 m, 20 Jun 1912, *Arsène 8300* (F, GH, MO, NY); **Nuevo León**: Mpio. Galeana, Cerro El Gallo, [24.9200, -99.7800], 2085 m, 15 Jun 1991, *Hinton 21019* (GH, IEB, TEX); **Oaxaca**: Dto. Cuicatlan, cañada al SW de la estación de ferrocarril El Venado, 17.5842, -97.0083, 1500 m, 11 Jun 2002, *Medina-Lemos 1084* (MEXU); a las afueras de Guadalupe Membrillos, 18.0228, -97.5508, 2276 m, 12 Aug 2004, *Téllez-V. 17009* (MEXU); **Puebla**: Mpio. Caltepec, between Cerro Pochote and Cerro Gavilán Chico in hills SE of town of Caltepec, along road to Atolotitlan, near small valley called La Laguna, [18.1784, -97.4698], 2073–2104 m, 15 Jul 1991, *Dean 228* (DAV, MEXU, XAL); Mpio. Caltepec, 0.85 km E of junction of Acatepec-Caltepec and Atecoxco-San Pedro Atzumba roads, along road to Caltepec, [18.2018, -97.5409], 2164 m, 29 Sep 1991, *Dean 274* (DAV, MEXU, UC, XAL); Mpio. Caltepec, hills SE of the town of Caltepec, between Cerro Gavilán Chico and Cerro Pochote, as well as an area called La Laguna, near rd to Atolotitlan, 2073–2104 m, 30 Sep 1991, *Dean 276* (DAV, MEXU, XAL); Mpio. Caltepec, Barranca El Tocotín al E de Caltepec, [18.1828, -97.4787], 2020 m, 5 Jul 1983, *Tenorio-L. 3990* (MEXU); **Querétaro**: Cadereyta, [20.6833, -99.8167], 2000 m, 15 Jun 1975, *Arguelles 73* (MEXU); camino a Huimilpán, km 11, cerca de barranca, [20.5062, -100.3253], 2297 m, 27 Jul 1984, *Arguelles 2147* (MEXU); camino entre Huimilpán-Querétero y carr. Mexico-Los Cues, km 6, [20.5061, -100.2862], 2020 m, 29 Jun 1986, *Arguelles 2538* (MEXU); km 12 camino a Huimilpán, lado del camino, entrando un poco, lado izquierdo, [20.5040, -100.3243], 2100 m, 12 Jul 1987, *Arguelles 2985* (IEB, MEXU); Mpio. Cadereyta, camino a Tovares, junto al Jardin Botanico, [20.6885, -99.8068], 2050 m, 5 Jul 2007, *Chavez 122* (MEXU); Mpio. Huimilpán, 0.5 road km S of marker for km 11 along the road between city of Querétaro and Huimilpán, N side of road leading down to upper reaches of barranca, [20.5029, -100.3250], 2297 m, 2 Jul 1990, *Dean 203* (BM, DAV, F, MEXU, MO, NY, UC, XAL); Mpio. Huimilpán, along rd to Los Cues, 6 rd km E of intersection with Querétaro-Huimilpán road, just NE of 3 km marker, [20.4961, -100.3039], 2297 m, 2 Jul 1990, *Dean 204* (DAV, MEXU, NY, XAL); same location, 2020 m, 16 Sept 1991, *Dean 255* (DAV, UC, XAL); same location and elevation, 29 Oct 1991, *Dean 305* (DAV, MEXU, NY, UC, XAL); Mpio. Cadereyta, along rd between Cadereyta and Lizarro, by town of Charco Frío, [20.7905, -99.7262], 2226 m, 30 Oct 1991, *Dean 307* (DAV, MEXU, MO, NY, UC, XAL); Mpio. Querétaro, Cerro El Cimatario, [20.5083, -100.5050], 2110 m, 27 Jun 2001, *Pantoja 169* (IEB); San Juan del Río, [20.3878, -99.9960], 14 Jul 1896, Pringle 7202 (MO); Mpio. Ezequiel Montes, 2.09 km al NO de Bernal, [20.7508, -99.9572], 2240 m, 21 Sep 2012, *Rubio-García 263* (IEB); Mpio. Ezequiel Montes, 4.2 km al ONO de la Florida, [20.7717, -99.8592], 2117 m, 28 Jun 2013, *Rubio-García 640* (IEB); **San Luis Potos**í: Santa María del Río, 23 Jun 1959, *Rzedowski 3251* (ENCB, WIS).

**15. *Lycianthes fredyclaudiae***

**Guatemala. Baja Verapaz**: along Hwy 3 to Cobán, 15 km by rd. S-SW of Puruhlá, [15.1752, -90.2449], 1800 m, 16 Jul 1976, *Breckon 2148* (DAV, F, MO); Unión Barrios, [15.1820, -90.1943], 28 Feb 1972, *Contreras 11080* (CAS, F, LL, MO); Unión Barrios, [15.1820, -90.1943], 12 Mar 1972, *Contreras 11260* (CAS, F, LL, MO); en aldea Unión Barrios, km 150 de la carretera que conduce a Cobán A.V. en el lado oeste de la aldea en la parte media de un cerro, [15.1825, -90.2147], 7 Nov 1973, *Contreras 11601* (MEXU, TEX); on Hwy CA 14 to Cobán, 3 miles south of Purulhá, [15.2156, -90.2134], 1500 m, 16 Jul 1977, *Croat 41222* (MO); Mpio. Purulhá, along Hwy CA 14 between El Progreso and Cobán, 3 mi S of Purulhá, 17 mi N of junction with Hwy 17 to Salamá and San Jeronimo vic. km marker 160, 15.2172, -90.2108, 1620–1720 m, 26 Jan 1987, *Croat 63719* (DAV, MO); along Cobán-Guatemala Hwy 14 between Unión Barrios and Niños Perdidos. west side of the road, 15.15616, -90.1841, 1558 m, 9 Aug 2017, *Dean 9506* (DAV); Along Guatemala Highway 14 just south of highway marker 158 and La Ram Tzul nature reserve, west side of the road, 15.20637, -90.20648, 1610 m, 11 Aug 2017, *Dean 9508* (BIGU, DAV); Unión Barrios, in high forest, top of hill, E of km 154, [15.1903, -90.17608], 7 Jun 1975, *Lundell 19388* (CAS, F, MEXU, LL, MO); Niño Perdido, on Cerro Verde, [15.1607, -90.1651], 12 Jun 1977, *Lundell 21085* (CAS, F, MEXU, MO, TEX); a 4 km al S de Purulhá, camino Guatemala-Cobán, cerca Biotopo, [15.2114, -90.2063], 1660 m, 15 Jun 1985, *Martínez-S. 13132* (MEXU, NY); Biotopo del Quetzal, [15.2138, -90.2189], 1630 m, 22 Jul 1988, *Martínez-S. 23082* (DAV, MEXU, MO, NY); Mpio. Purulhá, Biotopo del Quetzal, [15.2138, -90.2189], 1590 m, 5 Sep 1988, *Martínez-S. 23514* (MEXU, MO); mountain of Purulhá between La Unión and Purulhá, [15.2103, -90.2073], 1600 m, 1 Oct 1972, *Molina-R. 27743* (F, MEXU, TEX); Sierra de las Minas, about 5 km S of Purulhá [15.1885, -90.2353], 1600 m, 2 Jan 1973, *Williams 41952* (F, NY); Sierra de la Minas, 3 km SE of Purulhá, [15.2146, -90.2188], 1800 m, 2 Jan 1974, *Williams 43119* (F); about three miles N of Chilascó in mountains E of Salamá and San Gerónimo, [15.1624, -90.09657], 23 May 1971, *Wilbur 14788* (DUKE).

**16. *Lycianthes geminiflora***

**Mexico. Hildalgo**: Mpio. Tianguistengo. Loc. 10 km al E de Tianguistengo, [20.7265, -98.5279], 6 Jul 1995, *Sousa-Peña 594* (IEB, MEXU); **Oaxaca**: Dto. Ixtlán de Juárez, camino en construccion Tiltepec-La Luz km 7–4, 17.4986, -96.3542, 1790 m, 06 Aug 1997, *Arellanes 72* (MEXU1024661); Dto. Ixtlán, Mpio. Comaltepec, SW of Cerro Relampago, just above (to N of) Río Soyalapan near Federal Electricity Commission Camp, down trail that descends from Hwy 175, 17.4875, -96.4014, 1750 1780 m, 3 Dec 1993, *Boyle 2631* (MEXU); Valle National, senda de San Antonio Ocote a Cerro Cuate, 23 Sep 1990, *Calzada 16427* (MEXU); Mpio. San Felipe Usila, al SW de Cerro Verde, [17.9041, -96.5478], 1140 m, 25 May 1991, *Calzada 16982* (MEXU); Dto. Ixtlán, between km 84 and 85 on hwy 175 SW of Tuxtepec, 71 km NE of Ixtlán, [17.6733, -95.3293], 1 Nov 1996, *Daniel 8360* (MEXU); Sierra de Juárez, Mpio. Comaltepec, along Hwy 175 to the NE of the turnoff to Comaltepec, and NE of the cabins and restaurant of Mirador, trail on the SE side of the road called Sendero Relampago, 17.5918, -96.3999, 2080 m, 10 Sep 2017, *Dean 9521* (DAV); Sierra de Juárez, Mpio. Comaltepec. along Hwy 175 to the NE of the turnoff to Comaltepec, and NE of the cabins and restaurant of Mirador, SW of turnoff to Yólox, near gate to Sendero La Capilla between km 96 and 97, 17.5871, -96.4486, 2158 m, 10 Sep 2017, *Dean 9523* (DAV); Sierra de Juárez, Mpio. Comaltepec, along Hwy 175 between km 66 and 67 just south of the town of Metates, 17.6860, -96.3289, 870 m, 11 Sep 2017, *Dean 9525* (DAV); Chinantla, Jul 1844, *Galeotti 1225S* (W, photo MO); Mpio. Comaltepec, alrededores de la carretera federal Tuxtepec-Oaxaca, 2.8 km en línea recta al suroeste de La Esperanza, 17.6089, -96.4039, 1600 m, 10 Jan 1995, *Gallardo-Hernandez 1309* (MEXU, XAL); Mpio. Ixtlán de Juárez, camino Tiltepec – Josaa 1 km, 17.5192, -96.3217, 1890 m, 9 Jun 1997, *J. García R. 146* (MEXU); Mpio. Ixtlán de Juárez, Tiltepec, 17.5133, -96.3247, 1380 m, 2 Aug 1999, *García-R. 386* (MEXU); Mpio. Tuxtepec, 2 km al S de La Esperanza, carretera Oaxaca-Tuxtepec, 1680 m, 3 Aug 1981, *García-M. 676A* (MEXU, MO, NY); Mpio. San José Tenango, Agua Golondrina, Sierra Mazateca, [18.1964, -96.6917], 800 m, 6 Jun 2005, *Giovannini 83* (MEXU1270738); same location and elevation, 12 Oct 2005, *Giovannini 236A* (MEXU1268369); 3 m N of Comaltepec exit on Hwy 172, [17.5719, -96.5424], 3200 m, 3 Jul 1981, *Hahn 653* (MO3661203); Mpio. Comaltepec, La Esperanza, 17.6167, -96.3667, 1600 m, 27 Nov 1987, *Hernandez-G. 153* (MO, NY); Valle Nacional, alrededores de Cerro Mirador, 15 km en línea recta al nor-noroeste de Valle Nacional, 17.9108, -96.3942, 1100 m, 27 Apr 1993, *Ibarra-Manriquez 3830* (IEB); Mpio. Comaltepec, La Esperanza, 17.7500, -96.5000, 1600 m, 8 Dec 1987, *López-Luna 146* (MEXU, NY); Mpio. Comaltepec, La Esperanza, [17.6167, -96.3500], 1600 m, 20 Sep 1988, *López-Luna 367* (MEXU, NY); same location, 13 Jul 1989, *López-Luna 482* (MEXU, NY); entre Vista Hermosa y Comaltepec, km 95 carr. Tuxtepec-Oaxaca, Sierra Juárez, 1000 m, 6 Sep 1965, *Martínez-C. 269* (BRIT); Mpio. Comaltepec, Vista Hermosa, Sierra de Juárez, [17.6322, -96.3425], 1500 m, 12 May 1966, *Martínez-C. 806* (MO, XAL); Dto. Ixtlán, a 31 km al S de Valle Nacional, camino a Oaxaca, [17.5759, -96.4622], 22 Nov 1984, *Martínez-S. 8787* (MO, NY); Mpio. San Felipe Usila, Cuenca del Río Perfume (ladera oeste), 7 km en línea recta al S de Santa Cruz Tepototula, 17.6778, -96.5442, 1830 m, 19 May 1994, *Meave 1629* (IEB, MEXU); entre Vista Hermosa y Comaltepec, km 82 carr. Tuxtepec-Oaxaca, Sierra Juárez, [17.5948, -96.4994], 31 Aug 1965, *Ortíz-C. 241* (MEXU); Mpio. San Felipe Usila, alrededores del Río Tlacuache, 2.2 km en línea recta al SO de Santa Cruz Tepetotutla, 17.7247, -96.5728, 1050 m, 7 May 1994, *Osorio-H. 114* (MEXU); Mpio. San Felipe Usila, Cuenca del Río Perfume (ladera E), 5 km en línea recta al SE de Santa Cruz Tepetotutla, 17.7042, -96.5303, 1160 m, 2 Apr 1996, *Rincón-G. 780* (MEXU); Huautla, 2 km SW of market, 18.1333, -96.8667, 1400 m, 15 Jan 1984, *Solheim 1354* (NY); 3 km sureste de la Esperanza, vereda a Tarabundi, 17.6106, -96.0125, 1200 m, 10 Sep 1993, *Torres 35A* (XAL); Mpio. Comaltepec, 9 km sur-suroeste de La Esperanza, vereda Cerro Relampago, 17.5861, -96.3842, 2000 m, 11 Sep 1993, *Torres 53* (MEXU); Dto. Ixtlán, 24.8 km al N de Humo Chico, carr. Oaxaca-Tuxtepec, [17.8129, -96.1651], 27 Sep 1982, *Torres-C. 1434* (MEXU, NY); Dto. Tehuantepec, 22 km al NW de La Chiviza, carr. a Santiago Lachiguiri, 1160 m, 24 Aug 1984, *Torres-C. 5909* (MEXU); Mpio. Comaltepec, 3 km al S de Metates, carretera Tuxtepec-Oaxaca, [17.5519, -96.5340], 10 Sep 1985, *Torres-C. 7271* (MEXU, XAL); Dto. Tuxtepec, 4 km al SE de Metates, bajada a la casita carr. Tuxtepec-Oax, [17.6678, -96.3270], 9 Dec 1985, *Torres-C. 7820* (MEXU, XAL); Mpio. Comaltepec, 1.4 km al S de Metates, carr. Tuxtepec-Oaxaca, [17.6817, -96.3290], 820 m, 26 Aug 1986, *Torres-C. 8658* (MEXU, MO); Dto. Tehuantepec, Cerro Picacho, 8.1 km al N de Guevea de Humbolt, [16.8122, -95.3921], 1800 m, 30 Aug 1986, *Torres-C. 8847* (MEXU); Dto. Mixe, Mpio. Totontepec Villa de Morelos, Mirador de Amatepec, 7 km al N de Totontepec, 17.2833, -96.0167, 1930 m, 27 Oct 1987, *Torres-C. 10381* (MEXU); Dto. Tehuantepec, Mpio. Santiago Lachiguiri, Cerro Selva del Aserradero, al E de crucero Guadalupe, el cual sale se encuentra 12.5 km al NE de Santiago Lachiguiri, 16.7167, -95.5000, 1400–1700 m, 9 May 1991, *Torres-C. 13973* (MO); along rd. between Ixtlán and Valle Nacional, 26.5 mi by road above Valle Nacional, 17.6167, -96.4167, 6500 ft, 20 Jun 1969, *Webster 15385* (DAV, MEXU, MO); **Puebla**: San Sebastian Tlacotepec, La Guacamaya, [18.3375, -96.8231], 1100 m, Oct 2006, *Mota-Cruz 652* (XAL); Mpio. Xicotepec de Juárez, 5 km al NE de Xicotepec, carr. a La Ceiba, 20.3167, -97.8000, 1200 m, 24 Feb 1987, *Toriz-A. 276* (MEXU, NY); **Veracruz**: Mpio. Xico, Texolo, 3 km al SW de Xico, 19.4500, -96.9500, 22 May 1971, *Dorantes 97* (NY, XAL); Mpio. Xico, Texolo, 3 km al SW de Xico, 19.4500, -96.9500, 22 May 1971, *Dorantes 100* (NY, XAL); Mpio. Huatusco, Las Cañadas, 19.1775, -96.9722, 1300 m, Mar 2004, *Gallardo-H. 3065* (MEXU, XAL); Mpio. Coatepec, Rancho El Riscal, 19.4544, -96.9964, 2180 m, 26 Sep 2007, *Hernandez-M. 24* (XAL); Xico, 3500 ft, 20 Sep 1906, *Johnson s.n.* (NY); Mpio. Cerro del Aguila, 13 km (19 cm by road) N of Altotonga on road to Tlapacoyan, 19.8833, -97.2167, 1250 m, 28 Jun 1980, *Nee 18519* (XAL); Vic. “La Calavera,” 10 km N of Altotonga, (13 km by road) on road to Tlapacoyan, 19.8500, -97.2167, 1350 m, 28 Jun 1980, *Nee 18657* (MO); Mpio. Teocelo, gorge of Río Teocelo, 2 km NW of Teocelo at bridge of Teocelo-Coatepec highway, 7 km (by air) SSW of Coatepec, 19.4000, -96.9833, 1050 m, 2 Dec 1981, *Nee 23551* (BR, BRIT, MEXU, MO, NY, WIS); Mpio. Xico, gorge at Puente Acabaloya, ca. 1 km SE of Xico Viejo and 5 km NW of Xico along trail between the two, 19.4500, -97.0500, 1600 m, 31 Mar 1983, *Nee 26261* (NY, XAL); Mpio. Xalapa, Parque Ecológico of Jardín Botánico Clavijero, 3 km SW of Xalapa on road to Coatepec, 19.5000, -96.9333, 1300 m, 22 Jan 1984, *Nee 28981* (BR, MEXU, MO, WIS); barranca de Tenampa, Zacuapan, [19.2813, -96.8477], Mar 1914, *Purpus 7085* (NY); Mpio. Atzalan, 11 km al N de Atzalan, “La Calavera” (Rancho La Jornada), camino a Tlapacoyan, [19.8570, -97.2199], 1050 m, 25 Jun 1995, *Sousa-P. 588* (MEXU); Mpio. Teocelo, carretera a Teocelo, 1 km antes de Teocelo, barranca de Teocelo, 19.3833, -96.9667, 1000 m, 19 Nov 1981, *Vazquez-B. 352* (XAL); Mpio. Teocelo, barranca de Teocelo, 1 km antes de Teocelo, 19.3833, -96.9667, 1000 m, 16 Jun 1982, *Vazquez- B. 525* (XAL); Mpio. Totutla, Zochiapa, [19.2058, -96.9502], 1300 m, 26 Apr 1972, *Ventura-A. 5299* (WIS, XAL); Mpio. Huatusco, Tepezingo, [19.1803, -96.9491], 1350 m, 28 Jul 1975, *Ventura-A. 11734* (MEXU); Mpio. Totutla, El Mirador, 1000 m, 23 Jun 1981, *Ventura-A. 14787* (MEXU); Mpio. Ixhuacán, Amatla, [19.3303, -97.0524], 1300 m, 14 Mar 1980, *Ventura-A. 16938* (MEXU, MO); Mpio. Tlalnehuayocan, al E del Rancho Tejocotal, 19.5083, -96.9861, 1640 m, 24 Nov 1990, *Zamora-C. 2732* (MEXU, XAL); Mpio. Xalapa, kilometro 7 carretera San Andresito, 1320 m, 8 Apr 1976, *Zola-B. 609* (MEXU, NY).

**17. *Lycianthes glabripetala***

**Mexico: Querétaro**: Mpio. Landa, 1 km al oriente del Puerto del Sabino, [21.2375, -99.1023], 1040 m, 4 Oct 1988, *Rubio 190* (DAV, IEB); Mpio. Landa, 1 km al sureste de El Naranjo, [21.2421, -99.1014], 1050 m, 24 Jul 1989 *Rubio 909* (DAV, IEB); **Veracruz**: Mpio. Chocaman, Tetela, Cerro Peña, 1450 m, 24 Oct 1993, *Janis-G. 12* (MEXU); Mpio. Zontecomatlán, along Huayacocotla-Zontecomatlán road, 1 km NE of San Antonio Ixtatetla, 20.7, -98.3833, 1300 m, 27 Apr 1983, *Nee 26820* (NY, XAL); San Andrés Tlalnehuayopan, [19.5644, -96.9738], 1450 m, 26 Jan 1976, *Ventura-A. 12352* (MEXU, XAL).

**18. *Lycianthes gongylodes***

**Guatemala. El Quiché**: El Boquerón, 8000–8200 ft, 6 Aug 1964, *Proctor 25432* (BRIT, MO, TEX); **Huehuetenango**: Sierra de los Cuchumatanes, Cruz de Limón, between San Mateo Ixtatán and Nucá, [15.8264, -91.4385], 2600–3000 m, 31 Jul 1942, *Steyermark 49839* (NY); Mpio. San Mateo Ixtalán, Sierra de los Cuchumatanes, near the place called Kurus Lemun, 4 miles E of San Mateo Ixtatán along road to Barillas, [15.8200, -91.4385], 8500 ft, 7 Aug 1965, *Breedlove 11628* (TEX).

**19. *Lycianthes gorgonea***

**Belize: Cayo**: high ridge, base of hill, 48 miles section, Humming Bird Highway, [16.9938, -88.3008], 2 May 1956, *Gentle 9081* (LL, MO); Valentin, [16.767865, -89.129992], Jun-Jul 1936, *Lundell 6248* (A, NY); Chalillo Crossing, [16.85897, -89.016933], Jun–Aug 1936, *Lundell 6508* (A, NY, TEX).

**Guatemala. Alta Verapaz**: between Limón and Chisec, [15.786853, -90.366697], 200–230 m, 19 Mar 1942, *Steyermark 45116* (NY, LL); **Petén**: Dolores, about 4 km E of the village, [16.4755, -89.3227], 26 Apr 1961, *Contreras 2191* (LL); Tikal, on trail between Group E ruins and Temple I, [17.2225, -89.6271], 29 Aug 1959, *Contreras 125* (LL, MO); Sto Toribio, bordering the village, E in clearing [16.6180, -89.5196], 28 Jul 1961, *Contreras 2697* (LL, MO); Dolores, on Río Mopan trail, 5 km E, [16.4755, -89.3227], 800 m, 30 Aug 1961, *Contreras 2831* (LL, MO); Lacandon, on La Ruina trail about 4 km E, [17.102789, -91.172465], 800 m, 7 Feb 1962, *Contreras 3345* (LL); Lacandon, about 3 km SW on El Caribal trail, [17.043755, -91.176318], 800 m, 19 Feb 1962, *Contreras 3414* (LL, MO); Sayaxché, on San Juan Acul. about 3 km SE from Río Pasion, [16.543073, -90.280076], 16 Mar 1964, *Contreras 4045* (LL); bordering the village, W Tikal National Park, [17.200122, -89.752932], 800 m, 11 Mar 1966, *Contreras 5544* (NY); Tikal National Park on Uaxactún Road, 1 km, [17.2317, -89.6192], 15 Apr 1968, *Contreras 7702* (LL, MO); Tikal National Park, Tikal, Aguada Las Cucas, [17.224589, -89.613593] Mar 1961, *Ibarra 69* (LL); Tikal National Park, Tikal, [17.222463, -89.622098], Mar 1961, *Ibarra 79* (LL); Tikal, on trail to Uaxactún, [17.289758, -89.636089], 7 Apr 1959, *Lundell 15867* (LL, MO); Aguada La Cucas, [17.224589, -89.613593], 5 Mar 1961, *Lundell 16893* (LL, MO); Sayaxché, about 6 km SE of village, [16.507705, -90.134386], Feb 1964, *Lundell 18054* (LL); Uaxactún, on Dos Lagunas road, in zapotal/ramonal, 3 km W, [17.189682, -89.83063], 22 Jan 1977, *Lundell 20534* (LL, MO); Ruinas Plaza Mayor Tikal, [17.225, -89.61305], 333 m, 16 Nov 1965, *Molina-R. 15793* (NY); Santa Elena en orillan de el camino para Sayaxché, a km 47, lado oeste, 18 Nov 1971, *Ortíz 2094* (G, MO).

**Mexico. Chiapas**: 50 km SW of Palenque on road to Ocosingo near Colonia Ursulo Galvano, [17.2441, -92.0400], 370 m, 9 Nov 1980, *Breedlove 47381* (NY); 5 miles SE of Palenque on the road to Chancala, Ocosingo and San Cristóbal de las Casas, [17.4700, -91.9600], 200 m, 4 Jul 1977, *Croat 40161* (MO); Mpio. Berriozábal. along road from Berriozábal to Las Maravillas, ca. 1.4 km south of the town of Efraín A Gutiérrez, in remnant of tall forest called La Mata Café, 16.8711, -93.2956, 1005 m, 12 Sep 2017, *Dean 9528* (DAV); Mpio. of Berriozábal, 13 km N of Berriozábal near Pozo Turipache and Finca El Suspiro, [16.8744, -93.3252], 900 m, 25 Dec 1972, *Breedlove 30791* (MO); Mpio. of Berriozábal, 13 km N of Berriozábal near Pozo Turipache and Finca El Suspiro, [16.8800, -93.3200], 1000 m, 3 Sep 1976, *Breedlove 39894* (MO); Mpio. of La Libertad, 15–20 km towards Chancala on road to Bonampak from the Palenque-Ocosingo road, [17.3969, -91.8122], 280 m, 4 Jan 1981, *Breedlove 49142* (MO, NY); Mpio Ococingo, a 2 km al S de Bonampak en dirección al Río Lacanja, 16.6854, -91.0627], 320 m, 26 Sep 1984, *Martínez 7884* (LL, MO, NY); Crucero de Bonampak a 0.8 km de la carretera rumbo al SE, 16.7992, -91.0964, 464 m, 25 Nov 2002, *Aguilar-M. 4373* (NY); Mpio. of Ocosingo, 70 km SW of Palenque on road to Ocosingo along the Jol Uk’um, [17.1739, -92.1175], 550 m, 11 Nov 1980, *Breedlove 47400* (LL, MO); same location, 4 Dec 1980, *Breedlove 48370* (LL, NY); same location, 14 Jan 1981, *Breedlove 49584* (MO); Mpio. of Palenque, 6–12 km S on the road to Ocosingo, [17.4542, -91.9875], 300 m, 22 Feb 1972, *Breedlove 24237* (MO, NY); same location, 27 Jul 1972, *Breedlove 26530* (MO); Mpio. of Palenque, 50 km SW of Palenque on road to Ocosingo near Colonia Ursulo Galvano, [17.2441, -92.0400], 370 m, 9 Nov 1980, *Breedlove 47381* (MO); **Oaxaca**: Mpio. Santa María Jacatepec, predio La Joya, [17.8736, -96.0226], 23 Feb 1985, *Gutiérrez 808* (MEXU); Mpio. Santa María Jacatepec, predio La Joya del Obispo, [17.8599, -96.2060], 12 Aug 1990, *Ramos 435* (IEB, MEXU, XAL); Mpio. Santa María Jacatepec, predio El Águila subida, en San Agustín, 25 km al oeste de la Reforma, carretera a Ayozintepec, 17.8333, -96.1000 21 Feb 1988, *Torres-C. 11538* (MEXU); **Tabasco**: Teapa, ladera W del Cerro del Madrigal, cerca de la base, Puyacatengo, [17.5213, -92.9225], 25 Mar 1992, *Guadarrama-O. 1244* (TEX); **Veracruz**: Mpio. Minatitlán, 6.6 km al N de la terracería La Laguna-Río Grande, sobre el camino nuevo (no completo) a Ejido Belisario Domínguez, el cual sale de la terracería 14.7 km al E. de La Laguna, 17.3333, -94.3667 130 m, 13 Jul 1980, *Wendt 2557* (MO).

**20. *Lycianthes grandifolia***

**Mexico. Chiapas**: Mpio. Jaltenango, al SE de la Reserva El Triunfo, camino a Cupresal, 1825 m, 13 May 1982, *Calzada 8921* (XAL); Mpio. Angel Albino Corzo, 2 km de las instalaciones de El Triunfo vereda a Cipresal, Jaltenango de la Paz, 15.6558, -92.8081, 1850 m, 11 May 1988, *Calzada 13870* (XAL); Mpio. Jaltenango, Reserva El Triunfo, Poligono 1, Camp HQ – Finca Prusia, 15.65, -92.80, 1700 m, 23 Jun 1990, *Heath 1251* (MEXU); Mpio. Mapastepec, Reserva El Triunfo, Poligono 1, Deslave – Cipressal, 15.65, -92.80, 1750 m, 14 Jun 1990, *Heath 1012* (MO).

**21. *Lycianthes heteroclita*** – Due to the large number of specimens examined, the specimens cited here are just from Mexico and Guatemala, and do not reflect the full extent of the specimens studied (which encompass Mexico to Panama).

**Guatemala. Alta Verapaz**: Chahal, 3 km from airfield, , [15.7862, -89.6049], 6 Oct 1968, *Conteras 7843* (MO, LL); 7 miles up road to Oxec, along road which turns off Hwy 7E between Tucuru and El Estor ca. 6 km NE of Panzos, [15.4401, -89.6113], 20 Jul 1977, *Croat 41645* (MEXU, MO); city of Cobán, garden of residence along 1^st^ St belonging to the family of Fredy Archila, seed of this plant originally collected at Finca Siguanha in forest at 1400 m in elevation, 15.4749, -90.3722, 1335 m, 9 Aug 2017, *Dean 9500* (DAV); Puente del Río Cahabón, [15.4667, -90.3000], 1000 m, 7 Jun 1997, *Equipo CECON 529* (BIGU); Cobán, Aldea Sanimtacá (?), 15.4911, -90.4656, 1245 m, 29 Sep 2013, *Mérida 121* (BIGU); Carcha, [15.4333, -90.3167], 1320 m, 6 Jun 1997, *Solorzano* (BIGU); Mpio. Panzós, along road to Hidrochulac and Cahabón from (N of) Tactic-El Estor road, [15.4793, -89.6614], 650–750 m, 2 Sep 1988, *Stevens 25363* (MEXU, MO); along route no. 5 between Chirriacté and Semococh, [15.8622, -89.7195], 500–900 m, 10 May 1942, *Steyermark 46341* (MO, NY); Cubilquitz [Cubilhuitz], [15.6675, -90.4293], 350 m, Oct 1903, *von Tuerkheim 8556* (M); **Escuintla**: 8 km S of Palin, [15.8622, -90.7391], 7 Aug 1970, *Harmon 2958* (MEXU); Mpio. Palín, Finca Comunal, El Chilar, 14.3536, -90.7283, 959 m, 10 Nov 2010, *Véliz 22280* (BIGU); **Huehuetenango**: along road from San Ramón to Barillas, 15.8644, -91.2149, 720 m, 15 Aug 2017, *Dean 9514* (DAV); between Ixcan and Finca San Rafael, Sierra de los Cuchumatanes, 200–800 m, [15.8744, -90.9859], 24 July 1942, *Steyermark 49399* (NY); **Izabal**: Puerto Méndez, on Toquela River road, 5 km from the village, in zapotal-corozal, [15.8609, -89.2038], 6 Sep 1969, *Conteras 9083* (MO, LL); same location, 7 Sep 1969, *Conteras 9085* (MO, LL); Puerto Méndez, on Arenales Road, 5 km NWW, in corozal, [15.9025, -89.2373], 18 Sep 1969, *Conteras 9204* (MEXU, MO, LL); Santo Tomás de Castilla, on road past Las Escobas going to the Guatel tower, 15.7000, -88.6333, 17 Mar 1988, *Marshall 411* (M, NY); W of Santo Tomás de Castilla, near Guatel antennas on one of the summits of Cerro San Gil, 15.6961, -88.6597, 300 m, 21 Sep 1997, *Nee 47314* (MO, NY); same location, 800–900 m, 21 Sep 1997, *Nee 47320* (MO, NY); vicinity of Quirigua, [15.2751, -89.0724], 75–225 m, 15–31 May 1922, *Standley 24145* (NY); Montañas del Mico, 11 km W of Santo Tómas de Castilla, microwave tower, [15.6719, -88.6929], 940 m, 8 Sep 1988, *Stevens 25500* (MEXU, MO, NY); along Río Frío, Cerro San Gil, [15.4984, -88.6159], 50–75 m, 19 Dec 1941, *Steyermark 41614* (MO); along railroad, between Puerto Barrios and Milla 7, [15.3942, -88.8844], 5–20 m, 3 Jan 1942, *Steyermark 42061* (NY); **Petén**: La Cumbre, bordering Cadenas Road, km 137/138, [16.0667, -89.3457], 27 Sep 1966, *Contreras 6257* (LL); Dolores, on Sto. Toribio trail, 2 km NWW of the village, [16.5189, -89.4432], 27 May 1961, *Conteras 2396* (MO, LL); La Cumbre, S, [16.0787, -89.3483], 20 Sep 1966, *Conteras 6164* (MEXU, MO, LL); Cansis, km 134 of Cadenas Road, [16.1347, -89.3895], 17 Oct 1966, *Conteras 6439* (MO, LL); 5 km from La Cumbre, bordering Pusila River, 1 km S from the village, [16.0809, -89.3145], 22 Aug 1969, *Conteras 9006* (MO, LL); La Cumbre, in zapotal, E, on Río Purula road, [16.0823, -89.3126], 19 Sep 1975, *Lundell 19880* (MEXU, MO, LL); La Cumbre, Pusila road, 5 km, [16.1078, -89.3417], 17 Aug 1976, *Lundell 20194* (MO, LL); entre orilla de Río Pasión y Ceiba, [16.5203, -90.0482], 150 m, 18 Nov 1965, *Molina-R. 15834* (MO, NY); Sayaxche, en orillando el camino para Seibal, a km 69, lado suroeste, [16.5249, -90.1891], 18 Nov 1971, *Tún-Ortíz 2097* (NY); **Quetzaltenango**: Mpio. Colomba, Fca. San Francisco Pie de la Cuesta, 14.7281, -91.7178, 1113 m, 15 Feb 2011, *Velásquez 1728* (BIGU); **Quiché**: Nebaj, about 12 km W, [15.4058, -91.1461], 8000 ft, 4 Jul 1964, *Conteras 5196* (LL); Nebaj, about 12 km W, [15.40521, -91.2392], 8000 ft, 4 Jun 1964, *Contreras 5198* (TEX); **Retalhuleu**: Retalhuleu, [14.5248, -91.6854], Mar 1876, *Bernoulli 2393* (GOET); **Santa Rosa**: region of Platanares, between Taxisco and Guazacapán, [14.0672, -90.4415], 220 m, 3 Dec 1940, *Standley 79062* (MO); **Zacapa**: Gualan, [15.1194, -89.3556], 122 m, 28 Dec 1905, *Kellerman 5678* (LL).

**Mexico. Chiapas**: Mpio. Ocosingo, 4.5 km al NE de Nuevo Guerrero en Arroyo Aguado, 17.0136, -91.2864, 189 m, 17 May 2002, *Aguilar-M. 802* (MEXU); Mpio. Ocosingo, Restaurante la Escondida, en la 11 de Julio, camino a Palenque, 17.1772, -91.4881, 163 m, 12 Jun 2002, *Aguilar-M. 1388* (MEXU); Mpio. Ocosingo, Rancho El Edén, a 4 km al E del Poblado Nuevo Guerrero, 17.0017, -91.2797, 185 m, 27 Jul 2002, *Aguilar-M. 1977* (MEXU); Mpio. Ocosingo, a 1 km al S de San Javier, 16.7894, -91.1017, 295 m, 24 Aug 2002, *Aguilar-M. 2149* (MEXU); Mpio. Ocosingo, al E del crucero de San Javier, 16.7997, -91.1036, 434 m, 26 Aug 2002, *Aguilar-M. 2289* (MEXU); Mpio. Ocosingo, El Encaño a 3.2 km al NO de Naité, 16.7811, -91.0625, 220 m, 20 Sep 2002, *Aguilar-M. 2934* (MEXU); Mpio. Ocosingo, a 1.2 km al S del crucero San Javier, 16.7892, -91.1083, 390 m, 9 Oct 2002, *Aguilar-M. 3237* (MEXU); Mpio. Ocosingo, a 3.8 km al SE del paraíso, 16.9300, -91.2711, 385 m, 14 Oct 2002, *Aguilar-M. 3453* (MEXU); Mpio. Ocosingo, Puente Chansayab, 16.7672, -91.1083, 328 m, 18 Oct 2002, *Aguilar-M. 3787* (MEXU); Mpio. Ocosingo, a 9 km al NO del crucero San Javier, 16.7956, -91.0928, 394 m, 12 Dec 2002, *Aguilar-M 4608* (MEXU); Mpio. Ocosingo, a 4.4 km al SO de San Javier, 16.8136, -91.0678, 386 m, 16 Dec 2002, *Aguilar-M. 4703* (MEXU); Mpio. Ocosingo, a 1.25 km al SO de San Javier, [16.7883, -91.1081], 388 m, 18 May 2003, *Aguilar-M. 6762* (MEXU); Mpio. Ocosingo, a 2.46 km al S de San Javier, 16.8108, -91.2717, 262 m, 21 May 2003, *Aguilar-M. 6804* (MEXU); Mpio. Ocosingo, a 6.11 km al NE del poblado Nuevo Guerrero, 17.3503, -91.2567, 248 m, 21 Nov 2003, *G. Aguilar 8496* (DAV, MEXU); Mpio. Ocosingo, a 0.64 km al O del crucero de Bonampak, 16.7692, -91.1097, 313 m, 13 Dec 2003, *Aguilar-M. 8911* (MEXU); Mpio. Ocosingo, a 1.38 km al NE de Lacanjá Chasayab, [16.7597, -91.1167], 330 m, 14 Dec 2003, *Aguilar-M. 8942* (MEXU); Mpio. Ocosingo, a 5.86 km al E del crucero de San Javier, 16.8147, -91.0611, 216 m, 17 Dec 2003, *Aguilar-M. 9025* (MEXU); Mpio. Ocosingo, a 3.86 km al NE de Nuevo Guerrero, 17.0067, -91.2686, 247 m, 24 Dec 2003, *Aguilar-M. 9123* (MEXU); Mpio. Ocosingo, a 6.5 km al S de Nuevo Guerrero sobre el camino a Santo Domingo, 16.9303, -91.2714, 400 m, 6 May 2002, *Alvarez-M. 888* (MEXU); Mpio. Ocosingo, a 1.7 km al S del crucero San Javier, 16.7903, -91.1075, 372 m, 24 Nov 2002, *Alvarez-M. 2556* (MEXU); Mpio. Oxchuc, secondary road to Tolbilja, off main road from Oxchuc to Ocosingo, about 500 m straight line) after turnoff from main road, 16.8317, -92.2650, 1514 m, 4 Dec 2012, *Bohs 3946* (DAV, MEXU); Mpio. Ocosingo, road from Ocosingo to Palenque. In pueblo de Bahtaj, about 28 km NE of Ocosingo, roadside, 17.1447, -92.1283, 858 m, 6 Dec 2012, *Bohs 3962* (DAV, MEXU); Mpio. of Ocozocoautla de Espinosa, just above the community and lake of Malpaso. 45 km N of Ocozocoautla, [17.0605, -93.5732], 550 m, 17 Oct 1971, *Breedlove 20732* (MEXU, MO); Mpio. Arriaga, along ravines 13 km N of Arriaga along Mexican Hwy 195, [16.3308, -93.8780], 830 m, 18 May 1972, *Breedlove 25226* (MEXU, MO); Mpio. Palenque, along the ridges 6–12 km S of Palenque on the road to Ocosingo. 300 m, 27 Jul 1972, *Breedlove 26501* (MO); Mpio. Ocozocoautla de Espinosa, 18–20 km N of Ocozocoautla along road to Mal Paso, [16.8950, -93.4527], 800 m, 27 Sep 1972, *Breedlove 28143* (MEXU, MO); Mpio. Huixtla, 6–8 km NE of Huixtla along road to Motozintla, [15.1858, -92.4183], 200 m, 6 Oct 1972, *Breedlove 28561* (MEXU, MO); Mpio. Cintalapa, at crest of ridge 3 km E of Francisco Madero, NE of Cintalapa, [16.8143, -93.7346], 1250 m, 25 Aug 1974, *Breedlove 36672* (MEXU, MO); Mpio. Ixtacomitan, along road 7 km SW of Ixtacomitan, [17.4173, -93.1472], 250 m, 6 Oct 1980, *Breedlove 45923* (MEXU, MO); Mpio. Villa Corzo, above Colonia Vicente Guerrero on road to Finca Cuxtepec, 1100 m, 20 Oct 1980, *Breedlove 46587* (MO); Mpio. Palenque, 50 km SW of Palenque on road to Ocosingo near Colonia Ursulo Galvano, [17.2440, -92.0401], 370 m, 9 Nov 1980, *Breedlove 47384* (MEXU); Mpio. Angel Albino corzo above finca Cuxtepec, [15.8568, -92.7264], 11 Aug 1981, *Breedlove 52066* (MEXU); Mpio. Solosuchiapa, 2–4 km, below Ixhuatán along road to Pichucalco, [17.3258, -92.9992], 1200 m, 11 Sep 1981, *Breedlove 52691* (MEXU, MO, NY); Mpio. La Trinitaria, 15 km ENE of Dos Lagos above Santa Elena, [16.1317, -91.5538], 29 Dec 1981, *Breedlove 56629* (MEXU, MO); En los alrededores de la zona Arqueológica de Palenque, [17.4868, -92.0394], 5 Mar 1982, *Cabrera-C. 1954* (MEXU); Mpio. Ocosingo, a 13 km al S de Palenque, sobre la carretera a Ococingo, [17.4339, -91.9956], 8 Jul 1983, *Cabrera-C. 5022* (MEXU); en los alrededores de la zona Arqueológica de Palenque, a 9 km al oeste de el Pueblo de Palenque, [17.4875, -92.0398], 16 Aug 1983, *Cabrera-C. 5215* (NY); same location and date, *Cabrera-C. 5283* (MEXU, MO, NY); en los alrededores de la Zona Arqueológica de Palenque, a 9 km al W de la Ciudad de Palenque, [17.4875, -92.0390], 27 Sep 1983, *Cabrera-C. 5604* (MEXU); a 6 km al S de la desviación a Chancala, sobre la carretera a Ocosingo, [17.4200, -91.9981], 9 Dec 1983, *Cabrera-C. 6217* (MO, NY); a 12 km al S del entronque a Chancala, sobre la carretera Palenque-Ocosingo, [17.3743, -91.9905], 3 Aug 1984, *Cabrera-C. 7041* (MEXU); 12 km al S de Palenque o 3 km al S de la desv. a Chancala, sobre la carr. a Ocosingo, [17.4389, -91.9880], 19 Nov 1986, *Cabrera-C. 12341* (MEXU); Mpio. Ocosingo, 4.1 km al E de Nuevo Guerrero, 16.9822, -91.2464, 299 m, 5 May 2002, *Calonico-S. 23099* (MEXU); Mpio. Ocosingo, a 2.07 km al noroeste de Corozal (crucero de San Javier), 16.8039, -91.0886, 379 m, 10 Oct 2002, *Calonico-S. 24103* (MEXU); Mpio. Ocosingo, a 4.69 km al S de Nuevo Guerrero, 16.9514, -91.2589, 327 m, 16 Oct 2002, *Calonico-S. 24535* (MEXU); Mpio. Ocosingo, a 3.2 km al N de Corozal, 16.8142, -91.0614, 371 m, 21 Oct 2002, *Calonico-S. 24897* (MEXU); Mpio. Escuintla, El Triunfo, [15.3494, -92.5444], 350 m, 24 Nov 1977, *Calzada 4018* (MEXU); Mpio. Ocozocoautla de Espinosa, La Cueva, al NW del rancho Corocito, Reserva del Ocote, 770 m, 29 Apr 1983, *Calzada 9742* (MEXU); 27 km N of Huixtla, near Belisario Domínguez, 10 Sep 1985, *Cate* (NY); Palenque to Bonampak, 60 miles SE of Palenque, [17.0089, -92.1248], 400 m, 5 Jul 1977, *Croat 40178* (MEXU, MO); along gravel road between Palenque and Bonampak, 88–90 miles SW of Palenque, 350–370 m, 5 Jul 1977, *Croat 40205* (MO); ca 8.5 miles NE of Escuintla on gravel road to El Triunfo, [15.3516, -92.5558], 250 m, 21 Aug 1977, *Croat 43829* (MEXU, MO); along trail between Finca California (at base of S slope of Monte Ovando and ca 4 km N of Ovando), [15.3860, -92.6610], 450–850 m, 14 Feb 1979, *Croat 47554* (MEXU, NY); along road between Bochil and Pichucalco, near shrine along road at 430 m, 1.7 km S of Ixhatan, 3.5 mi N of Ignacio Zaragosa, 17.2667, -93.0167, 430 m, 24 Aug 1996, *Croat 78586* (DAV, MO); Mpio. Palenque, vicinity of the Palenque archeological site, [17.4875, -92.0390], 170 m, 11 May 1982, *Davidse 20333* (MEXU, MO); along switchbacks on road just before Palenque ruins, 18 Nov 1975, *Dreyer 358* (MO); Mpio. Ocosingo, 27 km al sureste de Palenque, por la carretera fronteriza hasta el cricerpo Chancala, despues 55.6 km por el camino de terracería hacia Monte Libano [Note by Ellen Dean: Original elevation on this record was 1900 m, but the location it maps to is 1100 m], 16.9333, -91.5500, 1100 m, 25 Jul 1999, *Duran-Fernandez 1332* (MEXU, XAL); Mpio. Ocosingo, 2 km al NE de la comunidad Maya de Lacanjá, 16.7500, -91.1333, 450 m, 6 Sep 1994, *Flores 4542* (MEXU); Mpio. Ocosingo, 3 km al SE de la comunidad Maya de Lacanjá-Chansayab, 16.7500, -91.1333, 450 m, 2 Oct 1994, *Flores 4641* (MEXU); Mpio. Ocosingo, 750 m al N de Lacanjá-Chanzayab en área de acahuales, [16.7635, -91.1303], 500 m, 21 Sep 1989, *González-Espinosa 755* (MEXU); 3 mi N of Acacoyagua, foothills of Mt. Ovando, [15.3853, -92.6690], 30 m, 1 Jun 1973, *Hansen 1627* (MEXU); ruinas de Palenque, CA 110 km ESE of Villahermosa, 120 m, 13 Dec 1972, *Iltis 27264* (WIS); Mpio. Ocosingo, la comunidad Lacandona de Lacanhá-Chansayab, a 130 km al sureste de Palenque, por la carretera fronteriza hasta el crucero San Javier, después 8 km hacia el oeste, 16.7333, -91.0833, 400 m, 22 Feb 1994, *Levy-T. 43* (MEXU); Mpio. Ocosingo, Bonampak, [16.7039, -91.0646], 350 m, 19 May 1984, *Martínez-S. 6426* (BIGU, MEXU); Mpio. Ococingo, en campamento COFOLASA, a 24 km al SE de Crucero Corozal, camino a Boca Lacantum, 220 m, 12 Aug 1984, *Martínez-S. 7013* (MO, NY); same location and date, *Martínez-S. 7020* (MEXU, MO, NY); Mpio. Ococingo, a 5 km al E de Crucero Corozal, camino a Frontera Corozal,16.7692, -90.9636, 200 m, 16 Aug 1984, *Martínez-S. 7223* (MEXU, MO, NY); Mpio. Ocosingo, a 10 km al SE de Crucero Corozal camino a Boca Lacantum, [16.7244, -90.9267], 200 m, 18 Aug 1984, *Martínez-S. 7395* (MEXU, MO, NY); Mpio. Ocosingo, a 10 km al E de Crucero Corozal camino a Frontera Corozal, [16.7915, -90.9281], 200 m, 19 Aug 1984, *Martínez-S. 7475* (MEXU, NY); Mpio. Ococingo, 6 km al S de campamento COFOLASA el cual esta a 24 km al SE de Crucero Corozal, camino Palenque-Boca Lacantum, [16.6623, -90.8155], 200 m, 17 Oct 1984, *Martínez-S. 8467* (MEXU, MO, NY, LL); Mpio. Ocosingo, en Crucero Corozal sobre el camino Palenque-Boca Lacantum, [16.7622, -91.0072], 180 m, 18 Apr 1985, *Martínez-S. 12115* (MEXU, MO); Mpio. Ocosingo, 5 km al S de Crucero Corozal, [16.7438, -90.9879], 250 m, 21 Apr 1985, *E. Martínez S. 12206* (MEXU, MO); Mpio. Ococingo, en crucero Corozal, camino Palenque-Boca Lacantum, [16.7622, -91.0072], 180 m, 9 Nov 1985, *Martínez-S. 15306* (MEXU, MO, NY); Mpio. Ococingo, a 14 km al NW de Crucero Corozal, sobre el Camino Palenque Boca Lacantum, [16.7983, -91.0975], 450 m, 23 Jan 1986, *Martínez-S. 16629* (MEXU, NY); Mpio. Ocosingo, 2 km al N de Tanhi Perla camino a Monte Líbano-Ocosingo, [16.8303, -91.5399], 700 m, 11 Apr 1986, *Martínez-S. 17644* (MEXU); Mpio. Tuxtla Chico, en el Río Suchiate, a 3 km al E de Cacahoatán, [14.8714, -92.1476], 400 m, 4 May 1987, *Martínez-S. 20714* (MO); Mpio. Ocosingo, El Pozo Cantil 1, a 10 km al E de Zamora Pico de Oro, [16.3603, -90.6914], 153 m, 19 May 1987, *Martínez-S. 21105* (MEXU); Mpio. Ocosingo, a 6.4 km al SSE de Nuevo Guerrero, 16.9319, -91.2619, 380 m, 9 Aug 2002, *Martínez-S. 35600* (MEXU); Mpio. Ocosingo, a 6.4 km al SSE de Nuevo Guerrero, 16.9319, -91.2619, 380 m, 9 Aug 2002, *Martínez-S. 35605* (MEXU); Mpio. Ocosingo, banco de grava de San Javier, [16.8000, -91.0972], 440 m, 13 Aug 2002, *Martínez-S. 35713* (MEXU); Escuintla, [15.3230, -92.6541], Nov–Dec 1937, *Matuda 2141* (MEXU, NY, LL); Mpio. Escuintla, Esperanza, [15.2447, -92.6342], 23 Feb 1948, *Matuda 17477* (MEXU, NY); 10–15 mi S of Pichucalco, off Hwy 195, 1200 ft, 8 Jul 1965, *Maxwell 204* (MO); Mpio. Ocosingo, 3 km al SE de San Javier, en el camino a Corozal o Frontera Echeverría, [16.0876, -91.0823], Dec 1979, *González-Medrano 11735* (MEXU); Mpio. Palenque, Misolja, [17.3928, -92.0002], 250 m, 30 Nov 1982, *Méndez-Ton 5132* (MEXU, MO); Mpio. Tila, Colonia Kokijaz, [17.2800, -92.4850], 1200 m, 5 Dec 1982, *Méndez-T. 5215* (MO); same location, *Méndez-T. 5283* (MEXU); Mpio same location, 1000 m, 5 May 1983, *Méndez-T. 5953* (MEXU, MO); same location, 1300 m, 1 Jun 1983, *Méndez-T. 6085* (MEXU, MO); Mpio. Yajalón, Yajalón, Ch’ijtaltik, [17.1850, -92.3689], 700 m, 5 Jul 1983, *Méndez-T. 6282* (MEXU, MO, WIS); Mpio. Sabanilla, Finca Carmen, 500 m, 15 Jul 1983, *Méndez-T. 6334* (MEXU637398, MO); Mpio. Tila, Colonia Kokijaz, [17.2800, -92.4850], 900 m, 25 Jul 1983, *Méndez-T. 6395* (MEXU, MO); Mpio. Tajalón, la espalda del cerco Tz’iz Ton, 600 m, 30 Aug 1983, *Méndez-T. 6555* (MO); Mpio. Yajalón, camino la punta del Cerro Ventana, [17.5920, -92.3281], 20 Sep 1983, *Méndez-T. 6704* (MEXU, MO); Mpio. Tila, Finca Morelia, [17.3244, -92.5322], 1 Oct 1983, *Méndez-T. 6741* (MEXU); Mpio. Yajalon, el Río del Paraíso, [17.2139, -92.3156], 6 Oct 1983, *Méndez-T. 6803* (IEB); Mpio. Trinitaria, Colonia Cuauhtémoc, 25 Jun 1984, *Méndez 7741* (MEXU); Mpio. Trinitaria, Col. Cuauhtémoc, km 24 de la carr. Monte Bello-Santa Elena. [16.1092, -91.6161], 15 Jun 1985, *Méndez-G. 8293* (MEXU, NY); Mpio. Palenque, entrance to ruins of Palenque, 17.4833, -92.0333, 100 m, 28 Dec 1985, *Nee 32369* (MO, NY); near Tapachula, 1986, *Nelson 3846* (NY); Mpio. Ocozocoautla de Espinosa, 9 km al NW Emilio Rabasa, 0.5 km al SE EL Aguajito, [16.9500, -93.6611], 640 m, 7 Oct 1991, *Ochoa-Gaona 3692* (MEXU); Mpio. Jitotol, Francisco I. Madero, 17.0806, -92.8014, 1600 m, 12 May 1999, *Osorio-Hernandez 61* (MEXU); Mpio. Ocosingo, Vereda de Enrique Paniagua, 105 m, 15 Oct 1994, *Paniagua 57* (MEXU); Mpio. Ocosingo, Vereda de Kim Bor Paniagua, 105 m, 16 Dec 1994, *Paniagua 264* (MEXU); Saracha, Finca Mexiquito, Jun 1913, *Purpus 979* (NY); Barsovia, Cerro del Boqueron, Jun 1914, *Purpus 7320* (MO, NY); Mpio. Ococingo, 1 km al W del crucero de Bonampak, [16.7917, -91.1085], 5 Aug 1982, *Quintanilla 40* (MEXU, MO); Mpio. Tenejapa, inmediaciones de Loma Bonita por la vereda que va a Rodulfo Figueroa, 16.2012, -93.3097, 350 m, 8 Oct 2002, *Ramírez-Marcial 916* (MEXU); Mpio. Arriaga, Sobre la carretera Arriaga-Tuxtla Gutiérrez, en el puente Toronjal, 16.3186, -93.8711, 400 m, 24 Feb 2002, *Reyes-García 4002* (MEXU); Mpio. Arriaga, cerca del Rancho Monte Bonito, sobre la carr. México 190, 16.3208, -93.9064, 450 m, 23 Apr 2002, *Reyes-García 4498* (MEXU); Mpio. Arriaga, Ejido López Mateos, [16.3558, -93.9661], 500 m, 10 Jun 2002, *Reyes-García 5118* (MEXU); Mpio. Cintalapa, Ejido La Majada, 16.4147, -94.0375, 580 m, 16 Jan 2004, *Reyes-García 6177B* (MEXU); rolling hills of NW end of Valley of Chiapas on road to Mal Paso, 41 km (by road), NW of Ocozocoautla. 17.2000, -93.6667, 350 m, 4, 5 Aug 1965, *Roe 913* (WIS); interior lowlands, km 33 (by road) S of Sureste on road to Mal Paso, near Tabasco border, 17.4167, -93.5833, 350 m, 25 Aug 1965, *Roe 1381* (WIS); Mpio. of Ocozocoautla de Espinosa, on the SW side of the Presa de Malpaso. [17.1269, -93.6004], 2200 ft, 5 Dec 1967, *Shilom-T. 3310* (DUKE, MEXU); Mpio. Ocosingo, camino al Miranda 2.4 km, [16.1222, -90.9372], *Sinaca-Colin 1137bis* (MEXU); Mpio. Ocosingo, 8 km antes de Zona arqueologica de Bonampak, 16.7656, -91.1025, 340 m, 30 Oct 2003, *Soto 74* (MEXU); Mpio. Ocosingo, a 2.07 km al NO de Corozal (Crucero de San Javier), 16.8039, -91.0886, 379 m, 10 Oct 2002, *Soto-Nuñez 24161* (MEXU); Mpio. Ocosingo, a 2.07 km al NO de Corozal (Crucero de San Javier), 16.8039, -91.0886, 379 m, 10 Oct 2002, *Soto-Nuñez 24162* (MEXU); Mpio. Ocosingo, a 6.8 km al NO de Crucero San Javier, Sierra de la Cojolita, 16.8453, -91.1481, 414 m, 11 Oct 2002, *Soto-Nuñez 24170* (MEXU); Mpio. Ocosingo, a 6.4 km al SSE de Nuevo Guerrero, 16.9319, -91.2619, 380 m, 15 Oct 2002, *Soto-Nuñez 24458* (MEXU); Mpio. Ocosingo, a 5.03 km al N de Corozal, 16.8292, -91.0536, 235 m, 21 Oct 2002, *Soto-Nuñez 24844* (MEXU); Mpio. Ocosingo, a 5.04 km al NE del crucero San Javier, rumbo a San Jacinto, 16.8139, -91.0608, 190 m, 24 Nov 2002, *Soto-Nuñez 24990* (MEXU); road toward La Fortuna from Huixtla Motozintla hwy, [15.1806, -92.4603], 420 m, 16 Sep 1988, *Stevens 25676* (MEXU, MO, NY); 11 km S of Crucero El Piñal, 2 km SE of El Piedron, 17.1667, -91.7500, 600 m, 26 Sep 1988, *Stevens 25896* (MEXU, MO, NY); a 13 km al NE de Arriaga, rumbo a Tuxtla Gutiérrez, [16.3310, -93.8794], 6 Apr 1983, *Téllez-Valdes 6544* (MEXU); Mpio. Ocosingo, Camino al Ejido de Cintalapa, [16.9344, -91.3717], 510 m, 29 Nov 1976, *Valdivia Q. 2373* (MEXU); Mpio. Tapachula, Santa Clara, 100 m, 9 Sep 1984, *Ventura 135* (MO); Mpio. Cacahoatan, Alpujarra, [15.0736, -92.1717], 750 m, 17 Oct 1984, *Ventura 530* (MEXU); Mpio. Monte Negro, Tapachula, [14.9489, -92.2417], 165 m, 24 Oct 1984, *Ventura 592* (MEXU, MO); Mpio. Tapachula, Río Malpaso, [14.9903, -92.2425], 300 m, 28 Mar 1985, *Ventura 1458* (MEXU); Mpio. Tuxtla Chico, Chico Primero de Cauhán, [14.9825, -92.2443], 200 m, 6 Jun 1985, *Ventura 1844* (MEXU, MO, XAL); Mpio. Cacahoatan, Ahuacatlan, [15.0406, -92.1774], 600 m, 8 May 1986, *Ventura 3624* (IBUG, IEB, MEXU, NY); Mpio. Cacahoatán, Unión Roja, [15.0068, -92.1677], 400 m, 20 Jun 1986, *Ventura 3812* (IEB, MEXU, MO, NY); Mpio. Palenque, 12.4 km al S de Palenque, sobre carretera a Mpio. Ocosingo (Carr. 199), 3 km al S del entronque con la carretera a Chancala (Carr. 192). Frecuente a orillas de la carretera, 17.4500, -91.9833, 320 m, 1 Dec 1979, *Wendt 2291* (MO, TEX); **Guerrero**: Mpio. Atoyac de Alvarez, 8 km al NE de Paraíso, carr. a Puerto del Gallo, [17.3622, -100.2071], 1050 m, 16 Aug 1982, *Tenorio-L. 1400* (NY); Mpio. San Luis Acatlán Yoloxóchitl, 4.37 km en línea recta al N de la comunidad, en el terreno del Sr. Enrique Rómulo (tierras de uso común), sobre el arroyo que va al paraje Salto de la Mona, 16.8531, -98.6723, 545 m, 22 Apr 2017, *Velazco-G. 40341* (DAV); **Jalisco**: Mpio: Casimiro Castillo, parte poniente del puente “La Calera” por la carretera Guadalajara-Barra de Navidad, [19.6730, -104.4287], 550 m, 17 Aug 1990, *López-V. 213* (WIS); **Oaxaca**: Dto. Pochutla, 15 km al N de Chacalapa, en Finca La Concordia, [16.1039, -97.5623], 26 Apr 1983, *Cedillo 2299* (MO); Dto. Pochutla, Río Concordia, [15.8963, -96.4871], 600 m, 13 Apr 1917, *Conzatti 3066* (MO); Mpio. Comaltepec, Sierra de Juárez, along Hwy 175 between km 66 and 67 just S of the town of Metates, 17.6860, -96.3289, 870 m, 11 Sep 2017, *Dean 9524* (DAV); Dto. Choapan, camino de Totontepec a Comaltepec, a 5 km al NE de Totontepec, [17.2803, -96.0087], 1850 m, 11 Nov 1983, *García-M. 4369* (MO); Mpio. Sta María Chimalapa. 0.5 km al E de Sta María, al SE de la vereda a Paso Lagarto, orilla del arroyo, 16.9083, -94.6667, 270 m, 28 May 1984, *Hérnandez-G. 26* (MO, NY); Mpio. Sta. María Chimalapa, cerca de Sta María, 16.9167, -94.6667, 300 m, 25 Jul 1984, *Hernández-G. 251* (MO); Dto. Pochutla, Río Concordia, 9 km al NE de la desviación al Cafetal Concordia, hacia Pluma Hdgo, la desviación está a 3 km de Chacalapa, [15.8362, -96.4881], 650 m, 26 Jun 1982, *Torres-C. 667* (NY); Dto. Juchitán, 4 km al W de la frontera Oaxaca-Chiapas, carretera Tapanatepec, 450 m, 27 Mar 1984, *Torres-C. 4808* (MO); **Tabasco**: Mpio. Tacotalpa. 0.2 km abajo (NW) de, y antes de entrar a Tapijulapa. Hasta 0.5 km arriba del camino por el arroyo a pie. Suelo derivado de lutitas, 30 May 1982, *Cowan 3507* (NY); Mpio. Tacotalpa. 0.2 km abajo (NW) de, y antes de entrar a Tapijulapa, [17.4703, -94.6256], 30 May 1982, *Cowan 3508* (NY); Mpio. Tenosique, 1.6 km (1 mi) arriba de la ruta Tenosique-Emiliano Zapata en el camino de la planta de cal que va a la torre de television, cerca al puentes Boca del Cerro, [17.4200, -91.4900], 270 m, 11 May 1984, *Cowan 4699* (MO, NY, TEX); vicinity of Teapa, along road between Teapa and Tacotalpa, 3.1 m. E of Teapa along stream and limestone cliffs, ca 1/4 mi S of highway, 17.5500, -92.9833, 150 m, 19 Feb 1987, *Croat 65351* (MEXU, MO); Mpio. Teapa, Sierra El Madrigal, al E del edificio principal del Centro Regional Tropical Puyacatengo, [17.5172, -92.9028], 600 m, 6 Jun 1991, *Hanan-Alipi 440* (MEXU); km 28.5 carretera Tenosique-Zapata, [17.5160, -91.5923], 7 Dec 1978, *González-Leija 307* (MEXU); Mpio. Tacotalpa, km 9 de la desviación de Teapa-Tacotalpa hacia Tapijulapa, 21 Nov 1983, *Magaña 1179* (MO); Tenosique, [17.4632, -91.4178], 14–16 Jun 1939, *Matuda 3449* (MEXU, MO, NY); Mpio. Teapa, Colecta en el Cerro del Madrigal subiendo por el oeste, [17.5190, -92.9206], 110 m, 10 Jan 1981, *Ramos 682* (MEXU); ladera kârstica al S de Los Azufres, 14 Feb 1984, *Rico 712* (MO); Mpio. Teapa, El Azufre, 30 m, 18 Jun 1984, *Ventura-A. 21059* (NY); **Veracruz**: Mpio. San Andres Tuxtla, Estación Biológica de Los Tuxtlas, Catemaco, [18.5830, -95.0731], May 1971, *Collector Unknown 438* (MO); Mpio Catemaco, Cerro Pipiapan, [18.4500, -95.0333], 400 m, 15 Jun 1986, *Acosta-P. 1216* (TEX); lado SE. de Laguna Catemaco, arriba del Río Cuetzalpan, Catemaco, 18.3833, -95.0167, 500 m, 20 Oct 1971, *Beaman 5140* (NY); Mpio. San Andrés Tuxtla, Estación de Biología Trop. Los Tuxtlas, [18.5845, -95.0742], 145 m, 18 Jun 1968, *Calderón 1706* (IEB, MO, NY, LL); Mpio. San Andres Tuxtla, Estación de Biología Tropical Los Tuxtlas, 18.5833, -95.0667, 110 m, 27 Jan 1978, *Calzada 4252* (IBUG, IEB); Mpio. Hidalgotitlán, Benito Juárez Segundo, 17.7833, -94.6500, 100 m, 2 Nov 1978, *Castillo 372* (NY); Estación Biológica, Sontecomapa, [18.5871, -95.0735], 60 m, 23 Dec 1968, *Cedillo 486* (MO, NY); Mpio. Hidalgotitlán, km 0–2 del camino Plan de Arroyos, Álvaro Obregón, 17.2500, -94.6667, 130–150 m, 15 Apr 1974, *Dorantes 2834* (MO); Mpio. Hidalgotitlán, km 0–2 camino Plan de Arroyo, Río Alegre, 17.2500, -94.5833, 140 m, 23 Apr 1974, *Dorantes 2969* (MO, NY); Mpio. Hidalgotitlán, camino Campamento Hermanos Cedillo, Plan de Arroyo, 25 Oct 1974, *Dorantes 3619* (MO); Mpio. Hidalgotitlán, camino Campamento Hermanos Cedillo, Plan de Arroyo, 17.2500, -94.6667, 140 m, 25 Oct 1974, *Dorantes 8265* (NY); Estación de Biología Tropical Los Tuxtlas. Transect 9, [18.5870, -95.0727], 170–200 m, 1 Jun 1981, *Gentry 32553* (MO); hills between Playa Escondida and Estación de Biología Los Tuxtlas, [18.5926, -95.0570], 50–100 m, 27 May 1981, *Gentry 32362* (MO); Estación Biol. Trop. Los Tuxtlas, 18.5000, -95.0833, 200 m, 9 Sep 1975, *Holstein 20362* (DAV); Mpio. San Andres Tuxtla, Lote 67, Est. Biol. Trop. Los Tuxtlas, [18.5871, -95.0732], 200 m, 8 Aug 1983, *Manriquez. 840* (MO); Mpio. San Andres Tuxtla, Estación de Biología Tropical Los Tuxtlas, Lote 67, [18.5667, -95.0667], 160 m, 24 Jun 1984, *Manriquez 1771* (MO, NY); Mpio. San Andrés Tuxtla, Lote 67, limite N, Estación de Biología Tropical “Los Tuxtlas,” [18.5667, -95.0667], 200 m, 26 Jun 1989, *Manriquez 3417* (MO); Mpio. De San Andrés Tuxtla, Estación de Biología Tropical “Los Tuxtlas” entre Montepio y Sontecomapan, [18.5871, -95.0731], 150 m, 13 Jun 1981, *Lorenae 3486* (MO); Estación de Biología de Los Tuxtlas, [18.5858, -95.0773], 27 Jun 1969, *Lot 329* (MO); Región de Los Tuxtlas, a los lados del camino hacia Laguna Escondida, [18.5921, -95.0841], 300 m, 1 Nov 1970, *Lot 971* (NY); Mpio. Jesús Carranza, 2 km al W. del campamento Hnos. Cedillo, 17.2667, -94.6167, 150 m, 9 May 1974, *Marquez-R. 201* (MO); same location, 5 Jul 1975, *Marquez-R. 241* (MO); same location, 2 Jul 1974, *Marquez-R. 256* (MO); same location, 19 Oct 1974, *Marquez-R. 275* (MO); Mpio. Pajapan, 5 km NW of Pajapan, SE slopes of Cerro San Martín Pajapan, 18.2917, -94.7167, 700 m, 3 Nov 1981, *Nee 22743* (MO); Mpio. Hidalgotitlán, 1 km SE of Agustín Melgar, 17.2500, -94.5500, 100 m, 2 Mar 1984, *Nee 29754* (MO, NY); between La Laguna and Uxpanapa, [17.2755, -94.4782], 18 Jul 1978, *Poole 1505* (TEX); Zacuapan, [20.4330, -98.3440], Jul 1915, *Purpus 7502* (NY); same location, [20.4330, -98.3440], Sep 1917, *Purpus 8064* (M, MO, NY); Mpio. San Andres Tuxtla, Estación de Biología Tropical de Los Tuxtlas, cerca de la Laguna Zacatal, [18.5858, -95.0773], 18 Sep 1982, *Ramamoorthy 4120* (NY); Estación de Biología de Los Tuxtlas, [18.5871, -95.0735], 17 Sep 1974, *Soto-E. 45* (NY); Mpio. Catemaco, Estación de Biología “U.V,” [18.5850, -95.0746], 500–650 m, 14 Sep 1998, *Torres-R. 13* (MEXU); Mpio. Catemaco, Cerro del Marinero, poblado Adolfo López Mateos, 18 km E de Catemaco, 18.4444, -94.9633, 500 m, 8 Jun 1991, *Torres 495* (MEXU); Mpio. Hidalgotitlán, Campamento Hnos. Cedillo, 17.2667, -94.6167, 152 m, 4 Jul 1974, *Vazquez 393* (NY); Mpio. Hidalgotitlán, brecha Hnos. Cedillo, La Escuadra, 17.3000, -98.6333, 162 m, 4 May 1974, *Vásquez 547* (MO, NY); Mpio. Tlaltetela, Limón, [19.3348, -96.8939], 400 m, 5 Jun 1976, *Ventura-A. 12809* (XAL); Mpio. San Andres Tuxtla, Estación Biol. Trop. Los Tuxtlas, slightly disturbed roadside 0.5 mi S of station hdq, 18.5000, -95.0833, 200 m, 11 Sep 1975, *Webster 20403* (DAV); Mpio. San Andres Tuxtla, Estación Biol. Trop. “Los Tuxtlas,” at Laguna Escondida, 18.3500, -95.0500, 300–400 ft, 20 Aug 1976, *Webster 20912* (DAV); Mpio. Hidalgotitlán, 4.5 km al S de Ejido Hidalgo Amajac, sobre el camino al Campamento La Laguna, 14.6 km al N del Poblado 10, cerca del camino, 17.4167, -94.4500, 170 m, 11 Jul 1980, *Wendt 2511* (MO).

**22. *Lycianthes hintonii***

**Mexico. Nuevo León**: Mpio. Aramberri, Cerro El Viejo, [23.9885, -99.7612], 1495 m, 3 Aug 1993, *Hinton 23263* (DAV, GBH, TEX, UC).

**23. *Lycianthes hypoleuca***

**Belize. Cayo**: vicinity of old lumber camp at Grano de Oro, [16.673, 89.026], 1700 ft, 2 Jun 1973, *Croat 23324* (BR, MO); vicinity of Cuevas S of Millionario, [16.6759, -89.0237], 1900 ft, 29–30 May 1973, *Croat 23552* (DAV, MO); same location and date, *Croat 23580A* (MO); same location and date, *Croat 23624* (MO); Río Frio Cave area near Augustine, [16.8188, -89.0069], 9 Jul 1972, *Dwyer 10195* (MO); at Millionario, on road toward Cuevas, , [16.7174, -89.0128], 1900 ft, 30 May 1973, *Dwyer 10780a* (MO); same location and date, *Dwyer 10790* (MO); Humming Bird Highway, 40 miles section, [17.1126, -88.9015], 30 Jun 1955, *Gentle 8776* (MO); Humming Bird Highway, 34 miles section, [17.1126, -88.9015], 1 Oct 1955, *Gentle 8887* (MO); Humming Bird Highway, 27 miles section, [17.0540, -88.5690], 31 Jul 1956, *Gentle 9196* (MO); Yucatán Peninsula, Vaca, [17.2587, -88.7591], 4 May 1938, *Gentle 2561* (MO, NY); S of Millionario, [16.7088, -89.0136], 1900 ft, 29 May 1973, *Gentry 7668* (BR, MO); vicinity of Millionario, [16.7167, -88.9833], 1800 ft, 30 May 1973, *Gentry 7696* (MO); Valentin, [16.7059, -89.1678], Jun–Jul 1936, *Lundell 6237* (MO, NY); Cahune Ridge, [16.8167, -89.0833], Jun–Aug 1936, *Lundell 6454* (NY); W of Terra Nova Forest Reserve, near Eligio Cocom farm, 13 miles N of Iguana Creek Bridge over the Belize River, entering by Black Man Eddy from the Western Hwy, 17.35, -88.9166, 100 m, 24 May 1996, *Nee 46818* (DAV); 3 miles S of Guacomallo Bridge, [16.9998, -89.0295], 650 m, 28 Aug 1980, *Sutton 302* (MO); Vaca Plateau, below Blue Hole Camp, [16.6772, -89.0923], 550 m, 8 Jun 1980, *Whitefoord 2046* (MO); New María Camp, [16.8272, -89.0167], 550 m, 25 May 1995, *Whitefoord 9461* (BR); **Orange Walk**: Coastal Region, Honey Camp, [18.0462, -89.4433], Sep 1929, *Lundell 343* (NY); Río Bravo Resource Management and Conservation Area, Chan Chich Area, [17.5466, -89.0389], 100–200 m, 24 Jul to 6 Aug 1988, *Brokaw 258A* (NY); **Toledo**: southwestern Maya Moutains, Columbia River Forest Reserve, Unión Camp, 16.3833, -89.1500, 700–750 m, 6 Apr 1992, *Holst 4125* (MO).

**Guatemala. Petén**: 2 mi S of entrance of Tikal National Park, [17.2107, -89.6247], 500 ft, 19 Jun 1973, *Croat 24707* (MO); ca. 5 miles S of entrance to Tikal National Park, [17.1793, -89.6380], 19 Jun 1973, *Croat 24743* (DAV, MO); same location and date, *Dwyer 11265* (MO); S of Tikal National Park, [17.2163, -89.6241], 19 Jun 1973, *Gentry 8333* (MO); Yaxha-Remate Road, [17.0284, -89.5273], Jun 1933, *Lundell 3986* (G); Tikal, on Temple VI road, [17.2218, -89.6227], 30 Apr 1959, *Lundell 15942* (MO); Tikal, on Temple VI road, [17.2218, -89.6227], 4 Jul 1959, *Lundell 16170* (MO); La Cumbre, in zapotal, Pusila road, on Porcela de Santiago Cuz, [16.0789, -89.3449], 12 Aug 1976, *Lundell 20138* (MO); alrededor del hotel de Tikal, en Parque Nacional de Tikal, [17.2279, -89.6108], 27 May 1970, *Tún-Ortíz 1146* (MO); Tikal, [17.2246, -89.6121], 600 m, 6 Jul 1976, *Bernhardt T7* (MO);

**Honduras. Comayagua**: Villa de Taulabé, [14.6689, -87.9894], 600 m, 12–13 Jun 1976, *Nelson 3563* (MO).

**Mexico. Campeche**: Mpio. Calakmul, N de Rancho El Sacrificio, camino a Nuevo Centro de Población Ejidal Ley de Fomento Agropecuario, 17.9897, -89.3944, 61 m, 5 Aug 1997, *Martínez-S. 28113* (NY); **Chiapas**: Mpio. Margaritas, Col. Maravilla, Tenejapa, [16.143018, -91.293515], 2 Jun 1986, *Méndez-G. 9067* (BR, MO); Mpio. Ocosingo, al SW de Sto. Domingo, [17.0458, -91.4317], 30 Jul 1982, *Quintanilla 14* (MO3657679); **Quintana Roo**: Mpio. Tomás Garrido, a 3 km al N de Estero Franco, sobre el camino en construcción a Tomás Garrido, [17.9544, -88.8831], 31 Jul 1982, *Cabrera-C. 3279* (MO, NY); Mpio. Tomás Garrido, a 12 km al S de San José de la Montaña, sobre el camino a Tomás Garrido, [18.2667, -89.0351], 13 Jul 1983, *Cabrera-C. 5151* (MO); a 66 km al S de Ucum, o a 22 km al N de la Unión, [18.0456, -88.7980], 13 Jul 1983 *Cabrera-C. 5187* (NY); a 2 km al N de Estero Franco, sobre la carretera La Unión-Ucum, [17.9512, -88.8769], 20 Aug 1983, *Cabrera-C. 5444* (MO, NY); Mpio. Tomás Garrido, en la Unión, [17.8972, -88.8806], 4 Sep 1984, *Cabrera-C. 7203* (NY).

**24. *Lycianthes inconspicua***

**Costa Rica. Alajuela**: Bella Vista de Lancero, Caribbean watershed, 1525 m, 9 Feb 1939, *Smith 1597* (NY); **Cartago**: Cerro La Carpintera, immediately N of Tres Ríos and E of San José, 1600–1800 m, 1 Jun 1984, *Taylor 3924* (DUKE); **Puntarenas**: Monteverde, 1250 m, 18 Jan 1981, *Haber 431* (MO); Monteverde, lower community, 1300 m 29 Nov 1984, *Haber 1068* (MO); at waterfall on Queb. Cuecha along Sendero El Río, Monteverde Cloud Forest Reserve, 10.4763, -84.7916, 1600 m, 27 Jul 1988, *Hayworth 201* (DAV, WIS).

**Guatemala. San Marcos**: 1 mile above Africa, ca. 3.3 miles above Finca Armenia above San Rafael, 1600 m, 13 Jul 1977, *Croat 40950* (MO, NY); Finca Armenia, Rafael de Cuesta, San Marcos, 5000 ft, 6–7 Jul 1977, *Dwyer 14408* (LL, MO); **Suchitepéquez**: Mpio. San Francisco Zapotitlán. Reserva Natural Privada Las Nubes, 14.7061, -90.9744, 1600 m, 3 Jul 2014, *Escobar 171* (BIGU).

**Panama. Chiriqui**: ridge road at Cerro Colorado 7.6 km from main road, 1450–1750 m, 15 Aug 1977, *Folsom 4821* (G, MO, NY); along trail between Bajo Charro and Bajo Mona, 7300 ft, 13 Apr 1979, *Hammel 7035* (MO, NY); Distr. de Bugaba, Santa Clara, Hartmann’s Finca, 8.8333, -82.7333, 1300 m, 26 Feb 1985, *van der Werff 7141* (MO); Distr. de Bugaba, Santa Clara, summit of Cerro pando, 8.8333, -82.7333, 1300 m, 28 Feb 1985, *van der Werff 7205* (NY).

**25. *Lycianthes jalicensis***

**Mexico. Jalisco**: 24 km by road S of Autlán on road to Barra de Navidad, [19.6781, -104.4216], 760 m, 19 Sep 1983, *Anderson 12717* (CAS, IBUG, IEB, NY); near Puerto el Triumfo, 19 km WSW of Talpa de Allende along road to La Cuesta and Tomatlan, [20.2489, -104.9276], 1555 m, 11 Sep 1986, *Breedlove 64125* (MEXU, NY); Mpio. Casimiro Castillo, en la Calera, entre Puerto Los Mazos y el Tigre, [19.6698, -104.4364], 600–800 m, 24 Sep 1988, *Cházaro-B. 5674* (CAS, WIS, XAL); Mpio. Casimiro Castillo, subiendo a el Cerro de La Petaca, despues de cruzar el arroyo Tacubaya, [19.5966, -104.4305], 25 Sep 1988, *Cházaro-B. 5681* (IBUG, IEB, MEXU); 5.6 km por camino al W de Puerto Los Mazos, en el lado N del “Cerro Autlán,” 10 km distancia aérea al N de Casimiro Castillo, 19.6886, -104.4378, 1050 m, 6 Jan 1985, *Cochrane 10849B* (GH, US, WIS); along highway 200 between Puerto Vallarta and Tuito and vicinity of large bridge ca. 19 km south of Puerto Vallarta, [20.5076, -105.3028], 150 m, 9 Jan 1979, *Croat 45421* (MO); same location and date, *Croat 45423* (MO); Mpio. Casimiro Castillo, Sierra de Manantlán, 6–8 km al N de C. Castillo, 19.6541, -104.4375, 800–1000 m, 5 Dec 1987, *Cuevas 2902* (WIS); Casimiro Castillo, 19.6119, -104.4255, 400–500 m, 25 Sep 1988, *Cuevas-G. 3205* (WIS); Mpio. Casimiro Castillo, 19–20 km al sur-suroeste de Autlán, 2–5 km al sureste de Casimiro Castillo, 19.5861, -104.4036, 700–1000 m, 15 Jul 1988, *Cuevas-G. 3131* (BRIT); S of Puerto Vallarta and N of El Tuito, along hwy 200, 20.3 road km S of Playa Mismaloya, W side of the road, along footpath that follows small drainage, [20.3806, -105.3102], 500 m, 27 Nov 1991, *Dean 331*, (DAV, UC, XAL); pasando el puente del Río Las Juntas carretera Chamela-Puerto Vallarta, cerca de El Tuito, [20.3197, -105.3233], 450 m, 27 Sep 1976, *Delgado-S. 192* (MO); “Arroyo de Tacubaya,” at base of “Cerro La Petaca,” 1–4 km E of Casimiro Castillo, ca. 20 km SSW of Autlán, 19.6167, -104.4333, 350–500 m, 19 Sep 1978, *Iltis 305* (WIS); S- and SE-facing midslopes and shoulders of “Cerro La Petaca,” along trail to El Durazno, 5–10 km ESE of Casimiro Castillo, [19.5658, -104.3999], 900–1100 m, no date, *Iltis 348* (WIS); 1 km W of El Divisadero (1 km W of Villa Guerrero), 18 km ESE of Tomatlan, 19.8619, -105.1656, 14 Jan 1979, *Iltis 1637* (WIS); Next to Mexico 80 on N-facing slope of Sierra de Autlán (but SW of Sierra de Manantlán, NW-end), ca. 1.5 km W of Puerto Los Mazos, 9 km (by air) SSWof Autlán, 19.70, -104.4166, ca. 1350 m, 20 Jun 1984, *Iltis 29181* (CAS, MEXU, MO, NY); Reserva Biosfera Sierra de Manantlán, La Calera, just NW of km 188 marker on Autlán-Manzanillo hwy (Mex. 80), 9 km (by air) NNE of La Resolano (Casimiro Castillo) and ca. 16 km SSE of Autlán, [19.6777, -104.4083], 800–1100 m, 10 Mar 1992, *Iltis 31037* (DAV, WIS); same location and date, *Iltis 31058* (WIS); a 30 km al S de Puerto Vallarta, ca de las Juntas, 28 Oct 1985, Lott 2715 (MEXU); Mpio. Villa Purificacion, Rancheria Los Achiotes, al oeste de Villa Purificacion, siguiendo el río hacia Los Herbores, [19.6630, -104.7865], 360 m, 17 Aug 1990, *Martínez-Mayorga 55* (IEB); Mpio. Talpa de Allende, 17 km sur-suroeste de Talpa de Allende, 11 km sur-suroeste Aranjuez, 0.4 km S de estación microondas, 20.2894, -104.9038, 1740 m, 1 Aug 1994, *Medina 99* (IEB); Quimixto, trail from San Pedro el Tuito, [20.5088, -105.3765], 60 m, 2 Dec 1926, *Mexia 1243* (G, UC); Santa Cruz de Vallarta, [20.5811, -105.2197], 700 m, 11 Dec. 1926, *Mexia 1286* (CAS); Mpio. Cabo Corrientes, El Tuito, 2 km al N del Tuito, por la carretera 200, [20.3327, -105.3112], 650 m, 23 Sep 1989, *Ramírez-Delgadillo 1627* (IBUG, IEB, MEXU); Mpio. Casimiro Castillo, arroyo El Tigre, 4 km al NE de El Zapotillo, [19.6876, -104.3976], 1600 m, 11 Oct 1986, *Santana-M. 2638* (IBUG, IEB); Mpio. Casimiro Castillo, 2 km SE de Casimiro Castillo, Cerro La Petaca, 19.5877, -104.4133, 600 m, 8 Jul 1992, *Santana-M. 5773* (WIS); Mpio. Casimiro Castillo, 15 km al SW de Autlán, Barranca del Tecolote, 19.6222, -104.3875, 800–900 m, 22 Jul 1987, *Vázquez 4521* (WIS); SW of Autlán, rd. to Barra de Navidad (hwy. 80), 19.6666, -104.4167, 2500–2600 ft, 28 Oct 1970, *Webster 16053* (DAV).

**26. *Lycianthes limitanea***

**Belize. Toledo**: beyond Unión Camp, Edwards Road, beyond Columbia, [16.3359, -89.1483], 13 Mar 1948, *Gentle 6470* (LL, MO).

**Guatemala. Huehuetenango**: Nentón, along the road from Nuevo San José Frontera to Las Palmas, 16.0333, -91.5500, 900–1200 m, 17 Mar 2009, *Christenhusz 5619* (NY); San José Maxbal-Barillas (camino), Barillas, 20 Sep 2006, *Pérez 1363* (MO); **Izabal**: Puerto Méndez, on Río Dulce Road, 4 km, [15.8660, -89.2119], 15 Jun 1970, *Contreras 10068* (MO, LL); El Estor, [15.5291, -89.3368], 5 Mar 1972, *Contreras 11151* (MO, LL); 1.4 km N of San Felipe, [15.6496, -89.0088], 80 m, 12 Mar 1970, *Harmon 2083* (MO); along road, Vic. EXMIBAL Camp 1 (Sepos), NW of Lake Izabal, [15.2500, -89.0000], 500–600 m, 13 May 1966, *C. Jones 3402* (NY); El Estor, La Llorona, 15.5142, -89.4236, 500 m, 30 Aug 1998, *Véliz 98.6652* (BIGU); **Petén**: on Melchor de Mencos Road, 8 May 1967, *Contreras 6873* (MO, NY, LL);

**Mexico. Chiapas**: Mpio. Palenque, 6–12 km S of Palenque on road to Ocosingo, [17.4468, -91.9623], 300 m, 10 May 1973, *Breedlove 35007* (MO); Mpio. La Trinitaria, Lagunas de Montebello National Park, along road to Cinco Lagunas, [16.1076, -91.6796], 1380 m, 29 Jan 1981, *Breedlove 49707* (MO); Mpio. La Trinitaria, between Lago Tziscao and Dos Lagos, Lagos de Montebello National Park, [16.0936, -91.6381], 1372 m, 13 Oct 1981, *Breedlove 53462* (MO, LL); a 15 km al E de las Lagunas de Monte Bello, [16.1086, -91.5641], 1 Mar 1982, *Cabrera-C. 1857* (MEXU); a 10 km al E de Tziscao, sobre el camino a Santa Elena, [16.1069, -91.5942], 5 Dec 1983, *Cabrera 6091* (MO, WIS); Mpio. Ocozocoautla de Espinosa, Cerro la Colmena, al NE del rancho Corocito, [16.7647, -93.6439], 1350 m, 27 Apr 1983, *Calzada 9622* (XAL); Mpio. La Trinitaria, Parque Nacional Lagos de Montebello, Lago de Montebello, 16.1083, -91.7000, 1500 m, 2 May 1985, *Espejo 1616* (MO); Mpio. Ocosingo, en Nuevo Chihuahua, a 28 km al N del Vertice del río, Chixoy camino a Boca Lacantum, [16.0822, -90.6817], 130 m, 12 Jan 1986, *Martínez-S. 16185* (MO, NY).

**27. *Lycianthes manantlanensis***

**El Salvador. Ahuachapán**: Lago de Ninfas, Cordillera de Grande de Apaneca, NW of Juayua, 13.8747, -89.7986, 1600 m, 6 Feb 1998, *Davidse 37395* (MO, MSB); orilla de la Laguna Las Ninfas, 13.8833, -89.8000, 1756 m, 5 Feb 1998, *Renderos 438* (MO, NY); Laguna de las Ninfas, Apaneca, [13.8786, -89.7969], 4 Mar 1991, *Reyna 1492* (MO); Cerro Campana, 13.8500, -89.9167, 1265 m, 23 Oct 1979, *Witsberger 750* (MO); **Santa Ana**: Mpio. Chalchuapa, alrededores de la Laguna de Las Ranas, 13.9000, -89.7167, 1750 m, 19 Dec 1999, *Linares 4702* (MEXU); Cerro El Aguila, calle a la cumbre, 13.8333, -89.6833, 1654 m, 18 Feb 2007, *Rodríguez 736* (MEXU); Finca Pinón on Cerro de Los Naranjos, Volcán Santa Ana, [13.8560, -89.6581], 1670 m, 16 Jan 1949, *Williams 15147* (GH); **Sonsonate**: Mpio. Juayúa, 24 km al SO de la Cuidad de Santa Ana, 13.8667, -89.6861, 1600 m, 2 Apr 1995, *Linares 2499* (MO); Mpio. Juayúa, Finca El Olimpo, 13.8989, -89.6947, 1850 m, 19 Nov 2007, *Linares 12751* (MEXU); Cerro El Pilón, Sierra Apaneca, [13.8736, -89.6844], 1800 m, 23 Feb 1968, *Molina-R. 21616* (NY).

**Guatemala. Chimaltenango**: Panajabal, [14.7347, -90.9347], 1350 m, 5 Jan 1939, *Standley 62136* (G); **Quetzaltenango**: Slopes of Volcán de Zunil, at and above Aguas Amargas, [14.7541, -91.4707], 2430–2850 m, 17 Feb 1939, *Standley 65304* (NY, US); Aguas Amargas, on the western slope of Volcán de Zunil, [14.7934, -91.4754], 2450 m, 14 Jan 1941, *Standley 83341* (US); along road between Finca Pirineos and Patzulín, [14.6946, -91.5415], 1200–1400 m, 9 Feb 1941, *Standley 87013* (US); same location and date, *Standley 87146* (US); Montaña Chicharro, lower SE-facing slopes of Volcán Santa María, 2–4 miles south of Santa María de Jesús, [14.6920, -91.5429], 1400–1500 m, 17 Jan 1940, *Steyermark 34272* (US).

**Mexico. Chiapas**: Mpio. Villacorzo, Cerro La Peña, al O del ejido Sierra Morena, 16.1642, -93.6022, 1450 m, 19 Oct 2002, *Alvarado-C. 575* (DAV); same location and date, *Alvarado 622* (MEXU); Mpio.Angel Albino Corzo, above Finca Cuxtepec, [15.7664, -92.9550], 1380 m, 21 Oct 1980, *Breedlove 46701* (MO); same location, 14 Dec 1980, *Breedlove 48651* (DAV); same location, 10 Jan 1982, *Breedlove 56926* (TEX); Mpio. Motozintla de Medoza, steep canyon, SW side of Cerro Mozotal, 11 km NW of the junction of the road to Motozintla along the road to El Porvenir and Siltepec, [15.4000, -92.3000], 2100 m, 23 Nov 1981, *Breedlove 55762* (CAS); Mpio. Acacoyagua, 5 km del camino Ejido Las Golondrinas a Rosario Sacatonal, 15.4436, -92.6856, 1400 m, 9 Mar 2006, *Martínez-Camilo 936* (MO); Mt. Ovando, [15.4100, -92.6100], 2200 m, 14–18 Nov 1939, *Matuda 3981* (A, NY); **Guerrero**: 33.3 km SW of Filo de Caballo, on Chilpancingo-Atoyac road, [17.6405, -99.7304], 2000 m, 7 Nov 1999, *Yahara 1925* (MEXU); **Michoacán**: Mpio. Tumbiscatío, District: Coalcoman. Naranjillo, [18.7624, -103.1397], 1400 m, 2 Aug 1941, *Hinton 15942* (F, G, NY, W); **Oaxaca**: Mpio. San jeronimo Coatlan, 19.2 km al suroeste de San Jeronimo Coatlan, brecha a Piedra Larga, 16.2000, -96.9667, 1900 m, 13 Aug 1988, *Campos-Villanueva 2246* (MEXU); Mpio San Jeronimo Coatlan, 26.7 km al suroeste de San Jeronimo, brecha a Piedra Larga, 16.1833, -97.0000, 1700 m, 2 Dec 1990, *Campos-Villanueva 3514* (MEXU); Mpio. San Jeronimo Coatlan, 22 km al suroeste de San Jeronimo Coatlan, brecha a Piedra Larga, [16.0928, -97.0199], 1750 m, 2 Jun 1992, *Campos-Villanueva 4626* (MEXU); Mpio. Santa María Chimalapa, Cabecera de Arroyo de las Señoritas, ca. 7 km en línea recta al NO de Benito Juárez, ca. 43 km en línea recta al N de San Pedro Tapanatepec, 16.7500, -94.1833, 1300–1700 m, 26–27 Oct 1985, *Maya-J. 2413* (MEXU); Mpio. Santa María Chimalapa, cañada al N de Cerro de la Leona (cerro al NE de Cerro Quetzal y ca. 7–9 km al N de Cerro Guayabitos) ca. 46 km en línea recta al N de San Pedro Tapantepec, 16.7833, -94.1833, 1300 m, 28 Feb 1987, *Maya-J. 4226* (MEXU).

**28. *Lycianthes mariovelizii***

**El Salvador. Sonsonate**: Laguna Verde, 13.90, -89.7833, 1650 m, 26 Jan 1999, *Renderos 548* (MO).

**Guatemala. Huehuetenango**: Mpio. Nentón, en la orilla del Río Nentón, en el sumidero, 15.8014, 91.7542, 800 m, 1 Nov 1995, *Castillo 2660* (F, MO, NY); Paso del Boquerón, Río Trapichillo, below La Libertad [15.5174, 91.8385], 1200–1300 m, 21 Aug 1942, *Steyermark 51177* (F); Mpio. Santa Ana Huista, Aldea El Tabacal, 15.6949, 91.8721, 753 m, 15 Aug 2017, *Véliz 26106* (BIGU, DAV); Mpio. Santa Ana Huista, Aldea El Tabacal, 15.6949, 91.8721, 753 m, 15 Aug 2017, *Velásquez 5381* (BIGU); **Progreso**: a 2 km al N de los Leones, camino El Rancho-Cobán [14.95, 90.2], 700 m, 4 Aug 1988, *Martínez-S. 23237* (MEXU, MO, NY).

**Mexico. Chiapas**: Mpio. Frontera Comalapa, along road to Ciudad Cuauhtémoc, 6–8 km E of Frontera Comalapa [15.6916, 92.0871], 1000 m, 15 Aug 1972, *Breedlove 27009* (CAS, MEXU, MO); Mpio. Frontera Comalapa, along road to Ciudad Cuauhtémoc, [15.6916, 92.0871], 1000 m, 23 Oct 1974, *Breedlove 39087* (CAS; MO). **Oaxaca**: Mpio. San Miguel del Puerto, en el cafetal Arroyo Arena, a orilla del río a 1.47 km del Rancho Dioon, 15.9777, 96.1005, 16 Nov 2003, *Nava-Z.* 205 (DAV).

**Nicaragua. Nueva Segovia**: Mpio. El Jícaro, Monte Rico, 5 km al NE de El Jícaro, 13.7333, -86.0833, 705 m, 3 Sep 1984, *Moreno 24615* (MO).

**29. *Lycianthes michaelneei***

**Mexico. Veracruz**: Mpio. Calcahualco: Barranca de Atotonilco, arriba del poblado de Atotonilco, [19.1308, -97.0915], 2450 m, 14 Feb 1982, *Cházaro-B. 29-3293* (WIS); Mpio. Ayahualulco, parte alta Cerro Coatepec, a medio camino entre Ayahualulco y Patlanalan, [19.3236, -97.0763], 2500–2600 m, 23 Aug 1985, *Cházaro-B. 29-3701* (WIS, MEXU, XAL); San Miguel Tlacotiopa, camino a Nueva Vaquería, Barranca Cuapa, 19.10, -97.2333, 2600 m, 30 Jul 1985, *Martínez 525* (MEXU, XAL); 500 m al SW de Maquistla, camino a Jacal, 19.1166, -97.1833, 2200 m, 30 Jul 1985, *Martínez-G 538* (MEXU, XAL); 1 km al S de Escola, 9.1166, -97.1333, 1950 m, 24 Jul 1986, *Martínez 1227* (NY, XAL); Mpio. Cuitlahuac, Cuitlahuac, 17 May 1937, *Matuda 1513* (US); Mpio. Acajete, Plan de Cedeño, [19.5710, -97.0027], 1750 m, 4 Jun 1981, *Ventura-A. 18551* (IEB, XAL).

**30a. Lycianthes moziniana var. moziniana**

**Mexico. State Unknown and no exact location**: 1833, *Andrieux 60* (G); *H. Buek s.n.* (HBG); Lerma, 3 Jul 1959, *Davis s.n.* (TEX); Herb. Jan (W); Aug 1840, *Galeotti 1185* (BR); hab. in Mexico ad Guayconalpo, *Hage s.n*, 1906 (HBG); *Karwinski s.n.* (M); Cumbre de Estepe, 1842, *Liebmann 1450* (C); *Pavon s.n*, actually Sessé and Mociño specimens (G); *Schmitz s.n.* (W); *Schmitz s.n.* (GH); *Schmitz 484* (BM, F, NY, W); 1788–1803, *Sessé & Moçiño s.n.* (BM, photo WIS; according to M. Nee, specimen is annotated by Dunal); *Sessé & Moçiño s.n.* (BM);1787–1795–1804, *Sessé, Moçiño, Castillo et Maldonado 1529* (photo NY – of Madrid Specimen – F negative 48228; another photo at NY of a duplicate specimen is *L. ciliolata*); 1787–1795–1804, *Sessé, Moçiño, Castillo et Maldonado 1530* (F); 1787–1795–1804, *Sessé, Moçiño, Castillo et Maldonado 5388* (F). **Dto. Federal**: Pedregal, 7000 ft, 30 Jun 1938, *Balls B5207* (BM, K, UC); cultivated fields in the hills of the Pedregal by Zapan, [19.2781, -99.2467], 26 Jun 1865, *Bourgeau 351* (BR, G, GH, L, S); foothills, SE Mexico City, [19.3296, -99.0092], 28 Jun 1935, *Clark 7352* (MO, NY); Cañada de Contreras, [19.2640, -99.2721], Sep 1927, *Lyonnet 177* (BM, GH, K, MEXU, MO, NY); C. Cuatepec, S. Guadalupe, Valle de Mexico, [19.5907, -99.1209], 2800 m, 15 Jul 1951, *Matuda 21226* (MEXU); Valle de Mexico, Santa Fe-Cuajimalapa, [19.3970, -99.2369], 2450 m, 12 Aug 1951, *Matuda 21431* (MEXU); Valle de Mexico, Cuajimalpa a Río Hondo, [19.4315, -99.2919], 2400 m, 9 Sep 1951, *Matuda 21819* (MEXU); Valle de Mexico, Cerro Guarda, [19.1219, -99.1700], 2600 m, 13 Jul 1952, *Matuda 26205* (MEXU); Cerro Tianguillo, Sierra de Guadalupe, al N de la ciudad de Mexico ,[19.5762, -99.1206], 6 Aug 1959, *Paray 2934* (MEXU); Mpio. Huixquilucan, fraccionamiento “La Herradura,” [19.4193, -99.2457], 2800 m, 8 Jul 1968, *Perusquía-O. s.n.* (F); Santa Fe, [19.3895, -99.2229], Jul 1905, *Rose 8617* (NY); lava beds near Eslava, [19.2907, -99.2519], 7500 ft, 19 Jul 1910, *Rusby 131* (NY); 2 km al N de la Cabrera, delegación de Cuajimalpa, [19.3667, -99.2833], 2500 m, 4 Aug 1971, *Rzedowski 28358* (ENCB, F, IEB, MEXU); Valle de México, [19.5167, -99.2500], 1875, *Schaffner 551* (GOET); Loma de la Era, delegación de Alvaro Obregón, [19.3217, -99.2626], 2650 m, 11 Jun 1977, *Ventura-A. 2832* (ENCB, F, MEXU); Limbo, delegación de Alvaro Obregón, [19.3247, -99.2633], 2700 m, 4 Aug 1979, *Ventura-A. 3477* (ENCB, F, MEXU); road from Pedregal to Ajusco, 8000 ft, 25 Jun 1976, *Wonderly 275* (NY); **Durango**: 1 km al noroeste de Santa María Ocotan, Mezquital, [24.1000, -104.6000], 13 Jul 1984, *González-Espinosa 1378* (MEXU); **Hidalgo**: Mpio. Acatlan, Los Reyes, ca. 9 rd mi from Acatlan along rd to Huasca, NW of Tulancingo, [20.1888, -98.4489], 2195 m, 21 Sep 1991, *Dean 259* (DAV, NY, XAL); Agua Blanca de Iturbide, 20.3333, -98.3500, 2100 m, 9 Jul 1972, *Gimate-L. 690* (F, IEB); Mpio. Acatla, 9 km al N de Acatlán, hacia Huasca, 2300 m, 11 Aug 1981, *Hernández-M. 6329* (MEXU); Zimapán, [20.7500, -99.3667], 25 Aug 1938, *Kenoyer A305* (F); Samariel to Las Lajas, [20.1167, -98.7167], Aug 1905, *Rose 9218* (GH, NY); **Jalisco**: Rancho “La Joya Grande,” 10 km al N de Zapotlanejo, 1600 m, 7 Jul 1977, *Carvajal 641* (WIS); El huerto el Zarzamoro, Las Joyas, 19.6208, -104.2561, 1900 m, 15 Jul 1986, *Cuevas-R. 1391* (WIS); Huerto el Zarzamoro, Las Joyas, Autlán, [19.6208, -104.2561], 1900 m, 16 Jul 1986, *Cuevas-R. 1420* (WIS); Mpio. Tapalpa, Juanacatlan, [20.1000, -103.7500], 2561 m, 7 Aug 1991, *Dean 246* (DAV, XAL); Mpio. Jocotepec, near town of Zapotitan de Hidalgo, cultivated field in valley at the base of Cerro Viejo, [20.3307, -103.4798], 1605–1620 m, 11 Aug 1991, *Dean 247* (DAV, XAL); Mpio. Mazamitla, Sierra del Tigre, ca. 4.6 rd mi along rd to El Corral de la Mula, S of Mazamitla, rd leaves hwy 110 at Puerto Zapotería and winds into the mountains, [19.8981, -103.0642], 1982–2012 m, 22 Nov 1991, *Dean 328* (DAV, MEXU, NY, XAL); Mpio. Jocotepec, Cerro Viejo, area opposite the Presa de Chayote, [20.3477, -103.3885], 2200 m, 24 Nov 1991, *Dean 330* (DAV, UC, XAL); Mpio. Venustiano Carranza, along rd between Ciudad Guzmán and El Grullo, rd to microondas Las Víboras, near Puerto el Floripondo, [19.6333, -103.6167], 2317 m, 1 Dec 1991, *Dean 333* (DAV, MEXU, NY, UC, US, XAL); Rancho el Isote, just W of Sayulapan, along rd between El Grullo and Ciudad Guzmán, along dirt rd that enters on S side of road, [19.6500, -103.6333], 2012 m, 3 Dec 1991, *Dean 335* (DAV, MEXU, NY, UC, XAL); Mpio. El Mirador, Cocula (Colula), [20.3167, -103.8500], 1800 m, 16 Jul 1976, *Delgado-S. 262* (MEXU); Mpio. Bolaños, 24 km sobre el camino a Los Amoles a partir de Bolaños, [21.9306, -103.8742], 2400 m, 24 Jul 1991, *Flores-C. 470* (IEB); Mpio. Bolaños, 5 km al oeste del crucero Tuxpan de Bolaños-Los Amoles “Vanderitas,” 21.8922, -103.8764, 2390 m, 30 Jun 1996, *Flores-F. 4601* (MEXU); Mpio. Bolaños, 3.5 km al suroeste de la desviacion Los Amoles-Tuxpan de Bolaños, 21.9108, -103.8833, 2370–2460 m, 16 Jul 1997, *Flores-F. 4850* (GH, MEXU); 1–3 km al suroeste de las Berengenas, en el Arroyo Hondo, hasta entronca con el Arroyo La Mulera, 21.8500, -103.9089, 1850 m, 18 Jul 1997, *Flores-F. 4882* (K, MEXU); Mpio. Tlajomulco de Zuñiga, al suroeste de San Sebastian, fraccionamiento Jardines de Verano, [20.5342, -103.4406], 1500 m, 16 Jul 1991, *Huerta 102* (IBUG, IEB, MEXU); Mpio. Volcánes, La Campana, [20.3833, -104.5833], 1800 m, 29 Aug 1968, *Díaz-L. 1386* (IBUG, F); steep S-facing slope above “Salto” of Río Tapalpa, 2 km NW of Tapalpa, [19.9333, -103.7833], 1800–2000 m, 7 Aug 1960, *Iltis 797* (MEXU, WIS); Tepotitlán, 5 mi E. of Roadside, [20.8500, -102.7667], 18 Jul 1960, *Knoblock 1652* (WIS, MEXU); Mpio. Jocotepec, Cerro Viejo, [20.3601, -103.4451], 2380 m, 19 Jul 1986, *Machuca-N. 2781A* (TEX, WIS); Mpio. Tenamaxtlan, Presa del Durazno, al NE de Tenamaxtlan, [20.2167, -104.1167], 7 Aug 1994, *Machuca-N. 7164* (IBUG, IEB); Mpio. Chiquilistlan, camino Chiquilistlan-Tapalpa, al S de Capula, 20.0600, -103.8732, 24 Aug 1997, *Machuca-N. 8110* (IEB, MEXU); Sierra del Tigre, 3 mi S of Mazamitla, [19.8760, -103.0181], 2100–2200 m, 19 Sep 1952, *McVaugh 13066* (BM, MEXU, NY, TEX); N slopes of the Nevada de Colima, W of summit of the N ridge, near junction of the old pack road to Zapotlán with Atenqique-Jazmín rd, [19.6000, -103.6833], 2100 m, 20 Jul 1957, *McVaugh 14941* (MEXU); Sierra del Halo, near a lumber road leaving the Colima hwy 7 mi SSW of Tecalitlán and extending SE toward San Isidro, 2 mi from hwy, [19.3151, -103.2514], 1400 m, 13 Aug 1957, *McVaugh 16154* (DUD, MEXU, NY, TEX; a duplicate specimen at TEX is *L. acapulcensis*, WIS); along Hwy 110, km 7–8; open pine forest between Mazamitla and Tamazula, [19.8937, -103.0737], 4 Aug 1965, *Mertz 180* (MEXU); Río Blanco, [20.7833, -103.3167], July 1886, *Palmer 186* (BM, G, GH, IEB MEXU, MO, NY, US, WIS, WU); banks of ravines near Guadalajara, [20.7500, -103.2833], 5000 ft, 14 Jul 1902, *Pringle 11317* (GH, K, L, MEXU, WIS); Mpio. Mezquitic, 1 km al suroeste de Nueva Colonia, Santa Catarina, [22.1554, -104.1165], 2200 m, 20 Jul 1992, *Reynoso-Dueñas 929* (IBUG, IEB, MEXU); Mpio. Venustiano Carranza, Sayulapa, faldas del Nevado de Colima, [19.6333, -103.4333], 2100 m, 22 Aug 1987, *Rodríguez-C. 941* (MEXU, WIS); Carr. Mazamitla-Tamazula de Gordiano, cerca del Rancho “El Ternero”, [19.9000, -103.0667], 2100 m, 21 Jul 1988, *Rodríguez-C. 1326* (IEB, MEXU, WIS); Mpio. Amacueca, 2 km al suroeste de Lagunillas por la carretera a Tapalpa, apartir de la carretera Guadalajara-Sayula, 19.9333, -103.6667, 1950 m, 18 Jul 1995, *Rodríguez-C. 2682* (IBUG, MEXU); Mpio. Zapopan, km 13.5 de la brecha prolongacion Maríano Otero-Planillas, 2 km suroeste del rancho Los Cerezos, 20.6000, -103.5167, 1850 m, 27 Jul 1995, *Rodríguez-C. 2690* (BM, IBUG, MEXU); Mpio. Tapalpa, km 40–41 de la carretera Zacoalco de Torres-Tapalpa via Atemajac de Brizuela, 16 km al N de La Frontera, 20.0756, -103.7079, 2280 m, 21 Aug 2005, *Rodríguez-C.4400* (BM, G, IBUG, IEB, MEXU); Mpio. Tapalpa, 3 km al NE de Tapalpa frente al crucero de San Francisco y Los Espinos, 20.0667, -103.7000, 2200 m, 21 Aug 2005, *Rodríguez-C. 4408* (GH, IBUG, IEB, WU); Cerro Alto, al noroeste de Ixtlahuacan del Río, 20.9500, -103.2833, 2000–2300 m, 6 Jul 1986, *Villareal de Puga 2560* (IBUG, IEB); 42 miles E of Guadalajara, 18 Aug 1959, *Waterfall 15636* (BRIT); trail between Chante and Manantlán about 15 mi SSE of Autlán, [19.5879, -104.2307], 4500 ft, 1 Aug 1949, *Wilber 2081* (DS, IEB, MEXU, WIS); **México**: Mpio. Ixtapaluca, km 42 carretera Mexico-Puebla, [19.3303, -98.7917], 2650 m, 6 Sep 1970, *Aguirre J. 4* (IEB, MEXU, MO); near Toluca, [19.3015, -99.6745], Apr 1834, *Andrieux 196* (G-DC, photos: F, GH, MO, NY, WIS); 1.5 km al E de colonia Gustavo Baz, 19.0319, -99.4000, 2600 m, 20 Jul 1990, *Gómez-Barrera 58* (IEB); San Cayetano, ca. 1.5 rd mi from old hwy 15 along a gravel rd that begins ca. 3.4 rd mi E of turnoff to Villa de Allende, NW of Valle de Bravo, [19.4167, -100.1500], 2515 m, 11 Nov 1991, *Dean 319* (DAV); Mpio. Villa de Allende, 5 km al N de Agua Escondida (Criadero de San Cayetano), [19.4018, -100.1653], 2500 m, 1 Aug 1982, *Diaz-P. 245* (ENCB, IEB); Amecameca, 26 Jul 1924, *Fisher s.n.* (ARIZ, F, MO); Mpio. Texcoco, lado sur de la Cañada de Aguas, 13.5 km al SE de Texcoco (11 km al ESE de Coatlinchán), [19.4219, -98.8019], 2740 m, 29 Jul 1977, *García-P. 343* (CAS, MEXU); Mpio. Tepetlaoxtoc, 18 km por carr. Texcoco-Calpulalpan, limite entre Edo. México y Tlaxcala, [19.5590, -98.7148], 2870 m, 3 Sep 1982, *García-P. 1690* (MEXU); District Temascaltepec, Comunidad, [19.1322, -99.9176], 2610 m, 7 Jul 1932, *Hinton 972* (BM, G, K); 30 mi E of Mexico City on the road to Puebla, [19.3168, -98.7196], 8500 ft, 9 Jul 1940, *Hitchcock 7013* (DS, F, GH, UC); Mpio. Jilotepec, Jilotepec, [19.9500, -99.5333], 2300 m, 21 Sep 1953, *Matuda 28021* (MEXU); Cumbre de Acambay, [19.9500, -99.8333], 3000 m, 9 Aug 1953, *E. Matuda 28905* (MEXU); entre El Oro y Villa Victoria, 2000 m, 19–20 Jun 1954, *Matuda 30923* (MEXU); San Cayetano, Villa Allende, [19.3742, -100.0983], 2500 m, 3 Jul 1954, *Matuda 31073* (MEXU); Mpio. Villa de Allende, San Cayetano (Estación Experimental) al N de Agua Escondida y a 26 km al NE de Valle de Bravo, [19.3828, -100.1016], 2490 m, 7 Jul 1974, *Maury-H. s.n.* (MEXU); along old hwy 190 between turnoff to Chalco (hwy 115) and Santa Barbara, ca. 30 m above Azotla, [19.2500, -98.8667], 21 Jul 1964, *Mick 300* (IEB, WIS); Mpio. Huixquilucan, Fracionamiento La Herradura, [19.4193, -99.2457], 2800 m, 8 Jul 1968, *Perusquia-O s.n*. (F); near Toluca, [19.3015, -99.6745], 23 Jun 1887, *Pringle 2926* (GH); near Tultenango, [19.8444, -100.0859], 13 Jul 1901, *Rose 5413* (NY); no exact location, 22 Aug–19 Sep 1930, *Rusby 114* (CAS); Lava beds near Eslava, 19 Jul 1910, H.H. *Rusby 137* (NY); vertiente N del Cerro Sacromonte, cerca de Amecameca, [19.1299, -98.7751], 2500 m, 24 Jul 1966, *Rzedowski 22825* (ENCB, F); Mpio. Naucalpan, 6 km al E de San Francisco Chimalpa, [19.4420, -99.3043], 2550 m, 20 Aug 1966, *Rzedowski 22961* (CAS, ENCB, F, MEXU); Sierra de Alcaparrosa, 5 km al NW de Tepozotlán, [19.7279, -99.2582], 2600 m, 29 Jul 1971, *Rzedowski 28243* (ENCB, F, MEXU); Cerro del Tigre, al NW de Atizapán, [19.5651, -99.2799], 2500 m, 4 Aug 1974, *Rzedowski 32040* (ENCB, F); Mpio. Tepotzotlán, alrededores de la Hacienda Lanzarote, [19.7167, -99.2167], 2350 m, 31 Jul 1977, *Rzedowski 35068* (ENCB, F, MEXU); parte alta de la Sierra de Alcaparrosa, 10 km al NW de Tepotzotlán, [19.7573, -99.2891], 2800 m, 7 Aug 1979, *Rzedowski 36283* (ENCB, F); Ixtapaluca, km 42 antigua carretera México-Puebla, [19.3303, -98.7917], 2650 m, 6 Sep 1970, *Segura-R. 117* (CAS, F, IEB, MEXU); Mpio. Texcoco, San Pablo Ixayoc, [19.4707, -98.8004], 2600 m, 10 Sep 1983, *Ventura-V. 1375* (IEB, MEXU); 41 miles N of Toluca, 18 Aug 1957, *Waterfall 14048* (BRIT); Mpio. Ixtapaluca, Coatepec, cañada Xaltomatla, cerca del camino que pasa por la mina de arena Popotla, [19.4027, -98.8256], 13 Aug 1984, *Williams 578* (IEB, MEXU); **Michoacán**: Mpio. Zinapecuaro, aproximadamente 1 km al E del Balneario Las Adjuntas, carretera Querétaro-Jerahuaro, [19.8452, -100.7584], 2120 m, 24 Jul 1998, *Carranza 5567* (IEB, MEXU); Mpio. Chilochota, 8 km de Carapan, sobre la carretera a Cheran, [19.7849, -102.0289], 2140 m, 30 Jul 1998, *Carranza 5596* (IEB, MEXU); Mpio. Huandacareo, aproximadamente 1.5 km al E de Llano Grande, [19.9818, -101.2527], 2070 m, 28 Aug 1998, *Carranza 5620* (IEB); Mpio. Contepec, Cerro Altamirano, camino al llano, 19.9697, -100.1575, 2630 m, 29 Apr 2004, *Cornejo-Tenorio 597* (IEB); Mpio. Contepec, 2.5 km hacia el noroeste de Santa María la Ahogada, 20.0017, -100.1492, 3100 m, 15 Sep 2005, *Cornejo-Tenorio 1428* (IEB); Mpio. Lagunillas, lado sureste del Cerro El Aguila, subiendo por el poblado de Huatzanguio, 19.6064, -101.3792, 2530 m, 14 Aug 2008, *Cornejo-Tenorio 2840* (IEB); near Capácuaro, [19.5333, -102.0500], 2150 m, 7 Aug 1965, *Correll 31351* (LL); Mpio. Villa Escalante/Pátzcuaro, along Pátzcuaro-Villa Escalante rd, W side of rd, across from the microondas, west of Cerro El Tecolote, [19.7667, -101.8667], 2360 m, 22 Jul 1990, *Dean 216* (DAV, MEXU); Mpio. Villa Escalante, meadows and roadside SW Lagunita de San Gregorio, south of Cerro El Barro, [19.4045, -101.5724], 2360 m, 22 Jul 1990, *Dean 217* (DAV, MEXU, XAL); Mpio. Villa Escalante, along rd to San Gregorio, ca. 4.7 rd km E of intersection with Pátzcuaro-Villa Escalante rd, N side of road, [19.4115, -101.5619], 2470 m, 22 Jul 1990, *Dean 218* (DAV, MEXU); Mpio. Pátzcuaro, E of Pátzcuaro, along hwy 190, upslope (S and SSE) of Las Trojes, [19.5258, -101.0653], 2134 m, 23 Jul 1990, *Dean 219* (DAV, MEXU); Ejido Cruz de Caminos, along old hwy 15, W of Ciudad Hidalgo, [19.6667, -100.5000], 2195 m, 12 Nov 1991, *Dean 321* (DAV, MEXU, UC, XAL); Mpio. Villa Escalante/Pátzcuaro, Las Joyas de las Navas, along Pátzcuaro-Villa Escalante rd, about 3.2 rd mi N of turnoff to Tucambaro, [19.3306, -101.6505], 2348 m, 18 Nov 1991, *Dean 324* (DAV, XAL); Mpio. Villa Escalante, SW of Laguna de San Gregorio, along rd leading to radio tower, [19.4045, -101.5730], 2439 m, 18 Nov 1991, *Dean 325* DAV, NY, XAL); Mpio. Pátzcuaro, Las Trojes, E of Pátzcuaro along hwy 120, slopes SSE of town, [19.5258, -101.0653], 2134 m, 18 Nov 1991, *Dean 326* (DAV, MEXU, XAL); Mpio. Paztcuaro, camino a Las Trojes, sobre la carretera Patzcuaro-Morelia, [19.4877, -101.5895], 2100 m, 22 Jun 1985, *Diaz-Barriga 1179* (IEB); Mpio. Paztcuaro, 2 km al suroeste de los Tanques, [19.4826, -101.5972], 2300 m, 18 Aug 1988, *Diaz-Barriga 4719* (IEB); Mpio. Tingambato, Cerro La Chimilpa, cerca de Pichataro, [19.4667, -101.8000], 2400 m, 9 Jul 1986, *Escobedo 1012* (IEB, MEXU); Mpio. Paztcuaro, Cerro El Bao, cerca de Tzurumutaro, [19.5569, -101.5763], 2100 m, 12 Aug 1986, *Escobedo 1128* (IEB); Mpio. Zinapécuaro, km 116.5 de la carretera Toluca-Morelia, 19.9000, -100.8167, 2300 m, 21 Jun 1988, *González-M. 16986* (MEXU); Cerro de la Campana, unos 5–6 kms al N de Puruandiro, [20.1329, -101.5352], 19 Sep 1959, *Hernández-M. 32* (MEXU); Sierra Torrecillas, District Coalcoman, [18.8167, -103.0333], 2350 m, 25 Jul 1939, *Hinton 13994* (F, GH, LL, MO, NY, TEX, [specimen at W is *L. acapulcensis*]); Mpio. Morelia, aproximadamente 2 km al noroeste de La Concepcion, [19.7133, -101.3302], 2300 m, 5 Jul 1986, *Huerta 555* (IEB, MEXU); along hwy 15, 3 km ESE of Comanja, 16 km ESE of Zacapu, 19.7167, -101.6667, 2300 m, 26 Jul 1960, *Iltis 442* (WIS); at km 19, S. of Carapan on road to Uruapan 19.7500, -102.0500, 2300 m, 27 Jul 1960, *Iltis 483* (WIS); Mpio. Nahuatzen, Cerro Las Flores, 6 km al S de Sevina, [19.6052, -101.9008], 2600 m, 14 Jun 1990, *García 2666* (IEB); Cerro Huashan, aproximadamente 2 km al S de Nahuatzen, [19.6396, -101.9122], 2400 m, 28 Jun 1990, *García 2719* (IEB, MEXU); Mpio. Cheran, Ladera oeste del Cerro La Virgen, [19.6754, -101.9453], 2800 m, 7 Aug 1991, *García 3702* (IEB); Mpio. Zinapecuaro, 2 km al E de Jerahuaro, hacia Huajumbaro, [19.8778, -100.6267], 2380 m, 29 Sep 1988, *Jasso 259* (IEB); Mpio. Zinapecuaro, 1 km al N de Jerahuaro, [19.8907, -100.6464], 2440 m, 1 Jul 1989, *Jasso 1189* (IEB, MEXU); Mpio. Zinapecuaro, lado N de la presa La Gachupina, [19.8360, -100.6527], 2870 m, 4 Jul 1989, *Jasso 1213* (IEB); Mpio. Zinapecuaro, Cruz de Caminos, 2 km al oeste de Jerahuaro, [19.8850, -100.6632], 2440 m, 29 Jul 1989, *Jasso 1295* (IEB); Mpio. Zinapecuaro, 500 m al oeste de Santa Cruz, carretera Morelia-Mexico, [19.8318, -100.8049], 2000 m, 2 Aug 1989, *Jasso 1311* (IEB); Mpio. Zinapecuaro, La Lagunita, 2.5 km al S de Cruz de Caminos, [19.8629, -100.6653], 2430 m, 4 Aug 1989, *Jasso 1341* (IEB); Mpio. Zinapecuaro, El Cerrito, 1.5 km al E de Jerahuaro, [19.8764, -100.6273], 2540 m, 19 Aug 1989, *Jasso 1430* (IEB); Mpio. Uruapan, Tancítaro region, west of Uruapan on trail to Tancítaro, 19.4000, -102.2667, 6000 ft, 17 Jul 1941, *Leavenworth 1008* (F); Mpio. Quiroga, Mesa de los pastores, camino al cerro del Tzirate, [19.7066, -101.5023], 17 Jun 1986, *López-Campos 969* (IEB); Mpio. Tingambato. San Francisco Pichataro, [19.5667, -101.8000], 2300 m, 10 Nov 1978, *Mapes 23C* (IEB); Sicuicho, 1.5 km N del Pueblo, [19.6727, -102.3358], 2360 m, 27 Jun 1985, *Martínez-Estrada 11* (IEB); Mpio. Morelia, desviacion de San Miguel del Monte, rumbo a Atecuaro aproximadamente 2 km, [19.6155, -101.1569], 2300 m, 15 Jul 1989, *Medina 1787* (IEB); along the highway from Carapan to Paracho, km 33, [19.8027, -102.0583], 7000 ft, 3 Aug 1965, *Mertz 171* (MEXU); 23 km E of Morelia, km 290 from México City, just E of Ciudad Hidalgo, along Mex. hwy 15, 19.6667, -100.5167, 2600 m, 28 Jun 1964, *Mick 162* (WIS); salida poniente de San Isidro, [19.7000, -101.2667], 2650 m, 4 Jun 1981, *Motte 289* (MEXU); Santa Clara del Cobre, [19.4000, -101.6333], 2200 m, 16 Jul 1988, *Pérez-C. 109* (IEB, MO, TEX); faldas del Cerro San Miguel, frente a las antenas, carretera Patzcuaro-Santa Clara del Cobre, [19.4416, -101.6093], 2300 m, 6 Aug 1988, *Pérez-C. 139* (IEB); Mpio. Paracho, 1 km al noroeste de Cheran-Atzicurin, [19.7136, -102.0143] 2200 m, 30 Jul 1987, *Pérez-R. 121* (IEB, MEXU); Mpio. Santa Clara del Cobre, Lagunita de San Gregorio, [19.4000, -101.5333], 2750 m, 12 Jul 1989, *Pérez-C. 521* (IEB); Mpio. Santa Clara del Cobre, Joyas de las Navas, [19.4192, -101.7828], 2300 m, 28 Jul 1989, *Pérez-C. 552* (IEB, MEXU); Mpio. Tlalpujahua, al NE de Tlalpujahua, [19.8049, -100.1698], 2450–2550 m, 23 Jul 1998, *Pérez-C. 3831* (IEB); Mpio. Santa Clara del Cobre, laguna cerca de San Gregorio, [19.4333, -101.6833], 2700 m, 7 Jul 1985, *Rzedowski 38722* (IEB, MEXU, TEX); Santiaguito, al suroeste de Maravatio, [19.7667, -100.45], 2400 m, 28 Jun 1986, *Santos-Martínez 1412* (IBUG, IEB); 5 miles W of Cd. Hidalgo, [19.6833, -100.6000], 7 Jul 1947, *Sauer 1109* (UC); SSW of Morelia on the road to Villa Madero (just NE of Tiripetio), km 21, [19.5333, -101.3333], 2000 m, 19 Jul 1963, *Ugent 5786* (WIS); SSW of Morelia on the road to Villa Madero (just NE of Tiripetio), km 21, [19.5333, -101.3333], 2000 m, 19 Jul 1963, *Ugent 5786* (WIS); Mt. Punguato, Morelia, [19.6953, -101.1374], 2120 m, 4 Aug 1965, *Ugent 6108* (WIS); near Cheran, [19.6667, -101.9333], 21 Aug 1976, *Wonderly 320* (IEB); Mpio. Coeneo, 2 km al S de La Constitucion, [19.7193, -101.6523], 1970 m, 2 Aug 1988, *X- Ramos 158* (IEB); Mpio. Charo, Irapeo, [19.6913, -101.0558], 23 Oct 1991, *Zamudio-Ruiz s.n.* (IEB); Mpio. Tingambato, Llano de Cananguio, [19.5308, -101.8051], 2600 m, 2 Aug 1996, *Zamudio-Ruiz 9831* (IEB); Mpio. Tingambato, Llano Canangio, aproximadamente 3 km al N de Pichataro. [note by databaser, the coordinates put this location at 3 km south of Pichataro, not north], 19.5289, -101.8294, 2470 m, 21 Jul 1997, *Zamudio-Ruiz 10392* (DAV, IEB); Mpio. Zinapecuaro, Las Adjuntas, 19.8419, -100.7608, 2050 m, 20 Aug 2005, *Zamudio-Ruiz 13183* (IEB); Mpio. Morelia, 4 km al S de Jesús del Monte, [19.6160, -101.1482], 2100 m, 20 Jul 1986, *Rzedowski 39979* (IEB); Mpio. Morelia, 3 km al S de San Miguel del Monte, [19.5999, -101.1212], 2300 m, 24 Jul 1988, *Rzedowski 47034* (MEXU); **Nayarit**: Sierra Madre, territory of Tepic, between Sta. Gertrudis and Sta. Teresa, [21.7167, -104.7333], 8 Aug 1897, *Rose 2068* (GH); **Puebla**: Mpio. Chignahuapan, N of town of Chignahuapan, E of small town of San Joaquín, both E and W sides of Barranca El Salto, [19.8692, -97.9777], 2225 m, 22 Sep 1991, *Dean 264* (DAV, NY, XAL); Mpio. Chignahuapan, N of Chignahuapan, near town of San Joaquín, both sides of Barranca El Salto, [19.8692, -97.9777], 2225 m, 27 Oct 1991, *Dean 300* (DAV, MEXU, NY, UC, XAL); 9 miles W of San Martin Texmelucan, 19 Aug 1971, *Dwyer 891* (MO); Barranca de Ocoxicuaya, Chignahuapan, [19.8512, -97.9829], 2300 m, 14 Jul 1975, *Hernández-M. 2117* (CAS, MEXU, US,); Mpio. Xochiapulco, Rosa Chica, en un sitio llamado El Plan, cerca de la escuela priMaría, 19.7950, -97.6568, 2041 m, 25 Jun 2015, *Jimenez-Chimil 30760* (DAV); Amazoc, [19.0333, -98.0333], Aug 1946, *Martínez 15026* (MO); Mpio. Zautla, Rosa de Castilla, 19.7884, -97.6547, 2163 m, 8 Jul 2014, *Tenorio-L. 30027* (DAV); **Querétaro**: camino entre Amealco y Chiteje de la Cruz, lado del camino a unos 4 kms de caminata, [20.1724, -100.1666], 2850 m, 9 Jul 1978, *Arguelles 1127* (MEXU); La Muralla, [20.2047, -100.0040], 2460 m, 6 Jul 1986, *Arguelles 2556* (IEB); Mpio. Amealco, W of Amealco, along rd to Chiteje de la Cruz which turns off hwy 120, ca. 0.3 rd mi from junction with hwy 120, E side of road, 20.1860, -100.1569, 2530 m, 5 Aug 1991, *Dean 245* (DAV, XAL); Mpio. Amealco, W of Amealco, along rd to Chiteje de la Cruz which turns off hwy 120, ca. 0.3 rd mi from junction with hwy 120, E side of road, [20.1860, -100.1569], 2530 m, 18 Sep 1991, *Dean 256* (DAV, XAL); Mpio. Amealco, W of Amealco, along rd to Chiteje de la Cruz which turns off hwy 120, ca. 0.3 rd mi from junction with hwy 120, E side of road, 20.1860, -100.1569, 2530 m, 30 Oct 1991, *Dean 306* (DAV, XAL); Mpio. Amealco, Laguna de Servin, 20.2719, -100.2725, 2760 m, 5 Jul 2002, *Hernandez 4953* (IEB); Mpio. Paracho, El Picacho, desviacion San Pedro Tenango, 3 km al sureste de Amealco, [20.1374, -100.1152], 2650 m, 20 Jul 2003, *Serrano-Serrano 121* (IEB); 34 miles SE of San Juan del Río, 18 Aug 1957, *Waterfall 14022* (BRIT); **San Luis Potosí (it is doubtful that the following specimens were collected in San Luis Potosí; they are probably from a trip that Palmer took between San Luis Potosí and Mexico City)**: near San Luis Potosí, [22.1500, -100.9833], 6000–8000 ft, Apr 1879, *Parry and Palmer 662* (GH, K, MO; K collection mixed with Lycianthes moziniana var. margaretiana); chiefly in the region of San Luis Potosí, 6000–8000 ft, 1878, *Parry and Palmer 662* (NY). **Tlaxcala**: Tlaxcala, 6000–8000 ft, 19 Jun 1938, *Ball B4838* (K); Mpio. Españita, along rd to Españita, ca. 0.95 rd mi from intersection with hwy 136, W side of rd near large turnout, 19.4931, -98.4599, 2720 m, 22 Sep 1991, *Dean 262* (DAV, NY, XAL); Mpio. Hueyotlipan, E side of town of Hueyotlipan, down dirt rd and across a stream, [19.4667, -98.3333], 2591 m, 22 Sep 1991, *Dean 263* (DAV, XAL); Mpio. Tlaxco, N side of town of Acopinalco del Peñon, along old rd to Chignahuapan, 19.6196, -98.0999, 2652 m, 27 Oct 1991, *Dean 301* (UC, XAL); Mpio. Españita, 0.95 rd mi from intersection with hwy 136, along road to Españita, [19.4931, -98.4599], 2720 m, 28 Oct 1991, *Dean 302* (DAV); 7 mi E of Hwy 119, turnoff 1.0 mi S of Puebla/Tlaxcala state line, 19.5 mi N of Tlaxco, 19.67, -98.10, 10000 ft, 5 Aug 1977, *Reeves R5937* (ASU); 22 km al NE de Texcoco, sobre la carretera a Calpulalpan, [19.560966, -98.679898], 2900 m, 17 Aug 1971, *Rzedowski 28509* (ENCB, F); Mpio. Ixtacuixtla, San Juan Nepopoalco, [19.3167, -98.3667], 2500 m, 10 Sep 1982, *Williams 1* (CAS); Mpio. Calpulalpan, 4 km al S de Calpulalpan, sobre el camino rural a Nanacamilpa, [19.5536, -98.5706], 2650 m, 12 Aug 1983, *Williams 87* (F, TEX); Mpio. Nanacamilpa, 1km al S de Nancamilpa, sobre el camino rural a San Martín Texmelucan, 19.4840, -98.5518, 2700 m, 12 Aug 1983, *Williams 90* (CAS, MO); Mpio. Hueyotlipan, terrenos de la hacienda Sta. Cruz Tenancingo, 7 km al S de Hueyotlipan, [19.4235, -98.3490], 2600 m, 12 Aug 1983, *Williams 96* (WIS); Mpio. Españita, 8 km al noroeste de Hueyotlipan sobre la carretera 136, [19.5027, -98.3943], 2600 m, 6 Sep 1983, *Williams 145* (MEXU); Mpio. Panotla, San Tadeo Huiloapan, 5 km por vereda al N de San Tadeo, por la Barranca del Río Temeyaya (cerca de Barranca Honda), [19.4112, -98.2833], 2480 m, 7 Sep 1983, *Williams 151* (CAS, MO); Mpio. Amaxac de Guerrero, San Damian Tlacocalpan, 2 km al oeste de la carretera Apizaco-Tlaxcala, [19.3755, -98.2166], 2500 m, 8 Sep 1983, *Williams 162* (IEB); Mpio. Tlaxco, Acopinalco del Peñon, 1 km al N del pueblo, camino a Chignahuapan, [19.6255, -98.1253], 2660 m, 21 Sep 1983, *Williams 201* (CAS, IEB, MEXU, MO, NY); Mpio. Ixtacuixtla, Cruz Tenancingo, 12 km al S de Hueyotlipan, camino a San Felipe Ixtacuixtla, [19.7000, -101.2667], 2570 m, 24 Sep 1983, *Williams 289* (NY); Mpio. Panotla, San Tadeo Huilapan, 5 km al N de Huiloapan por vereda, Barranca del Río Temeyeya, [19.4112, -98.2833], 2480 m, 4 Oct 1983, *Williams 449* (CAS, F, IEB, MEXU, MO, TEX, WIS). **Veracruz**: Région d’Orizaba et Ingenio, 6 Oct 1886, *Bourgeau 3227* (G); Mpio. Acultzingo, high mountains near border of Veracruz and Puebla, El Sumidero, S of town of Puerto del Aire which is along hwy 150 between Orizaba and Tehuacán, [18.6888,-97.3314], 2439, 27 Sep 1991, *Dean 270* (DAV, NY, XAL); Maltrata, [18.8159, -97.2780], May 1937, *Matuda 30160* (MEXU); Orizaba, [18.8612, -97.1250], Aug 1853, *Müller 1117* (BR, NY, W); Orizaba, 1855, *Müller s.n.* (NY); Orizaba, [18.8612, -97.1250], 1853, *Müller 1617* (L); Mt. Orizaba, [18.8612, -97.1250], 1891, *Seaton s.n.* (GH); Mt. Orizaba, Esperanza, 18.8667, -97.3667, 8000 ft, 15 Aug 1891, *Seaton 373* (GH, NY); Mpio. Maltrata, camino a Zacatonal, [18.7869, -97.2767], 9 Jul 2013, *Vargas-Rueda 637* (MEXU).

**30b. Lycianthes moziniana var. margaretiana**

**Mexico. Nuevo León**: Summit of Mt. Infernillo, 9.5 miles south of Pablillo and 9.2 miles south of Mex. hwy 60, 9000 ft, 17 Jun 1963, *Bell 17856* (UC); Mpio. Galeana, E of the town of Pablillo, San Francisco Canyon, [24.5666, -99.9666], 4 Sep 1993, *Dean 360* (DAV, XAL, ANSM); Mpio. Zaragoza, Cerro El Viejo, [23.9277, -99.9636], 2485 m, 29 Jul 1992, *Hinton 22222* (GBH, TEX); Mpio. Galeana, above Agua Blanca, [24.4816, -99.9063], 2305 m, 4 Jul1991, *Hinton 22291* (TEX); Mpio. Aramberri, W of La Escondida, [24.0942, -99.9758], 2200 m, 3 Aug 1993, *Hinton 23168* (GBH, TEX); San Francisco Canyon, about 15 mi SW of Pueblo Galeana, [24.6790, -100.1254], 7500–8000 ft, 16 May 1934, *Mueller 404* (GH); lower San Francisco Canyon, about 15 mi SW of Galeana, [24.6790, 24.6790], 7500–8000 ft, 12 Jun 1934, *Mueller 778* (GH); Sierra Madre Oriental, descent to Banco de Santa Ana, about 15 mi SW of Galeana, in woods below Puerta de Santa Ana, [24.5500, -99.9466], 17 Jun 1934, *Mueller 855* (F, GH, TEX); **Querétaro**: Mpio. San Joaquin, aproximadamente 3 km al oeste de La Veracruz, carretera a San Joaquín, 20.9008, -99.5311, 2350 m, 6 Jul 2002, *Carranza 6365* (DAV, IBUG, IEB); Mpio. San Joaquin, entre San Joaquín y Corral Blanco, 20.9522, -99.5503, 2200 m, 20 Jul 1990, *Zamudio-Ruiz 7952* (DAV, IEB); **San Luis Potosí**: Hwy 86, 25 mi from Juárez Circle, beyond Xoconostle, [22.2, -100.9666], 9000 ft, 5 Jul 1971, *Andreasen 544* (MO); 96.2 mi SE of Huizache along hwy 80. 25 mi NW of El Salto, [22.4721, -99.7123], 3720 ft 25 Jul 1967, *Clarke s.n.* (ASU); 10–20 mi E of Ciudad del Maiz, [22.4036, -99.5216], 3000–4000 ft, 23 Jul 1953, *Manning 53479* (GH); same location and date, *Manning 53458* (GH, TEX); Alvarez, [22.0296, -100.6063], 5–10 Sep 1902, *Palmer 138* (NY); chiefly in the region of San Luis Potosí, 6000–8000 ft, 1878, *Parry 662* (K, mixed collection with Lycianthes moziniana var. moziniana); Santa María del Río, [21.8, -100.7333], 23 Jun 1959, *Rzedowski 3251* (ENCB, WIS); km 40 carretera San Luis Potosí-Rioverde, [22.0572, -100.6099], 2500 m, 20 Aug 1955, *Rzedowski 6228* (WIS); Sierra de Alvarez, cerca de Puerto Huerta, [22.0920, -100.6488], 2300 m, 10 Jul 1956, *Rzedowski 7730* (WIS); Cerro de San Pedro, cerros al SE de Jesús María, [22.15, -100.7666], 2300 m, 28 Jul 1956, *Rzedowski 7876* (WIS); Mpio. Tierra Nueva, entre Joyita y Paso de Ordena, [21.65, -100.5666], 1700 m, 9 Jun 1966, *Rzedowski 10727* (ENCB); Mpio. Zaragoza, Sierra de Alvarez, cerca de Puerto de la Huerta, [22.0920, -100.6488], 2300 m, *Rzedowski 11271* (ENCB).

**30c. Lycianthes moziniana var. oaxacana**

**Mexico. Oaxaca**: Llano de las flores, on the Oaxaca-Valle Nacional Highway, 20 km E of Ixtlán, 2870 m, 22 Jul 1960, *Beaman 3703* (GH, LL); Dto. Juxtlahuaca, Mpio. Santos Reyes Tepejillo, 24 km de Santiago Juxtlahuaca, carretera a Santos Reyes Tepejillo, 17.2400, -97.5700, 2100 m, 15 Jul 1996, *Calzada 21023*; Dto. de Ixtlán, Mpio. Ixtlán, Las Animas, carretera Oaxaca a Ixtlán de Juárez, [17.30, -96.4667], 2340 m, 29 Jul 1981, *Cedillo-T. 872* (F, MEXU); Dto.: Etla, Mpio.San Felipe Tejalapa, El Capulín, 17.0375, -96.9331, 2578 m, 8 Sep 2011, *Cervantes M. 220* (DAV); no exact location, Jun 1901, *Conzatti 1220* (GH); 22.95 km from route 190 jct. on rd to Díaz Ordaz, [16.9667, -96.4762], 31 Jul 1977, *Davis 801* (MEXU); field site same as 285 (holotype), grown from field collected seed in the greenhouse at UC Berkeley, harvested 31 Oct 1993, *Dean 285a* (DAV, MEXU, UC, XAL); Mpio. Sta. María Jaltianguis, Sta. María Jaltianguis, [17.3568, -96.5279], 2195 m, 20 Oct 1991, *Dean 296* (DAV, NY, UC, XAL); Dto. Sola de Vega, El Algodon cerca de Río Humo, 16.7177, -97.2470, 2232 m, 27 Jun 2006, *Jacob-Salinas 99* (MEXU); Dto. Sola de Vega, Mpio. Santiago Textitlán, Llano Borrego, 16.7456, -97.2481, 2509 m, 8 Aug 2006, *Jacob-Salinas 268* (MEXU); Dto. Tlaxiaco, Mpio. Santiago Yosondua, Imperio Santiago Yosondua, 16.9139, -97.6283, 2323 m, 12 Jul 2005, *Mendoza-Osorio 25* (MEXU); E-facing mountains along Route 175, 12 km N of Ixtlán de Juárez on the road to Valle Nacional, [17.4047, -96.509], 2500 m, 26 Jul 1959, *King 2011* (MEXU, TEX, WIS); Mpio. Putla Villa de Guerrero, 13 km al N de Santo Domingo Chicahuaxtla sobre la carretera a Tlaxiaco, 17.1799, -97.8260, 2400 m, 7 Jul 1994, *Panero-E. 4093* (BR, IEB, NY, TEX); Cerro Verde,[17.6501, -97.2450], Jul 1908, *Purpus 3611* (UC); Mpio. Tlalixtac, a 15 km al S de El Punto, [17.0882, -96.6395], 4 Jul 1981, *Ramamoorthy 2422* (MEXU); Mpio. San Juan Mixtepec, Tres Cruces, a 16 km al NE de San Juan Mixtepec, [17.4350, -97.7582], 2400 m, 19 Jul 1989, *Reyes-Santiago 1665* (MEXU); no exact location, *Salle s.n.* (BM); Dto. Etla, S. Juan del Estado, [17.2881, -96.8088], 18 Jun 1888, *Seler 812* (GH); Mts. San Juan del Estado, [17.2881, -96.8088], 25 Jun 1895, *Smith 401* (GH); N of Oaxaca about 40 miles, along Hwy 175 to Tuxtepec about 10–30 miles N of Ixtlán de Juárez, 2575 m, 24 Jul 1983, *Taylor 2432* (DUKE, ENCB); Mpio. Coixtlahuaca, Cerro Verde al NE de Marcos Pérez, 17°55'N, 97°22'W, 2700 m, 7 Jul 1986, *Tenorio-L. 11655* (MEXU).

**31. *Lycianthes nitida***

**Belize. Cayo**: Cohune Ridge, Humming Bird Highway, [16.8152, -89.0832], 20 Apr 1955, *Gentle 8681* (MO); 30 miles section Humming Bird Highway, [17.0846, -88.6021], 12 Jul 1956, *Gentle 9166* (MO); high ridge, hill slope, 33 miles section of Humming Bird Highway, [17.0923, -88.6422], 17 Jul 1956, *Gentle 9176* (MO); **Stann Creek**: Middlesex, [17.0475, -88.5148], 29 Jul 1939, *Gentle 2942* (NY); Stann Creek Valley, Big Eddy Ridge, 26 Apr 1940, *Gentle 3312* (MO, NY); 19 Mile Stann Creek Valley, [16.9498, -88.4170], 250 ft, 6 Jul 1932, *Schipp S303* (G, MO, NY); Humming Bird Highway, [17.0374, -88.5230], 5 Jun 1954, *Gentle 8203* (MO); **Toledo**: Bladen Nature Reserve, on Doyle’s Delight Ridge, 16.4923, -89.0460, 10 Aug 2004, *Brewer 1650* (DAV); vicinity of San José Mayan Indian Village, 6.7 mi N of Columbia Forest Station, [16.2749, -89.1035], 13 Jun 1973, *Croat 24456* (MO, NY); southern Maya Mountains, Bladen Nature Reserve, riverside vegetation between “AC Camp” and “AC Camp” helicopter landing site, upper Bladen Branch, 16.4919, 88.9102, 13 May 1997, *Davidse 36356* (DAV, MO); southern Maya Mountains, Bladen Nature Reserve, Cave Creek Canyon, dry tributary of upper Bladen Branch, between “AC Camp” and Muckle Bal Tsul archaeological ruin, 16.4941, -88.9241, 300 m, 13 May 1997, *Davidse 36388* (MO); southern Maya Mountains, Bladen Nature Reserve, upper Bladen Branch basin, along main Bladen Canyon, 16.4833, -88.9166, 250–300 m, 16 ay 1997, *Davidse 36486* (MO); southern Maya Mountains, Bladen Nature Reserve, trail from Central Snake Creek Camp to Tusbil Pek Cave archaeological site, 16.4602, -88.9869, 450 m, 21 May 1997, *Davidse 36668* (DAV, MO); southern Maya Mountains, Bladen Nature Reserve, ridge just south of the main divide of the Mayan Mountains, Augusta Divide Camp, 16.4894, -88.9925, 920 m, 22 May 1997, *Davidse 36692* (MEXU); southern Maya Mountains, Bladen Nature Reserve, trail from central Snake Creek Camp to Roochire Selipan archeological site, 16.4633, -88.9783, 450 m, 29 May 1997, *Davidse 36915* (MO); between Rancho Chico and Cockscomb, Monkey River, [16.3763, -88.5052], 7 Apr 1943, *Gentle 4394* (MO); in high ridge, base of hill, near Manga Camp, Edwards road, beyond Columbia, [16.3003, -89.0999], 30 Mar 1948, *Gentle 6494* (MO); beyond Central Camp (near Río Blanco), Edwards Road beyond Columbia, [16.2304, -89.0878], 24 Mar 1951, *Gentle 7247* (MO); beyond Carmelita camp, Edwards Road beyond Columbia, [16.2500, -89.0010], 25 Jun 1951, *Gentle 7382* (MO); southern Maya Mountains, Bladen Nature Reserve, West Snake Creek, along Snake Creek, 16.4649, -89.0177, 580 m, 29 May 1997, *Holland 39* (DAV, MO); southwestern Maya Mountains, Columbia River Forest Reserve, trail between Unión and Gloria Camps, 16.3894, -89.1361, 700–750 m, 13 Apr 1992, *Holst 4386* (MO); along divide of Maya Mountains, Bladen Nature Reserve, 16.5166, -88.9500, 11 May 1996, *Holst 5215* (DAV, MO); Columbia Forest Reserve, vicinity of forest camp, ca 6 miles due S of Cabro, in upper Río Grande drainage area, [16.3429, -88.9876], 1000 ft, 5–9 May 1976, *Proctor 36114* (MO, NY).

**Costa Rica. Alajuela**: 2.5 km NE of Arenal Volcano, 10.4666, -84.6833, 450 m, 5 Aug 1972, *Lent 2769* (MO, NY); lower slopes of Volcán Arenal, NE side, 10.4833, -84.6833, 500 m, 17 Sep 1972, *Lent 2940* (MO); two km N of Bijagua (on the road from Canas to Upala) on a branch road/trail to San Miguel, [10.7420, -85.0448], 470 m, 7 Nov 1975, *Utley 3237* (DUKE, MO); 9.8 km N of Río Naranjo or ca 50 km N of Cañas, on road to Upala, [10.1535, -84.3845], 440 m, 8 Nov 1975, *Utley 3253* (NY); **Limón**: vicinity of USDA rubber experiment station Los Diamantes on Río St. Clara (1.6 km E of Guapiles), [10.2042, -83.7662], 0 m, 12 Jul 1949, *Holm 408* (G).

**El Salvador. Ahuachapán**: entrada a la Presa, 13.81667, -89.9333, 780 m, 6 Mar 1998, *Sandoval 1805* (MO).

**Guatemala. Alta Verapaz**: Sebol, [15.8217¸ -89.9414], Jul 1964, *Contreras 5371* (MO); Semococh, 16 km from Sebol on Cobán Road, [15.5839, -89.5837], 15 May 1964, *Contreras 4696* (MO); Chahal, 2 km from airfield, [15.7903, -89.8149], 6 Oct 1968, *Contreras 7846* (MO); 1.5 to 2 mi S of Cubilquitz [Cubilhuitz], [15.6556, -90.4299], 300–350 m, 1 Mar 1942, *Steyermark 44485* (G); Cubilquitz [Cubilhuitz], [15.6675, -90.4293], Apr 1913, *von Tuerkheim 59* (WIS); same location, 350 m, May 1900, *von Tuerkheim 7637* (M, NY); **Huehuetenango**: between Ixcán and Río Ixcán, Sierra de los Cuchumatanes, bordering Río Lacandón, [15.9523, -91.1164], 150–200 m, 23 Jul 1942, *Steyermark 49352* (NY); **Izabal**: El Estor, [15.5388, -89.3651], 20 Mar 1972, *Contreras 11428* (MO); Mpio. Pto. Barrios, en la torre de Guatel, Sierra del Mico, [15.715414, -88.592611], 940 m, 8 Sep 1988, *Martínez-S. 23554* (MO); **Petén**: Mpio. Dolores, on km 82, about 300 m W of Machaquilá Road, [16.4887¸ -89.4317], 23 May 1961, *Contreras 2374* (MO); La Cumbre, Cadenas Rd, W of km 146, [16.0588, -89.3443], 14 May 1967, *Contreras 6907* (MO); [La Cumbre], km 142/143 of El Petén/ Izabal Road, [16.0790, -89.3452], 6 Mar 1975, *Lundell 19056* (MO, DUKE).

**Honduras. Atlántida**: base of N slope of Pico Bonito, in front of new CURLA (Centro Universitario Regional del Litoral Atlántico) camp building on Quebrada Grande, ca 1/3 km above its confluence with the Río Bonito, ca 10 km SW of La Ceiba, Parque Nacional Pico Bonito, 15.70, -86.85, 140 m, 8 May 1993, *Evans 1556* (MO, NY); NW slopes of the Cerro Los Mangungos, El Eden stream watershed, 15.5527, -87.3791, 120–195 m, 23 Apr 1995, *Hawkins 801* (MO); Pico Bonito National Park, Pico Bonito, trail between CURLA camp building and first river camp, along the E side of the river, 15.70, -86.8472, 400 m, 26 Apr 1996, *Hawkins 938* (MO); near the Danto river, slopes of Mt. Cangrejal, vicinity of La Ceiba, [15.7715, -86.8207], 800 ft, 30 Jul 1938, *Yuncker 8684* (G, MO, NY); **Gracias a Dios**: along creek 1 km below Camp Tiro, 2 mi NW of Bulevar on third northern branch of Quebrada Tiro, tributary of Río Platano, 15.7166, -84.8333, 200 ft, 23 Mar 1981, *Saunders 1099* (MO, NY); **Yoro**: Cordillera Nombre de Dios, hills S of San José de Texíguat, 15.4833, -87.4333, 300–400 m, 17 May 1991, Davidse 34497 (MO).

**Mexico. Chiapas**: Mpio. Berriozábal, 13 km N of Berriozábal near Pozo Turipache and Finca El Suspiro, [16.8744, -93.3252], 900 m, 9 Oct 1971, *Breedlove 20272* (MO); Mpio. Cintalapa, at crest of ridge 3 km E of Francisco Madero, NE of Cintalapa, [16.8143, -93.7346], 1250 m, 25 Aug 1974, *Breedlove 36670* (MO); Mpio. Ocosingo, 70 km SW of Palenque on road to Ocosingo along the Jol Uk’um, [17.1739, -92.1175], 550 m, 12 Apr 1981, *Breedlove 50886* (MO); Mpio. La Trinitaria, 15 km E NE of Dos Lagos above Santa Elena, [16.1341, -91.5576], 1000 m, 29 Dec 1981, *Breedlove 56663* (MO, NY); Mpio. Ocosingo, Nvo. centro de población Velasco Suárez (Selva Lacandona), 16.7994, -91.1170, 05 Oct 1976, *Ismael-Calzada 2696* (XAL73445); Mpio. Ocozocoautla de Espinosa, Cerro La Colmena, al NE del Rancho Corocito, Reserva del Ocote, [17.1111, -93.5627], 1350 m, 27 Apr 1983, *Calzada 9636* (XAL); Mpio. Ocosingo, a 2 km al N de Naja camino a Chancalá, [16.9425, -91.5858], 900 m, 17 Jun 1986, *Martínez-S. 18777* (MO, NY); Mpio. Ocosingo, en Estación Chajul [16.1178, -90.9242], 150 m, 16 Mar 1993, *Martínez-Salas 26347* (XAL); Mpio. Trinitaria, Col. Cuauhtémoc, km 24 de la carretera Monte Bello-Santa Elena, [16.1092, -91.6000], 15 Jun 1985, *Méndez-G. 8300* (NY); Mpio. Ocosingo, al N de la Estación Chajul, 16.0833, -90.4167, 180 m, 23 Jun 2000, *Sinaca-C. 2548* (XAL); Mpio. Ocozocoautla, 6 km del Pueblo de Ocozocoautla, al Cerro Horizonte, [16.8935, -93.5285], 1200 m, 9 Jun 1983, *Vásquez-B. 910* (XAL); Mpio. Ocozocoautla de Espinosa, 7 km de Horizonte a Ciprés, al Cerro El Banadero [16.8254, -93.3873], 1200 m, 10 Jun 1983, *Vázquez 932* (XAL); Mpio. Ocozocoautla de Espinosa, 7 km de Horizonte a Cipres, al Cerro El Banadero [16.8924, -93.5410], 1200 m, 10 Jun 1983, *Vázquez-B. 940* (MEXU); **Oaxaca**: Mpio. Santa María Chimalapa, San Antonio Nuevo Paraíso, a 3 km al W, Plan de la Ceiba, 17.1625, -94.3711, 250 m, 21 Sep 1997, *Torres 1353* (XAL); **Veracruz**: Mpio. Catemaco, Cerro Pipiapan, Predio Pipiapan, 18.4500, -95.0333, 700 m, 15 May 1986, *Acosta-P. 1173* (XAL); Mpio. Soteapan, Ejido Santa Marta, Barranca la Ventana, 18.3500, -95.9000, 1050 m, 19 Sep 1986, *Acosta 1308* (XAL); entre Bastonal y Arroyo claro, 14 km al E de Lago Catemaco, 18.3833, -94.8833, 900 m, 9 Jun 1972, *Beaman 6101* (MO); Mpio. San Andrés Tuxtla, Poblado de Laguna Escondida, a 5 km de la Est. de Biol. Trop. De Los Tuxtlas, Mapa 64-B-2, [18.5942, -95.0884], 300 m, 26 Sep 1974, *Calzada 1543* (NY); Estación de Biología Tropical, México, 18.5833, -95.0167, Jun 1971, *Calzada 321* (NY); Mpio. Mecayapan, Volcán de San Martin Pajapan, al S del Ejido La Valentina, [18.3359, -94.7228], 5 Aug 1985, *Calzada 11210* (XAL); Estación Biológica Tropical Los Tuxtlas (Laguna Verde), [18.5871, -95.0735], 400 m, 13 Apr 1972, *Cedillo-T, 173* (MO); La Laguna Escondida, 3.5 km NW Estación de Biología Tropical Los Tuxtlas, 18.5667, -95.0667, 200 m, 16 Apr 1985, *Colin 53* (MO, WIS); Mpio. Hidalgotitlán, 0–3 km del tramo Cedillo-La Laguna, [17.2553, -94.5819], 140 m, 5 Jul 1974, *Dorantes 3404* (XAL); Bastonal-Sierra Santa Marta Road, ca. 14 km E of Lago Catemaco, [18.396472¸ -94.92668], 700–800 m, 29 May 1981, *Gentry 32426* (MEXU, MO); Mpio. San Andrés Tuxtla, Laguna Escondida, 18.5833, -95.5833, 170 m, 12 May 1970, *Gómez-Pompa 4818* (XAL); Mpio. Catemaco, camino Bastonal a Santa Marta, 18.4000, -94.9500, 750 m, 26 Nov 1978, *Gómez-Pompa 5396* (XAL); Mpio. San Andrés Tuxtla, L. Zacatal. Estación de Biología Tropical los Tuxtlas, [18.5667, -95.0667], 150 m, 31 Aug 1983, *Ibarra-M. 844* (MO); Mpio. San Andrés Tuxtla, Estación de Biología Tropical Los Tuxtlas, Laguna Escondida. 3.5 km NW, 18.5667, -95.0667, 200 m, 16 Apr 1985, *Ibarra-M. 1986* (XAL); Mpio. Las Choapas, Rancho “El Milagro,” 5 km en línea recta al sureste de la colonia Nueva Tabasqueña 17.5300, -94.0289, 115 m, 5 Aug 2002, *López 195* (XAL); Mpio. San Andrés Tuxtla, Estación Biológica Los Tuxtlas, [18.5848, -95.0745], 230 m, 22 May 1970, *Martínez-Calderón 3006* (XAL); Mpio. Soteapan, along dirt road 13 km E of Tebanca (13 km E of E side of Lago Catemaco) on way to Santa Marta, [18.2596, -95.0321], 800–950 m, 5 Jul 1980 *Nee 18808* (WIS); Mpio. Pajapan, 5 km NW of Pajapan, SE slopes of Cerro San Martín Pajapan, 18.2917, -94.7167, 700 m, 3 Nov 1981, *Nee 22761* (MO); Mpio. Las Choapas, along Río Grande, upstream from the main gravel road of the Uxpanapa region, 17.2667, -94.3667, 100 m, 4 Mar 1984, *Nee 29868* (MO, NY); Mpio. Catemaco, entre Coyame y Bastonal, [18.434857, -95.012974], 3 Aug 1982, *Ramamoorthy 3897* (NY); Mpio. Mecayapan, Santa Marta, alrededores del Ejido Santa Marta, 18.3500, -94.8833, 1200 m, 11 Apr 1980, *Ramírez-R. 831* (XAL); Mpio. San Andrés Tuxtla, Laguna Zacatál, Estación de Biología Los Tuxtlas, 18.5667, -95.0667, 350 m, 15 Dec 1984, *Salazar 366* (BIGU); Mpio. San Andrés Tuxtla, Laguna Escondida, 3.5 km NW Estación de Biología Tropical Los Tuxtlas, [18.5915, -95.0879], 200 m, 16 Apr 1985, *Sinaca-C. 53* (WIS); Mpio. San Andrés Tuxtla, Estación de Biología Tropical los Tuxtlas, Cerro Lázaro Cárdenas, 18.5874, -95.0732, 450 m, 16 Jun 1986, *Sinaca-Colin 807* (XAL); Mpio. San Andrés Tuxtla, Estación de Biología Tropical los Tuxtlas, 73, 18.5874, -95.0732, 650 m, 16 Oct 1991, *Sinaca-Colin 1693* (XAL); Mpio. Catemaco, Cerro Egega, 8 km al N de Catemaco, camino a la colonia Cuauhtémoc, 18.4666, -95.0774, 500–700 m, 31 Jul 1999, *Torres-R. 235* (XAL); Mpio. Catemaco, Cerro Buenavista, 3 km al N de Catemaco, carretera Sontecomapan, 18.4833, -95.1000, 500–700 m, 27 Aug 1999, *Torres-R. 292* (XAL); Mpio. Río Solosúchil, entre Hnos. Cedillo y la Escuadra Hidalgotitlán, 17.2667, -94.6000, 150 m, 23 Aug 1974, *Vázquez 978* (MO); Mpio. Jesús Carranza, 2 km N del Poblado 2, Ejido F. J. Mina, 17.2667, -94.6667, 120 m, 28 Jul 1983, *Vázquez-T. 2626* (MO); Estación tropical Los Tuxtlas at Laguna Escondida, 18.5833, -95.0833, 300–400 m, 20 Aug 1976, *Webster 20915* (DAV); Mpio. Minatitlán, lomas al S del Poblado 11, ca 30 km al E del Campamento La Laguna, 17.2333, -94.3000, 180 m, 20 Jul 1980, *Wendt 2641* (MO); Mpio. Minatitlán, lomas al S del Poblado 11, ± 27 km al E de La Laguna, arroyo (cascada) empinado pedregoso en cañón, zona caliza, 17.2333, -94.2917, 200 m, 3 Jun 1981, *Wendt 3393* (MO, NY).

**Nicaragua. Boaco**: Upper SW slope of Cerro Mombachito, S of road between Boaco and Camoapa, 12.40, -85.55, 900–1000 m, 3 Oct 1979, *Stevens 14588* (BRIT); **Jinotega**: Cerro Kilambé, 1 km al NE de la Comarca de Sta. Cruz, 13.5666, -85.6666, 800–1000 m, 24 Mar 1981, *Moreno 7393* (MO); **Matagalpa**: falda NW del Cerro Musún, trocha de Palán, [13.0399, -85.2604], 300–600 m, 14 May 1980, *Araquistain 2477* (MO); al NW del cerro Musún, sobre el filo de la montaña, en el area faldar, a partir trocha a Paylo, [13.0043, -85.2690], 500–800 m, 15 May 1980, *Araquistain 2574* (NY); NW del cerro Musún, al lado del Río Bilampí, 4 km SW de Wanawás, 13.0003, -85.2333, 200–500 m, 15 May 1980, *Araquistain 2625* (MO); same location and date, *Araquistain 2684* (MO); Cerro Musún west and NW side above the Salto Grande of Quebrada Negra, 800 m, and in the valley of Rio Bilampi, property of Augustin Lacayo-B., [12.7922¸ -85.1219], 500 m, 20–21 1977, *Neill 1733* (MO); **Zelaya**: costado suroeste de Cerro El Hormiguero, 13.7361, -84.9972, 900–1000 m, 18 Apr 1979, *Grijalva 461* (MO); Cañon Monte Cristo, La Grupera, [11.55, -83.80], 10 m, 4 Feb 1982, *Moreno 14754* (MO); Cañon Monte Cristo, “Las Faldas,” 11.60, -83.85, 40–60 m, 5 Feb 1982, *Moreno 14764* (MO); Cerro La Pimienta number 1 and 2, eastern range, summit of 2 peaks, northernmost and central, 13.75, -84.8333, 900–1160 m, 17 Apr 1979, *Pipoly 5270* (MO); Mpio. de Siuna, Reserva Bosawas, costado oeste del cerro Saslaya, 13.75, -85.0333, 800–1300 m, 16 Apr 1996, *Rueda 4329* (MO).

**Panama. Bocas Del Toro**: vicinity of Fortuna Dam, along continental divide trail W of highway, 8.75, -82.25, 1250 m, 5 Sep 1987, *McPherson 11637* (MO); vicinity of Chiriqui Lagoon, [8.9402, -82.1280], 28 Oct 1940, *von Wedel 1387* (GH); **Coclé**: El Cope on Pacific side, 1/2 hour walk from sawmill, [8.4384, -80.6821], 2400 ft, 16 Oct 1979, *Antonio 2168* (MO).

**32. *Lycianthes ocellata***

**Guatemala. Alta Verapaz**: Mpio. San Juan Chamelco, Montaña Caquipec, Aufstieg von Caquipec, [15.4083, -90.1994], 1700–1900 m, 4 Sep 1999, *Förther 10407* (MSB, W); Mpio. San Juan Chamelco, Secochoy, [15.3986, -90.1814] 2300 m, 30 Sep 1998, *Robles 186* (MSB); Cobán, 1350 m, Jun 1907, *Von Tuerkheim 1810* (G, GH, NY, W); **Baja Verapaz**: Along Cobán-Guatemala Highway 14 near Biotopo del Quetzal Reserve, 15.2107, -90.2169, 1800 m, 9 Aug 2017, *Dean 9504* (DAV226610); along Cobán-Guatemala Hwy 14 near Biotopo del Quetzal Reserve, 15.2107, -90.2169, 1800 m, 9 Aug 2017, *Dean 9505* (DAV); Biotopo del Quetzal, [15.2188, -90.2272], 1630 m, 22 Jul 1988, *Droege 23023* (MEXU); Niño Perdido, on San José Espinero road, 6 km, [15.1371, -90.1786], 23 May 1977, *Lundell 20957* (MEXU, MO, NY); **Quiché**: Cerro Putul, “Zona Reyna,” 5300 ft, 3 Dec 1934, *Skutch 1835* (A).

**Mexico. Chiapas**: La Trinitaria, E of Laguna Tzikaw [*Tziscao*], Monte Bello National Park, [16.0873, -91.6625], 1300 m, 13 May 1973, *Breedlove 35135* (MEXU, MO).

**33. *Lycianthes orogenes***

**Guatemala. Alta Verapaz**: San Cristóbal, Finca Pamac II, 15.3986, -90.5883, 2146 m, 16 Aug 2015, *Borrayo MGC12* (BIGU); same location and date, ***Car 35*** (BIGU); Sierra de Chama/Montaña Yalijux, Chelem-ha, ca. 15 km NE von Tucuru, 2150 m, 20 Mar 1989, *Förther 2031* (M); Mpio. San Juan Chamelco, Montaña Caquipec, von Chicacnab I, in Richtung Saquil, ca. 1 km E Manuels Hutte, [15.3978, -90.1964] 2100–2200 m, 6 Apr 1998, *Förther 10048* (W); Mpio. San Juan Chamelco, Montaña Caquipec, Chicacnab I, Umgebung der biologischen Station des Proyecto Ecologico Quetzal, 15.3814, -90.1858, 2100–2170 m, 4 Sep 1999, *Förther 10432* (MSB, W); Mpio. San Cristóbal, A. V. finca Pacmac II, 15.4148, -90.6242, 1757 m, 15 Aug 2015, *Véliz 25054* (BIGU); **Baja Verapaz**: Unión Barrios, on Soloma/ Cobán road, on top of hill about 3 km W, 8 Feb 1975, *Lundell 18963* (F, LL, MO); Unión Barrios, top of hill, west of km 153/154, 15 Aug 1975, *Lundell 19642* (F, LL, MO); same location, 16 Aug 1975, *Lundell 19655* (F, LL, MO).

**Mexico. Chiapas**: Mpio. La Independencia, third ridge along logging road from Las Margaritas to Campo Alegre, [16.4756, -91.8231], 2300 m, 18 Feb 1973, *Breedlove 33573* (MEXU); 3 km al O de la carretera San Cristóbal de las Casas-Tenejapa, sobre el camino a Matzala. [16.6652, -92.5487], [2500 m], 29 Sep 1983, *Cabrera-C. 5773* (MEXU).

**34. *Lycianthes peduncularis***

**Field Origin Unknown.** No locality data or collector data (W); same (NY); Leipzig Botanic Garden, no collector, 1840 (MO); Leipzig Botanic Garden, no collector, label handwriting matches that of neotype, but not signed by Kunze (NY); Leipzig Botanic Garden, 1844, *Kunze s.n.* (W).

**Mexico. Guanajuato**: Mpio. San José Iturbide, near Rancho El Guajolote, SW of San José Iturbide, one hwy exit S of exit to San José, on W side of Hwy 57, [20.9008, -100.4085], 1829 m, 16 Sep 1991, *Dean 254* (DAV, XAL); Mpio. San José Iturbide, near Rancho El Guajolote, SW of San José Iturbide, one hwy exit S of exit to San José, dirt rd that goes W to large drainage, farm of Margarita Vargaz Fuentes de Acosta, [20.9018, -100.4214], 1829 m, 31 Oct 1991, *Dean 308* (DAV, XAL); Mpio. San José Iturbide, alrededores de El Guajolote, [20.9016, -100.4154], 2250 m, 10 Jul 1988, *Rzedowski 46920* (IEB); Mpio. San José Iturbide, cerca de El Guajolote, [20.899, -100.4091], 2200 m, 22 Aug 1988, *Rzedowski 47099* (IEB, MEXU); **Hidalgo**: Mpio. Ixmiquilpan, cañada de Arrollo Hondo, 25.9 km al NE de Ixmiquilpan, carretera a Tolatongo, 20.6319, -99.0268, 1870 m, 17 Jun 2000, *Cruz-Duran 4674* (MEXU); Mpio. Atotonilco El Grande, N of Atononilco El Grande and E of hwy 105, in canyon just upstream of the baths of Santa María de Amajac, [20.3099, -99.6970], 2050 m, 30 Jun 1990, *Dean 200* (MEXU); Mpio. Huichapán, 0.6 road km E of town of Comodoje, which is 18 rd km E of first exit to Huichapán traveling E on Hwy 45, [20.4171, -99.5575], 2200 m, 2 Jul 1990, *Dean 205* (DAV, MEXU); cerca de Atotonilco, en los Baños de Amajac, [20.3069, -99.7042], 2050 m, 3 Jun 1976, *Delgado-S. 232* (CAS, MEXU, US); autopista Querétaro, [20.6817, -100.4142], km 79, 6 Jun 1964, *Gold 135* (MEXU); Mpio. Huichapán, Comodoje, 13 km al E de Huichapán, [20.4195, -99.5619], 2200 m, 25 Jun 1980, *Hernández-M. 4568* (CAS, MEXU, MO); Mpio. Zimapán, camino a Minas de San Miguel, 10 km al N de Zimapán, [20.8148, -99.3944], 2100 m, 6 Oct 1980, *Hernández-M. 5135* (MEXU, US); Mpio. Zimapán, 10 km al N de Zimapán, hacia a la mina San Miguel, [20.7771, -99.3867], 2200 m, 28 Jun 1981, *Hernández-M. 6263* (MEXU, MO); Mpio. Huichapan, Comodeje, a 20 km al E de Huichapan, 20.4244, -99.5688, 2000 m, 12 Sep 1981, *Hernández-M. 6516* (MEXU, MO); 3.5 air km SW of Zimapan on road to Estanzuela, 20.7227, -99.405, 1740 m, 14 Jul 1991, *Mayfield 844* (TEX) ; Mpio. Ixmiquilpan, 5000–6000 ft, July-Aug, *Purpus s.n.* (UC); Tula, [20.05, -99.33], Jul 1905, *Rose 8342* (NY); Mpio. Ixmiquilpan, barranca de Tolantongo, E-facing slope, along main trail to bottom. 20°37'N, 99°4'W, 7 Jul 1974, *Sohmer 9329* (MEXU). **México**: Mpio. Huehuetoca, N of Huehuetoca along the road to Apaxco, ca. 4.2 road mi from building “Los Arcos” in downtown Huehuetoca, W side of road, [19.8896, -99.2083], 2200 m, 1 Jul 1990, *Dean 201* (DAV, MEXU, MO, NY, UC, WIS, XAL); Mpio. Huehuetoca, N of town of Huehuetoca along the rd to Apaxco, ca. 4.9 rd mi from building “Los Arcos”(in downtown Huehuetoca), E side of rd, near where RR tracks come close to rd, [19.8896, -99.2083], 2134 m, 3 Aug 1991, *Dean 244* (DAV, MEXU, UC, XAL); same location and elevation, 18 Sep 1991, *Dean 257* (DAV, MEXU, UC, XAL); same location and elevation, 29 Oct 1991, *Dean 303* (DAV, MEXU, UC, XAL); Valle de Mexico, Mpio. Huehuetoca, Huehuetoca, [19.8416, -99.1853], 2400 m, 24 Aug 1952, *Matuda 26639* (MEXU); Mpio. Apaxco, Cerro la Manga, ladera NE, [19.9992, -99.1280], 2350 m, 8 Jul 1981, *Romero-R. 1319* (MEXU); Mpio. Huehuetoca, ladera oeste del Cerro Mesa la Ahumada, [19.8833, -99.2], 2300 m, 15 Jun 1981, *Romero-R.1354* (ENCB); parte alta del Cerro de la Cruz, 7 km al N de Tepotzotlán, [19.7355, -99.2735], 2500 m, 12 Jul 1975, *Rzedowski 33255* (F, MEXU); 6 km al N de Huehuetoca, sobre la carretera a Apaxco, [19.8883, -99.2155], 2350 m, 11 Sep 1977, *Rzedowski 35236* (F, IEB); Cerro Ahumada, al N de Huehuetoca, [19.8664, -99.2228], 2400 m, 5 Jul 1981, *Rzedowski 37329* (IEB, MEXU); **Oaxaca**: Comotlán y cercanías, [17.4333, -96.1666], 1700 m, 29 Apr 1953, *Bravo s.n.* (MEXU, very depauperate form); 55 mi SE of Oaxaca along the road to Tehuantepec, in the mountains 9 mi NW of La Junta, [16.6622, -96.0740], 13 Sep 1971, *Clarke 20463-6* (TEX); Valle de Oaxaca, 1550 m, 8 Jun 1897, *Conzatti 285* (BH, GH, GOET, MEXU); no exact locality, 1750 m, July-Aug 1900, *Conzatti 1072* (GH); de Matatlán a Tlacolula, [16.9261, -96.395], 1600 m, 19–23 Jun 1906, *Conzatti 1470* (GH); Nochixtlán, [17.4333, -97.1666], 2000 m, 20 Jun 1907, *Conzatti 1844* (F, MEXU); Mitla, 27 Jun 1900, *C. Dean 10* (F, GH); Mpio. San P. Villa de Mitla, ca. 2.7 rd mi E of main road into the town of Mitla along the Mitla-Zacatepec rd, where rd meets foothills and crosses over river, W side of rd and N side of river, [16.9320, -96.3243], 1799 m, 18 Jul 1991, *Dean 230* (DAV, MEXU, XAL); Mpio. Nochixtlán, NW edge of town of Nochixtlán, along Hwy 190, NW of city of Oaxaca, [17.4668, -97.2443], 2073 m, 22 Jul 1991, *Dean 235* (DAV, MEXU, XAL); Mpio. Tamazulapan, E side of hwy 175, S of city of Oaxaca, ca. 15 rd km S of town of Miahuatlán in the Sierra Madre del Sur, [16.0705, -96.5728], 2012 m, 25 Jul 1991, *Dean 236* (DAV, XAL); Mpio. San Carlos Yautepec, along hwy 190 between city of Oaxaca and Tehuantepec, ca. 3 rd mi S of town of Las Minas, ca. 7 rd mi S of turnoff to San Juan la Jarcia, dirt rd, S side of hwy 190, [16.4525, -95.8729], 770 m, 29 Jul 1991, *Dean 239* (DAV, MEXU, NY, UC, XAL); Mpio. San Juan La Jarcia, slopes above Hwy 190 between city of Oaxaca and Tehuantepec, nearly opposite turnoff to San Juan la Jarcia, E side of the road, [16.5257, -95.9256], 915 m, 29 Jul 1991, *Dean 240* (DAV, XAL); Mpio. Teposcolula, slopes above town of Teposcolula, along rd to San Andrés Laguna, in the Mixteca region of Oaxaca, [17.5127, -97.5006], 2043 m, 31 Jul 1991, *Dean 242* (DAV, MEXU, XAL); Mpio. Nochixtlán, NW side of town of Nochixltán, near large drainage that crosses hwy 190, [17.4668, -97.24439], 2073 m, 9 Oct 1991, *Dean 283* (DAV, MEXU, UC, XAL); Mpio. San P. Villa de Mitla, ruins of Mitla, [16.9166, -96.3], 13 Oct 1991, *Dean 288* (DAV, XAL); Mpio. San P. Villa de Mitla, ca. 2.7 rd mi N of entrance to town of Mitla, along Mitla-Zacatepec rd, where road meets foothills, crosses drainage, and turns N, [16.9320, -96.3243], 1799 m, 13 Oct 1991, *Dean 289* (DAV, MEXU, UC, XAL); Mpio. San Carlos Yautepec, along hwy 190 between Oaxaca City and Tehuantepec, ca. 3 rd mi S of town of Las Minas, ca. 7 mi S of turnoff to San Juan la Jarcia, W side of rd, [16.4525, -95.8729], 770 m, 17 Oct 1991, *Dean 292* (DAV, MEXU, NY, UC, XAL); Mpio. San Juan la Jarcia, slopes above hwy 190 between city of Oaxaca and Tehuantepec, nearly opposite turnoff to San Juan la Jarcia, NE side of the rd, [16.5245, -95.9181], 915 m, 17 Oct 1991, *Dean 293* (DAV, XAL); Dist. Teposcolula, sobre el camino Teposcolula-San Andrés Lagunas, Mixteca Alta, [17.5115, -97.5142], 2160 m, 16 Apr 1981, *García-M. 258* (MEXU); in recently burned clearing, 7 mi by road SE of San Juan la Jarcia along Mex. hwy 190, [16.4891, -95.894], 1000 m, 31 May 1973, *Hansen 1586* (MEXU, WIS); Dto. Tlacolula, Mpio. Mitla, La Colorada, 16.92611, -96.395, 1767 m, 2 Jun 2009, *Hernandez O. 137* (DAV); 15 rd km SE of Miahuatlán on rd to Puerto Angel, in high mountains of Sierra Madre del Sur, [16.2424, -96.5260], 2400 m, 6 Jul 1969, *Marcks 1082* (WIS); Las Naranjas, in the vicinity of San Luis Tultitlanapa, Puebla, [17.083, -96.7], May 1908, *Purpus 3565* (F, NY, UC); Las Naranjas, [18.18, -97.45], May 1908, *Purpus 3566* (GH, MO, NY, UC); Santa Catarina, [16.2333, -97.2833], 14 Jul 1910, *Rusby 73* (NY); Dist. de Teposcolula, 5 km al NE de Chilapa de Díaz, [17.6316, -97.6189], 1750 m, 2 Jul 1977, *Rzedowski 34811* (CAS, F, IEB); Mitla, [16.9166, -96.3833], Jun 1888, *Seler 49* (GH); along Mitla-San Lorenzo rd on mountainside, [16.8944, -96.3078], 28 Jun 1968, *Smith 4787* (MEXU); Dto. Tlaxiaco, a 200 m del puente Yutama, sobre la brecha a Santa Cruz Itundujia, 16.9189, -96.6414, 2048 m, 18 Aug 2005, *Velasco-Gutiérrez 903* (MEXU); 3 mi N of Mitla, 5800 ft, 16 Jul 1969, *Weaver 2149* (MEXU, MO). **Puebla**: Mpio. Tehuacán, 4.4 km al E de San Pablo Tepetzingo, [18.4229, -97.2959],24 Jul 1979, *Chiang F60* (MEXU); Mpio. Zapotitlán de las Salinas, canyon of the onyx mines of San Antonio Texcala, S of Tehuacán along hwy 125 (Tehuacán-Huajuapan de León rd), [18.3833, -97.45], 1677 m, 13 Jul 1991, *Dean 226* (DAV, MEXU, UC, XAL); Mpio. Zapotitlán de las Salinas, San Antonio Texcala, canyon where there are onyx mines, N of town, S of Tehuacán along Hwy 125, [18.4003, -97.4464], 1677 m, 28 Sep 1991, *Dean 273* (DAV, UC, XAL); Mpio. Zapotitlán de las Salinas, San Antonio Texcala, along hwy 125, just S of Tehuacán, canyon with onyx mine N of town, [18.4003, -97.4464], 1677 m, 22 Oct 1991, *Dean 298* (DAV, MEXU, NY, UC, XAL); Mpio. Zapotitlán de las Salinas, minas de las canteras de San Antonio Texcala, [18.4004, -97.4456], 1700 m, 9 Jun 1978, *Ventura-A. 15264* (CAS, ENCB, F, MEXU); Mpio. Zapotitlán Salinas. San Antonio Texcala, 5 km al S de Texcala, sobre carr. 125 a Huajuapan de León, [18.3653, -97.4386], 13 Sep 1984, *Williams 584* (MO); **Querétaro**: Mpio. Ezequiel Montes, alrededores de Bernal, 20.7422, -99.9566, 2200 m, 5 Jun 1992, Rafael *Hernandez-M. 9905* (MEXU); Mpio. Cadereyta, 5 km al S de Vizarrón, sobre el camino a Cadereyta, 2300 m, [20.7872, -99.7239], 16 March 1989, *Rzedowski 48677* (IEB, XAL); Mpio. Cadereyta, 3 km al S de Vizarron, 20.8029, -99.7217, 2150 m, 1 Aug 1990, *Rzedowski 49676* (MEXU); Mpio. San Joaquin, Las Calabazas, en cercan¡as de Corral Blanco, [20.9502, -99.5378], 2050 m, 20 Jul 1990, *Zamudio-Ruiz 7977* (DAV, IEB, TEX); Mpio. Cadereyta, 4.5 km al S de Vizarron, [20.7904, -99.7232], 2150 m, 16 Aug 1996, *Zamudio-Ruiz 9866* (MEXU); Mpio. Cadereyta, Parador El Tepozan, [20.8937, -99.6524], 2300 m, 16 Jul 1997, *Zamudio-Ruiz 10321* (IEB).

**35. *Lycianthes pilifera***

**Mexico**. **Oaxaca**: Dto. Ixtlán, Mpio. Comaltepec, Cerro La Esperanza, [17.6306, -96.3687], 1749 m, 30 Aug 2011, *Aragon-Parada 96* (MEXU); Dto. de Ixtlán, Santa María Yavesia, [17.2354, -96.4291], 2910 m, 13 Apr 2002, *Benítez 10* (MEXU); Dto. Ixtlán, Sierra Norte, Mpio. San Miguel Yotao, Yotao, hacia Calpulalpan, 17.3658, -96.3583, 2150 m, 12 May 1999, *Blanco-M. 593* (MEXU); Mpio. Comaltepec, just off Highway 175 (road from Ixtlán de Juárez to Valle Nacional), on Caribbean slope, [17.7388, -96.3300], 2250 m, 17 Oct 1990, *Boyle 550* (MEXU); 20 km NE of Teotitlán del Camino on road to Huautla de Jiménez, [17.5755, -96.5059], 2130 m, 7 Nov 1983, *Breedlove 59916* (CAS); NE of Teotitlán del Camino on road to Huautla de Jiménez, 2260 m, 7 Nov 1983, *Breedlove 59916A* (MO); NW slope of Cerro Humo Chico, 43 km N of Ixtlán de Juárez junction on road to Valle Nacional, [17.5755, -96.5059], 2870 m, 9 Nov 1983, *Breedlove 59965* (MEXU); NE slope of Cerro Humo, [17.5753, -96.5047], 9 Nov 1983, *Breedlove 59987* (CAS, MEXU, MO);35 km N of Ayutla along road from Mitla to Choapam, N slope of Cerro Zempoaltepetl, [17.1576, -96.0526], 2470 m, 26 Sep 1986, *Breedlove 64691* (MEXU, NY, TEX); same location 17 Apr 1988, *Breedlove 66854* (MO, NY); Dto. Ixtlán, Mpio. Capulálpam de Méndez, NE de Río Natividad, 17.3117, -96.3775, 26 Sep 2002, *Brito-S. 282* (MEXU); Dto. Ixtlán, Sierra de Juárez, ruta 175 Tuxtepec a Oaxaca, 24 km por el camino N de la desviación Yólox, [17.6053, -96.5555], 2000 m, 6 Apr 1981, *Cedillo-T. 654* (MEXU, MO); Dto. Villa Alta, a 7 km de la desviación a San Andres Yaa, al S de Villa Alta, camino a Oaxaca, [17.3050, -96.1586], 2030 m, 15 Mar 1982, *Cedillo-T. 1199* (IBUG); a 5 km al N del Cerro Humo Chico, carr. Ixtlán a Valle Nacional, [17.3050, -96.1586], 2606 m, 26 Sep 1982, *Cedillo-T. 1871* (MEXU); Dto. Ixtlán, camino de Tepananacualco, [17.3306, -96.4872], 28 Mar 1912, *Conzatti 190* (MEXU); along Hwy 175 between Valle Nacional and Oaxaca, 2.3 mi below summit of Cerro Pelón, 32.1 mi NNE of Ixtlán de Juárez, 17.6167, -96.3833, 2580 m, 22 Feb 1987, *Croat 65628* (MEXU, MO, NY); Mpio. San Pedro Yólox, Sierra de Juárez, along Hwy 175 to the NE of the turnoff to Comaltepec and NE of the cabins and restaurant of Mirador, along old undeveloped road, 17.6028, -96.4175, 2022 m, 10 Sep 2017, *Dean 9522* (DAV); along road between Mitla and Zacatepec, 34 km by road ENE of Ayutla, 4 km by road E of intersection with road to Totontepec, 17.1333, -96.0333, 2554 m, 22 Jun 1986, *G. Diggs 3887* (NY); 30 km by road ENE of Ayutla along road between Mitla and Zacatepec, just E of intersection with road to Totontepec, 17.1333, -96.0667, 2500 m, 22 Jun 1986, *Diggs 3928* (NY); Talea (near San Pedro Nolasco), [17.2740, -96.4208], Aug 1844, *Galeotti, H. 1225J* (BR); Mpio. San Felipe Usila, camino a Cerro Carrizo, 3.1 km en línea recta al S de Santa Cruz Tepetotutla, 17.7111, -96.5642, 2000 m, 22 Nov 1993, *Gallardo-H. 846* (IEB, MEXU, XAL); Mpio. San Felipe Usila, cuenca del Río Perfume (ladera oeste), 8.1 km en línea recta al S de Santa Cruz Tepetotutla, 17.6650, -96.5567, 2400 m, 31 Mar 1994, *Gallardo-H. 1012* (CAS, IEB, MEXU, XAL); Mpio. San Felipe Usila, cuenca del Río Perfume (ladera oeste), 6.9 km en línea recta al S de Santa Cruz Tepetotutla, 17.6772, -96.5456, 2000 m, 18 May 1994, *Gallardo-H. 1078* (IEB, MEXU, XAL); Mpio. San Felipe Usila, cuenca del Río Perfume (ladera oeste), 8 km en línea recta al S de Santa Cruz Tepetotutla, 17.6656, -96.5600, 2500 m, 14 Sep 1994, *Gallardo-H. 1186* (IEB, MEXU, XAL); same location and date *Gallardo-H. 1192* (IEB, MEXU, XAL); Dto. Ixtlán, 13 km al N de La Esperanza, [17.6275, -96.3624], 1900 m, 9 Apr 1987, *García-M. 3041* (IBUG, MEXU); Dto. Cuicatlán, Mpio. Concepción Pápalo, 15 km al NE de Concepción Pápalo, cañada húmeda, [17.9024, -96.8070], 2700 m, 16 Nov 1993, *García-S. 155* (MEXU); San Pedro Nolasco, Talea, etc, [17.2740, -96.4208],1843–44, *Jurgensen, C. 961* (G); Mexico: no location, no date, *Karwinski s.n.* (BR, M, W); Dto. de Ixtlán, Sierra de Juárez; Ruta 175 Tuxtepec a Oaxaca, a 15 km al NE de la desviación a Yólox, [17.5911, -96.4325], 2200 m, 16 Apr 1982, *Lorence 4035* (CAS, MEXU); Dto. de Villa Alta, camino de Natividad a Talea de Castro, a 5 km al S de la desviación a Yalina (comedor Maravillas), [17.5930, -96.5042], 2580 m, 3 Aug 1985, *Lorence 4713* (IBUG, MEXU); Dto. Ixtlán, Mpio. Comaltepec, Sierra de Juárez, camino de Ruta 175 a la cascada (brecha 60 Comaltepec) al N de Cerro Pelón, [17.5930, -96.5042], 2350 m, 5 Aug 1985, *Lorence 4738* (MEXU); desviación a San Juan Acatepec sobre la carretera Ixtlán, 17.4235, -96.5331, 2200 m, 15 Jan 1983, *Lorenzo 9* (MEXU); desviación a San Pedro Yólox, sobre la carretera Ixtlán-Valle Nacional, [17.5793, -96.5114], 2800 m, 15 Jan 1983, *Lorenzo 16* (MEXU); Carretera Oaxaca-Tuxtepec, km 110 more or less, [17.5882, -96.4375], 24 May 63, *MacDougall s.n.* (MEXU); Mpio. San Felipe Usila, cuenca del Río Perfume (ladera oeste), 9.1 km en línea recta al S de Santa Cruz Tepetotutla, 17.6578, -96.5469, 2430 m, 30 May 1995, *Meave 1692* (IEB, MEXU, MO); Mpio. San Felipe Usila, poza con cascada, a orilla de terracería, 12.5 km en línea recta al S de Santa Cruz Tepetotutla, 17.6364, -96.5175, 2720 m, 3 Jun 1995, *Meave 1720* (IEB, MEXU, XAL); Mpio. San Felipe Usila, cuenca del Río Perfume (ladera oeste), terrenos de Santa Cruz Tepetotutla, 17.6942, -96.5608, 1930 m, 23 Nov 1996, *Meave 2051* (XAL); Dto. Ixtlán, Mpio. San Pedro Yólox, 8 km E of Yólox on road between Yólox and Hwy 175, [17.5953, -96.5425], 13 Apr 1981, *Martin 532* (MEXU, MO, US); Sierra Mazateca. Aproximadamente 30 km de Teotitlan de Flores Magon, por la carretera a Huautla de Jimenez (MEX 182). Antes de llegar al Puerto de la Soledad, 18.1681, -97.0031, 2300 m, 3 Oct 2002, *Munn-Estrada 1483* (NY); Sierra Mazateca, aprox. 400 [m?] del Puerto de la Soledad, por la carretera de Huautla a Teotitlán de Flores Magón (MEX 182), 18.1681, -97.0017, 2320 m, 13 Feb 2002, *Munn-Estrada 1946* (NY); Mpio. Mazatlán Villa de Flores, Sierra Mazateca, San Pedro de los Encinos, 18.1681, -97.0017, 2325 m, 23 Apr 2002, *Munn-Estrada 2254* (NY); same location and date, *Munn-Estrada 2270A* (NY); Dto. Ixtlán, Mpio. Comaltepec, Sierra Norte, brecha área de las cascadas, [17.5814, -96.5233], 2606 m, 21 Jun 2001, *Pérez-P. 36* (IBUG, MEXU); Mpio. San Felipe Usila, cuenca del Río Perfume (ladera oeste), 7.3 km en línea recta al S de Santa Cruz Tepetotutla, 17.6731, -96.5578, 2220 m, 15 May 1994, *Rincón-G. 416* (IEB, MEXU, XAL); Mpio. San Felipe Usila, cuenca del Río Perfume (ladera oeste), 7.6 km en línea recta al S de Santa Cruz Tepetotutla, 17.6694, -96.5528, 2240 m, 16 May 1994, *Rincón-G. 446* (IEB, MEXU, MO, XAL); same location and date, *Rincón-G. 452* (IEB); same location but 2430 m, 9 Jul 1994, *A. Rincón G. 468* (IEB, MEXU); Mpio. San Felipe Usila, cuenca del Río Perfume (ladera O), 8 km en línea recta al S de Santa Cruz Tepetotutla, 17.6689, -96.5453, 2000 m, 2 Nov 1994, *Rincón-G. 514* (IEB, MEXU, MO); Mpio. San Felipe Usila, parteagua, S de la cuenca del Río Perfume, 10.5 km en línea recta al S de Santa Cruz Tepetotutla, 17.6483, -96.5275, 2560 m, 15 Sep 2004, *Rincón-G. 3068*, (IEB, MEXU, XAL); Totontepec Villa de Morelos, Distrito Mixe. Mpio. Totontepec Villa de Morelos. Kets tekum, tonun kux, [17.2597, -96.0291], 2 Apr 1991, *Rivera-Reyes 2609* (MEXU); Dto. Mixe, Totontepec Villa de Morelos, Kets tekum, tonun kux, [17.2047, -96.0254], 17 Jul 1994, *Rivera-R. JR-3141* (IBUG); same location, 2 Apr 1991, *Rivera-R. 2609* (MEXU); 39 km al S de Valle Nacional sobre la carretera a Oaxaca, [17.5946, -96.4545], 1800 m, 26 Dec 1975, *Rzedowski 33768* (MEXU, NY); 39 km al S de Valle Nacional sobre la carretera a Oaxaca, [17.5946, -96.4545], 10 Apr 1976, *Rzedowski 34028* (MEXU); San Juan Tepeuxila, Cuicatlan, cañada, [17.7310, -96.8672], 1990 m, 1 May 1993, *Silvia Salas M. 510* (MEXU); San Juan Tepeuxila, Cuicatlán, 1990 m, 1 May 1993, *Salas-M. 510* (MEXU); N of Oaxaca about 40 miles, along hwy 175 to Tuxtepec, about 10–30 miles N of Ixtlán de Juárez, [17.5112, -96.5089], 2575 m, 24 Jul 1983, *Taylor 2452* (DUKE); Mpio. Teotitlán de Flores Magón, Puerto de la Soledad, 30 km al NE de Teotitlán, 18.1667, -97.0000, 2360 m, 18 May 1986, *Tenorio-L. 11284* (MEXU); Dto. Teotitlán de Flores Magón, Mpio. Teotitlán de Flores Magón, Raya San Jerónimo, 4 km al SE de Plan de Guadalupe, brecha a Mazatlán de Flores, 18.1670, -97.0170, 2800 m, 15 Feb 1993, *Tenorio-L. 18547* (MEXU); Dto. Teotitlán de Flores Magón, Mpio. Teotitlán de Flores Magón, Puerto de la Soledad, [18.1322, -97.0707], 2105 m, 10 Apr 2001, *Tenorio-L. 20020* (MEXU); Dto. Ixtlán, Llano Verde, 16 km al sureste de Calpulalpan [Calpulálpam], [17.2491, -96.4076], 2230 m, 15 Apr 1982, *Torres-C. 305* (IBUG, MEXU); Dto. de Mixe, a 11 km al N de Mixtepec ó 3 km al N de la desviación a Metepec, sobre la brecha a Totontepec, [17.2554, -96.0532], 16 May 1982, *Torres-C. 455* (IBUG, MEXU, NY); Dto. de Mixe, 5.2 km al NE de la desviación a Zacatepec, [17.2146, -95.9762], 2380 m, 23 Apr 1983, *Torres-C. 2681* (IBUG, MEXU); Dto. de Villa Alta, 8.1 km al N de Maravillas, camino a Talea de Castro por Yalina, [17.3115, -96.3000], 2370 m, 15 May 1983, *Torres-C. 2939* (IBUG, MEXU); Dto. de Mixe, entrada a Mixistlán, 30.8 km al NE de Tamazulapan [Tamazulapam], [17.1434, -96.1130], 2430 m, 7 Apr 1984, *Torres-C. 4942* (IBUG, MEXU); Dto. Mixe, 2 km al suroeste de Totontepec, carretera a Oaxaca, 17.2647, -96.0452, 17 Jun 1986, *Torres-C. 8650* (MEXU); Dto. Ixtlán, Mpio. Comaltepec, 22 km al oeste de La Esperanza, carretera Tuxtepec-Oaxaca, [17.5879, -96.4982], 2900 m, 9 Apr 1987, *Torres-C. 9518* (IBUG, MEXU); same location and date, *Torres-C. 9521*, (IBUG, MEXU); Dto. Ixtlán, Mpio. Comaltepec, entrada a San Isidro Yólox, 10.9 km al suroeste de La Esperanza, carretera Tuxtepec-Oaxaca, 17.5965, -96.4149, 2070 m, 24 Feb 1988, *Torres-C. 11712* (MEXU); Dto. Mixe, Mpio. Totontepec Villa de Morelos, 1 km al N de la desviación a Villa Alta, la cual se encuentra 7.5 km al S de Totontepec, 17.2333, -96.0833, 2460 m, 25 Feb 1988, *Torres-C. 11789* (IBUG, MEXU); Dto. Ixtlán, Mpio. San Juan Quiotepec, Cerro Zacate recorrido de la Cruz a la capilla, Santa María Nieves, 17.6282, -96.5580, 25 May 2002, *Torres-C. 16414* (MEXU); Dto. Ixtlán, Mpio. Comaltepec, 11.1 km suroeste de La Esperanza, carretera Oaxaca-Tuxtepec, entranda al camino de San Isidro Yólox, 17.6000, -96.3833, 1950 m, 17 Dec 1987, *Campos-Villanueva 910* (IBUG); Dto. Teotitlán, mountain ridges between Teotitlán del Camino and Huautla de Juárez, 7500–7800 ft, 8 Jul 1972, *Webster 17270* (DAV, DUKE); Sierra Madre Oriental, ca. 3.5 mi NE of Cerro Pelón, 17.6333, -96.4167, 2250 m, 19 Jul 1972, *Webster 17455* (DAV, MEXU).

**36. *Lycianthes pringlei***

**Mexico. Guerrero**: Cañada del Espino, puerto de León, [18.3226, -100.7041], 8 Sep 1973, *González-Medrano 6198* (MEXU); Leonardo Bravo, a 1 km al S de Filo de Caballo, camino a Chichihualco, [17.6443, -99.8402], 2270 m, 12 Jun 1982, *Martínez-Salas 791* (MEXU); 4 km al S de Tetipac, sobre el camino Tetipac-Taxco, [18.6285, -99.6452], 1820 m, 5 Dec 1982, *Martínez-Salas 2863* (MEXU, NY); **Jalisco**: Ahualulco del Mercado, Carretera Ahualulco del Mercado-Ameca, aproximadamente 15 km al noroeste de Ahualulco del Mercado, aproximadamente 1 km sobre la brecha a Piedras Bola, 20.6467, -104.0353, 1870 m, 27 Sep 2016, *Anguiano-Constante 181* (XAL); Cerro Santa María, 8–10 km SW of Jiquilpan and ca. 5 km NE of Quitupan, [19.9269, -102.7525], 2000 m, 8–9 Aug 1959, *Charles 195* (TEX); Ahualulco de Mercado, Area Natural Protegida Piedras Bola, 20.6489, -104.0435, 1947 m, 29 Oct 2011, *García-Martínez 128* (MEXU); Mpio. Jocotepec, cauce conocido con el nombre de Jaral, exposición sur, a 2.5 km de Huejotitan, [20.3591, -103.4725], 1950 m, 2 Aug 1986, *Machuca-N. 3052* (NY); Mpio. Jocotepec, Paraje Peña Prieta, Cerro Viejo, enfrente del poblado de Zapotitan de Hidalgo, [20.3554, -103.4676], 1800 m, 14 Aug 1988, *Machuca-N. 6180* (CAS, IEB, XAL); Mpio. Tlajomulco de Zuñiga, ladera norte del Cerro Viejo, frente de San Miguel Cuyutlan, [20.3752, -103.3975], 2300 m, 23 Sep 1990, *Machuca-N. 6563* (IEB); Mpio. Jocotepec, Barranca de Sayula, al oeste de San Pedro Tesistan, [20.2423, -103.4395], 1750 m, 5 Jun *1992*, *Machuca-N. 6886* (IEB, MEXU, NY); Lake Chapala, [20.2954, -103.2080], 19 Oct 1895, *Pringle, C.G. 6154* (BH, BM, BR, C, G, GH, GOET, HBG, JE, K, L, M, MEXU, MO, NY, S, UC, W, ZT); Mpio. Tlajomulco de Zuñiga, ladera de exposición norte del Cerro Viejo, subiendo por la barranca de Las Cruces, al S de San Miguel Cuyutlan, [20.3881, -103.4268], 23 Sep 1990, *Ramírez-Delgadillo 2289* (IEB, MEXU); Mpio. Bolaños, primer manantial, brecha Bolanios-Puente de Camotlan, [21.8245, -104.1068], 1970 m, 25 Mar 1991, *Rodríguez-C.2352* (IEB, MEXU); Mpio. Tecalitlán, 2 a 5 km al NE de Plan de Lego, brecha a Las Animas, [19.4423, -103.3218], 1850 m, 10 Dec 1989, *Villa-C. 433* (NY); **México**: Temascaltepec, Nanchititla, [18.8346, -100.4071], 27 Nov 1935, *Hinton 8750* (CAS, GH, NY); **Michoacán**: Morelia, Rincón, 1900 m, 20 Jun 1909, *Arsène 2707* (NY); vicinity of Morelia, Rincón, [19.6399, -101.2045], 1950 m, 14 Apr 1910, *Arsène 6554* (GH, MO); Mpio. Erogaricuaro, 3 km antes de Arocutin, a un lado de la carretera Patzcuaro-Erongaricuaro, [19.5483, -101.6950], 24 Sep 1978, *Caballero 509* (IEB); Mpio. Erogaricuaro, derrame basaltico (malpais), 5 km al oeste de Erongaricuaro, [19.5882, -101.7286], 1900–2000 m, 22 Aug 1993, *Chazaro-B. 7200* (IEB, MEXU, NY, TEX); malpais despues caseta cobros de Cuitzdo, [19.9121, -101.1492], 2045 m, 13 Nov 1995, *Chazaro-B. 7581* (IEB, TEX); Mpio. Jimenez, La Alberca de Teremendo de los Reyes, 19.8064, -101.4556, 2072 m, 15 Oct 2013, *Contrera- L. 93* (MEXU); Mpio. Morelia, cañada del Río Grande, near Presa Cointzeo, SE of city of Morelia, [19.6273, -101.2430], 6400 ft, 15 Nov 1991, *Dean 323* (DAV); Mpio. Jimenez, La Laguna de la Alberca, N of Zacapu, Volcáno crater with lagoon, [19.9080, -101.7658], 6650 ft, 20 Nov 1991, *Dean 327* (DAV, MEXU); al E de Zacapu, cerca de Celanese, [19.8203, -101.7621], 2100 m, 22 Oct 1986, *Diaz-Barriga 3178* (IEB, MEXU, MO); Mpio. Coeneo, Transmaraan, [19.8378, -101.5752], 2100 m, 15 Oct 1991, *Escobedo 2223* (IEB114722, MEXU926156); Mpio. Erogaricuaro, Pedregal de Tocuaro, [19.5381, -101.6951], 2100 m, 7 Aug 1992, *Escobedo 2399* (IEB, MEXU); Mpio. Morelia, Cañada del Río Grande, [19.6706, -101.2526], 1850 m, 17 Aug 1991, *García 3800* (IEB, MEXU); Mpio. Erogaricuaro, Pedregal de Arocutin, 19.5522, -101.7142, 2100 m, 17 Jul 2004, *Molina 569* (IEB, MEXU, TEX); Mpio. Jimenez, Cerro La Alberca, [19.9054, -101.7703], 2000 m, 21 Dec 1990, *Pérez-Calix 2056* (IEB, MEXU); Mpio. Jimenez, Cerro La Alberca, [19.9054, -101.7703], 2000 m, 20 Nov 1991, *Pérez-Calix 2586* (IEB, MEXU); Mpio. Uruapan, 2 km al N de Caltzontzin, [19.4334, -102.0054], 1700 m, 2 Nov 1991, *Pérez-Calix 2600* (IEB114729, MEXU027142); Coru, [19.4675, -101.9450], 12 Oct 1904, *Pringle 13466* (C, M, S); Coru Station, [19.4736, -101.9473], 6000 ft, 26 Jan 1907, *Pringle 13903* (C, CAS, GH, L, LL, LL, MEXU, MO, NY, S, TEX, UC); Lava beds near Coru, [19.4800, -101.9478], 12 Oct 1904, *Pringle s.n.* (GH); los filtros viejos, cerca de Morelia, [19.6770, -101.1538], 1950 m, 19 Aug 1986, *Rzedowski 40403* (IEB, MEXU); alrededores de los filtros viejos cerca de Morelia, 1950 m, 12 Nov 1986, *Rzedowski 41770* (IEB); corriente de lava basaltica, 3 km al W de Zacapu, 2150 m, 22 Nov 1987, *Rzedowski 45867* (IEB); Mpio. Erogaricuaro, alrededores de Tocuaro, [19.5369, -101.6929], 2050 m, 17 Sept. 1989, *Rzedowski 48965* (IEB, XAL); Mpio. Morelia, alrededores de los filtros viejos, [19.6770, -101.1538], 2000 m, 12 Oct 1999, *Rzedowski 53740* (IEB, MEXU, XAL); Mpio. Huaniqueo, NE del pedregal pequenio, 5 km al oeste de Tendeparacua, [19.9003, -101.4406], 2080 m, 22 Aug 1992, *Silva-Saenz 109* (IEB, MEXU); Mpio. Huaniqueo, oeste del pedregal pequeño, 1.7 km al oeste-suroeste de Tendeparacua, [19.8925, -101.4398], 2150 m, 06 Oct 1992, *Silva-Saenz 317* (IEB, MEXU); Mpio. Huaniqueo, suroeste del pedregal pequeño, 2 km al suroeste de Tendeparacua, [19.8914, -101.4298], 2070 m, 16 Jan 1993, *Silva-Saenz 566* (IEB); Cerro Zinaparo, 3.5 km al sureste de Churintzio, 20.1167, -102.0333, 1980 m, 29 Aug 1992, *Trejo 2483* (MEXU); Mpio. Ziracuaretiro, malpais de San Andres Coru, 19.4589, -101.9447, 1654 m, 10 Aug 2012, *Valentin-Martínez 309* (MEXU).

**37. *Lycianthes purpusii***

**Belize. Toledo**: southwestern Maya Mountains, Columbia River Forest Reserve, trail between Unión and Gloria Camps, [16.3894, -89.1361], 700–750 m, 13 Apr 1992, *Holst 4389* (MO); Temash River, [15.9848, -89.0635], 150 ft, 8 Feb 1935, *Schipp 1328* (A, GH, MO, NY).

**Guatemala. Alta Verapaz**: Mpio. San Juan Chamelco, Montaña Caquipec, Aufstieg von Caquipec nach Chicacnab, 15.3842, -90.1831, 1900 m, 6 Sep 1999, *Forther 10496* (MSB, W); Mpio. San Juan Chamelco. Montaña Caquipec, Chicacnab, Sendero Secochoy Abajo, 2300 m, 20 May 1998, *Robles 86* (MSB); **Baja Verapaz**: Purulhá, Biótopo del Quetzal, 15.2131, -90.2200, 400 m, 18 Oct 1995, *Cahuec s.n*. (BIGU); Chilasco, San Jorge, on hill, 5 km, [15.1161, -90.1567], 8 Aug 1971, *Contreras 10981* (MO); Purulhá, Transecta 44 B, [15.2131, -90.2200], 2100 m, 24 Jul 1996, *García s. n*. (BIGU); Niño Perdido, E of km 150/151, 30 Aug 1975, *Lundell 19762* (MO); **Izabal**: on Río Dulce Road, 10 Jul 1970, *Contreras 10173* (MO); El Estor, 3 Mar 1972, *Contreras 11118* (MO); Mpio. Puerto Barrios, en el Río las Escobas, camino entre Pto. Barrios y Punta de Palma, 120 m, 10 Sep 1988, *Martínez-S. 23662* (MO); vicinity of Puerto Barrios, 0 m, 2–6 Jun 1922, *Standley 25086* (GH); Montañas del Mico, 11 km W of Santo Tomás de Castillas, microwave tower, [15.6719, -88.6929], 940 m, 8 Sep 1988, *Stevens 25496* (NY); **Petén**: La Esperanza, on Cadenas Road, W of km 148/149, 2 Nov 1966, *Contreras 6508* (MO); 19 km N of Modesto Méndez, 200 m, 21 Jun 1971, *Harmon 5855* (MO).

**Honduras. Atlántida**: valley near dam for water supply of Progreso, [15.4300, -87.8451], 12 Aug 1929, *Bangham 367* (A); Cordillera Nombre de Dios, Quebrada Grande on lower N slope of Pico Bonito, [15.6949, -86.8549], 25 May 1987, *Blackmore 4192* (MO); base of N slope of Pico Bonito, E of new CURLA (Centro Universitario Regional del Litoral Atlántico) camp building on Quebrada Grande, ca. 1/3 km above its confluence with the Río Bonito, ca. 10 km SW of La Ceiba, Parque Nacional Pico Bonito, 15.70, -86.85, 140 m, 8 May 1993, *Evans 1560* (NY); Campamento Quebrada Grande ca. 10 km SW of La Ceiba, at base of N slope of Pico Bonito, from camp to 2 km E of camp, 15.70, -86.85, 80–150 m, 9 May 1993, *Liesner 26094* (MO, NY); Lancetilla, 10 km SO de Tela, [15.7368, -87.54419], 30 m, 18 Aug 1979, *Soto 15* (MO); Lancetilla Valley, near Tela, [15.7368, -87.4575], 20–600 m, 6 Dec to 20 Mar 1928, *Standley 53285* (A); same location and date, *Standley 53670* (A); same location and date, *Standley 55231* (A); same location and date, *Standley 56550* (A); Lancetilla Valley, ca. 3 mi S of Tela, along trail to dam, [15.7412, -87.4561], 200–500 ft, 30 Jul 1962, *Webster, 12705* (DAV); hills near Lancetilla, [15.7282, -87.4497], 200 m, 26 Apr 1947, *Williams 13058* (GH); Lancetilla, Floresta de la Presa de Lancetilla, [15.7618, -87.5342], 80 m, 26 Jul 1948, *Williams 14428* (GH); above Lancetilla, [15.7409, -87.4559], 200 ft, 16 Jul 1934, *Yuncker 4603* (A, MO, NY); near the Danto river, slopes of Mt. Cangrejal, vicinity of La Ceiba, [15.6875, -86.6237], 1200 ft, 2 Jun to Aug 1938, *Yuncker 8752* (GH, MO, NY); **Yoro**: foothills of the Cordillera Nombre de Dios, S de San José de Texíguat and on the western side of the canyon of the Río Texíguat, 15.50, -87.4333, 250–350 m, 15 May 1991, *Davidse 34414* (NY); Cascada de Río Guán Guán, 15.4944, -87.4544, 420 m, 21 Apr 1995, *Hawkins 789* (NY); cerro between Río Guán Guán and Río Texíguat, E of Cerro Guán Guán, S of San José in the Río Leán Valley, western end of the Cordillera de Nombre de Dios, [15.4917, -87.45], 700–870 m, 6 Nov 1988, *MacDougal 3200A* (MO).

**Mexico. Chiapas**: Mpio. Ococingo, along road 199, ca. 5.6 km W of entrance to Palenque and Rd. 307, 17.4206, -91.9956, 431 m, 19 Sep 2016, *Acevedo-Rdgz. 16426* (MEXU); Mpio. Ocosingo, crucero de Bonampak a 0.8 km de la carretera rumbo al SE, 16.7992, -91.0964, 464 m, 25 Nov 2002, *Aguilar-M. 4374* (BIGU); Mpio. Ococingo, a 1.47km del crucero de San Javier, 16.8031, -91.1189, 368 m, 27 Oct 2003, *Aguilar-M. 8295* (IEB, NY); Mpio. Ocozocoautla de Espinosa, 18–20 km N of Ocozocoautla along road to Mal Paso, [16.8940, -93.4528], 800 m, 20 Oct 1971, *Breedlove 20984* (MO); Mpio. Palenque, along ridges 6–12 km S of Palenque on the road to Ocosingo, [17.4468, -91.9623], 300 m, 22 Feb 1972, *Breedlove 24214* (MO); Mpio. of Ocozocoautla de Espinosa, 18–20 km N of Ocozocoautla along road to Mal Paso, 800 m, 18 Aug 1972, *Breedlove 27127* (MO); Mpio. Ocozocoautla de Espinosa, 32 km NW of Ocozocoautla. [16.9866, -93.4900], 600 m, 27 Aug 1972, *Breedlove 27493* (MO); Mpio. Ocozocoautla de Espinosa, 18–20 km N of Ocozocoautla along road to Mal Paso, [16.8940, -93.4528], 800 m, 27 Sep 1972, *Breedlove 28104* (MO); Mpio Ocozocualtla de Espinosa, Cerro del Ocote, 20 km NW of Ocozocoautla, 1500 m, 14 Oct 1972, *Breedlove 28893* (MO); Mpio. Palenque, S of Tulija river 40 km SW of Palenque on road to Ocosingo, [17.3054, -92.0501], 300 m, 26 Oct 1980, *Breedlove 46931* (MO); Mpio. Ococingo, 70 km SW of Palenque on road to Ocosingo along the Jol Uk’um, [17.1739, -92.1175], 550 m, 4 Dec 1980, *Breedlove 48373* (MO); Mpio. La Libertad, 15–20 km towards Chancala on road to Bonampak from the Palenque-Ocosingo road, 280 m, 4 Jan 1981, *Breedlove 49119* (MO); Mpio. Palenque, near Cascada Mizola, 25 km S of Palenque on road to Ocosingo, 300 m, 11 Nov 1981, *Breedlove 55394* (MO); Palenque, near Agua Azul, 600 m, 14 Jan 1982, *Breedlove 57265* (MO); Mpio. La Libertad, 10 km towards Chancala on road to Bonampak from the Palenque-Ocosingo road, 600 m, 25 Jan 1982, *Breedlove 57827* (MO); sobre el camino a Sibal, a 8 km al S de Santo Domingo, [16.9927, -91.4307], 13 May 1982, *Cabrera 2703* (MO); Mpio. Palenque, vicinity of Palenque archeological site, [17.4875, -92.0390], 170 m, 11 May 1982, *Davidse 20331* (MO); Mpio. Ocosingo, 8 km NW of Bonampak, Lacanja-Changayab at the Río Lacanja, 350 m, 14–15 May 1982, *Davidse 20488* (MO); Mpio. Berriozábal, along road from Berriozábal to Las Maravillas, ca. 1.4 km S of the town of Efraín A Gutiérrez, in remnant of tall forest called La Mata Café, 16.8711, -93.2956, 1005 m, 12 Sep 2017, *Dean 9527* (DAV); Mpio. Tumbala, Agua Azul, 350 m, 16 Mar 1983, *Fernández-N. 1503* (NY); Mpio. Ococingo, 1 km antes del Río Chancalá, viniendo desde Palenque, [17.2884, -91.6428], 300 m, 27 Sep 1989, *González-Espinosa 860* (MEXU); on trail behind Naranjo, 8 km from the ruins of Palenque, 4 Mar 1975, *Hoover 156* (MO); Mpio. Ococingo, a 2 km al NE de Bonampak, sobre la sierra de Cojolite, [16.7216, -91.0729], 350 m, 28 Sep 1984, *Martínez-S. 7942* (NY); Mpio. Ococingo, en el Ojo de Agua de San Javier, a 23 km al SE de Nvo. camino a Boca Lacantum, [16.8014, -91.1047], 370 m, 29 Jan 1986, *Martínez-S. 16910* (NY); Mpio. Javalinero, Palenque, [17.5095, -91.9821], 6–9 Jul 1939, *Matuda 3649* (NY); NO de Berriozábal, [16.8018, -93.2885], 23 Aug 1953, *Miranda 7861* (MEXU); Mpio. Ococingo, en el camino de Lacanha Chanizayab a Bonampak, [16.7578, -91.1183], 300 m, 18 Jan 1984, *Narave-F. 1210* (XAL); Mpio. Palenque, on road to Bonampak, 16 k SE of turnoff to Chancala, 400 m, 8 Jan 1978, *Perino 3066* (MO); Finca Irlanda, Jun 1914, *Purpus 7314* (M); 17 km NW of Ocozocoautla on the road to Mal Paso, 775 m, 4 Aug 1965, *Roe 889* (WIS); 11 km S of Crucero El Piñal, 2 km SE of El Piedron, 17.1667, -91.7500, 600 m, 26 Sep 1988, *Stevens 25898* (MO); Mpio. Ocozocoautla de Espinosa, on the SW side of the Presa de Malpaso, 2200 ft, 5 Dec 1967, *Ton 3327* (DUKE); Mpio. Tumbala, Cascadas de Agua Azul, [17.2529, -92.1189], 400 m, 16 Mar 1983, *Ventura-A. 20014* (MO); Mpio. Palenque, 12.4 km al S de Palenque, sobre carretera a Ocosingo (Carr. 199), 3 km al S del entroque con carretera a Chancala (Carr. 192), 17.4500, -91.9833, 320 m, 1 Dec 1979, *Wendt 2303* (NY); **Oaxaca**: Dto. Juchitán, Uxpanapa Region, along gravel road between Esmeralda (17 km E of Sarabia) and Río Verde, 1.1 mi S of Esmeralda, [17.1600, -94.7500], 100 m, 19 Jan 1987, *Croat 63291* (DAV, MO); Mpio. Santa María Chimalapa, Cabecera del Arroyo Sardina, ca. 1 km al S de la vereda a Carrizal, ca. 8–9 km al E de Sta. María, 16.9167, -94.6333, 250 m, 9 Jul 1985, *Hernández-G. 1310* (NY); Mpio. San Felipe Usila, Nueva Santa Flora, 17.9275, -96.4608, 700 m, 20 Dec 1992, *Ibarra-Manriquez 3764* (XAL); same location, 22 Dec 1992, *Ibarra-Manriquez 3781* (XAL); Mpio. San Pedro Ixcatlan, en el poblado de Cerro Quemado, entrada por la cerretera Ojitlan-Xalapa de Diaz, [17.0475, -96.7976], 230 m, 8 Feb 1984, *Calzada 10280* (XAL); Mpio. Juchitán, Chichihua, camino a Sta. María Chimalapa, [16.8922, -94.6950], 28 Aug 1984, *Torres-C. 5989* (MO); **Puebla**: Mpio. de Hueytamalco, 1 km hacia el oeste de las instalaciones del Campo Experimental “Las Margaritas,” Instituto Nacional de Investigaciones Forestales, Agrícolas y Pecuarias (INIFAP), 20.0044, -97.3167, 550 m, 19 Nov 2007, *Gómez-Chagala 349* (MEXU); Bosque do Ajenjibre, May 1952, *Ramírez-Cantu 21* (MEXU); **Veracruz**: Mpio. Catemaco, Cerro Pipiapan, 18.3500, -95.0833, 600 m, 17 May 1986, *Acosta-P. 1214* (MO); Mpio. San Andres Tuxtla, Monte Pio, 50 m, 21 May 1972, *Avendaño-D. 1932* (WIS); around and near isolated mountain village of Santa Marta, on S slopes of Cerro San Martin, immediately around Santa Marta, 18.3500, -94.9167, 1300 m, 23 Jun 1993, *Ballard 93-53* (WIS); Mpio. Catemaco, lado SE de Laguna Catemaco, arriba del Río Cuetzalapan,18.3167, -95.0167, 19 Oct 1971, *Beaman 5130* (NY); Mpio. Hidalgotitlán, San Andres Tuxtla, 10 km al NE de Tapalapan, [18.5996, -95.2455], 450 m, 31 May 1972, *Beaman 6025* (BRIT); ca. 3 km al E de Lago Catemaco, camino a Bastonal, [18.3666, -94.9863], 550 m, 11 Aug 1972, *Beaman 6434* (NY); Estacíon Biológica Los Tuxtlas, 18.5833, -95.0167, 400 m, 24 Apr 1972, *Cedillo-T. 209* (XAL); Mpio. Catemaco, 4.4 mi (7km) W of Sontecomapan on road to Catemaco, 510 m, 25 Sep 1985, *Cowan 5789* (NY); Mpio. Hidalgotitlán, km 12 del camino Cedillo-La Escuadra, 17.7833, -94.6667, 140 m, 2 Nov 1974, *Dorantes 3702* (XAL); Mpio. Hidalgotitlán, camino viejo de Hnos. Cedillo-La Laguna, 17.2500, -94.5833, 140 m, 10 Apr 1974, *Dorantes 45239* (MO); trail to Ejido Laguna Escondida near Estacíon Biología Los Tuxtlas. 170–200 m, 28 May 1981, *Gentry 32371* (MO); Mpio. San Andres Tuxtla, Estacíon de Biología Tropical Los Tuxtlas, 18.5847, -95.0739, 80 m, 28 Dec 1968, *Gómez-Pompa 3915* (XAL); Mpio. Coatzacoalcos, Parque Ecologico Jaguaroundi. Sendero de Educacion, 18.1077, -94.3618, 19 m, 12 Aug 2010, *Gómez-Chagala 1022* (XAL); Mpio. Soteapan, San Fernande, 670 m, 15 May 1986, *González-R. 178* (XAL); Mpio. Coatzacoalcos, zona de Salvaguarda-Pemex-La Cangrejera, a 8 km al SSE de Coatzacoalcos, [18.0830, -94.3454], 28 Dec 1998, *Hanan-Alipi 1221* (XAL); Mpio. Coatzacoalcos, Zona de Salvaguarda-Pemex-La Cangrejera, a 8 km al SSE de Coatzacoalcos, [18.0830, -94.3454], 9 Jul 1999, *Hanan-Alipi 1357* (XAL); Mpio. San Andres Tuxtla, Estación de Biología Tropical “Los Tuxtlas,” 18.5000, -95.0833, 200 m, 9 Sep 1975, *Holstein 20361* (DAV); camino a Laguna Escondida, Estacíon de Biología Tropical de Los Tuxtlas, [18.5871, -95.0735], 170 m, 5 Jul 1977, *Horvitz 203* (NY); Mpio. San Andres Tuxtla, Estación de Biología Tropical Los Tuxtlas, 18.6000, -95.1500, 160 m, 3 Nov 1983, *Ibarra-M. 986* (MO); same location, 24 Jun 1984, *Ibarra-M. 1779* (MO, NY); Mpio. San Andres Tuxtla, Estación de Biología Tropical Los Tuxtlas, 18.6000, -95.1500, 250 m, 27 Aug 1986, *Ibarra-M. 3003* (MO); Mpio. San Andres Tuxtla, Volcán de San Martin Tuxtla lado sur, camino para el crater lado sur, [18.5484, -95.2109], 1150 m, 30 May 1984, *Calzada 10611* (XAL); Mpio. Choapas, Rancho “El Milagro,” 5 km en línea recta al sureste de la colonia Nueva Tabasquenia. 17.5300, -94.0289, 115 m, 5 Aug 2002, *López 192* (XAL); 7.5 km E of Tebanca (7.5 km E of E side of Lago Catemaco) on dirt road to Bastonal and Santa Marta, [18.3704, -94.9594], 880 m, 5 Jul 1980, *Nee 18846* (MO, WIS); Mpio. San Andres Tuxtla, Estación de Biología tropical de Los Tuxtlas, 15 Sep 1982, *Ramamoorthy 4107* (NY); Mpio. Catemaco, Cumbres de Bastonal, 18.3969, -94.9520, 500 m, 19 Nov 1974, *Cedillo-Trigos 423* (XAL); Mpio. Uxpanapa, El Luchador, por el camino a San Antonio Nuevo Paraíso, 17.1886, -94.3592, 400 m, 22 Jun 1999, *Rivera-H. 1230* (XAL); Mpio. Soteapan, Mirador Pilapa, 200 m, 15 May 1986, *Vásquez-T. 3450* (XAL); Mpio. Hidalgotitlán, brecha hermanos Cedillo-La Laguna, [17.2550, -94.5956], 200 m, 14 Feb 1974, *Vazquez 12* (XAL); Mpio. Soteapan, camino San Fernando-Santa Martha, 18.3333, -94.8833, 1150 m, 29 Jun 1982, *Vásquez-B. 538* (XAL); Mpio. Hidalgotitlán, brecha Hermanos Cedillo-A. Melgar, 17.2667, -94.6000, 150 m, 24 Aug 1974, *Vazquez 1007* (MO); Mpio. Hidalgotitlán, 5 km al noroeste del campamento hermanos Cedillo, por la brecha Cedillo-La Escuadra, 17.2667, -94.6000, 150 m, 14 Jan 1975, *Vazquez 1674* (XAL); Mpio. San Andres Tuxtla, Estación de Biología Tropical “Los Tuxtlas,” ca. 19 mi by road NW of Catemaco, 18.5833, -95.0667, 400 ft, 28 Aug 1976, *Webster 20962* (DAV); Mpio. Minatitlán. 6.6 km al N de la terracería La Laguna-Río Grande, sobre el camino nuevo (no completo) a Ejido Belisa Río Domínguez, el cual sale de la terracería 14.7 km al E de La Laguna, 17.3333, -94.3667, 130 m, 13 Jul 1980, *Wendt 2548* (MO); Mpio. Jesús Carranza, 1.5 km del Poblado 2. Ejido F.J. Mina, 17.2667, -94.6667, 120 m, 25 Jun 1984, *Zambrano-C. 1300* (MO); Mpio. Soteapan, camino San Fernando-Santa Martha, 18.3333, -94.8833, 1000 m, 29 Jun 1982, *Zavaleta-P. 20* (NY, XAL).

**38. *Lycianthes quichensis***

**Guatemala**. **Chimaltenango**: Volcán de Acatenango, 11 Sep 1993, *I. Arias 93-3291* (MEXU); Santa Helena, [14.7842, -91.0186], 5 Dec 1936, *Johnston 406* (NY); Volcán de Acatenango, 2800 m, 22 Apr 1999, *Véliz 99-7020* (BIGU, MEXU); slopes of Cerro Chichoy, above Tecpán, [14.7483, -90.9859], 2670 m, 6 Jul 1949, *Williams 16817* (MO); Cerro de Tecpám, region of Santa Elena, [14.7842, -91.0186], 2400–2700 m, 26 Dec 1938, *Standley 61108* (NY). **Huehuetenango**: Mpio. San Mateo Ixtatán, along road to San Pedro Soloma, 3 mi SW of San Mateo Ixtatán, [15.6971, -91.4386], 9600 ft, 6 Feb 1965, *Breedlove 8630* (DS); along road to Huehuetenango, 5 miles south of San Juan Ixcoy, [15.5342, -91.4890], 9200 ft, 5 Aug 1965, *Breedlove 11485* (DS); ridge NE of Cerro Boquerón on road from El Rosario to Niquivil, 2255 m, 29 Nov 1986, *Breedlove 66145* (CAS); 5 km N of San Juan Ixcoy, 8950 ft, 12 Nov 1970, *Harmon 4832A* (MO); Mpio. La Libertad, Peña Blanca, 15.5075, -91.9158, 3193 m, 14 Dec 2000, *Véliz 10846* (BIGU, CAS). **Quetzaltenango**: Mpio. Zunil, NW slopes of Volcán Zunil, 2900 m, 14 May 2004, *Quedensley 654* (BIGU, TEX); Volcán Zunil, 3000–3200 m, 27 Dec 1976, *Schwabe s.n.* (MEXU); Cumbre de Alaska, [14.7560, -91.4705], 3100 m, 10 Sep 1999, *Véliz 99-7285* (BIGU, MEXU). **Sacatepequez**: Volcán de Agua, [14.4799, -90.7338], 5 Mar 1994, *Bill 94-3621* (MEXU, MEXU); Volcán de Agua, road between Santa María de Jesús and crater, 14.4569, -90.7403, 2800–3300 m, 7 Jul 1986, *Diggs 4049* (NY); Volcán Agua, 3000 m, 13 Nov 1967, *Molina-R. 21023* (NY); Santa María de Jesús,Volcán de Agua, 14.4769, -90.7322, 2852 m, 6 Feb 2006, *Véliz 16671* (BIGU, TEX). **San Marcos**: Mpio. San José Ojetenam, San José Ojetenam, [15.2342, -91.9736], 3100 m, 26 Nov 2009, *Pérez 18* (BIGU); Cerro El Bonete, south of Volcán Tajumulco, 11 Mar 1971, *Plowman 3052* (TEX); two miles S of San Sebastian, Sierra Madre Mountains, [15.0405, -91.8376], 10000 ft, 13 Dec 1963, *Williams 25930* (NY, WIS). **Sololá**: Volcán Santa Clara, 2100–3000 m, 5 Jun 1942, *Steyermark 47001* (NY); San Pedro La Laguna, Volcán San Pedro, ladera NE del volcán, 14.6572, -91.2664, 3006 m, 28 Jan 2005, *Pardo 27* (CAS). **Totonicapan**: Lake Atitlán, ca 15 km N of Sololá, old road to Chichicastenango, 3000 m, 6 May 1972, *Burch 5949* (MO); María Tecún, [14.8341, -91.2174], 3000–3600 m, 12–23 Jan 1966, *A. Molina R. 16399* (DS, NY); 5–10 km W of Los Encuentros, Cerro María Tecún, Sierra Madre Mountains, 2900–3100 m, 24 Dec 1972, *Williams 41742* (NY).

**Mexico**. **Chiapas**: Mpio. Chamula, Civilhobeletic, Jul 1975, *Anderson s.n.* (DS); Mpio. San Cristóbal de Las Casas, SW of Mexican Highway 190 near Rancho Nuevo, about 14 km SE of San Cristóbal de las Casas, steep slope near crest of ridge, [16.6669, -92.5680], 9000 ft, 20 Aug 1966, *Breedlove 15098* (DS); same location, 28 Jul 1981, *Breedlove 51805* (CAS, MEXU); Mpio. El Porvenir, 3–4 km west of El Porvenir along road from Huixtla to Siltepec, [15.4522, -92.2578], 2800 m, 17 Jan 1973, *Breedlove 31746* (DS, MEXU, MO); Mpio. El Porvenir, NW slope of Cerro Male, 3–4 km W of El Porvenir along road from Huixtla to Siltepec, [15.4522, -92.2578], 2800 m, 19 Sep 1976, *Breedlove 40387* (DS, MEXU, MO, NY); Mpio. Motozintla de Mendoza, on the N and W slope of Cerro Mozotal, below the microwave tower along road from Huixtla to El Porvenir and Siltepec, 3000 m, 22 Nov 1976, *Breedlove 41749* (DS); Mpio. Zinacantan, at paraje Pij, [16.7492, -92.7636], 2460 m, 9 Mar 1981, *Breedlove 50023* (CAS, MEXU, MO, NY); Mpio. Motozintla de Mendoza, Cerro Mozotal, near summit, 2750 m, 24 Nov 1981, *Breedlove 55915* (CAS); Cerro Mozotal, near summit, [15.4261, -92.3424], 2740 m, 27 Nov 1991, *Breedlove 72675* (CAS); Cerro Sontehuits, 10 miles by road from Las Casas [San Cristóbal de las Casas] to Tenejapa, then up trail to top, [16.7067, -92.4670], 3000 m, 28 Jan 1952, *Carlson 2399* (MEXU); Mpio. Chamula, Tzontehuitz, 16.8108, -92.5775, 2740 m, 20 Apr 1999, *Domínguez-T. 70* (MEXU); Mpio. Cristóbal de Las Casas, Estación Biológica Huitepec-PRONATURA, [16.7318, -92.6681], 2500 m, 22 Jan 1991, *González-E. 1244* (MEXU, XAL); same location, 16.7489, -92.6743, 23 Oct 1991, *González-E. 1616* (MEXU); Mpio. Chamula, Paraje Vo’ta Mesté, 16.8178, -92.7572, 2470 m, 12 Feb 1992, *González-E. 1752* (MEXU); Mpio. Motozintla de Mendoza, a 6 km de Motozintla, Cerro Mozotal,15.3661, -92.3486, 3058 m, 15 Jul 2009, *Jonapa 150* (MEXU); Mt. Pasitar, 30 Dec 1936, *Matuda 255* (MEXU; MO; NY); Mpio. Siltepec, Fraylesca, [15.5508, -92.5708], 2000 m, 3 Mar 1945, *Matuda 5259* (CAS, MEXU); Sierra Madre de Chiapas, Saxchanal, [15.5050, -92.6057], 2000 m, 11 May 1948, *Matuda 17812* (MEXU); Sta. Rosa, Escuintla, [15.4472, -92.5339], 25 May 1948, *Matuda 17852* (DS, MEXU); Mpio. Chamula, Cerro Zontehuitz, NE of San Cristóbal de Las Casas, 16.8108, -92.5775, 9000–9400 ft, 25 Jun 1962, *Webster 11741* (DAV).

**39. *Lycianthes rafatorresii* – Paratypes**

**Mexico. Oaxaca**: a 5 km al N de Vista Hermosa, carretera Ixtlán a Valle Nacional, 1175 m, 1 Jul 1982, *Cedillo-T. 1597* (NY, MEXU); Uxpanapa Region, along gravel road from Esmeralda (17 km E of Sarabia) to Río Verde, 1.1 mi S of Esmeralda, 17.1667, -94.7500, 100 m, 18 Jan 1987, *Croat 63216* (MEXU, MO); Oaxaca, 1859, *Cuming s.n.* (G); Mpio. San Felipe Usila, cuenca del Río Perfume (ladera oeste), 4.7 km en línea recta al S de Santa Cruz Tepetotutla, 17.7006, -96.5378, 1280 m, 28 Mar 1995, *Gallardo-Hernandez 1364* (IEB, MEXU, MO); Sta. María Chimalapa Región del Río Verde en área de explotación forestal, ca. 8–10 km en línea recta al N de Sta. María y al S del Arroyo Hamaca, 17.0000, -94.6833, 250 m, 12 Jun 1985, *Hernández-G. 1234* (NY); Mpio. Comaltepec, La Esperanza, 17.6167, -96.3500, 1600 m, 3 Jul 1989, *López-L. 470* (MEXU, NY); same location, 27 Jul 1990, *López-L. 66*8 (MEXU, NY); Sierra Juárez, entre Vista Hermosa a Comaltepec, km 95 entre Tuxtepec a Oaxaca, 1000 m, 11 Sep 1965, *Martínez-Calderón 286* (NY); Mpio. San Miguel Chimalapa, Loma El Calvario, a 2 km al O de la Coralilla (Díaz Ordáz), ca. 37 km en línea recta al N de San Pedro Tapanatepec, 16.7167, -94.1833, 1400–1700 m, 13 Nov 1984, *Maya-J. 889* (NY, MEXU); Mpio. Santa María Chimalapa, San Antonio Nuevo Paraíso, a 2 km en línea recta al oeste, en cerro Camedor, 17.1444, -94.3578, 475 m, 29 Jun 1999, *Perret 352* (IEB, MEXU); Mpio. Santa María Chimalapa, San Antonio Nuevo Paraíso, a 500 m en línea recta al W, por el camino al Plan de la Ceiba, en el panteón, 17.1536, -94.3533, 350 m, 23 Jun 1999, *Rivera-H. 1279* (MEXU); Mpio. Santa María Chimalapa, San Antonio Nuevo Paraíso, a 500 m en línea recta al oeste, por el camino al plan de la Ceiba, 17.1536, -94.3533, 350 m, 28 Jun 1999, *Rivera-H. 1502* (IEB, MEXU); Mpio. Santa María Chimalapa, San Antonio Nuevo Paraíso, a 2 km en línea recta al W, en cerro Camedor, 17.1444, -94.3578, 475 m, 22 Sep 1997, *Torres-B. 1371* (MEXU); Mpio. Comaltepec, 3 km al S de Metates, carretera Tuxtepec-Oaxaca, [17.5871, -96.5065], 10 Sep 1985, *Torres-C. 7270* (IEB, MEXU, MO); **Puebla**: Mpio. Hueytamalco, Limónateno, [19.9110, -97.3076], 1000 m, 12 May 1970, *Ventura-A.1077* (IEB, MEXU, MO); **Veracruz**: Mpio. Mecayapan, ladera NW del Volcán San Martin Pajapan, 3 km (en línea recta) al S de La Valentina, 18.3167, -94.7333, 700 m, 9 Mar 1994, *Bojorquez-Galvan 114* (XAL); Mpio. Soteapan, al SE de Barra de Pilapa, vereda para el Ejido Pilapillo, 5 Jul 1985, *Calzada 11158* (XAL); Mpio. Soteapan, Bastonal, 3–5 km adelante, camino a la Sierra de Santa Marta, 18.4000, -94.9500, 850 m, 25 Nov 1985, *Castillo-Campos 4417* (XAL); Mpio. Mecayapan, Las Tuxlas range, 200 m, 18 May 1986, *LaFrankie 1215* (MO); Mpio. Las Choapas, Rancho El Milago, 5 km en línea recta al suroeste de la colonia Nueva Tabasquenia, 17.5300, -94.0289, 115 m, 5 Jul 2002, *López 139* (IEB, XAL); Mpio. Soteapan, along dirt road 13 km E of Tebanca, 13 km E of E side of Lago Catemao, 800–950 m, 5 Jul 1980, *Nee 18802* (MEXU, MO); Mpio. Catemaco, Bastonal, el camino a Arroyo Claro, 29 Jul 1988, *Soto 1147* (IEB, MEXU, MO); Mpio. Jalacingo, El Bravo, [19.8880, -97.2574], 850 m, 30 May 1970, *Ventura-A. 1192* (MEXU, WIS); Mpio. Atzalan, La Calavera, [19.8623, -97.2425], 1050 m, 4 May 1977, *Ventura-A. 13997* (MEXU, MO); Mpio. Hidalgotitlán, desde el Poblado 6, al S por la brecha y la vereda al horcajo oriental del Río Cuevas, 17.2500, -94.5000, 200 m, 17 Jul 1980, *Wendt 2612* (MO).

**40. *Lycianthes rantonnetii***

**Guatemala. Guatemala**: Guatemala City, Ave. Reforma, 23 Oct 1965, *Andrews 137* (NY); **Huehuetenango**: ca. 25 km (by road) WNW of Huehuetenango on road to Mesilla, 10 Jan 1976, *Iltis 103* (MO).

**Mexico. Baja California**: 21.35, -117.05, 10 m, 14 Jun 1980, *Moran 28811* (MO, NY); **Jalisco**: Mpio. Zapopan, Hacienda Trailer Park, near the Pan American University, just SW of Guadalajara, 18 Aug 1991, *Dean 250* (DAV); **Veracruz**: Ciudad de Orizaba, 15 Jan 1989, *Montrel s.n.* (WIS); Xalapa, 1400 m, 30 Jun 1980, *Nee 18736* (WIS); Xalapa, 1300 m, 12 Nov 1981, *Nee 22986* (WIS).

**41. *Lycianthes rzedowskii***

**Mexico. México**: Mpio. Sultepec, NE of Capula along the road to Sultepec, ca. 2.5 rd km from the outskirts of Capula, 1 rd km NW of the turnoff to Tejupilco, [18.8800, -99.9400], 2317 m, 13 Jul 1990, *Dean 212* (DAV, ENCB, IEB, MEXU, MO, NY, UC, XAL); Nanchititla, Barranca de la Cueva de Santos, downstream from dam, [18.8468, -100.4114], 1794 m, 9 Nov 1991, *Dean 317* (DAV, XAL); Mex. hwy 130, 10.1 miles N of Temascaltepec, [19.1342, -99.9258], 1940 m, 27 Jul 1972, *Denton 1900* (UC); Mpio. Sultepec, 3 km al NE de Capula, [18.8821, -99.9376], 2400 m, 6 Jul 1968, *García-Saucedo 165* (ENCB, IEB); Dto. Temascaltepec, Comunidad, [19.1277, -99.9331], 2610 m, 7 Jul 1932, *Hinton 971* (BM, F, G, MEXU); Dto. Temascaltepec, Nanchititla, [18.8592, -100.4424], 26 Jul 1935, *Hinton 8102* (F, GH, MO, NY); same location, 29 Aug 1935, *Hinton 8234* (GH, K); Mpio. Valle de Bravo, Avandaro, Cerro Gordo, 19.1142, -100.1341, 2301 m, 22 Jun 2011, *Corral 1801* (MEXU); Mpio. Coatepec Harinas, Río Las Flores, km 13.5, carretera Mex. 12, rumbo a Chiltepec, 18.9161, -99.7956, 2086 m, 17 Jun 2011, *Rodríguez-Barquet 189* (MEXU); La Ciénega, 5 km al S de Sultepec, sobre el camino a Amatepec, [18.8191, -99.9678], 2400 m, 15 Jul 1973, *Rzedowski 30884* (CAS, ENCB, F, IEB, MEXU); Mpio. Ocuilan, km 13 a 16 Ocuilan a Cuernavaca, Morelia, 18.9667, -99.4000, 2150 m, 29 Jul 1988, *Socorro L. 30* (MEXU); Along Mexico 130 just SW of La Comunidad [Hwy 130 runs from Pachuca to Veracruz and there is no town of La Comunidad along that highway and this species would not occur in that area. Tropicos has georeferenced this location as along Hwy 134. There is a town of La Comunidad along Hwy 134 due west of Parque Nacional Nevado de Toluca, in the state of Mexico], [19.1277, -99.9331], 29 July 1969, *Weaver 2197* (DUKE); **Michoacán**: Mpio. Zitacuaro, a 1 km al NNE de Rincon de Ahorcados, Reserva de la Biosfera Mariposa Monarca, Cerro El Casique, 19.3722, -100.3042, 2453 m, 30 Jul 2014, *Alvarez 13027* (MEXU); 13 miles E of Morelia, [19.6792, -101.0109], 13 Aug 1947, *Barkley 2786* (F, TEX); 4 km al S de San Miguel del Monte por el camino a Piedras de Lumbre, 19.5917, -101.1206, 2300 m, 1 Sep 2011, *Cornejo-Tenorio 3762* (MEXU); Mpio. Charo, aproximadamente 2 km del Parque José María Morelos (27 km), [19.6622, -101.0045], 2100 m, 9 Aug 2003, *Carranza 6533* (IEB); Mpio. Zitacuaro, San Miguel Chichimequillas, Volcán El Molcajete, 19.3983, -100.3681, 2230 m, 13 Jul 2007, *Corral 362* (MEXU); Mpio. Zitacuaro, San Miguel Chichimequillas La Mesa, 19.4065, -100.3631, 2018 m, 15 Jul 2007, *Corral 376* (MEXU); along route 15 between K268 and 269, ca. 45 km E of Morelia,] , [19.6667, -100.85002830 m, 10 Aug 1966, *Cruden 1171* (MEXU, UC); Mpio. Charo, along hwy 15, 20 rd km E of Morelia, just E of Pontezuelas, [19.6600, -100.9978], 2165 m, 24 Jul 1990, *Dean 220* (DAV, MEXU, XAL); Mpio. Queréndaro, along old hwy 15 ca. 0.4 rd km W of San José de la Cumbre, near the km 195 marker, [19.6678, -100.8266], 2500 m, 25 Jul 1990, *Dean 221* (DAV, MEXU); Mpio. Zitácuaro, near Macho de Agua, E of Zitácuaro, along old hwy 15, ca. 6–8 rd mi E of RR crossing, [19.4421, -100.2447], 2584–2645 m,11 Nov 1991, *Dean 320* (DAV, ENCB, IEB, UC, XAL); Mpio. Charo, waterfall along old hwy 15, E of Morelia, just E of intersection with rd to Tzitzio, [19.6543, -100.9557], 2250 m, 13 Nov 1991, *Dean 322B* (DAV, NY, UC, XAL); same location, 7 Dec 1991, *Dean 336* (DAV, XAL); Mpio. Zitacuaro, Francisco Serrato, [19.5111, -100.2514], 2500 m, 10 Jul 1999, *Heredia 165* (MEXU); Jesús del Monte, near Morelia, [19.6487, -101.1606], *Galeotti 1182* (BR); Zitácuaro, Cerro Pelón, [19.4167, -100.3500], 17 Jun 1938, *Hinton 11967* (GH, K); Mpio. Ciudad Hidalgo, en La Venta, a 17 km al oeste de Ciudad Hidalgo, carretera a Morelia, por Mil Cumbres, [19.6045, -100.4711], 2450 m, 11 Oct 1983, *Martínez-S. 4622* (MEXU); 23 km E of Morelia, km 290 from Mexico City (on old Highway 15), 2600 m, 28 Jun 1964, *Mick 163, 163A* (WIS); km 212–213 carretera Mex. 15, entre Morelia y Ciudad Hidalgo, paraje El Salto de Agua, [19.6500, -100.9667], 27 km al E de Morelia, 1 km al E del crucero a Huetamo, 19.6538, -100.9497, 2215 m, 19 Sep 2004, *Rodríguez-C. 4174* (IBUG, IEB, MEXU, XAL); Pontezuelas, 20 km al E de Morelia, sobre la carretera a Zitácuaro (km 289), [19.6500, -100.9833], 2100 m, 21 Jul 1964, *Rzedowski 18374* (ENCB, WIS); Mpio. Morelia, 3 km al W de San Miguel del Monte, sobre el camino a Atecuaro, [19.6122, -101.1669], 2300 m, 24 Aug 1987, *Rzedowski 44230* (IEB); Mpio. Susupuato, La Ziranda, [19.2333, -100.4000], 1650 m, 26 Sep 1989, *Rzedowski 48988* (IEB); Mpio Morelia, parte alta del Cerro Campanario, 4 km al sureste de Agua Zarca, [19.4220, -101.1688], 2300 m, 27 Jul 2002, *Rzedowski 53965* (IEB); Mpio. Zinapecuaro, cañada del Salta, perteneciente a Bocaneo, [19.8667, -100.8000], 2100 m, 29 Jul 1987, *Santos-Martínez 2134* (DAV, IEB); Mpio. Zitácuaro, camino al cerro Cacique, por Nicolas Romero, 2 km al sureste de Zitácuaro, [19.4057, -100.3310], 2230 m, 16 Sep 1989, *Torres C. 12986* (DAV, IEB); Mpio. Ocampo, Cerro Camacho, ladera SE al O de Ocampo, [19.5838, -100.3255], 2390 m, 17 Sep 1989, *Torres C. 13085* (DAV, IEB, TEX); Mpio. Ocampo, Camino al Salto, al E de Ocampo, [19.5859, -100.3291], 2400 m, 1 Oct 1989, *Torre- C. 13242* (DAV, IEB); Las Peras, 38 km E of Morelia (ca. 33 km air dist.), km 272 on hwy 15 (Ciudad Hidalgo to Morelia), [19.6667, -100.6333], 2515 m, 13 Sept 1962, *Ugent 2012* (WIS); Mpio. Charo, km 23 de la carretera Mil Cumbres, Morelia-Ciudad Hidalgo, [19.6500, -100.9167], 2100 m, 19 Jul 1986, *Zamudio-Ruiz 4147* (DAV, IEB); **Morelos**: Sierra de Morelos, Cuernavaca, 2050 m, 26 Jul 1969, *Hinton 17221* (NY); Huitzilac, [19.0167, -99.2667], 1 Jul 1930, *Lyonnet 713* (BM, GH, MEXU (one duplicate is a mixed collection with *L. acapulcensis*), MO; duplicate at NY is *L. acapulcensis*); hacia el Valle del Tepeite, Canal Zempoala, [19.0053, -99.2586], Aug 1932, *Lyonnet 1002* (CAS, MEXU).

**42a. Lycianthes scandens var. scandens**

**Belize. State unknown**: Sibun River, [17.1984, -88.5909], 1 Dec 1934, *Gentle 1423* (MO); **Belize**: old Northern Highway, mile 34, 17.8833, -88.3167, 24 Aug 1992, *Arvigo 667* (MO); along Northern Highway, vicinity of Maskall River, [17.8793, -88.3112], 6 Jun 1973, *Croat 23938* (MO, NY); savanna at mile 41 on Northern Highway, [17.9209, -88.3472], 6 Jun 1973, *Croat 23959* (MO); mile 42.5 on Northern Highway, N of Maskall River, [17.9262, -88.3613], 7 Jun 1973, *Dwyer 11001* (MO, NY); mile 20–35, Northern Highway, [17.7629, -88.3189], 2 Jun 1974, *Dwyer 12596* (MO); Mile 34–35, Northern Highway, road leading from vicinity St. Anne, [17.8389, -88.3136], 3 Jun 1974, *Dwyer 12625* (MO, NY); Western Highway, near Roaring Creek, [17.2762, -88.7639], 4 Jun 1974, *Dwyer 12707* (MO); River Ridge Plantation near Belize River, 11 miles from Belize on Northern Highway, near sea level, [17.5742, -88.3372], 25, 26 May 1973, *Gentry 7580* (DAV, MO); Belize City Dump, 0.5–1 mi W of city limits on Western Highway, [17.4905, -88.2369], 0 m, 22 Jan 1974, *Liesner 1539* (MO); alrededores de la Ciudad de Belize, [17.5075, -88.2297], 0–50 m, 28 Nov 1981, *Ramamoorthy 2816* (NY); oeste de Tropical Park, 1 May 1982, *Ramamoorthy 3607* (NY); Belize City, 35 Gabourel Lane, [17.4958, -88.1838], 8 Sep 1970, *Ugent 86* (MO); **Cayo**: 3 mi S of Grano de Oro on road between Millionario and La Flor, [16.6286, -89.0272], 2 Jun 1973, *Croat 23401* (NY); **Corozal**: Cerros Maya Ruins, Lowry’s Bight, coastal area, [18.3541, -88.2540], 29 Mar 1983, *Crane 444* (MO); same location, 2 Apr 1983, *Crane 493* (MO); 1 mi west of Northern Highway on secondary road, 1 mi N of Buena Vista, 100 ft, 23 Jun 1973, *Croat 24959* (MO, NY); 2 miles W on secondary road, 1 mile N of Buena Vista, [18.2502, -88.5212], 23 Jun 1973, *Dwyer 11360* (MO); 1 mile west of Northern Highway on secondary road, 1 miles N of Buena Vista, 23 Jun 1973, *Dwyer 11376* (MO, NY); Corozal District, no exact location, 1931–1932, *Gentle 218* (NY); Corozal-Orange Walk Road, [18.1476, -88.5712], Oct 1993, *Gentle 816* (MO, NY); San Andres, [18.3810, -88.3998], Jul 1933, *Gentle 4930* (M, MO, NY); NE side of Corozal, 1 km NE of central square, 18.3967, -88.3750, 2 m, 5 Jun 1996, *Nee 46987* (NY); **Cayo**: near San Ignacio, Chial Road, 17.1000, -89.0667, 9 Mar 1989, *Arvigo 200* (MO); Chial Road, 17.1000, -89.1000, 16 Feb 1993, *Arvigo 736* (MO); **Orange Walk**: Northern Highway at crossing of New River near Tower Hill, [18.0266, -88.5574], 23 Jun 1973, *Gentry 8526* (MO, NY); **Stann Creek**: Gragra Creek, [16.9488, -88.2462], 4 Sep 1953, *Gentle 8024* (MO, NY); All Pines, [16.6802, -88.3241], 5 ft, 29 Aug 1930, *Schipp 606* (MO, NY); **Toledo**: Río Mojo mouth to 8 miles upstream, 0–50 m, 8 Aug 1975, *Dwyer 12982* (DAV, MO); Temash River, [15.9860, -89.0608], 6 Jun 1944, *Gentle 4662* (DUKE).

**Guatemala. Chiquimula**: Jocotán, roadside, [14.8167, -89.3869], 450 m, 1 Nov 2001, *Kufer 273* (MSB, W); vicinity of Chiquimula town, [16.1226, -90.9299], 400 m, 4 Dec 1969, *Molina-R. 25111* (NY); **Izabal**: Finca Mucielago, along Jaqua trail, [15.5823, -89.1455], 22 Jun 1967, *Snedaker E178* (NY); **Petén**: La Libertad and vicinity, [16.7819, -90.1108], 16 Jun 1934, *Aguilar-H. 258* (NY); Ixcanrio [Ikanrio], in bajo, 1 km, 700 m, in El Tintal, NEE, [17.7457, -89.3022], 23 May 1969, *E. Contreras 8626* (MO); N of El Cambio, along Río Machaquila, [16.3225, -89.8413], 75–100 m, 25 Apr 1942, *Steyermark 45984* (NY); Umgebung des Westufers des Lago Petén Itzá, Gebüschsaum a Straßenrand NNE-NE Nuevo San José gegen “La Providencia” (=La Trinidad”), das ist NNE-NE San José, 17.0003, -89.8833, 170 m, 15 Aug 1993, *Wallnöfer 5910* (MO, MSB, NY,W); NW-Ufer des Lago Petén Itzá, 16.9917, -89.8983, 120–140 m, 30 Nov 1994, *Wallnöfer 9577* (MSB, NY, W).

**Honduras. Colón**: Mpio. Trujillo, old road to Castilla from Trujillo, 1 km E of airport, [15.9353, -85.9251], 29 Jan 1981, *Saunders 978* (MO); **Copan**: valley of Ruinas de Copan, [14.8498, -89.1468], 7 May 1970, *Barkley 40282* (DAV); **Cortés**: San Pedro Sula, [15.4997, -88.0548], 22 Aug 1972, *D’Arcy 6879* (MO); San Pedro Sula, 3 Jul 1992, *D’Arcy 18111* (MO); alrededores de San Manuel, [15.3378, -87.9195], 22 Apr 1971, *Hernández-R. 1111* (NY); Mpio. Las Limas, Las Limas, [15.4211, -87.8965], *Jorgensen 20* (WIS); **Islas de la Bahía**: Isla de Roatán, camino entre Roatán y Sandy Bay, [16.3366, -86.5408], 0–50 m, 13–20 Mar 1978, *Nelson 4419* (MO); **Olancho**: márgenes del Río Talgua, 6 km SE de Catacamas, [14.7887, -85.8474], 355 m, 27 May 1987, *Ortega-U. 353* (MO); **Yoro**: El Progreso, [15.2949, -87.7797], 60 m, Oct 1982, *Bados-S. 93* (MO); Mpio. Olanchito, Coyoles Central, valley of Río Aguán, 12 km WSW of Olanchito, (United Fruit Co.), 15.4333, -86.7000, 400 m, 19 Oct 1991, *Iltis 30897* (MO, WIS); camino a las Guanchías, [15.2050, -87.8676], 21 Apr 1971, *Mancías 1022* (MO); Mpio. El Progreso, campos abiertos, [15.1436, -87.1193], 60 m, 14 Apr 1984, *Navarro-O. 177* (MO);

**Mexico. State unknown**: no exact location, *Orcutt 5295* (MO); **Campeche**: en la Ciudad del Carmen, sobre Avenida Camarón, [18.6632, -91.8145], 23 Nov 1987, *Cabrera-C. 14856* (MEXU, MO); **Chiapas**: Mpio. Villa Corzo, near Colonia Vincente Guerrero on road to Finca Cuxtepec, [15.7304, -92.966], 915 m, 10 Aug 1981, *Breedlove 52044* (MO); Mpio. Ocosingo, a 50 km al S de Boca Lacantum, camino a Nvo. Chihuahua, zona Marqués de Comillas, [16.2497, -90.5019], 120 m, 16 May 1987, *Martínez-S. 20915* (NY); Mpio. Ocosingo, en Estación Chajul, [16.1127, -90.9409], 150 m, 21 Julio 1992, *Martínez-S. M-25075* (NY); Mpio. Ocosingo, Estación Chajul, sobre el Río Lacantun, [16.1227, -90.9300], 150 m, 9 Sep 1992, *Martínez-S. 25292* (MEXU); Mpio. Las Garzas, Acapet, Chiapas, [15.2164, -92.7989], 4–7 Jun 1938, *Matuda 2708* (NY); Mpio. Ocosingo, frente a Puerto Rico, [16.8376, -91.7276], *Sinaca-C. 2548* (MEXU); Mpio. Tapachula, Nuevo Mundo, [14.8738, -92.2629], 150 m, 18 Jun 1985, *Ventura 1925* (WIS); **Hidalgo**: just N of Tuxpan, along the road to Tamiahua on the gulf coast about 4–8 miles from its intersection with the Tuxpan bypass on hwy 180, [21.0090, -97.4078], 60 m, 12 Jul 1983, *Taylor 2063* (DUKE); **Jalisco**: Mpio. Cihuatlán, Arroyo Seco, [19.2110, -104.6577], 30 m, 28 May 1990, *Rodríguez-C. 2077* (WIS); **Nayarit**: vicinity of Chacala, ca. 5 miles west of Las Varas, [21.1637, -105.2219], 25–50 m, 14 Sep 1960, *McVaugh 19019* (G, NY [this is a mixed collection of L. scandens var. scandens and L. scandens var. flavicans]); **Oaxaca**: near Huilotepec, [16.2421, -95.1482], 5 Jan 1945, *Alexander 264* (NY); near Mixtequilla, [16.3769, -95.2643], 13 Jan 1945, *Alexander 329* (NY); Mpio. Santiago Tetepec, ca. 5 rd mi NW of Jamiltepec along Hwy 200 between Puerto Escondido and Pinotepa Nacional, S side of rd, [16.3241, -97.8928], 650 ft, 28 Jul 1991, *Dean 238* (DAV); Mpio. Santa María Huatulco, El Botazoo, la bocana del Copalita, 15.7936, -96.0528, 20 m, 21 Oct 2003, *Elorsa-C. 7454* (DAV); Mpio. San Pedro Huamelula, Hacienda del Rosario, Río Seco, 15.9367, -95.7719, 33 m, 22 Aug 2010, *García-Sosa 226* (DAV); Mpio. Santiago Astata, La Tortolita, 15.9772, -95.6072, 74 m, 27 Oct 2009, *Gopar-Vásquez 323* (DAV); Mpio. Santiago Astata, 15.9575, -95.6431, 14 m, 31 Aug 2009, *Petri-L 348* (DAV); Mpio. Santiago Astata, El Roble, 15.9172, -95.6977, 9 m, 5 Nov 2009, *Sánchez-Martínez 2522* (MO); **Quintana Roo**: Mpio. José María Morelos, a 3.4 km al SE de Sabana San Francisco, 19.5200, -89.0750, 86 m, 3 Sep 2004, *Álvarez 10473bis* (DAV); a 10 km al oeste de La Pantera, sobre el camino a Margarita Maza, por la carretera vía corta a Mérida, [19.0800, -88.5500], 5 Aug 1982, *Cabrera 3344* (MO, NY); 21 km al N de Chetumal, sobre el camino a Laguna Guerrero, [18.6851, -88.2705], 19 Aug 1983, *Cabrera 5377* (MO, NY); a 6 km al S de Nuevo Xcan, camino a Cobá, [20.8158, -87.6024], 11 Jun 1980, *Téllez 2549* (MO); a 9 km al SE de Dziuche, carr. F. Carrillo Puerto-Dziuche, [19.8229, -88.7662], 9 Jul 1980, *Téllez 2789* (MO); a 22 km al E de Polyuc, [19.6417, -88.3591], 10 Aug 1980, *Téllez 3148* (MO); **Tabasco**: Mpio. Nacajuca, Colectas de Masateupa, al N de Nacajuca, [18.2089, -93.0081], 17 Jan 1979, *Cowan 1885* (MO, NY); Mpio. Paraíso, Las Flores segunda sección, [18.4204, -93.1975], 12 Sep 1983, *Magaña 1142* (MO); Mpio. Comalcalco, Reyes Hernández, [18.2445, -93.2829], 0 m, 10 Sep 1984, *Ventura-A. 21240* (NY); **Tamaulipas**: Mpio. Tampico, vicinity of Tampico, [22.2361, -97.8748], 15 m, 27–30 Apr 1910, *Palmer 334* (G, MO, NY); **Veracruz**: Mpio. Papantla, Cerro del Carbón, 200 m, 30 Jun 1982, *Cortés 362* (MO); vicinity of Poza Rica, [20.5669, -97.4332], 6 Jun 1987, *Croat 66115* (NY); Cordillera de Veracruz, Vaquería del Jacal, [Note by M. Nee, 1986: This location is on the NE slopes of Pico de Orizaba, elev. ca. 3000 m. The label is surely in error, because this species is restricted to below 300 m. elev in the state of Veracruz], 9750 ft, Jun–Oct 1840, *Galeotti 1176* (BR); Playa Escondida, N of Sontecomapan along Caribbean, [18.5923, -95.0524], 10–60 m, 4 Jun 1981, *Gentry 32616* (MO); Mpio. Tihuatlán, 1.5 km NE of Tihuatlán, along E side of hwy, 20.7333, -97.5167, 125 m, 23 Jun 1980, *Hansen 7395* (MO, NY, WIS); Veracruz, 1855, *Müller 1853* (NY); Mpio. Chalma, 7.5 km of Huejulta on road to Platón Sánchez and Tempoal, 21.2083, -98.3833, 250 m, 24 Oct 1981, *Nee 22344* (NY); Mpio. Tlacotalpan, along the highway following the Río Papaloapan towards the coast, 2 km NE of Tlacotalpan, 18.6333, -95.6500, 2 m, 8 Apr 1983, *Nee 26557* (NY); Mpio. Poza Rica, 5 km SW of jct. of hwys Mex 130 and Mex 180 and N side of Poza Rica, 3 km NE of border with Edo. Puebla and 1 km on gravel road N from Hwy Mex 130, 20.5000, -97.5167, 40 m, 28 Jan 1984, *Nee 29119* (NY); Mpio. Cosamaloapan, W side of Río Papaloapan, 3 km S of Cosamaloapan, 18.3167, -95.8167, 5 m, 1 Feb 1984, *Nee 29243* (MO, NY); Misantla, [19.9338, -96.8443], Aug 1912, *Purpus 5882* (MO, NY); Misantla, [19.9317, -96.8527], Aug 1912, *Purpus 5952* (NY) [A duplicate of this collection at MO is *L. stephanocalyx*]; NW of Túxpam, along Hwy 127 to Panuco, about 18 miles from its junction with Hwy 180, about 42 mi from the Túxpam bypass, 400 m, 12 Jul 1983, *Taylor 2027* (DUKE); Mpio. Martínez de la Torre, Cañadas, [20.0989, -97.0879], 50 m, 8 Sep 1982, *Ventura-A. 19728* (MO); **Yucatán**: Mpio. Chemax, Punta Laguna, 12.5 km al N de Cobá, 20.6458, -87.6313, 28 Sep 2003, de Stefano 1705 (BRIT, MO); Mpio. Pixoy, camino rumbo a San Lorenzo, 20.7147, -88.2625, 22 m, 13 Jul 1988, *Remmers 30* (MO).

**Nicaragua.** [Many of these Nicaragua collections are intermediate with var. flavicans in terms of trichome size and type.] **Carazo**: Río Escalante, Estación Biológica de Chacocente, [11.5399, -86.1881], 27 Feb 1985, *Estrada 112* (MO); **Chontales**: Puerto Díaz, N de Lago de Nicaragua, 11.9667, -85.5000, 14 Sep 1981, *Sandino 1422* (MO); **Granada**: Laguna Blanca, camino a Juan Tallo, 11.7667, -85.9667, 60 m, 25 Feb 1981, *Moreno 7231* (MO); Río Manares, Hacienda Mecatepe, 11.7583, -85.9389, 50 m, 6 May 1983, *Grijalva 2487* (MO); Camino a Casa Tejas, “El Agua Agria,” 11.7667, -85.9667, 80–100 m, 21 Jun 1982, *Moreno 16670* (MO); 1 km N de Intécna, secundo puente en camino hacia el Paso de Panaloya, 11.9333, -85.9333, 24 Jun 1981, *Sandino 736* (MO); **Managua**: camino a Cofradía, ca 15 km al E de Managua, 12.1500, -86.0667, 50 m, 2 Apr 1983, *Grijalva 2454* (MO); Laguna de Jiloá, [12.2279, -86.3129], 134 m, 20 Aug 1981, *Mejía 60* (MO); Mpio. Tipitapa, sobre la carretera a Tisma, ca 2.5 km de San Juan, 12.1667, -86.0500, 40 m, 16 Jul 1980, *Moreno 1136* (MO); El Charco, Río El Carmen, 2 km al oeste de Salamina, 11.9833, -86.6167, 20–40 m, 7 Sep 1981, *Moreno 10811* (MO); costado SE de Laguna Jiloá, 12.2167, -86.3167, 8 Jul 1981, *Sandino 904* (MO); km 18.5 carretera Norte, Río Panamá, 12.1667, -86.1167, 21 Apr 1982, *Sandino 2554* (MO); **Rivas**: Las Piedras, costa del Lago de Nicaragua, a 11 km de la Carretera Panamericana, 11.6333, -85.8500, 40–50 m, 15 Jul 1981, *Moreno 9888* (MO); Isla Ometepe, Volcán Maderas, bordeando la costa desde la Ensenada “Santa Cruz” hasta :Balgüe,” 11.4833, -85.5167, 45 m, 16 Jun 1984, *Robleto 886* (MO); Isla de Ometepe, pantanos entre playa de Finca Santa Cruz y el Istmo de Istián, 11.4833, -85.5500, 17 Jul 1981, *Sandino 1009* (MO); Carretera Granada-Rivas, entreada a Pica-Pica, en Finca Punta de Agua, 11.6166, -85.9333, 40–45 m, 28 Jun 1982, *Sandino 3148* (MO).

**42b. Lycianthes scandens var. flavicans**

**Costa Rica. Guanacaste**: along Río Higuerón near agricultural experimentation area near Taboga, 10.3333, -85.2000, 0–100 m, 29–30 Jun 1977, *Liesner 2746* (MO).

**El Salvador. La Libertad**: Santa Tecla [13.6769, -89.2797], Jun 1922, *Calderón 835* (NY); El Amatal, 13.4833, -89.2833, 26 Jun 1992, *Villacorta 1132* (MO); **La Paz**: carretera que va de Los Blancos a San Salvador, [13.4046, -88.9947], 20 Feb 1959, *Montalvo 3118* (MO); Centro Experimental de Agronomía, UES, Comalapa, [13.7189, -89.2011], 17 Sep 1991, *Montalvo 6202* (MO); **San Miguel**: vicinity of San Miguel, [13.4788, -88.1793], 110 m, 24–27 Feb 1922, *Standley 21152* (NY); south shore of Lake Olomega, 13.2833, -88.0667, 60 m, 1 Feb 1942, *Tucker 871* (NY); **San Salvador**: San Martín, [13.7417, -89.0564], May 1922, *Calderón 703* (NY); **San Vicente**: Laguna de Apastepeque, [13.6887, -88.7453], 4 Mar 1922, *Standley 21356* (NY); vicinity of San Vicente, [13.6387, -88.7836], 350–500 m, 2–11 Mar 1922, *Standley 21710* (NY); **Santa Ana**: matorrales a la orilla de la carretera a Metapán, entre la Hacienda El Milagro y la Hacienda San Cayetano, como 3 km al S de la Ciudad de Santa Ana, 13.9561, -89.6073, 600 m, 25 Oct 1993, *Linares 828* (MEXU); Barranca de Santa Lucía, en el sector NO de la ciudad de Santa Ana, 14.0028, -89.5625, 650 m, 27 Apr 1995, *Linares 2594* (MO); **Usulut*á*n**: along Hwy CA-2, 2 km E of Usuluapan [Usulut*á*n] and W of San Miguel, [13.3517, -88.33], 500 ft, 29 Jul 1979, *Case 162* (MO).

**Guatemala. [Chimaltenango**]: Agua Caliente [Dept. and coordinates from Tropicos, although the coordinates do not map to a place called Agua Caliente.], [14.8, -90.85], 28 Mar 1922, *Greenman 5944* (MO); **El Progreso**: Tulumaje, [14.9286, -90.0313], 346 m, 23 Oct 2003, *Ávila t-71* (BIGU, MEXU); camino de El Rancho a Paso de los Jalapa, 14.9406, -89.9814, 300 m, 9 Feb 2003, *Véliz 12830* (BIGU, MEXU); **Huehuetenango**: between Santa Ana Huista and woods of Rancho Lucas, Sierra de los Cuchumatanes, [15.6791, -91.8151], 800–900 m, 26 Aug 1942, *Steyermark 51365* (MO); **Zacapa**: NW of Zacapa along Río San Juan, off highway CA10 between Zacapa and junction with highway CA9 between El Progreso and Puerto Barrios, 15.0167, -89.6000, 220 m, 12 Feb 1987, *Croat 64723* (MO); San Agustín Acasaguastlan, [14.9480, -89.9647], 300 m, 8 Feb 2003, *Ramírez 254* (MEXU); Mpio. Gualan, 1/2 mile W, [15.1228, -89.3382], 620 ft, 20 Jun 1909, *Deam 6360* (MO, NY).

**Honduras. Comayagua**: Comayagua Valley, [14.4178, -87.6351], 400 m, 4 Sep 1968, *Molina-R. 22684* (MO, NY); **Copán**: vicinity of Copán Ruins town, [14.8503, -89.1467], 500 m, 18 Nov 1969, *Molina-R. 24593* (NY); **Ocotepeque**: El Cerro, vicinity of San Antonio, [13.8747, -87.7016], 1300 m, 30 Aug 1968, *Molina-R. 22463* (MO, NY).

**Mexico. Chiapas**: Mpio. Tuxtla Gutiérrez, roadside on MEX 190 from Tuxtla Gutiérrez to San Fernando, about 8 km NW from the center of Tuxtla Gutiérrez (in a straight line), 16.7944, -93.1836, 733 m, 29 Nov 2012, *Bohs 3920* (DAV, MEXU); Mpio. Tzimol, road from Comitán de Domínguez to Uninajab, roadside; about 3 km after Tzimol, 16.1489, -92.1997, 1150 m, 3 Dec 2012, *Bohs 3941* (MEXU); Mpio. Terán, 6.5 km W of Tuxtla Gutiérrez along Mexican Hwy 190, [16.7732, -93.2058], 600 m, 8 Oct 1971, *Breedlove 20148* (MO); Mpio. Ocozocoautla de Espinosa, near a small spring 13–15 km S of Ocozocoautla along road to Villa Flores, [16.6407, -93.4265], 850 m, 17 Apr 1972, *Breedlove 24589* (MO); Mpio. Cintalapa de Figueroa, 5 km W of Rizo de Oro along Mexican Hwy 190, [16.4479, -94.0878], 820 m, 18 Apr 1972, *Breedlove 24655* (MO); Mpio. Chiapa de Corzo, Above El Chorreadero, [16.7518, -92.9711], 800 m, 9 Jul 1972, *Breedlove 25998* (MO, NY); Mpio. Frontera Comalapa, 6–8 km E of Frontera Comalapa along road to Ciudad Cuauhtémoc, [15.7001, -92.0864], 1000 m, 15 Aug 1972, *Breedlove 26953* (MO, NY); Mpio. Ocozocoautla de Espinosa, steep-walled canyon at the head of the Río de la Venta at the Chorreadero near Derna, [16.9083, -93.6241], 800–1000 m, 24 Aug 1972, *Breedlove 27358* (MO); Mpio. Suchiapa, 15 km SW of Suchiapa along road to Villa Flores, [16.5159, -93.0822], 750 m, 3 Oct 1972, *Breedlove 28232* (MO); Mpio. Ocozocoautla de Espinosa, 20 km W-NW of Ocozocoautla, adjacent to Mexican Hwy 190, [16.8969, -93.4522], 1000 m, 5 Oct 1972, *Breedlove 28365* (MO); Mpio. Ocozocoautla de Espinosa, 15 km W-NW of Ocozocoautla, [16.8652, -93.4510], 800 m, 15 Oct 1972, *Breedlove 29007* (NY); Mpio. Ocozocoautla de Espinosa, steep-walled canyon at the head of Río de la Venta at the Chorreadero near Derna, [16.6992, -93.5551], 800–1000 m, 24 Aug 1974, *Breedlove 36568* (MO2602579); Mpio. Cintalapa, near Francisco Madero, 20 km N of Cintalapa, [16.7962, -93.7540], 1250 m, 25 Aug 1974, *Breedlove 36620* (MO); Mpio. Cintalapa, 5 km W of Rizo de Oro, [16.4480, -94.0874], 900 m, 26 Aug 1974, *Breedlove 36737* (MEXU, MO, NY); Mpio. Acala, bluffs above Presa La Angostura, 45 km from Tuxtla, [16.3930, -92.9633], 700 m, 9 Sep 1974, *Breedlove 37427* (MO); Mpio. Chiapa de Corzo, above El Chorreadero, [16.7544, -92.9713], 800 m, 16 Aug 1976, *Breedlove 39638* (MO); Mpio. La Trinitaria, along small dirt road to Boqueron and Ejido Mujica W of Mexican Hwy 190 at point 18 km SW of La Trinitaria, [16.0039, -92.0152], 900 m, 12 Oct 1980, *Breedlove 46079* (MO); Mpio. La Trinitaria, 18 km S of La Trinitaria on side road to Colonia Morelos and Colonia Chihuahua, [16.0041, -92.0318], 1170 m, 18 Oct 1980, *Breedlove 46484* (MO); Mpio. Ocozocoautla de Espinosa, steep walled canyon at the head of the Río de la Venta at the Chorreadero near Derna, [16.6992, -93.5551], 800–1000 m, 29 Mar 1981, *Breedlove 50479* (MO, NY); Mpio. Chiapa de Corzo, above El Chorreadero, [16.7544, -92.9713], 800 m, 16 Jul 1981, *Breedlove 51555* (MO); Mpio. Tzimol, 15 km S of Comitán on road to Tzimol and Tuxtla Gutiérrez, [16.1491, -92.1995], 1200 m, 18 Jul 1981, *Breedlove 51623* (MO3657674, NY); Mpio. Tuxtla Gutiérrez, on road to Chicoasen, 7 km from Hwy 190, [16.9629, -93.1030], 785 m, 1 Nov 1980, *Fryxell 3239* (MO, NY); Mpio. Tuxtla Gutiérrez, on road to Chicoasen, 7 km from Hwy 190, [16.8319, -93.1916], 785 m, 1 Nov 1980, *Fryxell 3242* (MEXU, NY); 3.5 mi N of the turnoff at Juan Crispin, 5 mi NW of the city of Tuxtla Gutiérrez, [16.0822, -93.1866], 700–750 m, 3 Jun 1973, *Hansen 1643* (MO); Mpio. Tzimol, 1 km al SE del entronque Tzimol-Uninajab, camino a Uninajab, 16.1333, -92.3622, 1110 m, 15 Sep 1988, *Reyes-García 835* (MEXU, MO); Mpio. Ocozocoautla, 1 km al NW del entronque Aeropuerto-Ocozocoautla-Mexico, sobre la carretera 190, [16.7515, -93.3474], 1000 m, 13 Nov 1988, *Reyes-García 1267* (MEXU, MO); Mpio. Tzimol, 6 km al SW de Tzimol, [16.1428, -92.1975], 1150 m, 25 Oct 1989, *Reyes-García 1344* (MEXU, MO); Mpio. Tuxtla Gutiérrez, a orillas de Tuxtla Gutiérrez, al N de Avenida 5a. Norte, [16.7748, -93.1354], 600–640 m, 5 Jul 1990, *Reyes-García 1788* (MO); Mpio. Ocozocoautla de Espinosa, 500 m al S del Rancho El Palmar, 7 km al W de Ocozocoautla, sobre la carretera Mexico 190, [16.6887, -93.5364], 600–700 m, 18 Jul 1990, *Reyes-García 1970* (MEXU); Mpio. Chiapa de Corzo, El Chorreadero, 5.6 mi SE of Chiapa de Corzo, along Mexican Hwy #190, [16.7667, -93.1429], 2400 ft, 20 Sep 1967, *Shilom-T. 2957* (NY); Mpio. Tapachula, Alvaro Obregón, [14.9763, -92.3750], 50 m, 3 May 1985, *Ventura 1640* (WIS); Mpio. Frontera Comalapa, approx. 2.67 km (as crow flies) E of Col. Nuevo Mexico, at Rancho Pilatos, [15.6915, -92.0722], Jan–May 1977, *Voorhies 71-22* (MO); **Colima**: Mpio. Ruiz Cortinez, Tecoman, [18.9293, -103.8923], 25 Mar 1985, *Maillet 85* (MEXU); hills W of Manzanillo bay, 5 mi W of Santiago, thence 2.5 miles on road to the coast at Peña Blanca, [19.1290, -103.4609], 90–150 m, 24 Jul 1957, *McVaugh 15712* (MEXU, NY); hills W of Manzanillo Bay, 5 mi W of Santiago, thence 2.5 mi on road to the coast at Peña Blanca, [19.1291, -104.4574], 90–150 m, 14 Sep 1960, *McVaugh 19019* (G [This specimen has calyx hairs like var. flavicans and leaf veins of var. scandens]); Comala, 17–18 km al NW de Colima, 1 km al S de Campo Cuatro, [19.3138, -103.7604], 1300 m, 15 Aug 1991, *Santana-Michael 5281* (MEXU); **Jalisco**: Mpio. La Huerta, Estación de Biología, Chamela, UNAM, [19.4999, -105.0452], 26 Jul 1982, *Bullock 1210* (F1926450); Mpio. La Huerta, Estación de Biología, Chamela, UNAM; Arroyo Chamela ca. 0.5 km E of Hwy 200, [19.4957, -105.0469], 27 Jul 1982, *Bullock 1213* (F, MEXU); Estación de Biología, Chamela, Eje Central, 825 m, 26 Sep 1985, *Bullock 1681* (F, MO, NY); Estación de Biología Chamela, [19.4980, -105.0445], 150 m, 17 Jul 1976, *Delgado-Salinas 310* (MEXU); Mpio. La Huerta, Eje Central. Estación de investigacion, experimental y difusion Chamela, UNAM, [19.4985, -105.0445], 9 Sep 1985, *Ayala 210* (MEXU); Mpio. La Huerta, Chamela – Cuixmala región; Arroyo Careyes, [19.4500, -105.0330], 7 Jul 1991, *Ayala 91-232* (TEX); Mpio. La Huerta, Estación de Biología Chamela (UNAM), Los Pozos, 19.5000, -105.0500, 27 May 1982, *Lott 1058* (F, MEXU); Mpio. La Huerta, Estación de Biología, Arroyo Chamela, 19.5000, -105.0500, 25 Aug 1982, *Lott 1231* (F, MEXU); Mpio. La Huerta, Pueblo Careyes, a ca. 7 km al sureste de la Estación de Biología Chamela por la carretera Puerto Vallarta-Barra de Navidad. Cañon al E del Pueblo, [19.4383, -105.0232], 25 m, 13 Jul 1986, *Lott 2799* (MEXU, MO, NY); Mpio. La Huerta, Chamela Bay region; Rancho Cuixmala, Río Cuixmala, ca. 0.5 mi downriver from headquarters, 19.3833, -104.9667, 0 m, 11 May 1991, *Lott 3426* (NY); Mpio. La Huerta, Chamela Bay region, Rancho Cuixmala and environs, N side of Río Cuixmala by dirt road heading inland to Cumbres 1 & 2, 1 km E of the Puerto Vallarta-Barra de Navidad (Mex 200) highway, 19.4167, -104.9580, 11 Jul 1991, *Lott 3697* (MO, TEX); Mpio. La Huerta, Rancho Cuixmala. Station 45 km 45 of the Puerta Vallarta-Barra de Navidad Hwy (Mex 200), 19.4167, -104.9960, 19 Aug 1991, *Lott 3807* (MO, NY); Mpio. La Huerta, Estación Biología de Chamela, [19.4985, -105.0445], 30 Jul 1970, *Pérez-J. 228* (F); Mpio. La Huerta, Rancho Cuixmala, along the Río Cuitzmala near the ranch headquarters, from the road crossing to Zapata downstream about 1 km, [19.3800, -104.9800], 10 m, 16 Mar 1991, *Sanders 10509* (MO, NY); Mpio. Arroyo El Colorado, El Rancho El Zarco, Chamela, [19.5092, -105.0530], 2 Aug 1988, *Solis-M. 742* (MEXU); La Huerta, Estación de Investigación, Experimentación y Difusión Chamela, UNAM, 19.4984, -105.0447, 2 Sep 1981, *Solis-M. 3100* (MEXU); Mpio. La Huerta, Eje Central 2400 m, Estación de Investigación, Experimentación y Difusión, Chamela, UNAM, [19.4980, -105.0445], 28 Jun 1984, *Solis-M. 4224* (MEXU); **Michoacán**: Apatzingan, Tancitaro Region. La Majada, [19.1278, -102.4243], 1200 ft, 5 Aug 1941, *Leavenworth 1315* (MO); Mpio. Lázaro Cárdenas, Rancho El Malacate, ca 2 km al S de Solera de Agua, [18.0069, -102.4400], 12 Sep 2010, *Ramírez- Amézcua 1927* (DAV); cuenca media del Río Balsas, a 2 km al NE de la desviación a Aquila, camino a Aquila, carretera Cerro de Ortega-Lázaro Cárdenas, [18.5627, -103.6038], 50 m, 2 Sep 1980, *Soto-Nuñez 2612* (MO); Mpio. Lázaro Cárdenas, Rancho El Malacate. ca 2 km al S de Solera de Agua, [18.0046, -102.4335], 1 Jan 2009, *Steinmann 6533* (DAV); Mpio. Lázaro Cárdenas, Cuatrocaminos, 4 km al N de Playa Azul, rumbo a la desviación a Lázaro Cárdenas-Tecoman, [18.0196, -102.3414], 50 m, 21 Jun 1998, *P. Tenorio L. 19750* (MEXU); **Oaxaca**: Istmo de Tehuantepec, Hwy 185 between Ventosa and Matias Romero, 18.5 km N of jct with Hwy 190, 16.7000, -94.9917, 160 m, 3 Jul 1976, *Breckon 2056* (DAV, MO, WIS); Mpio. Santiago Lachiguiri, a 13 km adelante de Santiago Lachiguiri, cerro Lachiguiri, carretera para las Cuevas, 16.4100, -95.3100, 1135 m, 24 Aug 1994, *Calzada 19278* (MO); Mpio. San Bartolo Yautepec, Toma de Agua, 16.4433, -90.0044, 1036 m, 24 May 2012, *D López-P. 3039* (DAV); Dto. Tehuantepec, Las Animas, [16.6237, -96.0376], 3000 ft, 10 Feb 1971, *Mac Dougall s.n*. (NY); Dto. Tehuantepec, Arroyo Las Minas, Rancho El Limón, El Limón se encuentra 17 km al oeste de Tehuantepec, [16.3663, -95.3849], 23 Apr 1987, *Martínez-Ramírez 923* (MEXU); Mpio. Santiago Astata, Puente Zimatan, 5 km al N, camino a Xadani, [15.9758, -95.6751], 17 Jun 1999, *Martínez-Salas 32419* (MEXU); Mpio. San Miguel Chimalapa, Arroyo El Caracol, lado N del Canion, al noroeste de Benito Juárez en las faldas del Cerro Guayabitos, ca. 39–40 km en línea recta al N de San Pedro Tapanatepec, 16.7333, -94.1667, 1300 m, 24 May 1986, *Maya-J. 3378* (MEXU); Mpio. San Miguel Chimalapa, Río Portamonedas, segundo arroyo al lado E al S de Benito Juárez rumbo a Rancho de los Domínguez, ca. 36 km en línea recta al N de san Pedro Tapanatpec, 16.7000, -94.1333, 950 m, 20 Jul 1986, *Maya-J. 3619* (MEXU); Mpio. Santa María Huamelula. El Recodo, 16.0131, -95.6967, 73 m, 4 Jan 2011, *Molina-Bende 919* (DAV); Mpio. Santa Ana Tavela, la piedra de Cal Dto. San Carlos Yuatepec, 16.6506, -95.8797, 875 m, 21 May 2009, *Nolasco-Reyes 95* (MO); Mpio. San Carlos Yautepec, 16.4339, -96.1036, 862 m, 31 Jul 2009, *Ortiz-Vásequez 89* (DAV); Mpio. Asunción Ixtaltepec, Cerro Verde, a 1.75 en línea recta al NE de Nizanda, 16.6539, -94.9853, 300 m, 5 Nov 2002, *Pérez-García 2260* (MEXU); a 2.5 km de Tuxtla Gutiérrez, camino a Villa Flores, [16.7335, -93.0518], 9 Sep 1981, *Téllez-V. 4685* (MEXU); Mpio. Santa María Ecatepec, Río Otate, al S de Santa María Ecatepec, 16.2333, -95.9167, 800 m, 9 May 1993, *Tenorio-L. 18765* (MEXU); Dto. Tehuantepec, Cerro Marimba al NW de Rincón Bamba, el cual esta a 14 km al oeste de Salinas Cruz, [16.1647, -95.2709], 14 Dec 1983, *Torres-C. 4355* (MEXU, MO); Dto. Tehuantepec, 18 km al suroeste de Buenos Aires, hacia Tenango, entrando por Hierba Santa, 10 km al noroeste de Tehuantepec, [16.3527, -95.3653], 1300 m, 13 Sep 1985, *Torres-C. 7391* (MEXU, MO); **Sinaloa**: Culiacán, [24.7399, -107.4127], 27 Aug-15 Sep 1981, *Palmer 1502* (NY).

**Nicaragua. Carazo**: 1 km al S de Jinotepe, 11.8333, -86.1833, 480–500 m, 31 Aug 1981, *Moreno 10733* (MO); **Chinandega**: faldas de Volcán Cosegüina, [12.9696, -87.5819], 300 m, 24 Apr 1975, *Neill 15* (MO); Potosí, near estuary, 24 Apr 1975, *Neill 7115* (MO); Potosí, “Sitio Santa Julia,” 13.0500, -87.5500, 8–10 m, 26 Oct 1983, *Robleto 71* (MO); **Granada**: en la Isla El Carraco, Isletas de Granada, [11.9145, -85.9142] , 35 m, 11 Aug 1982, *Martínez-S. 1551* (NY); **León**: Mpio. La Paz Centro, 4 km SW del pueblo, 12.3000, -86.7000, 60–80 m, 12 Sep 1980, *Guzmán 1010* (MO); **Managua**: ca. 7 km W of airport on Hwy 1 to Tipitapa, near Lake Managua, [12.1376, -86.3294], 150 m, 17 Mar 1977, *Croat 39036* (MO); **Masaya**: Mpio. Masatepe, del Tanque 2 km al NE, zona de plantaciones de café, 300–400 m, [11.9333, -86.1333], 18 Oct 1980, *M. Guzmán 1329* (MO); **Rivas**: Isla Ometepe, Volcán Concepción, “Los Hatillos,” camino entre Los Rodeos y Los Hastillos, zona con amplios cultivos de Citrullus, 11.5833, -85.6333, 100–200 m, 19 Aug 1984, *Robleto 1011* (MO); Isla de Ometepe, N shore of isthmus, last high (rocky) ground on Volcán Concepcion side, 11.4833, -85.5333, 40 m, 26 Feb 1978, *Stevens 6636* (MO).

**43. *Lycianthes sideroxyloides***

**El Salvador. Ahuachapán**: P.N. El Imposible, San Benito, cima del Cerro Campana, 13.8166, -89.9333, 1400 m, 11 Jun 1997, *Sandoval 1577* (MO).

**Guatemala. Alta Verapaz**: on Cobán Road, between Chiracte and Chapultepec Farm, in clearing betweeen km 284/285, [15.6301, -90.0457], 19 May 1964, *Contreras 4726* (MO); **Huehuetenango**: Mpio. San Mateo Ixtatán, E of the town of Aguacate, along road to town of Yalanhuitz, 16.0430, -91.4674, 1393 m, 15 Aug 2017, *Dean 9512* (DAV); along road from San Ramón to Barillas, 15.8626, -91.2147, 790 m, 15 Aug 2017, *Dean 9515* (DAV).

**Honduras. Atlántida**: above Roma switch of S.F. Co. RR, about 15 mi E of Ceiba, [15.7672, -86.5524], 500 ft, 21 Jul 1938, Yuncker 8588 (MO, NY); **Olancho**: La Muralla Vistor Center and environs, 8 km NNW of La Unión, [15.0833, -86.7333], 1415–1580 m, 4 Jun 1992, *D’Arcy 18127* (MO); **Santa Barbara**: Cerro Santa Barbara, [14.8987, -88.1670], 4200 ft, 21 Jun 1970, *Barkley 40805* (GH); same location and date, *Barkley 40815* (GH); same location and date, *Barkley 40822* (DAV).

**Mexico.** No exact location, 1853, *Müller 968* (NY); **Campeche**: Hopelchen, a 2.2 km al ENE de Chun-Ek [this elevation and location does not make sense for this species. There must be a mistake in the location], 19.1950, -89.1736, 90 m, 26 Aug 2005, *Alvarez-M. 11659* (MEXU); **Chiapas**: Mpio. Ocosingo, a 1.44 km al SE del poblado Frontera Corozal, 16.8053, -90.8722, 154 m, 11 Oct 2004, *Aguilar-M. 11449* (MEXU); Mpio. Ocosingo, a 1.66 km al SE de Frontera Corozal, 16.7244, -90.8681, 135 m, 28 Oct 2004, *Aguilar-M. 11991* (MEXU); Mpio. Ocosingo, a 1.96 km al SE de Frontera Corozal, 16.8031, -90.8678, 140 m, 1 Nov 2004, *Aguilar-M. 12070* (MEXU); Mpio. Villaflores, a 0.5 km al N del poblado Nuevo Independencia, 16.2144, -93.5842, 1324 m, 9 Jul 2004, *Àlvarez 9917* (MEXU); Mpio. Ocozocoautla de Espinosa, 10 miles N of Oczocoautla along road to Mal Paso, 3800 ft, 12 Jun 1965, *Breedlove 10328* (DS); Mpio. Berriozábal, 13 km N of Berriozábal near Pozo Turipache and Finca El Suspiro, [16.8991, -93.3719], 900 m, 9 Oct 1971, *Breedlove 20239* (MO); Mpio. Berriozábal, 13 km N of Berriozábal near Pozo Turipache and Finca El Suspiro, 900 m, 9 Oct 1971, Breedlove *20279* (MO3694001); Mpio. Cintalapa, SE of Cerro Baul on the border with the state of Oaxaca. 16 km NW of Rizo de Oro along a logging road to Colonia Figaroa, [16.5350, -94.1650], 1600 m, 3 Nov 1971, *Breedlove 21818* (MEXU, MO); Mpio. Villa Corzo, at the E base of Cerro Tres Picos near Cerro Bola along a logging road SW of Colonia Agronomos Mexicanos, [16.3483, -93.5330], 1550–1800 m, *Breedlove 25461* (MEXU, NY); Mpio. Larrainzar, near the summit of Chuchil Ton, NE of Bochil, [16.9949, -92.8326], 2700 m, 3 Aug 1972, *Breedlove 26814* (MEXU); Mpio. Cintalapa, SE of Cerro Baul on the border with the state of Oaxaca,16 km NW of Rizo de Oro along a logging road to Colonia Figaroa, 1600 m, 6 Sep 1972, *Breedlove 27618* (MO); Mpio. Ocozocoautla de Espinosa, Cerro Brujo, 20 km south of Ocozocoautla, [16.5947, -93.4381], 1350 m, *Breedlove 29068* (MEXU, MO); Mpio. Cintalapa, at crest of ridge 3 km E of Francisco Madero, NE of Cintalapa, 1250 m, 25 Aug 1974, *Breedlove 36669* (MO); Mpio. Ocozocoautla de Espinosa, 13–18 km S of Ocozocoautla, 900 m, 13 Sep 1974, *Breedlove 37831* (MO); Mpio. Cintalapa, SE of Cerro Baul on the border of the state of Oaxaca, 17 km NW of Rizo de Oro, along a logging road to Colonia Figaroa, 1600 m, 12 Oct 1979, *Breedlove 44417* (MO); Mpio. San Fernando, steep slopes at the tunnel on the road from Tuxtla Gutiérrez to the Chicoasen Dam, 950 m, 14 Jul 1981, *Breedlove 51518* (CAS, MO); Mpio. La Trinitaria, 10 km ENE of Dos Lagos above Santa Elena, [16.1107, -91.5646], 1170 m, 15 Aug 1981, *Breedlove 52286* (CAS, LL, MEXU, MO, TEX); same location and date, *Breedlove 52308* (MO); 15 km al E de Tzizcao, sobre el camino a Santa Elena, [16.1086, -91.5641], 23 Jun 1982, *Cabrera-C. 2978* (MEXU, MO, NY); Mpio. Las Margaritas, 12 km E of Tziscao, along carretera fronteriza, [16.1065, -91.5763], 1200–1300 m, 16 Nov 1984, *Davidse 29914* (MEXU, NY); Mpio. Berriozábal, along road from Berriozábal to Las Maravillas, ca. 9 km from the center of Berriozábal. S of the town of Efraín A Gutiérrez, Colonia la Ventana, 16.8686, -93.2886, 1036 m, 12 Sep 2017, *Dean 9526* (DAV); Mpio. Ocosingo, la comunidad Lacandona de Naha, 27 km al sureste de Palenque, por la carretera fronteriza hasta el crucero Chacala, despues de 55.6 km por el camino de terracería hacia Monte Libano, [17.0167, -91.6333], 900 m, 16 Jun 1999, *Duran-Fernandez1250* (XAL); same location, 12 Jul 1999, *Duran-Fernandez1282* (XAL); Mpio. Tuxtla Gutiérrez, Mirador El Roblar, Parque Nacional Cañón del Sumidero, 16.7972, -93.0897, 940 m, 23 Aug 2007, *Espinosa-J. 273* (MEXU); Mpio. Siltepec, Cascada, Siltepec, [15.4864, -92.4047], 1600 m, 1 Mar 1945, *Matuda 5146* (F, MEXU, MO); Mpio. La Trinitaria, carretera terracería, km 20, Col. Cuauhtemox. [16.1067, -91.6119], 10 Aug 1984, *Méndez-Girón 7851* (MO); Selva Negra (N.O. Pueblo Nuevo S), [17.2171, -92.9640], 1200–1300 m, 3 Mar 1953, *Miranda 7761* (MEXU); Cerro del Boqueron, [15.2400, -92.3000], Aug 1913, *Purpus 7106* (GH; [other duplicates of this collection number at GH and NY are *L. arrazolensis*]); 11 km S of Crucero El Piñal, 2 km SE of El Piedron, 17.1670, -91.7500, 600 m, 26 Sep 1988, *Stevens 25899* (MEXU, MO); Mpio. Tenejapa, Río Seco Ch’ish Tontik, [16.9257, -92.4503], 900 m, 5 Jul 1982, *Méndez-Ton 4390* (MEXU, NY); Mpio. Yajalon, Arroyo Azufre, [17.1645, -92.3310], 830 m, 11 Jun 1983, *Méndez-Ton 6320* (MEXU); Mpio. La Trinitaria, Carretera terracería, km 20 Col. Cuauhtémoc, [16.1067, -91.6119], 10 Aug 1984, *Shilon-Ton 7851* (MEXU); Mpio. Tuxtla Gutiérrez, 17 km al NE de Tuxtla Gutiérrez Chiapas, Cañón del Sumidero, [16.8303, -93.1019], 1300 m, 1 Oct 1984, *Torres-C. 6394* (MEXU); Mpio. Ocozocoautla de Espinosa, camino Horizonte a Santa Laura, [16.6776, -93.4176], 900 m, 12 Jun 1983, *Vazquez-B. 1015* (XAL); **Guerrero**: Mpio. Atoyac de Alvarez, 15 km al NE de El Parasio, [17.3574, -100.2194], 1100 m, 19 Aug 1985, *Soto-N. 10103* (MEXU); **Oaxaca**: Mpio. San Jeronimo Coatlan, 45 km al SW de Sn Jeronimo Coatlan, brecha a Progresso, 16.1500, -97.0167, 24 Jun 1990, *Campos 3116* (MEXU); Mpio. San Miguel Chimalapa, paraje palmero “El Venado” cerca del parteaguas continental en la region de Cerro El Retén, ca. 23 km en linéa recta al NNE de Zanatepec, 16.6833, -94.2667, 1700 m, 28–29 Jul 1986, *Maya-J. 3675* (MEXU); Mpio. Santa María Chilchotla, aprox. 6.3 km del Puerte de Fierro, por la terracería a Santa María Chilchotla, 18.1939, -96.8453, 1226 m, 4 Jul 2001, *Munn-Estrada 1313* (XAL); Mpio. San Miguel del Puerto, El Enjambre, camino a la Constancia, [15.9771, -96.1264] 1412 m, 27 May 2005, *Pascual 1524* (IEB, MEXU); Mpio. Santa María Jacatepec, predio La Joya del Obispo, [17.8599, -96.2060], 12 Aug 1990, *Ramos 422* (XAL); same location and date, *Ramos 434* (XAL); Mpio. San Miguel Chimalapa, cerro Salomón, ca. 2 km en línea recta al NNO del Cerro Guayabitos, ca. 43 km en línea recta al N de San Pedro Tapanatepec, ca. del límite con Mpio. De Sta. María Chimalapa, parte baja del filo, al S del cerro, 16.7500, -94.1917, 1850 m, 23 Dec 1985, *Wendt 5176* (NY); **Veracruz**: Mpio. Atoyac, Miraflores, 6 km al noroeste de Atoyac, 18.9500, -96.8167, 900 m, 18 May 1985, *Acevedo-R. 180* (IEB, XAL); Mpio. Atoyac, Cerro Infiernello al W del Rancho Santa Rosa, 18.9500, -96.7833, 700 m, 18 Jul 1985, *Acevedo-R. 326* (XAL); Orizaba, 1886, *Botteri 373B* (NY); same location, Aug 1854, *Botteri 855* (G); same location, Feb 1854, *Botteri 926* (G); Mpio. Chicontepec, El Mirador, 21 km E of Huatusco at km 45 along Hwy to Puente Nacional, 1200 m, 23 Aug 1997, *Croat 44003* (MO); Mirador, [19.2167, -96.8833] 3000 ft, Jul 184X [date missing last digit of year] *Galeotti 1157* (BR); Mpio. Ixtaczoquitlan, Cerro Buenavista, 18.8944, -97.0375, 1260 m, 31 Aug 1995, *Juárez-L. 674* (XAL); same location, 1255 m, 31 Aug 1995, *Juárez-L. 719* (MEXU, XAL); Mirador, Aug 1841, *Liebmann 1325* (NY); Mirador, *Linden 233* (BR); Orizaba, [18.8611, -97.1250], 1855, *Müller 968* (NY, W); Mpio. Coatepec 2 km al E de San Marcos, [19.4388, -96.9552], 7 Apr 1990, *Orea-L. 732* (XAL); Mpio. Jilotepec 2 km despues de la desviacion rumbo a la Concha, 19.6000, -96.8833, 1130 m, 25 Jun 1976, *Ortega 279* (IEB, XAL); Mirador, Feb 1922, *Purpus 323* (M); Mpio. Zacuapan, [20.4330, -98.3440], Jun 1916, *Purpus 7666* (MO); same location, Sep 1928, *C.A. Purpus 12070* (NY); same location and date, *Purpus 13010* (MO); Mpio. Ocozocoautla, 6 km del Pueblo de Ocozocoautla, al cerro Horizonte, 1250 m, 9 Jun 1983, *Vásquez-B. 887* (XAL); Mpio. Totutla, El Mirador, [19.2122, -96.9520], 950 m, 22 Sep 1972, *Ventura-A. 6091* (IEB); Mpio. Xalapa, Santa Rosa, [19.5547, -96.8805], 1200 m, 3 Jun 1974, *Ventura-A. 10074* (IEB, MEXU); Mpio. Xalapa El Castillo, [19.5459, -96.8499], 1100 m, 09 Jul 1974, *Ventura-A. 10265* (IEB, MEXU, XAL); Mpio. Xalapa, Martines de Chicago, [19.4950, -96.9133], 1300 m, 25 Jun 1975, *Ventura 11503* (MEXU, XAL); Mpio. Huatusco, Tepezingo, [19.1803, -96.9491], 1300 m, 2 Aug 1976, *Ventura 13129* (MEXU, XAL); Mpio. Totutla, Encinal, [19.2097, -96.8242], 30 Nov 1977, *Ventura 14787* (XAL); Mpio. Xico, 2 km al E de San Marcos, 19.4278, -96.9633, 700 m, 4 Jul 1990, *Zamora-C. 2604* (IEB, XAL); 2 km carretera a La Concepcion, [19.6114, -96.9186], 1130 m, 25 Jun 1976, *Zola-B. 451* (IEB, MEXU, NY, XAL).

**Nicaragua. Jinotega**: Cordillera de Darien, rain forest, 28–31 mi NE of Sebaco, [13.0479, -85.9643], 4200–4800 ft, 21 Jul 1962, *Webster 12499* (DAV).

**44. *Lycianthes starbuckii***

**Mexico. México**: Dto.Temascaltepec, outskirts of town of Nanchititla in the Sierra de Nanchititla, above the settlement of Palo Verde, near the Ojo de Agua de Palo Verde, [18.8823, -100.4300], 6400 ft, 9 Nov 1991, *Dean 318* (DAV, UC, XAL); Nanchititla, [18.8391, -100.4138], 18 Aug 1933, *Hinton 4550* (BM, G, K, MEXU, MO); Mpio. Luvianos, km 15 carretera El Estado-cañadas de Nanchititlan, camino Torrecillas, 18.8744, -100.3326, 1934 m, 27 Jul 2010, *Rodríguez-C. 6083* (IBUG, IEB, MEXU).

**45. *Lycianthes stephanocalyx***

**Belize**. **Belize**: mile 33 1/2 beyond Hattieville, Rich Woods Farm, 6 Jul 1972, *Dwyer 10112* (MO); Cayo: 37 Miles Section, Humming Bird Highway, 17.0017, -88.4466, 5 Nov 1955, *Gentle 8928* (LL, MO, NY); **Toledo**: Trail from Columbia Forest Station to Esperanza, 2 to 4 miles W of San José road, [16.3120, -89.0647], 600–1100 ft, 13 Jun 1973, *Gentry 8159* (MO3698371).

**Guatemala**. **Huehuetenango**: Sierra de los Cuchumatanes, between Xoxlac and Nucapuxlac, [15.3094, -91.4894], 1650–2500 m, 17 Jul 1942, *Steyermark 48960* (NY); **Izabal**: Mohanal, trail from Espiritu Santo to Las Playitas, [15.6077, -89.1146], 10–18 May 1919, *Pittier 8532* (NY); **Petén**: Dolores, bordering Río Mopan, in clearing 6 km SE, [16.5089, -89.4065], 29 Jun 1961, *Contreras 2566* (MO).

**Honduras**. **Atlántida**: near San Francisco, [15.6498, -87.0531], 2 May 1970, *Barkley 40239* (DAV123359); vicinity of La Ceiba, near the Congrejal River, foothills back of Ceiba, 5 Jul 1938, *Yuncker 8193* (MO, NY); **Copán**: 4 miles NE of Santa Rita, [14.8716, -89.0945], 700 m, 28 Aug 1975, *Molina R. 30832* (MO); **Cortés**: Aldea de Corinto y alrededores frontera con Guatemala, 55 km al O de Puerto Cortés, [15.5450, -88.0237], 9–11 Aug 1975, *Nelson 2824* (MO).

**Mexico**. **Chiapas**: Mpio. Ocosingo, Restaurante la Escondida, en la 11 de Julio, camino a Palenque, 17.1772, -91.4881, 163 m, 12 Jun 2002, *Aguilar 1380* (DAV); Mpio. Ocosingo, a 12 km al NW del crucero de Bonampak, 16.6872, -91.0325, 299 m, 12 Feb 2003, *G. Aguilar 5584* (DAV); Mpio. Barriozábal, along road from Berriozábal to Las Maravillas, ca. 1.4 km south of the town of Efraín A Gutiérrez, remnant of tall forest called La Mata Café, 16.8711, -93.2956, 1005 m, 12 Sep 2017, *Dean 9529* (DAV); vicinity of Palenque ruins, 17.4833, -92.0500, 200 m, 3 Jul 1969, *Marcks 967* (MO); Mpio. Ocosingo, 5 km al S de Campamento COFOLASA, el cual está a 24 km al SE de Crucero Corozal, camino Palenque-Boca Lacantum, [16.6557, -90.8063], 220 m, 24 Sep 1984, *Martínez-S. 7847* (NY); Mpio. Ocosingo, a 6.4 km al SSE de Nuevo Guerrero, 16.9319, -91.2619, 380 m, 9 Aug 2002, *Martínez-S. 35582* (MEXU); Mpio. Ocosingo, 5 km al S de Frontera Echeveria, sobre la orilla del Río Usumacinta, [16.8106, -90.8661], 80 m, 4 Dec 1984, *Martínez-S. 9121* (MEXU, MO); Mpio. Ocozocoautla, on road to Malpaso, 20 mi NW of Ocozocoautla, 17.0000, -93.5000, 1700 ft, 16 Aug 1972, *Webster 17907* (DAV); **Hidalgo**: twelve miles south of Tamazunchale, 5 Jul 1947, *Barkley 7261* (MEXU); Mpio. Chapulhuacan, 53 km al NE de Zimapan, [21.1661, -98.9166], 1000 m, 7 Nov 1979, *Hernández-M. 3898* (MEXU); Mpio Chapulhuacán, 3 km al NE de Chapulhuacán, 900 m, 7 Nov 1979, *Hernández-M. 3905* (MEXU); Mpio Chapulhuacán, near Chapulhuacán, km 340 of highway, 20 Aug 1943, *Lundell 12407* (LL); **Oaxaca**: 15 km N of Valle Nacional along road to Oaxaca City, [17.7705, -96.2986], 150 m, 5 Jan 1982, *Breedlove 56697* (MEXU, MO); alrededores de las bombas de auxilio de la presa de Temazcal, [18.2336, -96.4044], 22 Sep 1984, *Cabrera C. 7237* (MEXU); Mpio. Santa María Jacatepec, en el poblado La Joya, del ejido Corriente Ancha, [17.8750, -96.0204], 150 m, 17 Jul 1990, *Calzada 15416* (MEXU); Mpio. Santa María Jacatepec, al NO del poblado La Joya parcela de Melquiades, 17.8750, -96.0204, 22 Jun 1990, *Calzada 15636* (MEXU); Dto. Tehuantepec, Mpio. Santa María Guienagati, 3 km al N de Santa María Guienagati, carretera a Guevea de H, 16.7167, -95.3667, 460 m, 27 Aug 1991, *Campos-V. 3849* (MEXU932503, MEXU1163915); Dto. Tuxtepec, presa Temascal, camino a los vertederos, 90 m, 8 Sep 1985, *Cortes 30* (MEXU927219); same location and date, *Cortes 40* (MEXU1237410); Dto. Tuxtepec, Mpio. Soyaltepec, 500 m al SE del vertedor de la presa Temascal, [18.2329, -96.3930], 70 m, 24 Nov 1986, *Cortes 572* (MEXU571639, MO5074257); Dto. Tuxtepec, Mpio. Soyaltepec, 4 km al S de la Hidroeléctrica Temascal, 60 m, 22 Aug 1986, *Cortes-A. 390* (MEXU1138771); Dto. Tuxtepec, Mpio. Soyaltepec, Cerro Verde al sureste de Temascal, 18.2167, -96.3500, 300 m, 19 Aug 1987, *Cortes-A. 974* (MEXU1194594); Mpio. Santa María Chimalapa, Paso Lagarto (Huacatapac) del Río del Corte ca. 5 km al N de Sta. María, 16.9333, -91.6833, 180 m, 16 Aug 1984, *Hernández-G. 350* (NY); hacia Cerro de la Bola Tuxtepec, [18.0845, -96.2063], 16 Sep 1947, *Miranda 4248* (MEXU72979); Mpio. Soyaltepec, Presa Temascal, [18.2372, -96.4046], 150 m, 10 Aug 1981, *Ramos s.n.* (MEXU1361259); **Puebla**: road (575) Cuetzalan to San Antonio Rayón [Santiago Yancuictalpan], 20.0617, -97.4706, 592 m, 10 Nov 2014, *Acevedo-Rodríguez 16044* (DAV); Sierra Nororiental de Puebla, Mpio. Cuetzalan del Progreso, Tacuapan, just before the main road into town starts going up toward the church, 20.0821, -97.4738, 312 m, 9 Sep 2014, *Amith 2134* (DAV); **Querétaro**: Mpio. Landa, 2 km al sureste de Neblinas, Río Tancuilin, [21.2660, -99.0538], 610 m, 12 Jun 1990, *Rubio 1953* (DAV, IEB); Mpio. Landa, 2 km al sureste de Neblinas, Río Tancuilin, [21.2660, -99.0538], 610 m, 12 Sep 1990, *Rubio 1954* (DAV, IEB); 74 miles N of Zimapan, 22 Aug 1957, *Waterfall 14257* (BRIT); **San Luis Potosí**: San Antonio, [21.6180, -98.9039], 7 Sep 1978, *Alcorn 1649* (TEX); Tamazunchale (Poistapa), [21.2569, -98.7945], 12 Jul 1937, *Edwards 520* (TEX); same location, 22 Jul 1937, *Edwards 913* (WIS); Tamazunchale, [21.2569, -98.7945], 175 m, Jul 1937, *Lundell 7096* (NY); 7 miles E of Xilitla, 3000 ft, 18 Jul 1963, *McGregor 886* (LL); Tamasopo, [21.9187, -99.3941], 400–500 m, 8–9 Aug 1934, *Pennell 17998* (NY); **Tabasco**: Mpio. Tenosique, a orillas del Chinilkija en el ejido Linda Vista, [17.4058, -91.5075], 2 Aug 1990, *Magaña 2299* (MEXU717293); **Veracruz**: Mpio. Atoyac, Cerro La Perla, 3 km al sureste de Miraflores, 18.9500, -96.8000, 900 m, 17 May 1985, *Acevedo-R. 92* (IEB, MEXU); Mpio. Atoyac, La Joya a 1.5 km aproximadamente al NW del rancho de Santa Rosa, 18.9500, -96.2667, 600 m, 20 Jun 1985, *Acevedo-R. 207* (MEXU); Mpio. Atoyac,Vara Negra, 3 km al nor-noroeste de Atoyac, [18.9167, -96.2667], 650 m, 17 Jul 1985, *Acevedo-R. 287* (IEB, MEXU); Mpio. San Andrés Tuxtla, Laguna Encantada, 8 km al N de San Andrés Tuxtla, [18.4628, -95.1883], 22 Nov 1986, *Calzada 12988* (MEXU); ca. 6 miles S of Acayucan, 1 mile W of Sayula de Alemán, [17.8809, -94.9749], 90 m, 3 Jul 1977, *Croat 40020* (MO); about 4 rd miles from cuota road to Veracruz (Hwy 150), along old rd 150 between Orizaba and Fortín, N side of rd, 19.1791, -96.1769, 3600 ft, 26 Sep 1991, *Dean 265* (DAV); Mpio Coetzala, along old stone rd between Coetzala and Cuichapa, about 1–1.5 km from Coetzala, [18.7849, -96.9104], 2300 ft, 26 Sep 1991, *Dean 267* (DAV, MEXU); Mpio. Coetzala, 3 km WNW of Cuichapa on road to Coetzala, 1.5 km SE of Coetzala, 18.7833, -96.9000, 550–700 m, 3 Jul 1982, *Diggs 2716* (BRIT); Mpio. Cuichapa, 400 m al N de los Xúchiles, 565 m, 5 Jul 1990, *Fernández- C. 98* (MEXU); Mpio. Ixtaczoquitlán, Cerro Buenavista, [18.8944, -97.0375], 1335 m, 3 Oct 1995, *Juárez-L. 861* (MEXU); Mpio. Santiago Tuxtla, Acahual, [18.4615, -95.3019], 30 m, 10 Jul 1967, *Martínez-C. 1448* (MEXU, MO); Mpio. San Andrés Tuxtla, Salto de Eyipantla, 5 km by air S of San Andrés Tuxtla, [18.4221, -95.1444], 150 m, 4 Dec 1981, *Nee 23611* (MEXU, MO); Mpio. Ixtaczoquitlán, 5 km SW of Fortín, [18.8790, -97.0233], 1050 m, 7 Dec 1981, *Nee 23847* (MEXU); Misantla-Veracruz, [19.9317, -96.8527], Aug 1912, *Purpus 5952* (MO); Zacuapan, [20.4284, -98.3611], Jul 1915, *Purpus 7519* (MO, US); Mpio. Amatlán de los Reyes, 400 m al sureste del puente del Río Negro en La Patrona, [18.8684, -96.8575], 700 m, 19 Sep 1990, *Ramón 246* (IEB, WIS); Mpio. Zontecomatlán, 6 km en línea recta al sureste de Zontecomatlán, ejido Cabellete, 20.7172, -98.3667, 800–1100 m, 8 Sep 2000, *Rincón-G. 1869* (IEB, MEXU); Mpio. San Andrés Tuxtla, por la carretera cerca Máquina Vieja y Salto de Agua, al N de San Andrés, [18.4631, -95.1883], 10 Nov 1975, *Shapiro 218* (MEXU); Mpio. Santa Rosa, Los Tuxtlas, [18.4619, -95.1681], 14 Sep 1974, *Sousa 4442* (MEXU); Mpio. Soteapan, Benito Juárez a la orilla del camino rumbo Pop soj nas, [18.2300, -94.9062], 600 m, 6 Jul 1999, *Leonti 71* (MEXU); Mpio. Tlapacoyan, Río Zordo, [19.9817, -97.2125], 200 m, 11 Aug 1981, *Ventura 18934* (MEXU).

**46. *Lycianthes surotatensis***

**Mexico. Colima**: southwestern foothills of the Nevado de Colima, 1–1.5 miles above (south of) Hacienda San Antonio, [19.4285, -103.7194], 1200–1250 m, *McVaugh 16084* (MEXU); **Guerrero**: 20 miles S of Chilpancingo, 13 Apr 1960, *Smith M-75* (TEX); Mpio. Mochitlan, Agua de Obispo, 17.3139, -99.4667, 1050 m, 17 Oct 1963, *Kruse 1032* (IEB, M, MEXU, MO); **Jalisco**: Mpio. Tecalitlán, Sierra del Halo, Cañada La Jabalina, 12 km en línea recta al E de Pihuamo, 3.5 km al oeste de Alotitlan, 1750 m, 23 Feb 2012, *Castro 2665* (XAL); Mpio. Tecalitlán, Sierra del Alo, como 20–25 kms sobre la brecha de Tecalitlán a Jilotlán de los Dolores, [19.3758, -103.0914], 9 Jul 1987, *Cházaro-B. 4856* (IEB); Mpio. Casimiro Castillo, along road between Autlán and Barra de Navidad, just S of Autlán, Puerto de los Mozos, along rd. to radio tower, left side of rd, [19.6984, -104.3912], 5250 ft, 2 Dec 1991, *Dean 334* (DAV); Sierra de Manantlán Biosphere Reserve, Quince Ocotes area, [19.4469, -104.2678], 1900–2000 m, 27 Mar 1991, *Gentry 73592* (NY, MO); 11 miles from Autlán, along hwy SW of Autlán, toward Manzanillo, 1200–1500 m, 8 Apr 1949, *McVaugh 10211* (NY); Mpio. Casimiro Castillo, Puerto de Los Mazos, brecha a la Est. de Microondas, [19.6750, -104.3917], 1500 m, 9 Jul 1987, *Rodríguez-C. 909* (MEXU); 11 km SE of highway 110 on a lumber road leaving highway 12 km SSW of Tecalitlán and extending to San Isidro, 19.3000, -103.1333, 2200 m, 25 Sep 1965, *Roe 2113* (WIS); about 11 miles SSW of Autlán toward La Resolana, [19.6816, -104.4184], 4000 ft, 14 Aug 1949, *Wilbur 2340* (MEXU); **Michoacán**: Mpio. Coalcomán, approx. 4 km (en línea recta) al S de Puerto La Bufa, 25 May 2008, 18.4622, -102.9881, *Ramírez- Amézcua 1320* (DAV); **Nayarit**: Mpio. Tepic, 1 km al SW de El Cuarenteño, camino a El Cora, o 4 km al N del entronque del camino El cora-Palapitas, [21.4544, -105.0357], 820 m, *Flores-Franco 3457* (MEXU); Mpio. Tepic, 15 km sobre la desviación a El Cuarenteño, carretera Tepic-Miramar, [21.4764, -105.0006], 1330 m, *González 945* (MEXU); **Oaxaca**: Dto. Pochutla, Mpio. Pluma Hidalgo, Cerro Espino, al oeste de la Finca Cafetalera “Monte Cristo,” 15.8667, -96.4000, 1250 m, 19 May 1988, *Campos-Villanueva 1946* (MEXU, XAL); Dto. Pochutla, Río Concordia, finca 15 km al N de Chacalapa cafetal, 26 Apr 1983, *Cedillo-Trigos 2300* (MEXU); ca. 56 miles S of Miahuatlán on road between Oaxaca and Pochutla, 6.9 miles N of turn-off to Pluma Hidalgo [15.9859, -96.5249], 1480 m, 20 Jan 1979, *Croat 46110* (MEXU, MO); La Soledad [probably in southern Oaxaca, because on February 9, Ernst collected near Pochutla], 8 Feb 1966, *Ernst 2523* (US); Dto. Pochutla, a 7.9 km NE de Chacalapa por camino a Finca Monte Cristo, [15.8742, -96.4198], 680 m, 1 Jul 1984, *Hernández 411* (DAV, MEXU, MO); vicinity of Cafetal Concordia, 400–650 m, 1–15 Apr 1933, *Morton 2469* (MO); Dto. Pochutla, Mpio. San Miguel del Puerto, Cerro de la Virgen, 15.9673, -96.1095, 1662 m, 1 Apr 2006, *Pascual 1826* (MEXU); Dto. Pochutla, vicinity of Concordia, 630 m, 27 Feb 1937, *Makrinius 790* (US); Dto. Pochutla, Mpio. San Miguel del Puerto, Palo de Agua, camino de San Felipe la Chillo, 15.9833, -96.1167, 1168 m, 1 Oct 2009, *Pascual 2183* (DAV); Dto. Pochutla, Mpio. San Pedro Pochutla, La Alianza, 1 km al S rumbo a la Herradura, 15.8817, -96.3917, 650 m, 4 May 2001, *Saynes-Vazquez 2142* (MEXU); Dto. Pochutla, Río Concordia, 1 km al NE de la Finca Cafétalera Concordia, entrada 5 km de Chacalapa Carr. Pochutla-Oaxaca, [15.8814, -96.4323], 670 m, 18 Mar 1983, *Tenorio-L. 3534* (MEXU, NY); same location and date, *Tenorio-L. 3557* (NY); Dto. Pochutla, 6.5 km al E de la desv. a Totoltepec hacia las fincas Dolores y Independencia, la desv. está 4 km al N de Chacalapa, 270 m [this elevation is likely a typo and should read 670 m], [15.9030, -96.4313], 19 Feb 1984, *Torre-C. 4687* (MEXU); Dto. Pochutla, 9 km al NE de Chacalapa, camino a la Finca Montecristo, [15.8786, -96.3963], 1 Jul 1984, *Torres-C. 5456* (MEXU); Dto. Pochutla, Mpio. Chacalapa, 6.5 km de la desviacion a Totoltepec, de la carretera Pochutla-Oaxaca, 15.7500, -96.4500, 570 m, 19 Feb 1984, *Tenorio-Lezama 5502* (MEXU); Mpio. Santa María Ecatepec, Cerro Zapote, a 88 km en LR (325 N) de Santa María Zapotitlán, 16.1203, -95.8475, 1600 m, 15 Mar 2006, *Velasco-Gutiérrez 1248* (MEXU); **Querétaro**: Mpio. Pinal de Amoles, aprox. 3 km al SSW de Escanelilla, [21.1500, -99.5500], 1300 m, 8 Mar 1989, *Carranza-G. 1528* (DAV); same location and date, *Carranza-G. 1538B* (IEB); Mpio. Pinal de Amoles, ± 4 km al SE de Santa Agueda, [21.2468, -99.6235], 1190 m, 18 Apr 1989, *Carranza-G. 1634* (DAV, IEB); Mpio. Pinal de Amoles, ± 3 km al SSW de Escanelilla, [21.1500, -99.5500], 1280 m, 20 Aug 1991, *Carranza-G. 3426* (IEB, MEXU); Mpio. Pinal de Amoles, La Cuesta, 3 km al S de Escanelilla, [21.1411, -99.5089], 1100 m, 14 Dec 1994, *Fernandez-N. 2109* (MEXU); 1 km al S de Escanelilla, sobre la carretera a Pinal de Amoles, [21.1867, -99.5700], 1250 m, 18 May 1987, *Rzedowski 43384* (CAS, DAV, IEB); Mpio. Pinal de Amoles, 9 km al S de Santa Agueda, sobre el camino a Ahuacatlán, [21.2417, -99.6317], 1150 m, 13 May 1988, *Rzedowski 46601* (CAS, DAV, IEB); Mpio. Pinal de Amoles, El Puente Blanco, 12 km al E de Jalpan, carretera Jalpan-Pinal de Amoles, [21.1750, -99.4083], 1170 m, 21 Oct 1982, *Tenorio-L. 2310* (MEXU, NY); **Sonora**: Río Mayo region, Arroya Tepopa, about 35 km NE of Alamos, “main fork” of canyon above confluence with “waterfall fork,” above old ranch site, 27.3333, -108.7333, 1100 m, *M. Fishbein 1040* (NY).

**47. *Lycianthes textitlániana* – none; only known from the type collection**

**48. *Lycianthes tricolor***

**El Salvador. Ahuachapán**: Ahuachapán, Cerro Cachío, 13.9, -89.7333, 1300 m, 11 Dec 1996, *Renderos 111* (MO); **La Paz**: San Pedro Nonualco, Cantón Amulungo, 13.6, -88.9333, 13 Aug 1989, *González 525* (MO); **Santa Ana**: El Comun, northern slopes of Santa Ana Volcáno, 7000 ft, 25 Sep 1958, *Allen 6951* (NY, LL); entre el Olimpo y el Ojo de Agua de la Virgen cerca del Cerro El Aguila, [13.8923, -89.6743], m, 7 Dec 1993, *Linares 1210* (MEXU); Cerro El Aguila, [13.9, -89.7], 1925 m, 18 Feb 2007, *Rodríguez 749* (MEXU); [border of Depts. Santa Ana and Sonsonate], Cerro del Aguila, 13.9, -89.7, 1710 m, 28 Apr 1942, *Tucker 1315* (G); **Sonsonate**: Finca El Olimpo, 13.8991, -89.6949, m, 19 Nov 2007, *Linares 12758* (MEXU).

**Guatemala. State unknown**: walper vulkan bei S. María, 7000 ft, Aug 1894, *Raimann s.n.* (W); **Chimaltenango**: 9 mi W of San Miguel Dueñas on road to Yepocapa, at Tres Cruces on N side of Volcán Acatenango, [14.5403, -90.8889], 2500 m, 5 Aug 1960, *Beaman 4008* (DUKE, GH, TEX); S. Martin Jilotepeque, Feb 1878, *Bernoulli 2362* (GOET); Mpio. Tecpán Chimaltenango, Reserva Natural Privada El Encanto de Tecpán, Caserlo Panimachavac, Aldea Chajaljya, 14.8142, -90.9872, 2317 m, 8 Apr 2014, *Escobar BIEA87* (BIGU); in “Mano del Mico” forest, 4 km S of (San José) Calderas, [14.5317, -90.9081] , 2600 m, 11 Jun 1971, *Harmon w5647* (MO); cerca de San Bartolo, [14.5500, -91.3333], 1700 m, 18 May 1963, *Molina-R. 12436* (NY); on N slopes of Volcán Acatenango, 2 km W of San José Calderas, 14.5333, -90.8833, 2300 m, 10 Sep 1997, *Nee 47223* (NY); Volcán de Acatenango,14.5403, -90.8889, 2600 m, 22 May 1993, *Sajtoj 93-3050* (MEXU); above Las Calderas, 1800–2100 m, *Standley 60069* (A); along road between Tecpán and Los Encuentros, [14.8247, -91.0106], 3000 m, 1 Jul 1942, *Steyermark 48088* (NY); **Esquintla**: Volcán de Agua of Finca Rosario de La Vista Hermosa, [14.4411, -90.7506], 6000–8000 ft, 22 May 1971, *Wilbur 14748* (DUKE); **Guatemala**: 7 mi E of Guatemala City, [14.5144, -90.4756], 1840 m, 5 Jul 1970, *Harmon 2931* (MO); 15 km SE of Guatemala (City) on CA-1, [14.3911, -90.5696], 22 Jul 1970, *Harmon 3227* (MO); along old road to San Lucas, vicinity of San Rafael, 1800 m, 27 Sep 1972, *Molina 27614* (MEXU, TEX); **Huehuetenango**: Chanximil, Aldea San Martín, Todos Santos Cuchumatán, 15.5531, -91.6986, 2185 m, 16 Sep 2006, *Morales 3889* (MO); vicinity of Quetzal, 15.8155, -91.3761, 2300 m, 10 Jul 1942, *Steyermark 48514* (NY); Sierra de los Cuchumatanes, above San Juan Ixcoy, 15.5980, -91.4489, 2400 m, 4 Aug 1942, *Steyermark 49978* (NY); Cumbre Papal, between summit and La Libertad, [15.5128, -91.8689], 1800–3000 m, 19 Aug 1942, *Steyermark 50958* (NY, MO); Finca Injerto, 15.5644, -91.9153, 2468 m, 6 Aug 2013, *Velásquez 3731* (BIGU); **Quezaltenango**: Volcán Zunil, 6–8 km S of Zunil along road to Fuentes Georginas, [14.7463, -91.4254], 2375 m, 2 Oct 1986, *Breedlove 64803* (CAS, NY, TEX); ca. 60 miles E of Quetzaltenango, [14.8561, -91.5936], 21 Sep 1978, *D’arcy 12112* (MO); Mpio. Zunil, along dirt road from Aldea Chuimucuba, NW slopes of Volcán Zunil, [14.7653, -91.4689], 2523 m, 10 May 2007, *Quedensley 5121* (BIGU); **Quiché**: Nebaj, in clearing, on rocky hill, about 4 km west, 6700 ft, 11 Jun 1964, *Contreras 4953* (MO, LL); Nebaj, about 8 km S on Sacopulos road, [15.4058, -91.1461], 7000 ft, 12 Jun 1964, *Contreras 4960* (F, LL, MO); Nebaj, about 12 km west, 8000 ft, 4 Jun 1964, *Contreras 5198* (LL); hwy to Chichicastenango, between El Tesoro and Chicuas, 2000 m, 1 Dec 1969, *Molina-R. 25024* (NY); El Molino, 4 km from Chichicastenango, [14.9186, -91.1000], 2100 m, 14 Jan 1974, *Molina-R. 30308* (MO, NY); El Boquerón, [15.4186, -91.1469], 8000–8200 ft, 10 Aug 1964, *Proctor 25468* (LL, MO); Nebaj, [15.4058, -91.1461], 7000 ft, 21 Nov 1934, *Skutch 1750* (A); **Sacatepequez**: Volcán de Agua, [14.4694, -90.7397], 2500 m, 5 Mar 1994, *Bill 94-3622* (BIGU); Volcán de Agua, along road between Santa María de Jesús and crater, 14.4569, -90.7403, 2800–3300 m, 7 Jul 1986, *Diggs 4047* (NY, TEX); Volcán de Agua, [14.4708, -90.7392], 2500 m, 15 Aug 2003, *García 26* (BIGU, MEXU, TEX); Mpio. San Lucas Sacatépequez, Cerro Alux, Finca Lourdes, [14.6108, -90.6297], 2000 m, 23 Jun 2009, *Maza 59* (BIGU); Volcán Agua between Sta. María de Jesús and San Juan Obispo, 14.4805, -90.7235, 1700 m, 27 Nov 1969, *Molina-R. 24892* (G, MO); **San Marcos**: 20 miles S of San Marcos along road from San Raphael, 2100 m, 13 Jul 1977, *Croat 41006* (MO); Sierra Madre Mountains, about 6 km N of San Marcos, [15.0246, -91.8093], 2700 m, 13 Dec 1963, *Williams 25835* (NY); Sierra Madre Mountains, outer slopes of Tajumulco Volcáno, 8–10 km of W of San Marcos, [15.0841, -91.7899], 2300 m, 31 Dec 1964 to 1 Jan 1965, *Williams 26962* (MO); **Sololá**: S of Lake Atitlán, 1.5 km S Panajachel, [14.6417, -91.1692], 2200 m, 8 May 1972, *Burch* 5991 (MO); 6 km SE of Nahualá, along Hwy CA 1, 14.8000, -91.3000, 2400m, 17 Sep 1997, *Nee 47274* (MEXU, NY); Mpio. San Pedro La Laguna, Volcán San Pedro, [14.6564, -91.2672], 2500 m, 27 Jul 2005, *Pardo 665* (BIGU).

**Mexico. Chiapas**: Mpio. Motozintla de Mendoza, 11 km NW of the junction of the road to Motozintla along road to El Porvenir and Siltepec, SW side of Cerro Mozotal, [15.4195, -92.3368], 2100 m, 27 Jun 1972, *Breedlove 25699* (LL, MEXU, MO, NY); Mpio. Motozintla de Mendoza, 11 m NW of the junction of the road to Motozintla along road to El Porvenir and Siltepec, SW side of Cerro Mozotal, [15.4195, -92.3368], 2100 m, 18 Sep 1976, *Breedlove 40257* (MO); same location and elevation, 22 Nov 1976, *Breedlove 41757* (MO); same location and elevation, 13 Oct 1980, *Breedlove 46220* (MO); same location and elevation, 30 Jan 1982, *Breedlove 58135* (LL, MEXU, MO, NY); Reserva de la Biosfera El Triunfo, sendero Finca Prusia-Campamento El Triunfo, 22.1500, -99.5667, 1900 m, 12 Nov 2004, *Martínez-Melendrez 587* (MEXU); Mpio. Unión Juárez, en el Volcán Tacaná, entre Talquian y la cima del volcán, [15.0935, -92.0991], 1600–2400 m, 19 Jun 1985, *Martínez-S. 13187* (NY, MEXU MO); same location, elevation, and date, *Martínez-S. 13206* (MEXU, MO, NY); Mpio. Unión Juárez, en el Volcán Tacaná por el camino de Talquián a la cima del volcán, por la línea divisoria con Guatemala, [15.1303, -92.1076], 1700–2200 m, 4 Feb 1987, *Martínez-S. 19409* (MEXU); Mpio. Unión Juárez, en el Volcán Tacaná, camino entre Talquián y Chiquihuite, [15.1303, -92.1076], 1800 m, 28 Apr 1987, *Martínez-S. 20458* (BIGU, M, MEXU, MO); Mpio. Unión Juárez, entre Talquián y la línea divisoria con Guatemala, [15.0532, -92.0767], 1700–2300 m, 1 May 1987, *Martínez-S. 20616* (BIGU, MEXU); Mpio. Motozintla de Mendoza, Boqueron, near Motozintla, 2540 m, 3 May 1945, *Matuda 5373* (LL); hills E of Unión Juárez, lower slopes of Volcán Tacaná, 15.1000, -92.0833, 1700–2300 m, 3 May 1987, *Miller 2661* (DAV, MEXU, MO); Mpio. Jaltenango, Reserva de la Biosfera El Triunfo, [15.6807, -92.8047], 2050 m, 16 Jun 1994, *Ramírez-Marcial 490* (MEXU); Mpio. Mazapa de Madero, Granados Tlalcanaque, [15.3943, -92.1797], 2350 m, 19 May 1986, *Ventura 3655* (NY, WIS, XAL); Mpio. Unión Juárez, 5 km al NE de Unión Juárez, 15.0945, -92.0948, 1700 m, 10 Aug 1986, *Ventura 4077* (IEB); Mpio. Unión Juárez, Chiquihuite, 12 km al sureste de Unión Juárez, 15.0552, -92.0771 3000 m, 6 May 1988, *Ventura 5191* (TEX); **Guerrero**: below Puerto El Gallo, along road to Atoyac, [17.4767, -100.1800], 2255 m, 10 Oct 1986, *Breedlove 65113* (MEXU, NY); near Puerto El Gallo, W slope of Cerro Teotepec, [17.4767, -100.1800], 2290 m, 20 Oct 1984, *Breedlove 61977* (MO, MEXU); Puerto Pichones, [17.6723, -99.7482], 2450 m, 4 Jun 1980, *Fonseca 41* (MEXU); Mpio. Chilpancingo de los Bravo, Región Centro, Omiltemi, Barranca Potrerillos, [17.5595, -99.6759], 2170 m, 28 Nov 1993, *González 241* (MEXU); Mpio. Leonardo Bravo, El Carrizal, 4.5 km al SE, camino a Puerto El Jilguero, 2480 m, 24 Oct 1985, *Loera- H. 3591* (XAL); **Jalisco**: Mpio. Villa Purificacion, parte alta del ejido de Pabelo, cerca del predio Las Iglesias, 4.4 km al sur-suroeste de Santa Monica (Mpio. Ayutla) y 4 km al este-noreste de Plaza de Gallos, 19.8922, -104.5464, 2120–2160 m, 12 Aug 2012, *Carrillo-Reyes 6743* (IBUG, IEB, MEXU); N end of Sierra de Manantlán Central, 17.7 km S of El Chante, along lumber road, 0.3 km E of fork Cerro La Cumbre/ Ricon de Manantlán, 19.5583, -104.2208, 2250 m, 6 Jan 1980, *Iltis 2337* (MEXU, TEX); in headwaters of Arroyo Las Joyas, ca 1.5 km E of Las Joyas Biológical Station, 22 km SSE of Autlán, on road to Puerto San Campus, 19.5833, -104.2611, 2040 m, 12 Jun 1994, *Iltis 31127* (DAV, WIS); **México**: District Temascaltepec, Rincón. [Note that duplicates at other institutions are *L. arrazolensis*. This specimen has shallowly notched seeds, hairs that match L. tricolor and long pedicels.], [18.9017, -100.1200], 1960 m, 27 Dec 1933, *Hinton 3029* (G); **Michoacán**: Sierra de Coalcomán, 9.5 km SW of Ejido Varaloso, along road from Dos Aguas to Barranca Seca, [18.8414, -102.9612], 2130 m, 18 Sep 1986, *Breedlove 64456* (MEXU, MO, NY); Mpio. Coalcomán, 14 km al SE de Varaloso, sobre el camino a Barranca Seca, 18.6536, -102.9769, 2000 m, 22 Dec 2007, *Steinmann 6121* (DAV); **Oaxaca**: Dto. Santiago Juxtlahuaca, Mpio. San Sebastián Tecomoxtlahuaca, Sabino Solo, 5 km de San Sebastián Tecomoxtlahuaca, carretera Coicoyán de las Flores, 17.3167, -98.0500, 1710 m, 6 Oct 1994, *Calzada 19385* (NY); Dto. Santiago Juxtlahuaca, Mpio. San Sebastián Tecomoxtlahuaca, 5 km de San Martín Duraznos, desviación a Sierra de Lumbre, 17.2667, -98.0833, 2130 m, 20 Dec 1995, *Calzada 20701* (MEXU); Mpio. San Felipe Usila, Cerro de San Felipe, [17.8803, -96.5392], 2000 m, 29 Aug 1897, *Conzatti 442* (GH, MEXU); Mpio. Sta. María Jaltianguis, town of Sta. María Jaltianguis, 17.3640, -96.5283, 7200 ft, 20 Oct 1991, *Dean 297* (DAV, MEXU); NO de la Natividad, camino al Polvorin, 17.3103, -96.4306, 2150 m, 9 Oct 2002, *Figueroa-Brito 357* (MEXU); Dto. Ixtlan, NE del Río Natividad, brecha que sale del aserradero a 1 km, 17.3238, -96.4445, 29 Oct 2003, *Figueroa-Brito 897A* (MEXU); Dto. Juxtlahuaca, cañada del Río San Lucas, 1 km al noroeste de San Lucas, 1900 m, 22 Oct 1990, *García-Mendoza 5122* (MEXU); Mpio. Miahuatlán, Chanaguilla, [16.3327, -96.5956], above 2500 m, 5 Jun 1996, *Hinton 26622* (IEB, LL, NY); Cerro de Carion, San Andres Chicahuastla, [17.1577, -97.8584], 29 Mar [year missing], *MacDougall s.n.* (MEXU); Dto. Putla, Mpio. Santa Cruz Itundujia, Hidalgo, Paraje “El Peligro,” 16.8431, -97.6664, 2301 m, 22 Jul 2007, *Nava-Zafra 2364* (MO); Sierra de San Felipe, 8000 ft, 14 Jun 1894, *Pringle 5666* (GH); Dto. Juxtlahuaca, Mpio. San Juan Mixtepec, Cañada de San Lucas, 8 km O de Cabecera Municipal de San Juan Mixtepec, [17.3085, -97.8862], 1900–2000 m, 9 Nov 1988, *Reyes-S. 1117* (MEXU); Mpio. San Juan Mixtepec, Peña Blanca, 12 km oeste de San Juan Mixtepec, 17.3066, -97.9324, 2100 m, 10 Jul 1988, *Reyes-Santiago 361* (MEXU); Dto. Juxtlahuaca, Yuu nii (cañada antigua), a 9 km al E de San Juan Mixtepec, 17.2833, -97.7500, 1780 m, 23 Jul 1989, *Reyes-Santiago 1779* (MEXU); 6 km SO de Cuquila, carr. Tlaxiaco-Putla, [17.2192, -97.7646], 2000 m, 8 Jun 1985, *Torres-C. 6700* (MEXU); Dto. de Ixtlán, Mpio. Santiago Laxopa, S. Laxopa, 17.5000, -96.5000, 2000 m, 15 Oct 1987, *Maldonado-Vásquez 205* (NY); Dto. Putla, Mpio. Santa Cruz Itundujia, La Torre a 4.78 km en línea recta (S) de Santa Cruz Itundujia, 16.8319, -97.6703, 2336 m, 20 Jun 2008, *Velasco-Gutiérrez 2828* (MEXU); Mpio. Santiago Textitlán, Solo de Vega, 16.7061, -97.2344, 2698 m, 26 Aug 2006, *Zarate-Marcos 346* (IEB); Dto. Solo de Vega, Mpio. Santiago Textitlán, Peña Ahumada, 16.7256, -97.0781, 2 Sep 2006, *Zarate-Marcos 407* (IEB).

**49. *Lycianthes venturana***

**Mexico. Puebla**: Mpio. Zacapoaxtla, Hueyaktepet, vicinity of San Juan Tahitic, trail to Zapotepic, next to Arroyo de Huaxkonta, 19.9503, -97.5311, 1165 m, 2 Nov 2017, *Croat 107297* (DAV); Mpio. Ahuacatlán, 4.5 km al SE de Ahuacatlán, brecha a Zapotitlán, 20.00, -97.8166, 1250 m, 24 May 1986, *Tenorio-L. 11426* (GH, MEXU); Mpio. Teziutlán, Puente Colorado, carretera Teziutlán-Tlapacoyan, [19.8347, -97.3414], 1450 m, 24 Jun 1970, *Ventura-A. 1350* (IEB, MEXU, MO, XAL); Mpio. Hueytamalco, Rancho El Milagro, [20.1095, -97.2988], 1350 m, 27 May 1986, *Ventura-A. 21982* (IEB, MEXU, XAL); **Veracruz**: Cañada del Huerfano, 1450 m, 9 Jul 1966, *Gómez-Pompa 1574* (XAL); Mpio. Atzalán, El Siete, 19.80, -97.2166, 1445 m, 10 Jun 2008, *Krömer 3556 (or 2556?)* (MEXU MO,); Mpio. Huatusco, 3 km E of Tenejapa, 11 Apr 1948, *Langman 3631* (US); Mpio. Chiconquiaco, Santa Julia, 19.8166, -96.8166, 30 May 1981, *Pedraza-C. 268* (XAL).
